# A Multi-Omics Approach to Evaluate the Toxicity Mechanisms Associated with Silver Nanoparticles Exposure

**DOI:** 10.3390/nano12101762

**Published:** 2022-05-22

**Authors:** Guillermo Aragoneses-Cazorla, M. Pilar Buendia-Nacarino, Maria L. Mena, Jose L. Luque-Garcia

**Affiliations:** Department of Analytical Chemistry, Faculty of Chemical Sciences, Complutense University of Madrid, 28040 Madrid, Spain; guiarago@ucm.es (G.A.-C.); mabuen01@ucm.es (M.P.B.-N.); mariluz@ucm.es (M.L.M.)

**Keywords:** silver nanoparticles, metabolomics, transcriptomics, toxicity mechanisms, oxidative stress, metabolic pathways

## Abstract

Silver nanoparticles (AgNPs) are currently used in many different industrial, commercial and health fields, mainly due to their antibacterial properties. Due to this widespread use, humans and the environment are increasingly exposed to these types of nanoparticles, which is the reason why the evaluation of the potential toxicity associated with AgNPs is of great importance. Although some of the toxic effects induced by AgNPs have already been shown, the elucidation of more complete mechanisms is yet to be achieved. In this sense, and since the integration of metabolomics and transcriptomics approaches constitutes a very useful strategy, in the present study targeted and untargeted metabolomics and DNA microarrays assays have been combined to evaluate the molecular mechanisms involved in the toxicity induced by 10 nm AgNPs. The results have shown that AgNPs induce the synthesis of glutathione as a cellular defense mechanism to face the oxidative environment, while inducing the depletion of relevant molecules implicated in the synthesis of important antioxidants. In addition, it has been observed that AgNPs completely impair the intracellular energetic metabolism, especially affecting the production of adenosine triphosphate (ATP) and disrupting the tricarboxylic acids cycle. It has been demonstrated that AgNPs exposure also affects the glycolysis pathway. The effect on such pathway differs depending on the step of the cycle, which a significant increase in the levels of glucose as way to counterbalance the depleted levels of ATP.

## 1. Introduction

The use of AgNPs is continuously increasing due to their unique physical, chemical and biological properties [1,2,3]. Due to their antibacterial capacity especially, they have been used in wide range of fields, including the medical industry, where AgNPs are currently used as medical device coatings and in wound dressings. Moreover, AgNPs are routinely used as textile coatings and employed in the food industry to preserve food [4,5,6]. The wide extension in the use of AgNPs increase the exposure of the human body to these nanomaterials, whether it is respiratory, oral or skin exposure [7]. Although silver soluble compounds have been proved to induce kidney and liver damage, only limited studies have been conducted to evaluate the toxicity of AgNPs despite the extended use of these nanomaterials [8]. In these toxicity studies carried out so far, both in vitro and in vivo, AgNPs have been reported to induce DNA damage [9], oxidative stress [10], mitochondrial membrane potential impairment [11] and to be toxic to the liver among other organs [7]. In a previous work, our group proved the cytotoxicity associated with AgNPs exposure and carried out a SILAC-based quantitative proteomics assay, where it was demonstrated that 10 nm AgNPs induce nucleolar stress, ribosome biogenesis halt, DNA damage and oxidative stress through different pathways [12]. Nevertheless, the information provided by our study, or by similar studies so far, lacks information at the genome and metabolome levels, thus resulting insufficient evidence to fully elucidate the specific cellular pathways by which AgNPs exert their toxic action.

Metabolomics is a powerful tool which has been employed in the study of the toxicity of nanomaterials to elucidate toxicological pathways and to identify biomarkers of cause and/or effect of the toxic agent. This approach allows the identification of very different metabolites such as hormones, carbohydrates, amino acids or fatty acids among others. There are two possible metabolomics approaches: untargeted metabolomics, which provides a wide list of metabolites which are relative or absolutely quantified; and targeted metabolomics, which aim to absolutely quantify a limited number of specific known analytes. Although untargeted metabolomics approaches allow the identification of known and unknown metabolic changes, the data analysis only leads to the formulation of different hypothesis which need to be validated with, among others, targeted metabolomics approaches. Thus, the combination of targeted and untargeted approaches enables the identification and confirmation of changes in specific metabolic processes after exposure to xenobiotic agents [13,14,15,16]. On the other hand, transcriptomics and, specifically, the microarrays technique, allows the detection of the differential expression of almost all genes that can be found in a biological sample after exposure to any xenobiotic agent. This technology is based on the extraction of the mRNA, which is transformed into cDNA, which, in turn, is labeled with a fluorescence probe and then hybridized with its corresponding target [17,18]. Therefore, the integration of both techniques allows for the elucidation of the status of different metabolic pathways and, thus, provides a better understanding of the biological processes that are involved after a cytotoxic insult [19,20].

Although a metabolomics analysis has already been carried out in aneuploidy immortal keratinocyte (HaCaT) cells exposed to AgNPs with different sizes and coatings, it did not elucidate complete metabolic pathways, neither evaluated energetic metabolites levels after exposure to these agents [21]. In addition, while a DNA microarrays transcriptomics analysis has also been reported after exposure to 16 nm AgNPs in adenocarcinomic human alveolar basal epithelial (A549) cells, it only reported the effect of the nanoparticles on cell cycle progression and regulation genes and other transcripts involved in the oxidative stress response, while the effects on other metabolic pathways remained unknown [22]. To the best of our knowledge, an in vitro study of AgNPs toxicity in human cells combining both strategies is yet to be performed. For this reason, and as a continuation of a previous study [12], in the present study, we have combined untargeted and targeted metabolomics approaches with the microarrays technology to assess the effect of AgNPs on cellular metabolism and elucidate different impaired metabolic pathways after exposure to these agents in human hepatocellular carcinoma (HepG2) cells. To carry out this study, 10 nm AgNPs have been selected since, according to previous studies [12], they have a higher toxicity compared to larger nanoparticles.

## 2. Materials and Methods

### 2.1. Nanoparticles: Physicochemical Characterization and Stability

Commercial AgNPs of 10 nm of diameter suspension stabilized with 2 mM sodium citrate were purchased from NanoComposix (San Diego, CA, USA). The characterization of the nanoparticles was carried out with the following analytical techniques: transmission electron microscopy (TEM—JEOL JEM 1400), electrophoretic mobility measurements to calculate the values of the zeta-potential (ζ-potential—Zetasizer Nano ZS (Malvern Instruments Ltd., Malvern, United Kingdom)) and dynamic light scattering (DLS—Zetasizer Nano ZS (Malvern Instruments Ltd., United Kingdom). To evaluate the stability of AgNPs, 10 mL of cell culture media containing 5 mg/L of AgNPs was incubated at 37 °C in 5% CO_2_. After 72 h, the media was ultra-filtrated using Amicon 3 K ultra-centrifugal filters and the total Ag content released by the NPs was determined in the filtrate by inductively coupled plasma mass spectrometry (ICP-MS—Agilent) (*n* = 5).

### 2.2. Cell Culture and Treatment

HepG2 cell line was maintained in Dulbecco’s modified Eagle’s medium (DMEM), supplemented with 10% fetal bovine serum (FBS) and 100 unit/mL penicillin/streptomycin at 37 °C and 5% CO_2_. Cells were exposed to 5 µg/mL of 10 nm AgNPs for 72 h.

### 2.3. Cytotoxicity Assays

For the cell viability assay, HepG2 cells were seeded in a 96 well plate and incubated with AgNPs at concentrations ranging from 0.1 to 10 µg/mL for 72 h at 37 °C in 5% CO_2_. Then, 20 µL of MTT reagent (3-(4,5-dimethyl-thiazol-2-yl)2,5-diphenyl tetrazolium bromide) were added to each well and incubated for 5 h at 37 °C in 5% CO_2_. The media was then removed, and the generated formazan crystals were dissolved in 100 mL of dimethyl sulfoxide. Absorbance was measured at 595 nm in a microplate reader (Sunrise, Tecan).

For the cell death assay, HepG2 cells were seeded on 6 well plates and incubated with AgNPs at 5 µg/mL for 72 h at 37 °C in 5% CO_2_. Cells were then harvested, stained with a 0.4% trypan blue solution and counted (live and dead) using a Neubauer chamber.

### 2.4. Targeted Metabolomics

HepG2 cells were seeded into P100 plates and exposed to 5 µg/mL of AgNPs for 72 h. After this time, the four main energy-related metabolites: adeonisine triphosphate (ATP), adenosine diphosphate (ADP), 1,4-dihydronicotinamide adenine dinucleotide (NADH) and nicotinamide adenine dinucleotide (NAD+), were extracted. Cells were washed with 10 mL of 0.9% sodium chloride (NaCl, Fisher Scientific, Waltham, MA, USA), and 100 µL of methanol (Fisher Scientific, Waltham, MA, USA) at −20 °C were added to the cells before scrapping. Then, 400 µL of ice-cold 0.4% formic acid (*v/v*) (Fisher Scientific, Waltham, MA, USA) were added, cells were re-suspended, transferred to a 1.5 mL Eppendorf^TM^ tube (Fisher Scientific, Waltham, MA, USA), and vortexed for 1 min. After 3 min of incubation on ice, 45 µL of 15% ammonium bicarbonate (NH_4_HCO_3_ (*w*/*v*), Fisher Scientific, Waltham, MA, USA) were added to neutralize the pH of the samples. Samples were then vortexed for 1 min and incubated during 20 min on ice. Prior to the liquid chromatography-mass spectrometry (LC-MS) analysis, samples were centrifuged at 16,000× *g* and 4 °C for 10 min, and the supernatant was filtered through a Whatman^®^ PTFE membrane filters (pore size 0.22 µm, Merck, Darmstadt, Germany). Total protein concentration of each sample was calculated by means of the Bradford assay, thus allowing further normalization of the concentration of each metabolite. The LC-MS analysis was carried out in Multiple Reaction Monitoring (MRM) mode as previously described [23].

Differences in ATP, ADP, NADH and NAD+ contents between control and cells exposed to AgNPs were assessed by ANOVA statistical analysis at 95% confidence level (*p* value < 0.05), followed by Bonferroni test.

### 2.5. Untargeted Metabolomics

HepG2 cells were exposed to 5 µg/mL of AgNPs for 72 h. After exposure time, cells were washed with 10 mL of 0.9% NaCl. The metabolic cycle was quenched by adding 400 µL of methanol at −20 °C and 400 µL of ice-cold water (Fisher Scientific, Waltham, MA, USA). Then, cells were scraped and transferred into 1.5 mL tubes. Metabolites extraction was carried out by adding 400 µL of chloroform (Sigma-Aldrich, St. Louis, MI, USA) at −20 °C. Afterwards, tubes were agitated in a tube shaker for 20 min at 1400 rpm and 4 °C, and then centrifuged at 16,000× *g* and 4 °C for 5 min. After separation of the polar and non-polar phases, 300 µL from each phase were transferred to different glass vials and the protein interphase was isolated for further quantification, employing the Bradford method. After adding to both phases 50 mg/L of 4-chloroplenylalanine (Sigma-Aldrich, St. Louis, MI, USA) as internal standard, extracts were evaporated under a nitrogen stream. Chemical derivatization of metabolites for further gas chromatography-mass spectrometry (GC-MS) analysis was performed by adding 30 µL of 50 mg/L methoxyamine hydroxychloride in pyridine (Sigma-Aldrich, St. Louis, MI, USA), followed by mixing at 500 rpm for 90 min at 37 °C. Subsequently, 60 µL of *N*,*O*-Bis(trimethylsilyl)trifluoroacetamide (BTSFA) (Sigma-Aldrich, St. Louis, MI, USA) containing 1% trimethylsilyl chloride (TMCS) (Sigma Aldrich, St. Louis, MI, USA) was added and incubated at 60 °C by mixing at 500 rpm for 1 h [22]. Gas chromatography time-of-flight mass spectrometry (GC-TOF-MS) analysis was carried out using the conditions previously described [24].

Chromatographic data were analyzed with the Mass Lynx software. The peak area of each metabolite was normalized with the corresponding internal standard area. Metabolites were identified based on both mass spectra and accurate mass using the NIST MS search 2.0 library.

### 2.6. Transcriptome Analysis

Cells were seeded in P100 plates for 24 h, and then exposed to 5 µg/mL of AgNPs for 72 h. After the exposure time, mRNAs were extracted using the PureLink^®^ Invitrogen commercial kit (Invitrogen, Waltham, MA, USA). The purified RNA was stored at −80 °C for further analysis. Samples were processed with GeneChip^®^ WT PLUS Reagent Kit (Applied Biosystems, Waltham, MA, USA), hybridized with Clariom^TM^ D Array, human (Applied Biosystems, Waltham, MA, USA) and scanned with a GeneChip^®^ Scanner 3000 7G (Applied Biosystems, Waltham, MA, USA). Raw data were processed with an RMA algorithm included in Transcriptome Analysis Console (Applied Biosystems, Waltham, MA, USA) for normalization and gene levels analysis. For each experimental condition (control and cells exposed to AgNPs), three microarray experiments corresponding to three independent biological RNA replicates were processed and analyzed. Fold changes between both experimental conditions were calculated as a quotient between the mean of the gene expression signals. Statistical analysis was performed with e-bayes limma, included in the Transcriptome Analysis Console (Applied Biosystems, Waltham, MA, USA).

## 3. Results

### 3.1. Physicochemical Characterization and Stability of the AgNPs

**Figure 1:**
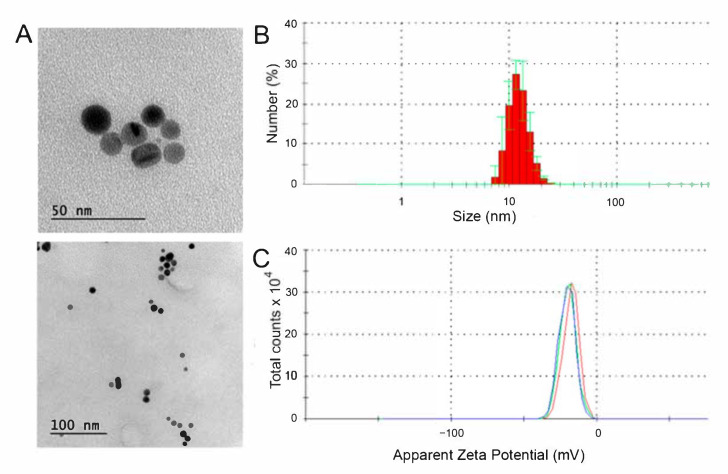
Characterization of the AgNPs: (**A**) TEM Micrographs; (**B**) Hydrodynamic size distribution; (**C**) ζ–potential.

TEM micrographs (Figure 1A) showed well dispersed AgNPs that exhibit spherical shape (ca. 10 nm) and good monodispersity (PDI = 0.273). The hydrodynamic size distribution of the AgNPs (Figure 1B) was monomodal and reasonably narrow (with a maximum centered approximately at 15 nm), which is in accordance with the size determined by TEM. In addition, ζ-potential measurements (Figure 1C) displayed a negative charge of ca. −18 ± 5 mV, which is in the zone of colloidal stability. Although the nanoparticles were commercial, the chemical composition was evaluated by EDX (Figure S1 in Supplementary Materials), where the presence of Ag was confirmed.

In order to evaluate the potential dissolution of the nanoparticles during the incubation time, the total amount of Ag in the culture medium after ultra-filtration was determined by ICP-MS at two times: 0 and 72 h. While the amount of Ag found at 0 h was below the detection limit of the technique, the Ag concentration found in the filtrates after 72 h of incubation was below 0.4 ± 0.03%, which can be considered negligible and confirms the stability of the AgNPs under the exposure conditions.

### 3.2. Cytotoxicity of AgNPs

**Figure 2:**
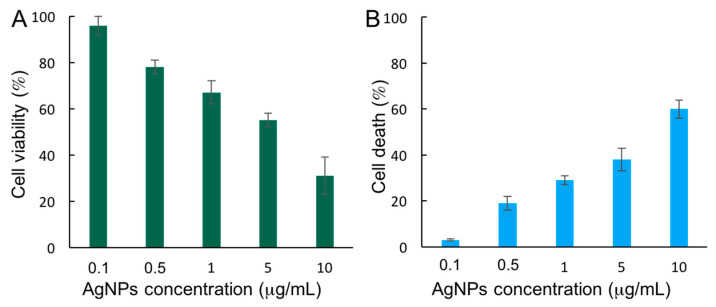
Cytotoxicity of AgNPs: (**A**) MTT-based cell viability assay; (**B**) Trypan blue-based cell death assay.

To evaluate the cytotoxic effect of AgNPs, cell viability (Figure 2A) and cell death (Figure 2B) assays were carried out in HepG2 cells exposed to 5 µg/mL AgNPs for 72 h. While the cell viability was estimated by means of the MTT assay, which measures the reducing potential of cells, the induction of cell death was evaluated using a trypan blue, a dye that can only penetrate and stain cells with a compromised nuclear membrane, meaning they are undergoing cellular death. The results showed that cell viability decreases with increasing AgNPs concentration, as expected (Figure 2A). The potential toxic effect of the AgNPs stabilizer citrate was also evaluated. As can be seen in Figure S2 (in Supplementary Materials), citrate did not alter cell viability at any of the concentrations tested, thus confirming that the observed toxic effect was exclusively due to AgNPs. Interestingly, the cell death measurements performed (Figure 2B) showed that the observed decrease in cell viability corresponds to the increase in cell death. Based on these results, 5 µg/mL AgNPs was selected as exposure conditions for further experiments, since it decreases the cell viability but without drastically compromising it.

### 3.3. Targeted Metabolomics

**Figure 3:**
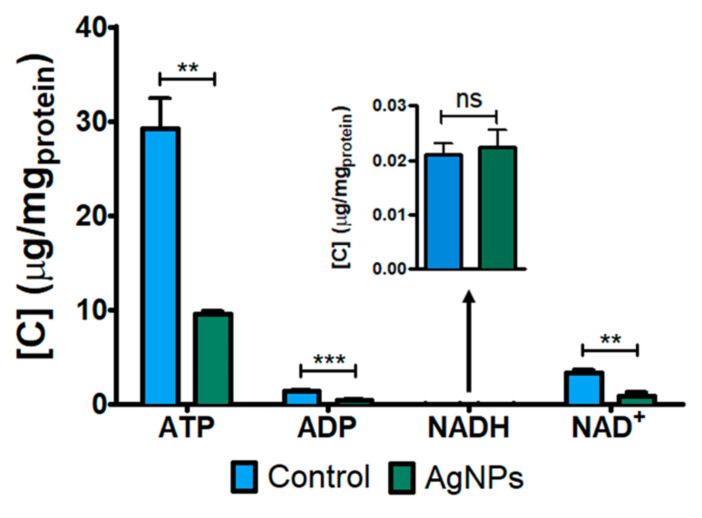
Intracellular levels of ATP, ADP, NADH and NAD+ metabolites in HepG2 cells after exposure to 5 µg/mL of 10 nm AgNPs for 72 h. Data were analyzed by ANOVA followed by Bonferroni’s multiple-comparison test. Statistical significance: ** *p* < 0.005. *** *p* < 0.001.

In order to evaluate the potential toxic effect of AgNPs on the energy machinery of HepG2 cells, the levels of the main energy-related metabolites (ATP. ADP, NADH and NAD+) were evaluated using targeted metabolomics. As shown in Figure 3, after exposure to 5 µg/mL AgNPs for 72 h, the levels of ATP, ADP and NAD+ in HepG2 cells were found significantly diminished compared to those in the control cells. NADH levels remained unaltered at the tested conditions.

### 3.4. Untargeted Metabolomics Results

A GC-MS-based untargeted metabolomics approach was carried out in order to identify additional metabolites with concentration levels in HepG2 cells that could have been affected by exposure to AgNPs. A total of 28 common metabolites were quantified between controls and cells exposed to AgNPs (Table S1 in Supplementary Materials). To ensure the reliability of the identification, a minimum NIST Rmatch value of 700 was considered, achieving the correct identification of metabolites of different nature: amino acids, fatty acids, sugars, organic acids and other inorganic and organic compounds. In addition, the internal standard was unequivocally identified in all the samples. The similarity between metabolites and, thus, their relation, is usually expressed using Pearson correlation indexes. Critical values of linear Pearson correlation coefficient (r), for 95% of confidence, should be considered 0.334 and 0.312 for *n* = 35 and *n* = 40, respectively [25]. According to these values, a lot of metabolites would be correlated; however, in order to set a stricter criterion, only those with r values higher than 0.6 were considered (Table S2 in Supplementary Materials). Among these data, malic acid and L-tyrosine (r = 0.8991); xylofuranose and L-proline (r = 0.8019); L-leucine and L-proline (r = 0.8623); L-leucine and L-cysteine (r = 0.8286); L-leucine and phosphoric acid (r = 0.8946); L-leucine and erythrose (r = 0.8651); L-proline and L-cysteine (r = 0.9361); L-proline and phosphoric acid (r = 0.9339); L-proline and L-threonine (r = 0.9197), L-proline and erythrose (r = 0.8851), L-cysteine and phosphoric acid (r = 0.9734), L-cysteine and L-threonine (r = 0.9245), L-cysteine and threitol (r = 0.8421), L-cysteine and erythrose (r = 0.9444), phosphoric acid and L-threonine (r = 0.8816), phosphoric acid and threitol (r = 0.8631), phosphoric acid and erythrose (r = 0.9548), L-threonine and threitol (r = 0.8264), L-threonine and erythrose (r = 0.8901), threitol and erythrose (r = 0.9666), threitol and D-fructose (r = 0.8484), threitol and pantothenic acid (r = 0.8772), threitol and β-Aminoisobutyric acid (r = 0.8187), D-fructose and pantothenic acid (r = 0.8636), glutamine and myo-inositol phosphate (r = 0.8330), glutamine and octadecanoic acid (r = 0.8290), glutamine and L-aspartic acid (r = 0.8568), glutamine and myo-inositol (r = 0.8466), myo-inositol phosphate and octadecanoic acid (r = 0.8286), myo-inositol phosphate and L-aspartic acid (r = 0.8704), myo-inositol phosphate and myo-inositol (r = 0.8196), octadecanoic acid and L-aspartic acid (r = 0.8023), octadecanoic acid and myo-inositol (r = 0.8719) and L-aspartic acid and myo-inositol (r = 0.9675) are worth noting, since they showed a high positive correlation (>0.8), so that if the levels of one of them are decreased, the other metabolite also had its levels decreased.

**Figure 4:**
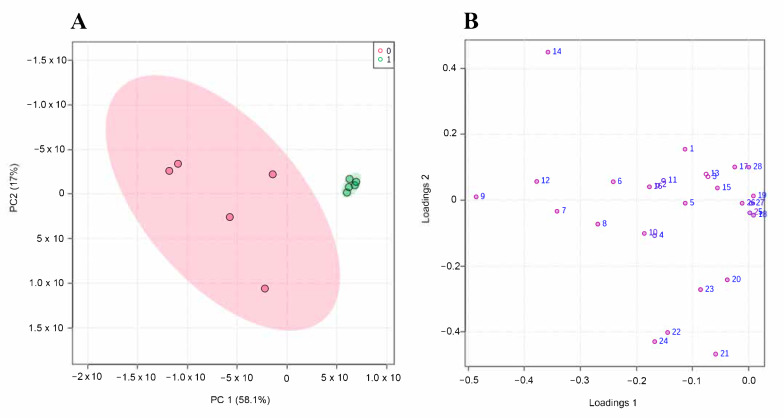
PCA results from the untargeted metabolomics experiment. (**A**) 2D scores plot of the first principal component (PC1) versus the second principal component (PC2) for control (*n* = 5) (red area) and cells treated with 5 μg/mL of AgNPs for 72 h (*n* = 5) (green area). (**B**) Loadings plot of PC1 versus PC2 for the 28 quantified metabolites (the number of each metabolite correlates with the identified metabolites as shown in Supplemental Table S2).

In order to detect patterns and to easily visualize the metabolic alterations, data from the 28 quantified common metabolites were subjected to a principal component analysis (PCA), which allows us to reduce the dimensionality of the multivariate data to a few principal components (PCs), being graphically visualized with a minimal loss of information. For this purpose, the data matrix was decomposed into matrices of scores (coordinates of the samples) and loadings (metabolites), providing information on samples and variables, respectively. According to this PCA analysis, a clear separation of both groups was verified on a basis of the two first principal components (Figure 4A). Numerical data of the explained variance for each component was well represented by the scores plot, where it can be noticed the contributions of both PCs (PC1 and PC2). These two components, PC1 and PC2, represented the 75.1% of the explained variance, and both experimental groups were separated along the first PC (PC1), which entails the 58.1% of the explained variance. Regarding the contribution of the variables (metabolites) represented by the loadings graph (Figure 4B), it can be observed that phosphoric acid (9), pantothenic acid (14), myo-inositol phosphate (21), octadecanoic acid (22) and myo-inositol (24) were the metabolites with the higher contribution for the separation of the experimental groups.

Additionally, considering the average normalized areas for metabolites correctly identified, a Student’s *t* test (*p*-values < 0.05) was carried out to evaluate significant differences for each metabolite between both experimental groups. The statistical analysis allowed us to detect significant differences in 20 out of the 28 quantified metabolites between the control and AgNPs-exposed cells. The altered metabolites are listed in Table 1, along with the chromatographic retention time and the Rmatch obtained from the NIST library, which certifies the correct identification of each of metabolite. Additionally, the R_M_ value has been defined as the ratio of the mean area of each metabolite measured in cells exposed to AgNPs to the mean area of the same compound in the control cells.

**Figure 5:**
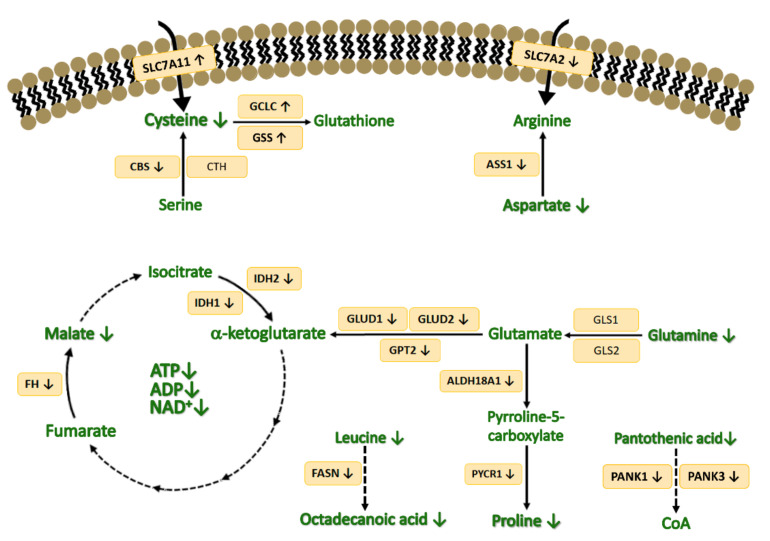
Illustration of the main metabolic reactions and pathways where the main impaired amino acids and TCA cycle intermediates found after AgNPs exposure take part.

**Figure 6:**
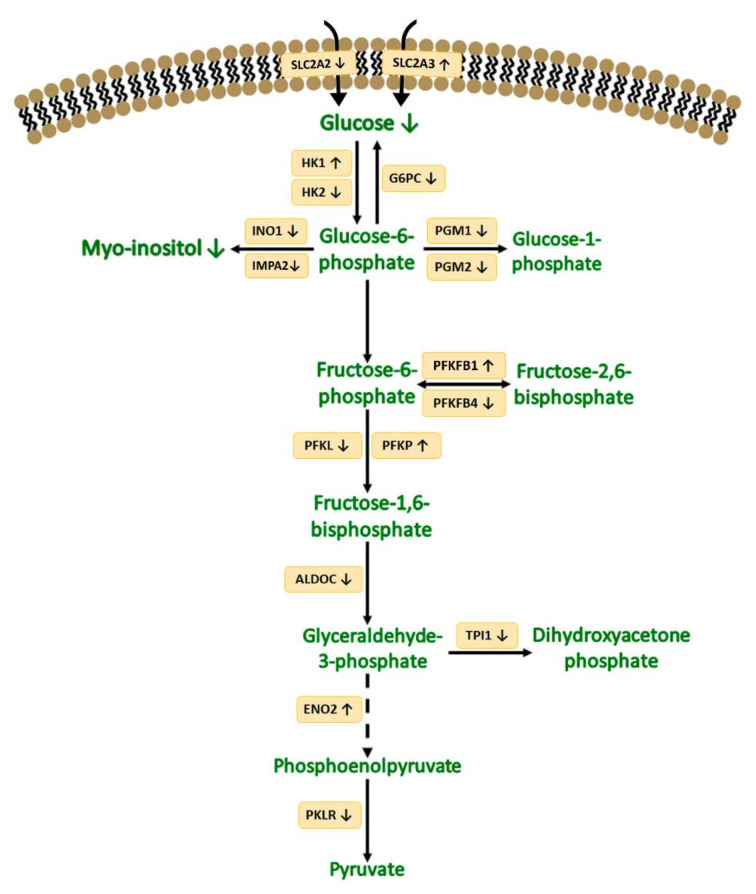
Scheme of the glycolysis pathway and the transcripts controlling its progression.

The untargeted metabolomics experiment allowed the identification and quantification of 28 metabolites of different nature, such as amino acids, pentose phosphate pathway (PPP) intermediates, carbon hydrates, tricarboxylic acid (TCA) cycle intermediates, fatty acids and other small organic and inorganic compounds. Out of the 28 metabolites, 20 of them were found deregulated in HepG2 cells after exposure to AgNPs. Different metabolic pathways were found impaired after exposure to 10 nm AgNPs including the glutathione and coenzyme A biosynthesis, the metabolism of glutamate, glutamine, leucine, aspartate, proline, myo-inositol and fatty acids, the TCA cycle and the glycolysis. Figure 5 and Figure 6 summarize the main altered metabolic pathways observed in HepG2 cells upon AgNPs exposure. Further discussion on the role of the different altered metabolites in relation to the observed toxicity of AgNPs in relation to previous studies is collected in the discussion section.

### 3.5. Transcriptome Analysis

To evaluate potential alterations in the mRNA expression levels of HepG2 cells exposed to AgNPs, a transcriptome microarray analysis was carried out. Among the more than 20,000 well-annotated human genes analyzed, 3229 were found significantly de-regulated in HepG2 cells after exposure to 10 nm AgNPs, considering a log2 fold change (FC) superior to 2.0 and inferior to 0.5 (with a *p*-value < 0.05). From those differentially expressed transcripts, 1616 were upregulated (ratios above 2.0), while 1613 were found downregulated (ratios below 0.5) (Table S3 in Supplementary Materials). Among these significantly impaired genes, some of them were found to be related to specific metabolic pathways, in which many of the altered metabolites previously found were also involved (Table 2).

The implications of the altered transcripts and their relationship to the metabolic pathways affected following exposure to AgNPs are discussed extensively in the discussion section.

## 4. Discussion

In the last decade, the use of AgNPs has drastically increased for a wide variety of applications; thus, the study of the potential toxicity associated with this type of nanoparticles is urgent and critical. Although the toxicity of AgNPs has been previously studied, most of these studies fail at giving a full picture of the molecular mechanisms by which the AgNPs exert their toxic effects. In a previous work, the toxicity of 10 nm AgNPs was evaluated using a SILAC-based quantitative proteomics approach. It was demonstrated that AgNPs were able to induce nucleolar stress, ribosome biogenesis halt, DNA damage and oxidative stress in HepG2 cells [12]. However, little is still known about what does occur at the genome and metabolome levels. Therefore, and with the aim of broadening the knowledge of the mechanisms of toxicity associated with exposure to AgNPs, this work has employed a combination of metabolomics (non-targeted and targeted) and transcriptomics approaches. Before delving into the mechanisms of toxicity, a cytotoxicity study was carried out in a HepG2 cell model using the MTT assay in order to select an appropriate concentration of AgNPs. The results showed that cytotoxicity decreases with increasing concentration, while on the opposite way, cell death increases with increasing concentration, as expected. Based on this, the concentration of 5 μg/mL and 72 h of exposure were selected for transcriptomics and metabolomics experiments.

In the present section, the main results obtained from the metabolomics study will be discussed together with the transcriptomics results, as well as with previous studies. For a better understanding, the discussion has been divided into several headings related to the different altered pathways.

**Glutathione biosynthesis activation.** One of the main observed and reported processes in previous in vitro and in vivo studies is the AgNPs-mediated oxidative stress [26]. Cysteine is one of the most important amino acids involved in the maintenance of the redox balance, since it takes part in the synthesis of glutathione (GSH) [27]. After AgNPs exposure, cysteine levels were found depleted (RM = 0.33); this has been linked to impaired cellular growth and cellular death due to the proliferation of oxidative stress and the limitation of iron-sulfur clusters, which are necessary for the electron transport chain (ETC) operation [28,29]. The low levels of cysteine might be explained by the downregulation of the CBS transcript (FC = 0.37) which is a key enzyme involved in the transsufuration pathway that, alongside CTH (FC = 0.70), also found to be downregulated, leads to the formation of cysteine. Consequently, the inhibition of CBS has also been reported to decrease GSH levels, leading to the increase in reactive oxygen species (ROS) levels [30]. In addition, due to the oxidative stress situation derived from the AgNPs action and since cysteine is also involved in the biosynthesis of GSH, the expression levels of the transcripts involved in this process were also assessed. GSH biosynthesis is catalyzed by two consecutive enzymes: glutamate cysteine ligase (GCL), which is the most important enzyme involved in GSH synthesis, and glutathione synthetase (GSS) [31]. After exposure to AgNPs, the catalytic subunit of GCL and GSS were found upregulated (FC = 3.49 and FC = 2.16, respectively); this was consistent with the activation and increased synthesis of GSH as a consequence of the oxidative environment induced by the AgNPs [32,33]. Moreover, it has been reported that GCL expression levels are also increased upon cysteine deficiency, which could also confirm the depletion of cysteine levels after AgNPs exposure [34]. Thus, the overexpression of these enzymes might contribute to the reduction in cysteine levels for generating GSH. In order to counterbalance this deficiency, cells can increase the import of cysteine, which was confirmed when it was observed that the expression levels of the main cysteine importer, SLC7A11, which imports cysteine in its oxidized form, was upregulated after AgNPs exposure (FC = 2.66) [35]. Therefore, AgNPs activate glutathione biosynthesis through the upregulation of the transsulfuration pathway as a consequence of ROS generation. Nevertheless, this activation might not be enough to overcome cysteine deficiency and could probably lead to ROS proliferation and, thus, to the induction of cellular death.

**Glutamine and glutamate metabolism impairment.** Apart from cysteine, it has also been reported that the deprivation of glutamine, as it has been observed in this study (RM = 0.69), contributes to maintain the oxidative stress situation. Under such conditions, glutamine is usually transformed to glutamate, which, in turn, is converted into α-ketoglutarate [35]. Therefore, under glutamine deficiency conditions, the generation and the intracellular levels of glutamate obtained from glutamine might also be reduced, despite the mRNA expression levels of the enzymes, which catalyze this reaction, GLS1 and GLS2, were not significantly impaired (FC = 0.98 and 0.92, respectively). In addition, after exposure to AgNPs, the production of α-ketoglutarate from glutamate might also be affected, since the transcripts of the two isoforms of the enzyme catalyzing this reaction, GLUD1 and GLUD2, were found significantly inhibited with a FC = 0.17. The inhibition of GLUD1 has also been related to increased ROS levels and, thus, oxidative stress, since GLUD1 inhibition leads to a decrease in fumarate levels, which finally leads to decreased glutathione peroxidases activity [36]. Additionally, the transcript encoding enzyme GPT2, which catalyzes the reversible transformation between α-ketoglutarate and glutamate, was also found to be downregulated (FC = 0.11); this is associated with the decrease in TCA cycle intermediates [37]. Thus, it can be concluded that AgNPs also impair some cellular defense mechanisms for facing the generated oxidative stress. Moreover, the biosynthesis of glutamine from glutamate was also compromised after AgNPs exposure, since the transcript encoding the enzyme involved in this transformation, GLUL, was also found downregulated (FC = 0.36). This downregulation has been previously linked to diminished cellular proliferation [38]. Hence, glutamine decrease after AgNPs exposure might lead to the decrease in glutamate levels, which, in turn, might affect TCA cycle anaplerosis by depleting α-ketoglutarate level, thus allowing ROS proliferation.

**TCA cycle and energetic metabolism disruption.** As the formation of α-ketoglutarate from glutamate is downregulated, it might also affect the correct operation of the TCA cycle. In this sense, the level of the TCA cycle intermediate malate was also found to be depleted (RM = 0.13) after exposure to AgNPs. This finding reinforces the idea of the impairment of this key metabolic pathway. Moreover, in addition to this metabolite, the mRNA expression levels of different enzymes involved in this cycle were also found deregulated. Among them, the expression level of the transcript encoding fumarate hydratase (FH) was inhibited after AgNPs action (FC = 0.41). This enzyme catalyzes the transformation of fumarate to malate, which could explain the observed low levels of malate. The inhibition of this enzyme has also been associated with the dysfunction of different respiratory chain complexes [39]. Besides this enzyme, the two isoforms of the transcript encoding the enzyme isocitrate dehydrogenase, IDH1 and IDH2, were also found downregulated after AgNPs exposure (FC = 0.23 and FC = 0.41, respectively). The inhibition of both isoforms of this enzyme can also be related to the induction of oxidative stress and, thus, to cellular death [40,41]. Hence, as the TCA cycle pathway might have a lower activity and since the performance of some respiratory chain complexes could be disrupted, it was expected that the oxidative phosphorylation pathway and, thus, the cellular energetic balance, might have been affected after AgNPs exposure. For this reason, a targeted metabolomics assay was performed to assess the cellular energetic status through the determination of the intracellular levels of ATP, ADP, NADH and NAD+ metabolites on HepG2 cells. After AgNPs exposure, ATP, ADP and NAD+ levels were significantly lower compared to control cells, thus proving the disruption of the oxidative phosphorylation pathway. Moreover, it has been reported that the increased ROS production associated with AgNPs action, along with the inhibition of ATP production, leads to the activation of apoptotic processes which finally induce cellular death [42]. In addition, the depletion of NAD+ contributes to the inhibition of the TCA cycle (as has been demonstrated) and to a decrease in oxidative phosphorylation activity, which could also make it difficult for the cellular capacity to regenerate ATP from ADP. Furthermore, the reduction in NAD+ contributes to the maintenance of the oxidative damage situation due to the disruption of the antioxidant defense system where it takes part, which finally induces cell death [43]. Hence, AgNPs induce cell death through the disruption of the cellular energetic balance by depleting the ATP intracellular levels, and by impairing different antioxidant mechanisms, which contribute to the generated oxidative stress situation. This depletion is driven by the decrease in different TCA cycle intermediates such as malate and α-ketoglutarate and the disruption of this pathway through the inhibition of the transcripts of enzymes involved in the course of the cycle.

**Effect on aspartate metabolism.** The hypoxic status induced by AgNPs alongside the electron chain transport (ETC) disruption has been reported to affect the aspartate biosynthesis and availability [44]. This fact was confirmed after AgNPs exposure, since the aspartate levels were found significantly depleted (RM = 0.24). This can lead to cellular death under TCA cycle inhibition and glutamine deficiency conditions, which have been observed. Moreover, aspartate is the precursor of arginine through a reaction catalyzed by the rate-limiting enzyme argininosuccinate synthase 1 (ASS1) [45], whose transcript was found downregulated after AgNPs exposure (FC = 0.12). ASS1 inhibition makes cells arginine-deficient and dependent on extracellular arginine, which is taken up by the arginine transporter encoded by the transcript SLC7A2, which in turn was also found inhibited after AgNPs exposure (FC = 0.28). Thus, the depletion of aspartate and the impossibility of cells to synthesize or import arginine might mean that AgNPs also produce a depletion in the intracellular levels of this amino acid, what has been reported to finally induce cellular death [46,47].

**Proline metabolism inhibition.** Another important metabolite which was found altered after exposure to AgNPs was proline (RM = 0.25). Its depletion may be due to the fact that glutamine, through glutamate, is the biosynthetic precursor of proline; since glutamate levels were probably depleted as well, it could affect proline levels. In addition, the transcript of the enzyme that catalyzes the transformation of glutamate into pyrroline-5-carboxylate (P5C), ALDH18A1, was strongly downregulated (FC = 0.12), reinforcing the idea that proline levels are reduced as a consequence of glutamine, and, thus, glutamate depletion [48]. This fact is also supported by the fact that the mRNA levels of PYCR1, an enzyme that catalyzes the final transformation of P5C into proline, was also found significantly inhibited (FC = 0.28). The inhibition of PYCR1 has been proved to reduce cell proliferation and also to increase the cellular apoptosis markers such as caspase 3 [49]. Moreover, proline can be hydroxylated to 4-hydroxyproline by the enzyme prolyl 4-hydroxylase to face oxidative stress and scavenge ROS. After AgNPs exposure, the transcript encoding the first isoform of this enzyme, P4HA1, was found downregulated (FC = 0.35), which has also been related to the reduction in cellular proliferation due to its inability to stabilize the antioxidant protein HIF-1 α [50,51]. Based on the above, it can be concluded that the decreased levels of proline after AgNPs exposure might also contribute to the maintenance of the oxidative stress situation [52]. Thus, AgNPs induced proline depletion through the impairment of glutamate metabolism and the downregulation of the biosynthetic pathway of these amino acids, which could finally lead to ROS increase and, therefore, cell death.

**Impairment of leucine and fatty acid metabolism.** In addition to the previously commented altered metabolites, the amino acid leucine was also found highly depleted in cells exposed to AgNPs (RM = 0.03). This depletion has been linked to the inhibition of cell proliferation and the induction of apoptosis, since the deficiency of this amino acid leads to the inhibition of the lipogenic gene FASN. As a matter of fact, the mRNA levels of this gene were also found significantly inhibited in cells exposed to AgNPs (FC = 0.27), which might, in turn, explain the observed reduced levels of the fatty acid octadecanoic acid (RM = 0.09) [53].

**Coenzyme A biosynthesis depletion.** Another important metabolite that was found depleted after AgNPs exposure was pantothenic acid (RM = 0.13), also known as vitamin B5. Pantothenic acid is the precursor of coenzyme A (CoA) and its reduced availability suppresses cellular proliferation, as CoA is involved in important metabolic processes such as the TCA cycle [54]. In addition, CoA biosynthesis is regulated by the pantothenate kinase (PANK) enzymes, of which PANK1 and PANK3 were found inhibited (FC = 0.11 and FC = 0.42, respectively), which has been reported to reduce CoA levels [55]. Thus, it can be concluded that CoA biosynthesis is also compromised due to the deficiency of its precursor and the downregulation of the key enzyme of this metabolic pathway.

**Effect on glycolysis pathway.** Although oxidative phosphorylation might be disrupted, cells can activate other metabolic pathways to obtain energy such as the glycolysis pathway, especially upon hypoxic situations (Figure 6). In this sense, the levels of glucose were significantly lower in AgNPs-exposed cells compared to control ones (RM = 0.31), which contributes to maintaining the oxidative stress situation, since under mitochondrial dysfunction situations, cells increase glucose metabolism to reduce ROS effect [56]. In order to confirm if the low levels of glucose were due to an enhanced glycolytic activity, the mRNA levels of different enzymes implicated in this pathway and some glucose transporters were evaluated. Among all the glucose transporters, two of them were found deregulated upon AgNPs exposure: SLC2A2, which was downregulated (FC = 0.23); and SLC2A3, which had the greatest affinity for glucose, and was found overexpressed (FC = 2.07). Although initially these results might appear contradictory [57], it has been reported that although SLC2A2 inhibition suppresses glucose uptake, it does not modify glucose homeostasis but might affect ATP production [58]. On the other hand, it has been reported that under low-glucose situations, cells upregulate SLC2A3 to increase glycolysis and cellular proliferation [57,59]. Based on these results suggesting that glucose metabolism might be increased after AgNPs exposure, the expression levels of different transcripts encoding enzymes implicated in glycolysis were subsequently assessed. Regarding the first glycolytic reaction, catalyzed by the enzyme hexokinase (HK), AgNPs induced the overexpression of the transcript encoding HK1 (FC = 11.86) and the inhibition of HK2 (FC = 0.36). It has been reported that HK1 overexpression could be a cellular mechanism to protect cells from oxidative stress situations through the activation of glycolysis and that HK2 downregulation inhibits the glycolytic flux [60]. In this sense, HK1 overexpression may overcome HK2 inhibition, thus promoting the glycolytic flux; this could explain the reduced glucose levels and the increased expression of the SLC2A3 transporter observed. Additionally, the mRNA levels of the isoforms PFKL and PFKP of the enzyme 6-phosphofructokinase were also oppositely impaired (FC = 0.40 and 33.45, respectively). The described effects of such de-regulations are also contradictory, since it has been reported that PFKL downregulation inhibits glycolysis, while PFKP overexpression promotes glycolysis [61]. Nevertheless, the activity of these enzymes is indirectly controlled by the PFKFB family of enzymes [62]. In this sense, PFKFB1 and PFKFB4 members were found differentially expressed with a FC = 4.62 and FC = 0.10, respectively. PFKFB1 overexpression apparently does not affect the course of the glycolysis pathway, while PFKFB4 inhibition has been reported to favor the glycolytic flux through an increase in fructose 2,6-bisphosphate levels [63]. On the contrary, the transcript encoding the enzyme ALDOC was found inhibited (FC = 0.13), which has been proved to downregulate glucose uptake, glycolysis and thus cellular proliferation [64]. This is coherent with the downregulation of the transcript encoding TPI1 protein (FC = 0.47) since it has also been related to a reduced glycolytic activity to face oxidative stress [65]. On the other hand, the mRNA levels of the ENO2 enzyme were upregulated (FC = 2.90), possibly contributing to the increase in glycolytic activity [66]. In contrast, the inhibition of PKLR as it has been observed in the present study (FC = 0.18) negatively regulates glycolysis [67].

Furthermore, the transcript encoding G6PC, which acts as a glycolysis regulator, was also found highly downregulated (FC = 0.02); this has been related to diminishing glucose production from glucose-6-phosphate and to the reduction in cellular proliferation [68]. Apart from this enzyme, PGM1 and PGM3, members of the family of proteins that catalyze the conversion between glucose-1-phosphate and glucose-6-phosphate, were also depleted after AgNPs exposure (FC = 0.14 and 0.45, respectively). The inhibition of PGM1 contributes to the increase in the flow of glucose into glycolysis, although in situations of glucose depletion it contributes to the suppression of cell proliferation [69]. In the case of PGM3, its downregulation has been proved to affect protein glycosylation, which finally contributes to the inducement of cell death [70,71]. Thus, taking all this information into account, the status of the glycolytic pathway after AgNPs exposure cannot be concluded, but it seems that the first part of this pathway might be activated driven by the overexpression of HK1 and the glucose transporter SLC2A3, as well as by the inhibition of PFKFB4. On the other hand, the rest of the pathway could be downregulated, since most of the transcripts are downregulated, which could explain the reduced ATP levels, since the upper activation of the pathway is not enough to counterbalance the disruption of the oxidative phosphorylation-generated ATP.

**Myo-inositol metabolism impairment.** Another important molecule that was found depleted upon AgNPs exposure was myo-inositol (RM = 0.10), which was also found depleted in its phosphorylated form with an RM = 0.07. Myo-inositol is considered as an essential nutrient as it enables cellular growth. Thus, its depletion might have important effects on cellular growth. Myo-inositol generation from glucose-6-phosphate is catalyzed by two consecutive enzymes: INO1 and IMPA, both of them were found inhibited after AgNPs exposure (INO1 FC = 0.43 and IMPA2 FC = 0.33), which is in accordance with the observed myoinositol levels. In addition, the inhibition of both enzymes has been proved to inhibit cellular proliferation and induce apoptosis, probably due to the impaired production of myo-inositol [72]. Moreover, myo-inositol is transformed to glucuronic acid by the enzyme MIOX, which, after a few transformations, enters into the glycolytic flux to support glycolysis. The mRNA levels of this enzyme were found upregulated (FC = 2.08) in exposed cells, which might be a cellular response to activate glycolysis to produce ATP. However, it has also been reported that MIOX overexpression is associated with contribute to the maintenance of oxidative stress situations and, thus, the inducement of cellular apoptosis [73]. Thus, AgNPs induced myo-inositol biosynthesis impairment and the decrease in myo-inositol levels, finally affecting cellular proliferation and inducing cell death.

## 5. Conclusions

The combination of targeted and untargeted metabolomics approaches, along with the microarray technique, allows the elucidation of the partial mechanisms by which 10 nm AgNPs exert their toxic effect on HepG2 cells. The analysis of the results confirmed the ability of the AgNPs to induce cellular oxidative stress; different pathways intended for counterbalancing this situation were found activated and the GSH biosynthesis pathway should be noted in particular. In addition, AgNPs depleted the levels of important molecules that constitute cellular defense systems against oxidative stress, which probably enhances the toxic effect exerted by this nanomaterial. In this sense, it was observed that AgNPs completely abolished the cellular energetic metabolism led by the decrease in intracellular ATP levels, which might be sustained by the diminished activity of the TCA cycle. The action of the AgNPs in the glycolysis pathway was also studied. Although the general status of such a pathway was not clear, it was observed that the upper steps could be activated in order to counterbalance ATP deficiency, although the mRNA levels of some enzymes catalyzing different steps of the pathway were downregulated. Thus, AgNPs exert their cytotoxic action by depleting the intracellular energetic metabolism and the levels of different important molecules involved in different cellular defense pathways, which could finally induce cellular death.

## Figures and Tables

**Table 1:** Altered metabolites found in HepG2 cells exposed to 5 μg/mL of AgNPs for 72 h by the GC-MS-based untargeted metabolomics approach (95% confidence level).

Metabolite Name	Retention Time (min)	NIST Rmatch	R_M_
Glutamine	19.890	874	0.69
Threitol	17.864	851	0.62
Xylofuranose	32.638	793	0.48
L-Cysteine	18.711	868	0.33
D-Glucose	24.964	870	0.31
L-Threonine	15.285	865	0.30
L-Proline	18.014	881	0.25
L-Aspartic acid	18.086	791	0.24
D-Fructose	24.294	767	0.23
β-D-Glucopyranose	26.375	890	0.15
D-Galactose	25.192	848	0.15
Pantothenic acid	26.473	887	0.13
Malic acid	17.466	728	0.13
Rythonic acid	18.541	767	0.12
Myo-inositol	27.874	830	0.10
Octadecanoic acid	30.225	839	0.09
Myo-inositol phosphate	32.426	827	0.07
L-Tyrosine	25.543	782	0.06
Phosphoric acid	22.634	868	0.05
L-Leucine	19.177	700	0.03

**Table 2:** Altered transcripts found in HepG2 cells exposed to 5 μg/mL of AgNPs for 72 h by the transcriptome approach.

Gene Name	Gene Code	FC
Phosphofructokinase, platelet	PFKP	33.45
Hexokinase 1	HK1	11.86
6-phosphofructo-2-kinase/fructose-2,6-biphosphatase 1	PFKFB1	4.62
Glutamate-cysteine ligase, catalytic subunit	GCLC	3.49
Enolase 2 (gamma, neuronal)	ENO2	2.90
Solute carrier family 7 (anionic amino acid transporter light chain, xc- system), member 11	SLC7A11	2.66
Glutathione synthetase	GSS	2.16
Myo-inositol oxygenase	MIOX	2.08
Solute carrier family 2 (facilitated glucose transporter), member 3	SLC2A3	2.07
Glutaminase	GLS1	0.98
Glutaminase 2 (liver, mitochondrial)	GLS2	0.92
Cystathionine gamma-lyase	CTH	0.70
Triosephosphate isomerase 1	TPI1	0.47
Phosphoglucomutase 3	PGM3	0.45
Inositol-3-phosphate synthase 1	INO1	0.43
Pantothenate kinase 3	PANK3	0.42
Fumarate hydratase	FH	0.41
Isocitrate dehydrogenase 2 (NADP+), mitochondrial	IDH2	0.41
Phosphofructokinase, liver	PFKL	0.40
Cystathionine-beta-synthase	CBS	0.37
Glutamate-ammonia ligase	GLUL	0.36
Hexokinase 2	HK2	0.36
Prolyl 4-hydroxylase, alpha polypeptide I	P4HA1	0.35
Inositol(myo)-1(or 4)-monophosphatase 2	IMPA2	0.33
Solute carrier family 7 (cationic amino acid transporter, y+ system), member 2	SLC7A2	0.28
Pyrroline-5-carboxylate reductase 1	PYCR1	0.28
Fatty acid synthase	FASN	0.27
Isocitrate dehydrogenase 1 (NADP+)	IDH1	0.23
Solute carrier family 2 (facilitated glucose transporter), member 2	SLC2A2	0.23
Pyruvate kinase, liver and RBC	PKLR	0.18
Glutamate dehydrogenase 1	GLUD1	0.17
Glutamate dehydrogenase 2	GLUD2	0.17
Phosphoglucomutase 1	PGM1	0.14
Aldolase C, fructose-bisphosphate	ALDOC	0.13
Argininosuccinate synthase 1	ASS1	0.12
Aldehyde dehydrogenase 18 family, member A1	ALDH18A1	0.12
Glutamic pyruvate transaminase (alanine aminotransferase) 2	GPT2	0.11
Pantothenate kinase 1	PANK1	0.11
6-phosphofructo-2-kinase/fructose-2,6-biphosphatase 4; microRNA 6823	PFKFB4	0.10
Glucose-6-phosphatase, catalytic subunit	G6PC	0.02

## Supplementary Materials

The following are available online at https://www.mdpi.com/article/10.3390/nano12101762/s1, Figure S1. EDX spectra obtained for the 10 nm AgNPs. Figure S2. MTT-based cell viability assay of HepG2 cells exposed to citrate. Table S1: Quantified metabolites in HepG2 cells after exposure to 5 µg/mL of AgNPs, Table S2: Pearson’s correlation matrix data for the 28 quantified metabolites. Table S3: Whole transcriptome analysis of HepG2 cells exposed to 10 nm AgNPs.
