# Supplementary material for: A Multi-Omics Approach to Evaluate the Toxicity Mechanisms Associated with Silver Nanoparticles Exposure
Source: Nanomaterials (Basel). 2022 May 22;12(10):1762. doi: 10.3390/nano12101762 (PMC9146515; doi:10.3390/nano12101762)
Supplement: Supplementary file 1 [file nanomaterials-12-01762-s001.zip › Supplemental Tables S1 and S2.pdf]

Table S1. Quantified metabolites in HepG2 cells after exposure to 5 μg/mL of AgNPs for 72 h with a match factor superior to 700.

Compound	Retention time (min)	NIST Rmatch
1- β-D-Glucopyranose	26.375	890
2- Malic acid	17.466	728
3- L-Tyrosine	25.543	782
4- D-Glucose	24.964	870
5- Xylofuranose	32.638	793
6- L-Leucine	19.177	700
7- L-Proline	18.014	881
8- L-Cysteine	18.711	868
9- Phosphoric acid	22.634	868
10- L-Threonine	15.285	865
11- Threitol	17.864	851
12- Erythrose	25.352	706
13- D-Fructose	24.294	767
14- Pantothenic acid	26.473	887
15- β-Aminoisobutyric acid	15.399	815
16- D-Galactose	25.192	848
17- Isocitric acid	23.595	815
18- L-Serine	14.743	803
19- Hexadecanoic acid	27.295	846
20- Glutamine	19.890	874
21- Myo-Inositol phosphate	32.426	827
22- Octadecanoic acid	30.225	839
23- L-Aspartic acid	18.086	791
24- Myo-Inositol	27.874	830
25- D-Ribofuranose	19.879	820
26- Palmitic acid	31.419	829
27- Rythonic acid	18.541	767

28- L-Phenylalanine	20.214	879
29- 4-Chlorophenylalanine (internal standard)	23.140	-

Table S2. Pearson's correlation matrix data for the 28 quantified metabolites.

	1	2	3	4	5	6	7	8	9	10	11	12	13	14	15	16	17	18	19	20	21	22	23	24	25	26	27	28
1	**1.0000**																											
2	**0.6045**	**1.0000**																										
3	**0.7217**	**0.8991**	**1.0000**																									
4	0.4772	0.2328	0.2271	**1.0000**																								
5	-0.2293	-0.0845	-0.2598	0.4698	**1.0000**																							
6	0.3376	0.5896	0.5680	0.5642	0.5395	**1.0000**																						
7	0.0628	0.4432	0.2546	0.5948	**0.8019**	**0.8623**	**1.0000**																					
8	0.3059	0.5859	0.3828	**0.6886**	**0.6773**	**0.8286**	**0.9361**	**1.0000**																				
9	0.3949	**0.6587**	0.5035	**0.6940**	**0.6459**	**0.8946**	**0.9339**	**0.9734**	**1.0000**																			
10	0.0457	0.4261	0.1552	0.5454	**0.7733**	**0.7548**	**0.9197**	**0.9245**	**0.8816**	**1.0000**																		
11	0.3772	**0.6338**	0.4349	0.3483	0.5804	**0.7870**	**0.7880**	**0.8421**	**0.8631**	**0.8264**	**1.0000**																	
12	0.3888	**0.6657**	0.4842	0.5187	**0.6236**	**0.8651**	**0.8851**	**0.9444**	**0.9548**	**0.8901**	**0.9666**	**1.0000**																
13	0.5350	**0.7361**	0.5886	0.0670	0.1340	0.5915	0.4553	0.5486	**0.6069**	0.4669	**0.8484**	**0.7455**	**1.0000**															
14	**0.6259**	**0.6470**	0.5680	0.2065	0.3161	0.5841	0.4808	0.5895	**0.6630**	0.4952	**0.8772**	**0.7954**	**0.8636**	**1.0000**														
15	-0.0214	0.1149	-0.1076	0.0747	**0.7102**	0.4904	**0.6015**	0.5574	0.5344	**0.6924**	**0.8187**	**0.7055**	**0.6269**	**0.6704**	**1.0000**													
16	0.5324	0.3271	0.1913	0.3560	0.4066	0.3789	0.3556	0.5469	0.5340	0.5468	**0.7504**	**0.6717**	**0.6198**	**0.7932**	**0.6990**	**1.0000**												
17	-0.1346	0.0974	-0.2016	-0.2655	0.2516	-0.0274	0.2128	0.1326	0.1367	0.2408	0.4035	0.2504	0.5033	0.3660	0.5928	0.2068	**1.0000**											
18	-0.2403	-0.0435	-0.3468	-0.1366	-0.1072	-0.3598	-0.1540	-0.1349	-0.1722	0.0527	-0.1068	-0.1860	0.0122	-0.2474	0.0130	-0.0102	0.4894	**1.0000**										
19	-0.0755	0.0113	-0.1832	-0.3290	-0.2961	-0.4487	-0.2216	-0.2176	-0.2525	-0.2436	-0.1450	-0.2208	0.1286	-0.0826	-0.0629	-0.2509	**0.7134**	0.4862	**1.0000**									
20	0.1982	0.2312	0.0701	0.4492	-0.1142	0.0305	0.0783	0.3058	0.1943	0.3248	0.0286	0.1363	-0.0640	-0.1716	-0.1868	0.2181	-0.3029	0.4140	-0.1049	**1.0000**								
21	-0.1849	0.1082	-0.0965	0.2763	0.1318	0.0178	0.1856	0.3298	0.1855	0.4110	0.0220	0.1381	-0.2123	-0.2574	-0.0987	0.1344	-0.3552	0.3187	-0.2928	**0.8330**	**1.0000**							
22	0.0678	0.1435	-0.0735	0.4686	0.3357	0.2823	0.3752	0.5498	0.4165	**0.6499**	0.3801	0.4434	0.1170	0.0924	0.3163	0.5462	-0.1748	0.2647	-0.3539	**0.8290**	**0.8286**	**1.0000**						
23	0.1748	0.5289	0.3293	0.2697	-0.0528	0.2400	0.2455	0.4612	0.3684	0.4687	0.2862	0.3686	0.2077	0.0770	-0.0264	0.3038	-0.2633	0.2860	-0.2319	**0.8568**	**0.8704**	**0.8023**	**1.0000**					
24	0.1997	0.4974	0.2610	0.2543	0.0246	0.2554	0.2670	0.4888	0.3937	0.5470	0.4052	0.4438	0.3122	0.1993	0.1660	0.4760	-0.1241	0.3481	-0.2119	**0.8466**	**0.8196**	**0.8719**	**0.9675**	**1.0000**				
25	-0.4482	-0.2318	-0.2240	0.1978	0.4370	-0.0174	0.2497	0.1290	0.0961	0.1616	-0.2037	-0.0472	-0.6068	-0.3089	-0.1978	-0.3013	-0.3262	-0.2732	-0.3023	-0.0723	0.3034	-0.0209	-0.0420	-0.1571	**1.0000**			
26	0.0194	0.0936	0.0564	**0.6254**	0.2924	0.2376	0.4441	0.3648	0.3846	0.2167	-0.0628	0.1259	-0.2225	-0.2173	-0.2775	-0.3558	-0.0142	0.0182	0.1922	0.0960	0.0442	-0.1052	-0.0739	-0.2030	0.4592	**1.0000**		
27	-0.1849	-0.2432	-0.2131	-0.2194	-0.0457	-0.4518	-0.3797	-0.3012	-0.3354	-0.1904	-0.2843	-0.2996	-0.4500	-0.1342	-0.1852	0.1395	-0.3769	-0.0137	-0.3916	0.1082	0.3918	0.1586	0.1808	0.1742	0.4765	-0.3884	**1.0000**	
28	**0.7399**	-0.0273	0.1148	0.4077	-0.1883	-0.1573	-0.2859	-0.0951	-0.0354	-0.2685	-0.0410	-0.0694	0.0824	0.2687	-0.1130	0.4246	-0.0664	-0.0303	0.0628	0.0869	-0.2934	-0.0483	-0.2028	-0.1366	-0.3441	0.0384	0.0179	**1.0000**
