# Supplementary material for: A Multi-Omics Approach to Evaluate the Toxicity Mechanisms Associated with Silver Nanoparticles Exposure
Source: Nanomaterials (Basel). 2022 May 22;12(10):1762. doi: 10.3390/nano12101762 (PMC9146515; doi:10.3390/nano12101762)
Supplement: Supplementary file 1 [file nanomaterials-12-01762-s001.zip › Table S3.pdf]

Table S3. Whole transcriptome analysis of HepG2 cells exposed to 10 nm AgNPs

Gene name	Gene code	FC
annexin A1	ANXA1	1364,89
SPANX family, member B1	SPANXB1	566,34
carboxypeptidase A4	CPA4	483,63
serpin peptidase inhibitor, clade B (ovalbumin), member 2	SERPINB2	440,35
chemokine (C-C motif) receptor 7	CCR7	333,90
lysosomal protein transmembrane 5	LAPTM5	295,82
cytidine deaminase	CDA	252,56
pregnancy specific beta-1-glycoprotein 9	PSG9	202,55
matrix metallopeptidase 3	MMP3	199,46
endothelial cell adhesion molecule	ESAM	171,97
nitric oxide synthase 3 (endothelial cell)	NOS3	171,19
matrix metallopeptidase 1	MMP1	165,48
progestagen-associated endometrial protein	PAEP	132,19
integrin alpha 3	ITGA3	129,85
cathepsin E	CTSE	118,54
interleukin 18	IL18	117,54
G protein-coupled receptor, class C, group 5, member A; microRNA 614	GPRC5A; MIR614	113,10
myeloid/lymphoid or mixed-lineage leukemia; translocated to, 11	MLLT11	111,44
multiple C2 domains, transmembrane 1	MCTP1	102,47
heat shock 22kDa protein 8	HSPB8	98,51
transmembrane 4 L six family member 19	TM4SF19	98,16
interleukin 1 receptor accessory protein-like 1	IL1RAPL1	97,28
aquaporin 3 (Gill blood group)	AQP3	82,62
cytochrome P450, family 2, subfamily S, polypeptide 1	CYP2S1	78,19
CD22 molecule; microRNA 5196	CD22; MIR5196	75,53
tensin 4	TNS4	74,91
tumor suppressor candidate 3	TUSC3	71,52
transmembrane protein 92	TMEM92	70,95
cytochrome P450, family 24, subfamily A, polypeptide 1	CYP24A1	70,27
myoferlin	MYOF	68,63
SP140 nuclear body protein	SP140	68,62
sel-1 suppressor of lin-12-like 3 (C. elegans)	SEL1L3	68,41
transgelin 2	TAGLN2	67,46
CD79a molecule, immunoglobulin-associated alpha	CD79A	62,78
apolipoprotein B mRNA editing enzyme, catalytic polypeptide-like 3B	APOBEC3B	62,47
heme oxygenase 1	HMOX1	60,53
glycerol-3-phosphate acyltransferase 3	GPAT3	58,92
GLI pathogenesis-related 1	GLIPR1	58,10

CAP-GLY domain containing linker protein family, member 4	CLIP4	57,82
AHNAK nucleoprotein 2	AHNAK2	56,78
S100 calcium binding protein A11	S100A11	54,86
RAB3B, member RAS oncogene family	RAB3B	53,30
crystallin alpha B	CRYAB	52,58
solute carrier family 16, member 6	SLC16A6	52,49
neurotensin receptor 1 (high affinity)	NTSR1	52,32
insulin like growth factor binding protein 6	IGFBP6	52,08
glutaminyl-peptide cyclotransferase	QPCT	50,08
tyrosine kinase with immunoglobulin-like and EGF-like domains 1	TIE1	49,65
interleukin 2 receptor, gamma	IL2RG	48,93
four and a half LIM domains 2	FHL2	48,71
solute carrier family 16, member 2 (thyroid hormone transporter)	SLC16A2	46,40
epithelial membrane protein 3	EMP3	45,63
TM4SF19-TCTEX1D2 readthrough (NMD candidate)	TM4SF19-TCTEX1D2	45,30
solute carrier family 12, member 8	SLC12A8	44,68
annexin A3	ANXA3	41,47
ankyrin repeat domain 1 (cardiac muscle)	ANKRD1	41,28
RNA binding motif, single stranded interacting protein 2	RBMS2	40,98
calpain 2, (m/II) large subunit	CAPN2	40,55
tripartite motif containing 29	TRIM29	39,87
synaptopodin	SYNPO	39,09
EH domain containing 2	EHD2	38,41
neuropilin 1	NRP1	36,87
paired box 6	PAX6	36,74
dual specificity phosphatase 5	DUSP5	36,44
coagulation factor II (thrombin) receptor-like 1	F2RL1	35,85
vanin 1	VNN1	34,63
phosphofructokinase, platelet	PFKP	34,45
golgin A8 family, member N	GOLGA8N	33,44
AXL receptor tyrosine kinase	AXL	33,11
BCL2-related protein A1	BCL2A1	32,58
stratifin	SFN	32,54
transmembrane protein 156	TMEM156	32,28
lymphocyte cytosolic protein 1 (L-plastin)	LCP1	32,11
S100 calcium binding protein A3	S100A3	31,89
leucine rich repeat neuronal 4	LRRN4	31,50
solute carrier family 16, member 4	SLC16A4	30,83
nucleic acid binding protein 1	NABP1	29,45
prostaglandin reductase 1	PTGR1	29,27
lymphatic vessel endothelial hyaluronan receptor 1	LYVE1	28,96

suprabasin	SBSN	28,81
solute carrier family 7 (amino acid transporter light chain, y+L system), member 7	SLC7A7	28,06
late cornified envelope 2C	LCE2C	27,74
FXYD domain containing ion transport regulator 5	FXYD5	27,70
keratin 12, type I	KRT12	27,68
chemokine (C-X-C motif) ligand 8	CXCL8	27,27
glia maturation factor, gamma	GMFG	26,94
FOS-like antigen 1	FOSL1	26,76
kallikrein related peptidase 6	KLK6	26,17
tissue factor pathway inhibitor 2	TFPI2	25,59
inositol 1,4,5-trisphosphate receptor, type 3	ITPR3	25,13
sushi-repeat containing protein, X-linked 2	SRPX2	25,05
katanin p80 subunit B-like 1	KATNBL1	24,68
SPANX family, member A2	SPANXA2	24,54
ATP binding cassette subfamily C member 3	ABCC3	24,21
solute carrier family 40 (iron-regulated transporter), member 1	SLC40A1	24,05
bile acid-CoA:amino acid N-acyltransferase	BAAT	23,92
laminin, beta 3; microRNA 4260	LAMB3; MIR4260	23,64
protein C receptor, endothelial	PROCR	23,58
UDP glucuronosyltransferase 1 family, polypeptide A1; UDP glucuronosyltransferase 1 family, polypeptide A6; UDP glucuronosyltransferase 1 family, polypeptide A4; UDP glucuronosyltransferase 1 family, polypeptide A10; UDP glucuronosyltransferase 1 family, polypeptide A8; UDP glucuronosyltransferase 1 family, polypeptide A7; UDP glucuronosyltransferase 1 family, polypeptide A5; UDP glucuronosyltransferase 1 family, polypeptide A3; UDP glucuronosyltransferase 1 family, polypeptide A9	UGT1A1; UGT1A6; UGT1A4; UGT1A10; UGT1A8; UGT1A7; UGT1A5; UGT1A3; UGT1A9	23,49
transmembrane protein 173	TMEM173	23,39
fatty acid binding protein 3, muscle and heart	FABP3	22,97
serine incorporator 2	SERINC2	22,96
Dab, mitogen-responsive phosphoprotein, homolog 2 (Drosophila)	DAB2	22,70
serglycin	SRGN	22,18
neuron navigator 3	NAV3	22,10
proline rich 20D; proline rich 20B	PRR20D; PRR20B	21,94
proline rich 20D; proline rich 20B	PRR20D; PRR20B	21,94
proline rich 20C; proline rich 20E	PRR20C; PRR20E	21,94
proline rich 20B; proline rich 20E; proline rich 20A; proline rich 20D	PRR20B; PRR20E; PRR20A; PRR20D	21,94
proline rich 20C; proline rich 20D	PRR20C; PRR20D	21,94
spexin hormone	SPX	21,64
karyopherin alpha 7 (importin alpha 8)	KPNA7	21,32

Ras association (RalGDS/AF-6) domain family member 2	RASSF2	21,24
major vault protein; PAXIP1 associated glutamate-rich protein 1	MVP; PAGR1	21,08
CD109 molecule	CD109	20,90
pyruvate dehyrogenase phosphatase catalytic subunit 1	PDP1	20,74
nitric oxide synthase trafficking	NOSTRIN	20,73
cAMP responsive element binding protein 5	CREB5	20,60
proteolipid protein 2 (colonic epithelium-enriched)	PLP2	20,53
tubulin, alpha 4a	TUBA4A	20,51
zinc finger protein 280A	ZNF280A	20,37
ELK3, ETS-domain protein (SRF accessory protein 2)	ELK3	20,26
golgin A8 family, member J; golgin A8 family, member I, pseudogene	GOLGA8J; GOLGA8IP	19,60
tetraspanin 1	TSPAN1	19,56
Kruppel-like factor 5 (intestinal)	KLF5	19,34
A kinase (PRKA) anchor protein 12	AKAP12	18,99
sushi domain containing 2	SUSD2	18,90
adhesion G protein-coupled receptor G1	ADGRG1	18,70
lipase, member H	LIPH	18,47
CD52 molecule	CD52	18,44
eukaryotic translation initiation factor 5A2	EIF5A2	18,37
calponin 1, basic, smooth muscle	CNN1	18,17
RAB7B, member RAS oncogene family	RAB7B	18,12
interleukin 6 receptor	IL6R	17,89
coronin, actin binding protein, 2B	CORO2B	17,86
adaptor-related protein complex 1 sigma 3 subunit	AP1S3	16,77
transforming growth factor beta 1 induced transcript 1	TGFB1I1	16,68
FERM domain containing 5	FRMD5	16,62
hexokinase domain containing 1	HKDC1	16,52
cell death-inducing DFFA-like effector c	CIDEC	16,48
tetratricopeptide repeat domain 9	TTC9	16,18
TIMP metallopeptidase inhibitor 1	TIMP1	16,11
tetraspanin 5	TSPAN5	16,10
palladin, cytoskeletal associated protein	PALLD	15,97
protein phosphatase 1, regulatory subunit 18	PPP1R18	15,95
golgin A8 family, member R	GOLGA8R	15,85
golgin A8 family, member T	GOLGA8T	15,70
engulfment and cell motility 1	ELMO1	15,37
E74-like factor 4 (ets domain transcription factor)	ELF4	14,97
solute carrier family 37 (glucose-6-phosphate transporter), member 2	SLC37A2	14,89
golgin A8 family, member J	GOLGA8J	14,84
serpin peptidase inhibitor, clade B (ovalbumin), member 8	SERPINB8	14,82

basic leucine zipper transcription factor, ATF-like	BATF	14,74
sulfide quinone reductase-like (yeast)	SQRDL	14,62
chromosome 19 open reading frame 33	C19orf33	14,60
SPANX family, member D	SPANXD	14,30
gamma-glutamyltransferase 5	GGT5	14,10
insulin like growth factor binding protein 7	IGFBP7	14,06
CEP295 N-terminal like; TIMP metallopeptidase inhibitor 2	CEP295NL; TIMP2	14,01
solute carrier family 48 (heme transporter), member 1	SLC48A1	13,86
SPANX family, member C; SPANX family, member D	SPANXC; SPANXD	13,84
RAS guanyl releasing protein 3 (calcium and DAG-regulated)	RASGRP3	13,82
myosin IF	MYO1F	13,81
leucine zipper-EF-hand containing transmembrane protein 2	LETM2	13,76
plasminogen activator, urokinase receptor	PLAUR	13,65
splA/ryanodine receptor domain and SOCS box containing 1	SPSB1	13,55
troponin C type 1 (slow)	TNNC1	13,45
arrestin domain containing 3	ARRDC3	13,19
retinol binding protein 1, cellular	RBP1	13,04
SH3 domain containing ring finger 1	SH3RF1	12,86
aldehyde oxidase 1	AOX1	12,67
SH3 domain binding glutamate-rich protein like 3	SH3BGRL3	12,65
zinc finger protein 185 (LIM domain)	ZNF185	12,41
suppressor of cytokine signaling 2	SOCS2	12,34
CD9 molecule	CD9	12,26
solute carrier family 22, member 18	SLC22A18	12,25
golgin A8 family, member O	GOLGA8O	12,24
cystatin SN	CST1	12,06
vestigial-like family member 3	VGLL3	11,92
hexokinase 1	HK1	11,86
sphingomyelin synthase 2	SGMS2	11,76
leukocyte-associated immunoglobulin-like receptor 2	LAIR2	11,75
testis expressed 37	TEX37	11,70
uridine phosphorylase 1	UPP1	11,63
ras-related C3 botulinum toxin substrate 2 (rho family, small GTP binding protein Rac2)	RAC2	11,56
ret finger protein-like 4A	RFPL4A	11,55
mucin 13, cell surface associated	MUC13	11,15
cellular retinoic acid binding protein 2	CRABP2	11,01
calpain 3	CAPN3	11,00
protein phosphatase 1, regulatory subunit 15A	PPP1R15A	10,99
keratin 23, type I	KRT23	10,97
F-box and leucine-rich repeat protein 2	FBXL2	10,80

colony stimulating factor 1 (macrophage)	CSF1	10,62
syntaxin 3	STX3	10,60
cardiotrophin-like cytokine factor 1	CLCF1	10,50
jun proto-oncogene	JUN	10,48
SH3 domain and tetratricopeptide repeats 2	SH3TC2	10,42
insulin like growth factor binding protein 4	IGFBP4	10,41
chemokine (C-C motif) receptor-like 2	CCRL2	10,36
cystatin S	CST4	10,29
neutrophil cytosolic factor 2	NCF2	10,26
CD68 molecule	CD68	10,23
toll-like receptor 4	TLR4	10,19
regulator of G-protein signaling 10	RGS10	10,17
lectin, galactoside-binding, soluble, 1	LGALS1	10,10
S100 calcium binding protein A16	S100A16	10,10
syndecan 4	SDC4	10,10
interferon, gamma-inducible protein 16	IFI16	10,07
peripheral myelin protein 22	PMP22	10,00
glucosaminyl (N-acetyl) transferase 3, mucin type	GCNT3	9,90
sequestosome 1	SQSTM1	9,90
2-5-oligoadenylate synthetase 1	OAS1	9,87
related RAS viral (r-ras) oncogene homolog	RRAS	9,77
chromosome 12 open reading frame 75	C12orf75	9,69
interleukin 4 receptor	IL4R	9,68
golgin A8 family, member M	GOLGA8M	9,67
toll-like receptor 6	TLR6	9,67
integrin alpha 5	ITGA5	9,67
ankyrin repeat and SOCS box containing 4	ASB4	9,62
matrix metallopeptidase 14 (membrane-inserted)	MMP14	9,55
P2RX5-TAX1BP3 readthrough (NMD candidate)	P2RX5-TAX1BP3	9,52
moesin	MSN	9,50
sperm-tail PG-rich repeat containing 1	STPG1	9,48
myeloma overexpressed	MYEOV	9,43
S100 calcium binding protein A6	S100A6	9,42
leupaxin	LPXN	9,23
solute carrier family 5 (sodium/inositol cotransporter), member 11	SLC5A11	9,22
sperm protein associated with the nucleus, X-linked, family member A1	SPANXA1	9,21
abhydrolase domain containing 4	ABHD4	9,14
histidine-rich glycoprotein	HRG	9,12
spermidine/spermine N1-acetyltransferase 1	SAT1	9,11
adenosine monophosphate deaminase 3	AMPD3	9,11
SLIT-ROBO Rho GTPase activating protein 1	SRGAP1	8,91
diacylglycerol kinase alpha	DGKA	8,91
claudin 4	CLDN4	8,90

septin 1	sept-01	8,87
sprouty-related, EVH1 domain containing 3	SPRED3	8,87
amphiregulin	AREG	8,72
chromosome 14 open reading frame 105	C14orf105	8,69
iduronate 2-sulfatase	IDS	8,68
ras homolog family member C	RHOC	8,67
lectin, galactoside-binding, soluble, 3	LGALS3	8,66
RAB27B, member RAS oncogene family	RAB27B	8,64
schlafen family member 5	SLFN5	8,64
ankyrin repeat and SOCS box containing 2	ASB2	8,58
serpin peptidase inhibitor, clade E (nexin, plasminogen activator inhibitor type 1), member 2	SERPINE2	8,56
leukemia inhibitory factor	LIF	8,53
potassium channel, voltage gated KQT-like subfamily Q, member 3	KCNQ3	8,51
sulfiredoxin 1	SRXN1	8,50
interleukin 11	IL11	8,41
SH3 domain containing ring finger 2	SH3RF2	8,37
purinergic receptor P2X, ligand gated ion channel, 5	P2RX5	8,35
potassium channel, voltage gated subfamily E regulatory beta subunit 3	KCNE3	8,35
SERTA domain containing 1	SERTAD1	8,34
pleckstrin homology domain containing, family M (with RUN domain) member 1; microRNA 4315-1	PLEKHM1; MIR4315-1	8,25
chromosome 6 open reading frame 132	C6orf132	8,25
cytochrome P450, family 3, subfamily A, polypeptide 5	CYP3A5	8,23
tubulin, beta 6 class V	TUBB6	8,22
claudin 6	CLDN6	8,21
killer cell lectin-like receptor subfamily C, member 2	KLRC2	8,17
matrix metallopeptidase 19	MMP19	8,17
solute carrier family 16 (monocarboxylate transporter), member 5	SLC16A5	7,97
septin 4	sept-04	7,90
Ras-related associated with diabetes	RRAD	7,87
progestin and adipoQ receptor family member III	PAQR3	7,85
copine VIII	CPNE8	7,84
synaptophysin-like 2	SYPL2	7,83
metallothionein 1H	MT1H	7,81
milk fat globule-EGF factor 8 protein	MFGE8	7,79
ubiquitin associated and SH3 domain containing B	UBASH3B	7,76
mitotic spindle positioning	MISP	7,75
synaptotagmin XI	SYT11	7,74
peroxisome proliferator-activated receptor delta	PPARD	7,68
transglutaminase 2	TGM2	7,66
chromosome 16 open reading frame 62	C16orf62	7,66

ADAM metallopeptidase with thrombospondin type 1 motif 4	ADAMTS4	7,64
PR domain containing 1, with ZNF domain	PRDM1	7,60
translocase of outer mitochondrial membrane 34	TOMM34	7,58
ryanodine receptor 3	RYR3	7,50
glutamine-fructose-6-phosphate transaminase 2	GFPT2	7,46
transmembrane channel like 5	TMC5	7,46
four and a half LIM domains 3	FHL3	7,39
protein phosphatase 1, regulatory (inhibitor) subunit 1C	PPP1R1C	7,38
growth factor receptor bound protein 10	GRB10	7,36
golgin A8 family, member N; golgin A8 family, member H	GOLGA8N; GOLGA8H	7,35
zinc finger protein 165	ZNF165	7,34
sirtuin 7	SIRT7	7,32
solute carrier family 44, member 4	SLC44A4	7,32
endogenous retrovirus group 48, member 1	ERVH48-1	7,30
solute carrier family 7 (amino acid transporter light chain, L system), member 8	SLC7A8	7,30
phosphatidylinositol transfer protein, cytoplasmic 1	PITPNC1	7,29
quiescin Q6 sulfhydryl oxidase 1	QSOX1	7,28
olfactomedin like 3	OLFML3	7,27
metallothionein 1M	MT1M	7,27
motile sperm domain containing 1	MOSPD1	7,27
transgelin	TAGLN	7,27
pregnancy specific beta-1-glycoprotein 1	PSG1	7,26
platelet-derived growth factor alpha polypeptide	PDGFA	7,25
ZFP36 ring finger protein	ZFP36	7,22
acyl-CoA synthetase long-chain family member 5	ACSL5	7,22
myotubularin related protein 11	MTMR11	7,19
dual specificity phosphatase 1	DUSP1	7,18
golgi transport 1A	GOLT1A	7,17
chromosome 16 open reading frame 45	C16orf45	7,17
ABRA C-terminal like	ABRACL	7,15
keratin 20, type I	KRT20	7,15
serine/threonine kinase 17b	STK17B	7,12
immediate early response 5	IER5	7,10
kynureninase	KYNU	7,10
nuclear factor, erythroid 2-like 3	NFE2L3	7,10
5-hydroxytryptamine (serotonin) receptor 1D, G protein-coupled	HTR1D	7,08
fatty acid desaturase 3	FADS3	7,07
pregnancy specific beta-1-glycoprotein 5	PSG5	7,06
phospholipase C, delta 3	PLCD3	7,01
internexin neuronal intermediate filament protein, alpha	INA	6,99

zinc finger protein 841	ZNF841	6,97
NDRG family member 4	NDRG4	6,95
arrestin domain containing 4	ARRDC4	6,94
keratin 19, type I	KRT19	6,92
solute carrier organic anion transporter family, member 1B3	SLCO1B3	6,91
killer cell lectin-like receptor subfamily C, member 3	KLRC3	6,91
ankyrin repeat domain 20 family, member A5, pseudogene; ras homolog family member T1 pseudogene 1	ANKRD20A5P; RHOT1P1	6,91
family with sequence similarity 65, member A	FAM65A	6,87
leukocyte-associated immunoglobulin-like receptor 1	LAIR1	6,86
Transcript Identified by AceView, Entrez Gene ID(s) 4846	NOS3	6,83
chromosome 9 open reading frame 72	C9orf72	6,82
polymerase (DNA-directed), delta 4, accessory subunit	POLD4	6,81
serum deprivation response	SDPR	6,77
proteolipid protein 1	PLP1	6,74
chondroitin sulfate N-acetylgalactosaminyltransferase 2	CSGALNACT2	6,72
oxidative stress induced growth inhibitor 1	OSGIN1	6,69
keratin associated protein 2-3	KRTAP2-3	6,69
homer scaffolding protein 2	HOMER2	6,66
extracellular matrix protein 1	ECM1	6,64
perforin 1 (pore forming protein)	PRF1	6,63
PQ loop repeat containing 2	PQLC2	6,58
sulfotransferase family 1C member 2	SULT1C2	6,56
formin 1	FMN1	6,55
FOS-like antigen 2	FOSL2	6,52
S100 calcium binding protein A5	S100A5	6,49
glycerophosphocholine phosphodiesterase 1	GPCPD1	6,47
pleckstrin homology-like domain, family A, member 2	PHLDA2	6,46
carbohydrate (chondroitin 4) sulfotransferase 11	CHST11	6,45
ankyrin repeat domain 42	ANKRD42	6,43
chromosome 1 open reading frame 100	C1orf100	6,42
B-cell CLL/lymphoma 9-like	BCL9L	6,40
Kruppel-like factor 9	KLF9	6,39
glutamate-cysteine ligase, modifier subunit	GCLM	6,35
nuclear receptor subfamily 1, group D, member 1	NR1D1	6,35
WD repeat domain 66	WDR66	6,35
glucosidase, beta, acid	GBA	6,35
cyclin-dependent kinase inhibitor 2A	CDKN2A	6,34
pyrimidinergic receptor P2Y, G-protein coupled, 4	P2RY4	6,34
early growth response 1	EGR1	6,33
carboxylesterase 1	CES1	6,32
formin 1	FMN1	6,28

PITH (C-terminal proteasome-interacting domain of thioredoxin-like) domain containing 1	PITHD1	6,24
chromosome 1 open reading frame 105	C1orf105	6,23
cathepsin L	CTSL	6,22
cyclin-dependent kinase inhibitor 1A (p21, Cip1)	CDKN1A	6,22
RAS protein activator like 2	RASAL2	6,20
transmembrane protein 40	TMEM40	6,16
polo-like kinase 2	PLK2	6,16
small integral membrane protein 9	SMIM9	6,11
microtubule associated protein 1B	MAP1B	6,11
sterile alpha motif domain containing 5	SAMD5	6,11
Fc fragment of IgE, high affinity I, receptor for; gamma polypeptide	FCER1G	6,11
CD55 molecule, decay accelerating factor for complement (Cromer blood group)	CD55	6,07
thioredoxin reductase 1	TXNRD1	6,04
UL16 binding protein 3	ULBP3	6,01
interferon, alpha-inducible protein 27	IFI27	6,01
aldo-keto reductase family 1, member B10 (aldose reductase)	AKR1B10	5,99
heparin-binding EGF-like growth factor	HBEGF	5,98
pregnancy specific beta-1-glycoprotein 6	PSG6	5,97
ubiquitin C-terminal hydrolase L1	UCHL1	5,94
platelet-activating factor receptor	PTAFR	5,94
aldo-keto reductase family 1, member B15	AKR1B15	5,93
poliovirus receptor-related 4	PVRL4	5,93
GABA(A) receptor-associated protein like 1	GABARAPL1	5,90
RAB31, member RAS oncogene family	RAB31	5,90
CDC42 small effector 1	CDC42SE1	5,90
metallothionein 1F	MT1F	5,89
v-ets avian erythroblastosis virus E26 oncogene homolog 1	ETS1	5,89
dermokine	DMKN	5,87
myosin light chain kinase	MYLK	5,87
purine nucleoside phosphorylase	PNP	5,86
diacylglycerol lipase, beta	DAGLB	5,84
dedicator of cytokinesis 11	DOCK11	5,82
kinesin family member 17	KIF17	5,79
dystrobrevin, alpha	DTNA	5,71
RIO kinase 3	RIOK3	5,69
chromosome 6 open reading frame 1	C6orf1	5,69
cyclin D3	CCND3	5,67
G protein-coupled receptor 137B	GPR137B	5,66
RNA binding protein with multiple splicing 2	RBPMS2	5,66

ATPase, aminophospholipid transporter, class I, type 8A, member 2	ATP8A2	5,64
adhesion G protein-coupled receptor E5	ADGRE5	5,63
CUB domain containing protein 1	CDCP1	5,62
ubiquitin specific peptidase 17-like family member 23 [Source:HGNC Symbol;Acc:HGNC:44451]	USP17L23	5,58
CD24 molecule	CD24	5,58
dystonin	DST	5,58
diacylglycerol kinase gamma	DGKG	5,57
basic helix-loop-helix family, member e41	BHLHE41	5,54
popeye domain containing 3	POPDC3	5,52
layilin	LAYN	5,52
nexilin (F actin binding protein)	NEXN	5,49
ADP-ribosylation factor like GTPase 8A	ARL8A	5,46
cadherin-related family member 2	CDHR2	5,46
clathrin, light chain B	CLTB	5,45
Tax1 (human T-cell leukemia virus type I) binding protein 3	TAX1BP3	5,45
anthrax toxin receptor 2	ANTXR2	5,40
transforming growth factor beta 1	TGFB1	5,39
jagged 1	JAG1	5,39
guanine nucleotide binding protein (G protein), alpha inhibiting activity polypeptide 1	GNAI1	5,39
zinc finger and BTB domain containing 20; microRNA 568	ZBTB20; MIR568	5,39
LCK proto-oncogene, Src family tyrosine kinase	LCK	5,37
protein kinase (cAMP-dependent, catalytic) inhibitor beta	PKIB	5,36
pecanex-like 2 (Drosophila)	PCNXL2	5,36
endothelial cell surface expressed chemotaxis and apoptosis regulator	ECSCR	5,35
dual specificity phosphatase 18	DUSP18	5,31
VGF nerve growth factor inducible	VGF	5,27
zinc finger, SWIM-type containing 6	ZSWIM6	5,20
matrix metallopeptidase 2	MMP2	5,20
dual specificity phosphatase 4	DUSP4	5,19
ubiquitin specific peptidase 17-like family member 27; ubiquitin specific peptidase 17-like family member 28	USP17L27; USP17L28	5,16
ubiquitin specific peptidase 17-like family member 28; ubiquitin specific peptidase 17-like family member 30	USP17L28; USP17L30	5,16
ubiquitin specific peptidase 17-like family member 29; ubiquitin specific peptidase 17-like family member 5	USP17L29; USP17L5	5,16
ubiquitin specific peptidase 17-like family member 25; ubiquitin specific peptidase 17-like family member 30	USP17L25; USP17L30	5,16
FERM domain containing 4A	FRMD4A	5,16

N-acetylglutamate synthase	NAGS	5,15
glucosamine-6-phosphate deaminase 1	GNPDA1	5,14
ret finger protein-like 4A-like 1	RFPL4AL1	5,14
ubiquitin specific peptidase 17-like family member 15	USP17L15	5,14
4-hydroxyphenylpyruvate dioxygenase	HPD	5,13
LIM and senescent cell antigen-like domains 2	LIMS2	5,12
chemokine (C-C motif) ligand 22	CCL22	5,11
ADAM metallopeptidase domain 19	ADAM19	5,06
rhomboid 5 homolog 2 (Drosophila)	RHBDF2	5,06
microtubule associated monooxygenase, calponin and LIM domain containing 1	MICAL1	5,04
STAM binding protein-like 1	STAMBPL1	5,04
CTTNBP2 N-terminal like	CTTNBP2NL	5,00
NUAK family, SNF1-like kinase, 2	NUAK2	5,00
distal-less homeobox 5	DLX5	4,97
intercellular adhesion molecule 2	ICAM2	4,95
neuregulin 1	NRG1	4,95
transient receptor potential cation channel, subfamily V, member 2	TRPV2	4,95
glucocorticoid induced 1	GLCCI1	4,93
chromosome 3 open reading frame 52	C3orf52	4,91
testis expressed 19	TEX19	4,91
actin binding LIM protein family, member 3	ABLIM3	4,89
calcium binding tyrosine-(Y)-phosphorylation regulated	CABYR	4,89
mucolipin 1	MCOLN1	4,88
coactosin-like F-actin binding protein 1	COTL1	4,87
transmembrane protein 59-like	TMEM59L	4,86
L1 cell adhesion molecule	L1CAM	4,83
sulfotransferase family 1C member 4	SULT1C4	4,82
hematopoietic cell signal transducer	HCST	4,82
STEAP family member 2, metalloreductase	STEAP2	4,82
tubulin, beta 3 class III; melanocortin 1 receptor (alpha melanocyte stimulating hormone receptor)	TUBB3; MC1R	4,80
granzyme B	GZMB	4,80
OTU deubiquitinase, ubiquitin aldehyde binding 2	OTUB2	4,78
Ras and Rab interactor 2	RIN2	4,77
glutamate receptor, ionotropic, N-methyl D-aspartate 2D	GRIN2D	4,74
adrenomedullin	ADM	4,73
transcobalamin II	TCN2	4,71
LDL receptor related protein 10	LRP10	4,66
regulator of cell cycle	RGCC	4,65
WD repeat domain 45; PRA1 domain family, member 2	WDR45; PRAF2	4,62
6-phosphofructo-2-kinase/fructose-2,6-biphosphatase 1	PFKFB1	4,62
transgelin 3	TAGLN3	4,62

HSPB (heat shock 27kDa) associated protein 1	HSPBAP1	4,61
paroxysmal nonkinesigenic dyskinesia; microRNA 6810	PNKD; MIR6810	4,60
calcium binding and coiled-coil domain 1	CALCOCO1	4,60
ubiquitin specific peptidase 17-like family member 28; ubiquitin specific peptidase 17-like family member 5; ubiquitin specific peptidase 17-like family member 24	USP17L28; USP17L5; USP17L24	4,58
S100 calcium binding protein A2	S100A2	4,57
F-box protein 32	FBXO32	4,57
TBC/LysM-associated domain containing 1	TLDC1	4,55
retinoic acid induced 14	RAI14	4,55
anterior gradient 2, protein disulphide isomerase family member	AGR2	4,54
polypeptide N-acetylgalactosaminyltransferase 10	GALNT10	4,53
carboxylesterase 1 pseudogene 1; carboxylesterase 1	CES1P1; CES1	4,52
GULP, engulfment adaptor PTB domain containing 1	GULP1	4,50
progestin and adipoQ receptor family member V	PAQR5	4,50
p21 protein (Cdc42/Rac)-activated kinase 1	PAK1	4,49
paxillin	PXN	4,48
angiotensin II receptor-associated protein	AGTRAP	4,47
zinc finger protein 385A	ZNF385A	4,47
trefoil factor 1	TFF1	4,47
late cornified envelope 1D	LCE1D	4,45
cancer/testis antigen family 45, member A6; cancer/testis antigen family 45, member A7	CT45A6; CT45A7	4,44
interferon-related developmental regulator 1	IFRD1	4,43
dopachrome tautomerase	DCT	4,43
hes-related family bHLH transcription factor with YRPW motif 1	HEY1	4,42
DNA (cytosine-5-)-methyltransferase 3 beta	DNMT3B	4,42
family with sequence similarity 107, member B	FAM107B	4,42
WW and C2 domain containing 1	WWC1	4,41
centrosomal protein 85kDa-like	CEP85L	4,40
poliovirus receptor-related 1 (herpesvirus entry mediator C)	PVRL1	4,40
mitochondrial fission regulator 2	MTFR2	4,39
lysophosphatidic acid receptor 5	LPAR5	4,38
dehydrogenase/reductase (SDR family) member 9	DHRS9	4,38
DEAD (Asp-Glu-Ala-Asp) box polypeptide 60-like	DDX60L	4,37
RNA binding motif protein 24	RBM24	4,36
lymphocyte antigen 96	LY96	4,36
GRB2-related adaptor protein	GRAP	4,36
formin 1	FMN1	4,36
six transmembrane epithelial antigen of the prostate 1	STEAP1	4,35
potassium channel, voltage gated eag related subfamily H, member 2	KCNH2	4,35

transmembrane channel like 7	TMC7	4,34
leucine rich adaptor protein 1-like	LURAP1L	4,34
neuronal calcium sensor 1	NCS1	4,34
ATPase, H+ transporting, lysosomal 31kDa, V1 subunit E1	ATP6V1E1	4,34
UDP-Gal:betaGlcNAc beta 1,3-galactosyltransferase 5	B3GALT5	4,33
DNA damage-induced apoptosis suppressor	DDIAS	4,33
gamma-aminobutyric acid (GABA) A receptor, epsilon; microRNA 224; microRNA 452	GABRE; MIR224; MIR452	4,33
metal-regulatory transcription factor 1	MTF1	4,32
ral guanine nucleotide dissociation stimulator-like 1	RGL1	4,32
mitogen-activated protein kinase kinase kinase 12	MAP3K12	4,32
STEAP family member 1B	STEAP1B	4,31
ectopic P-granules autophagy protein 5 homolog (C. elegans)	EPG5	4,31
transmembrane protein 144	TMEM144	4,29
zymogen granule protein 16B	ZG16B	4,28
nestin	NES	4,28
APEX nuclease (apurinic/apyrimidinic endonuclease) 2	APEX2	4,27
acyl-CoA synthetase long-chain family member 1	ACSL1	4,27
collagen triple helix repeat containing 1	CTHRC1	4,25
synovial sarcoma, X breakpoint 1	SSX1	4,25
cancer/testis antigen family 45, member A7; cancer/testis antigen family 45, member A6	CT45A7; CT45A6	4,24
ZFP36 ring finger protein-like 1	ZFP36L1	4,24
interleukin 20 receptor beta	IL20RB	4,21
polymerase (DNA directed) kappa	POLK	4,21
endothelin 1	EDN1	4,21
anaphase promoting complex subunit 15	ANAPC15	4,20
spindle and kinetochore associated complex subunit 1	SKA1	4,20
family with sequence similarity 50, member A	FAM50A	4,18
chromosome 18 open reading frame 54	C18orf54	4,17
active BCR-related	ABR	4,14
optineurin	OPTN	4,13
activin A receptor type I	ACVR1	4,13
epithelial membrane protein 1	EMP1	4,12
erythrocyte membrane protein band 4.1-like 1	EPB41L1	4,12
polymerase (RNA) III (DNA directed) polypeptide C (62kD)	POLR3C	4,12
ring finger protein 125, E3 ubiquitin protein ligase	RNF125	4,12
chemokine (C-C motif) ligand 26	CCL26	4,12
guanylate binding protein 3	GBP3	4,11
myosin VA	MYO5A	4,11
ORMDL sphingolipid biosynthesis regulator 2	ORMDL2	4,11
cyclin N-terminal domain containing 2	CNTD2	4,11

RNA binding motif protein 47	RBM47	4,10
basic leucine zipper nuclear factor 1	BLZF1	4,09
heat shock 70kDa protein 1A; heat shock 70kDa protein 1B	HSPA1A; HSPA1B	4,09
Memczak2013 ANTISENSE, coding, INTERNAL, UTR3 best transcript NM_002727	SRGN	4,08
interleukin 1 receptor, type II	IL1R2	4,07
inositol 1,4,5-trisphosphate receptor interacting protein	ITPRIP	4,07
ceroid-lipofuscinosis, neuronal 8	CLN8	4,07
cysteine-rich, angiogenic inducer, 61	CYR61	4,07
nuclear receptor subfamily 1, group I, member 2	NR1I2	4,07
IGF like family member 2	IGFL2	4,06
phorbol-12-myristate-13-acetate-induced protein 1	PMAIP1	4,06
biliverdin reductase B	BLVRB	4,04
pleckstrin homology domain containing, family G (with RhoGef domain) member 4	PLEKHG4	4,03
pregnancy specific beta-1-glycoprotein 8	PSG8	4,02
Rho GTPase activating protein 42	ARHGAP42	4,01
CD163 molecule-like 1	CD163L1	4,01
S100 calcium binding protein A10	S100A10	4,00
lymphoid enhancer-binding factor 1	LEF1	4,00
dynamin binding protein	DNMBP	4,00
CAP, adenylate cyclase-associated protein, 2 (yeast)	CAP2	3,99
solute carrier family 38, member 7	SLC38A7	3,99
caspase 8, apoptosis-related cysteine peptidase	CASP8	3,99
AP2 associated kinase 1	AAK1	3,98
two pore segment channel 1	TPCN1	3,98
latent transforming growth factor beta binding protein 1	LTBP1	3,96
signal transducing adaptor molecule (SH3 domain and ITAM motif) 1	STAM	3,96
solute carrier family 38, member 6	SLC38A6	3,95
tripartite motif containing 54	TRIM54	3,95
PALM2-AKAP2 readthrough	PALM2-AKAP2	3,95
BAI1-associated protein 2-like 1	BAIAP2L1	3,94
phosphatidylinositol-5-phosphate 4-kinase, type II, alpha	PIP4K2A	3,94
serine dehydratase-like	SDSL	3,93
tumor necrosis factor receptor superfamily, member 12A	TNFRSF12A	3,92
DnaJ (Hsp40) homolog, subfamily B, member 4	DNAJB4	3,92
heat shock 70kDa protein 1B; heat shock 70kDa protein 1A	HSPA1B; HSPA1A	3,92
SEC14-like lipid binding 2	SEC14L2	3,92
mitogen-activated protein kinase 13	MAPK13	3,91

stannin	SNN	3,90
snail family zinc finger 1	SNAI1	3,87
HECT, C2 and WW domain containing E3 ubiquitin protein ligase 2	HECW2	3,86
arachidonate 5-lipoxygenase-activating protein	ALOX5AP	3,86
melanoregulin	MREG	3,86
HUS1 checkpoint clamp component	HUS1	3,85
hepatocyte growth factor-regulated tyrosine kinase substrate	HGS	3,85
jun B proto-oncogene	JUNB	3,84
Transcript Identified by AceView, Entrez Gene ID(s) 3684	ITGAM	3,84
thioredoxin interacting protein	TXNIP	3,84
dedicator of cytokinesis 4	DOCK4	3,83
microtubule associated protein 1A	MAP1A	3,82
Memczak2013 ANTISENSE, CDS, coding, INTERNAL best transcript NM_003123	SPN	3,82
keratin associated protein 2-1	KRTAP2-1	3,82
thymosin beta 4, X-linked	TMSB4X	3,82
solute carrier family 8 (sodium/lithium/calcium exchanger), member B1	SLC8B1	3,81
keratin associated protein 2-2	KRTAP2-2	3,80
allantoicase	ALLC	3,80
SP110 nuclear body protein	SP110	3,79
Sec61 translocon alpha 2 subunit	SEC61A2	3,79
DPY30 domain containing 2	DYDC2	3,78
coronin 6	CORO6	3,78
laccase (multicopper oxidoreductase) domain containing 1	LACC1	3,77
leucine rich repeat containing 8 family, member A	LRRC8A	3,76
filamin A, alpha	FLNA	3,76
drebrin 1	DBN1	3,76
thymosin beta 10	TMSB10	3,76
calmodulin regulated spectrin-associated protein family, member 2	CAMSAP2	3,75
tumor necrosis factor (ligand) superfamily, member 15	TNFSF15	3,75
SET domain containing (lysine methyltransferase) 7	SETD7	3,75
zinc finger protein 860	ZNF860	3,75
solute carrier family 6 (neurotransmitter transporter), member 6	SLC6A6	3,74
cytoplasmic polyadenylation element binding protein 4	CPEB4	3,74
KIAA1217	KIAA1217	3,73
renin binding protein	RENBP	3,73
mab-21-like 3 (C. elegans)	MAB21L3	3,73
immediate early response 2	IER2	3,73

Kruppel-like factor 2	KLF2	3,72
interleukin 32	IL32	3,70
parvin, alpha	PARVA	3,70
ATPase, H+ transporting, lysosomal 34kDa, V1 subunit D	ATP6V1D	3,69
homolog of rat pragma of Rnd2; Tyrosine-protein kinase SgK223 [Source:UniProtKB/Swiss-Prot;Acc:Q86YV5]	SGK223	3,69
G protein-coupled receptor 3	GPR3	3,69
fyn-related Src family tyrosine kinase	FRK	3,69
coagulation factor II (thrombin) receptor	F2R	3,69
LON peptidase N-terminal domain and ring finger 3	LONRF3	3,68
thrombospondin 3	THBS3	3,67
oxidative stress induced growth inhibitor family member 2	OSGIN2	3,67
adaptor-related protein complex 1 sigma 2 subunit	AP1S2	3,67
RasGEF domain family member 1B	RASGEF1B	3,67
feline leukemia virus subgroup C cellular receptor family, member 2	FLVCR2	3,66
carbonic anhydrase XII	CA12	3,64
dynein, cytoplasmic 2, heavy chain 1	DYNC2H1	3,64
solute carrier family 25, member 27	SLC25A27	3,64
hippocalcin-like 1	HPCAL1	3,63
complement component 1, s subcomponent	C1S	3,63
RUN and SH3 domain containing 2	RUSC2	3,63
microtubule associated monooxygenase, calponin and LIM domain containing 2	MICAL2	3,63
metallothionein 1X	MT1X	3,63
spermine oxidase	SMOX	3,62
ring finger protein 217	RNF217	3,62
cholesteryl ester transfer protein, plasma	CETP	3,62
vacuolar protein sorting 11 homolog (S. cerevisiae)	VPS11	3,61
melanoma cell adhesion molecule; microRNA 6756	MCAM; MIR6756	3,61
cancer/testis antigen family 45, member A5	CT45A5	3,60
serine peptidase inhibitor, Kunitz type 1	SPINT1	3,60
collagen, type VI, alpha 1	COL6A1	3,60
POTE ankyrin domain family, member F	POTEF	3,59
NLR family, pyrin domain containing 10	NLRP10	3,59
IGF like family member 4	IGFL4	3,59
discoidin, CUB and LCCL domain containing 2	DCBLD2	3,58
EF-hand domain family member D2	EFHD2	3,57
dedicator of cytokinesis 9	DOCK9	3,56
cytohesin 3	CYTH3	3,56
regulator of G-protein signaling 20	RGS20	3,56
zinc finger protein 280C	ZNF280C	3,55
BEN domain containing 7	BEND7	3,55
centrosomal protein 128kDa	CEP128	3,54

activating transcription factor 3	ATF3	3,53
tripartite motif containing 38	TRIM38	3,53
zinc finger protein 804A	ZNF804A	3,52
syndecan binding protein	SDCBP	3,51
cytoglobin	CYGB	3,50
trophoblast glycoprotein	TPBG	3,50
late endosomal/lysosomal adaptor, MAPK and MTOR activator 1	LAMTOR1	3,50
transmembrane protein 87B	TMEM87B	3,50
long intergenic non-protein coding RNA 1588	LINC01588	3,49
ATPase, H+ transporting, lysosomal V0 subunit a4	ATP6V0A4	3,49
glutamate-cysteine ligase, catalytic subunit	GCLC	3,49
serine/threonine kinase 17a	STK17A	3,48
CDK5 regulatory subunit associated protein 2	CDK5RAP2	3,48
ferredoxin 1-like	FDX1L	3,47
sterile alpha motif and leucine zipper containing kinase AZK; Mitogen-activated protein kinase kinase kinase MLT [Source:UniProtKB/Swiss-Prot;Acc:Q9NYL2]; Salzman2013 ANNOTATED, CDS, coding, INTERNAL, OVCODE, OVERLAPTX, OVEXON best transcript NM_133646; Salzman2013 ANNOTATED, CDS, coding, OVCODE, OVERLAPTX, OVEXON, UTR3 best transcript NM_016653; Salzman2013 ANNOTATED, CDS, coding, OVCODE, OVERLAPTX, OVEXON, UTR3 best transcript NM_133646; Salzman2013 ANNOTATED, CDS, coding, INTERNAL, OVCODE, OVERLAPTX, OVEXON best transcript NM_016653; Transcript Identified by AceView, Entrez Gene ID(s) 51776	ZAK; pk	3,47
chromosome X open reading frame 56	CXorf56	3,47
integrin, alpha 2 (CD49B, alpha 2 subunit of VLA-2 receptor)	ITGA2	3,46
SP100 nuclear antigen	SP100	3,45
NUAK family, SNF1-like kinase, 1	NUAK1	3,45
major facilitator superfamily domain containing 11	MFSD11	3,44
pregnancy specific beta-1-glycoprotein 3	PSG3	3,44
centromere protein W	CENPW	3,43
FIG4 phosphoinositide 5-phosphatase	FIG4	3,43
RAB27A, member RAS oncogene family	RAB27A	3,43
pregnancy specific beta-1-glycoprotein 4	PSG4	3,43
protein phosphatase 1, regulatory (inhibitor) subunit 11	PPP1R11	3,43
alpha kinase 2	ALPK2	3,42
myocyte enhancer factor 2D	MEF2D	3,42
solute carrier family 20 (phosphate transporter), member 1	SLC20A1	3,42
t-complex 11, testis-specific-like 2	TCP11L2	3,41

caprin family member 2	CAPRIN2	3,40
versican	VCAN	3,40
PTPRF interacting protein, binding protein 1 (liprin beta 1)	PPFIBP1	3,40
Rho GTPase activating protein 5	ARHGAP5	3,40
Kruppel-like factor 6	KLF6	3,38
DBF4 zinc finger B	DBF4B	3,38
synovial sarcoma, X breakpoint 2; synovial sarcoma, X breakpoint 2B	SSX2; SSX2B	3,38
paraneoplastic Ma antigen 1	PNMA1	3,38
ring finger protein 14	RNF14	3,37
AT rich interactive domain 3B (BRIGHT-like)	ARID3B	3,37
matrix-remodelling associated 7	MXRA7	3,37
Kruppel-like factor 7 (ubiquitous)	KLF7	3,37
membrane protein, palmitoylated 1	MPP1	3,36
arachidonate lipoxygenase 3	ALOXE3	3,36
sorting nexin 24	SNX24	3,35
transmembrane protein 99	TMEM99	3,35
vacuolar protein sorting 37 homolog B (S. cerevisiae)	VPS37B	3,35
high mobility group AT-hook 1	HMGA1	3,35
cadherin 1, type 1	CDH1	3,35
vesicle associated membrane protein 5	VAMP5	3,34
family with sequence similarity 219, member A	FAM219A	3,34
tubulointerstitial nephritis antigen-like 1	TINAGL1	3,34
matrix-remodelling associated 8	MXRA8	3,33
solute carrier family 35, member F2	SLC35F2	3,33
lysophosphatidylcholine acyltransferase 2	LPCAT2	3,33
KIAA0196	KIAA0196	3,33
Fas cell surface death receptor	FAS	3,33
calpastatin	CAST	3,32
leucine rich repeat containing 8 family, member B	LRRC8B	3,31
ELOVL fatty acid elongase 1; microRNA 6734	ELOVL1; MIR6734	3,30
phosphoprotein enriched in astrocytes 15	PEA15	3,30
smoothelin	SMTN	3,30
FYVE, RhoGEF and PH domain containing 6	FGD6	3,30
receptor-interacting serine-threonine kinase 2	RIPK2	3,30
myosin light chain 9	MYL9	3,29
tripartite motif containing 21	TRIM21	3,28
VPS11, CORVET/HOPS core subunit [Source:HGNC Symbol;Acc:HGNC:14583]	VPS11	3,28
solute carrier family 25, member 51	SLC25A51	3,28
ataxin 7-like 1	ATXN7L1	3,28
Salzman2013 ALT_ACCEPTOR, ALT_DONOR, coding, INTERNAL, intronic best transcript NM_152740	HIBADH	3,26
flavin adenine dinucleotide synthetase 1	FLAD1	3,26

actin related protein 2/3 complex subunit 4	ARPC4	3,26
zinc finger protein 554	ZNF554	3,26
human immunodeficiency virus type I enhancer binding protein 2	HIVEP2	3,25
family with sequence similarity 155, member B	FAM155B	3,25
family with sequence similarity 49, member A	FAM49A	3,24
FYN proto-oncogene, Src family tyrosine kinase	FYN	3,23
proline-serine-threonine phosphatase interacting protein 2	PSTPIP2	3,23
tetraspanin 4	TSPAN4	3,23
RUN domain and cysteine-rich domain containing, Beclin 1-interacting protein; microRNA 922	RUBCN; MIR922	3,23
microtubule-associated protein 1 light chain 3 beta	MAP1LC3B	3,23
galactosidase, alpha	GLA	3,21
major facilitator superfamily domain containing 12	MFSD12	3,21
golgin A8 family, member F; golgin A8 family, member G	GOLGA8F; GOLGA8G	3,21
Rho/Rac guanine nucleotide exchange factor 18	ARHGEF18	3,20
family with sequence similarity 214, member B	FAM214B	3,20
ribonuclease P/MRP 30kDa subunit	RPP30	3,19
kinesin light chain 1	KLC1	3,19
SRY box 4	SOX4	3,18
discs, large homolog 4 (Drosophila)	DLG4	3,18
myosin binding protein H	MYBPH	3,18
TAF12 RNA polymerase II, TATA box binding protein (TBP)-associated factor, 20kDa	TAF12	3,18
TBC/LysM-associated domain containing 2	TLDC2	3,18
major facilitator superfamily domain containing 1	MFSD1	3,18
F-box protein 30	FBXO30	3,18
Myb/SANT-like DNA-binding domain containing 3	MSANTD3	3,17
POC5 centriolar protein	POC5	3,17
WD repeat domain 44	WDR44	3,17
transport and golgi organization 2 homolog	TANGO2	3,17
kinesin family member 3C	KIF3C	3,17
Sad1 and UNC84 domain containing 3	SUN3	3,17
gametocyte specific factor 1	GTSF1	3,16
Ras-related GTP binding C	RRAGC	3,16
notch 2	NOTCH2	3,16
formin like 1	FMNL1	3,16
complexin 2	CPLX2	3,15
zyxin	ZYX	3,15
GRB2-associated binding protein 2	GAB2	3,15
bone morphogenetic protein 1	BMP1	3,14
microtubule-associated protein, RP/EB family, member 3	MAPRE3	3,14

polycystic kidney disease 1-like 2 (gene/pseudogene)	PKD1L2	3,14
tetratricopeptide repeat domain 39A	TTC39A	3,14
solute carrier family 35, member F6	SLC35F6	3,13
KIAA0430; microRNA 6506	KIAA0430; MIR6506	3,13
ubiquitin C-terminal hydrolase L3	UCHL3	3,12
phospholipid scramblase 3; transmembrane protein 256; TMEM256-PLSCR3 readthrough (NMD candidate)	PLSCR3; TMEM256; TMEM256-PLSCR3	3,12
phospholipase B1	PLB1	3,12
NLR family, pyrin domain containing 10	NLRP10	3,12
zinc finger, AN1-type domain 2A	ZFAND2A	3,12
general transcription factor IIH subunit 1	GTF2H1	3,12
tribbles pseudokinase 1	TRIB1	3,10
platelet endothelial aggregation receptor 1	PEAR1	3,10
IQ motif containing GTPase activating protein 1	IQGAP1	3,10
zinc finger protein 83	ZNF83	3,10
GTP binding protein overexpressed in skeletal muscle	GEM	3,10
coiled-coil domain containing 109B	CCDC109B	3,09
tsukushi, small leucine rich proteoglycan	TSKU	3,09
ArfGAP with SH3 domain, ankyrin repeat and PH domain 1; ASAP1 intronic transcript 2	ASAP1; ASAP1-IT2	3,09
S100 calcium binding protein A14	S100A14	3,09
SMAD specific E3 ubiquitin protein ligase 2	SMURF2	3,08
Transcript Identified by AceView, Entrez Gene ID(s) 8878	SQSTM1	3,08
monoamine oxidase A	MAOA	3,08
solute carrier family 26 (anion exchanger), member 9	SLC26A9	3,08
BRISC and BRCA1 A complex member 1	BABAM1	3,08
cyclin E2	CCNE2	3,07
discs, large (Drosophila) homolog-associated protein 4	DLGAP4	3,06
syntaxin 1A (brain)	STX1A	3,06
ATP binding cassette subfamily C member 1	ABCC1	3,06
phospholipase A2, group IID	PLA2G2D	3,06
tumor necrosis factor, alpha-induced protein 8	TNFAIP8	3,05
VPS18 CORVET/HOPS core subunit	VPS18	3,05
ARPC4-TTLL3 readthrough	ARPC4-TTLL3	3,04
SPRY domain containing 3	SPRYD3	3,04
SLX4 interacting protein	SLX4IP	3,03
zinc finger, AN1-type domain 5	ZFAND5	3,03
golgi membrane protein 1	GOLM1	3,03
growth arrest and DNA-damage-inducible, beta	GADD45B	3,03
calcium channel, voltage-dependent, alpha 2/delta subunit 4	CACNA2D4	3,03

protein phosphatase 2, regulatory subunit B, alpha	PPP2R3A	3,03
RAS, dexamethasone-induced 1	RASD1	3,02
deafness, autosomal dominant 5	DFNA5	3,02
B-cell CLL/lymphoma 9	BCL9	3,02
CCDC144NL antisense RNA 1	CCDC144NL-AS1	3,02
anti-Mullerian hormone receptor, type II	AMHR2	3,02
cancer/testis antigen family 45, member A1	CT45A1	3,01
radixin	RDX	3,01
deoxyribonuclease I-like 1	DNASE1L1	3,01
malic enzyme 2, NAD(+)-dependent, mitochondrial	ME2	3,00
axin 2	AXIN2	3,00
mediator complex subunit 15	MED15	3,00
pleckstrin homology domain containing, family A member 6	PLEKHA6	3,00
heparan sulfate proteoglycan 2	HSPG2	2,99
GDP dissociation inhibitor 1	GDI1	2,99
Ras-related GTP binding D	RRAGD	2,99
RAD18 E3 ubiquitin protein ligase	RAD18	2,99
endoglin	ENG	2,99
supervillin	SVIL	2,98
golgin A8 family, member G	GOLGA8G	2,98
SHC (Src homology 2 domain containing) transforming protein 1	SHC1	2,98
tubulin, beta 2A class IIa	TUBB2A	2,97
retinol binding protein 5, cellular	RBP5	2,97
distal-less homeobox 1	DLX1	2,97
VPS11, CORVET/HOPS core subunit [Source:HGNC Symbol;Acc:HGNC:14583]	VPS11	2,96
cancer/testis antigen family 45, member A3; cancer/testis antigen family 45, member A4; cancer/testis antigen family 45, member A5	CT45A3; CT45A4; CT45A5	2,95
PR domain containing 7	PRDM7	2,94
striatin interacting protein 2	STRIP2	2,94
plasminogen activator, urokinase	PLAU	2,93
transmembrane protein 138	TMEM138	2,93
CKLF-like MARVEL transmembrane domain containing 3	CMTM3	2,93
exocyst complex component 1	EXOC1	2,93
phosphodiesterase 11A	PDE11A	2,93
pleckstrin homology domain containing, family M, member 3	PLEKHM3	2,93
ABL proto-oncogene 2, non-receptor tyrosine kinase	ABL2	2,92
suppressor of cytokine signaling 4	SOCS4	2,92
long intergenic non-protein coding RNA 1599	LINC01599	2,92
anoctamin 10	ANO10	2,91
golgin A7	GOLGA7	2,91

THAP9 antisense RNA 1	THAP9-AS1	2,91
nuclear envelope integral membrane protein 1	NEMP1	2,91
guanine nucleotide binding protein (G protein), gamma 12	GNG12	2,91
TBC1 domain family, member 19	TBC1D19	2,91
opioid growth factor receptor-like 1	OGFRL1	2,91
ectonucleoside triphosphate diphosphohydrolase 3	ENTPD3	2,90
enolase 2 (gamma, neuronal)	ENO2	2,90
cold shock domain containing C2, RNA binding	CSDC2	2,90
metallothionein 1E	MT1E	2,90
alkaline ceramidase 2	ACER2	2,89
carnitine palmitoyltransferase 1A (liver)	CPT1A	2,89
solute carrier family 30 (zinc transporter), member 1	SLC30A1	2,88
chromosome 12 open reading frame 4	C12orf4	2,88
pleckstrin homology domain containing, family B (evectins) member 2	PLEKHB2	2,88
chromosome 15 open reading frame 41	C15orf41	2,87
cyclin J-like	CCNJL	2,87
F-box and leucine-rich repeat protein 5	FBXL5	2,87
chromosome 1 open reading frame 94	C1orf94	2,87
fidgetin-like 1	FIGNL1	2,87
zinc finger protein 562	ZNF562	2,87
BCL2-like 13 (apoptosis facilitator)	BCL2L13	2,86
tetraspanin 31	TSPAN31	2,86
microsomal glutathione S-transferase 1	MGST1	2,86
myotubularin related protein 14	MTMR14	2,86
metallothionein 1G	MT1G	2,85
THAP domain containing, apoptosis associated protein 1	THAP1	2,85
DnaJ (Hsp40) homolog, subfamily B, member 2	DNAJB2	2,85
Rho/Rac guanine nucleotide exchange factor 2	ARHGEF2	2,84
CD63 molecule	CD63	2,84
arsA arsenite transporter, ATP-binding, homolog 1 (bacterial)	ASNA1	2,84
autophagy related 2A	ATG2A	2,84
receptor accessory protein 2	REEP2	2,83
ubiquitin specific peptidase 17-like family member 26; ubiquitin specific peptidase 17-like family member 24	USP17L26; USP17L24	2,83
ras homolog family member Q	RHOQ	2,83
LIM and cysteine-rich domains 1; long intergenic non-protein coding RNA 312	LMCD1; LINC00312	2,82
phospholipase A1 member A	PLA1A	2,82
pregnancy specific beta-1-glycoprotein 11	PSG11	2,81
zinc finger protein 528	ZNF528	2,81
cysteine rich hydrophobic domain 2	CHIC2	2,81
proteasome 26S subunit, non-ATPase 2	PSMD2	2,81

ubiquitin specific peptidase 53	USP53	2,81
dual specificity phosphatase 13	DUSP13	2,80
sulfatase modifying factor 2	SUMF2	2,80
nocturnin	NOCT	2,80
zinc finger protein 432	ZNF432	2,79
family with sequence similarity 127, member A	FAM127A	2,79
late cornified envelope 2B	LCE2B	2,79
parathyroid hormone-like hormone	PTHLH	2,79
chromosome 17 open reading frame 53	C17orf53	2,79
synovial sarcoma, X breakpoint 2B	SSX2B	2,78
vitamin D (1,25- dihydroxyvitamin D3) receptor	VDR	2,78
shisa family member 4	SHISA4	2,78
BTG family, member 3	BTG3	2,78
serine/threonine kinase 33	STK33	2,78
tectorin beta	TECTB	2,77
megakaryoblastic leukemia (translocation) 1	MKL1	2,77
lymphotoxin beta receptor (TNFR superfamily, member 3)	LTBR	2,77
cancer/testis antigen family 45, member A10	CT45A10	2,77
thyroid hormone receptor interactor 13	TRIP13	2,76
cyclin A1	CCNA1	2,76
vaccinia related kinase 3	VRK3	2,76
ADAM metallopeptidase with thrombospondin type 1 motif 2	ADAMTS2	2,76
fascin actin-bundling protein 1	FSCN1	2,76
integrin beta 4	ITGB4	2,76
sorting nexin 29	SNX29	2,75
ATPase, aminophospholipid transporter, class I, type 8B, member 3	ATP8B3	2,75
exonuclease NEF-sp; Putative RNA exonuclease NEF-sp [Source:UniProtKB/Swiss-Prot;Acc:Q96IC2]; Salzman2013 ANNOTATED, CDS, coding, OVCODE, OVERLAPTX, OVEXON, UTR3, UTR5 best transcript NM_001199053; Salzman2013 ANNOTATED, CDS, coding, INTERNAL, OVCODE, OVERLAPTX, OVEXON best transcript NM_001199053; Salzman2013 ANNOTATED, CDS, coding, OVCODE, OVERLAPTX, OVEXON, UTR3 best transcript NM_001199053; Transcript Identified by AceView, Entrez Gene ID(s) 81691; U4atac minor spliceosomal RNA [Source:RFAM;Acc:RF00618]	LOC81691; AC004381.6; U4atac	2,75
cystinosin, lysosomal cystine transporter	CTNS	2,74
BCL2-associated athanogene 3	BAG3	2,74
porcupine homolog (Drosophila)	PORCN	2,74
desmin	DES	2,74
cysteine-rich protein 1 (intestinal)	CRIP1	2,74

cytochrome P450, family 11, subfamily A, polypeptide 1	CYP11A1	2,74
zinc finger, NFX1-type containing 1	ZNFX1	2,74
BTB (POZ) domain containing 9	BTBD9	2,74
polymerase (RNA) II (DNA directed) polypeptide L, 7.6kDa	POLR2L	2,73
ring finger protein 141	RNF141	2,73
PR domain containing 4	PRDM4	2,73
protein tyrosine phosphatase, non-receptor type 21	PTPN21	2,73
fumarylacetoacetate hydrolase (fumarylacetoacetase)	FAH	2,73
metallothionein 1L (gene/pseudogene)	MT1L	2,72
G-protein signaling modulator 3	GPSM3	2,72
syndecan 3	SDC3	2,72
BLOC-1 related complex subunit 8; myocyte enhancer factor 2B; MEF2BNB-MEF2B readthrough	BORCS8; MEF2B; MEF2BNB-MEF2B	2,72
biogenesis of lysosomal organelles complex-1, subunit 2	BLOC1S2	2,72
S100 calcium binding protein P	S100P	2,72
ADP-ribosylation factor like GTPase 8B	ARL8B	2,72
hexosaminidase B (beta polypeptide)	HEXB	2,72
centrosomal protein 55kDa	CEP55	2,71
maestro heat-like repeat family member 1	MROH1	2,71
GTF2I repeat domain containing 1	GTF2IRD1	2,71
ras homolog family member F (in filopodia)	RHOF	2,71
Ras protein-specific guanine nucleotide-releasing factor 2	RASGRF2	2,71
microtubule interacting and trafficking domain containing 1	MITD1	2,70
N-terminal asparagine amidase	NTAN1	2,70
proline rich 13	PRR13	2,70
plexin A2	PLXNA2	2,70
annexin A11	ANXA11	2,70
protein tyrosine phosphatase, non-receptor type 14	PTPN14	2,70
solute carrier family 25 (mitochondrial carrier), member 16	SLC25A16	2,70
long intergenic non-protein coding RNA 598	LINC00598	2,70
B-cell CLL/lymphoma 10	BCL10	2,70
cystatin A (stefin A)	CSTA	2,70
NADH dehydrogenase (ubiquinone) complex I, assembly factor 1	NDUFAF1	2,69
echinoderm microtubule associated protein like 2; microRNA 330	EML2; MIR330	2,69
ER membrane protein complex subunit 3	EMC3	2,69
keratin associated protein 1-1	KRTAP1-1	2,69
heat shock 27kDa protein 1	HSPB1	2,69

inhibitor of DNA binding 3, dominant negative helix-loop-helix protein	ID3	2,69
ATP binding cassette subfamily A member 12	ABCA12	2,69
coagulation factor II (thrombin) receptor-like 2	F2RL2	2,69
tubulin tyrosine ligase-like family member 3	TTLL3	2,69
PDZ domain containing 11	PDZD11	2,69
KIAA1551	KIAA1551	2,68
nuclear receptor 2C2-associated protein	NR2C2AP	2,67
notch 2 N-terminal like	NOTCH2NL	2,67
antagonist of mitotic exit network 1 homolog	AMN1	2,67
sphingomyelin phosphodiesterase, acid-like 3A	SMPDL3A	2,67
solute carrier family 7 (anionic amino acid transporter light chain, xc- system), member 11	SLC7A11	2,66
endogenous retrovirus group V, member 2	ERVV-2	2,66
BMP2 inducible kinase	BMP2K	2,66
reticulon 4	RTN4	2,66
UDP-N-acetylglucosamine pyrophosphorylase 1 like 1	UAP1L1	2,65
zinc finger, MIZ-type containing 2	ZMIZ2	2,65
matrix metallopeptidase 10	MMP10	2,65
cadherin 17, LI cadherin (liver-intestine)	CDH17	2,65
TNF receptor-associated factor 1	TRAF1	2,65
Transcript Identified by AceView, Entrez Gene ID(s) 1174	AP1S1	2,65
interferon regulatory factor 9	IRF9	2,63
growth arrest and DNA-damage-inducible, alpha	GADD45A	2,63
SH3-domain kinase binding protein 1	SH3KBP1	2,63
solute carrier family 22 (organic cation transporter), member 18 antisense	SLC22A18AS	2,63
RAB10, member RAS oncogene family	RAB10	2,63
PSMC3 interacting protein	PSMC3IP	2,63
ubiquitin specific peptidase 17-like family member 18; ubiquitin specific peptidase 17-like family member 11	USP17L18; USP17L11	2,63
Shwachman-Bodian-Diamond syndrome	SBDS	2,63
neuropilin (NRP) and tolloid (TLL)-like 2	NETO2	2,62
mannose-6-phosphate receptor (cation dependent)	M6PR	2,62
calpastatin	CAST	2,62
5-nucleotidase domain containing 3	NT5DC3	2,62
gastric inhibitory polypeptide	GIP	2,62
Ras association (RalGDS/AF-6) domain family member 5	RASSF5	2,61
intraflagellar transport 22	IFT22	2,61
RAP1 GTPase activating protein 2	RAP1GAP2	2,61
mitochondrial ribosomal protein L24	MRPL24	2,60
WD repeat domain 47	WDR47	2,60

neurexophilin and PC-esterase domain family, member 3	NXPE3	2,60
CDC42 effector protein (Rho GTPase binding) 2	CDC42EP2	2,60
WAP four-disulfide core domain 3	WFDC3	2,60
apoptosis antagonizing transcription factor	AATF	2,60
STE20-related kinase adaptor alpha	STRADA	2,60
solute carrier family 9, subfamily A (NHE8, cation proton antiporter 8), member 8	SLC9A8	2,60
formin 1	FMN1	2,59
coiled-coil and C2 domain containing 1B	CC2D1B	2,59
calpain 5	CAPN5	2,59
extended synaptotagmin-like protein 1	ESYT1	2,59
transmembrane protein 55A	TMEM55A	2,59
Josephin domain containing 1	JOSD1	2,59
polymerase (RNA) III (DNA directed) polypeptide G (32kD)	POLR3G	2,59
phosphopantothenoylcysteine decarboxylase	PPCDC	2,58
LIM domain kinase 1	LIMK1	2,58
acyl-CoA dehydrogenase, very long chain	ACADVL	2,58
ST3 beta-galactoside alpha-2,3-sialyltransferase 4	ST3GAL4	2,58
retinol dehydrogenase 12 (all-trans/9-cis/11-cis)	RDH12	2,58
collagen, type I, alpha 1	COL1A1	2,58
RNA binding motif protein 18	RBM18	2,57
CSAG family, member 3	CSAG3	2,57
solute carrier family 2 (facilitated glucose transporter), member 8	SLC2A8	2,57
drebrin-like; microRNA 6837	DBNL; MIR6837	2,57
TSC22 domain family, member 2	TSC22D2	2,57
meiotic double-stranded break formation protein 1	MEI1	2,56
adaptor-related protein complex 2, beta 1 subunit	AP2B1	2,56
cysteine-rich transmembrane module containing 1	CYSTM1	2,56
S100 calcium binding protein A4	S100A4	2,56
phospholipase C, beta 1 (phosphoinositide-specific)	PLCB1	2,56
ubiquitin specific peptidase 17-like family member 22; ubiquitin specific peptidase 17-like family member 20	USP17L22; USP17L20	2,56
protein kinase, cAMP-dependent, regulatory, type I, alpha; arylsulfatase G	PRKAR1A; ARSG	2,56
KIAA0922	KIAA0922	2,56
pitrilysin metallopeptidase 1	PITRM1	2,56
cancer/testis antigen family 45, member A2	CT45A2	2,56
paralemmin 2	PALM2	2,55
cancer/testis antigen family 45, member A8	CT45A8	2,55
cancer/testis antigen family 45, member A9	CT45A9	2,55
ATPase, Ca++ transporting, type 2C, member 1	ATP2C1	2,55
REST corepressor 1	RCOR1	2,55

glutathione peroxidase 1	GPX1	2,55
NMDA receptor synaptonuclear signaling and neuronal migration factor; microRNA 7114	NSMF; MIR7114	2,54
latent transforming growth factor beta binding protein 4	LTBP4	2,54
SMAD family member 3	SMAD3	2,54
RNA terminal phosphate cyclase-like 1	RCL1	2,54
ATP binding cassette subfamily C member 2	ABCC2	2,54
pregnancy-associated plasma protein A, pappalysin 1	PAPPA	2,54
zinc finger CCCH-type containing 12C	ZC3H12C	2,53
late endosomal/lysosomal adaptor, MAPK and MTOR activator 3	LAMTOR3	2,53
centrosomal protein 135kDa	CEP135	2,53
X-ray repair complementing defective repair in Chinese hamster cells 4	XRCC4	2,53
CREB regulated transcription coactivator 2	CRTC2	2,53
CD58 molecule	CD58	2,53
bladder cancer associated protein	BLCAP	2,53
chitinase 3-like 1 (cartilage glycoprotein-39)	CHI3L1	2,53
dysbindin (dystrobrevin binding protein 1) domain containing 1	DBNDD1	2,52
upregulator of cell proliferation	URGCP	2,52
cystatin SA	CST2	2,52
zinc finger protein 382	ZNF382	2,52
zinc finger and BTB domain containing 21	ZBTB21	2,52
target of myb1 like 1 membrane trafficking protein	TOM1L1	2,52
RNA exonuclease 2	REXO2	2,52
ring finger protein (C3H2C3 type) 6	RNF6	2,52
immunoglobulin-like and fibronectin type III domain containing 1	IGFN1	2,52
Ras association (RalGDS/AF-6) domain family member 6	RASSF6	2,51
Homo sapiens uncharacterized LOC79999 (LOC79999), mRNA.; Homo sapiens uncharacterized protein ENSP00000382042 (LOC388436), mRNA.; novel transcript antisense to GRAPL	LOC79999; LOC388436; AC007952.6	2,51
CHRNA7 (cholinergic receptor, nicotinic, alpha 7, exons 5-10) and FAM7A (family with sequence similarity 7A, exons A-E) fusion	CHRFAM7A	2,51
monoamine oxidase B	MAOB	2,51
ninein (GSK3B interacting protein)	NIN	2,51
Ras-like without CAAX 1	RIT1	2,51
PDZ and LIM domain 7 (enigma)	PDLIM7	2,51
neural proliferation, differentiation and control, 1	NPDC1	2,51
cadherin-related family member 5	CDHR5	2,51

5-aminolevulinate synthase 1	ALAS1	2,51
VPS11, CORVET/HOPS core subunit [Source:HGNC Symbol;Acc:HGNC:14583]	VPS11	2,51
serine/threonine kinase 4	STK4	2,50
synovial sarcoma, X breakpoint 8	SSX8	2,50
dynactin associated protein	DYNAP	2,49
transmembrane channel like 2	TMC2	2,49
ubiquitin specific peptidase 17-like family member 13	USP17L13	2,49
metallothionein 1B; metallothionein 1C, pseudogene	MT1B; MT1CP	2,49
zinc finger, FYVE domain containing 1	ZFYVE1	2,49
target of myb1 like 2 membrane trafficking protein	TOM1L2	2,49
long intergenic non-protein coding RNA 654	LINC00654	2,49
ceramide synthase 5	CERS5	2,49
transmembrane protein 159	TMEM159	2,49
UDP-Gal:betaGlcNAc beta 1,4- galactosyltransferase, polypeptide 5	B4GALT5	2,48
linker for activation of T-cells	LAT	2,48
calpain 11	CAPN11	2,48
forkhead box P1	FOXP1	2,48
heat shock 105kDa/110kDa protein 1	HSPH1	2,48
olfactory receptor, family 51, subfamily B, member 5	OR51B5	2,48
isthmin 1, angiogenesis inhibitor	ISM1	2,48
chromosome 7 open reading frame 25	C7orf25	2,48
tetra-peptide repeat homeobox-like	TPRXL	2,47
vinculin	VCL	2,47
FH2 domain containing 1	FHDC1	2,47
chromosome 4 open reading frame 32	C4orf32	2,47
transmembrane protein 234	TMEM234	2,47
solute carrier family 48 (heme transporter), member 1	SLC48A1	2,47
solute carrier family 41 (magnesium transporter), member 2	SLC41A2	2,47
keratin 39, type I	KRT39	2,47
IZUMO1 receptor, JUNO	IZUMO1R	2,46
prefoldin subunit 1	PFDN1	2,46
family with sequence similarity 222, member B	FAM222B	2,46
AF4/FMR2 family, member 1	AFF1	2,46
zinc finger, CCHC domain containing 4	ZCCHC4	2,46
CD3d molecule, delta (CD3-TCR complex)	CD3D	2,46
family with sequence similarity 127, member C	FAM127C	2,45
runt-related transcription factor 1	RUNX1	2,45
kelch-like family member 21	KLHL21	2,45
tyrosine 3-monooxygenase/tryptophan 5-monooxygenase activation protein, eta	YWHAH	2,45
GRAM domain containing 3	GRAMD3	2,45

UDP-GlcNAc:betaGal beta-1,3-N-acetylglucosaminyltransferase 3	B3GNT3	2,44
MYST/Esa1-associated factor 6	MEAF6	2,44
telomerase-associated protein 1	TEP1	2,44
LSM3 homolog, U6 small nuclear RNA and mRNA degradation associated	LSM3	2,44
glutathione peroxidase 8 (putative)	GPX8	2,44
ubiquitin specific peptidase 17-like family member 24; ubiquitin specific peptidase 17-like family member 26; ubiquitin specific peptidase 17-like family member 5; ubiquitin specific peptidase 17-like family member 27; ubiquitin specific peptidase 17-like family member 29; ubiquitin specific peptidase 17-like family member 30	USP17L24; USP17L26; USP17L5; USP17L27; USP17L29; USP17L30	2,44
ubiquitin specific peptidase 17-like family member 25; ubiquitin specific peptidase 17-like family member 27	USP17L25; USP17L27	2,44
neuroblastoma breakpoint family, member 19	NBPF19	2,44
polo-like kinase 3	PLK3	2,44
glycerol-3-phosphate acyltransferase 4	GPAT4	2,44
kinesin light chain 2	KLC2	2,43
transmembrane protein 136	TMEM136	2,43
glucosaminyl (N-acetyl) transferase 1, core 2	GCNT1	2,43
family with sequence similarity 122B	FAM122B	2,43
FYVE and coiled-coil domain containing 1	FYCO1	2,43
family with sequence similarity 83, member G	FAM83G	2,43
annexin A2	ANXA2	2,43
centrosomal protein 89kDa	CEP89	2,42
cyclin K	CCNK	2,42
major facilitator superfamily domain containing 6	MFSD6	2,42
SAP30 binding protein	SAP30BP	2,42
CAP-GLY domain containing linker protein 1	CLIP1	2,42
dolichyl-phosphate mannosyltransferase polypeptide 2, regulatory subunit	DPM2	2,42
TAF13 RNA polymerase II, TATA box binding protein (TBP)-associated factor, 18kDa	TAF13	2,42
periplakin	PPL	2,42
doublecortin-like kinase 1	DCLK1	2,42
transmembrane channel like 8	TMC8	2,42
coiled-coil domain containing 70	CCDC70	2,41
MAS-related GPR, member X4	MRGPRX4	2,41
Kruppel-like factor 11	KLF11	2,41
natriuretic peptide receptor 1	NPR1	2,41
fucosyltransferase 8 (alpha (1,6) fucosyltransferase)	FUT8	2,41
5-nucleotidase domain containing 4	NT5DC4	2,40
failed axon connections homolog	FAXC	2,40
membrane protein, palmitoylated 3	MPP3	2,40

vacuolar protein sorting 4 homolog B (S. cerevisiae)	VPS4B	2,40
SMAD specific E3 ubiquitin protein ligase 1	SMURF1	2,40
Y box binding protein 3	YBX3	2,40
chemokine (C-X-C motif) receptor 4	CXCR4	2,40
ubiquitin specific peptidase 17-like family member 11; ubiquitin specific peptidase 17-like family member 18; ubiquitin specific peptidase 17-like family member 20	USP17L11; USP17L18; USP17L20	2,40
Berardinelli-Seip congenital lipodystrophy 2 (seipin)	BSCL2	2,40
CDC42 binding protein kinase beta (DMPK-like)	CDC42BPB	2,39
histone deacetylase 7	HDAC7	2,39
neutral sphingomyelinase activation associated factor	NSMAF	2,39
GEN1 Holliday junction 5 flap endonuclease	GEN1	2,39
MAGE family member A9; MAGE family member A9B	MAGEA9; MAGEA9B	2,39
AF4/FMR2 family, member 4	AFF4	2,38
solute carrier family 9, subfamily A (NHE1, cation proton antiporter 1), member 1	SLC9A1	2,38
ArfGAP with GTPase domain, ankyrin repeat and PH domain 2	AGAP2	2,38
ATPase, H+ transporting, lysosomal 42kDa, V1 subunit C1	ATP6V1C1	2,38
osteopetrosis associated transmembrane protein 1	OSTM1	2,37
DnaJ (Hsp40) homolog, subfamily B, member 5	DNAJB5	2,37
Hermansky-Pudlak syndrome 5	HPS5	2,37
uncoupling protein 2 (mitochondrial, proton carrier)	UCP2	2,37
LLP homolog, long-term synaptic facilitation (Aplysia)	LLPH	2,37
metallothionein 2A	MT2A	2,37
MpV17 mitochondrial inner membrane protein	MPV17	2,37
oxysterol binding protein-like 10	OSBPL10	2,37
metallothionein 1A	MT1A	2,37
meteorin, glial cell differentiation regulator-like	METRNL	2,37
chromosome 20 open reading frame 24	C20orf24	2,36
tRNA methyltransferase 1	TRMT1	2,36
plexin A3	PLXNA3	2,36
protein tyrosine phosphatase, receptor type, J	PTPRJ	2,36
ubiquitin-conjugating enzyme E2W (putative)	UBE2W	2,36
cortactin	CTTN	2,36
NLR family, pyrin domain containing 1	NLRP1	2,36
dynein, light chain, Tctex-type 1	DYNLT1	2,35
sarcoglycan beta	SGCB	2,35
mitochondrial ribosomal protein S11	MRPS11	2,35
lysine (K)-specific demethylase 1B	KDM1B	2,35
pleckstrin homology domain containing, family O member 2	PLEKHO2	2,35
zinc finger protein 365	ZNF365	2,35

ezrin	EZR	2,35
Memczak2013 ANTISENSE, CDS, coding, INTERNAL best transcript NM_001184717	TIPARP	2,35
tetraspanin 15	TSPAN15	2,35
dual specificity phosphatase 10	DUSP10	2,34
GRAM domain containing 1C	GRAMD1C	2,34
ARP3 actin-related protein 3 homolog (yeast)	ACTR3	2,34
pancreatic progenitor cell differentiation and proliferation factor	PPDPF	2,34
GRB2-related adaptor protein-like	GRAPL	2,34
protein phosphatase 2, catalytic subunit, beta isozyme	PPP2CB	2,34
general transcription factor IIIC subunit 5	GTF3C5	2,34
POP4 homolog, ribonuclease P/MRP subunit	POP4	2,34
MLX interacting protein	MLXIP	2,34
betaine--homocysteine S-methyltransferase 2	BHMT2	2,34
doublecortin domain containing 5; doublecortin domain containing 1	DCDC5; DCDC1	2,34
uncharacterized protein FLJ30679; uncharacterized protein FLJ30679 [Source:EntrezGene;Acc:146512]; novel transcript	FLJ30679; RP11-463O9.8	2,34
myeloma overexpressed	MYEOV	2,33
family with sequence similarity 122C	FAM122C	2,33
Transcript Identified by AceView, Entrez Gene ID(s) 7520	XRCC5	2,33
8-oxoguanine DNA glycosylase	OGG1	2,33
BTB and CNC homology 1, basic leucine zipper transcription factor 1	BACH1	2,33
U2AF homology motif (UHM) kinase 1	UHMK1	2,33
breast carcinoma amplified sequence 3	BCAS3	2,33
autophagy related 3	ATG3	2,33
phospholipase A2, group IVC (cytosolic, calcium-independent)	PLA2G4C	2,33
chloride intracellular channel 3	CLIC3	2,33
H2A histone family, member B3	H2AFB3	2,33
centromere protein P	CENPP	2,33
5, 3-nucleotidase, cytosolic	NT5C	2,33
zinc finger and BTB domain containing 41	ZBTB41	2,33
metallophosphoesterase 1	MPPE1	2,32
minichromosome maintenance 8 homologous recombination repair factor	MCM8	2,32
ADP-ribosylation factor like GTPase 4A	ARL4A	2,32
prion protein	PRNP	2,32
late cornified envelope 2D	LCE2D	2,32
family with sequence similarity 110, member B	FAM110B	2,32
metallothionein 3	MT3	2,32

coiled-coil domain containing 112	CCDC112	2,32
Williams Beuren syndrome chromosome region 27	WBSCR27	2,32
filamin binding LIM protein 1	FBLIM1	2,32
G protein-coupled receptor 39	GPR39	2,32
neurolysin (metallopeptidase M3 family)	NLN	2,31
tumor necrosis factor receptor superfamily, member 10a	TNFRSF10A	2,31
trafficking protein particle complex 1	TRAPPC1	2,31
notch 2 N-terminal like	NOTCH2NL	2,31
DENN/MADD domain containing 2C	DENND2C	2,30
family with sequence similarity 72, member B	FAM72B	2,30
acyl-CoA thioesterase 7	ACOT7	2,30
small VCP/p97-interacting protein	SVIP	2,30
phosphoseryl-tRNA kinase	PSTK	2,30
kallikrein related peptidase 13	KLK13	2,30
vesicle amine transport 1	VAT1	2,30
oxidative stress responsive serine-rich 1	OSER1	2,30
cap methyltransferase 2	CMTR2	2,30
primase, DNA, polypeptide 2 (58kDa)	PRIM2	2,29
ribonucleotide reductase M1	RRM1	2,29
plasminogen activator, tissue	PLAT	2,29
alpha kinase 1	ALPK1	2,29
zinc finger protein 267	ZNF267	2,29
zinc finger protein 585A	ZNF585A	2,29
poly(ADP-ribose) polymerase family member 3	PARP3	2,29
spermatogenesis associated 12	SPATA12	2,29
digestive organ expansion factor homolog (zebrafish)	DIEXF	2,29
TGIF2-C20orf24 readthrough	TGIF2-C20orf24	2,29
v-maf avian musculoaponeurotic fibrosarcoma oncogene homolog F	MAFF	2,28
ADAMTS like 5	ADAMTSL5	2,28
TatD DNase domain containing 1; microRNA 6844	TATDN1; MIR6844	2,28
phospholipid transfer protein	PLTP	2,28
KIAA0513	KIAA0513	2,28
oligonucleotide/oligosaccharide-binding fold containing 1	OBFC1	2,28
CTS telomere maintenance complex component 1	CTC1	2,28
kinesin-associated protein 3	KIFAP3	2,27
TBP-like 1	TBPL1	2,27
inhibitor of kappa light polypeptide gene enhancer in B-cells, kinase epsilon	IKBKE	2,27
UBX domain protein 8	UBXN8	2,27
tripartite motif containing 16-like	TRIM16L	2,27

ArfGAP with RhoGAP domain, ankyrin repeat and PH domain 2	ARAP2	2,27
CREB3 regulatory factor	CREBRF	2,27
gastrin-releasing peptide receptor	GRPR	2,27
PHD finger protein 11	PHF11	2,27
solute carrier family 15 (oligopeptide transporter), member 4	SLC15A4	2,27
tetratricopeptide repeat domain 1	TTC1	2,26
heat shock transcription factor 1	HSF1	2,26
KiSS-1 metastasis-suppressor	KISS1	2,26
programmed cell death 1	PDCD1	2,26
cathepsin B	CTSB	2,26
Rho family GTPase 3	RND3	2,26
sushi-repeat containing protein, X-linked	SRPX	2,26
chromosome 4 open reading frame 36	C4orf36	2,26
leucine zipper protein 1	LUZP1	2,26
thioredoxin-like 4B	TXNL4B	2,26
family with sequence similarity 72, member C	FAM72C	2,26
Transcript Identified by AceView, Entrez Gene ID(s) 114757; novel transcript, antisense to PRCD	CYGB; RP11-666A8.8	2,26
golgin A8 family, member K; ULK4 pseudogene 1	GOLGA8K; ULK4P1	2,26
phosphatidylinositol-4,5-bisphosphate 3-kinase, catalytic subunit delta	PIK3CD	2,26
ankyrin repeat and BTB (POZ) domain containing 2	ABTB2	2,26
novel transcript, antisense to C16orf53 and MVP; PAXIP1 associated glutamate-rich protein 1 [Source:HGNC Symbol;Acc:HGNC:28707]	AC009133.12; PAGR1	2,26
cyclin-dependent kinase 18	CDK18	2,25
DNA fragmentation factor, 45kDa, alpha polypeptide	DFFA	2,25
tenascin XA (pseudogene)	TNXA	2,25
sialic acid acetylesterase	SIAE	2,25
creatine kinase, mitochondrial 1B; creatine kinase, mitochondrial 1A	CKMT1B; CKMT1A	2,25
profilin 2	PFN2	2,25
praja ring finger 1, E3 ubiquitin protein ligase	PJA1	2,25
Ras and Rab interactor like	RINL	2,25
keratin associated protein 3-1	KRTAP3-1	2,25
epiplakin 1	EPPK1	2,25
platelet-activating factor acetylhydrolase 2	PAFAH2	2,25
solute carrier family 25, member 52	SLC25A52	2,25
migration and invasion enhancer 1	MIEN1	2,25
coagulation factor VIII-associated 2; coagulation factor VIII-associated 3; coagulation factor VIII-associated 1	F8A2; F8A3; F8A1	2,25
E2F transcription factor 3	E2F3	2,25

mediator complex subunit 10	MED10	2,25
CD59 molecule, complement regulatory protein	CD59	2,24
Transcript Identified by AceView, Entrez Gene ID(s) 27301	APEX2	2,24
solute carrier family 7, member 6 opposite strand	SLC7A6OS	2,24
BTB (POZ) domain containing 10	BTBD10	2,24
aldehyde dehydrogenase 3 family, member A1	ALDH3A1	2,24
Ras and Rab interactor 1	RIN1	2,24
pregnancy specific beta-1-glycoprotein 7 (gene/pseudogene)	PSG7	2,23
negative elongation factor complex member E; microRNA 1236	NELFE; MIR1236	2,23
leukotriene B4 receptor; leukotriene B4 receptor 2	LTB4R; LTB4R2	2,23
EH domain containing 4	EHD4	2,23
tumor protein p53 inducible protein 3	TP53I3	2,23
coiled-coil domain containing 84	CCDC84	2,23
ets homologous factor	EHF	2,23
pyrimidinergic receptor P2Y, G-protein coupled, 6	P2RY6	2,23
leucine rich repeat containing 8 family, member C	LRRC8C	2,23
SH3 domain binding kinase family, member 3	SBK3	2,23
zinc finger and BTB domain containing 2	ZBTB2	2,23
HAUS augmin like complex subunit 2	HAUS2	2,23
testin LIM domain protein	TES	2,23
hes family bHLH transcription factor 4	HES4	2,22
zinc finger protein 426	ZNF426	2,22
aldo-keto reductase family 1, member B1 (aldose reductase)	AKR1B1	2,22
ubiquitin-like 3	UBL3	2,22
chromosome 1 open reading frame 106	C1orf106	2,22
glycerophosphodiester phosphodiesterase domain containing 4	GDPD4	2,22
DNA-damage-inducible transcript 3	DDIT3	2,22
ATPase, H+ transporting, lysosomal 21kDa, V0 subunit b	ATP6V0B	2,22
chromosome 15 open reading frame 65	C15orf65	2,21
AMMECR1 like	AMMECR1L	2,21
adaptor-related protein complex 1, mu 1 subunit	AP1M1	2,21
FLYWCH-type zinc finger 1	FLYWCH1	2,21
tumor protein D52-like 1	TPD52L1	2,21
GTP binding protein 2	GTPBP2	2,20
glutaredoxin	GLRX	2,20
phospholipase C, beta 2	PLCB2	2,20
chemokine (C-C motif) ligand 5	CCL5	2,20
ladinin 1	LAD1	2,20
ER membrane protein complex subunit 4	EMC4	2,20
zinc finger, CCHC domain containing 8	ZCCHC8	2,20

small integral membrane protein 14	SMIM14	2,20
BLOC-1 related complex subunit 5	BORCS5	2,20
laminin, alpha 3	LAMA3	2,20
syntaxin 7	STX7	2,20
zinc finger and BTB domain containing 4	ZBTB4	2,20
serine incorporator 3	SERINC3	2,20
collagen, type XII, alpha 1	COL12A1	2,19
DENN/MADD domain containing 3	DENND3	2,19
stress-associated endoplasmic reticulum protein 1	SERP1	2,19
betaine--homocysteine S-methyltransferase	BHMT	2,19
solute carrier family 22, member 15	SLC22A15	2,19
ATPase, H+ transporting, lysosomal 9kDa, V0 subunit e1	ATP6V0E1	2,19
NADH dehydrogenase (ubiquinone) 1 beta subcomplex, 3, 12kDa	NDUFB3	2,19
ataxin 3	ATXN3	2,19
ZPR1 zinc finger	ZPR1	2,19
nuclear factor, erythroid 4	NFE4	2,19
zinc finger and BTB domain containing 43	ZBTB43	2,19
baculoviral IAP repeat containing 7	BIRC7	2,19
CD36 molecule (thrombospondin receptor)	CD36	2,18
zinc finger and SCAN domain containing 9	ZSCAN9	2,18
Memczak2013 ANTISENSE, coding, INTERNAL, intronic best transcript NM_018023	YEATS2	2,18
methyltransferase like 7B	METTL7B	2,18
tripartite motif containing 69	TRIM69	2,18
family with sequence similarity 72, member D	FAM72D	2,18
zinc finger protein 703	ZNF703	2,18
target of myb1 membrane trafficking protein	TOM1	2,18
zinc finger protein 823	ZNF823	2,18
KIAA0753	KIAA0753	2,18
transmembrane protein 133	TMEM133	2,18
adaptor-related protein complex 5, zeta 1 subunit	AP5Z1	2,18
vesicle associated membrane protein 2	VAMP2	2,18
Memczak2013 ANTISENSE, CDS, coding, INTERNAL best transcript NM_001456	FLNA	2,18
enabled homolog (Drosophila)	ENAH	2,18
sterile alpha motif domain containing 4B	SAMD4B	2,17
exophilin 5	EXPH5	2,17
MLX interacting protein	MLXIP	2,17
DDB1 and CUL4 associated factor 4	DCAF4	2,17
RAB22A, member RAS oncogene family	RAB22A	2,17
zinc finger protein 397	ZNF397	2,17
HHIP-like 2	HHIPL2	2,17
WD repeat domain 13	WDR13	2,17
autophagy related 4A, cysteine peptidase	ATG4A	2,17

guanine nucleotide binding protein (G protein), alpha 13	GNA13	2,17
protein phosphatase 2, regulatory subunit B, alpha	PPP2R2A	2,17
A kinase (PRKA) anchor protein 13; microRNA 7706	AKAP13; MIR7706	2,16
uncharacterized LOC389602; novel transcript	LOC389602; AC021218.2	2,16
keratin associated protein 4-4	KRTAP4-4	2,16
tripartite motif containing 55	TRIM55	2,16
prostaglandin I2 (prostacyclin) receptor (IP)	PTGIR	2,16
sestrin 2	SESN2	2,16
G-patch domain containing 11	GPATCH11	2,16
muscleblind like splicing regulator 1	MBNL1	2,16
3-oxoacid CoA-transferase 1	OXCT1	2,16
family with sequence similarity 72, member A	FAM72A	2,16
glutathione synthetase	GSS	2,16
COP9 signalosome subunit 8	COPS8	2,15
MOB kinase activator 3A	MOB3A	2,15
chitinase 3-like 2	CHI3L2	2,15
chromosome 16 open reading frame 46	C16orf46	2,15
SAS-6 centriolar assembly protein	SASS6	2,15
UBX domain protein 11	UBXN11	2,15
chromogranin B	CHGB	2,15
NIMA-related kinase 1	NEK1	2,15
diacylglycerol lipase, alpha	DAGLA	2,15
family with sequence similarity 103, member A1	FAM103A1	2,15
HECT domain containing E3 ubiquitin protein ligase 3	HECTD3	2,15
influenza virus NS1A binding protein	IVNS1ABP	2,15
phospholipase B domain containing 2	PLBD2	2,15
G protein-coupled receptor 55	GPR55	2,15
tubulin, beta 2B class IIb	TUBB2B	2,15
LYN proto-oncogene, Src family tyrosine kinase	LYN	2,15
NOP10 ribonucleoprotein	NOP10	2,15
dickkopf WNT signaling pathway inhibitor 1	DKK1	2,15
spermidine/spermine N1-acetyl transferase-like 1	SATL1	2,15
EPS8-like 3	EPS8L3	2,15
cytochrome b5 reductase 1	CYB5R1	2,14
upregulator of cell proliferation; URGCP-MRPS24 readthrough	URGCP; URGCP-MRPS24	2,14
CDC28 protein kinase regulatory subunit 1B	CKS1B	2,14
chromosome 11 open reading frame 1	C11orf1	2,14
family with sequence similarity 184, member B	FAM184B	2,14
phosphatidylinositol-5-phosphate 4-kinase, type II, gamma	PIP4K2C	2,14
mitogen-activated protein kinase kinase kinase 9	MAP3K9	2,14
bromodomain adjacent to zinc finger domain 1A	BAZ1A	2,14

ribonucleotide reductase M2	RRM2	2,14
ADP-ribosylation factor like GTPase 16	ARL16	2,14
N-ethylmaleimide-sensitive factor attachment protein, alpha	NAPA	2,14
cholecystokinin	CCK	2,14
PBX/knotted 1 homeobox 2	PKNOX2	2,14
T-cell leukemia translocation altered	TCTA	2,14
polycomb group ring finger 1	PCGF1	2,14
solute carrier family 41 (magnesium transporter), member 1	SLC41A1	2,14
surfeit 2 [Source:HGNC Symbol;Acc:HGNC:11475]	SURF2	2,14
complement component 5a receptor 1	C5AR1	2,14
transmembrane protein 33	TMEM33	2,14
plectin	PLEC	2,13
TNF receptor-associated factor 6, E3 ubiquitin protein ligase	TRAF6	2,13
synovial sarcoma, X breakpoint 7	SSX7	2,13
Rho GTPase activating protein 29	ARHGAP29	2,13
dehydrodolichyl diphosphate synthase subunit	DHDDS	2,13
host cell factor C2	HCFC2	2,13
spermatogenesis associated 2	SPATA2	2,13
Rho/Rac guanine nucleotide exchange factor 2	ARHGEF2	2,13
uncharacterized LOC441178; novel tanscript	LOC441178; RP11-351J23.1	2,13
ubiquitin specific peptidase 54	USP54	2,13
interleukin 7 receptor	IL7R	2,13
chromosome 14 open reading frame 2	C14orf2	2,13
keratin 80, type II	KRT80	2,13
myelin protein zero-like 2	MPZL2	2,12
exportin 6	XPO6	2,12
zinc finger, DHHC-type containing 11	ZDHHC11	2,12
coronin 7; presequence translocase-associated motor 16 homolog (S. cerevisiae); CORO7-PAM16 readthrough	CORO7; PAM16; CORO7-PAM16	2,12
zinc finger protein 778	ZNF778	2,12
protein kinase, AMP-activated, gamma 1 non-catalytic subunit	PRKAG1	2,12
phosphofurin acidic cluster sorting protein 1	PACS1	2,12
pleckstrin 2	PLEK2	2,12
actin related protein 2/3 complex subunit 2	ARPC2	2,12
tubulin, alpha-like 3	TUBAL3	2,12
antigen p97 (melanoma associated) identified by monoclonal antibodies 133.2 and 96.5	MFI2	2,12
LIM domains containing 1	LIMD1	2,12
family with sequence similarity 43, member A	FAM43A	2,12
NOP2/Sun domain family, member 6	NSUN6	2,12

pleckstrin homology-like domain, family B, member 2; phosphatidylinositol-specific phospholipase C, X domain containing 2	PHLDB2; PLCXD2	2,12
TCF3 (E2A) fusion partner (in childhood Leukemia)	TFPT	2,12
apolipoprotein O	APOO	2,12
transmembrane protein 209	TMEM209	2,12
C1QTNF9B antisense RNA 1	C1QTNF9B-AS1	2,11
WD repeat domain 41	WDR41	2,11
THAP domain containing 6	THAP6	2,11
RRN3 homolog, RNA polymerase I transcription factor	RRN3	2,11
epidermal growth factor receptor	EGFR	2,11
SH2 domain containing 2A	SH2D2A	2,11
F-box protein 33	FBXO33	2,11
breast cancer anti-estrogen resistance 3	BCAR3	2,11
latent transforming growth factor beta binding protein 2	LTBP2	2,11
transmembrane protein 185B	TMEM185B	2,11
solute carrier family 51, beta subunit	SLC51B	2,11
microtubule associated serine/threonine kinase family member 4	MAST4	2,11
bone morphogenetic protein 6	BMP6	2,10
SPOC domain containing 1	SPOCD1	2,10
ATPase, H+ transporting, lysosomal accessory protein 1-like	ATP6AP1L	2,10
nurim (nuclear envelope membrane protein)	NRM	2,10
chromodomain protein, Y-like	CDYL	2,10
SH2 domain containing 5	SH2D5	2,10
eukaryotic translation initiation factor 2B, subunit 2 beta, 39kDa	EIF2B2	2,10
RNA 3-terminal phosphate cyclase	RTCA	2,10
CWC27 spliceosome-associated protein homolog	CWC27	2,10
microtubule-associated protein 1S	MAP1S	2,10
retinoic acid receptor, alpha	RARA	2,10
zinc finger protein 461	ZNF461	2,10
synapsin I	SYN1	2,10
ADAM metallopeptidase with thrombospondin type 1 motif 9	ADAMTS9	2,10
tripartite motif containing 6	TRIM6	2,10
adaptor-related protein complex 1 sigma 1 subunit	AP1S1	2,10
vacuolar protein sorting 45 homolog (S. cerevisiae)	VPS45	2,10
cysteine-rich tail protein 1	CYSRT1	2,09
multiple EGF-like-domains 9	MEGF9	2,09
gap junction protein gamma 1	GJC1	2,09
transient receptor potential cation channel, subfamily M, member 4	TRPM4	2,09

ataxin 1	ATXN1	2,09
BCS1 homolog, ubiquinol-cytochrome c reductase complex chaperone	BCS1L	2,09
MORC family CW-type zinc finger 4	MORC4	2,09
TGFB-induced factor homeobox 1	TGIF1	2,09
zinc finger protein 706	ZNF706	2,09
solute carrier family 22 (organic anion/urate transporter), member 11	SLC22A11	2,09
aquaporin 8	AQP8	2,09
coiled-coil domain containing 74B	CCDC74B	2,09
disrupted in renal carcinoma 2	DIRC2	2,09
keratin 31, type I	KRT31	2,09
ADAM metallopeptidase domain 15	ADAM15	2,08
TNFAIP3 interacting protein 1	TNIP1	2,08
dehydrogenase/reductase (SDR family) member 2	DHRS2	2,08
X-ray radiation resistance associated 1	XRRA1	2,08
creatine kinase, mitochondrial 1A	CKMT1A	2,08
3-hydroxyisobutyrate dehydrogenase	HIBADH	2,08
v-maf avian musculoaponeurotic fibrosarcoma oncogene homolog K	MAFK	2,08
tumor necrosis factor, alpha-induced protein 2	TNFAIP2	2,08
leukocyte receptor cluster (LRC) member 8	LENG8	2,08
aspartate beta-hydroxylase	ASPH	2,08
TCDD-inducible poly(ADP-ribose) polymerase	TIPARP	2,08
aspartylglucosaminidase	AGA	2,08
myo-inositol oxygenase	MIOX	2,07
large 60S subunit nuclear export GTPase 1	LSG1	2,07
caspase 4	CASP4	2,07
solute carrier family 2 (facilitated glucose transporter), member 3	SLC2A3	2,07
interferon stimulated exonuclease gene 20kDa	ISG20	2,07
coiled-coil domain containing 80; long intergenic non-protein coding RNA 1279	CCDC80; LINC01279	2,07
long intergenic non-protein coding RNA 346	LINC00346	2,07
ZNF529 antisense RNA 1	ZNF529-AS1	2,07
cyclin Pas1/PHO80 domain containing 1	CNPPD1	2,07
cell division cycle 20	CDC20	2,07
PHD finger protein 20	PHF20	2,07
cryptochrome circadian clock 1	CRY1	2,07
ubiquitin specific peptidase 17-like family member 17	USP17L17	2,07
myosin light chain 12B	MYL12B	2,07
long intergenic non-protein coding RNA 243	LINC00243	2,07
regulator of calcineurin 1	RCAN1	2,07
tumor necrosis factor receptor superfamily, member 9	TNFRSF9	2,06

solute carrier family 16 (monocarboxylate transporter), member 1	SLC16A1	2,06
tubulin tyrosine ligase-like family member 7	TTLL7	2,06
adhesion G protein-coupled receptor G3	ADGRG3	2,06
LDL receptor related protein 12	LRP12	2,06
distal-less homeobox 3	DLX3	2,06
cytochrome b5 reductase 4	CYB5R4	2,06
killer cell lectin-like receptor subfamily C, member 1	KLRC1	2,06
DALR anticodon binding domain containing 3	DALRD3	2,06
methyltransferase like 22	METTL22	2,06
distal-less homeobox 2	DLX2	2,06
keratinocyte associated protein 2	KRTCAP2	2,06
spinster homolog 1 (Drosophila)	SPNS1	2,06
Fanconi anemia core complex associated protein 24	FAAP24	2,05
endonuclease domain containing 1	ENDOD1	2,05
charged multivesicular body protein 1B	CHMP1B	2,05
destrin (actin depolymerizing factor)	DSTN	2,05
hematological and neurological expressed 1	HN1	2,05
E2F transcription factor 7	E2F7	2,05
U-box domain containing 5; FAST kinase domains 5	UBOX5; FASTKD5	2,05
renin	REN	2,05
chloride channel, voltage-sensitive 3	CLCN3	2,05
insulin receptor substrate 1	IRS1	2,05
cyclin-dependent kinase 11B	CDK11B	2,05
GRP1 (general receptor for phosphoinositides 1)-associated scaffold protein	GRASP	2,04
anoctamin 2, calcium activated chloride channel	ANO2	2,04
PRKR interacting protein 1 (IL11 inducible)	PRKRIP1	2,04
integrator complex subunit 4	INTS4	2,04
protein kinase, AMP-activated, alpha 2 catalytic subunit	PRKAA2	2,04
prostate stem cell antigen	PSCA	2,04
cholinergic receptor, nicotinic beta 4	CHRNB4	2,04
monoacylglycerol O-acyltransferase 3	MOGAT3	2,04
SWI5-dependent homologous recombination repair protein 1	SFR1	2,04
cell division cycle 7	CDC7	2,03
heat shock 70kDa protein 6 (HSP70B)	HSPA6	2,03
epsin 2; EPN2 intronic transcript 1	EPN2; EPN2-IT1	2,03
potassium channel, voltage gated KQT-like subfamily Q, member 5	KCNQ5	2,03
regulator of chromosome condensation (RCC1) and BTB (POZ) domain containing protein 1	RCBTB1	2,03
glypican 1	GPC1	2,03
zinc finger protein 438	ZNF438	2,03
PTEN induced putative kinase 1; microRNA 6084	PINK1; MIR6084	2,03

endogenous retrovirus group 3, member 1; zinc finger protein 117	ERV3-1; ZNF117	2,03
programmed cell death 6 interacting protein	PDCD6IP	2,03
UTP4 small subunit (SSU) processome component	UTP4	2,03
replication termination factor 2 domain containing 1	RTFDC1	2,03
galactose-3-O-sulfotransferase 4	GAL3ST4	2,03
receptor (G protein-coupled) activity modifying protein 2	RAMP2	2,02
transmembrane protein 236	TMEM236	2,02
serine/threonine kinase 10	STK10	2,02
transport and golgi organization 6 homolog	TANGO6	2,02
CD151 molecule (Raph blood group)	CD151	2,02
tropomyosin 4	TPM4	2,02
pregnancy specific beta-1-glycoprotein 2	PSG2	2,02
synaptotagmin-like 3	SYTL3	2,02
Sp2 transcription factor	SP2	2,02
angiomotin like 1	AMOTL1	2,02
zinc finger protein 701; zinc finger protein 137, pseudogene	ZNF701; ZNF137P	2,02
UTP11-like, U3 small nucleolar ribonucleoprotein (yeast)	UTP11L	2,02
leucine-rich repeats and calponin homology (CH) domain containing 1	LRCH1	2,02
ATPase, H+ transporting, lysosomal 56/58kDa, V1 subunit B2	ATP6V1B2	2,02
RAN binding protein 10	RANBP10	2,02
EP300 interacting inhibitor of differentiation 3	EID3	2,01
hexosaminidase A (alpha polypeptide)	HEXA	2,01
zinc finger protein 114	ZNF114	2,01
syntaxin 18	STX18	2,01
uncharacterized LOC728485; novel transcript	LOC728485; CTD-2162K18.4	2,01
talin 1; microRNA 6852	TLN1; MIR6852	2,01
chromosome 18 open reading frame 32	C18orf32	2,01
zinc finger protein 844	ZNF844	2,01
Sjogren syndrome/scleroderma autoantigen 1	SSSCA1	2,01
nei-like DNA glycosylase 3	NEIL3	2,01
Transcript Identified by AceView, Entrez Gene ID(s) 51555	PEX5L	2,01
neurobeachin like 2	NBEAL2	2,01
pannexin 1	PANX1	2,01
protein tyrosine phosphatase, receptor type, M	PTPRM	2,01
ATPase, H+ transporting, lysosomal V0 subunit a1; microRNA 5010	ATP6V0A1; MIR5010	2,01
protein tyrosine phosphatase, non-receptor type 7	PTPN7	2,01

MICAL-like 1	MICALL1	2,01
mucin 12, cell surface associated	MUC12	2,00
family with sequence similarity 83, member H	FAM83H	2,00
TatD DNase domain containing 3	TATDN3	2,00
lin-37 DREAM MuvB core complex component	LIN37	2,00
ring finger protein, transmembrane 2	RNFT2	2,00
Tax1 (human T-cell leukemia virus type I) binding protein 1	TAX1BP1	2,00
STE20-like kinase	SLK	2,00
epithelial cell transforming 2	ECT2	2,00
DnaJ (Hsp40) homolog, subfamily C, member 24	DNAJC24	2,00
ring finger protein 213	RNF213	2,00
brain and reproductive organ-expressed (TNFRSF1A modulator)	BRE	2,00
inosine triphosphatase (nucleoside triphosphate pyrophosphatase)	ITPA	2,00
dual specificity phosphatase 8	DUSP8	2,00
bromodomain containing 9	BRD9	2,00
RAB13, member RAS oncogene family	RAB13	2,00
ubiquitin fusion degradation 1 like (yeast)	UFD1L	2,00
SKI-like proto-oncogene	SKIL	2,00
matrix metallopeptidase 20	MMP20	2,00
laminin, alpha 2	LAMA2	2,00
aryl hydrocarbon receptor	AHR	2,00
ATPase, H+ transporting, lysosomal 16kDa, V0 subunit c	ATP6V0C	1,99
folliculin	FLCN	1,99
glycosylated lysosomal membrane protein	GLMP	1,99
zinc finger protein 433	ZNF433	1,99
solute carrier family 10, member 3	SLC10A3	1,99
ankyrin repeat domain 20 family, member A1; ankyrin repeat domain 20 family, member A3	ANKRD20A1; ANKRD20A3	1,99
tripartite motif containing 22	TRIM22	1,99
aminoadipate aminotransferase	AADAT	1,99
mesothelin	MSLN	1,99
tripeptidyl peptidase I	TPP1	1,99
elongation factor Tu GTP binding domain containing 1	EFTUD1	1,99
protein phosphatase methylesterase 1	PPME1	1,99
transcription factor binding to IGHM enhancer 3	TFE3	1,99
B-cell receptor-associated protein 29	BCAP29	1,99
coagulation factor VIII-associated 2	F8A2	1,99
chromosome 2 open reading frame 76	C2orf76	1,98
phosphatidylinositol-3,4,5-trisphosphate-dependent Rac exchange factor 1	PREX1	1,98
transmembrane protein 14B	TMEM14B	1,98
RNA binding motif protein 19	RBM19	1,98

nuclear apoptosis inducing factor 1	NAIF1	1,98
TRIO and F-actin binding protein; nucleolar protein 12	TRIOBP; NOL12	1,98
chromosome 17 open reading frame 89	C17orf89	1,98
PERP, TP53 apoptosis effector	PERP	1,98
tripartite motif containing 36	TRIM36	1,98
TMEM9 domain family, member B	TMEM9B	1,98
follistatin-like 3 (secreted glycoprotein)	FSTL3	1,98
integrator complex subunit 4 pseudogene 2	INTS4P2	1,98
transglutaminase 5	TGM5	1,98
N(alpha)-acetyltransferase 30, NatC catalytic subunit	NAA30	1,98
MLX interacting protein	MLXIP	1,98
ROR1 antisense RNA 1	ROR1-AS1	1,98
mitogen-activated protein kinase 8 interacting protein 3	MAPK8IP3	1,97
transmembrane channel like 6	TMC6	1,97
nuclear receptor binding protein 2; microRNA 6845	NRBP2; MIR6845	1,97
calcium channel, voltage-dependent, T type, alpha 1H subunit	CACNA1H	1,97
TSEN2 tRNA splicing endonuclease subunit	TSEN2	1,97
ceroid-lipofuscinosis, neuronal 6, late infantile, variant	CLN6	1,97
shisa family member 5	SHISA5	1,97
N-acetylglucosamine kinase	NAGK	1,97
CD3e molecule, epsilon associated protein	CD3EAP	1,97
TBC1 domain family, member 2B	TBC1D2B	1,97
tuberous sclerosis 1	TSC1	1,97
SREK1-interacting protein 1	SREK1IP1	1,97
zinc fingers and homeoboxes 3	ZHX3	1,97
coiled-coil domain containing 84	CCDC84	1,97
adhesion G protein-coupled receptor E2	ADGRE2	1,97
microfibrillar associated protein 2	MFAP2	1,97
mitogen-activated protein kinase 8 interacting protein 2	MAPK8IP2	1,97
DNL-type zinc finger	DNLZ	1,97
transmembrane protein 127	TMEM127	1,97
nuclear protein 1, transcriptional regulator	NUPR1	1,96
NHL repeat containing E3 ubiquitin protein ligase 1	NHLRC1	1,96
tripartite motif containing 25; microRNA 3614	TRIM25; MIR3614	1,96
DAZ associated protein 2	DAZAP2	1,96
methyltransferase like 23	METTL23	1,96
actin-related protein 10 homolog (S. cerevisiae)	ACTR10	1,96
lipoyltransferase 1	LIPT1	1,96
KIAA1462	KIAA1462	1,96
alkB homolog 6	ALKBH6	1,96
uncharacterized LOC79999; Homo sapiens uncharacterized protein ENSP00000382042 (LOC388436), mRNA.; novel transcript	LOC79999; LOC388436; RP11-744A16.4	1,96

ferrochelatase	FECH	1,96
inhibitor of kappa light polypeptide gene enhancer in B-cells, kinase gamma	IKBKG	1,96
tensin 2	TNS2	1,96
vacuolar protein sorting 72 homolog (S. cerevisiae)	VPS72	1,96
charged multivesicular body protein 3	CHMP3	1,96
COX14 cytochrome c oxidase assembly factor	COX14	1,96
splicing factor 3b, subunit 4, 49kDa	SF3B4	1,96
solute carrier family 25 (mitochondrial carrier; phosphate carrier), member 24	SLC25A24	1,96
syntaxin 11	STX11	1,96
discoidin, CUB and LCCL domain containing 1	DCBLD1	1,96
pecanex-like 4 (Drosophila)	PCNXL4	1,96
ubiquitin-like domain containing CTD phosphatase 1	UBLCP1	1,96
enhancer of zeste 1 polycomb repressive complex 2 subunit	EZH1	1,95
myosin light chain 12A	MYL12A	1,95
roundabout guidance receptor 4	ROBO4	1,95
GINS complex subunit 4 (Sld5 homolog)	GINS4	1,95
ring finger protein 115	RNF115	1,95
forkhead box J3	FOXJ3	1,95
lymphocyte-specific protein 1 pseudogene 3	LSP1P3	1,95
glycolipid transfer protein	GLTP	1,95
chloride channel, voltage-sensitive 7	CLCN7	1,95
preferentially expressed antigen in melanoma	PRAME	1,95
chromosome 10 open reading frame 91	C10orf91	1,95
POTE ankyrin domain family, member I	POTEI	1,95
hydrogen voltage gated channel 1	HVCN1	1,95
cell division cycle 25B	CDC25B	1,95
apolipoprotein D	APOD	1,95
suppressor of glucose, autophagy associated 1	SOGA1	1,94
MORN repeat containing 4	MORN4	1,94
ribonucleotide reductase M2 B (TP53 inducible)	RRM2B	1,94
fucose mutarotase	FUOM	1,94
leucine rich repeat containing 16A	LRRC16A	1,94
IQ motif containing J; schwannomin interacting protein 1; IQCJ-SCHIP1 readthrough	IQCJ; SCHIP1; IQCJ-SCHIP1	1,94
myelin expression factor 2	MYEF2	1,94
coiled-coil domain containing 24	CCDC24	1,94
catsper channel auxiliary subunit gamma	CATSPERG	1,94
Nedd4 family interacting protein 1	NDFIP1	1,94
RAB21, member RAS oncogene family	RAB21	1,94
cAMP responsive element binding protein 3	CREB3	1,94
retinol dehydrogenase 10 (all-trans)	RDH10	1,94
glucose-6-phosphate dehydrogenase	G6PD	1,93

KIAA1033	KIAA1033	1,93
defensin, beta 108B	DEFB108B	1,93
nudix hydrolase 14	NUDT14	1,93
dickkopf WNT signaling pathway inhibitor 2	DKK2	1,93
growth arrest-specific 8	GAS8	1,93
agouti signaling protein	ASIP	1,93
Homo sapiens uncharacterized LOC79999 (LOC79999), mRNA.; uncharacterized protein ENSP00000382042; novel transcript	LOC79999; LOC388436; AC007952.5	1,93
cytohesin 2	CYTH2	1,93
tripartite motif containing 35	TRIM35	1,93
PTGES3L-AARSD1 readthrough; prostaglandin E synthase 3 (cytosolic)-like; alanyl-tRNA synthetase domain containing 1	PTGES3L-AARSD1; PTGES3L; AARSD1	1,93
transmembrane protein 69	TMEM69	1,93
lysozyme G-like 2	LYG2	1,93
capping protein (actin filament), gelsolin-like	CAPG	1,93
VPS26 retromer complex component A	VPS26A	1,93
adenylate cyclase 7	ADCY7	1,92
MAGE family member A2	MAGEA2	1,92
leucine-rich single-pass membrane protein 1	LSMEM1	1,92
limb development membrane protein 1-like	LMBR1L	1,92
zinc finger protein 611	ZNF611	1,92
chromosome 10 open reading frame 11	C10orf11	1,92
zinc finger CCCH-type containing 12A; microRNA 6732	ZC3H12A; MIR6732	1,92
exocyst complex component 8	EXOC8	1,92
TAF10 RNA polymerase II, TATA box binding protein (TBP)-associated factor, 30kDa	TAF10	1,92
SH3 domain containing 21	SH3D21	1,92
tropomodulin 1	TMOD1	1,91
cms1 ribosomal small subunit homolog (yeast)	CMSS1	1,91
Janus kinase 2	JAK2	1,91
ATP binding cassette subfamily C member 4	ABCC4	1,91
toll-like receptor adaptor molecule 1	TICAM1	1,91
potassium channel tetramerization domain containing 18	KCTD18	1,91
Rho GTPase activating protein 21	ARHGAP21	1,91
coagulation factor VIII-associated 3	F8A3	1,91
interleukin 23, alpha subunit p19	IL23A	1,91
ATP binding cassette subfamily B member 11	ABCB11	1,91
abhydrolase domain containing 2	ABHD2	1,91
mannosidase, beta A, lysosomal-like	MANBAL	1,91
myosin IE	MYO1E	1,91

exoribonuclease 1	ERI1	1,91
zinc finger protein 888	ZNF888	1,91
PHD finger protein 23	PHF23	1,91
TTK protein kinase	TTK	1,91
kinesin family member 21A	KIF21A	1,91
KIAA1683	KIAA1683	1,91
Jeck2013 ANTISENSE, CDS, coding, INTERNAL, OVCODE, OVEXON best transcript NM_001110556	FLNA	1,91
coiled-coil domain containing 14	CCDC14	1,91
cysteinyl-tRNA synthetase 2, mitochondrial (putative)	CARS2	1,90
ribonuclease/angiogenin inhibitor 1	RNH1	1,90
Memczak2013 ANTISENSE, CDS, coding, INTERNAL best transcript NM_001204426	LIMK1	1,90
zinc finger protein 646	ZNF646	1,90
syntaxin 8	STX8	1,90
Bardet-Biedl syndrome 1	BBS1	1,90
leucine rich repeat containing 49	LRRC49	1,90
proteasome 26S subunit, ATPase 4 [Source:HGNC Symbol;Acc:HGNC:9551]	PSMC4	1,90
dedicator of cytokinesis 5	DOCK5	1,90
microtubule-associated protein 1 light chain 3 beta 2	MAP1LC3B2	1,90
clathrin, light chain A	CLTA	1,90
6-phosphofructo-2-kinase/fructose-2,6-biphosphatase 3	PFKFB3	1,90
biglycan	BGN	1,89
salvador family WW domain containing protein 1	SAV1	1,89
myosin XVB	MYO15B	1,89
zinc finger protein 394	ZNF394	1,89
late cornified envelope 2A	LCE2A	1,89
alkB homolog 2, alpha-ketoglutarate-dependent dioxygenase	ALKBH2	1,89
zinc finger protein 808; ribosomal protein L39 pseudogene 34	ZNF808; RPL39P34	1,89
ubiquitin protein ligase E3 component n-recognin 4	UBR4	1,89
MAP/microtubule affinity-regulating kinase 3	MARK3	1,89
immediate early response 3	IER3	1,89
acyl-CoA thioesterase 9	ACOT9	1,89
testis expressed 15	TEX15	1,89
proteasome 26S subunit, non-ATPase 13	PSMD13	1,89
DnaJ (Hsp40) homolog, subfamily B, member 1	DNAJB1	1,88
sperm associated antigen 9	SPAG9	1,88
POTE ankyrin domain family, member E	POTEE	1,88
phosphatidylinositol transfer protein, alpha	PITPNA	1,88
tumor necrosis factor (ligand) superfamily, member 9	TNFSF9	1,88
nuclear receptor subfamily 1, group H, member 2	NR1H2	1,88

translocase of inner mitochondrial membrane 10 homolog (yeast)	TIMM10	1,88
nuclear factor of kappa light polypeptide gene enhancer in B-cells 2 (p49/p100)	NFKB2	1,88
patatin-like phospholipase domain containing 8	PNPLA8	1,88
RAD23 homolog A, nucleotide excision repair protein	RAD23A	1,88
sideroflexin 5	SFXN5	1,88
major facilitator superfamily domain containing 10	MFSD10	1,88
ATP binding cassette subfamily A member 7	ABCA7	1,88
itchy E3 ubiquitin protein ligase	ITCH	1,88
holocytochrome c synthase	HCCS	1,88
angiopoietin 2	ANGPT2	1,88
cyclin-dependent kinase 11A	CDK11A	1,88
DENN/MADD domain containing 5A	DENND5A	1,88
PC-esterase domain containing 1B	PCED1B	1,88
Zhang2013 ALT_ACCEPTOR, ALT_DONOR, coding, INTERNAL, intronic best transcript NM_006877	GMPR	1,87
ZFP90 zinc finger protein	ZFP90	1,87
poly(ADP-ribose) polymerase 2	PARP2	1,87
solute carrier family 2 (facilitated glucose transporter), member 14	SLC2A14	1,87
chromosome 12 open reading frame 45	C12orf45	1,87
keratin 75, type II	KRT75	1,87
oxysterol binding protein-like 7	OSBPL7	1,87
MET proto-oncogene, receptor tyrosine kinase	MET	1,87
core-binding factor, beta subunit	CBFB	1,87
solute carrier family 27 (fatty acid transporter), member 4	SLC27A4	1,87
serpin peptidase inhibitor, clade E (nexin, plasminogen activator inhibitor type 1), member 1	SERPINE1	1,87
cerebral cavernous malformation 2	CCM2	1,87
reticulon 1	RTN1	1,87
aldehyde dehydrogenase 3 family, member B1	ALDH3B1	1,87
solute carrier family 35, member E4	SLC35E4	1,87
FKBP1A-SDCBP2 readthrough (NMD candidate)	FKBP1A-SDCBP2	1,87
transmembrane protein 45B	TMEM45B	1,87
zinc finger protein 845	ZNF845	1,87
presenilin enhancer gamma secretase subunit	PSENEN	1,87
potassium channel tetramerization domain containing 5	KCTD5	1,86
regulatory subunit of type II PKA R-subunit (RIIa) domain containing 1	RIIAD1	1,86
chromosome 15 open reading frame 57; mitochondrial ribosomal protein L42 pseudogene 5	C15orf57; MRPL42P5	1,86
ubiquitin specific peptidase 4 (proto-oncogene)	USP4	1,86
Pim-1 proto-oncogene, serine/threonine kinase	PIM1	1,86

beta-1,3-glucuronyltransferase 3	B3GAT3	1,86
cholinergic receptor, nicotinic beta 1	CHRNB1	1,86
transglutaminase 4	TGM4	1,86
zinc finger and SCAN domain containing 26	ZSCAN26	1,86
nucleolar complex associated 4 homolog	NOC4L	1,86
nuclear receptor subfamily 3, group C, member 1 (glucocorticoid receptor)	NR3C1	1,86
ATP/GTP binding protein 1	AGTPBP1	1,86
xenotropic and polytropic retrovirus receptor 1	XPR1	1,85
Rho GTPase activating protein 40	ARHGAP40	1,85
keratinocyte proline-rich protein	KPRP	1,85
ArfGAP with dual PH domains 2	ADAP2	1,85
neural precursor cell expressed, developmentally down-regulated 4-like, E3 ubiquitin protein ligase	NEDD4L	1,85
abhydrolase domain containing 11	ABHD11	1,85
nitrilase family, member 2	NIT2	1,85
ribonuclease, RNase K; chromosome 17 open reading frame 49; RNASEK-C17orf49 readthrough	RNASEK; C17orf49; RNASEK-C17orf49	1,85
cadherin 24, type 2	CDH24	1,85
zinc finger and BTB domain containing 3	ZBTB3	1,85
fibrosin	FBRS	1,85
epiregulin	EREG	1,85
choroideremia-like (Rab escort protein 2)	CHML	1,85
Ras association (RalGDS/AF-6) domain family (N-terminal) member 10	RASSF10	1,85
transmembrane protein 185A	TMEM185A	1,85
thyroid hormone receptor interactor 4	TRIP4	1,84
zinc finger protein interacting with K protein 1	ZIK1	1,84
serine/threonine/tyrosine interacting-like 1	STYXL1	1,84
RAP2B, member of RAS oncogene family	RAP2B	1,84
KIAA0391	KIAA0391	1,84
AT rich interactive domain 5A (MRF1-like)	ARID5A	1,84
cell division cycle associated 7	CDCA7	1,84
centromere protein Q	CENPQ	1,84
myc target 1	MYCT1	1,84
doublecortin domain containing 2	DCDC2	1,84
ring finger protein 11	RNF11	1,84
pleckstrin homology domain containing, family F (with FYVE domain) member 1	PLEKHF1	1,84
coronin, actin binding protein, 1B	CORO1B	1,84
VPS50 EARP/GARPII complex subunit	VPS50	1,84
poly(ADP-ribose) polymerase family member 4	PARP4	1,84
nuclear transcription factor, X-box binding 1	NFX1	1,84

coiled-coil domain containing 77	CCDC77	1,84
centromere protein U	CENPU	1,84
RNF103-CHMP3 readthrough; charged multivesicular body protein 3	RNF103-CHMP3; CHMP3	1,84
integrin beta 1	ITGB1	1,84
testis expressed 10	TEX10	1,84
solute carrier family 45, member 3	SLC45A3	1,84
PDZ and LIM domain 4	PDLIM4	1,83
interleukin 20	IL20	1,83
spectrin, beta, non-erythrocytic 2	SPTBN2	1,83
tyrosyl-DNA phosphodiesterase 2	TDP2	1,83
glutaredoxin 3	GLRX3	1,83
kelch-like family member 18	KLHL18	1,83
myotubularin related protein 9	MTMR9	1,83
phospholipase A2-activating protein	PLAA	1,83
glucose-fructose oxidoreductase domain containing 1	GFOD1	1,83
signal transducer and activator of transcription 6, interleukin-4 induced	STAT6	1,83
holocarboxylase synthetase (biotin-(proprionyl-CoA-carboxylase (ATP-hydrolysing)) ligase)	HLCS	1,83
RNA binding protein with multiple splicing	RBPMS	1,83
Down syndrome critical region 3	DSCR3	1,83
spastic paraplegia 21 (autosomal recessive, Mast syndrome)	SPG21	1,83
charged multivesicular body protein 4B	CHMP4B	1,83
chromosome 12 open reading frame 43	C12orf43	1,83
dopey family member 2	DOPEY2	1,83
deltex 4, E3 ubiquitin ligase	DTX4	1,83
protein tyrosine phosphatase, non-receptor type 1	PTPN1	1,83
membrane associated ring finger 7	MARCH7	1,83
ubiquitin specific peptidase 17-like family member 3; ubiquitin specific peptidase 17-like family member 8	USP17L3; USP17L8	1,83
karyopherin alpha 4 (importin alpha 3)	KPNA4	1,83
arrestin domain containing 2	ARRDC2	1,83
junction plakoglobin	JUP	1,83
biogenesis of lysosomal organelles complex-1, subunit 3	BLOC1S3	1,83
solute carrier family 23 (ascorbic acid transporter), member 2	SLC23A2	1,82
calpain 8	CAPN8	1,82
metallothionein 1H-like 1	MT1HL1	1,82
t-complex 11, testis-specific-like 1	TCP11L1	1,82
paraoxonase 2	PON2	1,82
translocase of inner mitochondrial membrane 22 homolog (yeast)	TIMM22	1,82

VPS33B interacting protein, apical-basolateral polarity regulator, spe-39 homolog	VIPAS39	1,82
beaded filament structural protein 1, filensin	BFSP1	1,82
von Willebrand factor A domain containing 7	VWA7	1,82
BCL2-interacting killer (apoptosis-inducing)	BIK	1,82
protein phosphatase 1, regulatory subunit 7	PPP1R7	1,82
chromosome 18 open reading frame 8	C18orf8	1,82
zinc finger, C2HC-type containing 1A	ZC2HC1A	1,82
cytochrome b reductase 1	CYBRD1	1,82
trafficking protein particle complex 6B	TRAPPC6B	1,82
plexin domain containing 1	PLXDC1	1,82
transforming, acidic coiled-coil containing protein 2	TACC2	1,82
pyridine nucleotide-disulphide oxidoreductase domain 1	PYROXD1	1,82
ATPase, H+ transporting, lysosomal 34kDa, V1 subunit D	ATP6V1D	1,82
caspase recruitment domain family, member 6	CARD6	1,82
abhydrolase domain containing 12	ABHD12	1,82
Ras protein-specific guanine nucleotide-releasing factor 1	RASGRF1	1,82
dysbindin (dystrobrevin binding protein 1) domain containing 2	DBNDD2	1,82
ArfGAP with SH3 domain, ankyrin repeat and PH domain 2	ASAP2	1,82
TERF1 (TRF1)-interacting nuclear factor 2	TINF2	1,82
aryl hydrocarbon receptor nuclear translocator-like 2	ARNTL2	1,81
kelch-like family member 4	KLHL4	1,81
SEC14-like lipid binding 1; small Cajal body-specific RNA 16; small nucleolar RNA host gene 20; microRNA 6516	SEC14L1; SCARNA16; SNHG20; MIR6516	1,81
family with sequence similarity 86, member B1	FAM86B1	1,81
ornithine decarboxylase 1; small nucleolar RNA, H/ACA box 80B	ODC1; SNORA80B	1,81
discs, large homolog 3 (Drosophila)	DLG3	1,81
otoraplin	OTOR	1,81
GTP binding protein 1	GTPBP1	1,81
myomesin 3	MYOM3	1,81
SP140 nuclear body protein-like	SP140L	1,81
chromosome 11 open reading frame 86	C11orf86	1,81
family with sequence similarity 173, member B	FAM173B	1,81
Cbl proto-oncogene B, E3 ubiquitin protein ligase	CBLB	1,81
ALS2, alsin Rho guanine nucleotide exchange factor	ALS2	1,81
cyclin-dependent kinase-like 1 (CDC2-related kinase)	CDKL1	1,81
catenin (cadherin-associated protein), alpha 1	CTNNA1	1,81
ribosomal protein S6 kinase, 90kDa, polypeptide 4	RPS6KA4	1,81
zinc finger protein 142	ZNF142	1,81

chromobox homolog 6	CBX6	1,81
nucleoporin 50kDa	NUP50	1,81
coiled-coil domain containing 125	CCDC125	1,80
deleted in primary ciliary dyskinesia homolog (mouse)	DPCD	1,80
solute carrier family 38, member 1	SLC38A1	1,80
CAP, adenylate cyclase-associated protein 1 (yeast)	CAP1	1,80
ring finger protein 128, E3 ubiquitin protein ligase	RNF128	1,80
platelet-derived growth factor beta polypeptide	PDGFB	1,80
src kinase associated phosphoprotein 2	SKAP2	1,80
caspase recruitment domain family, member 19	CARD19	1,80
RNA polymerase II associated protein 1	RPAP1	1,80
synaptogyrin 4	SYNGR4	1,80
SH3 domain binding kinase family, member 2	SBK2	1,80
GLI family zinc finger 4	GLI4	1,80
cystatin B (stefin B)	CSTB	1,80
phosphoglucomutase 2-like 1	PGM2L1	1,80
tenascin XB	TNXB	1,80
TOX high mobility group box family member 4	TOX4	1,80
AKT1 substrate 1 (proline rich)	AKT1S1	1,80
zinc finger protein 274	ZNF274	1,79
EFR3 homolog B	EFR3B	1,79
serine/arginine repetitive matrix 1	SRRM1	1,79
zeta chain of T cell receptor associated protein kinase 70kDa	ZAP70	1,79
WD repeat domain 72	WDR72	1,79
cathepsin L family member 3, pseudogene	CTSL3P	1,79
epoxide hydrolase 4	EPHX4	1,79
lysophosphatidylcholine acyltransferase 1	LPCAT1	1,79
ephrin-B1	EFNB1	1,79
NCK-associated protein 1	NCKAP1	1,79
golgin A8 family, member S	GOLGA8S	1,79
FCH and double SH3 domains 1	FCHSD1	1,79
V-set and immunoglobulin domain containing 10 like	VSIG10L	1,79
GLI pathogenesis-related 2	GLIPR2	1,79
family with sequence similarity 46, member B	FAM46B	1,79
Transcript Identified by AceView, Entrez Gene ID(s) 23358	USP24	1,79
small nuclear RNA activating complex polypeptide 5	SNAPC5	1,79
charged multivesicular body protein 2A	CHMP2A	1,79
ubiquitin protein ligase E3 component n-recognin 1	UBR1	1,79
DEAD (Asp-Glu-Ala-Asp) box polypeptide 52	DDX52	1,79
SERTA domain containing 2	SERTAD2	1,79
dynein, light chain, roadblock-type 1	DYNLRB1	1,79
glutathione S-transferase zeta 1	GSTZ1	1,78
general transcription factor IIA 1	GTF2A1	1,78

bromodomain containing 2	BRD2	1,78
ZFP57 zinc finger protein	ZFP57	1,78
cysteine rich transmembrane BMP regulator 1 (chordin-like)	CRIM1	1,78
VPS9 domain containing 1	VPS9D1	1,78
RAB5C, member RAS oncogene family	RAB5C	1,78
ring finger protein 4	RNF4	1,78
late cornified envelope 1B	LCE1B	1,78
PIN2/TERF1 interacting, telomerase inhibitor 1; microRNA 1322	PINX1; MIR1322	1,78
BCL2-associated athanogene 2	BAG2	1,78
unkempt family zinc finger-like	UNKL	1,78
CDK5 regulatory subunit associated protein 1-like 1	CDKAL1	1,78
protein phosphatase 1, regulatory subunit 13 like	PPP1R13L	1,78
zinc finger protein 473	ZNF473	1,78
centrobin, centrosomal BRCA2 interacting protein	CNTROB	1,78
ring finger protein 114	RNF114	1,78
UDP-Gal:betaGlcNAc beta 1,4- galactosyltransferase, polypeptide 6	B4GALT6	1,78
myristoylated alanine-rich protein kinase C substrate	MARCKS	1,78
NSE1 homolog, SMC5-SMC6 complex component	NSMCE1	1,78
Fc fragment of IgM receptor	FCMR	1,78
myelin basic protein	MBP	1,78
sema domain, immunoglobulin domain (Ig), short basic domain, secreted, (semaphorin) 3C	SEMA3C	1,78
RAB29, member RAS oncogene family	RAB29	1,78
Myb/SANT-like DNA-binding domain containing 2	MSANTD2	1,78
Transcript Identified by AceView, Entrez Gene ID(s) 85442	KNDC1	1,78
nudE neurodevelopment protein 1; microRNA 484	NDE1; MIR484	1,78
annexin A2 receptor	ANXA2R	1,78
interleukin 10 receptor, alpha	IL10RA	1,78
collagen alpha-1(II) chain-like; novel transcript	LOC101928841; RP11-102K13.5	1,77
zinc finger protein 226	ZNF226	1,77
PBX/knotted 1 homeobox 1	PKNOX1	1,77
BTB (POZ) domain containing 1	BTBD1	1,77
cytochrome P450, family 4, subfamily F, polypeptide 11	CYP4F11	1,77
autophagy related 13	ATG13	1,77
phosphatase and actin regulator 2	PHACTR2	1,77
isocitrate dehydrogenase 3 (NAD+) beta	IDH3B	1,77
neuronal pentraxin receptor	NPTXR	1,77
lamin A/C	LMNA	1,77
Smith-Magenis syndrome chromosome region, candidate 8	SMCR8	1,77

tetra-peptide repeat homeobox 1	TPRX1	1,77
tripartite motif containing 3	TRIM3	1,77
Memczak2013 ANTISENSE, coding, INTERNAL, intronic best transcript NM_022157	RRAGC	1,77
protein phosphatase 1, regulatory subunit 10	PPP1R10	1,77
nucleolar protein 6 (RNA-associated)	NOL6	1,77
transmembrane protein 199; microRNA 4723	TMEM199; MIR4723	1,77
glutamate-rich WD repeat containing 1	GRWD1	1,77
interferon induced transmembrane protein 1	IFITM1	1,77
centrosomal protein 112kDa	CEP112	1,77
pleckstrin homology domain containing, family H (with MyTH4 domain) member 2	PLEKHH2	1,77
zinc finger protein 146	ZNF146	1,77
glycosyltransferase 1 domain containing 1	GLT1D1	1,77
UDP-glucose ceramide glucosyltransferase	UGCG	1,77
vaccinia related kinase 2	VRK2	1,77
galactose-3-O-sulfotransferase 2	GAL3ST2	1,76
Memczak2013 ANTISENSE, coding, INTERNAL, UTR3 best transcript NM_001001349	NKIRAS2	1,76
ankyrin repeat and LEM domain containing 2	ANKLE2	1,76
solute carrier family 3 (amino acid transporter heavy chain), member 2	SLC3A2	1,76
transcription elongation factor B (SIII), polypeptide 1 (15kDa, elongin C)	TCEB1	1,76
calpain, small subunit 1	CAPNS1	1,76
ADP-ribosylation factor like GTPase 6 interacting protein 5	ARL6IP5	1,76
Rho GDP dissociation inhibitor (GDI) alpha	ARHGDIA	1,76
WW domain binding protein 11	WBP11	1,76
pescadillo ribosomal biogenesis factor 1	PES1	1,76
solute carrier family 29 (equilibrative nucleoside transporter), member 1	SLC29A1	1,76
glycogen synthase kinase 3 beta	GSK3B	1,76
zinc finger protein 85	ZNF85	1,76
vacuolar protein sorting 16 homolog (S. cerevisiae)	VPS16	1,76
immunity-related GTPase family, M	IRGM	1,76
nuclear receptor subfamily 4, group A, member 3	NR4A3	1,76
KIAA0040	KIAA0040	1,76
tumor necrosis factor receptor superfamily, member 1A	TNFRSF1A	1,76
FK506 binding protein 6	FKBP6	1,76
adaptor-related protein complex 2 sigma 1 subunit	AP2S1	1,76
IWS1 homolog (S. cerevisiae)	IWS1	1,76
pleckstrin homology domain containing, family O member 1	PLEKHO1	1,76

phytanoyl-CoA 2-hydroxylase interacting protein	PHYHIP	1,76
thrombospondin 1	THBS1	1,76
calneuron 1	CALN1	1,76
G-patch domain containing 2 like	GPATCH2L	1,76
zinc finger protein 530	ZNF530	1,76
tetraspanin 14	TSPAN14	1,76
inhibitor of DNA binding 2, dominant negative helix-loop-helix protein	ID2	1,76
calpain 1, (mu/I) large subunit	CAPN1	1,75
interferon, alpha-inducible protein 27-like 2	IFI27L2	1,75
UTP14A small subunit (SSU) processome component	UTP14A	1,75
protein tyrosine phosphatase, non-receptor type 23	PTPN23	1,75
RecQ helicase-like	RECQL	1,75
EFR3 homolog A	EFR3A	1,75
carbonic anhydrase II	CA2	1,75
synuclein gamma	SNCG	1,75
SYS1-DBNDD2 readthrough (NMD candidate)	SYS1-DBNDD2	1,75
trafficking protein, kinesin binding 1	TRAK1	1,75
clarin 1	CLRN1	1,75
family with sequence similarity 160, member A1	FAM160A1	1,75
exosome component 5	EXOSC5	1,75
MLX interacting protein	MLXIP	1,75
zinc finger, DHHC-type containing 3	ZDHHC3	1,75
autophagy related 7	ATG7	1,75
arylsulfatase G	ARSG	1,75
ubiquitin specific peptidase 36	USP36	1,75
Cbl proto-oncogene-like 1, E3 ubiquitin protein ligase	CBLL1	1,75
methyltransferase like 6	METTL6	1,75
UTP15, U3 small nucleolar ribonucleoprotein, homolog (S. cerevisiae)	UTP15	1,75
sprouty RTK signaling antagonist 4	SPRY4	1,75
myosin, heavy chain 9, non-muscle	MYH9	1,75
GRINL1A complex locus 1; myocardial zonula adherens protein; polymerase (RNA) II (DNA directed) polypeptide M	GCOM1; MYZAP; POLR2M	1,75
mast cell immunoglobulin-like receptor 1	MILR1	1,75
pirin	PIR	1,75
mitogen-activated protein kinase kinase kinase 6	MAP3K6	1,75
regulatory associated protein of MTOR, complex 1	RPTOR	1,75
A-Raf proto-oncogene, serine/threonine kinase	ARAF	1,75
eukaryotic translation initiation factor 3, subunit D	EIF3D	1,75
coagulation factor VIII, procoagulant component	F8	1,75
ras homolog family member G	RHOG	1,75
caspase recruitment domain family, member 16	CARD16	1,75

Jeck2013 ANTISENSE, coding, INTERNAL, intronic best transcript NM_020973	GBA3	1,74
furin (paired basic amino acid cleaving enzyme)	FURIN	1,74
Memczak2013 ANTISENSE, CDS, coding, INTERNAL best transcript NM_001109	ADAM8	1,74
presenilin 1	PSEN1	1,74
cyclin D binding myb-like transcription factor 1	DMTF1	1,74
phosphatidylinositol-4,5-bisphosphate 3-kinase, catalytic subunit beta	PIK3CB	1,74
zinc finger protein-like 1	ZFPL1	1,74
RAS and EF-hand domain containing	RASEF	1,74
ATPase, H+ transporting, lysosomal 50/57kDa, V1 subunit H	ATP6V1H	1,74
UBX domain protein 10	UBXN10	1,74
membrane bound O-acyltransferase domain containing 2	MBOAT2	1,74
REX1, RNA exonuclease 1 homolog; microRNA 1909	REXO1; MIR1909	1,74
IQ motif containing F1	IQCF1	1,74
fibrous sheath interacting protein 1	FSIP1	1,74
defender against cell death 1	DAD1	1,74
family with sequence similarity 150, member B	FAM150B	1,74
trafficking protein, kinesin binding 2	TRAK2	1,74
solute carrier family 35, member G2	SLC35G2	1,74
steroid receptor RNA activator 1	SRA1	1,74
Ras association (RalGDS/AF-6) and pleckstrin homology domains 1	RAPH1	1,74
MDM4, p53 regulator	MDM4	1,74
golgin A8 family, member E, pseudogene; golgin A8 family, member D, pseudogene	GOLGA8EP; GOLGA8DP	1,73
cleavage stimulation factor, 3 pre-RNA, subunit 2	CSTF2	1,73
zinc finger protein 749	ZNF749	1,73
phosphate cytidylyltransferase 1, choline, alpha	PCYT1A	1,73
myosin VB	MYO5B	1,73
membrane associated ring finger 5	MARCH5	1,73
ribosome production factor 2 homolog	RPF2	1,73
WD repeat domain 26; microRNA 4742	WDR26; MIR4742	1,73
olfactory receptor, family 11, subfamily H, member 12	OR11H12	1,73
SPC24, NDC80 kinetochore complex component	SPC24	1,73
zinc finger and BTB domain containing 8 opposite strand	ZBTB8OS	1,73
repetin	RPTN	1,73
solute carrier family 4 (anion exchanger), member 1, adaptor protein	SLC4A1AP	1,73
ral guanine nucleotide dissociation stimulator-like 2	RGL2	1,73

Memczak2013 ANTISENSE, CDS, coding, INTERNAL best transcript NM_014959; Transcript Identified by AceView; novel transcript, antisense to CARD8	CTC-453G23.8; CARD8; nekera	1,73
ribosomal RNA processing 9, small subunit (SSU) processome component, homolog (yeast)	RRP9	1,73
acid phosphatase 5, tartrate resistant	ACP5	1,73
male-specific lethal 3 homolog (Drosophila)	MSL3	1,73
IQ motif and Sec7 domain 2	IQSEC2	1,73
ELMO/CED-12 domain containing 3	ELMOD3	1,73
potassium channel tetramerization domain containing 17	KCTD17	1,73
brain protein I3	BRI3	1,72
cholinergic receptor, nicotinic alpha 9	CHRNA9	1,72
mitogen-activated protein kinase kinase kinase kinase 5	MAP4K5	1,72
regulator of G-protein signaling 2	RGS2	1,72
family with sequence similarity 135, member A	FAM135A	1,72
transmembrane protein 131	TMEM131	1,72
epidermal growth factor receptor pathway substrate 8	EPS8	1,72
ATPase, H+ transporting, lysosomal 38kDa, V0 subunit d1	ATP6V0D1	1,72
AHA1, activator of heat shock 90kDa protein ATPase homolog 2 (yeast)	AHSA2	1,72
scavenger receptor class F, member 2	SCARF2	1,72
ligase IV, DNA, ATP-dependent	LIG4	1,72
ASB16 antisense RNA 1	ASB16-AS1	1,72
ataxin 2	ATXN2	1,72
Rho family GTPase 1	RND1	1,72
DnaJ (Hsp40) homolog, subfamily C, member 13	DNAJC13	1,72
SWT1 RNA endoribonuclease homolog	SWT1	1,72
histone H4 transcription factor	HINFP	1,72
DNA cross-link repair 1C	DCLRE1C	1,72
Rho GTPase activating protein 12	ARHGAP12	1,72
chemokine (C-C motif) receptor 4	CCR4	1,72
IMP (inosine 5-monophosphate) dehydrogenase 1	IMPDH1	1,72
zinc finger protein 746	ZNF746	1,72
zinc finger protein 567	ZNF567	1,72
SHC SH2-domain binding protein 1	SHCBP1	1,72
solute carrier family 9, subfamily A (NHE7, cation proton antiporter 7), member 7	SLC9A7	1,72
protein kinase (cAMP-dependent, catalytic) inhibitor gamma	PKIG	1,72
lysosomal-associated membrane protein 3	LAMP3	1,72
secernin 1	SCRN1	1,72
CEBPZ opposite strand	CEBPZOS	1,72

EBNA1 binding protein 2; microRNA 6733	EBNA1BP2; MIR6733	1,72
argonaute RISC catalytic component 2	ago-02	1,72
phosphatidylinositol 4-kinase type 2 alpha	PI4K2A	1,72
EMG1 N1-specific pseudouridine methyltransferase	EMG1	1,72
testis-specific kinase 1; microRNA 4667	TESK1; MIR4667	1,71
RAR-related orphan receptor C	RORC	1,71
early endosome antigen 1	EEA1	1,71
cadherin-like and PC-esterase domain containing 1	CPED1	1,71
zinc finger, CCHC domain containing 6	ZCCHC6	1,71
guanine nucleotide binding protein-like 2 (nucleolar)	GNL2	1,71
sperm acrosome associated 7	SPACA7	1,71
mitochondrial inner membrane organizing system 1	MINOS1	1,71
opiate receptor-like 1	OPRL1	1,71
tripartite motif containing 8	TRIM8	1,71
annexin A8-like 1; annexin A8	ANXA8L1; ANXA8	1,71
TRAF3 interacting protein 2	TRAF3IP2	1,71
TBC1 domain family, member 2B pseudogene; Transcript Identified by AceView, Entrez Gene ID(s) 646938	LOC646938	1,71
Memczak2013 ANTISENSE, coding, INTERNAL, intronic best transcript NM_001243234	TCF4	1,71
transmembrane protein 14C	TMEM14C	1,71
D-dopachrome tautomerase	DDT	1,71
KIAA0556	KIAA0556	1,71
transient receptor potential cation channel, subfamily V, member 3	TRPV3	1,71
breast cancer anti-estrogen resistance 1	BCAR1	1,71
tocopherol (alpha) transfer protein-like	TTPAL	1,71
Memczak2013 ALT_ACCEPTOR, ALT_DONOR, INTERNAL, intronic, ncRNA best transcript NR_037774	STAM	1,71
PDZ and LIM domain 5	PDLIM5	1,71
Memczak2013 ANTISENSE, CDS, coding, INTERNAL best transcript NM_003075	SMARCC2	1,71
APH1B gamma secretase subunit	APH1B	1,71
apoptosis, caspase activation inhibitor	AVEN	1,71
programmed cell death 1 ligand 2	PDCD1LG2	1,71
cancer/testis antigen family 47, member A5; cancer/testis antigen family 47, member A2; cancer/testis antigen family 47, member A1; cancer/testis antigen family 47, member A11; cancer/testis antigen family 47, member A12	CT47A5; CT47A2; CT47A1; CT47A11; CT47A12	1,71
IQ motif containing GTPase activating protein 3	IQGAP3	1,70
tuftelin interacting protein 11	TFIP11	1,70
tubulin, alpha 1a	TUBA1A	1,70

keratin associated protein 2-4	KRTAP2-4	1,70
tuftelin 1	TUFT1	1,70
UBA-like domain containing 2	UBALD2	1,70
connector enhancer of kinase suppressor of Ras 1	CNKSR1	1,70
metallothionein 1I, pseudogene	MT1IP	1,70
F-box and leucine-rich repeat protein 14	FBXL14	1,70
fasciculation and elongation protein zeta 2 (zygin II)	FEZ2	1,70
adaptor-related protein complex 2, mu 1 subunit	AP2M1	1,70
angiomotin like 2; microRNA 6827; ribosomal protein L39 pseudogene 5	AMOTL2; MIR6827; RPL39P5	1,70
COMMD3-BMI1 readthrough; BMI1 proto-oncogene, polycomb ring finger; COMM domain containing 3	COMMD3-BMI1; BMI1; COMMD3	1,70
serine palmitoyltransferase, small subunit B	SPTSSB	1,70
blood vessel epicardial substance	BVES	1,70
zinc finger, DHHC-type containing 9	ZDHHC9	1,70
EF-hand domain (C-terminal) containing 1	EFHC1	1,70
POTE ankyrin domain family, member M; POTE ankyrin domain family, member G	POTEM; POTEG	1,70
Wiskott-Aldrich syndrome-like	WASL	1,70
chromosome 4 open reading frame 36	C4orf36	1,70
fibronectin type III domain containing 4	FNDC4	1,70
eukaryotic translation initiation factor 2, subunit 1 alpha, 35kDa	EIF2S1	1,70
chromosome 5 open reading frame 30	C5orf30	1,70
regulator of G-protein signaling 14	RGS14	1,70
UDP-GlcNAc:betaGal beta-1,3-N-acetylglucosaminyltransferase 7	B3GNT7	1,70
POTE ankyrin domain family, member A	POTEA	1,70
forkhead-associated (FHA) phosphopeptide binding domain 1	FHAD1	1,70
solute carrier family 6 (neurotransmitter transporter), member 2	SLC6A2	1,70
chromosome 12 open reading frame 71	C12orf71	1,69
glucose 6 phosphatase, catalytic, 3	G6PC3	1,69
keratin associated protein 1-3	KRTAP1-3	1,69
chromosome 6 open reading frame 136	C6orf136	1,69
HERPUD family member 2	HERPUD2	1,69
sorting nexin 16	SNX16	1,69
fatty acyl-CoA reductase 2 pseudogene 1	FAR2P1	1,69
small nuclear ribonucleoprotein D2 pseudogene 2	SNRPD2P2	1,69
ATP binding cassette subfamily B member 9	ABCB9	1,69
small nuclear RNA activating complex polypeptide 4	SNAPC4	1,69
H3 histone, family 3B (H3.3B)	H3F3B	1,69
dual specificity phosphatase 14	DUSP14	1,69

MAX dimerization protein 1	MXD1	1,69
ubiquitin specific peptidase 17-like family member 21	USP17L21	1,69
zinc finger protein 79	ZNF79	1,69
poly(A) binding protein, cytoplasmic 1-like	PABPC1L	1,69
zinc finger protein 92	ZNF92	1,69
ribosomal protein S26	RPS26	1,69
methionine sulfoxide reductase B3	MSRB3	1,69
proline and serine rich 3	PROSER3	1,69
septin 7	sept-07	1,69
CDC42 effector protein (Rho GTPase binding) 3	CDC42EP3	1,68
numb homolog (Drosophila)	NUMB	1,68
ZNF816-ZNF321P readthrough	ZNF816-ZNF321P	1,68
serine/threonine kinase 3	STK3	1,68
dual specificity tyrosine-(Y)-phosphorylation regulated kinase 3	DYRK3	1,68
diaphanous-related formin 1	DIAPH1	1,68
protein inhibitor of activated STAT 3	PIAS3	1,68
bone morphogenetic protein receptor type II	BMPR2	1,68
Fas associated factor family member 2	FAF2	1,68
family with sequence similarity 134, member A	FAM134A	1,68
zinc finger, BED-type containing 2	ZBED2	1,68
nucleolar protein interacting with the FHA domain of MKI67	NIFK	1,68
DEF6 guanine nucleotide exchange factor	DEF6	1,68
olfactory receptor, family 2, subfamily A, member 7; Rho guanine nucleotide exchange factor 34, pseudogene	OR2A7; ARHGEF34P	1,68
Rho guanine nucleotide exchange factor 5	ARHGEF5	1,68
microsomal glutathione S-transferase 3	MGST3	1,68
uncharacterized LOC100288748; novel transcript, antisense to ESRP1	LOC100288748; RP11-22C11.2	1,68
seizure threshold 2 homolog (mouse)	SZT2	1,68
syntaxin 12	STX12	1,68
growth differentiation factor 15	GDF15	1,68
transmembrane protein 242	TMEM242	1,68
sialophorin	SPN	1,68
zinc finger protein 20	ZNF20	1,68
DiGeorge syndrome critical region gene 14	DGCR14	1,68
DEAD (Asp-Glu-Ala-Asp) box polypeptide 41	DDX41	1,68
allograft inflammatory factor 1	AIF1	1,68
FSHD region gene 2	FRG2	1,67
ring finger protein 185	RNF185	1,67
chromosome 5 open reading frame 34	C5orf34	1,67
Transcript Identified by AceView, Entrez Gene ID(s) 91057	CCDC34	1,67

GINS complex subunit 3 (Psf3 homolog)	GINS3	1,67
anaphase promoting complex subunit 13	ANAPC13	1,67
calcium activated nucleotidase 1	CANT1	1,67
family with sequence similarity 102, member A	FAM102A	1,67
eukaryotic translation initiation factor 5	EIF5	1,67
major facilitator superfamily domain containing 5	MFSD5	1,67
cullin 4A	CUL4A	1,67
ULK4 pseudogene 1; ULK4 pseudogene 2	ULK4P1; ULK4P2	1,67
kinesin family member C2	KIFC2	1,67
ubiquitin specific peptidase 47	USP47	1,67
dynein, axonemal, heavy chain 11	DNAH11	1,67
zwilch kinetochore protein	ZWILCH	1,67
zinc finger, DHHC-type containing 5	ZDHHC5	1,67
exosome component 1	EXOSC1	1,67
par-6 family cell polarity regulator beta	PARD6B	1,67
v-ral simian leukemia viral oncogene homolog B	RALB	1,67
serine incorporator 1	SERINC1	1,67
zinc finger protein 560	ZNF560	1,67
eukaryotic translation initiation factor 1	EIF1	1,67
RAB3D, member RAS oncogene family	RAB3D	1,67
coordinator of PRMT5, differentiation stimulator	COPRS	1,67
tRNA methyltransferase 6	TRMT6	1,67
coiled-coil domain containing 124	CCDC124	1,67
GRB2 associated regulator of MAPK1 1	GAREM1	1,67
forkhead box C1	FOXC1	1,67
chromosome 11 open reading frame 24	C11orf24	1,66
SWAP switching B-cell complex 70kDa subunit	SWAP70	1,66
TRAF interacting protein	TRAIP	1,66
ubiquitin specific peptidase 17-like family member 7	USP17L7	1,66
heme binding protein 2	HEBP2	1,66
chromosome 19 open reading frame 70	C19orf70	1,66
yrdC N(6)-threonylcarbamoyltransferase domain containing	YRDC	1,66
PHD finger protein 20-like 1	PHF20L1	1,66
proopiomelanocortin	POMC	1,66
ADP-ribosylarginine hydrolase	ADPRH	1,66
alkaline ceramidase 3	ACER3	1,66
cofilin 1 (non-muscle)	CFL1	1,66
EP300 interacting inhibitor of differentiation 1	EID1	1,66
ER membrane-associated RNA degradation	ERMARD	1,66
centrosomal protein 85kDa	CEP85	1,66
TATA box binding protein (TBP)-associated factor, RNA polymerase I, A, 48kDa	TAF1A	1,66
ubiquitin specific peptidase 17-like family member 22	USP17L22	1,66

potassium channel, calcium activated intermediate/small conductance subfamily N alpha, member 4	KCNN4	1,66
zinc finger protein 34	ZNF34	1,66
CDC like kinase 4	CLK4	1,66
shootin 1	SHTN1	1,66
WAS protein family, member 2	WASF2	1,66
enolase superfamily member 1	ENOSF1	1,66
RELT tumor necrosis factor receptor	RELT	1,66
sphingosine kinase 2	SPHK2	1,66
tryptase beta 2 (gene/pseudogene); tryptase alpha/beta 1	TPSB2; TPSAB1	1,66
folate receptor 3 (gamma)	FOLR3	1,66
jumonji domain containing 1C	JMJD1C	1,66
Transcript Identified by AceView, Entrez Gene ID(s) 56261	GPCPD1	1,66
chromosome 1 open reading frame 54	C1orf54	1,66
stonin 2	STON2	1,66
crystallin zeta	CRYZ	1,66
FBJ murine osteosarcoma viral oncogene homolog	FOS	1,66
2-5-oligoadenylate synthetase 3	OAS3	1,65
protein phosphatase, Mg2+/Mn2+ dependent, 1N (putative)	PPM1N	1,65
coiled-coil domain containing 84	CCDC84	1,65
BAI1-associated protein 2	BAIAP2	1,65
polynucleotide kinase 3-phosphatase	PNKP	1,65
G-patch domain containing 4	GPATCH4	1,65
TNFRSF1A-associated via death domain	TRADD	1,65
DDB1 and CUL4 associated factor 15	DCAF15	1,65
BCL2-like 15	BCL2L15	1,65
glucosaminyl (N-acetyl) transferase 4, core 2	GCNT4	1,65
topoisomerase (DNA) I	TOP1	1,65
synovial sarcoma, X breakpoint 9	SSX9	1,65
formin 1	FMN1	1,65
breast cancer 1, early onset	BRCA1	1,65
ganglioside induced differentiation associated protein 2	GDAP2	1,65
intestine-specific homeobox	ISX	1,65
basic helix-loop-helix family, member a15	BHLHA15	1,65
C2 calcium-dependent domain containing 3	C2CD3	1,65
autophagy related 16-like 1	ATG16L1	1,65
zinc finger, FYVE domain containing 26	ZFYVE26	1,65
alpha- and gamma-adaptin binding protein	AAGAB	1,65
brevican	BCAN	1,65
transmembrane protein 8A	TMEM8A	1,65
calreticulin 3	CALR3	1,65

potassium channel subfamily M regulatory beta subunit 4	KCNMB4	1,65
lactamase, beta 2	LACTB2	1,65
hydroxycarboxylic acid receptor 1	HCAR1	1,65
membrane magnesium transporter 1	MMGT1	1,65
low density lipoprotein receptor adaptor protein 1	LDLRAP1	1,64
adhesion regulating molecule 1	ADRM1	1,64
early growth response 2	EGR2	1,64
death-associated protein kinase 3; microRNA 637	DAPK3; MIR637	1,64
SPT20 homolog, SAGA complex component	SUPT20H	1,64
carbohydrate sulfotransferase 10	CHST10	1,64
SH3-domain binding protein 4	SH3BP4	1,64
rhomboid domain containing 1	RHBDD1	1,64
Homo sapiens family with sequence similarity 103, member A1, mRNA (cDNA clone MGC:2560 IMAGE:2989772), complete cds.; Homo sapiens family with sequence similarity 103, member A1, mRNA (cDNA clone MGC:18029 IMAGE:3924570), complete cds.; Homo sapiens family with sequence similarity 103, member A1, mRNA (cDNA clone MGC:102778 IMAGE:5578103), complete cds.	FAM103A1	1,64
DR1-associated protein 1 (negative cofactor 2 alpha)	DRAP1	1,64
MRT4 homolog, ribosome maturation factor	MRTO4	1,64
lectin, galactoside-binding, soluble, 8	LGALS8	1,64
poly(rC) binding protein 4	PCBP4	1,64
seryl-tRNA synthetase	SARS	1,64
G elongation factor, mitochondrial 2	GFM2	1,64
GTP binding protein 4	GTPBP4	1,64
protein kinase C, zeta	PRKCZ	1,64
transmembrane protein 184C	TMEM184C	1,64
DNA cross-link repair 1B	DCLRE1B	1,64
flightless I actin binding protein	FLII	1,64
tumor protein D52-like 2	TPD52L2	1,64
calbindin 2	CALB2	1,64
vacuolar protein sorting 39 homolog (S. cerevisiae)	VPS39	1,64
protein phosphatase 3, catalytic subunit, gamma isozyme	PPP3CC	1,64
leucine rich repeat (in FLII) interacting protein 2	LRRFIP2	1,64
arginine/serine-rich protein 1	RSRP1	1,64
annexin A5	ANXA5	1,64
potassium channel, voltage gated eag related subfamily H, member 3	KCNH3	1,64
thioredoxin	TXN	1,64
matrix metallopeptidase 7	MMP7	1,64

Transcript Identified by AceView, Entrez Gene ID(s) 728833	FAM72D	1,64
mitogen-activated protein kinase 11	MAPK11	1,63
ets variant 3	ETV3	1,63
mirror-image polydactyly 1	MIPOL1	1,63
C-type lectin domain family 16, member A	CLEC16A	1,63
family with sequence similarity 53, member C	FAM53C	1,63
interferon, alpha 14	IFNA14	1,63
long intergenic non-protein coding RNA 337	LINC00337	1,63
syntaxin binding protein 3	STXBP3	1,63
myotubularin related protein 6	MTMR6	1,63
ATP binding cassette subfamily C member 10	ABCC10	1,63
linker for activation of T-cells family member 2	LAT2	1,63
small proline-rich protein 2A	SPRR2A	1,63
formin binding protein 1-like	FNBP1L	1,63
poliovirus receptor	PVR	1,63
cyclin T1	CCNT1	1,63
nuclear RNA export factor 1	NXF1	1,63
tripartite motif containing 32	TRIM32	1,63
proteasome 26S subunit, non-ATPase 14	PSMD14	1,63
RNA binding motif, single stranded interacting protein 3	RBMS3	1,63
tyrosine 3-monooxygenase/tryptophan 5-monooxygenase activation protein, zeta	YWHAZ	1,63
zinc finger protein 519	ZNF519	1,63
desmoglein 2	DSG2	1,63
carboxypeptidase A1 (pancreatic)	CPA1	1,63
zinc finger protein 256	ZNF256	1,63
family with sequence similarity 110, member C	FAM110C	1,63
Zic family member 5	ZIC5	1,63
ubiquitin conjugating enzyme E2D 1	UBE2D1	1,63
NEDD4 binding protein 1	N4BP1	1,63
FK506 binding protein 1A; microRNA 6869	FKBP1A; MIR6869	1,63
cytochrome P450, family 3, subfamily A, polypeptide 4	CYP3A4	1,63
protein kinase, cGMP-dependent, type II	PRKG2	1,63
coenzyme Q4	COQ4	1,63
coiled-coil domain containing 179	CCDC179	1,63
solute carrier family 12 (sodium/potassium/chloride transporter), member 2	SLC12A2	1,63
SH3-domain GRB2-like endophilin B2	SH3GLB2	1,63
adaptor-related protein complex 3, sigma 2 subunit; microRNA 5009	AP3S2; MIR5009	1,63
histone cluster 1, H2bk	HIST1H2BK	1,63
mitogen-activated protein kinase associated protein 1	MAPKAP1	1,63

Homo sapiens ribosomal protein S26, mRNA (cDNA clone MGC:27148 IMAGE:4807004), complete cds.; Homo sapiens ribosomal protein S26, mRNA (cDNA clone MGC:88201 IMAGE:6388128), complete cds.; Homo sapiens ribosomal protein S26, mRNA (cDNA clone MGC:104291 IMAGE:4287636), complete cds.; Homo sapiens ribosomal protein S26, mRNA (cDNA clone MGC:104292 IMAGE:6726218), complete cds.	RPS26	1,63
ribonuclease L (2,5-oligoisoadenylate synthetase-dependent)	RNASEL	1,63
EF-hand calcium binding domain 13	EFCAB13	1,63
SATB homeobox 2	SATB2	1,62
MAX network transcriptional repressor	MNT	1,62
RAD51 paralog C	RAD51C	1,62
ring finger protein 43	RNF43	1,62
Vac14 homolog (S. cerevisiae)	VAC14	1,62
actin filament associated protein 1	AFAP1	1,62
cerebellar degeneration-related protein 2-like	CDR2L	1,62
WD repeat domain 53	WDR53	1,62
mitochondrial ribosomal protein L21	MRPL21	1,62
syntaxin 16	STX16	1,62
survival of motor neuron 1, telomeric	SMN1	1,62
NIPA-like domain containing 1	NIPAL1	1,62
dynein, axonemal, assembly factor 1	DNAAF1	1,62
folliculin interacting protein 1	FNIP1	1,62
chromosome alignment maintaining phosphoprotein 1	CHAMP1	1,62
FGGY carbohydrate kinase domain containing	FGGY	1,62
carbohydrate (N-acetylglucosamine-6-O) sulfotransferase 2	CHST2	1,62
abhydrolase domain containing 5	ABHD5	1,62
ATPase type 13A3	ATP13A3	1,62
RAB5A, member RAS oncogene family	RAB5A	1,62
basic helix-loop-helix and HMG box domain containing 1	BHMG1	1,62
myosin light chain 6	MYL6	1,62
pleckstrin homology domain containing, family M (with RUN domain) member 2	PLEKHM2	1,62
THAP domain containing 10	THAP10	1,62
meningioma expressed antigen 5 (hyaluronidase)	MGEA5	1,62
histidyl-tRNA synthetase 2, mitochondrial	HARS2	1,62
BCL2-associated agonist of cell death	BAD	1,62
zinc finger protein 335	ZNF335	1,62
amyotrophic lateral sclerosis 2 chromosome region candidate 12	ALS2CR12	1,62
tropomyosin 3	TPM3	1,62
zinc finger protein 730	ZNF730	1,62

EF-hand domain family, member B	EFHB	1,62
von Willebrand factor A domain containing 5A	VWA5A	1,62
PAK1 interacting protein 1	PAK1IP1	1,62
autophagy related 16-like 1	ATG16L1	1,62
prolyl endopeptidase-like	PREPL	1,61
late cornified envelope 1B	LCE1B	1,61
CDC28 protein kinase regulatory subunit 2	CKS2	1,61
axin interactor, dorsalization associated	AIDA	1,61
neuroblastoma 1, DAN family BMP antagonist	NBL1	1,61
zinc finger protein 557	ZNF557	1,61
kinesin heavy chain member 2A	KIF2A	1,61
cathepsin D	CTSD	1,61
stanniocalcin 1	STC1	1,61
sorting nexin 8; microRNA 6836	SNX8; MIR6836	1,61
SAP domain containing ribonucleoprotein	SARNP	1,61
mannosyl (alpha-1,3-)-glycoprotein beta-1,4-N-acetylglucosaminyltransferase, isozyme B	MGAT4B	1,61
eukaryotic translation initiation factor 1B	EIF1B	1,61
gamma-aminobutyric acid (GABA) A receptor, beta 3	GABRB3	1,61
lysophospholipase II	LYPLA2	1,61
keratin 15, type I; microRNA 6510	KRT15; MIR6510	1,61
ubiquitin specific peptidase 15; microRNA 6125	USP15; MIR6125	1,61
regenerating islet-derived 1 beta	REG1B	1,61
rhomboid domain containing 2	RHBDD2	1,61
TYRO protein tyrosine kinase binding protein	TYROBP	1,61
sarcoglycan alpha	SGCA	1,61
mitochondrial ribosomal protein S10	MRPS10	1,61
casein kinase 1, delta	CSNK1D	1,61
tumor protein p53 binding protein 1	TP53BP1	1,61
peptidylprolyl isomerase H (cyclophilin H)	PPIH	1,61
serine/threonine kinase 32C	STK32C	1,61
RAB11A, member RAS oncogene family	RAB11A	1,61
p21 protein (Cdc42/Rac)-activated kinase 2	PAK2	1,61
ADP-ribosylation factor related protein 1	ARFRP1	1,61
V-set and immunoglobulin domain containing 2	VSIG2	1,61
denticleless E3 ubiquitin protein ligase homolog (Drosophila)	DTL	1,61
phosphodiesterase 6C, cGMP-specific, cone, alpha prime	PDE6C	1,61
phosducin like 3	PDCL3	1,61
hypoxia inducible factor 1, alpha subunit (basic helix-loop-helix transcription factor)	HIF1A	1,61
Memczak2013 ALT_ACCEPTOR, ALT_DONOR, coding, INTERNAL, intronic best transcript NM_020772; Transcript Identified by AceView	NUFIP2; rerdy	1,61

WD repeat domain 77	WDR77	1,61
mutS homolog 5; MSH5-SAPCD1 readthrough (NMD candidate); suppressor APC domain containing 1	MSH5; MSH5-SAPCD1; SAPCD1	1,61
methyl-CpG binding domain protein 1	MBD1	1,61
DNA-damage regulated autophagy modulator 2	DRAM2	1,61
epithelial splicing regulatory protein 2; microRNA 6773	ESRP2; MIR6773	1,60
serine/threonine kinase 38	STK38	1,60
filaggrin	FLG	1,60
M-phase phosphoprotein 6	MPHOSPH6	1,60
zinc finger and SCAN domain containing 21	ZSCAN21	1,60
interleukin 10	IL10	1,60
ATPase, H+ transporting, lysosomal 38kDa, V0 subunit d2	ATP6V0D2	1,60
transmembrane protein 50A	TMEM50A	1,60
glucokinase (hexokinase 4) regulator	GCKR	1,60
developmentally regulated GTP binding protein 2	DRG2	1,60
GEM interacting protein	GMIP	1,60
K(lysine) acetyltransferase 2B	KAT2B	1,60
zinc finger protein 260	ZNF260	1,60
fatty acid 2-hydroxylase	FA2H	1,60
proteasome subunit beta 3	PSMB3	1,60
synovial sarcoma, X breakpoint 4B; synovial sarcoma, X breakpoint 4	SSX4B; SSX4	1,60
CDKN1A interacting zinc finger protein 1	CIZ1	1,60
v-rel avian reticuloendotheliosis viral oncogene homolog B	RELB	1,60
DET1 and DDB1 associated 1	DDA1	1,60
karyopherin alpha 3 (importin alpha 4)	KPNA3	1,60
annexin A7	ANXA7	1,60
myeloid leukemia factor 1	MLF1	1,60
CD40 molecule, TNF receptor superfamily member 5	CD40	1,60
MLX interacting protein	MLXIP	1,60
interleukin 17 receptor D	IL17RD	1,60
SPT5 homolog, DSIF elongation factor subunit	SUPT5H	1,60
solute carrier family 16, member 14	SLC16A14	1,60
cytoplasmic polyadenylation element binding protein 2	CPEB2	1,60
thioredoxin domain containing 17	TXNDC17	1,60
solute carrier family 25 (aspartate/glutamate carrier), member 12	SLC25A12	1,60
formin 1	FMN1	1,60
5-hydroxytryptamine (serotonin) receptor 3C, ionotropic	HTR3C	1,60
HSPA (heat shock 70kDa) binding protein, cytoplasmic cochaperone 1	HSPBP1	1,60
kelch-like family member 12	KLHL12	1,60

protein kinase, AMP-activated, gamma 2 non-catalytic subunit	PRKAG2	1,60
ankyrin repeat domain 20 family, member A1	ANKRD20A1	1,60
beta-1,4-N-acetyl-galactosaminyl transferase 1	B4GALNT1	1,60
CD44 molecule (Indian blood group)	CD44	1,60
zinc finger protein 506	ZNF506	1,60
POTE ankyrin domain family, member J	POTEJ	1,60
UbiA prenyltransferase domain containing 1	UBIAD1	1,60
nuclear pore complex interacting protein family, member B11	NPIPB11	1,59
poly(rC) binding protein 3	PCBP3	1,59
Zhang2013 ALT_ACCEPTOR, ALT_DONOR, coding, INTERNAL, intronic best transcript NM_002224	ITPR3	1,59
zinc finger protein 81	ZNF81	1,59
caudal type homeobox 2	CDX2	1,59
HEAT repeat containing 5B	HEATR5B	1,59
PR domain containing 2, with ZNF domain	PRDM2	1,59
ubiquitin-conjugating enzyme E2E 3	UBE2E3	1,59
twinfilin actin binding protein 1	TWF1	1,59
methylphosphate capping enzyme	MEPCE	1,59
ELK1, member of ETS oncogene family	ELK1	1,59
GPN-loop GTPase 1	GPN1	1,59
popeye domain containing 2	POPDC2	1,59
zinc finger homeobox 4	ZFHX4	1,59
adhesion molecule, interacts with CXADR antigen 1	AMICA1	1,59
UEV and lactate/malate dehyrogenase domains	UEVLD	1,59
UV radiation resistance associated	UVRAG	1,59
single-stranded DNA binding protein 1, mitochondrial	SSBP1	1,59
inositol polyphosphate-5-phosphatase E	INPP5E	1,59
family with sequence similarity 9, member C	FAM9C	1,59
WAS/WASL interacting protein family, member 2	WIPF2	1,59
huntingtin	HTT	1,59
TSR3, 20S rRNA accumulation, homolog (S. cerevisiae)	TSR3	1,59
oral cancer overexpressed 1	ORAOV1	1,59
sterile alpha motif domain containing 4A	SAMD4A	1,59
coiled-coil-helix-coiled-coil-helix domain containing 10	CHCHD10	1,59
nuclear factor I/B	NFIB	1,59
ankyrin repeat domain 13 family, member D	ANKRD13D	1,59
four jointed box 1	FJX1	1,59
biliverdin reductase A	BLVRA	1,59
5-3 exoribonuclease 2	XRN2	1,59
sperm antigen with calponin homology and coiled-coil domains 1	SPECC1	1,59
myozenin 2	MYOZ2	1,59
isocitrate dehydrogenase 3 (NAD+) gamma	IDH3G	1,59

Memczak2013 ALT_ACCEPTOR, ALT_DONOR, coding, INTERNAL, intronic best transcript NM_001098797	TOX2	1,59
ring finger protein 146	RNF146	1,58
polymerase (DNA-directed), epsilon 4, accessory subunit	POLE4	1,58
BAI1-associated protein 2-like 2	BAIAP2L2	1,58
ubiquitin specific peptidase 12	USP12	1,58
ring finger protein 167	RNF167	1,58
peroxisome proliferator-activated receptor gamma, coactivator 1 beta	PPARGC1B	1,58
late cornified envelope 1F	LCE1F	1,58
potassium channel, voltage gated subfamily E regulatory beta subunit 5; acyl-CoA synthetase long-chain family member 4	KCNE5; ACSL4	1,58
NOC2-like nucleolar associated transcriptional repressor	NOC2L	1,58
striatin, calmodulin binding protein 3	STRN3	1,58
Memczak2013 ANTISENSE, coding, INTERNAL, UTR3 best transcript NM_004475	FLOT2	1,58
cathepsin A	CTSA	1,58
missing oocyte, meiosis regulator, homolog (Drosophila)	MIOS	1,58
breast cancer metastasis suppressor 1	BRMS1	1,58
GDNF-inducible zinc finger protein 1	GZF1	1,58
syndecan 1	SDC1	1,58
phospholipid phosphatase related 2	PLPPR2	1,58
perilipin 3	PLIN3	1,58
coiled-coil domain containing 82	CCDC82	1,58
vacuolar protein sorting 8 homolog (S. cerevisiae)	VPS8	1,58
centromere protein O	CENPO	1,58
LIM and SH3 protein 1	LASP1	1,58
ring finger protein 145	RNF145	1,58
chorionic somatomammotropin hormone 2	CSH2	1,58
capping protein (actin filament) muscle Z-line, alpha 1	CAPZA1	1,58
leucine-rich repeat LGI family, member 3	LGI3	1,58
ELF1 homolog, elongation factor 1	ELOF1	1,58
bridging integrator 3	BIN3	1,58
zinc finger protein 850	ZNF850	1,58
RAS p21 protein activator 2	RASA2	1,58
nitrilase 1	NIT1	1,58
general transcription factor IIE subunit 2	GTF2E2	1,58
SET domain containing 1B	SETD1B	1,58
zinc finger and BTB domain containing 17	ZBTB17	1,58
LIM homeobox 1	LHX1	1,58
transmembrane protein 192	TMEM192	1,58
Yip1 domain family member 3	YIPF3	1,58

NME/NM23 nucleoside diphosphate kinase 6	NME6	1,58
protein phosphatase 2, regulatory subunit B, gamma	PPP2R3C	1,58
protein phosphatase 2, regulatory subunit B, epsilon isoform	PPP2R5E	1,58
zinc finger protein 534	ZNF534	1,58
C-type lectin domain family 7, member A	CLEC7A	1,58
chromosome 12 open reading frame 60	C12orf60	1,58
mitochondrial ribosomal protein L18	MRPL18	1,58
protein phosphatase 5, catalytic subunit	PPP5C	1,58
branched chain keto acid dehydrogenase E1, alpha polypeptide	BCKDHA	1,58
coiled-coil domain containing 61	CCDC61	1,58
integrin alpha 6	ITGA6	1,58
inhibitor of kappa light polypeptide gene enhancer in B-cells, kinase beta	IKBKB	1,58
cell division cycle associated 5	CDCA5	1,58
ring finger protein 38	RNF38	1,58
dystrophia myotonica-protein kinase	DMPK	1,58
lipoma HMGIC fusion partner-like 4	LHFPL4	1,58
CD274 molecule	CD274	1,58
RIMS binding protein 3C; RIMS binding protein 3B	RIMBP3C; RIMBP3B	1,58
Jeck2013 ANTISENSE, coding, INTERNAL, OVEXON, UTR3 best transcript NM_001007098	SCP2	1,58
tyrosyl-tRNA synthetase	YARS	1,57
Transcript Identified by AceView, Entrez Gene ID(s) 22874	PLEKHA6	1,57
pre-B-cell leukemia homeobox 4	PBX4	1,57
phosphatidylinositol glycan anchor biosynthesis class T	PIGT	1,57
pancreatic polypeptide	PPY	1,57
small integral membrane protein 13	SMIM13	1,57
zinc finger protein 280B	ZNF280B	1,57
heat shock 70kDa protein 1-like	HSPA1L	1,57
chromosome 6 open reading frame 52	C6orf52	1,57
claudin 5	CLDN5	1,57
stromal interaction molecule 2	STIM2	1,57
UBX domain protein 1	UBXN1	1,57
sialic acid binding Ig-like lectin 9	SIGLEC9	1,57
Bloom syndrome, RecQ helicase-like	BLM	1,57
phosphodiesterase 6G, cGMP-specific, rod, gamma	PDE6G	1,57
voltage-dependent anion channel 2	VDAC2	1,57
yippee like 5	YPEL5	1,57
Rho GDP dissociation inhibitor (GDI) beta	ARHGDIB	1,57
ELOVL fatty acid elongase 4	ELOVL4	1,57
leucine rich repeat containing 4C	LRRC4C	1,57

Ras association (RalGDS/AF-6) domain family (N-terminal) member 8	RASSF8	1,57
F-box and leucine-rich repeat protein 13	FBXL13	1,57
autophagy related 16-like 1	ATG16L1	1,57
exosome component 9	EXOSC9	1,57
chromosome X open reading frame 23	CXorf23	1,57
apoptogenic 1, mitochondrial	APOPT1	1,57
leucine-rich repeats and calponin homology (CH) domain containing 4	LRCH4	1,57
thioredoxin reductase 2	TXNRD2	1,57
MAP7 domain containing 1	MAP7D1	1,57
beclin 1, autophagy related	BECN1	1,57
cell division cycle 16	CDC16	1,57
pre-B-cell leukemia homeobox 3	PBX3	1,57
zinc finger protein 827	ZNF827	1,57
chromosome 10 open reading frame 113	C10orf113	1,57
melanophilin	MLPH	1,57
cell division cycle 14A	CDC14A	1,57
shugoshin-like 1 (S. pombe)	SGOL1	1,57
lymphocyte antigen 6 complex, locus D	LY6D	1,57
RIB43A domain with coiled-coils 2	RIBC2	1,57
Ral GTPase activating protein, beta subunit (non-catalytic)	RALGAPB	1,57
protein arginine methyltransferase 3	PRMT3	1,57
adenosine deaminase, tRNA-specific 1	ADAT1	1,57
zinc finger protein 18	ZNF18	1,57
RUN domain containing 3A	RUNDC3A	1,57
protein phosphatase, Mg2+/Mn2+ dependent, 1J	PPM1J	1,56
kinesin family member 14	KIF14	1,56
laminin, beta 4	LAMB4	1,56
epithelial membrane protein 2	EMP2	1,56
zinc finger protein 317	ZNF317	1,56
CD82 molecule	CD82	1,56
granulin	GRN	1,56
calcium binding protein 39	CAB39	1,56
chemokine (C-C motif) ligand 24	CCL24	1,56
ALG8, alpha-1,3-glucosyltransferase	ALG8	1,56
chromosome 16 open reading frame 89	C16orf89	1,56
regulator of G-protein signaling 6	RGS6	1,56
rotatin	RTTN	1,56
nudix hydrolase 1	NUDT1	1,56
Transcript Identified by AceView, Entrez Gene ID(s) 6845	VAMP7	1,56
S100 calcium binding protein A7	S100A7	1,56
TBC1 domain family, member 8 (with GRAM domain)	TBC1D8	1,56

metastasis associated 1 family member 2	MTA2	1,56
oxysterol binding protein-like 3	OSBPL3	1,56
abhydrolase domain containing 14B	ABHD14B	1,56
zinc finger protein 143	ZNF143	1,56
poly(A) polymerase gamma	PAPOLG	1,56
coiled-coil domain containing 122	CCDC122	1,56
polymerase (DNA directed), eta	POLH	1,56
mastermind-like domain containing 1	MAMLD1	1,56
mitochondrial carrier 1	MTCH1	1,56
cancer/testis antigen family 47, member A1	CT47A1	1,56
egl-9 family hypoxia-inducible factor 2	EGLN2	1,56
ribonuclease P/MRP 21kDa subunit	RPP21	1,56
basic helix-loop-helix family, member e40	BHLHE40	1,56
chemokine (C-X-C motif) receptor 2	CXCR2	1,56
pleckstrin homology domain containing, family G (with RhoGef domain) member 3	PLEKHG3	1,56
heat shock 27kDa protein 2; chromosome 11 open reading frame 52; HSPB2-C11orf52 readthrough (NMD candidate)	HSPB2; C11orf52; HSPB2-C11orf52	1,56
IQ motif containing K	IQCK	1,56
inositol-trisphosphate 3-kinase C	ITPKC	1,56
short stature homeobox 2	SHOX2	1,56
dickkopf WNT signaling pathway inhibitor 3	DKK3	1,55
myosin VI	MYO6	1,55
lysophosphatidylcholine acyltransferase 4	LPCAT4	1,55
transportin 3	TNPO3	1,55
myeloid cell leukemia 1	MCL1	1,55
lactalbumin, alpha-	LALBA	1,55
interleukin 23 receptor	IL23R	1,55
F-box and WD repeat domain containing 7, E3 ubiquitin protein ligase	FBXW7	1,55
Memczak2013 ALT_ACCEPTOR, ALT_DONOR, coding, INTERNAL, intronic best transcript NM_015275	KIAA1033	1,55
Salzman2013 ANNOTATED, CDS, coding, OVCODE, OVEXON, UTR3 best transcript NM_000947	PRIM2	1,55
cyclin-dependent kinase 17	CDK17	1,55
autophagy related 16-like 1	ATG16L1	1,55
SPRY domain containing 7	SPRYD7	1,55
calcium channel, voltage-dependent, P/Q type, alpha 1A subunit	CACNA1A	1,55
NPL4 homolog, ubiquitin recognition factor	NPLOC4	1,55
SPT4 homolog, DSIF elongation factor subunit	SUPT4H1	1,55
retinol saturase (all-trans-retinol 13,14-reductase)	RETSAT	1,55
golgi to ER traffic protein 4	GET4	1,55
pyridoxamine 5-phosphate oxidase	PNPO	1,55

myosin binding protein H-like	MYBPHL	1,55
Src-like-adaptor	SLA	1,55
MAGE family member B2	MAGEB2	1,55
X-linked inhibitor of apoptosis, E3 ubiquitin protein ligase	XIAP	1,55
chromosome 2 open reading frame 74	C2orf74	1,55
VPS11, CORVET/HOPS core subunit [Source:HGNC Symbol;Acc:HGNC:14583]	VPS11	1,55
autophagy related 16-like 1	ATG16L1	1,55
transmembrane protein 87A	TMEM87A	1,55
spermatogenesis associated, serine-rich 2-like	SPATS2L	1,55
Memczak2013 ANTISENSE, CDS, coding, INTERNAL best transcript NM_174956	ATP2A3	1,55
D-dopachrome tautomerase-like; D-dopachrome tautomerase	DDTL; DDT	1,55
potassium channel, voltage gated subfamily A regulatory beta subunit 2	KCNAB2	1,55
NOC3-like DNA replication regulator	NOC3L	1,55
survival of motor neuron 1, telomeric; survival of motor neuron 2, centromeric	SMN1; SMN2	1,55
exosome component 10	EXOSC10	1,55
Transcript Identified by AceView, Entrez Gene ID(s) 401067	IQCF3	1,54
Homo sapiens ribosomal protein S26, mRNA (cDNA clone MGC:104291 IMAGE:4287636), complete cds.	RPS26	1,54
chitobiase, di-N-acetyl-	CTBS	1,54
ubiquitin specific peptidase 17-like family member 10	USP17L10	1,54
activated leukocyte cell adhesion molecule	ALCAM	1,54
TSC22 domain family, member 4	TSC22D4	1,54
MDS1 and EVI1 complex locus	MECOM	1,54
NADH dehydrogenase (ubiquinone) complex I, assembly factor 7	NDUFAF7	1,54
filamin C, gamma	FLNC	1,54
H2A histone family, member B2	H2AFB2	1,54
dynein, cytoplasmic 1, intermediate chain 2	DYNC1I2	1,54
zinc finger protein 468	ZNF468	1,54
microtubule associated protein 4	MAP4	1,54
protein tyrosine phosphatase, non-receptor type 3	PTPN3	1,54
apoptosis-inducing factor, mitochondrion-associated, 2	AIFM2	1,54
phosphotriesterase related	PTER	1,54
transmembrane protein 160	TMEM160	1,54
ancient ubiquitous protein 1	AUP1	1,54
SLAIN motif family member 2	SLAIN2	1,54
cadherin 2, type 1, N-cadherin (neuronal)	CDH2	1,54
programmed cell death 10	PDCD10	1,54

GABA(A) receptor-associated protein	GABARAP	1,54
striatin, calmodulin binding protein	STRN	1,54
SFI1 centrin binding protein	SFI1	1,54
TM2 domain containing 1	TM2D1	1,54
retention in endoplasmic reticulum sorting receptor 1	RER1	1,54
inositol polyphosphate-5-phosphatase K	INPP5K	1,54
keratin associated protein 12-2	KRTAP12-2	1,54
ubiquitin-conjugating enzyme E2D 4 (putative)	UBE2D4	1,54
carboxypeptidase A6	CPA6	1,54
methionine adenosyltransferase II, alpha	MAT2A	1,54
vezatin, adherens junctions transmembrane protein	VEZT	1,54
hes family bHLH transcription factor 1	HES1	1,54
myeloma overexpressed 2	MYEOV2	1,54
autophagy related 16-like 1	ATG16L1	1,54
LRRN4 C-terminal like	LRRN4CL	1,54
mitochondrial elongation factor 2	MIEF2	1,54
D4, zinc and double PHD fingers family 2	DPF2	1,54
syntaxin 2	STX2	1,54
cardiotrophin 1	CTF1	1,54
CDV3 homolog (mouse)	CDV3	1,54
Transcript Identified by AceView, Entrez Gene ID(s) 5747	PTK2	1,54
Memczak2013 ALT_ACCEPTOR, ALT_DONOR, coding, INTERNAL, intronic best transcript NM_017841	SDHAF2	1,54
VPS11, CORVET/HOPS core subunit [Source:HGNC Symbol;Acc:HGNC:14583]	VPS11	1,54
long intergenic non-protein coding RNA 656	LINC00656	1,54
VPS11, CORVET/HOPS core subunit [Source:HGNC Symbol;Acc:HGNC:14583]	VPS11	1,54
ubiquitin-like 7	UBL7	1,54
autophagy related 16-like 1	ATG16L1	1,53
zinc finger protein 84	ZNF84	1,53
chromosome 18 open reading frame 25	C18orf25	1,53
zinc finger protein 229	ZNF229	1,53
ribonuclease H1	RNASEH1	1,53
C-x(9)-C motif containing 4; mature T-cell proliferation 1	CMC4; MTCP1	1,53
phosphorylase kinase, alpha 1 (muscle)	PHKA1	1,53
zinc finger, CCHC domain containing 17	ZCCHC17	1,53
coiled-coil alpha-helical rod protein 1	CCHCR1	1,53
interferon gamma receptor 1	IFNGR1	1,53
Memczak2013 ANTISENSE, coding, INTERNAL, intronic best transcript NM_015995	KLF13	1,53
ankyrin repeat domain 50	ANKRD50	1,53
bromodomain adjacent to zinc finger domain 2B	BAZ2B	1,53

ataxin 1-like	ATXN1L	1,53
Memczak2013 ANTISENSE, CDS, coding, INTERNAL best transcript NM_002087	GRN	1,53
small cell adhesion glycoprotein	SMAGP	1,53
chromosome 9 open reading frame 78	C9orf78	1,53
zinc finger protein 280D	ZNF280D	1,53
BRO1 domain and CAAX motif containing	BROX	1,53
caspase 5	CASP5	1,53
Sp6 transcription factor	SP6	1,53
Fanconi anemia complementation group D2	FANCD2	1,53
late cornified envelope 1C	LCE1C	1,53
arylsulfatase D	ARSD	1,53
ATPase, H+ transporting, lysosomal 14kDa, V1 subunit F	ATP6V1F	1,53
TBC1 domain family, member 2	TBC1D2	1,53
tripartite motif containing 5	TRIM5	1,53
chromosome 8 open reading frame 22	C8orf22	1,53
Sp8 transcription factor	SP8	1,53
chromosome 19 open reading frame 53	C19orf53	1,53
transcription termination factor, RNA polymerase I	TTF1	1,53
chromosome 1 open reading frame 52	C1orf52	1,53
caveolin 2	CAV2	1,53
family with sequence similarity 86, member B2	FAM86B2	1,53
UBX domain protein 2B	UBXN2B	1,53
testis expressed 33	TEX33	1,53
CUGBP, Elav-like family member 3	CELF3	1,53
zinc finger protein 765	ZNF765	1,53
R3H domain and coiled-coil containing 1-like	R3HCC1L	1,53
basic leucine zipper and W2 domains 1	BZW1	1,53
mitogen-activated protein kinase kinase 4; microRNA 744	MAP2K4; MIR744	1,53
small integral membrane protein 5	SMIM5	1,53
phosphoinositide-3-kinase, regulatory subunit 6	PIK3R6	1,53
potassium channel modulatory factor 1	KCMF1	1,53
dynactin 2 (p50)	DCTN2	1,53
zinc finger, SWIM-type containing 8	ZSWIM8	1,52
transmembrane protein 126B	TMEM126B	1,52
interferon regulatory factor 5	IRF5	1,52
zinc finger protein 221	ZNF221	1,52
proteasome 26S subunit, ATPase 4 [Source:HGNC Symbol;Acc:HGNC:9551]	PSMC4	1,52
sodium channel modifier 1; tumor necrosis factor, alpha-induced protein 8-like 2	SCNM1; TNFAIP8L2	1,52
golgin A8 family, member B	GOLGA8B	1,52
coiled-coil domain containing 173	CCDC173	1,52
SMAD family member 7	SMAD7	1,52

insulin-like growth factor 2 receptor	IGF2R	1,52
ring finger protein 10	RNF10	1,52
NADPH dependent diflavin oxidoreductase 1	NDOR1	1,52
zinc finger protein 100	ZNF100	1,52
Spi-1 proto-oncogene	SPI1	1,52
eukaryotic translation initiation factor 3, subunit I	EIF3I	1,52
solute carrier family 35 (GDP-fucose transporter), member C2	SLC35C2	1,52
membrane protein, palmitoylated 6	MPP6	1,52
kallikrein related peptidase 15	KLK15	1,52
family with sequence similarity 205, member C	FAM205C	1,52
leucine rich repeat neuronal 1	LRRN1	1,52
ATPase, H+ transporting, lysosomal 31kDa, V1 subunit E2	ATP6V1E2	1,52
golgin A6 family-like 3	GOLGA6L3	1,52
zinc finger protein 28	ZNF28	1,52
exostosin-like glycosyltransferase 3	EXTL3	1,52
DENN/MADD domain containing 1C	DENND1C	1,52
peptidylprolyl isomerase (cyclophilin)-like 4	PPIL4	1,52
ring finger and WD repeat domain 3	RFWD3	1,52
leucine carboxyl methyltransferase 1	LCMT1	1,52
family with sequence similarity 118, member B	FAM118B	1,52
WW domain binding protein 1-like	WBP1L	1,52
cut-like homeobox 1	CUX1	1,52
eukaryotic translation initiation factor 5B	EIF5B	1,52
chromosome 19 open reading frame 84	C19orf84	1,52
chromosome 5 open reading frame 38	C5orf38	1,52
MAP3K7 C-terminal like	MAP3K7CL	1,52
FGFR1OP N-terminal like	FOPNL	1,52
neutral cholesterol ester hydrolase 1	NCEH1	1,51
nuclear transport factor 2	NUTF2	1,51
globoside alpha-1,3-N-acetylgalactosaminyltransferase 1	GBGT1	1,51
spire-type actin nucleation factor 1	SPIRE1	1,51
cancer susceptibility candidate 3; microRNA 6866	CASC3; MIR6866	1,51
harakiri, BCL2 interacting protein	HRK	1,51
transmembrane protein 106B	TMEM106B	1,51
midnolin	MIDN	1,51
YdjC homolog (bacterial)	YDJC	1,51
KLRC4-KLRK1 readthrough; killer cell lectin-like receptor subfamily K, member 1; killer cell lectin-like receptor subfamily C, member 4	KLRC4-KLRK1; KLRK1; KLRC4	1,51
sphingosine-1-phosphate phosphatase 1	SGPP1	1,51
transaldolase 1	TALDO1	1,51
lysine (K)-specific demethylase 6B	KDM6B	1,51

S100 calcium binding protein A13	S100A13	1,51
NIPA-like domain containing 3	NIPAL3	1,51
chemokine (C-X-C motif) ligand 1 (melanoma growth stimulating activity, alpha)	CXCL1	1,51
ubiquitin associated protein 2; small nucleolar RNA, C/D box 121A; small nucleolar RNA, C/D box 121B	UBAP2; SNORD121A; SNORD121B	1,51
transmembrane and tetratricopeptide repeat containing 1	TMTC1	1,51
WD repeat domain 73	WDR73	1,51
jade family PHD finger 2	JADE2	1,51
Salzman2013 ALT_ACCEPTOR, ALT_DONOR, coding, INTERNAL, intronic best transcript NM_133474	ZNF721	1,51
RAB, member of RAS oncogene family-like 3	RABL3	1,51
PRELI domain containing 3B; ATP synthase, H+ transporting, mitochondrial F1 complex, epsilon subunit	PRELID3B; ATP5E	1,51
radial spoke 3 homolog (Chlamydomonas)	RSPH3	1,51
pleckstrin homology domain containing, family G (with RhoGef domain) member 2	PLEKHG2	1,51
chromodomain protein, Y-like 2	CDYL2	1,51
BRCA1 associated protein	BRAP	1,51
host cell factor C1 regulator 1 (XPO1 dependent)	HCFC1R1	1,51
zinc finger protein 337	ZNF337	1,51
A kinase (PRKA) anchor protein 2	AKAP2	1,51
tryptophanyl-tRNA synthetase	WARS	1,51
HAUS augmin like complex subunit 6	HAUS6	1,51
intersectin 1	ITSN1	1,51
intraflagellar transport 46	IFT46	1,51
solute carrier family 25 (mitochondrial iron transporter), member 37	SLC25A37	1,51
family with sequence similarity 160, member A2	FAM160A2	1,51
HECT and RLD domain containing E3 ubiquitin protein ligase 4	HERC4	1,51
membrane associated ring finger 3	MARCH3	1,51
K(lysine) acetyltransferase 7	KAT7	1,51
TNF receptor-associated factor 3	TRAF3	1,51
galactose-1-phosphate uridylyltransferase	GALT	1,51
family with sequence similarity 192, member A	FAM192A	1,50
chromosome 20 open reading frame 194	C20orf194	1,50
syndecan binding protein (syntenin) 2	SDCBP2	1,50
component of oligomeric golgi complex 1	COG1	1,50
zinc finger protein 556	ZNF556	1,50
Janus kinase 1	JAK1	1,50
glucose-fructose oxidoreductase domain containing 2	GFOD2	1,50
leucine-rich, glioma inactivated 1	LGI1	1,50

olfactory receptor, family 52, subfamily K, member 2	OR52K2	1,50
PIH1 domain containing 2	PIH1D2	1,50
dolichol kinase	DOLK	1,50
MIF4G domain containing	MIF4GD	1,50
G elongation factor, mitochondrial 1	GFM1	1,50
BCL2-antagonist/killer 1	BAK1	1,50
LIM domain binding 1	LDB1	1,50
TSSK6 activating co-chaperone	TSACC	1,50
transmembrane protein 184B	TMEM184B	1,50
adrenomedullin 2	ADM2	1,50
chromosome 6 open reading frame 89	C6orf89	1,50
small glutamine-rich tetratricopeptide repeat (TPR)-containing, alpha	SGTA	1,50
chromosome 14 open reading frame 79	C14orf79	1,50
vacuolar protein sorting 41 homolog (S. cerevisiae)	VPS41	1,50
immunoglobulin superfamily, member 9B	IGSF9B	1,50
family with sequence similarity 231, member D; long intergenic non-protein coding RNA 869	FAM231D; LINC00869	1,50
RAB7A, member RAS oncogene family	RAB7A	1,50
argininosuccinate lyase	ASL	1,50
mitogen-activated protein kinase-activated protein kinase 2	MAPKAPK2	1,50
signal peptide, CUB domain, EGF-like 2	SCUBE2	1,50
G protein-coupled receptor 83	GPR83	1,50
vesicle transport through interaction with t-SNAREs 1A	VTI1A	1,50
VIM antisense RNA 1	VIM-AS1	1,50
DEAD (Asp-Glu-Ala-Asp) box polypeptide 27	DDX27	1,50
heat shock protein family B (small), member 11	HSPB11	1,50
zinc finger protein 518B	ZNF518B	1,50
partner of NOB1 homolog	PNO1	1,50
long intergenic non-protein coding RNA 1537	LINC01537	1,50
Smad nuclear interacting protein 1	SNIP1	1,50
complement component 1, q subcomponent, B chain	C1QB	1,50
breast carcinoma amplified sequence 2	BCAS2	1,50
sterile alpha motif domain containing 8	SAMD8	1,50
fidgetin	FIGN	1,49
RAS-like, estrogen-regulated, growth inhibitor	RERG	1,49
kelch-like family member 2	KLHL2	1,49
chromosome 5 open reading frame 63	C5orf63	1,49
zinc finger, DHHC-type containing 7	ZDHHC7	1,49
pleckstrin homology domain containing, family N member 1	PLEKHN1	1,49
coiled-coil domain containing 74A	CCDC74A	1,49
RAS-like, estrogen-regulated, growth inhibitor	RERG	1,49
major histocompatibility complex, class II, DM alpha	HLA-DMA	1,49

RAP1, GTP-GDP dissociation stimulator 1	RAP1GDS1	1,49
leucine-rich repeat containing G protein-coupled receptor 6	LGR6	1,49
proteasome subunit beta 7	PSMB7	1,49
acyl-CoA wax alcohol acyltransferase 1	AWAT1	1,49
proteasome 26S subunit, non-ATPase 8	PSMD8	1,49
EYA transcriptional coactivator and phosphatase 3	EYA3	1,49
Rab geranylgeranyltransferase, alpha subunit	RABGGTA	1,49
zinc finger protein 555	ZNF555	1,49
zinc finger protein 717	ZNF717	1,49
centrosomal protein 57kDa-like 1	CEP57L1	1,49
small nuclear ribonucleoprotein polypeptide C	SNRPC	1,49
zinc finger, AN1-type domain 3	ZFAND3	1,49
bolA family member 3	BOLA3	1,49
mitogen-activated protein kinase kinase 1	MAP2K1	1,49
solute carrier family 43 (amino acid system L transporter), member 2	SLC43A2	1,49
interferon induced transmembrane protein 2	IFITM2	1,49
exocyst complex component 5	EXOC5	1,49
calponin 2	CNN2	1,49
ubiquitin-conjugating enzyme E2M	UBE2M	1,49
proteasome 26S subunit, ATPase 4 [Source:HGNC Symbol;Acc:HGNC:9551]	PSMC4	1,49
eukaryotic translation initiation factor 2-alpha kinase 2	EIF2AK2	1,49
FSHD region gene 2 family, member B	FRG2B	1,49
lipoma HMGIC fusion partner-like 5	LHFPL5	1,49
RNA binding motif protein 22	RBM22	1,49
autophagy related 16-like 1	ATG16L1	1,49
hyaluronan and proteoglycan link protein 1	HAPLN1	1,49
secretory leukocyte peptidase inhibitor	SLPI	1,49
glutamate receptor, ionotropic, AMPA 2	GRIA2	1,49
kinesin family member 3B	KIF3B	1,49
TAF4b RNA polymerase II, TATA box binding protein (TBP)-associated factor, 105kDa	TAF4B	1,49
deoxyribose-phosphate aldolase (putative)	DERA	1,49
threonine synthase-like 1	THNSL1	1,49
NOTCH-regulated ankyrin repeat protein	NRARP	1,49
family with sequence similarity 49, member B	FAM49B	1,48
Salzman2013 ANTISENSE, coding, INTERNAL, intronic, OVERLAPTX best transcript NM_005603; novel transcript, antisense to ATP8B1	RP11-35G9.5; ATP8B1	1,48
coiled-coil domain containing 144 family, N-terminal like	CCDC144NL	1,48
transcription elongation factor B polypeptide 3C-like	TCEB3CL	1,48
transmembrane protein 233	TMEM233	1,48

SDA1 domain containing 1	SDAD1	1,48
small nuclear ribonucleoprotein, U11/U12 35kDa subunit	SNRNP35	1,48
BEN domain containing 5	BEND5	1,48
staufen double-stranded RNA binding protein 2	STAU2	1,48
coiled-coil domain containing 51	CCDC51	1,48
myelin protein zero-like 3	MPZL3	1,48
protein phosphatase 2, regulatory subunit A, alpha	PPP2R1A	1,48
RAB18, member RAS oncogene family	RAB18	1,48
acylphosphatase 1, erythrocyte (common) type	ACYP1	1,48
debranching RNA lariats 1	DBR1	1,48
zinc finger protein 697	ZNF697	1,48
fibronectin type III domain containing 3A	FNDC3A	1,48
RAB30, member RAS oncogene family	RAB30	1,48
polymerase (DNA-directed), delta 3, accessory subunit	POLD3	1,48
NECAP endocytosis associated 2	NECAP2	1,48
tumor protein D52	TPD52	1,48
transmembrane protein 30A	TMEM30A	1,48
vesicle associated membrane protein 7	VAMP7	1,48
queuine tRNA-ribosyltransferase domain containing 1	QTRTD1	1,48
Transcript Identified by AceView, Entrez Gene ID(s) 26269	FBXO8	1,48
Kruppel-like factor 12	KLF12	1,48
toll-like receptor 3	TLR3	1,48
meiosis/spermiogenesis associated 1	MEIG1	1,48
novel transcript; Transcript Identified by AceView, Entrez Gene ID(s) 54453	RP1-122P22.2; RIN2	1,48
zinc finger protein 415	ZNF415	1,48
zinc finger protein 529	ZNF529	1,48
Transcript Identified by AceView, Entrez Gene ID(s) 238	ALK	1,48
F-box and leucine-rich repeat protein 18	FBXL18	1,48
Memczak2013 ANTISENSE, coding, INTERNAL, intronic best transcript NM_001165136	HERC6	1,48
N-terminal Xaa-Pro-Lys N-methyltransferase 1	NTMT1	1,48
nucleoporin 62kDa; interleukin 4 induced 1	NUP62; IL4I1	1,48
sterile alpha motif domain containing 15	SAMD15	1,48
keratin 6B, type II	KRT6B	1,48
cytochrome c oxidase subunit VIc	COX6C	1,48
family with sequence similarity 157, member B	FAM157B	1,48
WD repeat domain 1	WDR1	1,47
centrosomal protein 170kDa	CEP170	1,47
WW domain binding protein 2	WBP2	1,47
general transcription factor IIE subunit 1	GTF2E1	1,47
MAGE family member A2; MAGE family member A2B	MAGEA2; MAGEA2B	1,47

chromosome 17 open reading frame 62	C17orf62	1,47
DENN/MADD domain containing 2A	DENND2A	1,47
centrosomal protein 170B	CEP170B	1,47
potassium channel tetramerization domain containing 10	KCTD10	1,47
actin binding LIM protein 1	ABLIM1	1,47
motile sperm domain containing 2	MOSPD2	1,47
GATA zinc finger domain containing 2B	GATAD2B	1,47
SDE2 telomere maintenance homolog (S. pombe)	SDE2	1,47
2-5-oligoadenylate synthetase 2	OAS2	1,47
N-myristoyltransferase 1	NMT1	1,47
formin binding protein 1	FNBP1	1,47
SET binding factor 2	SBF2	1,47
WD repeat domain 55	WDR55	1,47
5-aminolevulinate synthase 2	ALAS2	1,47
SH3 domain binding glutamate-rich protein	SH3BGR	1,47
zinc finger protein 736	ZNF736	1,47
legumain	LGMN	1,47
regulatory factor X, 2 (influences HLA class II expression)	RFX2	1,47
GATS protein-like 3	GATSL3	1,47
sphingomyelin phosphodiesterase 1, acid lysosomal	SMPD1	1,47
TAF7 RNA polymerase II, TATA box binding protein (TBP)-associated factor, 55kDa	TAF7	1,47
elongation factor Tu GTP binding domain containing 2	EFTUD2	1,47
B double prime 1, subunit of RNA polymerase III transcription initiation factor IIIB	BDP1	1,47
Yip1 domain family member 1	YIPF1	1,47
chromosome 17 open reading frame 78	C17orf78	1,47
zinc finger protein 296	ZNF296	1,47
glutathione S-transferase mu 3 (brain)	GSTM3	1,47
U2 small nuclear RNA auxiliary factor 1-like 4	U2AF1L4	1,47
coiled-coil domain containing 50	CCDC50	1,47
hypoxia inducible factor 3, alpha subunit	HIF3A	1,47
POU class 2 homeobox 2	POU2F2	1,47
zinc finger protein 281	ZNF281	1,47
molybdenum cofactor synthesis 3	MOCS3	1,47
TNFAIP3 interacting protein 2	TNIP2	1,47
zinc finger protein 829	ZNF829	1,47
protocadherin 17	PCDH17	1,47
stimulated by retinoic acid 6	STRA6	1,47
histone cluster 1, H2ac	HIST1H2AC	1,47
proteasome 26S subunit, non-ATPase 12	PSMD12	1,47
neuropilin 2	NRP2	1,47

fibroblast growth factor 13; long intergenic non-protein coding RNA 889	FGF13; LINC00889	1,47
ubiquitin conjugating enzyme E2E 2	UBE2E2	1,47
polyhomeotic homolog 3 (Drosophila)	PHC3	1,47
thioredoxin domain containing 9	TXNDC9	1,47
zinc finger protein 134	ZNF134	1,47
CDC like kinase 3	CLK3	1,47
synovial sarcoma, X breakpoint 5	SSX5	1,47
inscuteable homolog (Drosophila)	INSC	1,46
G protein-coupled receptor 151	GPR151	1,46
keratin associated protein 4-12	KRTAP4-12	1,46
WWC family member 3	WWC3	1,46
epidermal growth factor receptor pathway substrate 15	EPS15	1,46
arylsulfatase A	ARSA	1,46
family with sequence similarity 32, member A	FAM32A	1,46
dynactin 5 (p25)	DCTN5	1,46
GRIP and coiled-coil domain containing 1	GCC1	1,46
keratin 17, type I	KRT17	1,46
STAM binding protein	STAMBP	1,46
kinesin family member C3	KIFC3	1,46
ATP synthase, H+ transporting, mitochondrial F1 complex, epsilon subunit pseudogene 2	ATP5EP2	1,46
exonuclease 1	EXO1	1,46
ALG6, alpha-1,3-glucosyltransferase	ALG6	1,46
cell division cycle 42	CDC42	1,46
midkine (neurite growth-promoting factor 2)	MDK	1,46
splicing factor 3a, subunit 1, 120kDa	SF3A1	1,46
zinc finger, CCHC domain containing 7	ZCCHC7	1,46
interleukin 17 receptor C	IL17RC	1,46
lysyl oxidase-like 4	LOXL4	1,46
NAD(P)H dehydrogenase, quinone 1	NQO1	1,46
serine/arginine-rich splicing factor 12	SRSF12	1,46
chitinase domain containing 1	CHID1	1,46
dolichyl-phosphate (UDP-N-acetylglucosamine) N-acetylglucosaminephosphotransferase 1 (GlcNAc-1-P transferase)	DPAGT1	1,46
cytochrome b561 family, member A3	CYB561A3	1,46
snail family zinc finger 2	SNAI2	1,46
adipogenesis associated, Mth938 domain containing	AAMDC	1,46
leucine rich repeat containing 6	LRRC6	1,46
glucosaminyl (N-acetyl) transferase 2, I-branching enzyme (I blood group)	GCNT2	1,46
CD300a molecule	CD300A	1,46
nucleotide-binding oligomerization domain containing 1	NOD1	1,46
cholinergic receptor, nicotinic alpha 7	CHRNA7	1,46

DEAD (Asp-Glu-Ala-Asp) box polypeptide 10	DDX10	1,46
transmembrane protein 39B	TMEM39B	1,46
speedy/RINGO cell cycle regulator family member C	SPDYC	1,46
coiled-coil domain containing 88A	CCDC88A	1,46
ubiquitin specific peptidase 25	USP25	1,46
vestigial-like family member 4	VGLL4	1,46
LIM domain 7	LMO7	1,46
cell division cycle 45	CDC45	1,46
zinc finger protein 90	ZNF90	1,46
salvador family WW domain containing protein 1	SAV1	1,46
polypeptide N-acetylgalactosaminyltransferase 7	GALNT7	1,46
nuclear RNA export factor 3	NXF3	1,46
charged multivesicular body protein 1A	CHMP1A	1,46
proteasome inhibitor subunit 1	PSMF1	1,46
proline rich 16	PRR16	1,46
NSFL1 (p97) cofactor (p47)	NSFL1C	1,46
cilia and flagella associated protein 61	CFAP61	1,46
Transcript Identified by AceView, Entrez Gene ID(s) 134145	FAM173B	1,46
transmembrane protein 179B; microRNA 6748	TMEM179B; MIR6748	1,46
TBC1 domain family, member 10A	TBC1D10A	1,46
surfeit 2	SURF2	1,46
ring finger protein 44	RNF44	1,46
INO80 complex subunit C	INO80C	1,46
zinc finger protein 570	ZNF570	1,46
F-box protein 45	FBXO45	1,46
asparagine synthetase (glutamine-hydrolyzing)	ASNS	1,46
ganglioside induced differentiation associated protein 1-like 1	GDAP1L1	1,46
trafficking protein particle complex 2	TRAPPC2	1,46
keratin 76, type II	KRT76	1,46
KxDL motif containing 1	KXD1	1,46
RAD23 homolog B, nucleotide excision repair protein	RAD23B	1,46
polymerase (RNA) I polypeptide C	POLR1C	1,45
paraneoplastic Ma antigen family-like 2	PNMAL2	1,45
VPS11, CORVET/HOPS core subunit [Source:HGNC Symbol;Acc:HGNC:14583]	VPS11	1,45
tropomyosin 2 (beta)	TPM2	1,45
adenosine A2a receptor	ADORA2A	1,45
family with sequence similarity 210, member A	FAM210A	1,45
vasodilator-stimulated phosphoprotein	VASP	1,45
HMG box domain containing 3	HMGXB3	1,45
uncharacterized LOC100130370; novel transcript	LOC100130370; RP11-1055B8.3	1,45

family with sequence similarity 153, member A	FAM153A	1,45
ZMYM6 neighbor; zinc finger, MYM-type 6	ZMYM6NB; ZMYM6	1,45
sodium channel, voltage gated, type I beta subunit	SCN1B	1,45
ring finger protein 216	RNF216	1,45
A kinase (PRKA) interacting protein 1	AKIP1	1,45
coiled-coil domain containing 130	CCDC130	1,45
zinc finger protein 569	ZNF569	1,45
family with sequence similarity 177, member A1	FAM177A1	1,45
transmembrane phosphoinositide 3-phosphatase and tensin homolog 2	TPTE2	1,45
mitochondrial ribosomal protein L52	MRPL52	1,45
fatty acid binding protein 5 (psoriasis-associated)	FABP5	1,45
recombination signal binding protein for immunoglobulin kappa J region-like	RBPJL	1,45
dynamin 1	DNM1	1,45
dynactin 6	DCTN6	1,45
programmed cell death 11	PDCD11	1,45
GATS protein-like 2	GATSL2	1,45
ubiquitin specific peptidase 17-like family member 19	USP17L19	1,45
nucleolar protein 8	NOL8	1,45
sparc/osteonectin, cwcv and kazal-like domains proteoglycan (testican) 2	SPOCK2	1,45
SUN domain containing ossification factor	SUCO	1,45
Memczak2013 ALT_ACCEPTOR, ALT_DONOR, coding, INTERNAL, intronic best transcript NM_021937	EEFSEC	1,45
Rho GTPase activating protein 9	ARHGAP9	1,45
GLIS family zinc finger 3	GLIS3	1,45
G protein-coupled receptor kinase interacting ArfGAP 2	GIT2	1,45
chromosome 9 open reading frame 16	C9orf16	1,45
coiled-coil domain containing 64	CCDC64	1,45
zinc finger protein 490	ZNF490	1,45
RAB39A, member RAS oncogene family	RAB39A	1,45
chemokine (C-C motif) ligand 23	CCL23	1,45
family with sequence similarity 214, member A	FAM214A	1,45
Memczak2013 ALT_ACCEPTOR, ALT_DONOR, coding, INTERNAL, intronic best transcript NM_172373	ELF1	1,45
serine hydrolase-like 2	SERHL2	1,45
Memczak2013 ANTISENSE, CDS, coding, INTERNAL best transcript NM_001174167	SYK	1,45
tubulin folding cofactor E	TBCE	1,45
ADAM metallopeptidase domain 28	ADAM28	1,45
aldehyde dehydrogenase 1 family, member L2	ALDH1L2	1,45
signal-induced proliferation-associated 1	SIPA1	1,45
protein kinase, cGMP-dependent, type I	PRKG1	1,45

adhesion G protein-coupled receptor D1	ADGRD1	1,45
glucosamine (N-acetyl)-6-sulfatase	GNS	1,45
solute carrier family 16, member 12	SLC16A12	1,45
uncharacterized LOC102723701; novel transcript, antisense to ERLIN2	LOC102723701; RP11-863K10.7	1,45
casein kinase 1, alpha 1	CSNK1A1	1,45
KIAA1671	KIAA1671	1,45
neurochondrin	NCDN	1,45
xin actin binding repeat containing 2	XIRP2	1,45
cell division cycle 37-like 1	CDC37L1	1,45
glucan (1,4-alpha-), branching enzyme 1	GBE1	1,45
olfactory receptor, family 4, subfamily C, member 46	OR4C46	1,45
regulator of telomere elongation helicase 1; tumor necrosis factor receptor superfamily, member 6b, decoy; RTEL1-TNFRSF6B readthrough (NMD candidate)	RTEL1; TNFRSF6B; RTEL1-TNFRSF6B	1,45
ELL associated factor 1	EAF1	1,45
tubulin polyglutamylase complex subunit 2	TPGS2	1,45
transmembrane protein 79	TMEM79	1,45
MINOS1-NBL1 readthrough	MINOS1-NBL1	1,45
prosaposin	PSAP	1,45
zinc finger, DHHC-type containing 3	ZDHHC3	1,44
elongation factor RNA polymerase II	ELL	1,44
islet cell autoantigen 1 like	ICA1L	1,44
X-prolyl aminopeptidase (aminopeptidase P) 1, soluble	XPNPEP1	1,44
long intergenic non-protein coding RNA 518	LINC00518	1,44
histone deacetylase 9	HDAC9	1,44
keratin 33B, type I	KRT33B	1,44
GIPC PDZ domain containing family, member 1	GIPC1	1,44
sperm specific antigen 2	SSFA2	1,44
tRNA-yW synthesizing protein 3 homolog (S. cerevisiae)	TYW3	1,44
family with sequence similarity 21, member C	FAM21C	1,44
germ cell associated 2 (haspin)	GSG2	1,44
selectin P ligand	SELPLG	1,44
kelch-like family member 5	KLHL5	1,44
myosin IC	MYO1C	1,44
kelch repeat and BTB (POZ) domain containing 8	KBTBD8	1,44
MLX interacting protein	MLXIP	1,44
chromosome 11 open reading frame 68	C11orf68	1,44
eukaryotic translation initiation factor 5A	EIF5A	1,44
polymerase (RNA) II (DNA directed) polypeptide K, 7.0kDa	POLR2K	1,44
nanos homolog 2 (Drosophila)	NANOS2	1,44
cleavage and polyadenylation factor I subunit 1	CLP1	1,44
chromosome 8 open reading frame 46	C8orf46	1,44
farnesyltransferase, CAAX box, alpha	FNTA	1,44

glucosidase, beta, acid 3 (gene/pseudogene)	GBA3	1,44
tec protein tyrosine kinase	TEC	1,44
zinc finger protein 266	ZNF266	1,44
diaphanous-related formin 3	DIAPH3	1,44
CD81 molecule	CD81	1,44
BCL2-like 12 (proline rich)	BCL2L12	1,44
C15orf38-AP3S2 readthrough	C15orf38-AP3S2	1,44
bone morphogenetic protein/retinoic acid inducible neural-specific 1	BRINP1	1,44
alkB homolog 8, tRNA methyltransferase	ALKBH8	1,44
SUZ RNA binding domain containing 1	SZRD1	1,44
tropomodulin 3 (ubiquitous)	TMOD3	1,44
olfactory receptor, family 10, subfamily A, member 3	OR10A3	1,44
MUS81 structure-specific endonuclease subunit	MUS81	1,44
additional sex combs like transcriptional regulator 2	ASXL2	1,44
tyrosine 3-monooxygenase/tryptophan 5-monooxygenase activation protein, beta	YWHAB	1,44
chromosome 6 open reading frame 10	C6orf10	1,44
integrator complex subunit 1	INTS1	1,44
cysteine-serine-rich nuclear protein 2	CSRNP2	1,44
autophagy related 16-like 1	ATG16L1	1,44
zinc finger E-box binding homeobox 1	ZEB1	1,44
defensin, beta 116	DEFB116	1,44
like-glycosyltransferase	LARGE	1,44
ZNF582 antisense RNA 1 (head to head)	ZNF582-AS1	1,44
olfactory receptor, family 2, subfamily B, member 6	OR2B6	1,44
UBAP1-MVB12-associated (UMA) domain containing 1	UMAD1	1,44
placenta specific 1	PLAC1	1,44
C-type lectin domain family 9, member A	CLEC9A	1,44
collagen, type XXI, alpha 1	COL21A1	1,44
coiled-coil domain containing 79	CCDC79	1,44
mucin 19, oligomeric	MUC19	1,44
DAZ interacting zinc finger protein 1-like	DZIP1L	1,44
POTE ankyrin domain family, member B	POTEB	1,44
chloride channel CLIC-like 1	CLCC1	1,44
Ran GTPase activating protein 1	RANGAP1	1,44
RAB guanine nucleotide exchange factor (GEF) 1	RABGEF1	1,44
cysteine and glycine-rich protein 3 (cardiac LIM protein)	CSRP3	1,44
nucleoporin 153kDa	NUP153	1,44
snurportin 1	SNUPN	1,44
mitochondrial ribosomal protein L9	MRPL9	1,44
cordon-bleu WH2 repeat protein	COBL	1,44
cation channel, sperm associated 1	CATSPER1	1,44
platelet-activating factor acetylhydrolase 1b, regulatory subunit 1 (45kDa)	PAFAH1B1	1,44

LIM homeobox 6	LHX6	1,44
PDZ domain containing 9	PDZD9	1,44
cyclin-dependent kinase inhibitor 2B (p15, inhibits CDK4)	CDKN2B	1,43
transmembrane protein 154	TMEM154	1,43
centromere protein H	CENPH	1,43
transmembrane protein 31	TMEM31	1,43
TELO2 interacting protein 1	TTI1	1,43
zona pellucida glycoprotein 3 (sperm receptor)	ZP3	1,43
spindlin family, member 2B	SPIN2B	1,43
interphotoreceptor matrix proteoglycan 1	IMPG1	1,43
suppression of tumorigenicity 7 like	ST7L	1,43
CUE domain containing 1	CUEDC1	1,43
cytochrome P450, family 39, subfamily A, polypeptide 1	CYP39A1	1,43
Memczak2013 ANTISENSE, CDS, coding, INTERNAL best transcript NM_001130967	ZNF385A	1,43
cerebral endothelial cell adhesion molecule	CERCAM	1,43
sarcolemma associated protein	SLMAP	1,43
related RAS viral (r-ras) oncogene homolog 2	RRAS2	1,43
myosin IXB	MYO9B	1,43
modulator of apoptosis 1	MOAP1	1,43
RuvB-like AAA ATPase 2	RUVBL2	1,43
transmembrane protein 54	TMEM54	1,43
nuclear receptor subfamily 2, group F, member 2	NR2F2	1,43
tripartite motif containing 34	TRIM34	1,43
glucocorticoid modulatory element binding protein 1	GMEB1	1,43
meiosis specific with OB domains; long intergenic non-protein coding RNA 254	MEIOB; LINC00254	1,43
ADAM metallopeptidase with thrombospondin type 1 motif 17	ADAMTS17	1,43
C-type lectin domain family 4, member E	CLEC4E	1,43
casein kinase 1, epsilon	CSNK1E	1,43
RAD9 checkpoint clamp component A	RAD9A	1,43
signal transducing adaptor molecule (SH3 domain and ITAM motif) 2	STAM2	1,43
dual specificity phosphatase 28	DUSP28	1,43
gamma-glutamyltransferase 1	GGT1	1,43
patatin-like phospholipase domain containing 1	PNPLA1	1,43
long intergenic non-protein coding RNA 1296; double homeobox A pseudogene 10	LINC01296; DUXAP10	1,43
B and T lymphocyte associated	BTLA	1,43

Homo sapiens vesicle-associated membrane protein 7 (VAMP7), transcript variant 2, mRNA.; Homo sapiens vesicle-associated membrane protein 7 (VAMP7), transcript variant 3, mRNA.; Homo sapiens vesicle-associated membrane protein 7 (VAMP7), transcript variant 1, mRNA.; Homo sapiens vesicle-associated membrane protein 7 (VAMP7), transcript variant 4, non-coding RNA.; Homo sapiens vesicle-associated membrane protein 7 (VAMP7), transcript variant 5, non-coding RNA.; vesicle-associated membrane protein 7 [Source:HGNC Symbol;Acc:HGNC:11486]; Homo sapiens vesicle-associated membrane protein 7, mRNA (cDNA clone MGC:64832 IMAGE:6503665), complete cds.	VAMP7	1,43
laeverin	LVRN	1,43
centrosomal protein 72kDa	CEP72	1,43
G protein-coupled receptor 132	GPR132	1,43
A kinase (PRKA) anchor protein 8	AKAP8	1,43
methylenetetrahydrofolate dehydrogenase (NADP+ dependent) 1-like	MTHFD1L	1,43
THAP domain containing 8	THAP8	1,43
ecdysoneless homolog (Drosophila)	ECD	1,43
zinc finger, GRF-type containing 1	ZGRF1	1,43
family with sequence similarity 73, member A	FAM73A	1,43
zinc finger and BTB domain containing 8A	ZBTB8A	1,43
major facilitator superfamily domain containing 14B	MFSD14B	1,43
zinc finger protein 354A	ZNF354A	1,43
retinitis pigmentosa GTPase regulator	RPGR	1,43
family with sequence similarity 167, member B	FAM167B	1,43
formin 1	FMN1	1,43
casein kinase 1, epsilon	CSNK1E	1,43
single-minded family bHLH transcription factor 2	SIM2	1,43
coiled-coil domain containing 13	CCDC13	1,43
zinc finger protein 710	ZNF710	1,43
uveal autoantigen with coiled-coil domains and ankyrin repeats	UACA	1,43
late cornified envelope 1E	LCE1E	1,43
malignant fibrous histiocytoma amplified sequence 1	MFHAS1	1,43
actinin, alpha 1; high mobility group nucleosome binding domain 1 pseudogene 3	ACTN1; HMGN1P3	1,43
DMRT-like family A1	DMRTA1	1,43
sodium channel, voltage gated, type IV alpha subunit	SCN4A	1,42
cytoplasmic polyadenylation element binding protein 1	CPEB1	1,42
fibulin 5	FBLN5	1,42
HNRNPUL2-BSCL2 readthrough (NMD candidate)	HNRNPUL2-BSCL2	1,42

Memczak2013 ANTISENSE, coding, INTERNAL, intronic best transcript NM_139057	ADAMTS17	1,42
muscleblind-like splicing regulator 2	MBNL2	1,42
chromosome 15 open reading frame 52	C15orf52	1,42
U6 snRNA biogenesis 1	USB1	1,42
ripply transcriptional repressor 3	RIPPLY3	1,42
G protein-coupled receptor 137	GPR137	1,42
FES proto-oncogene, tyrosine kinase	FES	1,42
translin	TSN	1,42
HERV-H LTR-associating 3	HHLA3	1,42
ubiquitin C	UBC	1,42
protein kinase, membrane associated tyrosine/threonine 1	PKMYT1	1,42
peptidylprolyl isomerase D	PPID	1,42
leucine rich repeat and sterile alpha motif containing 1	LRSAM1	1,42
chaperonin containing TCP1, subunit 6A (zeta 1)	CCT6A	1,42
hemoglobin, alpha 1	HBA1	1,42
chemokine (C motif) receptor 1	XCR1	1,42
casein kinase 1, gamma 3	CSNK1G3	1,42
POP1 homolog, ribonuclease P/MRP subunit	POP1	1,42
NFKB inhibitor interacting Ras-like 1	NKIRAS1	1,42
hemoglobin, alpha 2	HBA2	1,42
hes family bHLH transcription factor 5	HES5	1,42
dystrophia myotonica, WD repeat containing	DMWD	1,42
SID1 transmembrane family, member 1	SIDT1	1,42
testis expressed 38	TEX38	1,42
integrin beta 3	ITGB3	1,42
collagen, type IV, alpha 3 (Goodpasture antigen) binding protein	COL4A3BP	1,42
cerebellin 3 precursor	CBLN3	1,42
paired-like homeodomain 3	PITX3	1,42
synaptic Ras GTPase activating protein 1; microRNA 5004	SYNGAP1; MIR5004	1,42
tuberous sclerosis 2	TSC2	1,42
carboxylesterase 4A	CES4A	1,42
deoxynucleotidyltransferase, terminal, interacting protein 2	DNTTIP2	1,42
zinc finger protein 583	ZNF583	1,42
zinc finger protein 761; tropomyosin 3 pseudogene 9	ZNF761; TPM3P9	1,42
zinc finger protein 738	ZNF738	1,42
fragile histidine triad	FHIT	1,42
caspase recruitment domain family, member 8	CARD8	1,42
immunoglobulin lambda-like polypeptide 1	IGLL1	1,42
acyl-CoA dehydrogenase family, member 11	ACAD11	1,42
general transcription factor IIH subunit 3	GTF2H3	1,42

long intergenic non-protein coding RNA 483	LINC00483	1,42
ubiquilin 1	UBQLN1	1,42
WD repeat domain 7	WDR7	1,42
lines homolog 1	LINS1	1,42
BUD31 homolog	BUD31	1,42
calmodulin binding transcription activator 2	CAMTA2	1,42
spermatogenesis associated 13; C1q and tumor necrosis factor related protein 9	SPATA13; C1QTNF9	1,42
heat shock 70kDa protein 8; small nucleolar RNA, C/D box 14C; small nucleolar RNA, C/D box 14D	HSPA8; SNORD14C; SNORD14D	1,41
ariadne RBR E3 ubiquitin protein ligase 1; microRNA 630	ARIH1; MIR630	1,41
LDL receptor related protein 2	LRP2	1,41
NFKB inhibitor interacting Ras-like 2	NKIRAS2	1,41
activating transcription factor 5; microRNA 4751	ATF5; MIR4751	1,41
solute carrier family 25, member 34	SLC25A34	1,41
nucleoporin 214kDa	NUP214	1,41
cancer/testis antigen family 47, member A7; cancer/testis antigen family 47, member A5; cancer/testis antigen family 47, member A6	CT47A7; CT47A5; CT47A6	1,41
sarcospan	SSPN	1,41
neuronal cell adhesion molecule	NRCAM	1,41
ataxin 7	ATXN7	1,41
IK cytokine, down-regulator of HLA II; microRNA 3655	IK; MIR3655	1,41
SPT7-like STAGA complex gamma subunit	SUPT7L	1,41
ferredoxin reductase	FDXR	1,41
mesoderm induction early response 1, transcriptional regulator	MIER1	1,41
thymosin beta 4, Y-linked	TMSB4Y	1,41
Transcript Identified by AceView, Entrez Gene ID(s) 4134	MAP4	1,41
ring finger protein 19B	RNF19B	1,41
SH3KBP1 binding protein 1	SHKBP1	1,41
dynein, cytoplasmic 1, light intermediate chain 2	DYNC1LI2	1,41
inhibitor of CDK, cyclin A1 interacting protein 1	INCA1	1,41
keratin associated protein 21-1	KRTAP21-1	1,41
serine/threonine kinase 40	STK40	1,41
ATP binding cassette subfamily F member 2	ABCF2	1,41
ring finger protein 31	RNF31	1,41
chromosome 10 open reading frame 90	C10orf90	1,41
zona pellucida binding protein	ZPBP	1,41
MAP kinase interacting serine/threonine kinase 2	MKNK2	1,41
retinitis pigmentosa 2 (X-linked recessive)	RP2	1,41

potassium channel, voltage gated subfamily A regulatory beta subunit 1	KCNAB1	1,41
translocator protein 2	TSPO2	1,41
protein tyrosine phosphatase, non-receptor type 11	PTPN11	1,41
ADP-ribosylation factor like GTPase 4C	ARL4C	1,41
olfactory receptor, family 52, subfamily E, member 1 (gene/pseudogene)	OR52E1	1,41
RAB2B, member RAS oncogene family	RAB2B	1,41
spermine synthase	SMS	1,41
TAF2 RNA polymerase II, TATA box binding protein (TBP)-associated factor, 150kDa	TAF2	1,41
sprouty-related, EVH1 domain containing 2	SPRED2	1,41
Memczak2013 ANTISENSE, CDS, coding, INTERNAL best transcript NM_014423	AFF4	1,41
CD3g molecule, gamma (CD3-TCR complex)	CD3G	1,41
Transcript Identified by AceView, Entrez Gene ID(s) 23670	TMEM2	1,41
damage-specific DNA binding protein 2	DDB2	1,41
family with sequence similarity 200, member B	FAM200B	1,41
cAMP responsive element modulator	CREM	1,41
chemokine (C-C motif) receptor 1	CCR1	1,41
tubulin tyrosine ligase-like family member 12	TTLL12	1,41
proliferation-associated 2G4	PA2G4	1,41
pogo transposable element with KRAB domain	POGK	1,41
tubulin tyrosine ligase-like family member 6	TTLL6	1,41
zinc finger, DHHC-type containing 18	ZDHHC18	1,41
WAP four-disulfide core domain 10B	WFDC10B	1,41
delta-like 4 (Drosophila)	DLL4	1,41
IQ motif containing D	IQCD	1,41
PC-esterase domain containing 1A	PCED1A	1,41
H2A histone family, member B1; H2A histone family, member B2; H2A histone family, member B3	H2AFB1; H2AFB2; H2AFB3	1,41
tetratricopeptide repeat domain 14	TTC14	1,41
poly(rC) binding protein 3	PCBP3	1,41
coatomer protein complex subunit gamma 2; testis specific 13	COPG2; TSGA13	1,41
olfactory receptor, family 5, subfamily M, member 1	OR5M1	1,41
DEAD (Asp-Glu-Ala-Asp) box polypeptide 20	DDX20	1,41
Zic family member 2	ZIC2	1,41
histone acetyltransferase 1	HAT1	1,41
inositol 1,4,5-trisphosphate receptor interacting protein-like 1	ITPRIPL1	1,41
bystin-like	BYSL	1,41
RALBP1 associated Eps domain containing 1	REPS1	1,41
transmembrane and coiled-coil domain family 2	TMCC2	1,41

ubiquitin specific peptidase 32 pseudogene 2; family with sequence similarity 106, member A; coiled-coil domain containing 144B (pseudogene)	USP32P2; FAM106A; CCDC144B	1,41
golgin A8 family, member A; golgin A8 family, member B	GOLGA8A; GOLGA8B	1,40
THUMP domain containing 1	THUMPD1	1,40
ring finger and FYVE-like domain containing E3 ubiquitin protein ligase	RFFL	1,40
arrestin, beta 2	ARRB2	1,40
COMM domain containing 6	COMMD6	1,40
Memczak2013 ANTISENSE, coding, INTERNAL, intronic best transcript NM_006990	WASF2	1,40
transcription factor 20 (AR1)	TCF20	1,40
transcription factor 20 (AR1)	TCF20	1,40
ATPase, H+ transporting, lysosomal 13kDa, V1 subunit G2	ATP6V1G2	1,40
COMM domain containing 4	COMMD4	1,40
sorting nexin 29 pseudogene 2; sorting nexin 29 pseudogene 1	SNX29P2; SNX29P1	1,40
zinc finger protein 585B	ZNF585B	1,40
dehydrogenase/reductase (SDR family) member 1	DHRS1	1,40
breast carcinoma amplified sequence 1	BCAS1	1,40
cylindromatosis (turban tumor syndrome)	CYLD	1,40
synaptojanin 1	SYNJ1	1,40
lysosomal trafficking regulator	LYST	1,40
chromosome 3 open reading frame 49	C3orf49	1,40
DDB1 and CUL4 associated factor 13	DCAF13	1,40
baculoviral IAP repeat containing 3	BIRC3	1,40
RAB3C, member RAS oncogene family	RAB3C	1,40
chromosome 8 open reading frame 88	C8orf88	1,40
chemokine (C-X-C motif) receptor 3	CXCR3	1,40
indoleamine 2,3-dioxygenase 1	IDO1	1,40
bridging integrator 1	BIN1	1,40
retinoic acid receptor, beta	RARB	1,40
zinc finger protein 737	ZNF737	1,40
RNA binding motif protein 48	RBM48	1,40
2-hydroxyacyl-CoA lyase 1	HACL1	1,40
SLP adaptor and CSK interacting membrane protein	SCIMP	1,40
ring finger protein 25	RNF25	1,40
Xg blood group; Xg pseudogene, Y-linked 2	XG; XGY2	1,40
RAB1B, member RAS oncogene family	RAB1B	1,40
trafficking protein particle complex 8	TRAPPC8	1,40
phosphate cytidylyltransferase 1, choline, alpha	PCYT1A	1,40
casein kinase 1, epsilon	CSNK1E	1,40
coiled-coil domain containing 84	CCDC84	1,40

chromosome 11 open reading frame 98; LBH domain containing 1	C11orf98; LBHD1	1,40
non-SMC condensin I complex subunit H	NCAPH	1,40
sphingomyelin phosphodiesterase, acid-like 3B	SMPDL3B	1,40
CD2 (cytoplasmic tail) binding protein 2	CD2BP2	1,40
proline rich 9	PRR9	1,40
neuralized E3 ubiquitin protein ligase 3	NEURL3	1,40
reticulon 4	RTN4	1,40
bromodomain, testis-specific	BRDT	1,40
G protein-coupled receptor 33 (gene/pseudogene)	GPR33	1,40
Memczak2013 ANTISENSE, CDS, coding, INTERNAL, UTR3 best transcript NM_004308	ARHGAP1	1,40
SPARC related modular calcium binding 2	SMOC2	1,40
mitogen-activated protein kinase kinase kinase kinase 2	MAP4K2	1,40
zinc finger protein 471	ZNF471	1,40
Transcript Identified by AceView, Entrez Gene ID(s) 22843	PPM1E	1,40
microphthalmia-associated transcription factor	MITF	1,40
ADAM metallopeptidase with thrombospondin type 1 motif 18	ADAMTS18	1,40
synaptogyrin 2	SYNGR2	1,40
abl-interactor 1	ABI1	1,40
nucleolar protein 10	NOL10	1,40
MAGE family member E2	MAGEE2	1,40
serpin peptidase inhibitor, clade H (heat shock protein 47), member 1, (collagen binding protein 1)	SERPINH1	1,40
sperm associated antigen 11B	SPAG11B	1,40
Zhang2013 ALT_ACCEPTOR, ALT_DONOR, coding, INTERNAL, intronic best transcript NM_000976	RPL12	1,40
zinc finger protein 235	ZNF235	1,40
sortilin-related VPS10 domain containing receptor 2	SORCS2	1,40
KIAA1522	KIAA1522	1,40
Rho GTPase activating protein 30	ARHGAP30	1,40
ficolin (collagen/fibrinogen domain containing lectin) 2	FCN2	1,40
vesicle associated membrane protein 1	VAMP1	1,40
zinc finger, MYND-type containing 12	ZMYND12	1,39
anoctamin 4	ANO4	1,39
cell division cycle 37; microRNA 1181	CDC37; MIR1181	1,39
origin recognition complex subunit 3	ORC3	1,39
ankyrin repeat domain 33	ANKRD33	1,39
Transcript Identified by AceView, Entrez Gene ID(s) 5426	POLE	1,39
dual specificity phosphatase 3	DUSP3	1,39

cancer/testis antigen family 47, member A9; cancer/testis antigen family 47, member A4; cancer/testis antigen family 47, member A11	CT47A9; CT47A4; CT47A11	1,39
cancer/testis antigen family 47, member A3; cancer/testis antigen family 47, member A10	CT47A3; CT47A10	1,39
cancer/testis antigen family 47, member A10; cancer/testis antigen family 47, member A8; cancer/testis antigen family 47, member A9	CT47A10; CT47A8; CT47A9	1,39
cancer/testis antigen family 47, member A6; cancer/testis antigen family 47, member A4	CT47A6; CT47A4	1,39
cancer/testis antigen family 47, member A3	CT47A3	1,39
cancer/testis antigen family 47, member A2	CT47A2	1,39
cancer/testis antigen family 47, member A12; cancer/testis antigen family 47, member A8	CT47A12; CT47A8	1,39
hydroxysteroid dehydrogenase like 1	HSDL1	1,39
Transcript Identified by AceView, Entrez Gene ID(s) 27019	DNAI1	1,39
synaptogyrin 3	SYNGR3	1,39
Transcript Identified by AceView, Entrez Gene ID(s) 8792	TNFRSF11A	1,39
vascular endothelial zinc finger 1	VEZF1	1,39
Jeck2013 ALT_ACCEPTOR, ALT_DONOR, coding, INTERNAL, intronic best transcript NM_012316	KPNA6	1,39
LSM10, U7 small nuclear RNA associated	LSM10	1,39
zinc finger protein 622	ZNF622	1,39
Transcript Identified by AceView, Entrez Gene ID(s) 8939	FUBP3	1,39
ubiquitin specific peptidase 17-like family member 12	USP17L12	1,39
glial cell derived neurotrophic factor	GDNF	1,39
ATP binding cassette subfamily G member 2 (Junior blood group)	ABCG2	1,39
claudin domain containing 1	CLDND1	1,39
excision repair cross-complementation group 6-like	ERCC6L	1,39
zinc finger protein 526	ZNF526	1,39
aarF domain containing kinase 1	ADCK1	1,39
guanylate kinase 1	GUK1	1,39
tetratricopeptide repeat domain 37	TTC37	1,39
tubulin tyrosine ligase	TTL	1,39
ubiquitin C-terminal hydrolase L5	UCHL5	1,39
Memczak2013 ANTISENSE, CDS, coding, INTERNAL, intronic best transcript NM_001178056	PARP8	1,39
COX17 cytochrome c oxidase copper chaperone	COX17	1,39
Rho GTPase activating protein 1	ARHGAP1	1,39
death effector domain containing 2	DEDD2	1,39
insulin like growth factor binding protein 2	IGFBP2	1,39

MIS12 kinetochore complex component	MIS12	1,39
glutamate rich 5	ERICH5	1,39
zinc finger and SCAN domain containing 10	ZSCAN10	1,39
gastric inhibitory polypeptide receptor	GIPR	1,39
synovial sarcoma, X breakpoint 4B	SSX4B	1,39
neuroplastin	NPTN	1,39
ectodysplasin A	EDA	1,39
antizyme inhibitor 1	AZIN1	1,39
Kin17 DNA and RNA binding protein	KIN	1,39
family with sequence similarity 76, member B	FAM76B	1,39
small nuclear ribonucleoprotein polypeptide E	SNRPE	1,39
Niemann-Pick disease, type C1	NPC1	1,39
NIMA-related kinase 5	NEK5	1,39
pantothenate kinase 4	PANK4	1,39
Memczak2013 ANTISENSE, coding, INTERNAL, intronic best transcript NM_181985	LILRA5	1,39
protein phosphatase 1, regulatory subunit 13B	PPP1R13B	1,39
chromosome 4 open reading frame 22	C4orf22	1,39
chromosome 2 open reading frame 42	C2orf42	1,39
DEAD/H (Asp-Glu-Ala-Asp/His) box helicase 11	DDX11	1,38
opioid binding protein/cell adhesion molecule-like	OPCML	1,38
golgin A6 family, member C	GOLGA6C	1,38
thioredoxin 2	TXN2	1,38
ESF1 nucleolar pre-rRNA processing protein homolog	ESF1	1,38
KIAA1614	KIAA1614	1,38
adenosine A3 receptor; transmembrane and immunoglobulin domain containing 3	ADORA3; TMIGD3	1,38
transcription factor 20 (AR1)	TCF20	1,38
transcription factor 20 (AR1)	TCF20	1,38
surfeit 2 [Source:HGNC Symbol;Acc:HGNC:11475]	SURF2	1,38
calmodulin 3 (phosphorylase kinase, delta)	CALM3	1,38
cyclic nucleotide gated channel beta 3	CNGB3	1,38
PROP paired-like homeobox 1	PROP1	1,38
lectin, galactoside-binding, soluble, 9B	LGALS9B	1,38
DPH2 homolog	DPH2	1,38
zinc finger and BTB domain containing 10	ZBTB10	1,38
HtrA serine peptidase 3	HTRA3	1,38
vesicle associated membrane protein 8	VAMP8	1,38
ATP binding cassette subfamily C member 11	ABCC11	1,38
ubiquitin associated protein 1	UBAP1	1,38
kallikrein related peptidase 2	KLK2	1,38
DGCR8 microprocessor complex subunit; microRNA 1306; microRNA 3618	DGCR8; MIR1306; MIR3618	1,38
small proline-rich protein 2D	SPRR2D	1,38
coiled-coil domain containing 9	CCDC9	1,38

centrosomal protein 120kDa	CEP120	1,38
NADH dehydrogenase (ubiquinone) 1 alpha subcomplex, 10, 42kDa	NDUFA10	1,38
tubulin, alpha 1c	TUBA1C	1,38
pre-mRNA processing factor 3	PRPF3	1,38
hes-related family bHLH transcription factor with YRPW motif 2	HEY2	1,38
E1A binding protein p400; small nucleolar RNA, H/ACA box 49	EP400; SNORA49	1,38
F-box protein 6	FBXO6	1,38
junctophilin 2	JPH2	1,38
interferon induced transmembrane protein 3	IFITM3	1,38
RNA binding motif protein 38	RBM38	1,38
myosin IXA	MYO9A	1,38
PPP5 tetratricopeptide repeat domain containing 1	PPP5D1	1,38
phosphodiesterase 12	PDE12	1,38
dual specificity tyrosine-(Y)-phosphorylation regulated kinase 1B	DYRK1B	1,38
diacylglycerol kinase, zeta	DGKZ	1,38
polymerase (DNA directed) iota	POLI	1,38
phenylalanyl-tRNA synthetase 2, mitochondrial	FARS2	1,38
zinc finger protein 578	ZNF578	1,38
adaptor-related protein complex 3, mu 1 subunit	AP3M1	1,38
inositol polyphosphate multikinase	IPMK	1,38
PHD finger protein 1	PHF1	1,38
MICAL-like 2	MICALL2	1,38
chromosome 11 open reading frame 49	C11orf49	1,38
chloride intracellular channel 1	CLIC1	1,38
tetratricopeptide repeat, ankyrin repeat and coiled-coil containing 2	TANC2	1,38
G protein-coupled receptor kinase interacting ArfGAP 1	GIT1	1,38
collagen, type VI, alpha 3	COL6A3	1,38
BUB1 mitotic checkpoint serine/threonine kinase	BUB1	1,38
sciellin	SCEL	1,38
solute carrier family 51, alpha subunit	SLC51A	1,38
endogenous Bornavirus-like nucleoprotein 1	EBLN1	1,38
aquaporin 7	AQP7	1,38
KIT ligand	KITLG	1,38
transmembrane protein 63A	TMEM63A	1,38
aconitase 2, mitochondrial	ACO2	1,38
formin 1	FMN1	1,38
signal transducer and activator of transcription 1	STAT1	1,38
kelch-like family member 28	KLHL28	1,37
collagen, type XVII, alpha 1; microRNA 936	COL17A1; MIR936	1,37

zinc finger protein 596	ZNF596	1,37
TP53 induced glycolysis regulatory phosphatase	TIGAR	1,37
ring finger protein 157	RNF157	1,37
tetratricopeptide repeat domain 24	TTC24	1,37
Cbl proto-oncogene, E3 ubiquitin protein ligase	CBL	1,37
growth hormone inducible transmembrane protein	GHITM	1,37
interferon, alpha 10	IFNA10	1,37
NOP2/Sun domain family, member 5	NSUN5	1,37
Transcript Identified by AceView, Entrez Gene ID(s) 23126	POGZ	1,37
zinc finger protein 865	ZNF865	1,37
autophagy related 16-like 1	ATG16L1	1,37
family with sequence similarity 188, member A	FAM188A	1,37
RNA binding motif protein 33	RBM33	1,37
dorsal inhibitory axon guidance protein	DRAXIN	1,37
protease, serine, 22	PRSS22	1,37
kinase D-interacting substrate 220kDa	KIDINS220	1,37
NADH dehydrogenase (ubiquinone) 1 alpha subcomplex, 10, 42kDa	NDUFA10	1,37
armadillo repeat containing 3	ARMC3	1,37
acylaminoacyl-peptide hydrolase	APEH	1,37
high mobility group nucleosome binding domain 5	HMGN5	1,37
ADAM metallopeptidase with thrombospondin type 1 motif 1	ADAMTS1	1,37
vacuolar protein sorting 53 homolog (S. cerevisiae); ribosomal protein S4X pseudogene 17	VPS53; RPS4XP17	1,37
glutamate receptor, ionotropic, N-methyl D-aspartate-associated protein 1 (glutamate binding)	GRINA	1,37
tripeptidyl peptidase II	TPP2	1,37
zinc finger, DHHC-type containing 6	ZDHHC6	1,37
WNT1 inducible signaling pathway protein 2	WISP2	1,37
family with sequence similarity 120A opposite strand	FAM120AOS	1,37
alpha tubulin acetyltransferase 1	ATAT1	1,37
GTF2H2 family member C, copy 2; GTF2H2 family member C; general transcription factor IIH subunit 2B (pseudogene)	GTF2H2C_2; GTF2H2C; GTF2H2B	1,37
T-box 21	TBX21	1,37
Memczak2013 ALT_ACCEPTOR, ALT_DONOR, coding, INTERNAL, intronic best transcript NM_001136017	CCND3	1,37
thioredoxin-related transmembrane protein 2; chromosome 11 open reading frame 31	TMX2; C11orf31	1,37
heat shock 27kDa protein family, member 7 (cardiovascular)	HSPB7	1,37
importin 11; leucine rich repeat containing 70	IPO11; LRRC70	1,37
phosphorylase, glycogen; brain	PYGB	1,37

ubiquitin specific peptidase 11	USP11	1,37
S-antigen; retina and pineal gland (arrestin)	SAG	1,37
chromosome 9 open reading frame 85	C9orf85	1,37
apolipoprotein B mRNA editing enzyme, catalytic polypeptide-like 3C	APOBEC3C	1,37
nucleolar protein 9	NOL9	1,37
claspin	CLSPN	1,37
zinc finger protein 619	ZNF619	1,37
zinc finger protein 330	ZNF330	1,37
AVL9 homolog (S. cerevisiase)	AVL9	1,37
nuclear pore complex interacting protein family, member B8	NPIPB8	1,37
zinc finger protein 304	ZNF304	1,37
Transcript Identified by AceView, Entrez Gene ID(s) 54940	OCIAD1	1,37
claudin 19	CLDN19	1,37
DMRT-like family B with proline-rich C-terminal, 1	DMRTB1	1,37
septin 1	sept-01	1,37
autophagy/beclin-1 regulator 1	AMBRA1	1,37
amyloid beta (A4) precursor protein-binding, family B, member 2	APBB2	1,37
zinc finger protein 610	ZNF610	1,37
polymerase I and transcript release factor	PTRF	1,37
guanine nucleotide binding protein-like 3 (nucleolar)-like	GNL3L	1,37
WD repeat domain 83 opposite strand	WDR83OS	1,37
podoplanin	PDPN	1,37
keratin 35, type I	KRT35	1,37
semaphorin 7A, GPI membrane anchor (John Milton Hagen blood group)	SEMA7A	1,37
myosin XVIIIB	MYO18B	1,37
KRAB box domain containing 4	KRBOX4	1,37
sema domain, immunoglobulin domain (Ig), transmembrane domain (TM) and short cytoplasmic domain, (semaphorin) 4B	SEMA4B	1,37
neutrophil cytosolic factor 1	NCF1	1,37
solute carrier family 26 (anion exchanger), member 11	SLC26A11	1,37
SNF8, ESCRT-II complex subunit	SNF8	1,36
Rho-associated, coiled-coil containing protein kinase 2	ROCK2	1,36
DEAD (Asp-Glu-Ala-Asp) box polypeptide 59	DDX59	1,36
empty spiracles homeobox 2	EMX2	1,36
Transcript Identified by AceView, Entrez Gene ID(s) 8089	YEATS4	1,36
ARRDC1 antisense RNA 1	ARRDC1-AS1	1,36
ubiquitin-like 5	UBL5	1,36

SMG8 nonsense mediated mRNA decay factor	SMG8	1,36
ADAM metallopeptidase with thrombospondin type 1 motif 10	ADAMTS10	1,36
ADAM metallopeptidase with thrombospondin type 1 motif 12	ADAMTS12	1,36
DEAH (Asp-Glu-Ala-His) box polypeptide 35	DHX35	1,36
BCL2/adenovirus E1B 19kDa interacting protein 2	BNIP2	1,36
colony stimulating factor 3	CSF3	1,36
proteasome subunit beta 2	PSMB2	1,36
runt-related transcription factor 2	RUNX2	1,36
oxysterol binding protein-like 8	OSBPL8	1,36
Ras and Rab interactor 3	RIN3	1,36
glutamic-oxaloacetic transaminase 1, soluble	GOT1	1,36
bassoon presynaptic cytomatrix protein	BSN	1,36
SH2B adaptor protein 3	SH2B3	1,36
akirin 2	AKIRIN2	1,36
SIK family kinase 3	SIK3	1,36
solute carrier organic anion transporter family, member 5A1	SLCO5A1	1,36
Jeck2013 ALT_ACCEPTOR, ALT_DONOR, coding, INTERNAL, intronic best transcript NM_032531	KIRREL3	1,36
heat shock transcription factor, Y-linked 2	HSFY2	1,36
syntrophin, gamma 2	SNTG2	1,36
protocadherin 7	PCDH7	1,36
ubiquitin specific peptidase 46	USP46	1,36
ankyrin repeat domain 39	ANKRD39	1,36
fidgetin-like 2	FIGNL2	1,36
ribosomal RNA processing 36	RRP36	1,36
barttin CLCNK-type chloride channel accessory beta subunit	BSND	1,36
tumor necrosis factor (ligand) superfamily, member 13b	TNFSF13B	1,36
vacuolar protein sorting 37 homolog A (S. cerevisiae)	VPS37A	1,36
trace amine associated receptor 1	TAAR1	1,36
neurogranin (protein kinase C substrate, RC3)	NRGN	1,36
estrogen receptor 2 (ER beta)	ESR2	1,36
RAD9 checkpoint clamp component B	RAD9B	1,36
SUMO1/sentrin/SMT3 specific peptidase 3	SENP3	1,36
CCAAT/enhancer binding protein (C/EBP), zeta	CEBPZ	1,36
ubiquitin specific peptidase 38	USP38	1,36
ADAM metallopeptidase domain 9	ADAM9	1,36
retinoic acid receptor, gamma	RARG	1,36
galactosidase beta 1 like	GLB1L	1,36
thyroid hormone responsive	THRSP	1,36
fibroblast growth factor 20	FGF20	1,36

G protein-coupled receptor 4	GPR4	1,36
zinc finger protein 410	ZNF410	1,36
alkaline phosphatase, placental like 2	ALPPL2	1,36
kelch-like family member 20	KLHL20	1,36
keratin 222, type II	KRT222	1,36
ArfGAP with FG repeats 1; microRNA 5703	AGFG1; MIR5703	1,36
tumor protein p53 binding protein 2	TP53BP2	1,36
GATS, stromal antigen 3 opposite strand	GATS	1,36
R3H domain containing 1	R3HDM1	1,36
zinc finger and BTB domain containing 7A	ZBTB7A	1,36
SCY1-like, kinase-like 2	SCYL2	1,36
ubiquitin specific peptidase 6	USP6	1,36
proteasome maturation protein	POMP	1,36
cadherin 5, type 2 (vascular endothelium)	CDH5	1,36
rabphilin 3A-like (without C2 domains)	RPH3AL	1,36
cysteine and glycine-rich protein 2	CSRP2	1,36
ring finger protein 103	RNF103	1,36
SWI5 homologous recombination repair protein	SWI5	1,36
ERGIC and golgi 2	ERGIC2	1,36
Transcript Identified by AceView, Entrez Gene ID(s) 10777	ARPP21	1,36
PTPRF interacting protein, binding protein 2 (liprin beta 2)	PPFIBP2	1,36
poly(ADP-ribose) polymerase family member 14	PARP14	1,36
zinc finger protein 501 [Source:HGNC Symbol;Acc:HGNC:23717]	ZNF501	1,36
leucine rich repeat and fibronectin type III domain containing 2	LRFN2	1,36
Transcript Identified by AceView, Entrez Gene ID(s) 7511	XPNPEP1	1,36
Memczak2013 ALT_ACCEPTOR, ALT_DONOR, coding, INTERNAL, intronic best transcript NM_033071	SYNE1	1,36
spermatid associated	SPERT	1,36
sphingomyelin synthase 1	SGMS1	1,36
Transcript Identified by AceView, Entrez Gene ID(s) 135112	NCOA7	1,36
FXYD domain containing ion transport regulator 4	FXYD4	1,36
zinc finger, FYVE domain containing 9	ZFYVE9	1,36
chromosome 7 open reading frame 34	C7orf34	1,36
family with sequence similarity 24, member B	FAM24B	1,36
diaphanous-related formin 2	DIAPH2	1,36
brain-specific homeobox	BSX	1,36
tRNA methyltransferase 5	TRMT5	1,36
Memczak2013 ANTISENSE, coding, INTERNAL, intronic best transcript NM_001170794	BACH2	1,36

EPH receptor A7	EPHA7	1,36
long intergenic non-protein coding RNA 482	LINC00482	1,36
uroplakin 1B	UPK1B	1,36
centrosomal protein 164kDa	CEP164	1,36
actin related protein 2/3 complex subunit 5-like	ARPC5L	1,36
activating transcription factor 7; neuropeptide FF-amide peptide precursor	ATF7; NPFF	1,36
ADP-ribosylation factor like GTPase 5C	ARL5C	1,36
barrier to autointegration factor 1	BANF1	1,36
shugoshin-like 2 (S. pombe)	SGOL2	1,36
nucleoporin 107kDa	NUP107	1,36
ferritin, heavy polypeptide 1	FTH1	1,36
NAC alpha domain containing	NACAD	1,36
CDK2-associated, cullin domain 1	CACUL1	1,36
RWD domain containing 2B	RWDD2B	1,36
G protein-coupled receptor 50	GPR50	1,36
zinc finger protein 516	ZNF516	1,35
SH3 and multiple ankyrin repeat domains 3	SHANK3	1,35
5-hydroxytryptamine (serotonin) receptor 7, adenylate cyclase-coupled	HTR7	1,35
charged multivesicular body protein 5	CHMP5	1,35
transmembrane protein 239; chromosome 20 open reading frame 141	TMEM239; C20orf141	1,35
sortilin-related VPS10 domain containing receptor 1	SORCS1	1,35
melanoma associated antigen (mutated) 1-like 1	MUM1L1	1,35
WD repeat and SOCS box containing 2	WSB2	1,35
thymocyte selection associated	THEMIS	1,35
proteasome 26S subunit, non-ATPase 6	PSMD6	1,35
ets variant 6	ETV6	1,35
mannose-P-dolichol utilization defect 1	MPDU1	1,35
family with sequence similarity 89, member B	FAM89B	1,35
chromosome 7 open reading frame 43; microRNA 4658	C7orf43; MIR4658	1,35
alkB homolog 3, alpha-ketoglutarate-dependent dioxygenase; SEC14-like 1 pseudogene 1	ALKBH3; SEC14L1P1	1,35
zinc finger protein 479	ZNF479	1,35
importin 4	IPO4	1,35
chemokine (C-C motif) ligand 28	CCL28	1,35
LY6/PLAUR domain containing 1	LYPD1	1,35
nuclear factor I/C (CCAAT-binding transcription factor)	NFIC	1,35
paired-like homeobox 2b	PHOX2B	1,35
MEF2 activating motif and SAP domain containing transcriptional regulator	MAMSTR	1,35
Memczak2013 ALT_ACCEPTOR, ALT_DONOR, coding, INTERNAL, intronic best transcript NM_001076787	TP53I11	1,35

BH3 interacting domain death agonist	BID	1,35
zinc finger protein 525	ZNF525	1,35
RAN binding protein 6	RANBP6	1,35
actin-like 6A	ACTL6A	1,35
BSD domain containing 1	BSDC1	1,35
ankyrin repeat domain 12	ANKRD12	1,35
Memczak2013 ALT_ACCEPTOR, ALT_DONOR, coding, INTERNAL, intronic best transcript NM_013314	BLNK	1,35
mitofusin 2	MFN2	1,35
mitofusin 1	MFN1	1,35
transmembrane and coiled-coil domains 3	TMCO3	1,35
general transcription factor IIIC subunit 3	GTF3C3	1,35
Memczak2013 ANTISENSE, coding, INTERNAL, intronic best transcript NM_001166373	MARCH1	1,35
CTD phosphatase subunit 1	CTDP1	1,35
AT rich interactive domain 5B (MRF1-like)	ARID5B	1,35
HAUS augmin like complex subunit 8	HAUS8	1,35
family with sequence similarity 114, member A2	FAM114A2	1,35
secretoglobin, family 2A, member 2	SCGB2A2	1,35
zinc finger protein 662	ZNF662	1,35
ubiquitin conjugating enzyme E2A	UBE2A	1,35
Ras homolog enriched in brain like 1	RHEBL1	1,35
activating signal cointegrator 1 complex subunit 2	ASCC2	1,35
INO80 complex subunit B	INO80B	1,35
otoancorin	OTOA	1,35
nudix hydrolase 9	NUDT9	1,35
mitochondrial calcium uniporter regulator 1	MCUR1	1,35
desumoylating isopeptidase 1	DESI1	1,35
defensin, beta 1	DEFB1	1,35
junctophilin 4	JPH4	1,35
septin 10	sept-10	1,35
olfactory receptor, family 52, subfamily A, member 5	OR52A5	1,35
valosin containing protein lysine (K) methyltransferase	VCPKMT	1,35
FSHD region gene 2 family, member C	FRG2C	1,35
karyopherin alpha 1	KPNA1	1,35
occludin	OCLN	1,35
endothelial differentiation-related factor 1	EDF1	1,35
saitohin	STH	1,35
Memczak2013 ANTISENSE, CDS, coding, INTERNAL best transcript NM_001039590	USP9X	1,35
chromosome 7 open reading frame 50	C7orf50	1,35
peroxisomal biogenesis factor 14	PEX14	1,35
Transcript Identified by AceView, Entrez Gene ID(s) 162494	RHBDL3	1,35
basic helix-loop-helix family, member a9	BHLHA9	1,35

transglutaminase 3	TGM3	1,35
mab-21-like 2 (C. elegans)	MAB21L2	1,35
endogenous retrovirus group K13, member 1	ERVK13-1	1,35
BCL2-like 1	BCL2L1	1,35
ATPase type 13A2	ATP13A2	1,35
thrombospondin 4	THBS4	1,35
serine-rich single-pass membrane protein 1	SSMEM1	1,35
IQ motif and Sec7 domain 3	IQSEC3	1,35
GRAM domain containing 2	GRAMD2	1,35
OTU deubiquitinase 7B	OTUD7B	1,35
unc-13 homolog D (C. elegans)	UNC13D	1,35
helt bHLH transcription factor	HELT	1,35
heat shock 70kDa protein 2	HSPA2	1,35
TEA domain family member 1 (SV40 transcriptional enhancer factor)	TEAD1	1,35
actin related protein 2/3 complex subunit 1A	ARPC1A	1,35
keratin associated protein 20-1	KRTAP20-1	1,35
DENN/MADD domain containing 2D	DENND2D	1,35
interleukin 9	IL9	1,35
myelin-associated oligodendrocyte basic protein	MOBP	1,35
olfactory receptor, family 14, subfamily A, member 16	OR14A16	1,35
inner mitochondrial membrane peptidase subunit 1	IMMP1L	1,35
ZFP64 zinc finger protein	ZFP64	1,35
DEAD (Asp-Glu-Ala-Asp) box helicase 24	DDX24	1,35
olfactory receptor, family 52, subfamily E, member 6	OR52E6	1,35
LMBR1 domain containing 2	LMBRD2	1,35
ATP binding cassette subfamily A member 4	ABCA4	1,35
spastic paraplegia 7 (pure and complicated autosomal recessive)	SPG7	1,35
ubiquitination factor E4A	UBE4A	1,35
family with sequence similarity 21, member A	FAM21A	1,35
tetratricopeptide repeat domain 21A; microRNA 6822	TTC21A; MIR6822	1,35
C-x(9)-C motif containing 2	CMC2	1,35
EGF domain-specific O-linked N-acetylglucosamine (GlcNAc) transferase	EOGT	1,35
solute carrier family 35 (adenosine 3-phospho 5-phosphosulfate transporter), member B3	SLC35B3	1,35
PRAME family member 8	PRAMEF8	1,34
late endosomal/lysosomal adaptor, MAPK and MTOR activator 4	LAMTOR4	1,34
zinc finger protein 616	ZNF616	1,34
1-acylglycerol-3-phosphate O-acyltransferase 4	AGPAT4	1,34
calcium homeostasis modulator 1	CALHM1	1,34
BCL2-like 10 (apoptosis facilitator)	BCL2L10	1,34
coiled-coil domain containing 30	CCDC30	1,34

trafficking protein particle complex 4	TRAPPC4	1,34
DnaJ (Hsp40) homolog, subfamily A, member 1	DNAJA1	1,34
serine/threonine kinase 32C	STK32C	1,34
Homo sapiens proteasome (prosome, macropain) 26S subunit, ATPase, 1, mRNA (cDNA clone MGC:8541 IMAGE:2822718), complete cds.; Homo sapiens proteasome (prosome, macropain) 26S subunit, ATPase, 1, mRNA (cDNA clone MGC:24583 IMAGE:4133348), complete cds.; Homo sapiens proteasome (prosome, macropain) 26S subunit, ATPase, 1, mRNA (cDNA clone MGC:86994 IMAGE:5264945), complete cds.; Homo sapiens proteasome (prosome, macropain) 26S subunit, ATPase, 1, mRNA (cDNA clone MGC:88853 IMAGE:5456334), complete cds.	PSMC1	1,34
chromosome 1 open reading frame 189	C1orf189	1,34
calcineurin-like EF-hand protein 2	CHP2	1,34
cancer/testis antigen family 47, member B1	CT47B1	1,34
phosphatidylglycerophosphate synthase 1	PGS1	1,34
zinc finger protein 320	ZNF320	1,34
synovial sarcoma, X breakpoint 3	SSX3	1,34
MAGE family member A1	MAGEA1	1,34
autophagy related 16-like 1	ATG16L1	1,34
BCL2-like 14 (apoptosis facilitator)	BCL2L14	1,34
Memczak2013 ANTISENSE, coding, INTERNAL, intronic best transcript NM_175061	JAZF1	1,34
lipocalin 6	LCN6	1,34
anti-silencing function 1A histone chaperone	ASF1A	1,34
VPS11, CORVET/HOPS core subunit [Source:HGNC Symbol;Acc:HGNC:14583]	VPS11	1,34
carcinoembryonic antigen-related cell adhesion molecule 8	CEACAM8	1,34
zinc finger CCCH-type containing 13	ZC3H13	1,34
intersectin 2	ITSN2	1,34
gasdermin C	GSDMC	1,34
zinc finger protein 678	ZNF678	1,34
ATP synthase, H+ transporting, mitochondrial Fo complex subunit C1 (subunit 9)	ATP5G1	1,34
protein kinase N2	PKN2	1,34
Kruppel-like factor 10	KLF10	1,34
zinc finger protein 449	ZNF449	1,34
guanylate binding protein 5	GBP5	1,34
actin related protein 2/3 complex subunit 1B	ARPC1B	1,34
bolA family member 2B; bolA family member 2	BOLA2B; BOLA2	1,34
OCIA domain containing 2	OCIAD2	1,34

origin recognition complex subunit 5	ORC5	1,34
general transcription factor IIH subunit 2	GTF2H2	1,34
apolipoprotein L domain containing 1; DEAD (Asp-Glu-Ala-Asp) box polypeptide 47	APOLD1; DDX47	1,34
ubiquitin associated protein 2 like	UBAP2L	1,34
trafficking protein particle complex 4; microRNA 3656	TRAPPC4; MIR3656	1,34
zinc finger protein 501 [Source:HGNC Symbol;Acc:HGNC:23717]	ZNF501	1,34
sparc/osteonectin, cwcv and kazal-like domains proteoglycan (testican) 1	SPOCK1	1,34
histone cluster 2, H2be	HIST2H2BE	1,34
amyloid beta (A4) precursor protein-binding, family A, member 3	APBA3	1,34
RELT-like 2	RELL2	1,34
RAD17 checkpoint clamp loader component	RAD17	1,34
NDRG family member 3	NDRG3	1,34
serpin peptidase inhibitor, clade I (pancpin), member 2	SERPINI2	1,34
chromosome 16 open reading frame 72	C16orf72	1,34
zinc finger with KRAB and SCAN domains 5	ZKSCAN5	1,34
ras homolog family member D	RHOD	1,34
numb homolog (Drosophila)-like	NUMBL	1,34
phosphatidylinositol binding clathrin assembly protein	PICALM	1,34
ubiquitin specific peptidase 16	USP16	1,34
phosphatase and actin regulator 3	PHACTR3	1,34
proteasome 26S subunit, ATPase 2	PSMC2	1,34
folate receptor 1 (adult)	FOLR1	1,34
chemokine (C-X-C motif) ligand 17	CXCL17	1,34
StAR-related lipid transfer domain containing 8	STARD8	1,34
synergin, gamma	SYNRG	1,34
developmental pluripotency associated 4	DPPA4	1,34
polycystic kidney disease 1 like 1	PKD1L1	1,34
junction mediating and regulatory protein, p53 cofactor	JMY	1,34
long intergenic non-protein coding RNA 222	LINC00222	1,34
transcription elongation factor A (SII) N-terminal and central domain containing 2; microRNA 4781	TCEANC2; MIR4781	1,34
zinc finger E-box binding homeobox 2	ZEB2	1,34
actin related protein 2/3 complex subunit 3	ARPC3	1,34
phospholipase A2, group V	PLA2G5	1,34
biorientation of chromosomes in cell division 1-like 1	BOD1L1	1,34
myoglobin	MB	1,34
CDC42 binding protein kinase gamma (DMPK-like)	CDC42BPG	1,34
forkhead box C2	FOXC2	1,34
enkurin, TRPC channel interacting protein	ENKUR	1,34
WD repeat domain 54	WDR54	1,34

troponin C type 2 (fast)	TNNC2	1,34
long intergenic non-protein coding RNA 525	LINC00525	1,34
ankyrin repeat domain 13B	ANKRD13B	1,34
prostaglandin E synthase 3 (cytosolic)	PTGES3	1,34
abl-interactor 2	ABI2	1,34
proteasome 26S subunit, ATPase 4	PSMC4	1,34
olfactory receptor, family 5, subfamily H, member 6 (gene/pseudogene)	OR5H6	1,33
glutamate decarboxylase 1	GAD1	1,33
sphingosine-1-phosphate receptor 1	S1PR1	1,33
C1q and tumor necrosis factor related protein 9B	C1QTNF9B	1,33
surfactant associated 2	SFTA2	1,33
GATA zinc finger domain containing 2A; microRNA 640	GATAD2A; MIR640	1,33
6-phosphofructo-2-kinase/fructose-2,6-biphosphatase 2	PFKFB2	1,33
slingshot protein phosphatase 3	SSH3	1,33
GUF1 homolog, GTPase	GUF1	1,33
inositol polyphosphate-5-phosphatase D	INPP5D	1,33
ubiquitin specific peptidase 45	USP45	1,33
Transcript Identified by AceView, Entrez Gene ID(s) 64895	PAPOLG	1,33
Transcript Identified by AceView, Entrez Gene ID(s) 124936	CYB5D2	1,33
uncharacterized LOC102724957; novel transcript	LOC102724957; RP11-396O20.1	1,33
ADP-ribosylation factor GTPase activating protein 1; microRNA 4326	ARFGAP1; MIR4326	1,33
SH2 domain containing 3A	SH2D3A	1,33
solute carrier family 12 (potassium/chloride transporter), member 6	SLC12A6	1,33
neuronal differentiation 4	NEUROD4	1,33
reticulon 4 receptor-like 2	RTN4RL2	1,33
BTAF1 RNA polymerase II, B-TFIID transcription factor-associated, 170kDa	BTAF1	1,33
TAF9 RNA polymerase II, TATA box binding protein (TBP)-associated factor, 32kDa; adenylate kinase 6	TAF9; AK6	1,33
calcium binding and coiled-coil domain 2	CALCOCO2	1,33
centromere protein T	CENPT	1,33
hydroxyacylglutathione hydrolase-like	HAGHL	1,33
tumor necrosis factor receptor superfamily, member 1B; microRNA 4632; microRNA 7846	TNFRSF1B; MIR4632; MIR7846	1,33
zinc finger protein 831	ZNF831	1,33
ArfGAP with coiled-coil, ankyrin repeat and PH domains 2	ACAP2	1,33

signal-induced proliferation-associated 1 like 2	SIPA1L2	1,33
small muscle protein, X-linked	SMPX	1,33
TNF receptor-associated factor 5	TRAF5	1,33
C-type lectin domain family 4, member M	CLEC4M	1,33
dynein, cytoplasmic 1, heavy chain 1	DYNC1H1	1,33
spindle and kinetochore associated complex subunit 3	SKA3	1,33
PRP40 homolog, pre-mRNA processing factor B	PRPF40B	1,33
KIAA1549-like	KIAA1549L	1,33
zona pellucida glycoprotein 2 (sperm receptor)	ZP2	1,33
JNK1/MAPK8-associated membrane protein	JKAMP	1,33
plakophilin 3	PKP3	1,33
TSR1, 20S rRNA accumulation, homolog (S. cerevisiae)	TSR1	1,33
5-hydroxytryptamine (serotonin) receptor 1F, G protein-coupled	HTR1F	1,33
polypyrimidine tract binding protein 3	PTBP3	1,33
matrix metallopeptidase 13	MMP13	1,33
zinc finger protein 334	ZNF334	1,33
tubulin, alpha 4b	TUBA4B	1,33
olfactory receptor, family 4, subfamily N, member 5	OR4N5	1,33
arginase 2	ARG2	1,33
amyotrophic lateral sclerosis 2 chromosome region candidate 11	ALS2CR11	1,33
Rac/Cdc42 guanine nucleotide exchange factor 6	ARHGEF6	1,33
prostaglandin reductase 2	PTGR2	1,33
keratin 38, type I	KRT38	1,33
guanine nucleotide binding protein (G protein), beta polypeptide 3	GNB3	1,33
LIM domain containing preferred translocation partner in lipoma	LPP	1,33
LFNG O-fucosylpeptide 3-beta-N-acetylglucosaminyltransferase; microRNA 4648	LFNG; MIR4648	1,33
glycogen synthase kinase 3 alpha	GSK3A	1,33
WD repeat domain 63	WDR63	1,33
ARP1 actin-related protein 1 homolog A, centractin alpha (yeast)	ACTR1A	1,33
GTPase activating Rap/RanGAP domain-like 3	GARNL3	1,33
Yip1 domain family member 7	YIPF7	1,33
trafficking protein particle complex 2-like	TRAPPC2L	1,33
lin-28 homolog A (C. elegans)	LIN28A	1,33
sorting nexin 9	SNX9	1,33
chromodomain helicase DNA binding protein 2; microRNA 3175	CHD2; MIR3175	1,33
G protein-coupled receptor, class C, group 5, member C	GPRC5C	1,33
MT-RNR2-like 13	MTRNR2L13	1,33
ATP binding cassette subfamily E member 1	ABCE1	1,33

glutamyl-tRNA(Gln) amidotransferase, subunit B	GATB	1,33
transmembrane protein 208	TMEM208	1,33
JRK-like	JRKL	1,33
acidic residue methyltransferase 1	ARMT1	1,33
zinc finger, DHHC-type containing 2	ZDHHC2	1,33
RAB11 family interacting protein 5 (class I)	RAB11FIP5	1,33
UPK1A antisense RNA 1	UPK1A-AS1	1,33
Jeck2013 ANTISENSE, coding, INTERNAL, intronic best transcript NM_001207014	SERPINH1	1,33
nuclear RNA export factor 5	NXF5	1,33
NIMA-related kinase 2	NEK2	1,33
fibroblast growth factor 1 (acidic)	FGF1	1,33
troponin T type 1 (skeletal, slow)	TNNT1	1,33
forkhead box K1	FOXK1	1,33
chemokine (C-C motif) ligand 7	CCL7	1,33
sulfotransferase family 1A member 4; sulfotransferase family 1A member 3	SULT1A4; SULT1A3	1,33
olfactomedin 4	OLFM4	1,33
serine peptidase inhibitor, Kunitz type 4	SPINT4	1,33
cytochrome b561 family, member D1	CYB561D1	1,33
HtrA serine peptidase 2	HTRA2	1,33
complement component 1, q subcomponent-like 2	C1QL2	1,33
growth hormone receptor	GHR	1,33
autophagy related 16-like 1	ATG16L1	1,33
syntrophin, alpha 1	SNTA1	1,33
suppressor of fused homolog (Drosophila)	SUFU	1,33
kinesin family member 3A	KIF3A	1,33
host cell factor C1	HCFC1	1,33
slingshot protein phosphatase 2	SSH2	1,33
nucleosome assembly protein 1-like 3	NAP1L3	1,33
calmodulin-like 6	CALML6	1,33
transmembrane protein 167B	TMEM167B	1,33
ALG3, alpha-1,3- mannosyltransferase	ALG3	1,33
variable charge, X-linked 3A; variable charge, X-linked	VCX3A; VCX	1,33
erbb2 interacting protein	ERBB2IP	1,33
mixed lineage kinase domain-like	MLKL	1,33
ribosome production factor 1 homolog	RPF1	1,32
phospholipase A2, group XV	PLA2G15	1,32
3-phosphoadenosine 5-phosphosulfate synthase 2	PAPSS2	1,32
solute carrier family 2 (facilitated glucose transporter), member 1	SLC2A1	1,32
torsin family 1, member A (torsin A)	TOR1A	1,32
C-type lectin domain family 3, member B	CLEC3B	1,32
piezo-type mechanosensitive ion channel component 1	PIEZO1	1,32

neural precursor cell expressed, developmentally down-regulated 1	NEDD1	1,32
small nuclear RNA activating complex polypeptide 1	SNAPC1	1,32
solute carrier family 12 (potassium/chloride transporter), member 5	SLC12A5	1,32
golgi SNAP receptor complex member 2	GOSR2	1,32
capping protein (actin filament) muscle Z-line, beta	CAPZB	1,32
coiled-coil-helix-coiled-coil-helix domain containing 5	CHCHD5	1,32
ubiquitin-conjugating enzyme E2Q family member 2	UBE2Q2	1,32
S-antigen; retina and pineal gland (arrestin)	SAG	1,32
zinc finger, HIT-type containing 6	ZNHIT6	1,32
tektin 4	TEKT4	1,32
BicC family RNA binding protein 1	BICC1	1,32
NADH dehydrogenase, subunit 5 (complex I)	ND5	1,32
YTH N(6)-methyladenosine RNA binding protein 1	YTHDF1	1,32
mediator complex subunit 9	MED9	1,32
protein phosphatase 1, catalytic subunit, alpha isozyme	PPP1CA	1,32
SRY box 15	SOX15	1,32
ethylmalonic encephalopathy 1	ETHE1	1,32
caspase 3	CASP3	1,32
YOD1 deubiquitinase	YOD1	1,32
unc-45 myosin chaperone A	UNC45A	1,32
high mobility group nucleosomal binding domain 2	HMGN2	1,32
mutS homolog 2	MSH2	1,32
rhomboid 5 homolog 1 (Drosophila)	RHBDF1	1,32
O-acyl-ADP-ribose deacylase 1	OARD1	1,32
protein phosphatase 3, regulatory subunit B, beta	PPP3R2	1,32
V-set and immunoglobulin domain containing 1	VSIG1	1,32
protocadherin beta 10	PCDHB10	1,32
protein phosphatase 1, regulatory (inhibitor) subunit 2	PPP1R2	1,32
stress-induced phosphoprotein 1	STIP1	1,32
glutamate decarboxylase like 1	GADL1	1,32
RAB35, member RAS oncogene family	RAB35	1,32
ring finger protein 112	RNF112	1,32
dynein, cytoplasmic 1, intermediate chain 1	DYNC1I1	1,32
pleckstrin homology domain containing, family A member 8 pseudogene 1	PLEKHA8P1	1,32
serpin peptidase inhibitor, clade E (nexin, plasminogen activator inhibitor type 1), member 3	SERPINE3	1,32
deoxycytidine kinase	DCK	1,32
solute carrier family 36 (proton/amino acid symporter), member 1	SLC36A1	1,32
laminin, alpha 4	LAMA4	1,32
kinesin family member 13A	KIF13A	1,32
SPT3 homolog, SAGA and STAGA complex component	SUPT3H	1,32

coiled-coil domain containing 39	CCDC39	1,32
ectodysplasin A2 receptor	EDA2R	1,32
zinc finger protein 184	ZNF184	1,32
cyclin D1	CCND1	1,32
NECAP endocytosis associated 1	NECAP1	1,32
trace amine associated receptor 2	TAAR2	1,32
pre-mRNA processing factor 4	PRPF4	1,32
zinc finger, DHHC-type containing 3	ZDHHC3	1,32
mitochondrial calcium uniporter	MCU	1,32
homeobox B3; homeobox B4; microRNA 10a	HOXB3; HOXB4; MIR10A	1,32
MAGE family member C2	MAGEC2	1,32
aprataxin and PNKP like factor	APLF	1,32
uncharacterized LOC55338; novel transcript	LOC55338; CTC-242N15.1	1,32
agrin	AGRN	1,32
protein kinase, membrane associated tyrosine/threonine 1	PKMYT1	1,32
chromosome 21 open reading frame 91; C21orf91 overlapping transcript 1	C21orf91; C21orf91-OT1	1,32
ATPase, aminophospholipid transporter, class I, type 8B, member 1	ATP8B1	1,32
spermatogenesis associated 17	SPATA17	1,32
aspartate beta-hydroxylase domain containing 1	ASPHD1	1,32
olfactory receptor, family 2, subfamily K, member 2	OR2K2	1,32
DnaJ (Hsp40) homolog, subfamily C, member 1	DNAJC1	1,32
Transcript Identified by AceView, Entrez Gene ID(s) 5287	PIK3C2B	1,32
ubiquitin conjugating enzyme E2H	UBE2H	1,32
ORAI calcium release-activated calcium modulator 1	ORAI1	1,32
tetraspanin 17	TSPAN17	1,32
zinc finger protein 724, pseudogene	ZNF724P	1,32
TATA box binding protein	TBP	1,32
chromosome 10 open reading frame 107	C10orf107	1,32
hematological and neurological expressed 1-like	HN1L	1,32
nuclear pore associated protein 1	NPAP1	1,32
SLU7 homolog, splicing factor	SLU7	1,32
dysferlin	DYSF	1,32
lipocalin 12	LCN12	1,32
double homeobox 4 like 6	DUX4L6	1,32
double homeobox 4 like 5	DUX4L5	1,32
double homeobox 4 like 1	DUX4L1	1,32
double homeobox 4 like 3	DUX4L3	1,32
double homeobox 4 like 2	DUX4L2	1,32
AF4/FMR2 family, member 2	AFF2	1,32

phospholipid phosphatase 1; ring finger protein 138, E3 ubiquitin protein ligase pseudogene 1	PLPP1; RNF138P1	1,32
atypical chemokine receptor 1 (Duffy blood group)	ACKR1	1,31
DEAD (Asp-Glu-Ala-Asp) box polypeptide 18	DDX18	1,31
glycerophosphodiester phosphodiesterase 1	GDE1	1,31
chromosome 1 open reading frame 194	C1orf194	1,31
Down syndrome cell adhesion molecule like 1	DSCAML1	1,31
triggering receptor expressed on myeloid cells 1	TREM1	1,31
tet methylcytosine dioxygenase 2	TET2	1,31
RIO kinase 1	RIOK1	1,31
zinc finger protein 597	ZNF597	1,31
Transcript Identified by AceView, Entrez Gene ID(s) 54108	CHRAC1	1,31
PAS domain containing 1	PASD1	1,31
ankyrin repeat domain 11	ANKRD11	1,31
ecotropic viral integration site 2A; ecotropic viral integration site 2B	EVI2A; EVI2B	1,31
activating transcription factor 6 beta	ATF6B	1,31
annexin A8	ANXA8	1,31
interleukin 17 receptor E	IL17RE	1,31
proteasome 26S subunit, ATPase 1	PSMC1	1,31
transducin (beta)-like 3	TBL3	1,31
heat shock 70kDa protein 4	HSPA4	1,31
cleft lip and palate associated transmembrane protein 1	CLPTM1	1,31
Memczak2013 ANTISENSE, coding, INTERNAL, intronic best transcript NM_182734	PLCB1	1,31
SYF2 pre-mRNA-splicing factor	SYF2	1,31
COP9 signalosome subunit 3	COPS3	1,31
dachsous cadherin-related 2	DCHS2	1,31
lactase-like	LCTL	1,31
cytochrome P450, family 17, subfamily A, polypeptide 1	CYP17A1	1,31
G protein-coupled receptor 19	GPR19	1,31
F-box and leucine-rich repeat protein 5	FBXL5	1,31
ring finger protein 34, E3 ubiquitin protein ligase	RNF34	1,31
zinc finger protein 816	ZNF816	1,31
syntaxin binding protein 2	STXBP2	1,31
solute carrier family 25 (mitochondrial carrier; adenine nucleotide translocator), member 31	SLC25A31	1,31
fatty acid binding protein 5 (psoriasis-associated)	FABP5	1,31
G antigen 10	GAGE10	1,31
muscle RAS oncogene homolog	MRAS	1,31
zinc finger protein 501 [Source:HGNC Symbol;Acc:HGNC:23717]	ZNF501	1,31
ribosomal protein L22-like 1	RPL22L1	1,31

dishevelled-binding antagonist of beta-catenin 2	DACT2	1,31
cytochrome c oxidase subunit VIIIA (ubiquitous)	COX8A	1,31
PET100 homolog	PET100	1,31
ATPase, Na+/K+ transporting, beta 4 polypeptide	ATP1B4	1,31
mitogen-activated protein kinase 1 interacting protein 1-like	MAPK1IP1L	1,31
dihydrolipoamide S-succinyltransferase (E2 component of 2-oxo-glutarate complex)	DLST	1,31
kinesin family member 21B	KIF21B	1,31
asparaginyl-tRNA synthetase	NARS	1,31
NPR3-like, GATOR1 complex subunit	NPRL3	1,31
cofilin 2 (muscle)	CFL2	1,31
olfactory receptor, family 8, subfamily I, member 2	OR8I2	1,31
v-myb avian myeloblastosis viral oncogene homolog	MYB	1,31
F-box and leucine-rich repeat protein 20	FBXL20	1,31
potassium channel, two pore domain subfamily K, member 3	KCNK3	1,31
zinc finger protein 507	ZNF507	1,31
EML2 antisense RNA 1	EML2-AS1	1,31
family with sequence similarity 175, member B	FAM175B	1,31
zinc finger, SWIM-type containing 1	ZSWIM1	1,31
cilia and flagella associated protein 58	CFAP58	1,31
charged multivesicular body protein 4C	CHMP4C	1,31
YTH domain containing 2	YTHDC2	1,31
forkhead box J2	FOXJ2	1,31
chromosome 17 open reading frame 105	C17orf105	1,31
trinucleotide repeat containing 6A	TNRC6A	1,31
zinc finger protein 350	ZNF350	1,31
solute carrier family 35, member B1	SLC35B1	1,31
TATA box binding protein like 2	TBPL2	1,31
TBC1 domain family, member 26	TBC1D26	1,31
repulsive guidance molecule family member b	RGMB	1,31
iroquois homeobox 5	IRX5	1,31
VPS11, CORVET/HOPS core subunit [Source:HGNC Symbol;Acc:HGNC:14583]	VPS11	1,31
integrin beta 6	ITGB6	1,31
prickle homolog 2	PRICKLE2	1,31
coiled-coil domain containing 93	CCDC93	1,31
CD209 molecule	CD209	1,31
T-box, brain, 1	TBR1	1,31
plexin B2	PLXNB2	1,31
ATPase, H+ transporting, lysosomal 13kDa, V1 subunit G1	ATP6V1G1	1,31
regenerating islet-derived 3 alpha	REG3A	1,31
L antigen family, member 3	LAGE3	1,31

ArfGAP with coiled-coil, ankyrin repeat and PH domains 1	ACAP1	1,31
nuclear receptor corepressor 2	NCOR2	1,31
PRAME family member 6; PRAME family member 5	PRAMEF6; PRAMEF5	1,31
Transcript Identified by AceView, Entrez Gene ID(s) 79875	THSD4	1,31
MAP/microtubule affinity-regulating kinase 2	MARK2	1,31
solute carrier family 1 (high affinity aspartate/glutamate transporter), member 6	SLC1A6	1,31
RIB43A domain with coiled-coils 1	RIBC1	1,31
large tumor suppressor kinase 2	LATS2	1,31
myosin light chain kinase 3	MYLK3	1,31
NGFI-A binding protein 2 (EGR1 binding protein 2)	NAB2	1,31
deleted in azoospermia 4; deleted in azoospermia 1	DAZ4; DAZ1	1,31
cystatin F (leukocystatin)	CST7	1,31
protease, serine, 1 (trypsin 1)	PRSS1	1,31
helicase (DNA) B	HELB	1,31
MYB antisense RNA 1 [Source:HGNC Symbol;Acc:HGNC:37457]; Memczak2013 ANTISENSE, coding, INTERNAL, intronic best transcript NM_005375; putative novel transcript; Transcript Identified by AceView	MYB-AS1; MYB; RP1-32B1.3; rawjer	1,31
MX dynamin-like GTPase 1	MX1	1,31
double C2-like domains, alpha	DOC2A	1,31
FRAS1 related extracellular matrix 3	FREM3	1,31
cutC copper transporter	CUTC	1,31
mitochondrial intermediate peptidase	MIPEP	1,30
NME/NM23 family member 7	NME7	1,30
ribonuclease, RNase A family, 9 (non-active)	RNASE9	1,30
chemokine (C-C motif) receptor 2	CCR2	1,30
serine/threonine/tyrosine kinase 1	STYK1	1,30
oxysterol binding protein-like 11	OSBPL11	1,30
actin binding LIM protein family, member 2	ABLIM2	1,30
chromosome 11 open reading frame 44	C11orf44	1,30
chromosome 12 open reading frame 49	C12orf49	1,30
twist family bHLH transcription factor 1	TWIST1	1,30
proteasome 26S subunit, ATPase 4 [Source:HGNC Symbol;Acc:HGNC:9551]	PSMC4	1,30
metallothionein 4	MT4	1,30
ret finger protein-like 4B	RFPL4B	1,30
MON1 secretory trafficking family member B	MON1B	1,30
brain expressed X-linked 2	BEX2	1,30
lysophosphatidic acid receptor 2	LPAR2	1,30
CDK5 regulatory subunit associated protein 3	CDK5RAP3	1,30

tripartite motif containing 37	TRIM37	1,30
zinc finger, CCCH-type with G-patch domain; Lck interacting transmembrane adaptor 1	ZGPAT; LIME1	1,30
kelch domain containing 8B	KLHDC8B	1,30
estrogen-related receptor gamma	ESRRG	1,30
N-acetyltransferase 1 (arylamine N-acetyltransferase)	NAT1	1,30
potassium channel, inwardly rectifying subfamily J, member 5	KCNJ5	1,30
WD repeat domain 37	WDR37	1,30
zinc finger protein 576	ZNF576	1,30
Dab, reelin signal transducer, homolog 1 (Drosophila)	DAB1	1,30
calcium channel, voltage-dependent, P/Q type, alpha 1A subunit	CACNA1A	1,30
Transcript Identified by AceView, Entrez Gene ID(s) 64478	CSMD1	1,30
inositol 1,3,4,5,6-pentakisphosphate 2-kinase	IPPK	1,30
Memczak2013 ANTISENSE, coding, INTERNAL, intronic best transcript NM_001018	RPS15	1,30
HMG-box transcription factor 1	HBP1	1,30
ceroid-lipofuscinosis, neuronal 5	CLN5	1,30
zinc finger, AN1-type domain 6	ZFAND6	1,30
Myb-like, SWIRM and MPN domains 1	MYSM1	1,30
Homo sapiens family with sequence similarity 231, member A (FAM231A), mRNA.; Homo sapiens family with sequence similarity 231, member C (FAM231C), mRNA.; Protein FAM231A/C [Source:UniProtKB/Swiss-Prot;Acc:A6NEA5]	FAM231A; FAM231C; RP5-1182A14.6	1,30
Transcript Identified by AceView, Entrez Gene ID(s) 339500	ZNF678	1,30
mitogen-activated protein kinase 12	MAPK12	1,30
spermatogenesis associated 21	SPATA21	1,30
zinc finger protein 454	ZNF454	1,30
glutaredoxin, cysteine rich 2	GRXCR2	1,30
RUN and FYVE domain containing 4	RUFY4	1,30
ring finger protein 40, E3 ubiquitin protein ligase	RNF40	1,30
Memczak2013 ANTISENSE, coding, INTERNAL, intronic best transcript NM_001105192	TLE3	1,30
TATA box binding protein (TBP)-associated factor, RNA polymerase I, B, 63kDa	TAF1B	1,30
zinc finger CCCH-type containing 18	ZC3H18	1,30
clusterin associated protein 1	CLUAP1	1,30
solute carrier family 25 (mitochondrial carrier; phosphate carrier), member 25	SLC25A25	1,30
centrosomal protein 41kDa	CEP41	1,30

histocompatibility (minor) 13; malignant T-cell amplified sequence 2, pseudogene	HM13; MCTS2P	1,30
translocase of outer mitochondrial membrane 5 homolog (yeast)	TOMM5	1,30
ISL LIM homeobox 2	ISL2	1,30
apoptosis inhibitor 5	API5	1,30
oleoyl-ACP hydrolase	OLAH	1,30
potassium channel, voltage gated shaker related subfamily A, member 3	KCNA3	1,30
proteasome 26S subunit, non-ATPase 9	PSMD9	1,30
C2CD2-like	C2CD2L	1,30
phosphatidylinositol glycan anchor biosynthesis class C	PIGC	1,30
signal peptide peptidase like 2C	SPPL2C	1,30
XPA binding protein 2	XAB2	1,30
Memczak2013 ANTISENSE, coding, INTERNAL, intronic best transcript NM_018150	RNF220	1,30
leucine rich repeat (in FLII) interacting protein 1	LRRFIP1	1,30
malonyl-CoA-acyl carrier protein transacylase	MCAT	1,30
centrosomal protein 68kDa	CEP68	1,30
defensin, beta 118	DEFB118	1,30
Ras interacting protein 1	RASIP1	1,30
ventricular zone expressed PH domain containing 1	VEPH1	1,30
polymerase (RNA) III (DNA directed) polypeptide F, 39 kDa	POLR3F	1,30
ecto-NOX disulfide-thiol exchanger 1	ENOX1	1,30
catenin (cadherin-associated protein), delta 2	CTNND2	1,30
ubinuclein 2	UBN2	1,30
hippocalcin	HPCA	1,30
phosphorylase, glycogen, muscle	PYGM	1,30
crystallin gamma S	CRYGS	1,30
trafficking protein particle complex 4	TRAPPC4	1,30
F-box protein 28	FBXO28	1,30
Memczak2013 ALT_ACCEPTOR, ALT_DONOR, coding, INTERNAL, intronic best transcript NM_001202557	CD44	1,30
tektin 4 pseudogene 2	TEKT4P2	1,30
Zhang2013 ALT_ACCEPTOR, ALT_DONOR, coding, INTERNAL, intronic, OVERLAPTX best transcript NM_014501	UBE2S	1,30
adaptor-related protein complex 3, beta 2 subunit	AP3B2	1,30
SHOC2 leucine-rich repeat scaffold protein	SHOC2	1,30
nudC nuclear distribution protein	NUDC	1,30
small glutamine-rich tetratricopeptide repeat (TPR)-containing, beta	SGTB	1,30
transmembrane protease, serine 11F	TMPRSS11F	1,30
paired-like homeodomain 1	PITX1	1,30

calmodulin binding transcription activator 1	CAMTA1	1,30
solute carrier family 35, member G4	SLC35G4	1,30
lymphocyte antigen 9	LY9	1,30
trafficking protein particle complex 4	TRAPPC4	1,30
microRNA 4738; H3 histone, family 3B (H3.3B)	MIR4738; H3F3B	1,30
C-type lectin domain family 18, member C	CLEC18C	1,30
cell death-inducing DFFA-like effector a	CIDEA	1,30
poly(A) binding protein, cytoplasmic 4-like	PABPC4L	1,30
chromosome X open reading frame 58	CXorf58	1,30
cytochrome P450, family 1, subfamily A, polypeptide 1	CYP1A1	1,30
zinc finger, DHHC-type containing 1	ZDHHC1	1,30
Transcript Identified by AceView, Entrez Gene ID(s) 23126	POGZ	1,30
chorionic somatomammotropin hormone-like 1	CSHL1	1,30
RIC8 guanine nucleotide exchange factor A; microRNA 6743	RIC8A; MIR6743	1,30
uncharacterized LOC100287225; novel transcript	LOC100287225; RP11-267C16.1	1,29
Memczak2013 ANTISENSE, CDS, coding, INTERNAL best transcript NM_022118	RBM26	1,29
G0/G1 switch 2	G0S2	1,29
calsyntenin 2	CLSTN2	1,29
WAP four-disulfide core domain 6	WFDC6	1,29
N-acetyltransferase 6 (GCN5-related)	NAT6	1,29
RecQ helicase-like 5	RECQL5	1,29
ferritin, light polypeptide	FTL	1,29
cache domain containing 1	CACHD1	1,29
CGRP receptor component	CRCP	1,29
protein phosphatase 1, regulatory subunit 35	PPP1R35	1,29
growth arrest-specific 6	GAS6	1,29
SPEG complex locus	SPEG	1,29
urocortin 2	UCN2	1,29
sodium channel, voltage gated, type VIII alpha subunit	SCN8A	1,29
hyperpolarization activated cyclic nucleotide gated potassium channel 1	HCN1	1,29
stromal cell-derived factor 2	SDF2	1,29
membrane bound O-acyltransferase domain containing 7	MBOAT7	1,29
zinc finger protein 718	ZNF718	1,29
WD repeat domain 27	WDR27	1,29
THUMPD3 antisense RNA 1	THUMPD3-AS1	1,29
KIAA1524	KIAA1524	1,29
mitochondrial ribosomal protein L20	MRPL20	1,29
SRY box 1	SOX1	1,29
tetratricopeptide repeat domain 5	TTC5	1,29

formin 1	FMN1	1,29
transmembrane protein 107; small nucleolar RNA, C/D box 118	TMEM107; SNORD118	1,29
zinc finger protein 300	ZNF300	1,29
testis specific 10	TSGA10	1,29
microtubule-actin crosslinking factor 1; KIAA0754	MACF1; KIAA0754	1,29
alcohol dehydrogenase, iron containing 1; chromosome 8 open reading frame 46	ADHFE1; C8orf46	1,29
PDZ domain containing 3	PDZD3	1,29
cornichon family AMPA receptor auxiliary protein 2	CNIH2	1,29
leucine rich repeat containing 57	LRRC57	1,29
X-ray repair complementing defective repair in Chinese hamster cells 5 (double-strand-break rejoining)	XRCC5	1,29
phosphatidylinositol 3-kinase, catalytic subunit type 3	PIK3C3	1,29
Transcript Identified by AceView; Memczak2013 ANTISENSE, CDS, coding, INTERNAL, intronic, UTR5 best transcript NM_018284	flawmawbo; GBP3	1,29
Jun dimerization protein 2	JDP2	1,29
lipase, family member M	LIPM	1,29
ret finger protein-like 3	RFPL3	1,29
zinc finger CCCH-type containing 12D	ZC3H12D	1,29
GRB10 interacting GYF protein 2	GIGYF2	1,29
casein alpha s1	CSN1S1	1,29
metallophosphoesterase domain containing 2	MPPED2	1,29
SprT-like N-terminal domain	SPRTN	1,29
caspase recruitment domain family, member 9	CARD9	1,29
Transcript Identified by AceView, Entrez Gene ID(s) 4114	MAGEB3	1,29
MAP/microtubule affinity-regulating kinase 1	MARK1	1,29
EH domain containing 1	EHD1	1,29
actin-like 7B	ACTL7B	1,29
slit guidance ligand 3	SLIT3	1,29
keratin 10, type I	KRT10	1,29
SH3 and PX domains 2B	SH3PXD2B	1,29
translocase of inner mitochondrial membrane 50 homolog (S. cerevisiae)	TIMM50	1,29
actin related protein 2/3 complex subunit 5	ARPC5	1,29
small integral membrane protein 12	SMIM12	1,29
collagen, type VI, alpha 6	COL6A6	1,29
dedicator of cytokinesis 1	DOCK1	1,29
tetratricopeptide repeat domain 26	TTC26	1,29
anillin actin binding protein	ANLN	1,29
GSG1-like 2	GSG1L2	1,29
down-regulator of transcription 1	DR1	1,29

fibroblast growth factor receptor substrate 3	FRS3	1,29
CASP8 and FADD like apoptosis regulator	CFLAR	1,29
titin-cap	TCAP	1,29
solute carrier family 22 (organic anion transporter), member 6	SLC22A6	1,29
tumor suppressing subtransferable candidate 1	TSSC1	1,29
ribosomal protein S6 kinase, 90kDa, polypeptide 1	RPS6KA1	1,29
protein kinase D3	PRKD3	1,29
SH3 and cysteine rich domain 3	STAC3	1,29
REX1, RNA exonuclease 1 homolog-like 11, pseudogene	REXO1L11P	1,29
aminoacyl tRNA synthetase complex-interacting multifunctional protein 1	AIMP1	1,29
3-hydroxyacyl-CoA dehydratase 1	HACD1	1,29
cadherin-related family member 4	CDHR4	1,29
calsequestrin 2 (cardiac muscle)	CASQ2	1,29
solute carrier family 29 (equilibrative nucleoside transporter), member 3	SLC29A3	1,29
alpha kinase 3	ALPK3	1,29
discs, large (Drosophila) homolog-associated protein 3	DLGAP3	1,29
fatty acid binding protein 6, ileal	FABP6	1,29
Memczak2013 ANTISENSE, coding, INTERNAL, intronic best transcript NM_173464	L3MBTL4	1,29
progestin and adipoQ receptor family member VI	PAQR6	1,29
maestro heat-like repeat family member 2A	MROH2A	1,29
keratin associated protein 4-11	KRTAP4-11	1,29
eukaryotic translation initiation factor 2B, subunit 1 alpha, 26kDa	EIF2B1	1,29
SRY box 2	SOX2	1,29
SNU13 homolog, small nuclear ribonucleoprotein (U4/U6.U5)	SNU13	1,29
disrupted in schizophrenia 1	DISC1	1,29
dispatched homolog 1 (Drosophila)	DISP1	1,29
PHD finger protein 12	PHF12	1,29
tumor protein D52-like 3	TPD52L3	1,29
heat shock transcription factor family, X-linked 2; heat shock transcription factor family, X-linked 1	HSFX2; HSFX1	1,29
xeroderma pigmentosum, complementation group C	XPC	1,29
paired box 5	PAX5	1,29
SUMO1/sentrin specific peptidase 7	SENP7	1,29
splA/ryanodine receptor domain and SOCS box containing 2	SPSB2	1,29
DMRT-like family C1B; DMRT-like family C1	DMRTC1B; DMRTC1	1,29
golgin A6 family-like 5, pseudogene; golgin A6 family-like 17, pseudogene	GOLGA6L5P; GOLGA6L17P	1,28

cytosolic thiouridylase subunit 2 homolog (S. pombe)	CTU2	1,28
Memczak2013 ANTISENSE, coding, INTERNAL, intronic best transcript NM_024830	LPCAT1	1,28
tripartite motif family like 2	TRIML2	1,28
SUMO1 activating enzyme subunit 1	SAE1	1,28
mitogen-activated protein kinase-activated protein kinase 3	MAPKAPK3	1,28
sodium channel, non voltage gated 1 gamma subunit	SCNN1G	1,28
fibrous sheath interacting protein 2	FSIP2	1,28
transmembrane and coiled-coil domains 5A	TMCO5A	1,28
ENTH domain containing 2	ENTHD2	1,28
HECT and RLD domain containing E3 ubiquitin protein ligase 3	HERC3	1,28
hemicentin 1	HMCN1	1,28
transmembrane protein 262	TMEM262	1,28
egl-9 family hypoxia-inducible factor 3	EGLN3	1,28
chimerin 1	CHN1	1,28
calcium/calmodulin-dependent protein kinase II beta	CAMK2B	1,28
protein tyrosine phosphatase, receptor type, Q	PTPRQ	1,28
v-rel avian reticuloendotheliosis viral oncogene homolog A	RELA	1,28
oral-facial-digital syndrome 1	OFD1	1,28
zinc finger protein 30	ZNF30	1,28
keratin 6A, type II	KRT6A	1,28
wingless-type MMTV integration site family, member 5A	WNT5A	1,28
Memczak2013 ANTISENSE, CDS, coding, INTERNAL, intronic, UTR3 best transcript NM_203305	FAM102A	1,28
cyclin L2	CCNL2	1,28
poly(rC) binding protein 3	PCBP3	1,28
PITPNM family member 3	PITPNM3	1,28
Transcript Identified by AceView, Entrez Gene ID(s) 89910	UBE3B	1,28
ATP5S-like	ATP5SL	1,28
acyl-CoA synthetase medium-chain family member 1	ACSM1	1,28
WD repeat domain 74	WDR74	1,28
corticotropin releasing hormone receptor 2	CRHR2	1,28
Transcript Identified by AceView, Entrez Gene ID(s) 54870	QRICH1	1,28
ubiquitin specific peptidase 17-like family member 1	USP17L1	1,28
leucine zipper protein 6; myotrophin	LUZP6; MTPN	1,28
sialidase 3 (membrane sialidase)	NEU3	1,28
megakaryocyte-associated tyrosine kinase	MATK	1,28
arylacetamide deacetylase	AADAC	1,28
microtubule associated serine/threonine kinase 2	MAST2	1,28

zinc finger with KRAB and SCAN domains 3	ZKSCAN3	1,28
EF-hand domain family member D1	EFHD1	1,28
transmembrane protein 50B	TMEM50B	1,28
S-phase kinase-associated protein 1	SKP1	1,28
diablo, IAP-binding mitochondrial protein	DIABLO	1,28
folylpolyglutamate synthase	FPGS	1,28
Transcript Identified by AceView, Entrez Gene ID(s) 3030	HADHA	1,28
caseinolytic mitochondrial matrix peptidase proteolytic subunit	CLPP	1,28
defensin, beta 129	DEFB129	1,28
serpin peptidase inhibitor, clade B (ovalbumin), member 5	SERPINB5	1,28
serum response factor binding protein 1	SRFBP1	1,28
transmembrane protein 231	TMEM231	1,28
polymerase (RNA) II (DNA directed) polypeptide C, 33kDa	POLR2C	1,28
glycerophosphodiester phosphodiesterase domain containing 3	GDPD3	1,28
Rap guanine nucleotide exchange factor 2	RAPGEF2	1,28
Rho guanine nucleotide exchange factor 3	ARHGEF3	1,28
G protein pathway suppressor 2	GPS2	1,28
cytochrome c oxidase subunit VIIa polypeptide 2 (liver)	COX7A2	1,28
serpin peptidase inhibitor, clade B (ovalbumin), member 1	SERPINB1	1,28
INO80 complex subunit E	INO80E	1,28
Homo sapiens proline dehydrogenase (oxidase) 1 (PRODH), transcript variant 2, mRNA.; Homo sapiens proline dehydrogenase (oxidase) 1 (PRODH), transcript variant 1, mRNA.; proline dehydrogenase 1, mitochondrial; Homo sapiens proline dehydrogenase (oxidase) 1, mRNA (cDNA clone MGC:148079 IMAGE:40108133), complete cds.	PRODH; LOC102724788; AC007325.2	1,28
hes family bHLH transcription factor 7	HES7	1,28
transmembrane and coiled-coil domains 1	TMCO1	1,28
stabilizer of axonemal microtubules 1	SAXO1	1,28
ral guanine nucleotide dissociation stimulator	RALGDS	1,28
E74-like factor 5 (ets domain transcription factor)	ELF5	1,28
lysyl-tRNA synthetase	KARS	1,28
family with sequence similarity 92, member B	FAM92B	1,28
nuclear pore complex interacting protein family, member B15	NPIPB15	1,28
endoplasmic reticulum to nucleus signaling 2	ERN2	1,28
eukaryotic translation initiation factor 3, subunit H	EIF3H	1,28
structural maintenance of chromosomes 2	SMC2	1,28

ribosomal modification protein rimK-like family member B	RIMKLB	1,28
thrombospondin type 1 domain containing 7A	THSD7A	1,28
MYC associated factor X	MAX	1,28
Jeck2013 ANTISENSE, CDS, coding, INTERNAL, intronic, OVCODE, OVEXON best transcript NM_000089	COL1A2	1,28
immunoglobulin-like domain containing receptor 2	ILDR2	1,28
inhibin beta B	INHBB	1,28
SUMO/sentrin peptidase family member, NEDD8 specific	SENP8	1,28
MAP7 domain containing 3	MAP7D3	1,28
Transcript Identified by AceView, Entrez Gene ID(s) 114794	ELFN2	1,28
ATP binding cassette subfamily A member 3	ABCA3	1,28
HEAT repeat containing 1	HEATR1	1,28
ribosome biogenesis regulator homolog	RRS1	1,28
adhesion G protein-coupled receptor G5	ADGRG5	1,28
WNT1 inducible signaling pathway protein 3	WISP3	1,28
ubiquinol-cytochrome c reductase complex assembly factor 2	UQCC2	1,28
general transcription factor IIA 2	GTF2A2	1,28
mast cell-expressed membrane protein 1	MCEMP1	1,28
serpin peptidase inhibitor, clade B (ovalbumin), member 10	SERPINB10	1,28
BCL2-associated athanogene 5	BAG5	1,28
olfactory receptor, family 10, subfamily V, member 1	OR10V1	1,28
Rho guanine nucleotide exchange factor 1	ARHGEF1	1,28
protein kinase D2	PRKD2	1,28
proteasome 26S subunit, non-ATPase 1	PSMD1	1,28
transmembrane protein 120B	TMEM120B	1,28
TRIM39-RPP21 readthrough	TRIM39-RPP21	1,28
UPF2 regulator of nonsense transcripts homolog (yeast)	UPF2	1,28
transmembrane protein 158 (gene/pseudogene)	TMEM158	1,28
submaxillary gland androgen regulated protein 3A	SMR3A	1,28
coiled-coil domain containing 73	CCDC73	1,28
troponin T type 3 (skeletal, fast)	TNNT3	1,28
proteasome activator subunit 3	PSME3	1,28
sialic acid binding Ig-like lectin 15	SIGLEC15	1,28
zinc finger, DHHC-type containing 12	ZDHHC12	1,27
choroideremia (Rab escort protein 1)	CHM	1,27
carboxypeptidase M	CPM	1,27
family with sequence similarity 129, member B	FAM129B	1,27
protease, serine 38	PRSS38	1,27
NudC domain containing 1	NUDCD1	1,27

general transcription factor IIH subunit 2; general transcription factor IIH subunit 2B (pseudogene)	GTF2H2; GTF2H2B	1,27
ZFP62 zinc finger protein	ZFP62	1,27
splicing factor 3a subunit 2	SF3A2	1,27
BMX non-receptor tyrosine kinase	BMX	1,27
collagen beta(1-O)galactosyltransferase 2	COLGALT2	1,27
X-ray repair complementing defective repair in Chinese hamster cells 3	XRCC3	1,27
ring finger protein 2	RNF2	1,27
family with sequence similarity 122A	FAM122A	1,27
flavin containing monooxygenase 1	FMO1	1,27
glycosylphosphatidylinositol anchor attachment 1	GPAA1	1,27
keratin 34, type I	KRT34	1,27
peroxisomal biogenesis factor 1	PEX1	1,27
chemokine (C-C motif) ligand 1	CCL1	1,27
protein phosphatase 1, regulatory subunit 3B	PPP1R3B	1,27
histatin 1	HTN1	1,27
polo-like kinase 4	PLK4	1,27
kinesin family member 9	KIF9	1,27
cytochrome P450, family 2, subfamily D, polypeptide 6	CYP2D6	1,27
caudal type homeobox 1	CDX1	1,27
complexin 3	CPLX3	1,27
tetratricopeptide repeat, ankyrin repeat and coiled-coil containing 1	TANC1	1,27
myeloid-associated differentiation marker	MYADM	1,27
sacsin molecular chaperone	SACS	1,27
bolA family member 1	BOLA1	1,27
dual specificity tyrosine-(Y)-phosphorylation regulated kinase 1A	DYRK1A	1,27
E2F transcription factor 6	E2F6	1,27
Jeck2013 ANTISENSE, CDS, coding, INTERNAL, intronic, OVCODE, OVEXON best transcript NM_012242	DKK1	1,27
stomatin (EPB72)-like 1	STOML1	1,27
glutathione reductase	GSR	1,27
beta-1,3-glucuronyltransferase 1	B3GAT1	1,27
T brachyury transcription factor	T	1,27
DnaJ (Hsp40) homolog, subfamily B, member 12	DNAJB12	1,27
zinc finger protein 345	ZNF345	1,27
ADP-ribosyltransferase 1	ART1	1,27
ERI1 exoribonuclease family member 2	ERI2	1,27
PQ loop repeat containing 2-like	PQLC2L	1,27
gamma-glutamyltransferase 2	GGT2	1,27
ADP-ribosylation factor like GTPase 2 binding protein	ARL2BP	1,27
chromosome 9 open reading frame 84	C9orf84	1,27

apolipoprotein B mRNA editing enzyme, catalytic polypeptide-like 3H	APOBEC3H	1,27
interferon, alpha-inducible protein 27-like 1	IFI27L1	1,27
heparan sulfate (glucosamine) 3-O-sulfotransferase 4	HS3ST4	1,27
neugrin, neurite outgrowth associated; tubulin tyrosine ligase-like family member 13, pseudogene	NGRN; TTLL13P	1,27
MDM2 proto-oncogene, E3 ubiquitin protein ligase	MDM2	1,27
GRB2-binding adaptor protein, transmembrane	GAPT	1,27
discs, large (Drosophila) homolog-associated protein 2	DLGAP2	1,27
actin, alpha 1, skeletal muscle	ACTA1	1,27
bromodomain containing 4	BRD4	1,27
family with sequence similarity 153, member B	FAM153B	1,27
benzodiazepine receptor (peripheral) associated protein 1	BZRAP1	1,27
selenoprotein V; selenoprotein V [Source:EntrezGene;Acc:348303]; Transcript Identified by AceView, Entrez Gene ID(s) 348303, RefSeq ID(s) NM_182704	SELV	1,27
uroporphyrinogen decarboxylase	UROD	1,27
poly(ADP-ribose) polymerase family member 11	PARP11	1,27
Rho GTPase activating protein 23	ARHGAP23	1,27
polyamine oxidase (exo-N4-amino)	PAOX	1,27
mitochondrial ribosomal protein S21	MRPS21	1,27
RNA binding motif protein 27	RBM27	1,27
Memczak2013 ANTISENSE, coding, INTERNAL, intronic best transcript NM_001195144	ANKRD44	1,27
piwi-like RNA-mediated gene silencing 4	PIWIL4	1,27
de-etiolated homolog 1 (Arabidopsis)	DET1	1,27
zinc finger homeobox 2	ZFHX2	1,27
olfactory receptor, family 8, subfamily K, member 3 (gene/pseudogene)	OR8K3	1,27
chromosome 1 open reading frame 27	C1orf27	1,27
Transcript Identified by AceView, Entrez Gene ID(s) 49855	SCAPER	1,27
5-nucleotidase, cytosolic II	NT5C2	1,27
translocase of inner mitochondrial membrane 23 homolog (yeast)	TIMM23	1,27
G-protein signaling modulator 1	GPSM1	1,27
tRNA methyltransferase 12 homolog (S. cerevisiae)	TRMT12	1,27
Sec23 homolog B, COPII coat complex component	SEC23B	1,27
protein phosphatase 1, regulatory subunit 17	PPP1R17	1,27
sema domain, immunoglobulin domain (Ig), short basic domain, secreted, (semaphorin) 3B; microRNA 6872	SEMA3B; MIR6872	1,27
FK506 binding protein 4	FKBP4	1,27
contactin 1	CNTN1	1,27

Memczak2013 ANTISENSE, CDS, coding, INTERNAL best transcript NM_001020819	MYADM	1,27
lysine (K)-specific demethylase 2A	KDM2A	1,27
interferon regulatory factor 3	IRF3	1,27
synovial sarcoma, X breakpoint 6 (pseudogene)	SSX6	1,27
OTU deubiquitinase 4	OTUD4	1,27
Zhang2013 ALT_ACCEPTOR, ALT_DONOR, coding, INTERNAL, intronic best transcript NM_002473	MYH9	1,27
family with sequence similarity 179, member B	FAM179B	1,27
yippee like 4	YPEL4	1,27
phosphatidylinositol 4-kinase, catalytic, beta	PI4KB	1,27
family with sequence similarity 221, member A	FAM221A	1,27
TBC1 domain family, member 27	TBC1D27	1,27
gamma-glutamyltransferase light chain 2	GGTLC2	1,27
thioredoxin-like 1	TXNL1	1,27
keratin 18 pseudogene 55	KRT18P55	1,27
prion protein 2 (dublet)	PRND	1,27
protein interacting with PRKCA 1	PICK1	1,27
semenogelin I	SEMG1	1,27
protection of telomeres 1	POT1	1,27
cholinergic receptor, nicotinic delta	CHRND	1,27
keratin associated protein 10-1	KRTAP10-1	1,27
leucine rich repeat containing 46	LRRC46	1,27
sideroflexin 3	SFXN3	1,27
LSM1 homolog, mRNA degradation associated	LSM1	1,27
peptidylprolyl isomerase F	PPIF	1,27
V-set and transmembrane domain containing 4	VSTM4	1,27
tubulin, gamma complex associated protein 6	TUBGCP6	1,27
tumor necrosis factor, alpha-induced protein 1 (endothelial)	TNFAIP1	1,27
chromosome 3 open reading frame 67	C3orf67	1,26
Memczak2013 ANTISENSE, coding, INTERNAL, intronic best transcript NM_001001660	LYRM5	1,26
Rho GTPase activating protein 17	ARHGAP17	1,26
ankyrin repeat domain 44	ANKRD44	1,26
family with sequence similarity 71, member E1	FAM71E1	1,26
membrane-spanning 4-domains, subfamily A, member 10	MS4A10	1,26
unc-51 like autophagy activating kinase 1	ULK1	1,26
envoplakin	EVPL	1,26
diphthamide biosynthesis 1; ovarian tumor suppressor candidate 2	DPH1; OVCA2	1,26
zinc finger protein 587	ZNF587	1,26
hedgehog acyltransferase	HHAT	1,26

eukaryotic translation initiation factor 2B, subunit 3 gamma, 58kDa	EIF2B3	1,26
chromosome 19 open reading frame 81	C19orf81	1,26
tyrosine 3-monooxygenase/tryptophan 5-monooxygenase activation protein, theta	YWHAQ	1,26
integrator complex subunit 7	INTS7	1,26
cytidine monophosphate N-acetylneuraminic acid synthetase	CMAS	1,26
cholinergic receptor, nicotinic alpha 3	CHRNA3	1,26
STIP1 homology and U-box containing protein 1, E3 ubiquitin protein ligase	STUB1	1,26
transcription elongation factor B (SIII), polypeptide 3 (110kDa, elongin A)	TCEB3	1,26
adaptor-related protein complex 1, sigma 2 subunit pseudogene	LOC653653; AC025048.1	1,26
C-type lectin domain family 1, member A	CLEC1A	1,26
surfeit 2 [Source:HGNC Symbol;Acc:HGNC:11475]	SURF2	1,26
olfactory receptor, family 51, subfamily G, member 1 (gene/pseudogene)	OR51G1	1,26
glycoprotein (transmembrane) nmb	GPNMB	1,26
TAF6 RNA polymerase II, TATA box binding protein (TBP)-associated factor, 80kDa	TAF6	1,26
chromodomain helicase DNA binding protein 1	CHD1	1,26
V-set and transmembrane domain containing 2 like	VSTM2L	1,26
mitochondria localized glutamic acid rich protein	MGARP	1,26
polymerase (RNA) II (DNA directed) polypeptide J, 13.3kDa	POLR2J	1,26
ribonucleic acid export 1	RAE1	1,26
Transcript Identified by AceView, Entrez Gene ID(s) 5783	PTPN13	1,26
cytokine inducible SH2-containing protein	CISH	1,26
neural precursor cell expressed, developmentally down-regulated 4, E3 ubiquitin protein ligase	NEDD4	1,26
G-patch domain and ankyrin repeats 1	GPANK1	1,26
WD repeat domain 76	WDR76	1,26
WD repeat domain 25	WDR25	1,26
polymerase (DNA directed), theta	POLQ	1,26
single-minded family bHLH transcription factor 1	SIM1	1,26
zinc finger protein 319	ZNF319	1,26
tandem C2 domains, nuclear	TC2N	1,26
deiodinase, iodothyronine, type II	DIO2	1,26
RAB5B, member RAS oncogene family	RAB5B	1,26
BBSome interacting protein 1	BBIP1	1,26
spondin 1, extracellular matrix protein	SPON1	1,26
vacuole membrane protein 1; microRNA 21	VMP1; MIR21	1,26

LDL receptor related protein 1B	LRP1B	1,26
GSG1-like	GSG1L	1,26
MT-RNR2-like 2; MT-RNR2-like 12	MTRNR2L2; MTRNR2L12	1,26
serine incorporator 5	SERINC5	1,26
family with sequence similarity 86, member C1	FAM86C1	1,26
mitochondrial transcription termination factor 1	MTERF1	1,26
transmembrane protein 132A	TMEM132A	1,26
ubiquitin domain containing 1	UBTD1	1,26
zinc finger, matrin-type 2	ZMAT2	1,26
centromere protein J	CENPJ	1,26
tripartite motif containing 68	TRIM68	1,26
olfactory receptor, family 13, subfamily C, member 3	OR13C3	1,26
pleckstrin homology-like domain, family A, member 1	PHLDA1	1,26
RAP1B, member of RAS oncogene family	RAP1B	1,26
small nuclear ribonucleoprotein, U4/U6.U5 27kDa subunit	SNRNP27	1,26
uncharacterized LOC100130449; novel transcript, antisense to GPC1; Transcript Identified by AceView, Entrez Gene ID(s) 100130449; uncharacterized LOC100130449 [Source:EntrezGene;Acc:100130449]	PP14571; AC110619.2	1,26
Transcript Identified by AceView, Entrez Gene ID(s) 6579	SLCO1A2	1,26
toll-interleukin 1 receptor (TIR) domain containing adaptor protein	TIRAP	1,26
SCL/TAL1 interrupting locus	STIL	1,26
peptidylprolyl isomerase A (cyclophilin A)-like 4A	PPIAL4A	1,26
Memczak2013 ANTISENSE, coding, INTERNAL, UTR3 best transcript NM_016815	GYPC	1,26
PRAME family member 5	PRAMEF5	1,26
leucine rich repeat containing 66	LRRC66	1,26
galanin receptor 2	GALR2	1,26
centrosomal protein 70kDa	CEP70	1,26
dystrophin related protein 2	DRP2	1,26
mediator complex subunit 11	MED11	1,26
thymidine kinase 1, soluble	TK1	1,26
phosphatase domain containing, paladin 1	PALD1	1,26
v-maf avian musculoaponeurotic fibrosarcoma oncogene homolog B	MAFB	1,26
prolyl 3-hydroxylase 2	P3H2	1,26
trafficking protein particle complex 10	TRAPPC10	1,26
arylsulfatase F	ARSF	1,26
vacuolar protein sorting 28 homolog (S. cerevisiae)	VPS28	1,26
cathepsin G	CTSG	1,26
olfactory receptor, family 52, subfamily N, member 2	OR52N2	1,26

transcriptional adaptor 2A	TADA2A	1,26
mucin 21, cell surface associated	MUC21	1,26
gastrulation brain homeobox 2	GBX2	1,26
pleckstrin homology domain containing, family F (with FYVE domain) member 2	PLEKHF2	1,26
protein phosphatase 3, catalytic subunit, alpha isozyme	PPP3CA	1,26
prefoldin subunit 4	PFDN4	1,26
leiomodin 2 (cardiac)	LMOD2	1,26
MAGE family member C3	MAGEC3	1,26
phosphodiesterase 7B	PDE7B	1,26
protein kinase, cAMP-dependent, regulatory, type I, beta	PRKAR1B	1,26
coiled-coil domain containing 58	CCDC58	1,26
olfactory receptor, family 8, subfamily D, member 2 (gene/pseudogene)	OR8D2	1,26
syncoilin, intermediate filament protein	SYNC	1,26
suppression of tumorigenicity 5	ST5	1,26
tryptase alpha/beta 1; tryptase beta 2 (gene/pseudogene)	TPSAB1; TPSB2	1,26
osteomodulin	OMD	1,26
chemokine (C-C motif) receptor 5 (gene/pseudogene)	CCR5	1,26
mitochondrial ribosomal protein S2	MRPS2	1,26
nucleoporin 35kDa	NUP35	1,26
maternal embryonic leucine zipper kinase	MELK	1,26
POU class 4 homeobox 1	POU4F1	1,26
LY6/PLAUR domain containing 5	LYPD5	1,26
glial cells missing homolog 2 (Drosophila)	GCM2	1,26
lipocalin 8	LCN8	1,26
one cut homeobox 3	ONECUT3	1,26
olfactory receptor, family 52, subfamily E, member 4	OR52E4	1,26
AKT interacting protein	AKTIP	1,26
cyclin-dependent kinase-like 3	CDKL3	1,26
ankyrin repeat domain 29	ANKRD29	1,26
T-box 1	TBX1	1,26
coiled-coil domain containing 90B	CCDC90B	1,26
amyloid beta (A4) precursor-like protein 2	APLP2	1,26
cysteine-serine-rich nuclear protein 1	CSRNP1	1,26
charged multivesicular body protein 7	CHMP7	1,26
SLAM family member 9	SLAMF9	1,26
methyltransferase like 8	METTL8	1,26
serine threonine kinase 39	STK39	1,26
protein tyrosine phosphatase, receptor type, C	PTPRC	1,26
torsin family 4, member A	TOR4A	1,26
collagen, type IX, alpha 1	COL9A1	1,26
ClpB homolog, mitochondrial AAA ATPase chaperonin	CLPB	1,26

sodium channel, non voltage gated 1 alpha subunit	SCNN1A	1,26
calcium/calmodulin-dependent protein kinase II delta	CAMK2D	1,26
pleckstrin homology domain containing, family G (with RhoGef domain) member 4B	PLEKHG4B	1,26
KIAA1324-like	KIAA1324L	1,26
Jeck2013 ALT_ACCEPTOR, ALT_DONOR, coding, INTERNAL, intronic best transcript NM_133509	RAD51B	1,25
C20orf166 antisense RNA 1	C20orf166-AS1	1,25
actin related protein 2/3 complex subunit 3	ARPC3	1,25
syntaxin 4	STX4	1,25
sorting nexin 2	SNX2	1,25
oxysterol binding protein-like 2	OSBPL2	1,25
paraneoplastic Ma antigen family member 5	PNMA5	1,25
rhomboid domain containing 3	RHBDD3	1,25
glycine receptor alpha 3	GLRA3	1,25
UFM1-specific peptidase 2	UFSP2	1,25
pyruvate kinase, muscle	PKM	1,25
LIM homeobox transcription factor 1, beta	LMX1B	1,25
anoctamin 9	ANO9	1,25
nuclear pore complex interacting protein family, member B3	NPIPB3	1,25
chromosome 9 open reading frame 47; sphingosine-1-phosphate receptor 3	C9orf47; S1PR3	1,25
gastrokine 1	GKN1	1,25
solute carrier family 7 (amino acid transporter light chain, y+L system), member 6	SLC7A6	1,25
exonuclease 3-5 domain containing 2	EXD2	1,25
lymphocyte-activation gene 3	LAG3	1,25
paired box 4	PAX4	1,25
transmembrane and ubiquitin-like domain containing 2	TMUB2	1,25
armadillo repeat gene deleted in velocardiofacial syndrome	ARVCF	1,25
katanin p60 (ATPase containing) subunit A 1	KATNA1	1,25
Purkinje cell protein 4 like 1	PCP4L1	1,25
microfibrillar associated protein 5	MFAP5	1,25
RCAN family member 3	RCAN3	1,25
galanin receptor 3	GALR3	1,25
neuroblastoma breakpoint family, member 1	NBPF1	1,25
NADH dehydrogenase (ubiquinone) 1 beta subcomplex, 2, 8kDa	NDUFB2	1,25
Memczak2013 ANTISENSE, coding, INTERNAL, intronic best transcript NM_014754	PTDSS1	1,25
5-phosphohydroxy-L-lysine phospho-lyase	PHYKPL	1,25
fms-related tyrosine kinase 4	FLT4	1,25
zinc finger protein 384	ZNF384	1,25

FERM and PDZ domain containing 4	FRMPD4	1,25
Ras association (RalGDS/AF-6) domain family member 3	RASSF3	1,25
NFAT activating protein with ITAM motif 1	NFAM1	1,25
MDM2 binding protein	MTBP	1,25
tweety family member 2	TTYH2	1,25
cysteinyl leukotriene receptor 2	CYSLTR2	1,25
piggyBac transposable element derived 1	PGBD1	1,25
WW domain binding protein 5	WBP5	1,25
polypeptide N-acetylgalactosaminyltransferase 6	GALNT6	1,25
mitochondrial fission factor	MFF	1,25
splicing factor 3b subunit 2	SF3B2	1,25
radical S-adenosyl methionine domain containing 2	RSAD2	1,25
eukaryotic translation initiation factor 6	EIF6	1,25
nuclear receptor subfamily 1, group H, member 2	NR1H2	1,25
Transcript Identified by AceView, Entrez Gene ID(s) 9215	LARGE	1,25
tRNA methyltransferase 11-2 homolog (S. cerevisiae)	TRMT112	1,25
ataxin 2-like	ATXN2L	1,25
sphingomyelin synthase 2	SGMS2	1,25
prolyl 4-hydroxylase, alpha polypeptide III	P4HA3	1,25
proteasomal ATPase-associated factor 1	PAAF1	1,25
zinc finger protein 732	ZNF732	1,25
vacuolar protein sorting 52 homolog (S. cerevisiae)	VPS52	1,25
RAB interacting factor	RABIF	1,25
ankyrin repeat and SOCS box containing 14	ASB14	1,25
PRAME family member 4	PRAMEF4	1,25
POU class 2 homeobox 3	POU2F3	1,25
RFX family member 8, lacking RFX DNA binding domain	RFX8	1,25
cell cycle progression 1; microRNA 628	CCPG1; MIR628	1,25
tripartite motif containing 42	TRIM42	1,25
ectonucleoside triphosphate diphosphohydrolase 8	ENTPD8	1,25
glutathione S-transferase omega 2	GSTO2	1,25
chromosome X open reading frame 49B; chromosome X open reading frame 49	CXorf49B; CXorf49	1,25
chromosome 9 open reading frame 129	C9orf129	1,25
DEAH (Asp-Glu-Ala-His) box polypeptide 32	DHX32	1,25
Transcript Identified by AceView, Entrez Gene ID(s) 65249	ZSWIM4	1,25
ribosomal protein S6 kinase, 90kDa, polypeptide 1	RPS6KA1	1,25
pellino E3 ubiquitin protein ligase family member 2	PELI2	1,25
testis-specific serine kinase substrate	TSKS	1,25
protein phosphatase 2, regulatory subunit B, beta	PPP2R2B	1,25
purinergic receptor P2X, ligand gated ion channel, 4	P2RX4	1,25
RNA binding motif protein 4	RBM4	1,25

paired box 1	PAX1	1,25
glycosyltransferase 8 domain containing 2	GLT8D2	1,25
phosphatidylinositol-4-phosphate 5-kinase, type I, alpha	PIP5K1A	1,25
transcription factor AP-2 epsilon (activating enhancer binding protein 2 epsilon)	TFAP2E	1,25
BarH-like homeobox 2	BARHL2	1,25
ATPase, Ca++ transporting, ubiquitous	ATP2A3	1,25
Memczak2013 ANTISENSE, CDS, coding, INTERNAL best transcript NM_004145	MYO9B	1,25
chromosome 3 open reading frame 56	C3orf56	1,25
ladybird homeobox 2	LBX2	1,25
galactose-3-O-sulfotransferase 3	GAL3ST3	1,25
KIAA1107	KIAA1107	1,25
ring finger protein 111	RNF111	1,25
myeloid/lymphoid or mixed-lineage leukemia; translocated to, 1	MLLT1	1,25
DNA meiotic recombinase 1	DMC1	1,25
diacylglycerol kinase, delta 130kDa	DGKD	1,25
Eukaryotic translation initiation factor 3 subunit F [Source:UniProtKB/Swiss-Prot;Acc:O00303]	EIF3F	1,25
ubiquitin-conjugating enzyme E2Q family member 1	UBE2Q1	1,25
spinster homolog 3 (Drosophila)	SPNS3	1,25
coiled-coil domain containing 53	CCDC53	1,25
ankyrin repeat and BTB (POZ) domain containing 1	ABTB1	1,25
sialidase 1 (lysosomal sialidase)	NEU1	1,25
S-antigen; retina and pineal gland (arrestin)	SAG	1,25
glycerol-3-phosphate acyltransferase 2, mitochondrial	GPAT2	1,25
calcium channel, voltage-dependent, gamma subunit 5	CACNG5	1,25
zinc finger protein 777	ZNF777	1,25
F-box protein 10	FBXO10	1,25
pyruvate dehydrogenase kinase, isozyme 4	PDK4	1,25
Transcript Identified by AceView, Entrez Gene ID(s) 93190	C1orf158	1,25
retrotransposon-like 1	RTL1	1,25
jumonji domain containing 7; phospholipase A2, group IVB (cytosolic); JMJD7-PLA2G4B readthrough	JMJD7; PLA2G4B; JMJD7-PLA2G4B	1,25
timeless circadian clock	TIMELESS	1,25
lectin, galactoside-binding, soluble, 14	LGALS14	1,25
Rho GTPase activating protein 4	ARHGAP4	1,25
pre-mRNA processing factor 18	PRPF18	1,25
cytochrome P450, family 2, subfamily D, polypeptide 6	CYP2D6	1,25
chromosome 3 open reading frame 38	C3orf38	1,25
Memczak2013 ANTISENSE, coding, INTERNAL, intronic best transcript NM_001020819; novel transcript, antisense to MYADM	AC008753.6; MYADM	1,25

mitochondrial ribosome recycling factor	MRRF	1,25
differentially expressed in FDCP 8 homolog (mouse)	DEF8	1,25
zinc finger protein 836	ZNF836	1,25
copine II	CPNE2	1,25
IQ motif containing F3	IQCF3	1,25
RAS p21 protein activator 3	RASA3	1,25
transketolase-like 1	TKTL1	1,25
transmembrane protein 17	TMEM17	1,25
membrane-spanning 4-domains, subfamily A, member 6A	MS4A6A	1,25
coiled-coil domain containing 114	CCDC114	1,24
DEAH (Asp-Glu-Ala-His) box polypeptide 38	DHX38	1,24
endothelial PAS domain protein 1	EPAS1	1,24
myeloid leukemia factor 2	MLF2	1,24
ChaC, cation transport regulator homolog 2 (E. coli)	CHAC2	1,24
WD repeat domain 34	WDR34	1,24
interferon regulatory factor 8	IRF8	1,24
follicular dendritic cell secreted protein	FDCSP	1,24
MORN repeat containing 5	MORN5	1,24
X-linked Kx blood group related 5	XKR5	1,24
nuclear pore complex interacting protein family, member B4	NPIPB4	1,24
NADH dehydrogenase (ubiquinone) complex I, assembly factor 3	NDUFAF3	1,24
olfactory receptor, family 9, subfamily A, member 2	OR9A2	1,24
G protein-coupled receptor, class C, group 6, member A	GPRC6A	1,24
spastic paraplegia 11 (autosomal recessive)	SPG11	1,24
ceroid-lipofuscinosis, neuronal 8	CLN8	1,24
T-cell lymphoma invasion and metastasis 2	TIAM2	1,24
zinc finger protein 835	ZNF835	1,24
SLX1 homolog A, structure-specific endonuclease subunit; SLX1 homolog B, structure-specific endonuclease subunit; SLX1A-SULT1A3 readthrough (NMD candidate); SLX1B-SULT1A4 readthrough (NMD candidate); sulfotransferase family 1A member 3	SLX1A; SLX1B; SLX1A-SULT1A3; SLX1B-SULT1A4; SULT1A3	1,24
Transcript Identified by AceView, Entrez Gene ID(s) 7265	TTC1	1,24
solute carrier family 15 (oligopeptide transporter), member 1	SLC15A1	1,24
zinc finger protein 626	ZNF626	1,24
delta-like 1 (Drosophila)	DLL1	1,24
secretory carrier membrane protein 4; adenosine deaminase, tRNA-specific 3	SCAMP4; ADAT3	1,24
microtubule associated serine/threonine kinase-like	MASTL	1,24
solute carrier family 30 (zinc transporter), member 6	SLC30A6	1,24

ADP-ribosylation factor 6	ARF6	1,24
ras homolog family member B	RHOB	1,24
DDHD domain containing 1	DDHD1	1,24
SH3-domain binding protein 5 (BTK-associated)	SH3BP5	1,24
thimet oligopeptidase 1	THOP1	1,24
RGP1 homolog, RAB6A GEF complex partner 1	RGP1	1,24
protein tyrosine phosphatase, receptor type, R	PTPRR	1,24
general transcription factor IIF subunit 2	GTF2F2	1,24
GRAM domain containing 1B	GRAMD1B	1,24
immunoglobulin superfamily containing leucine-rich repeat	ISLR	1,24
uncharacterized LOC100131315; novel transcript	LOC100131315; RP11-758N13.1	1,24
chromosome 2 open reading frame 69	C2orf69	1,24
zinc finger, DHHC-type containing 3	ZDHHC3	1,24
general transcription factor IIIA	GTF3A	1,24
ribosomal protein L10-like	RPL10L	1,24
solute carrier family 15 (oligopeptide transporter), member 3	SLC15A3	1,24
fermitin family member 1	FERMT1	1,24
transcription factor 4	TCF4	1,24
ras-related C3 botulinum toxin substrate 1 (rho family, small GTP binding protein Rac1)	RAC1	1,24
trafficking protein particle complex 4	TRAPPC4	1,24
long intergenic non-protein coding RNA 1343	LINC01343	1,24
transmembrane protein 52B	TMEM52B	1,24
involucrin	IVL	1,24
membrane associated ring finger 4	MARCH4	1,24
Cdc42 guanine nucleotide exchange factor 9; ARHGEF9 intronic transcript 1	ARHGEF9; ARHGEF9-IT1	1,24
claudin 8	CLDN8	1,24
coiled-coil domain containing 84	CCDC84	1,24
phosphatidylinositol glycan anchor biosynthesis class C	PIGC	1,24
Memczak2013 ANTISENSE, CDS, coding, INTERNAL best transcript NM_001080453	INTS1	1,24
nuclear factor of activated T-cells, cytoplasmic, calcineurin-dependent 4	NFATC4	1,24
ADP-ribosylhydrolase like 1	ADPRHL1	1,24
signal-regulatory protein gamma	SIRPG	1,24
caspase recruitment domain family, member 17	CARD17	1,24
ATPase, Ca++ transporting, plasma membrane 4	ATP2B4	1,24
transmembrane protein 237	TMEM237	1,24
DIS3 like 3-5 exoribonuclease 2	DIS3L2	1,24
HIG1 hypoxia inducible domain family, member 2B	HIGD2B	1,24
peptidoglycan recognition protein 3	PGLYRP3	1,24

RNA (guanine-7-) methyltransferase	RNMT	1,24
G protein regulated inducer of neurite outgrowth 2	GPRIN2	1,24
intestinal cell (MAK-like) kinase	ICK	1,24
5,3-nucleotidase, mitochondrial	NT5M	1,24
transcription factor 20 (AR1)	TCF20	1,24
transcription factor 20 (AR1)	TCF20	1,24
proteasome subunit beta 1	PSMB1	1,24
chromosome 21 open reading frame 59	C21orf59	1,24
cyclin D-type binding-protein 1	CCNDBP1	1,24
transmembrane protein 114	TMEM114	1,24
nuclear pore complex interacting protein family, member B6	NPIPB6	1,24
chromosome X open reading frame 57	CXorf57	1,24
pleckstrin homology-like domain, family A, member 3	PHLDA3	1,24
kelch-like family member 36	KLHL36	1,24
G antigen 12I; G antigen 12F	GAGE12I; GAGE12F	1,24
misshapen-like kinase 1	MINK1	1,24
uncharacterized LOC102724238; uncharacterized LOC554249	LOC102724238; LOC554249	1,24
cholinergic receptor, muscarinic 4	CHRM4	1,24
HEAT repeat containing 4	HEATR4	1,24
transmembrane protein 261	TMEM261	1,24
cadherin 15, type 1, M-cadherin (myotubule)	CDH15	1,24
membrane-spanning 4-domains, subfamily A, member 15	MS4A15	1,24
FERM and PDZ domain containing 1	FRMPD1	1,24
KH homology domain containing 1	KHDC1	1,24
transient receptor potential cation channel, subfamily M, member 3	TRPM3	1,24
WAP, follistatin/kazal, immunoglobulin, kunitz and netrin domain containing 2	WFIKKN2	1,24
sterile alpha motif domain containing 14	SAMD14	1,24
eukaryotic translation initiation factor 1A domain containing	EIF1AD	1,24
JAKMIP2 antisense RNA 1	JAKMIP2-AS1	1,24
chromosome 10 open reading frame 54	C10orf54	1,24
early growth response 3	EGR3	1,24
acrosomal vesicle protein 1	ACRV1	1,24
phosphatidylserine decarboxylase; microRNA 7109	PISD; MIR7109	1,24
mitochondrial calcium uptake 1	MICU1	1,24
toll-like receptor 1	TLR1	1,24
zinc finger protein 641	ZNF641	1,24
chromosome 1 open reading frame 123	C1orf123	1,24

bladder cancer associated transcript 1 (non-protein coding); LEM domain containing 1	BLACAT1; LEMD1	1,24
piggyBac transposable element derived 5	PGBD5	1,24
cysteinyl-tRNA synthetase	CARS	1,24
syntaxin binding protein 5-like	STXBP5L	1,24
leucine rich repeat containing 75A	LRRC75A	1,24
zinc finger protein 667	ZNF667	1,24
polymerase (RNA) II (DNA directed) polypeptide G	POLR2G	1,24
zinc finger protein 680	ZNF680	1,24
hdc homolog, cell cycle regulator	HECA	1,24
mitochondrial ribosomal protein L27	MRPL27	1,24
zinc finger protein 600	ZNF600	1,24
chromosome 19 open reading frame 35	C19orf35	1,24
nescient helix-loop-helix 2	NHLH2	1,24
rhodopsin	RHO	1,24
OTU deubiquitinase 1	OTUD1	1,24
RAB6C, member RAS oncogene family	RAB6C	1,24
ALDH1L1 antisense RNA 1	ALDH1L1-AS1	1,24
actin-like 8	ACTL8	1,24
cysteine sulfinic acid decarboxylase	CSAD	1,24
protamine 1	PRM1	1,24
StAR-related lipid transfer domain containing 3	STARD3	1,24
IMP4 homolog, U3 small nucleolar ribonucleoprotein	IMP4	1,24
zinc finger, DHHC-type containing 14	ZDHHC14	1,24
crystallin beta A2	CRYBA2	1,24
solute carrier family 9, subfamily A (NHE2, cation proton antiporter 2), member 2	SLC9A2	1,24
EF-hand calcium binding domain 1	EFCAB1	1,24
sialic acid binding Ig-like lectin 6	SIGLEC6	1,24
protein phosphatase 1, regulatory (inhibitor) subunit 14C	PPP1R14C	1,24
FXYD domain containing ion transport regulator 7	FXYD7	1,24
ATPase, H+/K+ exchanging, alpha polypeptide	ATP4A	1,24
Memczak2013 ALT_ACCEPTOR, ALT_DONOR, coding, INTERNAL, intronic best transcript NM_006224	PITPNA	1,24
prostaglandin E receptor 2	PTGER2	1,24
fatty acid binding protein 12	FABP12	1,24
SWI/SNF related, matrix associated, actin dependent regulator of chromatin, subfamily c, member 2	SMARCC2	1,24
CUB and Sushi multiple domains 1	CSMD1	1,24
peroxisomal biogenesis factor 19	PEX19	1,24

Transcript Identified by AceView, Entrez Gene ID(s) 23527; ACAP2 intronic transcript 1 (non-protein coding) [Source:HGNC Symbol;Acc:HGNC:41426]; novel transcript; ACAP2 intronic transcript 1 [Source:HGNC Symbol;Acc:HGNC:41426]	ACAP2; ACAP2-IT1; AC090018.3	1,24
late cornified envelope 3E	LCE3E	1,23
G protein pathway suppressor 1	GPS1	1,23
deleted in azoospermia 2; deleted in azoospermia 4	DAZ2; DAZ4	1,23
glutamate receptor, ionotropic, kainate 5	GRIK5	1,23
heat shock 70kDa protein 4-like	HSPA4L	1,23
thiopurine S-methyltransferase	TPMT	1,23
deleted in lymphocytic leukemia 1 (non-protein coding)	DLEU1	1,23
spermatogenesis associated 6-like	SPATA6L	1,23
RuvB-like AAA ATPase 1	RUVBL1	1,23
bromodomain and PHD finger containing 1	BRPF1	1,23
solute carrier family 4 (anion exchanger), member 1 (Diego blood group)	SLC4A1	1,23
fibroblast growth factor 9	FGF9	1,23
leucine carboxyl methyltransferase 2	LCMT2	1,23
MMP25 antisense RNA 1	MMP25-AS1	1,23
torsin family 2, member A	TOR2A	1,23
HMG box domain containing 4	HMGXB4	1,23
chromosome 2 open reading frame 50	C2orf50	1,23
peroxisomal biogenesis factor 16	PEX16	1,23
adrenoceptor alpha 1D	ADRA1D	1,23
chemokine (C-C motif) ligand 3-like 3; chemokine (C-C motif) ligand 3-like 1	CCL3L3; CCL3L1	1,23
CCAAT/enhancer binding protein (C/EBP), beta	CEBPB	1,23
transmembrane protein 44	TMEM44	1,23
N-ethylmaleimide-sensitive factor attachment protein, gamma	NAPG	1,23
DnaJ (Hsp40) homolog, subfamily A, member 4	DNAJA4	1,23
solute carrier family 25 (mitochondrial carrier; peroxisomal membrane protein, 34kDa), member 17	SLC25A17	1,23
keratin associated protein 5-1	KRTAP5-1	1,23
mir-99a-let-7c cluster host gene	MIR99AHG	1,23
excision repair cross-complementation group 1	ERCC1	1,23
forty-two-three domain containing 1	FYTTD1	1,23
tubulin, alpha 3c	TUBA3C	1,23
TAF1 RNA polymerase II, TATA box binding protein (TBP)-associated factor, 210kDa-like	TAF1L	1,23
phospholipase C, gamma 2 (phosphatidylinositol-specific)	PLCG2	1,23
vacuolar protein sorting 33 homolog A (S. cerevisiae)	VPS33A	1,23

protein phosphatase 1, regulatory (inhibitor) subunit 14D	PPP1R14D	1,23
cyclin-dependent kinase 5, regulatory subunit 1 (p35)	CDK5R1	1,23
deoxynucleotidyltransferase, terminal, interacting protein 1	DNTTIP1	1,23
solute carrier family 30 (zinc transporter), member 3	SLC30A3	1,23
Fanconi anemia complementation group B	FANCB	1,23
chemokine (C-X-C motif) ligand 3	CXCL3	1,23
TraB domain containing 2B	TRABD2B	1,23
family with sequence similarity 110, member D	FAM110D	1,23
nitric oxide synthase interacting protein	NOSIP	1,23
novel transcript; Transcript Identified by AceView, Entrez Gene ID(s) 389432	RP11-307P5.1; SAMD5	1,23
cat eye syndrome chromosome region, candidate 5	CECR5	1,23
nucleoporin 58kDa	NUP58	1,23
small proline-rich protein 2E	SPRR2E	1,23
signal peptide, CUB domain, EGF-like 1	SCUBE1	1,23
NPHP3-ACAD11 readthrough (NMD candidate)	NPHP3-ACAD11	1,23
integrin beta 3 binding protein (beta3-endonexin)	ITGB3BP	1,23
NOP58 ribonucleoprotein	NOP58	1,23
zinc finger, HIT-type containing 2	ZNHIT2	1,23
leucine rich repeat and fibronectin type III domain containing 5	LRFN5	1,23
absent in melanoma 2	AIM2	1,23
fibronectin leucine rich transmembrane protein 2	FLRT2	1,23
interferon stimulated exonuclease gene 20kDa like 2	ISG20L2	1,23
family with sequence similarity 228, member B	FAM228B	1,23
proline dehydrogenase (oxidase) 1	PRODH	1,23
neurofilament, medium polypeptide	NEFM	1,23
potassium channel, voltage gated Shal related subfamily D, member 1	KCND1	1,23
bolA family member 2; bolA family member 2B	BOLA2; BOLA2B	1,23
neuroblastoma breakpoint family, member 15	NBPF15	1,23
zinc finger, NFX1-type containing 1	ZNFX1	1,23
sorting nexin 3	SNX3	1,23
SGT1 homolog, MIS12 kinetochore complex assembly cochaperone	SUGT1	1,23
serine/arginine repetitive matrix 2	SRRM2	1,23
TELO2 interacting protein 2	TTI2	1,23
S-antigen; retina and pineal gland (arrestin)	SAG	1,23
vesicular, overexpressed in cancer, prosurvival protein 1	VOPP1	1,23
HKR1, GLI-Kruppel zinc finger family member	HKR1	1,23
olfactory receptor, family 51, subfamily S, member 1	OR51S1	1,23
Transcript Identified by AceView, Entrez Gene ID(s) 23144	ZC3H3	1,23

zinc finger and BTB domain containing 40	ZBTB40	1,23
copper metabolism (Murr1) domain containing 1	COMMD1	1,23
leucine rich transmembrane and O-methyltransferase domain containing	LRTOMT	1,23
RAN binding protein 9	RANBP9	1,23
Zhang2013 ALT_ACCEPTOR, ALT_DONOR, coding, INTERNAL, intronic best transcript NM_001122823	GTF3C5	1,23
cytochrome c oxidase subunit II	COX2	1,23
olfactory receptor, family 9, subfamily K, member 2	OR9K2	1,23
karyopherin alpha 6 (importin alpha 7)	KPNA6	1,23
ataxia, cerebellar, Cayman type	ATCAY	1,23
syntaxin binding protein 5 (tomosyn)	STXBP5	1,23
mitogen-activated protein kinase kinase kinase 14	MAP3K14	1,23
keratin associated protein 19-5	KRTAP19-5	1,23
transmembrane and coiled-coil domain family 3; microRNA 7844	TMCC3; MIR7844	1,23
periphilin 1	PPHLN1	1,23
SKI2 homolog, superkiller viralicidic activity 2-like	SKIV2L	1,23
C-type lectin domain family 4, member A	CLEC4A	1,23
calcium channel, voltage-dependent, beta 1 subunit	CACNB1	1,23
PRAME family member 19 [Source:EntrezGene;Acc:645414]	PRAMEF19	1,23
triggering receptor expressed on myeloid cells-like 2	TREML2	1,23
B-cell CLL/lymphoma 3; microRNA 8085	BCL3; MIR8085	1,23
protocadherin 1	PCDH1	1,23
TEA domain family member 4	TEAD4	1,23
NLR family, pyrin domain containing 11	NLRP11	1,23
coiled-coil domain containing 28A	CCDC28A	1,23
t-complex 11 family, X-linked 2	TCP11X2	1,23
sushi domain containing 5	SUSD5	1,23
V-set and immunoglobulin domain containing 4	VSIG4	1,23
paired box 8	PAX8	1,23
cytochrome c oxidase subunit VIb polypeptide 1 (ubiquitous)	COX6B1	1,23
valosin containing protein (p97)/p47 complex interacting protein 1	VCPIP1	1,23
trafficking protein particle complex 4	TRAPPC4	1,23
RAB36, member RAS oncogene family	RAB36	1,23
sialic acid binding Ig-like lectin 11	SIGLEC11	1,23
heat shock protein 90kDa alpha (cytosolic), class A member 1	HSP90AA1	1,23
Rho GTPase activating protein 27	ARHGAP27	1,23
serine peptidase inhibitor, Kazal type 14 (putative)	SPINK14	1,23
SWI/SNF related, matrix associated, actin dependent regulator of chromatin, subfamily d, member 3	SMARCD3	1,23

SET domain containing 4	SETD4	1,23
MAGE family member A11	MAGEA11	1,23
ST6 (alpha-N-acetyl-neuraminyl-2,3-beta-galactosyl-1,3)-N-acetylgalactosaminide alpha-2,6-sialyltransferase 5	ST6GALNAC5	1,23
zinc finger protein 812, pseudogene	ZNF812P	1,23
SLX1 homolog A, structure-specific endonuclease subunit; SLX1 homolog B, structure-specific endonuclease subunit	SLX1A; SLX1B	1,23
coiled-coil domain containing 113	CCDC113	1,23
ST8 alpha-N-acetyl-neuraminide alpha-2,8-sialyltransferase 1	ST8SIA1	1,23
mediator complex subunit 16	MED16	1,23
protein phosphatase, EF-hand calcium binding domain 2	PPEF2	1,23
ADAM metallopeptidase domain 29	ADAM29	1,23
trafficking protein particle complex 4	TRAPPC4	1,23
microcephalin 1	MCPH1	1,23
AT hook, DNA binding motif, containing 1	AHDC1	1,23
SET domain containing (lysine methyltransferase) 7	SETD7	1,23
leucine rich repeat containing 16B	LRRC16B	1,23
ribosomal protein S6 kinase, 90kDa, polypeptide 1	RPS6KA1	1,23
adenylate kinase 8	AK8	1,23
splicing factor 3b subunit 6	SF3B6	1,23
survival motor neuron domain containing 1	SMNDC1	1,23
olfactory receptor, family 52, subfamily I, member 2	OR52I2	1,23
peptidyl arginine deiminase, type IV	PADI4	1,23
nudix hydrolase 15	NUDT15	1,23
LIM domain containing 2	LIMD2	1,23
mediator complex subunit 17	MED17	1,23
proteasome 26S subunit, non-ATPase 4	PSMD4	1,23
TBC1 domain family, member 28	TBC1D28	1,23
coiled-coil domain containing 81	CCDC81	1,23
ubiquitin specific peptidase 8	USP8	1,23
carbonic anhydrase IV	CA4	1,23
keratin associated protein 5-4	KRTAP5-4	1,23
Rho guanine nucleotide exchange factor 4	ARHGEF4	1,23
crystallin gamma N	CRYGN	1,23
zinc finger, C4H2 domain containing	ZC4H2	1,23
flotillin 2	FLOT2	1,23
uncharacterized LOC101928436; novel transcript; Transcript Identified by AceView	LOC101928436; RP11-153F1.1; RP11-347K2.2; teyfawbu	1,23
prostate and testis expressed 4	PATE4	1,23
Down syndrome critical region 10 (non-protein coding)	DSCR10	1,23
family with sequence similarity 81, member A	FAM81A	1,23

neurocalcin delta	NCALD	1,22
TMEM189-UBE2V1 readthrough	TMEM189-UBE2V1	1,22
LY6/PLAUR domain containing 3	LYPD3	1,22
glutathione S-transferase kappa 1	GSTK1	1,22
FAT atypical cadherin 1	FAT1	1,22
HECT, C2 and WW domain containing E3 ubiquitin protein ligase 1	HECW1	1,22
cartilage acidic protein 1	CRTAC1	1,22
PWP2 periodic tryptophan protein homolog (yeast)	PWP2	1,22
leptin	LEP	1,22
Jeck2013 ALT_ACCEPTOR, ALT_DONOR, coding, INTERNAL, intronic best transcript NM_032531	KIRREL3	1,22
hexokinase 3 (white cell)	HK3	1,22
wingless-type MMTV integration site family, member 6	WNT6	1,22
zinc finger CCHC-type and RNA binding motif 1; microRNA 7851	ZCRB1; MIR7851	1,22
acid phosphatase 6, lysophosphatidic	ACP6	1,22
small integral membrane protein 2	SMIM2	1,22
inositol polyphosphate-5-phosphatase D	INPP5D	1,22
spectrin, alpha, non-erythrocytic 1	SPTAN1	1,22
keratin associated protein 19-4	KRTAP19-4	1,22
VAMP (vesicle-associated membrane protein)-associated protein B and C	VAPB	1,22
nucleic acid binding protein 2	NABP2	1,22
emerin	EMD	1,22
keratin associated protein 4-1	KRTAP4-1	1,22
Memczak2013 ALT_ACCEPTOR, ALT_DONOR, coding, INTERNAL, intronic best transcript NM_001031711	ERGIC1	1,22
myosin phosphatase Rho interacting protein	MPRIP	1,22
NADPH oxidase 1	NOX1	1,22
hydroxysteroid (17-beta) dehydrogenase 14	HSD17B14	1,22
myosin light chain, phosphorylatable, fast skeletal muscle	MYLPF	1,22
transmembrane protein 241	TMEM241	1,22
interleukin 20 receptor, alpha	IL20RA	1,22
novel transcript; Transcript Identified by AceView, Entrez Gene ID(s) 199964	RP11-67L3.5; RP11-67L3.4; TMEM61	1,22
keratin associated protein 1-5	KRTAP1-5	1,22
transmembrane protein 14E, pseudogene	TMEM14EP	1,22
ribonuclease, RNase A family, 1 (pancreatic)	RNASE1	1,22
opioid binding protein/cell adhesion molecule-like	OPCML	1,22
CD177 molecule	CD177	1,22
proline rich, Y-linked	PRORY	1,22

Salzman2013 ANNOTATED, CDS, coding, INTERNAL, OVCODE, OVEXON best transcript NM_006219	PIK3CB	1,22
zinc finger protein 491	ZNF491	1,22
Leber congenital amaurosis 5-like	LCA5L	1,22
zinc finger protein 582	ZNF582	1,22
cytochrome P450, family 2, subfamily A, polypeptide 7	CYP2A7	1,22
C1q and tumor necrosis factor related protein 2	C1QTNF2	1,22
otoferlin	OTOF	1,22
bobby sox homolog (Drosophila)	BBX	1,22
zinc finger, DHHC-type containing 21	ZDHHC21	1,22
ubiquitin conjugating enzyme E2D 3	UBE2D3	1,22
chromosome 9 open reading frame 43	C9orf43	1,22
keratin associated protein 4-8	KRTAP4-8	1,22
Memczak2013 ANTISENSE, coding, INTERNAL, intronic best transcript NM_079423	MYL6	1,22
centromere protein N	CENPN	1,22
Transcript Identified by AceView, Entrez Gene ID(s) 4643	MYO1E	1,22
UDP-galactose-4-epimerase	GALE	1,22
MAS1 proto-oncogene, G protein-coupled receptor	MAS1	1,22
complement component 8, gamma polypeptide	C8G	1,22
methyltransferase like 21C	METTL21C	1,22
long intergenic non-protein coding RNA 1387	LINC01387	1,22
BCL2-like 14 (apoptosis facilitator)	BCL2L14	1,22
chromosome 9 open reading frame 9	C9orf9	1,22
membrane associated guanylate kinase, WW and PDZ domain containing 3	MAGI3	1,22
neurexophilin 2	NXPH2	1,22
mohawk homeobox	MKX	1,22
forkhead box F1	FOXF1	1,22
neuromedin U receptor 2	NMUR2	1,22
glucosamine-6-phosphate deaminase 2	GNPDA2	1,22
family with sequence similarity 229, member A	FAM229A	1,22
Hermansky-Pudlak syndrome 3	HPS3	1,22
retinoblastoma binding protein 5	RBBP5	1,22
protein-O-mannosyltransferase 2	POMT2	1,22
pre-mRNA processing factor 31	PRPF31	1,22
deltex 3, E3 ubiquitin ligase	DTX3	1,22
keratin 13, type I	KRT13	1,22
TMF1-regulated nuclear protein 1	TRNP1	1,22
ATPase, H+ transporting, lysosomal 13kDa, V1 subunit G3	ATP6V1G3	1,22
CD72 molecule	CD72	1,22

uncharacterized LOC100652768; Salzman2013 ANNOTATED, ncRNA, OVCODE, OVERLAPTX, OVEXON best transcript NR_045215; Transcript Identified by AceView, Entrez Gene ID(s) 9159	LOC100652768; PCSK7	1,22
adaptor-related protein complex 2, alpha 1 subunit	AP2A1	1,22
G antigen 4; G antigen 7; G antigen 12G	GAGE4; GAGE7; GAGE12G	1,22
lectin, galactoside-binding, soluble, 7B	LGALS7B	1,22
Zhang2013 ALT_ACCEPTOR, ALT_DONOR, coding, INTERNAL, intronic best transcript NM_020338	ZMIZ1	1,22
Transcript Identified by AceView, Entrez Gene ID(s) 4731	NDUFV3	1,22
N-acetylneuraminate pyruvate lyase (dihydrodipicolinate synthase)	NPL	1,22
phenylalanyl-tRNA synthetase beta subunit	FARSB	1,22
nerve growth factor receptor	NGFR	1,22
MAGE family member A8	MAGEA8	1,22
interleukin 18 receptor accessory protein	IL18RAP	1,22
zinc finger protein 182	ZNF182	1,22
B-cell receptor-associated protein 31	BCAP31	1,22
zinc finger protein 750	ZNF750	1,22
CDP-diacylglycerol--inositol 3-phosphatidyltransferase	CDIPT	1,22
gamma-aminobutyric acid (GABA) B receptor, 1	GABBR1	1,22
centrosomal protein 76kDa	CEP76	1,22
thrombospondin type 1 domain containing 4	THSD4	1,22
SECIS binding protein 2-like	SECISBP2L	1,22
G protein regulated inducer of neurite outgrowth 1	GPRIN1	1,22
gap junction protein alpha 4	GJA4	1,22
acetylcholinesterase (Yt blood group)	ACHE	1,22
urocortin 3	UCN3	1,22
G protein-coupled receptor 162	GPR162	1,22
ubiquitin specific peptidase 17-like family member 2	USP17L2	1,22
diphthamide biosynthesis 3	DPH3	1,22
tubulin, beta 4A class IVa	TUBB4A	1,22
translocase of inner mitochondrial membrane domain containing 1	TIMMDC1	1,22
chromosome 5 open reading frame 52	C5orf52	1,22
protein phosphatase, Mg2+/Mn2+ dependent, 1M	PPM1M	1,22
scavenger receptor cysteine rich family, 5 domains	SSC5D	1,22
lymphocyte transmembrane adaptor 1	LAX1	1,22
keratin 74, type II	KRT74	1,22
zinc finger, DHHC-type containing 16	ZDHHC16	1,22
olfactory receptor, family 10, subfamily Z, member 1	OR10Z1	1,22
chromosome 20 open reading frame 78	C20orf78	1,22
exocyst complex component 3-like 2	EXOC3L2	1,22

transmembrane protein 42	TMEM42	1,22
stromal interaction molecule 1; microRNA 4687	STIM1; MIR4687	1,22
myelin transcription factor 1	MYT1	1,22
ryanodine receptor 2 (cardiac)	RYR2	1,22
methionyl-tRNA synthetase 2, mitochondrial	MARS2	1,22
mitochondrial trans-2-enoyl-CoA reductase	MECR	1,22
ubinuclein 1	UBN1	1,22
kielin/chordin-like protein	KCP	1,22
nudix hydrolase 17	NUDT17	1,22
anti-Mullerian hormone; microRNA 4321	AMH; MIR4321	1,22
RNA binding motif protein, Y-linked, family 1, member J; RNA binding motif protein, Y-linked, family 1, member F	RBMY1J; RBMY1F	1,22
lipocalin 9	LCN9	1,22
chromosome 22 open reading frame 24	C22orf24	1,22
tektin 5	TEKT5	1,22
WAP four-disulfide core domain 1	WFDC1	1,22
cilia and flagella associated protein 45	CFAP45	1,22
calcium binding protein 5	CABP5	1,22
heat shock 70kDa protein 12A	HSPA12A	1,22
phospholipid phosphatase 2	PLPP2	1,22
netrin G1	NTNG1	1,22
coiled-coil domain containing 84	CCDC84	1,22
diacylglycerol kinase, delta 130kDa	DGKD	1,22
DCN1, defective in cullin neddylation 1, domain containing 3	DCUN1D3	1,22
REST corepressor 3	RCOR3	1,22
zinc finger protein 486	ZNF486	1,22
COMM domain containing 5	COMMD5	1,22
selenoprotein W, 1	SEPW1	1,22
InaF-motif containing 1	INAFM1	1,22
Transcript Identified by AceView, Entrez Gene ID(s) 5552	SRGN	1,22
lysosomal-associated membrane protein 1	LAMP1	1,22
TSC22 domain family, member 1	TSC22D1	1,22
Fas (TNFRSF6)-associated via death domain	FADD	1,22
Transcript Identified by AceView, Entrez Gene ID(s) 7227	TRPS1	1,22
zinc finger, X-linked, duplicated B	ZXDB	1,22
cilia and flagella associated protein 73	CFAP73	1,22
ribosomal protein S6 kinase, 90kDa, polypeptide 1	RPS6KA1	1,22
zinc finger, MYND-type containing 8	ZMYND8	1,22
signal recognition particle 14kDa	SRP14	1,22
G protein-coupled receptor 142	GPR142	1,22

calcium channel, voltage-dependent, L type, alpha 1D subunit	CACNA1D	1,21
TATA box binding protein associated factor 8	TAF8	1,21
chromosome 9 open reading frame 170	C9orf170	1,21
otospiralin	OTOS	1,21
oxysterol binding protein-like 6	OSBPL6	1,21
uncharacterized LOC150527; uncharacterized LOC646743; Transcript Identified by AceView, Entrez Gene ID(s) 646743, RefSeq ID(s) NR_033930; Uncharacterized protein [Source:UniProtKB/TrEMBL;Acc:B9A039]	TISP43; LOC646743; AC013269.5	1,21
Fc fragment of IgG, low affinity IIa, receptor (CD32)	FCGR2A	1,21
membrane associated ring finger 2	MARCH2	1,21
pyrin and HIN domain family, member 1	PYHIN1	1,21
G antigen 1	GAGE1	1,21
cyclin-dependent kinase 9	CDK9	1,21
EF-hand calcium binding domain 5	EFCAB5	1,21
dynactin 3 (p22)	DCTN3	1,21
alkaline phosphatase, placental	ALPP	1,21
rabphilin 3A	RPH3A	1,21
chromosome X open reading frame 66	CXorf66	1,21
prefoldin subunit 6; microRNA 6834	PFDN6; MIR6834	1,21
DnaJ (Hsp40) homolog, subfamily C, member 9	DNAJC9	1,21
FK506 binding protein 5	FKBP5	1,21
family with sequence similarity 25, member A	FAM25A	1,21
protein phosphatase 1, regulatory (inhibitor) subunit 14B	PPP1R14B	1,21
Transcript Identified by AceView, Entrez Gene ID(s) 284440; 440515	ZNF506	1,21
chromosome 8 open reading frame 33	C8orf33	1,21
endothelin receptor type A	EDNRA	1,21
zinc finger and SCAN domain containing 23	ZSCAN23	1,21
Transcript Identified by AceView, Entrez Gene ID(s) 5126	PCSK2	1,21
kinase suppressor of ras 2	KSR2	1,21
myosin XIX	MYO19	1,21
solute carrier family 10 (sodium/bile acid cotransporter), member 1	SLC10A1	1,21
solute carrier family 5 (sodium/iodide cotransporter), member 5	SLC5A5	1,21
leucine-zipper-like transcription regulator 1	LZTR1	1,21
endogenous retrovirus group V, member 1	ERVV-1	1,21
myeloid zinc finger 1	MZF1	1,21
variable charge, Y-linked; variable charge, Y-linked 1B	VCY; VCY1B	1,21
limb bud and heart development	LBH	1,21

EGF-like repeats and discoidin I-like domains 3	EDIL3	1,21
keratin associated protein 5-8	KRTAP5-8	1,21
Transcript Identified by AceView, Entrez Gene ID(s) 80230	RUFY1	1,21
centrosomal protein 290kDa	CEP290	1,21
olfactory receptor, family 2, subfamily A, member 4	OR2A4	1,21
podocalyxin-like	PODXL	1,21
NADH dehydrogenase (ubiquinone) 1 alpha subcomplex, 3, 9kDa	NDUFA3	1,21
meprin A, beta	MEP1B	1,21
cofilin 1 (non-muscle)	CFL1	1,21
protein phosphatase 1, regulatory subunit 12A	PPP1R12A	1,21
crystallin beta A4	CRYBA4	1,21
LIM and calponin homology domains 1	LIMCH1	1,21
tubulin, beta 8 class VIII	TUBB8	1,21
family with sequence similarity 228, member A	FAM228A	1,21
myosin ID	MYO1D	1,21
proline-rich coiled-coil 2A	PRRC2A	1,21
testis-specific serine kinase 3	TSSK3	1,21
polymerase (RNA) III (DNA directed) polypeptide G (32kD)-like	POLR3GL	1,21
transmembrane protein 106C	TMEM106C	1,21
proline and serine rich 2	PROSER2	1,21
lysophospholipase-like 1	LYPLAL1	1,21
glycophorin C (Gerbich blood group)	GYPC	1,21
MORN repeat containing 3	MORN3	1,21
syntrophin, gamma 1	SNTG1	1,21
B-cell linker	BLNK	1,21
proline rich 26	PRR26	1,21
block of proliferation 1; microRNA 7112	BOP1; MIR7112	1,21
caveolin 1	CAV1	1,21
chromosome 2 open reading frame 61	C2orf61	1,21
mitochondrial ribosomal protein S18A	MRPS18A	1,21
C-type lectin domain family 14, member A	CLEC14A	1,21
ferritin, heavy polypeptide 1 pseudogene 18	FTH1P18	1,21
Kruppel-like factor 1 (erythroid)	KLF1	1,21
neuronal growth regulator 1	NEGR1	1,21
myotubularin related protein 1	MTMR1	1,21
olfactory receptor, family 5, subfamily L, member 1 (gene/pseudogene)	OR5L1	1,21
chromosome 12 open reading frame 56	C12orf56	1,21
urocortin	UCN	1,21
tRNA aspartic acid methyltransferase 1	TRDMT1	1,21
glutamate receptor, metabotropic 7	GRM7	1,21
keratin associated protein 9-6	KRTAP9-6	1,21

stabilin 2	STAB2	1,21
cytochrome P450, family 4, subfamily A, polypeptide 22	CYP4A22	1,21
CSE1 chromosome segregation 1-like (yeast)	CSE1L	1,21
IQ motif containing H	IQCH	1,21
CD200 molecule	CD200	1,21
potassium channel, two pore domain subfamily K, member 2	KCNK2	1,21
G antigen 12F; G antigen 12J; G antigen 12D; G antigen 5; G antigen 6; G antigen 12B; G antigen 4; G antigen 2E	GAGE12F; GAGE12J; GAGE12D; GAGE5; GAGE6; GAGE12B; GAGE4; GAGE2E	1,21
immediate early response 5-like	IER5L	1,21
spectrin, beta, non-erythrocytic 1	SPTBN1	1,21
NDUFA4, mitochondrial complex associated	NDUFA4	1,21
PWWP domain containing 2B	PWWP2B	1,21
CD200 receptor 1	CD200R1	1,21
solute carrier family 24 (sodium/potassium/calcium exchanger), member 2	SLC24A2	1,21
olfactory receptor, family 2, subfamily T, member 2	OR2T2	1,21
coiled-coil domain containing 94	CCDC94	1,21
calcium binding protein 1	CABP1	1,21
calmodulin regulated spectrin-associated protein 1	CAMSAP1	1,21
family with sequence similarity 196, member A	FAM196A	1,21
leucine-rich repeats and WD repeat domain containing 1; microRNA 4467; microRNA 5090	LRWD1; MIR4467; MIR5090	1,21
DDB1 and CUL4 associated factor 4-like 1	DCAF4L1	1,21
keratin associated protein 5-11	KRTAP5-11	1,21
FtsJ RNA methyltransferase homolog 1 (E. coli)	FTSJ1	1,21
WD repeat domain 20	WDR20	1,21
myelin associated glycoprotein	MAG	1,21
cyclin N-terminal domain containing 1	CNTD1	1,21
solute carrier family 35, member E3	SLC35E3	1,21
Jeck2013 ANTISENSE, CDS, coding, INTERNAL, intronic, OVCODE, OVEXON best transcript NM_002183	IL3RA	1,21
ubiquitin specific peptidase 5 (isopeptidase T)	USP5	1,21
atrophin 1	ATN1	1,21
autophagy related 12	ATG12	1,21
cyclin D2	CCND2	1,21
casein kinase 1, alpha 1-like	CSNK1A1L	1,21
dual specificity tyrosine-(Y)-phosphorylation regulated kinase 1B	DYRK1B	1,21
keratin associated protein 12-1	KRTAP12-1	1,21

POU class 1 homeobox 1	POU1F1	1,21
solute carrier family 16 (monocarboxylate transporter), member 1	SLC16A1	1,21
family with sequence similarity 173, member A	FAM173A	1,21
solute carrier family 30 (zinc transporter), member 2	SLC30A2	1,21
TSNAX-DISC1 readthrough (NMD candidate)	TSNAX-DISC1	1,21
kinesin family member 24	KIF24	1,21
ER membrane protein complex subunit 10	EMC10	1,21
mucolipin 3	MCOLN3	1,21
steroid-5-alpha-reductase, alpha polypeptide 1 (3-oxo-5 alpha-steroid delta 4-dehydrogenase alpha 1)	SRD5A1	1,21
formin homology 2 domain containing 3	FHOD3	1,21
single stranded DNA binding protein 3	SSBP3	1,21
chromosome 2 open reading frame 44	C2orf44	1,21
nuclear pore complex interacting protein family, member B4	NPIPB4	1,21
transmembrane protein 102	TMEM102	1,21
NFS1 cysteine desulfurase	NFS1	1,21
somatostatin receptor 3	SSTR3	1,21
RAB8A, member RAS oncogene family	RAB8A	1,21
Harvey rat sarcoma viral oncogene homolog	HRAS	1,21
polymerase (DNA directed), lambda	POLL	1,21
S100 calcium binding protein A9	S100A9	1,21
tetratricopeptide repeat and ankyrin repeat containing 1	TRANK1	1,21
nuclear fragile X mental retardation protein interacting protein 1	NUFIP1	1,21
Memczak2013 ANTISENSE, coding, INTERNAL, intronic best transcript NM_153234	LIX1	1,21
unc-13 homolog A (C. elegans)	UNC13A	1,21
T-cell leukemia homeobox 1	TLX1	1,21
R3H domain containing 4	R3HDM4	1,21
microtubule associated monooxygenase, calponin and LIM domain containing 2	MICAL2	1,21
collagen and calcium binding EGF domains 1	CCBE1	1,21
keratin associated protein 3-2	KRTAP3-2	1,21
ubiquitin specific peptidase 14 (tRNA-guanine transglycosylase)	USP14	1,21
sterile alpha motif domain containing 9-like	SAMD9L	1,20
olfactory receptor, family 52, subfamily A, member 1	OR52A1	1,20
phospholipid phosphatase 4	PLPP4	1,20
long intergenic non-protein coding RNA 521	LINC00521	1,20
cysteine-rich protein 2	CRIP2	1,20
SH3 domain and tetratricopeptide repeats 1	SH3TC1	1,20
Usher syndrome 1C	USH1C	1,20
GATA binding protein 3	GATA3	1,20

olfactory receptor, family 8, subfamily H, member 2	OR8H2	1,20
regulator of G-protein signaling 7	RGS7	1,20
transmembrane protein 105	TMEM105	1,20
chromosome 19 open reading frame 45	C19orf45	1,20
solute carrier family 46, member 2	SLC46A2	1,20
granzyme A	GZMA	1,20
ubiquitin related modifier 1	URM1	1,20
vestigial-like family member 2	VGLL2	1,20
unc-93 homolog B1 (C. elegans)	UNC93B1	1,20
flavin containing monooxygenase 3	FMO3	1,20
vacuolar protein sorting 4 homolog A (S. cerevisiae)	VPS4A	1,20
sirtuin 1	SIRT1	1,20
intelectin 1 (galactofuranose binding)	ITLN1	1,20
chromosome 5 open reading frame 22	C5orf22	1,20
rhomboid, veinlet-like 3 (Drosophila)	RHBDL3	1,20
solute carrier family 4, sodium borate transporter, member 11	SLC4A11	1,20
NADH dehydrogenase (ubiquinone) Fe-S protein 5, 15kDa (NADH-coenzyme Q reductase)	NDUFS5	1,20
keratin 24, type I	KRT24	1,20
long intergenic non-protein coding RNA 1547	LINC01547	1,20
CDC42 binding protein kinase alpha (DMPK-like)	CDC42BPA	1,20
potassium channel, voltage gated eag related subfamily H, member 7	KCNH7	1,20
TruB pseudouridine (psi) synthase family member 2	TRUB2	1,20
bicaudal D homolog 2 (Drosophila)	BICD2	1,20
elongator acetyltransferase complex subunit 4	ELP4	1,20
GRAM domain containing 4	GRAMD4	1,20
widely interspaced zinc finger motifs	WIZ	1,20
kelch domain containing 4	KLHDC4	1,20
ankyrin repeat domain 62	ANKRD62	1,20
late cornified envelope-like proline-rich 1	LELP1	1,20
Transcript Identified by AceView; family with sequence similarity 25, member D [Source:HGNC Symbol;Acc:23588]; family with sequence similarity 25, member E [Source:HGNC Symbol;Acc:HGNC:23587]	buskee; FAM25D; FAM25E; zawskaw	1,20
grainyhead-like transcription factor 2	GRHL2	1,20
chromosome 1 open reading frame 127	C1orf127	1,20
myosin light chain 4	MYL4	1,20
leukocyte receptor cluster (LRC) member 1	LENG1	1,20
nucleolar protein 3 (apoptosis repressor with CARD domain)	NOL3	1,20
defensin, beta 114	DEFB114	1,20
phospholipase A2, group IVD (cytosolic)	PLA2G4D	1,20
syntabulin (syntaxin-interacting)	SYBU	1,20

serpin peptidase inhibitor, clade A (alpha-1 antiproteinase, antitrypsin), member 13, pseudogene	SERPINA13P	1,20
double homeobox 4 like 7	DUX4L7	1,20
neuroligin 1	NLGN1	1,20
AT rich interactive domain 4B (RBP1-like)	ARID4B	1,20
leucine rich repeat containing 37, member A3	LRRC37A3	1,20
H3 histone, family 3C	H3F3C	1,20
chromosome 17 open reading frame 112	C17orf112	1,20
S-antigen; retina and pineal gland (arrestin)	SAG	1,20
carbamoyl-phosphate synthase 1	CPS1	1,20
uroplakin 3B-like; polymerase (RNA) II (DNA directed) polypeptide J2	UPK3BL; POLR2J2	1,20
kizuna centrosomal protein	KIZ	1,20
annexin A10	ANXA10	1,20
Meis homeobox 3	MEIS3	1,20
ankyrin repeat and EF-hand domain containing 1	ANKEF1	1,20
ELOVL fatty acid elongase 7	ELOVL7	1,20
variable charge, Y-linked 1B	VCY1B	1,20
CCR4-NOT transcription complex subunit 7	CNOT7	1,20
GINS complex subunit 1 (Psf1 homolog)	GINS1	1,20
leucine rich repeat containing 9	LRRC9	1,20
transportin 1	TNPO1	1,20
MARVEL domain containing 2	MARVELD2	1,20
ankyrin repeat domain 33B	ANKRD33B	1,20
keratin 73, type II	KRT73	1,20
solute carrier family 38, member 9	SLC38A9	1,20
olfactory receptor, family 10, subfamily H, member 2	OR10H2	1,20
DNA replication and sister chromatid cohesion 1	DSCC1	1,20
fragile X mental retardation, autosomal homolog 2	FXR2	1,20
tubulin, beta class I	TUBB	1,20
olfactory receptor, family 5, subfamily AK, member 2	OR5AK2	1,20
	AC208162.1	1,20
keratin 81, type II	KRT81	1,20
CD96 molecule	CD96	1,20
homeobox B8	HOXB8	1,20
keratin 8, type II	KRT8	1,20
diphthamide biosynthesis 7	DPH7	1,20
Transcript Identified by AceView, Entrez Gene ID(s) 64327	LMBR1	1,20
uncharacterized LOC730159; putative novel transcript	LOC730159; RP11-360D2.1	1,20
BUB3 mitotic checkpoint protein	BUB3	1,20
LIM domain binding 2	LDB2	1,20
ubiquitin conjugating enzyme E2L 3	UBE2L3	1,20

amyloid beta (A4) precursor protein-binding, family B, member 1 interacting protein	APBB1IP	1,20
fibroblast growth factor 2 (basic)	FGF2	1,20
solute carrier family 16 (monocarboxylate transporter), member 1	SLC16A1	1,20
LanC lantibiotic synthetase component C-like 1 (bacterial)	LANCL1	1,20
atypical chemokine receptor 2	ACKR2	1,20
SAM domain, SH3 domain and nuclear localization signals 1	SAMSN1	1,20
zinc finger protein 25	ZNF25	1,20
ubiquitin protein ligase E3 component n-recognin 5	UBR5	1,20
stabilin 1	STAB1	1,20
discoidin domain receptor tyrosine kinase 1; microRNA 4640	DDR1; MIR4640	1,20
coronin, actin binding protein, 1C	CORO1C	1,20
WD repeat domain 87	WDR87	1,20
tropomyosin 1 (alpha)	TPM1	1,20
homeobox A10; homeobox A9; microRNA 196b	HOXA10; HOXA9; MIR196B	1,20
polymerase (DNA directed), epsilon 3, accessory subunit	POLE3	1,20
MIR1-1 host gene; microRNA 133a-2	MIR1-1HG; MIR133A2	1,20
zinc finger protein 35	ZNF35	1,20
troponin I type 3 (cardiac)	TNNI3	1,20
Fc receptor-like B	FCRLB	1,20
transcription factor 20 (AR1)	TCF20	1,20
tumor necrosis factor receptor superfamily, member 13C; microRNA 378i	TNFRSF13C; MIR378I	1,20
ectonucleoside triphosphate diphosphohydrolase 1	ENTPD1	1,20
mitochondrial ribosomal protein L49	MRPL49	1,20
OCIA domain containing 1	OCIAD1	1,20
G antigen 2D; G antigen 13; G antigen 2E; G antigen 8; G antigen 2B; G antigen 2A; G antigen 2C	GAGE2D; GAGE13; GAGE2E; GAGE8; GAGE2B; GAGE2A; GAGE2C	1,20
male-enhanced antigen 1	MEA1	1,20
collagen, type IV, alpha 3 (Goodpasture antigen)	COL4A3	1,20
phosphorylated adaptor for RNA export	PHAX	1,20
zinc finger protein 169	ZNF169	1,20
NIP7, nucleolar pre-rRNA processing protein	NIP7	1,20
PWP1 homolog, endonuclein	PWP1	1,20

T-cell leukemia/lymphoma 1B; T-cell leukemia/lymphoma 6 (non-protein coding)	TCL1B; TCL6	1,20
tripartite motif containing 33	TRIM33	1,20
catsper channel auxiliary subunit delta	CATSPERD	1,20
CCR4-NOT transcription complex subunit 6	CNOT6	1,20
Memczak2013 ALT_ACCEPTOR, ALT_DONOR, coding, INTERNAL, intronic best transcript NM_201998	SF1	1,20
peptidyl-tRNA hydrolase 1 homolog	PTRH1	1,20
tubulointerstitial nephritis antigen	TINAG	1,20
outer dense fiber of sperm tails 2-like	ODF2L	1,20
frizzled class receptor 1	FZD1	1,20
lymphoid-restricted membrane protein	LRMP	1,20
Homo sapiens REX1, RNA exonuclease 1 homolog-like 2, pseudogene (REXO1L2P), non-coding RNA.; Synthetic construct Homo sapiens clone IMAGE:100068172, MGC:195789 REX1, RNA exonuclease 1 homolog (S. cerevisiae)-like 1 (REXO1L1) mRNA, encodes complete protein.; Transcript Identified by AceView, RefSeq ID(s) NR_003594	REXO1L2P; REXO1L1; blarbeyby	1,20
integrin alpha X	ITGAX	1,20
endoplasmic reticulum to nucleus signaling 1	ERN1	1,20
helicase, POLQ-like	HELQ	1,20
guanylate cyclase activator 2A (guanylin)	GUCA2A	1,20
catenin (cadherin-associated protein), alpha-like 1	CTNNAL1	1,20
thiosulfate sulfurtransferase (rhodanese)-like domain containing 3	TSTD3	1,20
Tctex1 domain containing 1	TCTEX1D1	1,20
Memczak2013 ANTISENSE, CDS, coding, INTERNAL best transcript NM_017607	PPP1R12C	1,20
cadherin 10, type 2 (T2-cadherin)	CDH10	1,20
MT-RNR2-like 2	MTRNR2L2	1,20
peptidoglycan recognition protein 1	PGLYRP1	1,20
heterogeneous nuclear ribonucleoprotein A/B	HNRNPAB	1,20
Memczak2013 ALT_ACCEPTOR, ALT_DONOR, coding, INTERNAL, intronic best transcript NM_000332	ATXN1	1,20
tRNA methyltransferase 61A	TRMT61A	1,20
cytochrome P450, family 4, subfamily F, polypeptide 8	CYP4F8	1,20
adenine phosphoribosyltransferase	APRT	1,20
Transcript Identified by AceView, Entrez Gene ID(s) 79875	THSD4	1,20
mitochondrial ribosomal protein S15	MRPS15	1,20
DnaJ (Hsp40) homolog, subfamily B, member 11	DNAJB11	1,20
R-spondin 2	RSPO2	1,20
odorant binding protein 2B	OBP2B	1,20
polycystic kidney disease 2-like 1	PKD2L1	1,19

transducin-like enhancer of split 1 (E(sp1) homolog, Drosophila)	TLE1	1,19
Memczak2013 ALT_DONOR, coding, INTERNAL, intronic best transcript NM_001080437	SNED1	1,19
zinc finger and BTB domain containing 18	ZBTB18	1,19
regulator of G-protein signaling 11	RGS11	1,19
neurexophilin 3	NXPH3	1,19
gremlin 2, DAN family BMP antagonist	GREM2	1,19
transportin 2	TNPO2	1,19
solute carrier family 37, member 3	SLC37A3	1,19
PRAME family member 14	PRAMEF14	1,19
programmed cell death 2-like	PDCD2L	1,19
TANK-binding kinase 1	TBK1	1,19
PDZK1 interacting protein 1	PDZK1IP1	1,19
olfactory receptor, family 4, subfamily C, member 45	OR4C45	1,19
zinc finger protein 726; zinc finger protein 92 pseudogene 3	ZNF726; ZNF92P3	1,19
NOBOX oogenesis homeobox	NOBOX	1,19
peroxisomal, testis specific 1	PXT1	1,19
LanC lantibiotic synthetase component C-like 3 (bacterial)	LANCL3	1,19
Transcript Identified by AceView, Entrez Gene ID(s) 54453	RIN2	1,19
complement factor properdin	CFP	1,19
SWI/SNF related, matrix associated, actin dependent regulator of chromatin, subfamily a, member 4	SMARCA4	1,19
SNW domain containing 1	SNW1	1,19
proprotein convertase subtilisin/kexin type 2	PCSK2	1,19
homeodomain interacting protein kinase 1	HIPK1	1,19
coiled-coil domain containing 42	CCDC42	1,19
NADH dehydrogenase (ubiquinone) 1 alpha subcomplex, 12	NDUFA12	1,19
calcitonin receptor like receptor	CALCRL	1,19
p53 and DNA-damage regulated 1	PDRG1	1,19
cyclin-dependent kinase 4	CDK4	1,19
dynein, light chain, Tctex-type 3	DYNLT3	1,19
autophagy related 16-like 1	ATG16L1	1,19
forkhead box Q1	FOXQ1	1,19
cysteine-serine-rich nuclear protein 3	CSRNP3	1,19
Transcript Identified by AceView, Entrez Gene ID(s) 116154	PHACTR3	1,19
TBC1 domain family, member 22A	TBC1D22A	1,19
ribosomal RNA adenine dimethylase domain containing 1	RRNAD1	1,19
ubiquitin conjugating enzyme E2 variant 1	UBE2V1	1,19

monooxygenase, DBH-like 1	MOXD1	1,19
hypoxia inducible factor 1, alpha subunit inhibitor	HIF1AN	1,19
patched domain containing 2	PTCHD2	1,19
defensin, beta 136	DEFB136	1,19
FCF1 rRNA-processing protein	FCF1	1,19
NLR family, pyrin domain containing 9	NLRP9	1,19
formin 1	FMN1	1,19
zinc finger protein 283	ZNF283	1,19
DnaJ (Hsp40) homolog, subfamily C, member 7	DNAJC7	1,19
transmembrane protein 182	TMEM182	1,19
unc-5 netrin receptor A	UNC5A	1,19
OTU deubiquitinase 3	OTUD3	1,19
BTB (POZ) domain containing 11	BTBD11	1,19
short chain dehydrogenase/reductase family 42E, member 2	SDR42E2	1,19
surfactant protein D	SFTPD	1,19
cytochrome P450, family 2, subfamily E, polypeptide 1	CYP2E1	1,19
transcription factor EC	TFEC	1,19
coiled-coil domain containing 103	CCDC103	1,19
iduronidase, alpha-L-	IDUA	1,19
chloride channel, voltage-sensitive Ka	CLCNKA	1,19
chromosome 10 open reading frame 88	C10orf88	1,19
pumilio RNA binding family member 3	PUM3	1,19
MAD1 mitotic arrest deficient-like 1 (yeast)	MAD1L1	1,19
MAK16 homolog	MAK16	1,19
keratin associated protein 10-2	KRTAP10-2	1,19
ATP binding cassette subfamily B member 5	ABCB5	1,19
proline-rich protein BstNI subfamily 4	PRB4	1,19
chromosome 14 open reading frame 180	C14orf180	1,19
calmodulin 1 (phosphorylase kinase, delta)	CALM1	1,19
actin filament associated protein 1-like 2	AFAP1L2	1,19
G protein-coupled receptor 135	GPR135	1,19
3-oxoacid CoA-transferase 2	OXCT2	1,19
desmocollin 3	DSC3	1,19
Transcript Identified by AceView, Entrez Gene ID(s) 143279	HECTD2	1,19
chromosome 10 open reading frame 12	C10orf12	1,19
TIMELESS interacting protein	TIPIN	1,19
microtubule crosslinking factor 1	MTCL1	1,19
leucine rich repeat containing 42	LRRC42	1,19
myotubularin related protein 2	MTMR2	1,19
cathepsin V	CTSV	1,19
heparan sulfate 6-O-sulfotransferase 2	HS6ST2	1,19
proenkephalin	PENK	1,19
transmembrane protein 251	TMEM251	1,19

eukaryotic translation elongation factor 1 alpha 2	EEF1A2	1,19
zinc finger and BTB domain containing 11	ZBTB11	1,19
cadherin 11, type 2, OB-cadherin (osteoblast)	CDH11	1,19
defensin, alpha 4, corticostatin	DEFA4	1,19
rabaptin, RAB GTPase binding effector protein 1	RABEP1	1,19
defensin, beta 106A	DEFB106A	1,19
kinesin family member 1B	KIF1B	1,19
uncharacterized LOC340074; Transcript Identified by AceView, Entrez Gene ID(s) 340074, RefSeq ID(s) NR_027127; novel transcript	LOC340074; CTC-321K16.4	1,19
kelch-like family member 31	KLHL31	1,19
Memczak2013 ANTISENSE, coding, INTERNAL, intronic best transcript NM_032131	ARMC2	1,19
C1q and tumor necrosis factor related protein 5; membrane frizzled-related protein	C1QTNF5; MFRP	1,19
zinc finger protein 843	ZNF843	1,19
Fas (TNFRSF6) associated factor 1	FAF1	1,19
short chain dehydrogenase/reductase family 42E, member 1	SDR42E1	1,19
mucin 2, oligomeric mucus/gel-forming	MUC2	1,19
HD domain containing 2	HDDC2	1,19
t-complex 1; small nucleolar RNA, H/ACA box 20; small nucleolar RNA, H/ACA box 29	TCP1; SNORA20; SNORA29	1,19
phosphodiesterase 6H, cGMP-specific, cone, gamma	PDE6H	1,19
FAT atypical cadherin 3	FAT3	1,19
sosondowah ankyrin repeat domain family member B	SOWAHB	1,19
HERV-H LTR-associating 2	HHLA2	1,19
apolipoprotein B mRNA editing enzyme, catalytic polypeptide-like 3A	APOBEC3A	1,19
interferon-induced protein with tetratricopeptide repeats 1B	IFIT1B	1,19
bestrophin 1	BEST1	1,19
O-sialoglycoprotein endopeptidase	OSGEP	1,19
cramped chromatin regulator homolog 1	CRAMP1	1,19
small proline-rich protein 2B	SPRR2B	1,19
GDP-D-glucose phosphorylase 1	GDPGP1	1,19
zinc finger and BTB domain containing 38	ZBTB38	1,19
spindlin family, member 2A	SPIN2A	1,19
plexin D1	PLXND1	1,19
CWC25 spliceosome-associated protein homolog	CWC25	1,19
zinc finger protein 141	ZNF141	1,19
chromosome 14 open reading frame 119	C14orf119	1,19
ALS2 C-terminal like	ALS2CL	1,19
long intergenic non-protein coding RNA 504	LINC00504	1,19
programmed cell death 5	PDCD5	1,19

keratin 36, type I	KRT36	1,19
chromosome 11 open reading frame 72	C11orf72	1,19
bicaudal D homolog 1 (Drosophila)	BICD1	1,19
solute carrier family 18 (vesicular monoamine transporter), member 2	SLC18A2	1,19
PIH1 domain containing 3	PIH1D3	1,19
prostaglandin E synthase	PTGES	1,19
glutamate receptor, ionotropic, kainate 1	GRIK1	1,19
transmembrane BAX inhibitor motif containing 6	TMBIM6	1,19
ring finger protein 144B	RNF144B	1,19
R-spondin 3	RSPO3	1,19
X-linked Kx blood group related 3	XKR3	1,19
URB2 ribosome biogenesis 2 homolog (S. cerevisiae)	URB2	1,19
uromodulin	UMOD	1,19
SRC proto-oncogene, non-receptor tyrosine kinase	SRC	1,18
chloride channel, voltage-sensitive 5	CLCN5	1,18
chromosome 16 open reading frame 74	C16orf74	1,18
thioesterase superfamily member 4	THEM4	1,18
histidyl-tRNA synthetase	HARS	1,18
ribosomal RNA processing 12 homolog	RRP12	1,18
DEAD (Asp-Glu-Ala-Asp) box polypeptide 58	DDX58	1,18
golgi-associated, gamma adaptin ear containing, ARF binding protein 1	GGA1	1,18
stabilizer of axonemal microtubules 2	SAXO2	1,18
inhibitor of DNA binding 4, dominant negative helix-loop-helix protein	ID4	1,18
cyclin O	CCNO	1,18
histone cluster 1, H1t	HIST1H1T	1,18
achalasia, adrenocortical insufficiency, alacrimia	AAAS	1,18
ankyrin repeat and SOCS box containing 17	ASB17	1,18
recoverin	RCVRN	1,18
N(alpha)-acetyltransferase 35, NatC auxiliary subunit	NAA35	1,18
chromosome 11 open reading frame 63	C11orf63	1,18
olfactory receptor, family 8, subfamily B, member 12	OR8B12	1,18
myelin protein zero	MPZ	1,18
Transcript Identified by AceView, Entrez Gene ID(s) 50804	MYEF2	1,18
notochord homeobox	NOTO	1,18
phosducin	PDC	1,18
major facilitator superfamily domain containing 7	MFSD7	1,18
ATPase, Ca++ transporting, type 2C, member 2	ATP2C2	1,18
nucleolar and coiled-body phosphoprotein 1	NOLC1	1,18
poly(A) polymerase alpha	PAPOLA	1,18
Transcript Identified by AceView, Entrez Gene ID(s) 5136	PDE1A	1,18

Tctex1 domain containing 2	TCTEX1D2	1,18
myosin binding protein C, fast type	MYBPC2	1,18
free fatty acid receptor 1	FFAR1	1,18
olfactomedin 1	OLFM1	1,18
vesicle associated membrane protein 3	VAMP3	1,18
A kinase (PRKA) anchor protein 8-like	AKAP8L	1,18
E2F transcription factor 4, p107/p130-binding	E2F4	1,18
MAM domain containing 2	MAMDC2	1,18
acetylserotonin O-methyltransferase-like	ASMTL	1,18
acetylserotonin O-methyltransferase-like	ASMTL	1,18
wingless-type MMTV integration site family, member 9B	WNT9B	1,18
calcium channel, voltage-dependent, T type, alpha 1I subunit	CACNA1I	1,18
insulin-like 4 (placenta)	INSL4	1,18
family with sequence similarity 98, member B	FAM98B	1,18
coenzyme Q10B	COQ10B	1,18
nischarin	NISCH	1,18
polymerase (DNA directed), beta	POLB	1,18
ankyrin repeat domain 2 (stretch responsive muscle)	ANKRD2	1,18
CD79b molecule, immunoglobulin-associated beta	CD79B	1,18
chromosome 10 open reading frame 35	C10orf35	1,18
serpin peptidase inhibitor, clade B (ovalbumin), member 4	SERPINB4	1,18
lon peptidase 1, mitochondrial	LONP1	1,18
carboxypeptidase A5	CPA5	1,18
TBC1 domain family, member 7	TBC1D7	1,18
diacylglycerol kinase, eta	DGKH	1,18
olfactory receptor, family 2, subfamily A, member 42; olfactory receptor, family 2, subfamily A, member 1	OR2A42; OR2A1	1,18
transmembrane 4 L six family member 4	TM4SF4	1,18
keratin 71, type II	KRT71	1,18
uncharacterized LOC101927322; novel transcript, antisense to MALT1	LOC101927322; RP11-126O1.5	1,18
erythrocyte membrane protein band 4.1	EPB41	1,18
solute carrier family 24 (sodium/potassium/calcium exchanger), member 4	SLC24A4	1,18
topoisomerase (DNA) III beta	TOP3B	1,18
CTP synthase 1	CTPS1	1,18
golgin A8 family, member D, pseudogene	GOLGA8DP	1,18
golgin A6 family-like 10	GOLGA6L10	1,18
DC-STAMP domain containing 2	DCST2	1,18
ankyrin repeat and MYND domain containing 1	ANKMY1	1,18
huntingtin interacting protein 1	HIP1	1,18

Transcript Identified by AceView, Entrez Gene ID(s) 642812	API5L1	1,18
proline-rich protein BstNI subfamily 3	PRB3	1,18
DC-STAMP domain containing 1	DCST1	1,18
LAS1-like, ribosome biogenesis factor	LAS1L	1,18
G protein-coupled receptor kinase 4	GRK4	1,18
COX10 heme A:farnesyltransferase cytochrome c oxidase assembly factor	COX10	1,18
Eukaryotic translation initiation factor 3 subunit F [Source:UniProtKB/Swiss-Prot;Acc:O00303]	EIF3F	1,18
reticulocalbin 1, EF-hand calcium binding domain	RCN1	1,18
cyclin-dependent kinase 7	CDK7	1,18
sodium channel, voltage gated, type III beta subunit	SCN3B	1,18
squamous cell carcinoma antigen recognized by T-cells 3	SART3	1,18
integrin alpha V	ITGAV	1,18
heat shock transcription factor family, X-linked 1	HSFX1	1,18
claudin 22	CLDN22	1,18
chromosome 18 open reading frame 63	C18orf63	1,18
unc-79 homolog (C. elegans)	UNC79	1,18
pleckstrin homology-like domain, family B, member 3	PHLDB3	1,18
ras homolog family member A	RHOA	1,18
nuclear factor of kappa light polypeptide gene enhancer in B-cells inhibitor, beta	NFKBIB	1,18
CCR4-NOT transcription complex subunit 4	CNOT4	1,18
long intergenic non-protein coding RNA 1272	LINC01272	1,18
endonuclease, polyU-specific	ENDOU	1,18
X antigen family, member 5	XAGE5	1,18
Fli-1 proto-oncogene, ETS transcription factor	FLI1	1,18
profilin 1	PFN1	1,18
POM121 transmembrane nucleoporin-like 2	POM121L2	1,18
aftiphilin	AFTPH	1,18
SIX homeobox 4	SIX4	1,18
RAB23, member RAS oncogene family	RAB23	1,18
synapse associated protein 1	SYAP1	1,18
DnaJ (Hsp40) homolog, subfamily C, member 8	DNAJC8	1,18
peroxidasin	PXDN	1,18
T-cell activation RhoGTPase activating protein	TAGAP	1,18
family with sequence similarity 135, member B	FAM135B	1,18
glutamate receptor, ionotropic, kainate 4	GRIK4	1,18
coiled-coil-helix-coiled-coil-helix domain containing 2	CHCHD2	1,18
Homo sapiens LSM12 homolog (S. cerevisiae), mRNA (cDNA clone MGC:57206 IMAGE:4794614), complete cds.	LSM12	1,18
ubiquitin specific peptidase 20	USP20	1,18

Memczak2013 ANTISENSE, coding, INTERNAL, UTR3 best transcript NM_080677	DYNLL2	1,18
death associated protein 3	DAP3	1,18
olfactory receptor, family 10, subfamily H, member 3	OR10H3	1,18
zinc finger protein 23	ZNF23	1,18
collagen, type V, alpha 1	COL5A1	1,18
ferredoxin-fold anticodon binding domain containing 1	FDXACB1	1,18
ATPase family, AAA domain containing 1	ATAD1	1,18
nuclear receptor subfamily 2, group E, member 1	NR2E1	1,18
lysine (K)-specific demethylase 5C	KDM5C	1,18
membrane associated ring finger 6	MARCH6	1,18
leukocyte immunoglobulin-like receptor, subfamily B (with TM and ITIM domains), member 4	LILRB4	1,18
nemo-like kinase	NLK	1,18
angiopoietin like 5	ANGPTL5	1,18
creatine kinase, mitochondrial 2 (sarcomeric)	CKMT2	1,18
protease, serine 36	PRSS36	1,18
chemokine (C-C motif) ligand 8	CCL8	1,18
H1 histone family, member N, testis-specific	H1FNT	1,18
late cornified envelope 3D	LCE3D	1,18
mediator complex subunit 6	MED6	1,18
polymerase (DNA directed), gamma	POLG	1,18
wingless-type MMTV integration site family, member 5B	WNT5B	1,18
DDB1 and CUL4 associated factor 17	DCAF17	1,18
centrosomal protein 83kDa	CEP83	1,18
Spi-B transcription factor (Spi-1/PU.1 related)	SPIB	1,18
coiled-coil domain containing 6	CCDC6	1,18
inositol polyphosphate-5-phosphatase D	INPP5D	1,18
major histocompatibility complex, class II, DM beta	HLA-DMB	1,18
3-oxoacyl-ACP synthase, mitochondrial	OXSM	1,18
G protein-coupled receptor 65	GPR65	1,18
ORMDL sphingolipid biosynthesis regulator 1	ORMDL1	1,18
CD19 molecule	CD19	1,18
arginine/serine-rich coiled-coil 1	RSRC1	1,18
epididymal protein 3B	EDDM3B	1,18
zinc finger with UFM1-specific peptidase domain	ZUFSP	1,18
cytochrome P450, family 4, subfamily F, polypeptide 3	CYP4F3	1,18
O-sialoglycoprotein endopeptidase-like 1	OSGEPL1	1,18
unc-51 like kinase 3	ULK3	1,18
retinoblastoma binding protein 6	RBBP6	1,18
dual specificity phosphatase 11	DUSP11	1,18
family with sequence similarity 83, member A	FAM83A	1,18
salt-inducible kinase 1	SIK1	1,18
trafficking protein particle complex 4	TRAPPC4	1,18

guanine nucleotide binding protein (G protein), beta polypeptide 2	GNB2	1,18
ABI family, member 3 (NESH) binding protein	ABI3BP	1,18
myosin light chain 2	MYL2	1,18
G protein-coupled receptor 137C	GPR137C	1,18
solute carrier family 6 (neutral amino acid transporter), member 15	SLC6A15	1,18
neuropeptide Y receptor Y1	NPY1R	1,18
uncharacterized LOC388882; novel transcript	LOC388882; AP000345.1	1,18
NADH dehydrogenase (ubiquinone) 1 alpha subcomplex, 10, 42kDa	NDUFA10	1,18
family with sequence similarity 227, member B	FAM227B	1,18
Transcript Identified by AceView, Entrez Gene ID(s) 84256	FLYWCH1	1,18
regulator of microtubule dynamics 1	RMDN1	1,18
RB1-inducible coiled-coil 1	RB1CC1	1,18
inhibitor of growth family member 2	ING2	1,18
polypeptide N-acetylgalactosaminyltransferase-like 5	GALNTL5	1,18
poly(ADP-ribose) polymerase family member 8	PARP8	1,18
ribosomal protein L7-like 1	RPL7L1	1,18
leukocyte specific transcript 1	LST1	1,18
microfibrillar associated protein 3	MFAP3	1,18
deleted in azoospermia 2; deleted in azoospermia 3	DAZ2; DAZ3	1,18
store-operated calcium entry-associated regulatory factor	SARAF	1,18
WD repeat domain 62	WDR62	1,18
protein-L-isoaspartate (D-aspartate) O-methyltransferase	PCMT1	1,18
SLX1A-SULT1A3 readthrough (NMD candidate)	SLX1A-SULT1A3	1,18
ribonuclease, RNase A family, 7	RNASE7	1,18
late endosomal/lysosomal adaptor, MAPK and MTOR activator 5	LAMTOR5	1,18
ELAV like neuron-specific RNA binding protein 3	ELAVL3	1,18
shroom family member 3	SHROOM3	1,18
bridging integrator 2	BIN2	1,18
complement component 5a receptor 2	C5AR2	1,18
transmembrane protein 63C	TMEM63C	1,18
derlin 1	DERL1	1,18
zinc finger protein 212	ZNF212	1,18
MRGPRG antisense RNA 1	MRGPRG-AS1	1,18
RNA binding motif protein, Y-linked, family 1, member F; RNA binding motif protein, Y-linked, family 2, member F pseudogene; RNA binding motif protein, Y-linked, family 1, member J	RBMY1F; RBMY2FP; RBMY1J	1,18

stromal antigen 3-like 4 (pseudogene)	STAG3L4	1,18
cytotoxic T-lymphocyte-associated protein 4	CTLA4	1,18
SKI family transcriptional corepressor 1	SKOR1	1,18
Transcript Identified by AceView, Entrez Gene ID(s) 22849	CPEB3	1,18
fucokinase	FUK	1,18
claudin 2	CLDN2	1,17
inositol polyphosphate phosphatase-like 1	INPPL1	1,17
glycoprotein IX (platelet)	GP9	1,17
fragile X mental retardation, autosomal homolog 1	FXR1	1,17
T-box 6	TBX6	1,17
mannosidase, alpha, class 2C, member 1	MAN2C1	1,17
G antigen 12H; G antigen 12B; G antigen 12C	GAGE12H; GAGE12B; GAGE12C	1,17
G antigen 12D	GAGE12D	1,17
G antigen 12G; G antigen 12E	GAGE12G; GAGE12E	1,17
glutamate receptor, metabotropic 8	GRM8	1,17
chromosome 10 open reading frame 76	C10orf76	1,17
mitochondrial pyruvate carrier 1-like	MPC1L	1,17
archaelysin family metallopeptidase 2	AMZ2	1,17
Williams Beuren syndrome chromosome region 22	WBSCR22	1,17
Kruppel-like factor 16	KLF16	1,17
3-ketodihydrosphingosine reductase	KDSR	1,17
chromosome 19 open reading frame 73	C19orf73	1,17
Transcript Identified by AceView, Entrez Gene ID(s) 10673	TNFSF13B	1,17
REX1, RNA exonuclease 1 homolog-like 2, pseudogene	REXO1L2P	1,17
kelch domain containing 7B	KLHDC7B	1,17
transmembrane protein 88	TMEM88	1,17
SCO1 cytochrome c oxidase assembly protein	SCO1	1,17
zinc finger protein 341	ZNF341	1,17
long intergenic non-protein coding RNA 1587	LINC01587	1,17
ankyrin repeat and SOCS box containing 16	ASB16	1,17
up-regulated during skeletal muscle growth 5 homolog (mouse); microRNA 1307	USMG5; MIR1307	1,17
membrane-spanning 4-domains, subfamily A, member 14	MS4A14	1,17
solute carrier family 39 (zinc transporter), member 6	SLC39A6	1,17
solute carrier family 8 (sodium/calcium exchanger), member 2	SLC8A2	1,17
cell division cycle 123	CDC123	1,17
GLIS family zinc finger 2	GLIS2	1,17
fragile X mental retardation 1 neighbor	FMR1NB	1,17

Transcript Identified by AceView, Entrez Gene ID(s) 54443	ANLN	1,17
peptidyl arginine deiminase, type III	PADI3	1,17
keratin associated protein 5-3	KRTAP5-3	1,17
MOB kinase activator 3B	MOB3B	1,17
complexin 1	CPLX1	1,17
ribosomal protein S6 kinase, 90kDa, polypeptide 5	RPS6KA5	1,17
aprataxin and PNKP like factor	APLF	1,17
enhancer of mRNA decapping 3	EDC3	1,17
olfactory receptor, family 4, subfamily C, member 5 (gene/pseudogene)	OR4C5	1,17
coiled-coil domain containing 160	CCDC160	1,17
DEAD/H (Asp-Glu-Ala-Asp/His) box polypeptide 12, pseudogene; DEAD/H (Asp-Glu-Ala-Asp/His) box helicase 11	DDX12P; DDX11	1,17
G protein-coupled receptor 87	GPR87	1,17
zinc finger protein 613	ZNF613	1,17
protein kinase C, alpha	PRKCA	1,17
SPATA31 subfamily D, member 3	SPATA31D3	1,17
surfactant associated 3	SFTA3	1,17
tenomodulin	TNMD	1,17
X-linked Kx blood group related 9	XKR9	1,17
Hermansky-Pudlak syndrome 1; microRNA 4685	HPS1; MIR4685	1,17
leucine rich repeat containing 25	LRRC25	1,17
olfactory receptor, family 5, subfamily T, member 2	OR5T2	1,17
MLX interacting protein	MLXIP	1,17
PHD finger protein 7	PHF7	1,17
PRP40 homolog, pre-mRNA processing factor B	PRPF40B	1,17
Transcript Identified by AceView, Entrez Gene ID(s) 54758; 100129637	KLHDC4	1,17
chromosome X open reading frame 21	CXorf21	1,17
peptidase inhibitor 16	PI16	1,17
mutS homolog 3	MSH3	1,17
mitogen-activated protein kinase 8 interacting protein 1	MAPK8IP1	1,17
lymphotoxin beta (TNF superfamily, member 3)	LTB	1,17
netrin 1	NTN1	1,17
prostaglandin D2 receptor (DP)	PTGDR	1,17
REX1, RNA exonuclease 1 homolog-like 4, pseudogene	REXO1L4P	1,17
ATP binding cassette subfamily A member 2	ABCA2	1,17
vasoactive intestinal peptide receptor 1	VIPR1	1,17
Dab, mitogen-responsive phosphoprotein, homolog 2 (Drosophila)	DAB2	1,17
Rho guanine nucleotide exchange factor 10	ARHGEF10	1,17
PRAME family member 1	PRAMEF1	1,17

protein tyrosine phosphatase, receptor type, f polypeptide (PTPRF), interacting protein (liprin), alpha 4	PPFIA4	1,17
tubulin, beta 4B class IVb	TUBB4B	1,17
chromosome 5 open reading frame 42	C5orf42	1,17
adenosylhomocysteinase like 1	AHCYL1	1,17
olfactomedin like 2A	OLFML2A	1,17
PSMG3 antisense RNA 1 (head to head)	PSMG3-AS1	1,17
tumor necrosis factor receptor superfamily, member 18	TNFRSF18	1,17
glutamate receptor, metabotropic 6	GRM6	1,17
beta-1,4-N-acetyl-galactosaminyl transferase 2	B4GALNT2	1,17
ubiquitin specific peptidase like 1	USPL1	1,17
activation-induced cytidine deaminase	AICDA	1,17
family with sequence similarity 221, member B	FAM221B	1,17
NOP2/Sun domain family, member 5 pseudogene 2; NOP2/Sun domain family, member 5 pseudogene 1	NSUN5P2; NSUN5P1	1,17
delta(4)-desaturase, sphingolipid 1	DEGS1	1,17
branched chain ketoacid dehydrogenase kinase	BCKDK	1,17
olfactory receptor, family 51, subfamily H, member 1	OR51H1	1,17
cold inducible RNA binding protein	CIRBP	1,17
chromosome X open reading frame 65	CXorf65	1,17
strawberry notch homolog 2 (Drosophila)	SBNO2	1,17
RAB8B, member RAS oncogene family	RAB8B	1,17
olfactory receptor, family 51, subfamily Q, member 1 (gene/pseudogene)	OR51Q1	1,17
zinc finger protein 773	ZNF773	1,17
zinc finger and SCAN domain containing 4	ZSCAN4	1,17
keratin 85, type II	KRT85	1,17
coiled-coil domain containing 84	CCDC84	1,17
microtubule associated protein 2	MAP2	1,17
thromboxane A synthase 1 (platelet)	TBXAS1	1,17
zinc finger protein 135	ZNF135	1,17
keratin 18, type I	KRT18	1,17
chaperonin containing TCP1, subunit 4 (delta)	CCT4	1,17
colony stimulating factor 2 receptor, beta, low-affinity (granulocyte-macrophage)	CSF2RB	1,17
interleukin 33	IL33	1,17
thyrotropin-releasing hormone degrading enzyme	TRHDE	1,17
zinc finger protein 230	ZNF230	1,17
spermatogenic leucine zipper 1	SPZ1	1,17
trio Rho guanine nucleotide exchange factor	TRIO	1,17
small proline-rich protein 1A	SPRR1A	1,17
tudor domain containing 12	TDRD12	1,17
cyclin J-like	CCNJL	1,17
v-myc avian myelocytomatosis viral oncogene neuroblastoma derived homolog	MYCN	1,17

NCK-associated protein 5	NCKAP5	1,17
peroxisomal biogenesis factor 7	PEX7	1,17
annexin A8	ANXA8	1,17
astrotactin 1	ASTN1	1,17
STE20-related kinase adaptor beta	STRADB	1,17
kelch-like family member 7	KLHL7	1,17
uncharacterized LOC729220; novel transcript antisense to TAC4	FLJ45513; RP11-304F15.3	1,17
TRIM6-TRIM34 readthrough	TRIM6-TRIM34	1,17
WD repeat domain 31	WDR31	1,17
mitogen-activated protein kinase kinase kinase 2	MAP3K2	1,17
proline-rich transmembrane protein 2	PRRT2	1,17
maltase-glucoamylase 2 (putative)	MGAM2	1,17
consortin, connexin sorting protein	CNST	1,17
neuromedin B	NMB	1,17
glycoprotein V (platelet)	GP5	1,17
coiled-coil domain containing 181	CCDC181	1,17
golgin A6 family, member A	GOLGA6A	1,17
fibroblast growth factor 18	FGF18	1,17
proline-rich acidic protein 1	PRAP1	1,17
Memczak2013 ANTISENSE, CDS, coding, INTERNAL best transcript NM_001076552	ACSS2	1,17
keratin associated protein 5-2	KRTAP5-2	1,17
collagen, type V, alpha 2	COL5A2	1,17
N-terminal EF-hand calcium binding protein 1	NECAB1	1,17
receptor (G protein-coupled) activity modifying protein 1	RAMP1	1,17
Fanconi anemia core complex associated protein 20	FAAP20	1,17
potassium channel, calcium activated intermediate/small conductance subfamily N alpha, member 1	KCNN1	1,17
mitogen-activated protein kinase kinase kinase 19	MAP3K19	1,17
aminoacyl tRNA synthetase complex-interacting multifunctional protein 2	AIMP2	1,17
gamma-aminobutyric acid (GABA) A receptor, gamma 3	GABRG3	1,17
adrenocortical dysplasia homolog (mouse)	ACD	1,17
hepatocyte growth factor (hepapoietin A; scatter factor)	HGF	1,17
mesenteric estrogen-dependent adipogenesis	MEDAG	1,17
ribosomal protein S6 kinase, 90kDa, polypeptide 1	RPS6KA1	1,17
MAFF interacting protein (pseudogene); tektin 4 pseudogene 2	MAFIP; TEKT4P2	1,17
speedy/RINGO cell cycle regulator family member E4	SPDYE4	1,17
dynein, axonemal, heavy chain 6	DNAH6	1,17
tryptophan hydroxylase 1	TPH1	1,17

triggering receptor expressed on myeloid cells 2	TREM2	1,17
basic leucine zipper transcription factor, ATF-like 2	BATF2	1,17
copine V	CPNE5	1,17
early B-cell factor 2	EBF2	1,17
neuromedin B receptor	NMBR	1,17
nerve growth factor receptor (TNFRSF16) associated protein 1	NGFRAP1	1,17
adaptor-related protein complex 1, gamma 1 subunit	AP1G1	1,17
ATP/GTP binding protein-like 2	AGBL2	1,17
glutamate receptor, ionotropic, kainate 2	GRIK2	1,17
complement factor D (adipsin)	CFD	1,17
solute carrier family 30 (zinc transporter), member 9	SLC30A9	1,17
tetraspanin 7	TSPAN7	1,17
diacylglycerol O-acyltransferase 2-like 6	DGAT2L6	1,17
de-etiolated homolog 1 (Arabidopsis)	DET1	1,17
napsin A aspartic peptidase	NAPSA	1,17
Transcript Identified by AceView, Entrez Gene ID(s) 54715	A2BP1	1,17
FAST kinase domains 1	FASTKD1	1,17
ubiquitin-like modifier activating enzyme 1	UBA1	1,17
coiled-coil domain containing 136	CCDC136	1,17
leucine zipper protein 2	LUZP2	1,17
tigger transposable element derived 1; microRNA 5001	TIGD1; MIR5001	1,17
interleukin 2 receptor, beta	IL2RB	1,17
Transcript Identified by AceView, Entrez Gene ID(s) 8829	NRP1	1,17
AHA1, activator of heat shock 90kDa protein ATPase homolog 1 (yeast)	AHSA1	1,17
G protein-coupled receptor 149	GPR149	1,17
uncharacterized LOC101927668; Transcript Identified by AceView, Entrez Gene ID(s) 346389; novel transcript, sense overlapping to MACC1	LOC101927668; AC005062.2; MACC1	1,17
calcium channel, voltage-dependent, gamma subunit 8; microRNA 935	CACNG8; MIR935	1,17
insulin receptor substrate 2	IRS2	1,17
katanin p60 subunit A-like 1	KATNAL1	1,17
fucosyltransferase 1 (galactoside 2-alpha-L-fucosyltransferase, H blood group)	FUT1	1,17
wingless-type MMTV integration site family, member 5A	WNT5A	1,16
ADAM metallopeptidase domain 18	ADAM18	1,16
serum/glucocorticoid regulated kinase family, member 3	SGK3	1,16
microtubule-associated protein 1 light chain 3 alpha	MAP1LC3A	1,16
RecQ mediated genome instability 1	RMI1	1,16

myosin IA	MYO1A	1,16
trinucleotide repeat containing 6B	TNRC6B	1,16
neuroguidin, EIF4E binding protein	NGDN	1,16
olfactory receptor, family 1, subfamily S, member 1 (gene/pseudogene)	OR1S1	1,16
huntingtin interacting protein M	HYPM	1,16
sphingosine-1-phosphate phosphatase 2	SGPP2	1,16
pseudouridylate synthase 3	PUS3	1,16
acyl-CoA oxidase-like	ACOXL	1,16
EBNA1 binding protein 2	EBNA1BP2	1,16
thymidine kinase 2, mitochondrial	TK2	1,16
olfactory receptor, family 1, subfamily L, member 1	OR1L1	1,16
family with sequence similarity 9, member B	FAM9B	1,16
tripartite motif containing 77	TRIM77	1,16
uncharacterized LOC284365; uncharacterized LOC284365 [Source:EntrezGene;Acc:284365]; novel transcript	MGC45922; CTD-2568A17.5	1,16
BCL2-like 14 (apoptosis facilitator)	BCL2L14	1,16
glutamate rich 6B	ERICH6B	1,16
transketolase-like 2	TKTL2	1,16
zinc finger protein 43	ZNF43	1,16
Kirsten rat sarcoma viral oncogene homolog	KRAS	1,16
interferon, alpha 6	IFNA6	1,16
ZNF559-ZNF177 readthrough; zinc finger protein 177; zinc finger protein 559	ZNF559-ZNF177; ZNF177; ZNF559	1,16
gypsy retrotransposon integrase 1	GIN1	1,16
highly divergent homeobox	HDX	1,16
calcium/calmodulin-dependent protein kinase ID	CAMK1D	1,16
C-type lectin-like 1	CLECL1	1,16
tetraspanin 10	TSPAN10	1,16
La ribonucleoprotein domain family, member 6	LARP6	1,16
MyoD family inhibitor	MDFI	1,16
WD repeat domain 19	WDR19	1,16
sperm acrosome associated 5B	SPACA5B	1,16
acid phosphatase 2, lysosomal	ACP2	1,16
fatty acid amide hydrolase 2	FAAH2	1,16
Y box binding protein 1	YBX1	1,16
uncharacterized LOC100131303; novel transcript, antisense to DUS2L	LOC100131303; CTC-479C5.6	1,16
solute carrier family 25 (S-adenosylmethionine carrier), member 26	SLC25A26	1,16
aspartyl-tRNA synthetase 2, mitochondrial	DARS2	1,16
formyl peptide receptor 3	FPR3	1,16
myosin VIIB	MYO7B	1,16
macrophage scavenger receptor 1	MSR1	1,16

adenosine deaminase	ADA	1,16
gigaxonin; microRNA 4720	GAN; MIR4720	1,16
lymphoblastic leukemia associated hematopoiesis regulator 1	LYL1	1,16
melan-A	MLANA	1,16
trypsinogen-like pseudogene	TRY2P	1,16
SLAM family member 7	SLAMF7	1,16
NADH dehydrogenase (ubiquinone) flavoprotein 2, 24kDa	NDUFV2	1,16
regulatory factor X, 6	RFX6	1,16
discs, large homolog 2 (Drosophila)	DLG2	1,16
SHC (Src homology 2 domain containing) family, member 4	SHC4	1,16
microtubule-associated protein 7	MAP7	1,16
dentin matrix acidic phosphoprotein 1	DMP1	1,16
docking protein 5	DOK5	1,16
G protein-coupled receptor 143	GPR143	1,16
tripartite motif containing 72, E3 ubiquitin protein ligase	TRIM72	1,16
integrin alpha FG-GAP repeat containing 1	ITFG1	1,16
tetraspanin 18	TSPAN18	1,16
Jeck2013 ANTISENSE, CDS, coding, INTERNAL, intronic, OVCODE, OVEXON best transcript NM_005529	HSPG2	1,16
Homo sapiens interleukin 3 receptor, alpha (low affinity) (IL3RA), transcript variant 2, mRNA.; Homo sapiens interleukin 3 receptor, alpha (low affinity) (IL3RA), transcript variant 1, mRNA.; interleukin 3 receptor, alpha (low affinity) [Source:HGNC Symbol;Acc:HGNC:6012]; Homo sapiens interleukin 3 receptor, alpha (low affinity), mRNA (cDNA clone MGC:34174 IMAGE:5167281), complete cds.; Salzman2013 ANNOTATED, CDS, coding, INTERNAL, OVCODE, OVERLAPTX, OVEXON best transcript NM_002183	IL3RA	1,16
myosin VIIA	MYO7A	1,16
BEAN1 antisense RNA 1	BEAN1-AS1	1,16
N-6 adenine-specific DNA methyltransferase 1 (putative)	N6AMT1	1,16
glutathione S-transferase, C-terminal domain containing	GSTCD	1,16
ciliary rootlet coiled-coil, rootletin family member 2 [Source:HGNC Symbol;Acc:HGNC:51677]	CROCC2	1,16
olfactory receptor, family 4, subfamily N, member 4	OR4N4	1,16
nudix hydrolase 19	NUDT19	1,16
C2 calcium-dependent domain containing 4A	C2CD4A	1,16

uncharacterized LOC100287036; novel transcript, antisense to ANKRD11	LOC100287036; AC137932.6	1,16
Transcript Identified by AceView, Entrez Gene ID(s) 127435	PODN	1,16
single-stranded DNA binding protein 2	SSBP2	1,16
D-amino acid oxidase activator	DAOA	1,16
regulator of G-protein signaling 5	RGS5	1,16
chromosome 12 open reading frame 50	C12orf50	1,16
zinc finger and SCAN domain containing 22; microRNA 6806	ZSCAN22; MIR6806	1,16
Kell blood group, metallo-endopeptidase	KEL	1,16
integrin alpha 7	ITGA7	1,16
chromosome 5 open reading frame 49	C5orf49	1,16
zinc finger protein 512	ZNF512	1,16
insulin-like growth factor 2 mRNA binding protein 3	IGF2BP3	1,16
phosphatidylinositol transfer protein, beta	PITPNB	1,16
zinc finger protein 705B	ZNF705B	1,16
tyrosine 3-monooxygenase/tryptophan 5-monooxygenase activation protein, gamma	YWHAG	1,16
solute carrier family 25 (mitochondrial carrier, brain), member 14	SLC25A14	1,16
family with sequence similarity 65, member B	FAM65B	1,16
insulin-like 6	INSL6	1,16
lymphocyte expansion molecule	LEXM	1,16
Gse1 coiled-coil protein	GSE1	1,16
chromosome 15 open reading frame 54	C15orf54	1,16
paired-like homeobox 2a	PHOX2A	1,16
chromosome 17 open reading frame 97	C17orf97	1,16
guanine nucleotide binding protein (G protein), alpha transducing activity polypeptide 2	GNAT2	1,16
Transcript Identified by AceView, Entrez Gene ID(s) 114800	CCDC85A	1,16
protein kinase C, iota	PRKCI	1,16
Transcript Identified by AceView, Entrez Gene ID(s) 54715	A2BP1	1,16
ST6 (alpha-N-acetyl-neuraminyl-2,3-beta-galactosyl-1,3)-N-acetylgalactosaminide alpha-2,6-sialyltransferase 2	ST6GALNAC2	1,16
endomucin	EMCN	1,16
citron rho-interacting serine/threonine kinase; microRNA 1178	CIT; MIR1178	1,16
cleavage stimulation factor, 3 pre-RNA, subunit 2, tau variant	CSTF2T	1,16
Kruppel-like factor 14	KLF14	1,16
zinc finger protein 383	ZNF383	1,16

ankyrin repeat and sterile alpha motif domain containing 3	ANKS3	1,16
Sp1 transcription factor	SP1	1,16
acyl-CoA dehydrogenase, long chain	ACADL	1,16
t-complex 11, testis-specific	TCP11	1,16
Sin3A associated protein 130kDa	SAP130	1,16
Cas scaffolding protein family member 4	CASS4	1,16
zinc finger protein 436	ZNF436	1,16
glia maturation factor, beta	GMFB	1,16
heterogeneous nuclear ribonucleoprotein U-like 1	HNRNPUL1	1,16
olfactory receptor, family 10, subfamily T, member 2	OR10T2	1,16
tRNA methyltransferase 2 homolog B	TRMT2B	1,16
defensin, beta 134	DEFB134	1,16
paraneoplastic Ma antigen 3	PNMA3	1,16
EMI domain containing 1	EMID1	1,16
chordin	CHRD	1,16
PRAME family member 11	PRAMEF11	1,16
zinc finger protein 853	ZNF853	1,16
trefoil factor 2	TFF2	1,16
long intergenic non-protein coding RNA 116	LINC00116	1,16
steroid sulfatase (microsomal), isozyme S	STS	1,16
platelet-activating factor acetylhydrolase 1b, catalytic subunit 2 (30kDa)	PAFAH1B2	1,16
fascin actin-bundling protein 3, testicular	FSCN3	1,16
transient receptor potential cation channel, subfamily M, member 6	TRPM6	1,16
PRAME family member 4	PRAMEF4	1,16
claudin 3	CLDN3	1,16
zinc finger protein 205	ZNF205	1,16
protein phosphatase 1, regulatory subunit 16B	PPP1R16B	1,16
family with sequence similarity 26, member F	FAM26F	1,16
methyltransferase like 4	METTL4	1,16
zinc finger protein 222	ZNF222	1,16
lectin, galactoside-binding, soluble, 9	LGALS9	1,16
structural maintenance of chromosomes 6	SMC6	1,16
N-acetyltransferase 2 (arylamine N-acetyltransferase)	NAT2	1,16
olfactory receptor, family 2, subfamily A, member 5	OR2A5	1,16
sema domain, transmembrane domain (TM), and cytoplasmic domain, (semaphorin) 6A	SEMA6A	1,16
Rho GTPase activating protein 33	ARHGAP33	1,16
RNA binding motif protein, Y-linked, family 1, member E; RNA binding motif protein, Y-linked, family 1, member B	RBMY1E; RBMY1B	1,16
MOB kinase activator 1B	MOB1B	1,16
sprouty-related, EVH1 domain containing 1	SPRED1	1,16

ankyrin repeat and FYVE domain containing 1	ANKFY1	1,16
glycerol kinase 2	GK2	1,16
LY6/PLAUR domain containing 5	LYPD5	1,16
BRF2, RNA polymerase III transcription initiation factor 50 kDa subunit	BRF2	1,16
nuclear pore complex interacting protein family, member B5	NPIPB5	1,16
transmembrane inner ear	TMIE	1,16
zinc finger protein 670; zinc finger protein 695; ZNF670-ZNF695 readthrough (NMD candidate)	ZNF670; ZNF695; ZNF670-ZNF695	1,16
RAB39B, member RAS oncogene family	RAB39B	1,16
LTV1 ribosome biogenesis factor	LTV1	1,16
neurexin 2	NRXN2	1,16
mitogen-activated protein kinase kinase kinase 7	MAP3K7	1,16
transmembrane protein 189	TMEM189	1,16
chromosome 2 open reading frame 27B	C2orf27B	1,16
SAM and SH3 domain containing 3	SASH3	1,16
annexin A4	ANXA4	1,16
complement component 1, q subcomponent, A chain	C1QA	1,16
peptidylprolyl isomerase (cyclophilin)-like 6	PPIL6	1,16
family with sequence similarity 133, member A	FAM133A	1,16
envoplakin-like	EVPLL	1,16
cone-rod homeobox	CRX	1,16
ring finger protein 32	RNF32	1,16
sodium channel and clathrin linker 1	SCLT1	1,16
chromosome 10 open reading frame 105	C10orf105	1,16
epithelial cell transforming 2 like	ECT2L	1,16
X-prolyl aminopeptidase 3, mitochondrial	XPNPEP3	1,16
strawberry notch homolog 1 (Drosophila); microRNA 8072	SBNO1; MIR8072	1,16
polymerase (RNA) II (DNA directed) polypeptide J3	POLR2J3	1,16
cadherin 13	CDH13	1,16
Memczak2013 ANTISENSE, coding, INTERNAL, intronic best transcript NM_005308	GRK5	1,16
olfactory receptor, family 5, subfamily K, member 1	OR5K1	1,16
aldehyde dehydrogenase 1 family, member A3	ALDH1A3	1,16
vimentin-type intermediate filament associated coiled-coil protein	VMAC	1,16
Memczak2013 ANTISENSE, CDS, coding, INTERNAL best transcript NM_002473	MYH9	1,16
transmembrane protein 51	TMEM51	1,16
cancer susceptibility candidate 1	CASC1	1,16
apelin	APLN	1,16
WSC domain containing 1	WSCD1	1,16
NLR family, pyrin domain containing 13	NLRP13	1,16

transmembrane protein 139	TMEM139	1,16
kelch repeat and BTB (POZ) domain containing 2	KBTBD2	1,16
family with sequence similarity 47, member A	FAM47A	1,16
solute carrier family 39 (zinc transporter), member 13	SLC39A13	1,16
KIAA0355	KIAA0355	1,16
chemokine (C-C motif) receptor 10	CCR10	1,16
crystallin gamma D	CRYGD	1,16
desert hedgehog	DHH	1,16
erythrocyte membrane protein band 4.1-like 3	EPB41L3	1,15
autophagy related 14	ATG14	1,15
angiopoietin like 7	ANGPTL7	1,15
calcium channel, voltage-dependent, gamma subunit 6	CACNG6	1,15
transmembrane protein 244	TMEM244	1,15
recombination activating gene 1	RAG1	1,15
interferon-induced protein with tetratricopeptide repeats 5	IFIT5	1,15
UPF3 regulator of nonsense transcripts homolog B (yeast)	UPF3B	1,15
zinc finger family member 783	ZNF783	1,15
armadillo repeat containing 6	ARMC6	1,15
homeobox C11	HOXC11	1,15
protein kinase C, delta	PRKCD	1,15
neurotrophic tyrosine kinase, receptor, type 2	NTRK2	1,15
Memczak2013 ANTISENSE, CDS, coding, INTERNAL best transcript NM_182962	BIRC3	1,15
MTND2	ND2	1,15
S100 calcium binding protein A7 like 2	S100A7L2	1,15
zinc finger protein 26	ZNF26	1,15
proline rich 5 like	PRR5L	1,15
Memczak2013 ANTISENSE, coding, INTERNAL, intronic best transcript NM_014699	ZNF646	1,15
uncharacterized LOC100506388; Transcript Identified by AceView; novel transcript, atisense to RPH3AL	LOC100506388; RP11-1260E13.4; hosaru	1,15
betacellulin	BTC	1,15
transmembrane protein 230	TMEM230	1,15
H6 family homeobox 2	HMX2	1,15
haloacid dehalogenase-like hydrolase domain containing 3	HDHD3	1,15
KAT8 regulatory NSL complex subunit 1	KANSL1	1,15
basic charge, Y-linked, 2C; basic charge, Y-linked, 2	BPY2C; BPY2	1,15
N-ethylmaleimide-sensitive factor	NSF	1,15
nicotinamide riboside kinase 2	NMRK2	1,15
PDGFA associated protein 1	PDAP1	1,15
zinc finger protein 385C	ZNF385C	1,15

DnaJ (Hsp40) homolog, subfamily B, member 3	DNAJB3	1,15
transmembrane protein 217	TMEM217	1,15
glucocorticoid modulatory element binding protein 2	GMEB2	1,15
succinate dehydrogenase complex assembly factor 2	SDHAF2	1,15
Fas (TNFRSF6) binding factor 1	FBF1	1,15
HKR1, GLI-Kruppel zinc finger family member	HKR1	1,15
keratin 26, type I	KRT26	1,15
VAMP associated protein A	VAPA	1,15
EP400 N-terminal like	EP400NL	1,15
MANSC domain containing 4	MANSC4	1,15
microtubule associated monooxygenase, calponin and LIM domain containing 3	MICAL3	1,15
vacuolar protein sorting 25 homolog (S. cerevisiae)	VPS25	1,15
coiled-coil domain containing 59	CCDC59	1,15
autophagy related 16-like 1	ATG16L1	1,15
zinc finger protein 880	ZNF880	1,15
Transcript Identified by AceView, Entrez Gene ID(s) 114876	OSBPL1A	1,15
MAD2L1 binding protein	MAD2L1BP	1,15
fetal and adult testis expressed 1	FATE1	1,15
thromboxane A2 receptor	TBXA2R	1,15
carboxypeptidase X (M14 family), member 2	CPXM2	1,15
RAS p21 protein activator 4B; RAS p21 protein activator 4	RASA4B; RASA4	1,15
ribonuclease P/MRP 25kDa subunit	RPP25	1,15
UBX domain protein 8	UBXN8	1,15
eva-1 homolog C (C. elegans)	EVA1C	1,15
adenylate cyclase activating polypeptide 1 (pituitary)	ADCYAP1	1,15
paired immunoglobin-like type 2 receptor beta; stromal antigen 3-like 5 pseudogene; poliovirus receptor related immunoglobulin domain containing 2, pseudogene; microRNA 6840; STAG3L5P-PVRIG2P-PILRB readthrough	PILRB; STAG3L5P; PVRIG2P; MIR6840; STAG3L5P-PVRIG2P-PILRB	1,15
family with sequence similarity 189, member A2	FAM189A2	1,15
centrosomal protein 97kDa	CEP97	1,15
glutamate decarboxylase 2	GAD2	1,15
mediator complex subunit 31	MED31	1,15
olfactory receptor, family 4, subfamily A, member 47	OR4A47	1,15
asteroid homolog 1 (Drosophila)	ASTE1	1,15
ST3 beta-galactoside alpha-2,3-sialyltransferase 6	ST3GAL6	1,15
potassium channel tetramerization domain containing 13	KCTD13	1,15
EPH receptor A8	EPHA8	1,15
CD34 molecule	CD34	1,15
tubulin, alpha 3e	TUBA3E	1,15

olfactory receptor, family 10, subfamily G, member 7	OR10G7	1,15
RAB, member of RAS oncogene family-like 2B	RABL2B	1,15
Memczak2013 ANTISENSE, CDS, coding, INTERNAL best transcript NM_015356	SCRIB	1,15
membrane-spanning 4-domains, subfamily A, member 12	MS4A12	1,15
sterile alpha motif domain containing 12	SAMD12	1,15
Memczak2013 ANTISENSE, CDS, coding, INTERNAL, UTR3 best transcript NM_080668	CDCA5	1,15
SH3 and cysteine rich domain	STAC	1,15
olfactory receptor, family 5, subfamily V, member 1	OR5V1	1,15
protease, serine, 55	PRSS55	1,15
TraB domain containing 2A	TRABD2A	1,15
barrier to autointegration factor 2	BANF2	1,15
golgin A6 family, member D	GOLGA6D	1,15
chromosome 6 open reading frame 141	C6orf141	1,15
calcium homeostasis modulator 2	CALHM2	1,15
Zhang2013 ALT_ACCEPTOR, ALT_DONOR, coding, INTERNAL, intronic best transcript NM_004559	YBX1	1,15
family with sequence similarity 96, member B	FAM96B	1,15
galanin-like peptide	GALP	1,15
von Willebrand factor	VWF	1,15
tissue specific transplantation antigen P35B	TSTA3	1,15
Smith-Magenis syndrome chromosome region, candidate 5 (non-protein coding)	SMCR5	1,15
ceroid-lipofuscinosis, neuronal 3; nuclear pore complex interacting protein family, member B7	CLN3; NPIPB7	1,15
oxoglutarate (alpha-ketoglutarate) receptor 1	OXGR1	1,15
CD27 molecule	CD27	1,15
zinc finger and BTB domain containing 24	ZBTB24	1,15
MORN repeat containing 2	MORN2	1,15
membrane protein, palmitoylated 2	MPP2	1,15
mitochondrial carrier 2	MTCH2	1,15
lactamase, beta	LACTB	1,15
carcinoembryonic antigen-related cell adhesion molecule 20	CEACAM20	1,15
defensin, beta 125	DEFB125	1,15
coiled-coil domain containing 8	CCDC8	1,15
tubby like protein 3	TULP3	1,15
transcription elongation factor B polypeptide 3C-like 2; transcription elongation factor B polypeptide 3C-like	TCEB3CL2; TCEB3CL	1,15
phosphatidylinositol-4,5-bisphosphate 3-kinase, catalytic subunit alpha	PIK3CA	1,15
NK1 homeobox 1	NKX1-1	1,15
receptor (chemosensory) transporter protein 1	RTP1	1,15

G protein-coupled receptor 61	GPR61	1,15
ubiquitin conjugating enzyme E2I	UBE2I	1,15
spermatogenesis associated, serine-rich 1	SPATS1	1,15
GIMAP1-GIMAP5 readthrough; GTPase, IMAP family member 5; GTPase, IMAP family member 1	GIMAP1-GIMAP5; GIMAP5; GIMAP1	1,15
integrin, alpha M (complement component 3 receptor 3 subunit)	ITGAM	1,15
coiled-coil domain containing 171	CCDC171	1,15
sialidase 2 (cytosolic sialidase)	NEU2	1,15
malate dehydrogenase 2	MDH2	1,15
ATPase, Na+/K+ transporting, alpha 4 polypeptide	ATP1A4	1,15
proteasome (prosome, macropain) assembly chaperone 4	PSMG4	1,15
translocase of inner mitochondrial membrane 17 homolog B (yeast)	TIMM17B	1,15
PHD finger protein 3	PHF3	1,15
tryptophan hydroxylase 2	TPH2	1,15
CD5 molecule	CD5	1,15
transmembrane protein 59	TMEM59	1,15
transmembrane protein 246	TMEM246	1,15
spermatogenesis associated 32	SPATA32	1,15
calcium channel, voltage-dependent, beta 3 subunit	CACNB3	1,15
gastric intrinsic factor (vitamin B synthesis)	GIF	1,15
hematopoietic cell-specific Lyn substrate 1	HCLS1	1,15
tryptase delta 1	TPSD1	1,15
PRAME family member 9	PRAMEF9	1,15
cytoplasmic FMR1 interacting protein 1	CYFIP1	1,15
signaling threshold regulating transmembrane adaptor 1	SIT1	1,15
v-myc avian myelocytomatosis viral oncogene lung carcinoma derived homolog	MYCL	1,15
ribosomal protein S6 kinase, 90kDa, polypeptide 1	RPS6KA1	1,15
malignant T-cell amplified sequence 1	MCTS1	1,15
coiled-coil domain containing 172	CCDC172	1,15
coiled-coil domain containing 71	CCDC71	1,15
cell cycle exit and neuronal differentiation 1	CEND1	1,15
cortexin 2	CTXN2	1,15
PHD finger protein 21A	PHF21A	1,15
mitochondrial coiled-coil domain 1	MCCD1	1,15
glyoxalase domain containing 5	GLOD5	1,15
ATP binding cassette subfamily C member 9	ABCC9	1,15
coiled-coil domain containing 84	CCDC84	1,15
ankyrin repeat domain 53	ANKRD53	1,15
ferric-chelate reductase 1-like	FRRS1L	1,15
ureidopropionase, beta	UPB1	1,15

SH2B adaptor protein 2	SH2B2	1,15
shisa family member 3	SHISA3	1,15
guanine nucleotide binding protein (G protein), alpha activating activity polypeptide, olfactory type	GNAL	1,15
coiled-coil domain containing 63	CCDC63	1,15
chromosome 4 open reading frame 45	C4orf45	1,15
TRAF3 interacting protein 3	TRAF3IP3	1,15
transcription factor 23	TCF23	1,15
glycosyltransferase like domain containing 1	GTDC1	1,15
armadillo repeat containing 9	ARMC9	1,15
NADH dehydrogenase (ubiquinone) 1 alpha subcomplex, 12	NDUFA12	1,15
transcription factor 12	TCF12	1,15
transmembrane protease, serine 11A	TMPRSS11A	1,15
zinc finger, ZZ-type containing 3	ZZZ3	1,15
tyrosinase	TYR	1,15
Transcript Identified by AceView, Entrez Gene ID(s) 6453	ITSN1	1,15
uncharacterized LOC100129697; novel transcript, antisense to CBFA2T3	LOC100129697; RP11-830F9.6	1,15
glycosylated lysosomal membrane protein	GLMP	1,15
Transcript Identified by AceView, Entrez Gene ID(s) 215	ABCD1	1,15
autophagy related 16-like 1	ATG16L1	1,15
lymphocyte antigen 6 complex, locus G5B; casein kinase 2, beta polypeptide	LY6G5B; CSNK2B	1,15
SLIT and NTRK-like family, member 3	SLITRK3	1,15
interferon-induced protein with tetratricopeptide repeats 3	IFIT3	1,15
keratin 40, type I	KRT40	1,15
long intergenic non-protein coding RNA 1567	LINC01567	1,15
transducin-like enhancer of split 3	TLE3	1,15
SMG9 nonsense mediated mRNA decay factor	SMG9	1,15
forkhead box I1	FOXI1	1,15
follicle stimulating hormone, beta polypeptide	FSHB	1,15
GSK3B interacting protein	GSKIP	1,15
isoleucyl-tRNA synthetase	IARS	1,15
paraneoplastic Ma antigen family-like 1	PNMAL1	1,15
zinc finger protein 197	ZNF197	1,15
osteoclast associated, immunoglobulin-like receptor	OSCAR	1,15
meiosis specific with coiled-coil domain	MEIOC	1,15
DDB1 and CUL4 associated factor 4-like 2	DCAF4L2	1,15
cholinergic receptor, nicotinic alpha 6	CHRNA6	1,15
DEAD (Asp-Glu-Ala-Asp) box polypeptide 39B; small nucleolar RNA, C/D box 84	DDX39B; SNORD84	1,15
coiled-coil serine rich protein 2	CCSER2	1,15

leucine-rich repeat containing G protein-coupled receptor 5	LGR5	1,15
WAP four-disulfide core domain 2	WFDC2	1,15
proline rich Gla (G-carboxyglutamic acid) 2	PRRG2	1,15
tripartite motif containing 17	TRIM17	1,15
spalt-like transcription factor 4	SALL4	1,15
NADH dehydrogenase, subunit 6 (complex I)	ND6	1,15
eukaryotic translation initiation factor 3, subunit G	EIF3G	1,15
adhesion molecule with Ig-like domain 2	AMIGO2	1,15
ring finger protein 219	RNF219	1,15
ribosomal protein L3-like	RPL3L	1,15
MANSC domain containing 1	MANSC1	1,15
inducible T-cell co-stimulator ligand	ICOSLG	1,15
ATP binding cassette subfamily D member 2	ABCD2	1,15
ATP/GTP binding protein-like 4	AGBL4	1,15
translin-associated factor X	TSNAX	1,15
zinc finger protein 430	ZNF430	1,15
mutL homolog 1	MLH1	1,15
neurexin 1	NRXN1	1,15
zinc finger protein 878	ZNF878	1,15
adenylate kinase 5	AK5	1,15
interleukin 31 receptor A	IL31RA	1,15
zinc finger protein 71	ZNF71	1,15
integrin alpha E	ITGAE	1,15
Pim-3 proto-oncogene, serine/threonine kinase	PIM3	1,14
LON peptidase N-terminal domain and ring finger 2	LONRF2	1,14
TRAF2 and NCK interacting kinase	TNIK	1,14
membrane-spanning 4-domains, subfamily A, member 5	MS4A5	1,14
purinergic receptor P2X, ligand gated ion channel, 2	P2RX2	1,14
guanylate cyclase 2C	GUCY2C	1,14
A kinase (PRKA) anchor protein 14	AKAP14	1,14
leucine rich repeat containing 52	LRRC52	1,14
non imprinted in Prader-Willi/Angelman syndrome 1	NIPA1	1,14
leucine rich repeat containing 37, member A11, pseudogene	LRRC37A11P	1,14
membrane bound O-acyltransferase domain containing 1	MBOAT1	1,14
protein tyrosine phosphatase, non-receptor type 12	PTPN12	1,14
NOP2/Sun RNA methyltransferase family, member 2	NSUN2	1,14
RNA binding motif protein 34	RBM34	1,14
natural killer cell triggering receptor	NKTR	1,14
solute carrier family 12 (potassium/chloride transporter), member 7	SLC12A7	1,14

Memczak2013 ANTISENSE, coding, INTERNAL, intronic best transcript NM_001024808	BCL7A	1,14
gametogenetin binding protein 2	GGNBP2	1,14
collagen, type IV, alpha 4	COL4A4	1,14
regulating synaptic membrane exocytosis 1	RIMS1	1,14
interleukin 1 beta	IL1B	1,14
mucin 5B, oligomeric mucus/gel-forming	MUC5B	1,14
butyrophilin-like 3	BTNL3	1,14
zinc finger protein 558	ZNF558	1,14
dihydrouridine synthase 3-like	DUS3L	1,14
nucleoporin 93kDa	NUP93	1,14
ankyrin repeat domain 20 family, member A4	ANKRD20A4	1,14
H2A histone family, member Z	H2AFZ	1,14
Fc receptor-like 4	FCRL4	1,14
extended synaptotagmin-like protein 3	ESYT3	1,14
chromosome 19 open reading frame 48; small nucleolar RNA, C/D box 88B; small nucleolar RNA, C/D box 88C	C19orf48; SNORD88B; SNORD88C	1,14
cytochrome P450, family 2, subfamily A, polypeptide 13	CYP2A13	1,14
theg spermatid protein-like	THEGL	1,14
glypican 2	GPC2	1,14
Transcript Identified by AceView, Entrez Gene ID(s) 9229	DLGAP1	1,14
chromosome 16 open reading frame 92	C16orf92	1,14
SH2 domain containing 1B	SH2D1B	1,14
SPRY domain containing 4	SPRYD4	1,14
keratin associated protein 1-4	KRTAP1-4	1,14
early B-cell factor 3	EBF3	1,14
Transcript Identified by AceView, Entrez Gene ID(s) 79875	THSD4	1,14
hyaluronoglucosaminidase 4	HYAL4	1,14
nuclear VCP-like	NVL	1,14
gamma-aminobutyric acid (GABA) A receptor, alpha 5	GABRA5	1,14
signal-regulatory protein beta 1	SIRPB1	1,14
WD repeat and FYVE domain containing 1	WDFY1	1,14
SKI family transcriptional corepressor 2	SKOR2	1,14
sex comb on midleg-like 4 (Drosophila)	SCML4	1,14
proline-rich transmembrane protein 4	PRRT4	1,14
coiled-coil domain containing 106	CCDC106	1,14
developmental pluripotency associated 5	DPPA5	1,14
solute carrier organic anion transporter family, member 1C1	SLCO1C1	1,14
aurora kinase C	AURKC	1,14
SH3 domain binding glutamate-rich protein like	SH3BGRL	1,14
TLC domain containing 2	TLCD2	1,14

hemoglobin, theta 1	HBQ1	1,14
sialic acid binding Ig-like lectin 10	SIGLEC10	1,14
PRAME family member 18	PRAMEF18	1,14
N-acetylglucosamine-1-phosphate transferase, gamma subunit	GNPTG	1,14
secretoglobin, family 1D, member 4	SCGB1D4	1,14
colony stimulating factor 1 receptor	CSF1R	1,14
keratin 9, type I	KRT9	1,14
dentin sialophosphoprotein	DSPP	1,14
WBP2 N-terminal like	WBP2NL	1,14
nudix hydrolase 6	NUDT6	1,14
nitric oxide synthase 1 (neuronal)	NOS1	1,14
amyloid beta (A4) precursor-like protein 1	APLP1	1,14
golgin A6 family-like 6	GOLGA6L6	1,14
zinc finger protein 416	ZNF416	1,14
establishment of sister chromatid cohesion N-acetyltransferase 2	ESCO2	1,14
zinc finger protein 492	ZNF492	1,14
phosphatidylinositol glycan anchor biosynthesis class B	PIGB	1,14
centrosomal protein 152kDa	CEP152	1,14
growth hormone releasing hormone receptor	GHRHR	1,14
coiled-coil domain containing 151	CCDC151	1,14
gap junction protein beta 6	GJB6	1,14
death domain containing 1	DTHD1	1,14
sorting nexin 6	SNX6	1,14
family with sequence similarity 196, member B	FAM196B	1,14
diacylglycerol kinase, epsilon 64kDa	DGKE	1,14
protein phosphatase, EF-hand calcium binding domain 1	PPEF1	1,14
keratin associated protein 22-2	KRTAP22-2	1,14
zinc finger protein 595	ZNF595	1,14
GA binding protein transcription factor, beta subunit 1	GABPB1	1,14
ST20 antisense RNA 1	ST20-AS1	1,14
gap junction protein alpha 3	GJA3	1,14
EF-hand calcium binding domain 7; deleted in lymphocytic leukemia 2-like	EFCAB7; DLEU2L	1,14
ubiquitin-fold modifier conjugating enzyme 1	UFC1	1,14
NTPase, KAP family P-loop domain containing 1	NKPD1	1,14
transmembrane protein 119	TMEM119	1,14
ATPase, H+/K+ transporting, nongastric, alpha polypeptide	ATP12A	1,14
epoxide hydrolase 1, microsomal (xenobiotic)	EPHX1	1,14
eukaryotic translation initiation factor 4E	EIF4E	1,14
asunder spermatogenesis regulator	ASUN	1,14
interleukin 15	IL15	1,14
decapping mRNA 1B	DCP1B	1,14

solute carrier family 52 (riboflavin transporter), member 2	SLC52A2	1,14
T-box 19	TBX19	1,14
brain expressed X-linked 4	BEX4	1,14
ribonuclease, RNase A family, 3	RNASE3	1,14
ribosomal protein S6 kinase, 90kDa, polypeptide 1	RPS6KA1	1,14
small integral membrane protein 21	SMIM21	1,14
phospholipase A2, group IVE	PLA2G4E	1,14
matrix metallopeptidase 26	MMP26	1,14
lectin, galactoside-binding, soluble, 7; lectin, galactoside-binding, soluble, 7B	LGALS7; LGALS7B	1,14
proteasome 26S subunit, non-ATPase 5	PSMD5	1,14
V-set and transmembrane domain containing 2A; VSTM2A overlapping transcript 1	VSTM2A; VSTM2A-OT1	1,14
solute carrier family 7 (cationic amino acid transporter, y+ system), member 3	SLC7A3	1,14
tyrosine hydroxylase	TH	1,14
spermatogenesis and oogenesis specific basic helix-loop-helix 2	SOHLH2	1,14
essential meiotic structure-specific endonuclease 1	EME1	1,14
inositol(myo)-1(or 4)-monophosphatase 1	IMPA1	1,14
zinc finger, matrin-type 5	ZMAT5	1,14
conserved helix-loop-helix ubiquitous kinase	CHUK	1,14
RNA binding motif protein, Y-linked, family 1, member E; RNA binding motif protein, Y-linked, family 1, member D; RNA binding motif protein, Y-linked, family 1, member B	RBMY1E; RBMY1D; RBMY1B	1,14
LY6/PLAUR domain containing 4	LYPD4	1,14
proline rich 23 domain containing 2; proline rich 23 domain containing 1	PRR23D2; PRR23D1	1,14
fuzzy planar cell polarity protein	FUZ	1,14
STT3B, subunit of the oligosaccharyltransferase complex (catalytic)	STT3B	1,14
RUN domain containing 1	RUNDC1	1,14
NudC domain containing 3	NUDCD3	1,14
CWF19-like 1, cell cycle control (S. pombe); small nucleolar RNA, H/ACA box 12	CWF19L1; SNORA12	1,14
neurotensin	NTS	1,14
thymocyte selection associated family member 2	THEMIS2	1,14
gamma-aminobutyric acid (GABA) A receptor, beta 2	GABRB2	1,14
ribonuclease P/MRP 38kDa subunit	RPP38	1,14
olfactory receptor, family 4, subfamily P, member 4	OR4P4	1,14
Mab-21 domain containing 1	MB21D1	1,14
zinc finger protein 699	ZNF699	1,14
fibroblast growth factor 4	FGF4	1,14

plexin B3	PLXNB3	1,14
premature ovarian failure, 1B	POF1B	1,14
zinc finger protein 786	ZNF786	1,14
tetratricopeptide repeat domain 34	TTC34	1,14
olfactory receptor, family 8, subfamily H, member 3	OR8H3	1,14
Charcot-Leyden crystal protein pseudogene	LGALS17A	1,14
PR domain containing 9	PRDM9	1,14
chromosome 17 open reading frame 47	C17orf47	1,14
iron/zinc purple acid phosphatase-like protein; Iron/zinc purple acid phosphatase-like protein [Source:UniProtKB/Swiss-Prot;Acc:Q6ZNF0]; Transcript Identified by AceView, Entrez Gene ID(s) 390928	PAPL	1,14
testis expressed 43	TEX43	1,14
spectrin repeat containing, nuclear envelope 1	SYNE1	1,14
lipase maturation factor 2	LMF2	1,14
suppressor of cytokine signaling 3	SOCS3	1,14
APEX nuclease (multifunctional DNA repair enzyme) 1	APEX1	1,14
DnaJ (Hsp40) homolog, subfamily C, member 30	DNAJC30	1,14
C1D nuclear receptor corepressor	C1D	1,14
Ras-related GTP binding B	RRAGB	1,14
splicing factor 3a subunit 3	SF3A3	1,14
chondrosarcoma associated gene 1	CSAG1	1,14
zinc finger protein 675	ZNF675	1,14
POTE ankyrin domain family, member H	POTEH	1,14
pantothenate kinase 2	PANK2	1,14
lectin, galactoside-binding, soluble, 3 binding protein	LGALS3BP	1,14
5-aminoimidazole-4-carboxamide ribonucleotide formyltransferase/IMP cyclohydrolase	ATIC	1,14
Josephin domain containing 2	JOSD2	1,14
small nucleolar RNA, C/D box 89; ring finger protein 149	SNORD89; RNF149	1,14
aprataxin	APTX	1,14
nuclear pore complex interacting protein family, member B9	NPIPB9	1,14
family with sequence similarity 149, member B1	FAM149B1	1,14
family with sequence similarity 90, member A1	FAM90A1	1,14
HRAS-like suppressor	HRASLS	1,14
zinc finger CCCH-type containing 4	ZC3H4	1,14
histocompatibility (minor) HA-1	HMHA1	1,14
olfactory receptor, family 6, subfamily A, member 2	OR6A2	1,14
TAF1 RNA polymerase II, TATA box binding protein (TBP)-associated factor, 250kDa	TAF1	1,14
raftlin, lipid raft linker 1	RFTN1	1,14
aarF domain containing kinase 5	ADCK5	1,14
keratinocyte differentiation-associated protein	KRTDAP	1,14

coiled-coil domain containing 157	CCDC157	1,14
nuclear RNA export factor 2B; t-complex 11 family, X-linked 2	NXF2B; TCP11X2	1,14
microRNA 1247	MIR1247	1,14
COL18A1 antisense RNA 1	COL18A1-AS1	1,14
chromosome 9 open reading frame 62	C9orf62	1,14
neutrophil cytosolic factor 4	NCF4	1,14
laminin, gamma 2	LAMC2	1,14
SRY box 13	SOX13	1,14
RTF1 homolog, Paf1/RNA polymerase II complex component	RTF1	1,14
basic helix-loop-helix family, member e22	BHLHE22	1,14
GAS6 antisense RNA 1	GAS6-AS1	1,14
transmembrane protein 167A	TMEM167A	1,14
tubulin, alpha 3d	TUBA3D	1,14
family with sequence similarity 160, member B2	FAM160B2	1,14
potassium channel, inwardly rectifying subfamily J, member 8	KCNJ8	1,14
glutamate rich 1	ERICH1	1,14
NADH dehydrogenase, subunit 3 (complex I)	ND3	1,14
keratin associated protein 20-2	KRTAP20-2	1,14
zinc finger protein 714; vomeronasal 1 receptor 81 pseudogene	ZNF714; VN1R81P	1,14
transmembrane and tetratricopeptide repeat containing 2	TMTC2	1,14
guanine nucleotide binding protein (G protein), beta polypeptide 1	GNB1	1,14
chemokine (C-X-C motif) ligand 14	CXCL14	1,14
HYDIN, axonemal central pair apparatus protein	HYDIN	1,14
neuralized E3 ubiquitin protein ligase 1B	NEURL1B	1,14
LanC lantibiotic synthetase component C-like 2 (bacterial)	LANCL2	1,14
5-hydroxytryptamine (serotonin) receptor 3E, ionotropic	HTR3E	1,14
unconventional SNARE in the ER 1 homolog (S. cerevisiae)	USE1	1,14
oncostatin M	OSM	1,14
autophagy related 2B	ATG2B	1,14
zinc finger protein 157	ZNF157	1,14
keratin associated protein 21-2	KRTAP21-2	1,14
taste receptor, type 2, member 16	TAS2R16	1,13
RNA methyltransferase like 1	RNMTL1	1,13
spermatid perinuclear RNA binding protein; MIR600 host gene; microRNA 600	STRBP; MIR600HG; MIR600	1,13
trace amine associated receptor 3 (gene/pseudogene)	TAAR3	1,13

coiled-coil domain containing 159	CCDC159	1,13
dual specificity tyrosine-(Y)-phosphorylation regulated kinase 1B	DYRK1B	1,13
POTE ankyrin domain family, member G; POTE ankyrin domain family, member M	POTEG; POTEM	1,13
GDNF family receptor alpha 1	GFRA1	1,13
centrosome and spindle pole associated protein 1	CSPP1	1,13
synaptosome associated protein 23kDa	SNAP23	1,13
transcriptional regulating factor 1	TRERF1	1,13
centrosomal protein 126kDa	CEP126	1,13
shroom family member 4	SHROOM4	1,13
Rho GTPase activating protein 31	ARHGAP31	1,13
chromosome 8 open reading frame 48	C8orf48	1,13
proteasome activator subunit 4	PSME4	1,13
ribosomal protein L26-like 1	RPL26L1	1,13
anosmin 1	ANOS1	1,13
tetratricopeptide repeat domain 12	TTC12	1,13
WD repeat domain 5B	WDR5B	1,13
G protein-coupled receptor 20	GPR20	1,13
persephin	PSPN	1,13
cytokine-dependent hematopoietic cell linker	CLNK	1,13
vomeronasal 1 receptor 10 pseudogene	VN1R10P	1,13
calcium and integrin binding family member 4	CIB4	1,13
hydroxypyruvate isomerase (putative)	HYI	1,13
thyroid adenoma associated	THADA	1,13
ubiquitin specific peptidase 19	USP19	1,13
DnaJ (Hsp40) homolog, subfamily C, member 2	DNAJC2	1,13
putative UPF0607 protein ENSP00000383144	LOC100129307	1,13
amylase, alpha 2B (pancreatic); actin gamma 1 pseudogene 4	AMY2B; ACTG1P4	1,13
ring finger protein 215	RNF215	1,13
vestigial-like family member 1	VGLL1	1,13
serpin peptidase inhibitor, clade B (ovalbumin), member 3	SERPINB3	1,13
C-type lectin domain family 4, member D	CLEC4D	1,13
chromosome 8 open reading frame 86	C8orf86	1,13
inositol hexakisphosphate kinase 3	IP6K3	1,13
GS homeobox 1	GSX1	1,13
kelch domain containing 7A	KLHDC7A	1,13
adaptor-related protein complex 1, beta 1 subunit	AP1B1	1,13
tektin 3	TEKT3	1,13
DEAD (Asp-Glu-Ala-Asp) box helicase 56	DDX56	1,13
ligand dependent nuclear receptor corepressor	LCOR	1,13
SH2 domain containing 4B	SH2D4B	1,13
sulfotransferase family 2B member 1	SULT2B1	1,13

zinc finger protein 324	ZNF324	1,13
protease, serine 42	PRSS42	1,13
IZUMO family member 2	IZUMO2	1,13
MARVEL domain containing 1	MARVELD1	1,13
zinc finger, HIT-type containing 1	ZNHIT1	1,13
chromosome 6 open reading frame 47	C6orf47	1,13
angiopoietin like 3	ANGPTL3	1,13
cyclin-dependent kinase 8	CDK8	1,13
Memczak2013 ALT_ACCEPTOR, ALT_DONOR, coding, INTERNAL, intronic best transcript NM_001012478	U2AF2	1,13
ependymin related 1	EPDR1	1,13
zinc finger protein 239	ZNF239	1,13
gap junction protein beta 3	GJB3	1,13
ring finger and CHY zinc finger domain containing 1, E3 ubiquitin protein ligase	RCHY1	1,13
chaperonin containing TCP1, subunit 8 (theta)-like 2	CCT8L2	1,13
SRY box 14	SOX14	1,13
epsin 1	EPN1	1,13
codanin 1	CDAN1	1,13
hook microtubule-tethering protein 3	HOOK3	1,13
gamma-aminobutyric acid (GABA) A receptor, alpha 4	GABRA4	1,13
schlafen like 1	SLFNL1	1,13
proteasome subunit alpha 5	PSMA5	1,13
cyclin-dependent kinase 2 interacting protein	CINP	1,13
interleukin 3 receptor, alpha (low affinity)	IL3RA	1,13
zinc binding alcohol dehydrogenase domain containing 2	ZADH2	1,13
Memczak2013 ALT_ACCEPTOR, ALT_DONOR, coding, INTERNAL, intronic best transcript NM_032493	AP1M1	1,13
lens epithelial protein	LENEP	1,13
transmembrane channel like 4	TMC4	1,13
integrator complex subunit 12	INTS12	1,13
periostin, osteoblast specific factor	POSTN	1,13
armadillo repeat containing 8	ARMC8	1,13
WD repeat domain 92	WDR92	1,13
zinc finger protein 501 [Source:HGNC Symbol;Acc:HGNC:23717]	ZNF501	1,13
melatonin receptor 1B	MTNR1B	1,13
Zhang2013 ALT_ACCEPTOR, ALT_DONOR, coding, INTERNAL, intronic best transcript NM_001007	RPS4X	1,13
prostate transmembrane protein, androgen induced 1	PMEPA1	1,13
kinesin family member 25	KIF25	1,13
ankyrin repeat, SAM and basic leucine zipper domain containing 1	ASZ1	1,13
coiled-coil domain containing 162, pseudogene	CCDC162P	1,13

CCDC169-SOHLH2 readthrough	CCDC169-SOHLH2	1,13
leucine-rich repeat LGI family, member 2	LGI2	1,13
polymerase (DNA directed), alpha 2, accessory subunit	POLA2	1,13
synaptotagmin like 1	SYTL1	1,13
SET domain and mariner transposase fusion gene	SETMAR	1,13
methyl-CpG binding domain 4 DNA glycosylase	MBD4	1,13
PAN3 poly(A) specific ribonuclease subunit; RNA, U6 small nuclear 82, pseudogene	PAN3; RNU6-82P	1,13
delta(4)-desaturase, sphingolipid 2	DEGS2	1,13
polymerase (RNA) II (DNA directed) polypeptide E, 25kDa	POLR2E	1,13
G protein-coupled receptor 89B	GPR89B	1,13
ER membrane protein complex subunit 2	EMC2	1,13
Fc fragment of IgG, low affinity IIc, receptor for (CD32) (gene/pseudogene)	FCGR2C	1,13
sema domain, immunoglobulin domain (Ig), transmembrane domain (TM) and short cytoplasmic domain, (semaphorin) 4D	SEMA4D	1,13
sperm autoantigenic protein 17	SPA17	1,13
SEC22 homolog A, vesicle trafficking protein	SEC22A	1,13
sema domain, immunoglobulin domain (Ig), short basic domain, secreted, (semaphorin) 3F	SEMA3F	1,13
immature colon carcinoma transcript 1	ICT1	1,13
homeobox C12	HOXC12	1,13
proline rich 14-like	PRR14L	1,13
zinc finger protein 800	ZNF800	1,13
taste receptor, type 2, member 1	TAS2R1	1,13
laminin, beta 1	LAMB1	1,13
mitochondrial E3 ubiquitin protein ligase 1	MUL1	1,13
solute carrier family 30 (zinc transporter), member 7	SLC30A7	1,13
delta/notch like EGF repeat containing	DNER	1,13
N(alpha)-acetyltransferase 50, NatE catalytic subunit	NAA50	1,13
phosphatase, orphan 1	PHOSPHO1	1,13
zinc finger protein 502	ZNF502	1,13
family with sequence similarity 206, member A	FAM206A	1,13
coiled-coil domain containing 67	CCDC67	1,13
potassium channel, two pore domain subfamily K, member 16	KCNK16	1,13
olfactory receptor, family 5, subfamily H, member 2	OR5H2	1,13
malic enzyme 1, NADP(+)-dependent, cytosolic	ME1	1,13
syntaxin 19	STX19	1,13
biorientation of chromosomes in cell division 1-like 2	BOD1L2	1,13
small proline-rich protein 3	SPRR3	1,13
SWI/SNF related, matrix associated, actin dependent regulator of chromatin, subfamily a, member 5	SMARCA5	1,13

keratin 7, type II	KRT7	1,13
bone morphogenetic protein 7	BMP7	1,13
olfactory receptor, family 4, subfamily N, member 4; olfactory receptor, family 4, subfamily N, member 3 pseudogene; olfactory receptor, family 4, subfamily M, member 2	OR4N4; OR4N3P; OR4M2	1,13
keratin associated protein 27-1	KRTAP27-1	1,13
collagen-like tail subunit (single strand of homotrimer) of asymmetric acetylcholinesterase	COLQ	1,13
glycoprotein hormone beta 5	GPHB5	1,13
zinc finger protein 224	ZNF224	1,13
dynactin 4 (p62)	DCTN4	1,13
AI894139 pseudogene; Transcript Identified by AceView, Entrez Gene ID(s) 155060	LOC155060; ZNF783	1,13
potassium channel, voltage gated Shab related subfamily B, member 2	KCNB2	1,13
RAS p21 protein activator 4	RASA4	1,13
MAS-related GPR, member G	MRGPRG	1,13
glutathione S-transferase theta 2 (gene/pseudogene)	GSTT2	1,13
frizzled class receptor 2	FZD2	1,13
SLIT-ROBO Rho GTPase activating protein 3	SRGAP3	1,13
cytochrome P450, family 4, subfamily F, polypeptide 2	CYP4F2	1,13
proline and serine rich 1	PROSER1	1,13
cytochrome P450, family 2, subfamily D, polypeptide 6	CYP2D6	1,13
family with sequence similarity 204, member A	FAM204A	1,13
microfibrillar associated protein 4	MFAP4	1,13
tripartite motif containing 49D2; tripartite motif containing 49D1	TRIM49D2; TRIM49D1	1,13
growth arrest-specific 1	GAS1	1,13
Transcript Identified by AceView, Entrez Gene ID(s) 4798	NFRKB	1,13
glutamate receptor, ionotropic, N-methyl-D-aspartate 3A	GRIN3A	1,13
Memczak2013 ALT_ACCEPTOR, ALT_DONOR, coding, INTERNAL, intronic best transcript NM_032792	ZBTB45	1,13
pappalysin 2	PAPPA2	1,13
coiled-coil domain containing 153	CCDC153	1,13
TBC1 domain family member-like; TBC1 domain family, member 3D [Source:HGNC Symbol;Acc:HGNC:28944]	LOC101060389; TBC1D3D	1,13
Bardet-Biedl syndrome 12	BBS12	1,13
homeobox D3	HOXD3	1,13
solute carrier family 24 (sodium/potassium/calcium exchanger), member 5	SLC24A5	1,13
lin-9 DREAM MuvB core complex component	LIN9	1,13
WAS/WASL interacting protein family, member 3	WIPF3	1,13

tRNA methyltransferase 2 homolog A	TRMT2A	1,13
DIRAS family, GTP-binding RAS-like 3	DIRAS3	1,13
eukaryotic translation initiation factor 3, subunit K	EIF3K	1,13
phosphatidylinositol-4-phosphate 5-kinase-like 1	PIP5KL1	1,13
G protein-coupled receptor 17	GPR17	1,13
inositol polyphosphate-4-phosphatase type I A	INPP4A	1,13
lactate dehydrogenase B	LDHB	1,13
leukocyte immunoglobulin-like receptor, subfamily B (with TM and ITIM domains), member 3	LILRB3	1,13
solute carrier family 22 (organic cation transporter), member 1	SLC22A1	1,13
acyloxyacyl hydrolase (neutrophil)	AOAH	1,13
solute carrier family 5 (glucose activated ion channel), member 4	SLC5A4	1,13
threonine synthase-like 2	THNSL2	1,13
brain and acute leukemia, cytoplasmic	BAALC	1,13
vacuolar protein sorting 36 homolog (S. cerevisiae)	VPS36	1,13
olfactory receptor, family 5, subfamily AN, member 1	OR5AN1	1,13
arylacetamide deacetylase-like 2	AADACL2	1,13
sperm-tail PG-rich repeat containing 2	STPG2	1,13
Memczak2013 ANTISENSE, CDS, coding, INTERNAL best transcript NM_001145304	KIAA1683	1,13
myosin, heavy chain 3, skeletal muscle, embryonic	MYH3	1,13
6-pyruvoyltetrahydropterin synthase	PTS	1,13
ovochymase 2 (gene/pseudogene)	OVCH2	1,13
craniofacial development protein 1	CFDP1	1,13
threonyl-tRNA synthetase	TARS	1,13
killer cell lectin-like receptor subfamily G, member 2	KLRG2	1,13
ROS proto-oncogene 1 , receptor tyrosine kinase	ROS1	1,13
ribosomal protein S6 kinase, 90kDa, polypeptide 1	RPS6KA1	1,13
FYVE, RhoGEF and PH domain containing 2	FGD2	1,13
zinc finger and SCAN domain containing 18	ZSCAN18	1,13
sperm acrosome associated 5	SPACA5	1,13
extracellular leucine-rich repeat and fibronectin type III domain containing 1	ELFN1	1,13
SH3-domain GRB2-like 1	SH3GL1	1,13
aquaporin 10	AQP10	1,13
G protein-coupled receptor 27	GPR27	1,13
EPS8-like 1	EPS8L1	1,13
retinoic acid early transcript 1L	RAET1L	1,13
protein kinase N3	PKN3	1,13

POTE ankyrin domain family member D-like; Synthetic construct Homo sapiens clone IMAGE:100066427, MGC:195564 POTE ankyrin domain family, member D (POTED) mRNA, encodes complete protein.; uncharacterized protein LOC100288966 [Source:RefSeq peptide;Acc:NP_001244291]	LOC100288966; POTED; BX072566.1	1,13
HIV-1 Tat interactive protein 2	HTATIP2	1,13
transmembrane protein 71	TMEM71	1,13
histocompatibility (minor) serpin domain containing	HMSD	1,13
DnaJ (Hsp40) homolog, subfamily C, member 15	DNAJC15	1,13
uncharacterized LOC442028; Transcript Identified by AceView, Entrez Gene ID(s) 729171	LOC442028; ANKRD20B	1,13
papilin, proteoglycan-like sulfated glycoprotein	PAPLN	1,13
olfactory receptor, family 10, subfamily Q, member 1	OR10Q1	1,13
glutamate receptor, ionotropic, N-methyl D-aspartate 2A	GRIN2A	1,13
zinc finger and BTB domain containing 42	ZBTB42	1,13
family with sequence similarity 71, member D	FAM71D	1,12
synaptopodin 2-like	SYNPO2L	1,12
Bardet-Biedl syndrome 10	BBS10	1,12
olfactory receptor, family 3, subfamily A, member 1	OR3A1	1,12
PRKC, apoptosis, WT1, regulator	PAWR	1,12
ankyrin repeat domain 13C	ANKRD13C	1,12
ZNF32 antisense RNA 3	ZNF32-AS3	1,12
zinc finger protein 688	ZNF688	1,12
Zhang2013 ALT_ACCEPTOR, ALT_DONOR, coding, INTERNAL, intronic best transcript NM_133374	ZNF618	1,12
S100 calcium binding protein B	S100B	1,12
undifferentiated embryonic cell transcription factor 1	UTF1	1,12
von Willebrand factor A domain containing 2	VWA2	1,12
ATPase, Na+/K+ transporting, alpha 2 polypeptide	ATP1A2	1,12
uncharacterized LOC339803; Transcript Identified by AceView, Entrez Gene ID(s) 339803; novel transcript	LOC339803; AC016747.3	1,12
chromosome 9 open reading frame 152	C9orf152	1,12
cysteine-rich secretory protein LCCL domain containing 2	CRISPLD2	1,12
GTPase activating protein (SH3 domain) binding protein 2	G3BP2	1,12
cyclin-dependent kinase inhibitor 1C (p57, Kip2)	CDKN1C	1,12
olfactory receptor, family 10, subfamily J, member 4 (gene/pseudogene) [Source:HGNC Symbol;Acc:HGNC:15408]	OR10J4	1,12
serine peptidase inhibitor, Kunitz type, 3	SPINT3	1,12
VPS35 retromer complex component	VPS35	1,12
ring finger protein 113B	RNF113B	1,12

protein tyrosine phosphatase, receptor type, A; vacuolar protein sorting 16 homolog (S. cerevisiae)	PTPRA; VPS16	1,12
potassium channel, voltage gated subfamily E regulatory beta subunit 2	KCNE2	1,12
cystatin 11	CST11	1,12
solute carrier family 5 (sodium/choline cotransporter), member 7	SLC5A7	1,12
zinc finger protein 322	ZNF322	1,12
CD70 molecule	CD70	1,12
neurensin 2	NRSN2	1,12
neuromedin U	NMU	1,12
leucine-rich repeats and transmembrane domains 2	LRTM2	1,12
chromosome 4 open reading frame 33	C4orf33	1,12
jumonji domain containing 8	JMJD8	1,12
cytochrome P450, family 2, subfamily D, polypeptide 6	CYP2D6	1,12
olfactory receptor, family 2, subfamily H, member 1	OR2H1	1,12
Transcript Identified by AceView, Entrez Gene ID(s) 3069	HDLBP	1,12
ZW10 interacting kinetochore protein	ZWINT	1,12
GLI family zinc finger 1	GLI1	1,12
ArfGAP with RhoGAP domain, ankyrin repeat and PH domain 3	ARAP3	1,12
homeobox B1	HOXB1	1,12
CUGBP, Elav-like family member 6	CELF6	1,12
potassium channel, voltage gated eag related subfamily H, member 5	KCNH5	1,12
upper zone of growth plate and cartilage matrix associated	UCMA	1,12
solute carrier family 37, member 3	SLC37A3	1,12
upstream transcription factor 2, c-fos interacting	USF2	1,12
C-type lectin domain family 19, member A	CLEC19A	1,12
glutaminyl-tRNA synthase (glutamine-hydrolyzing)-like 1	QRSL1	1,12
defensin, beta 104A; defensin, beta 104B	DEFB104A; DEFB104B	1,12
Transcript Identified by AceView, Entrez Gene ID(s) 148753; novel transcript	RP11-12M5.1; FAM163A	1,12
leucine rich repeat containing 14B	LRRC14B	1,12
neurexophilin 4	NXPH4	1,12
mitogen-activated protein kinase binding protein 1	MAPKBP1	1,12
leucine rich repeat containing 37, member A2	LRRC37A2	1,12
forkhead-associated (FHA) phosphopeptide binding domain 1	FHAD1	1,12
cytochrome b5 reductase 2	CYB5R2	1,12
polypeptide N-acetylgalactosaminyltransferase 14	GALNT14	1,12

ubiquinol-cytochrome c reductase complex III subunit VII	UQCRQ	1,12
autophagy related 16-like 1	ATG16L1	1,12
MAGE family member B16	MAGEB16	1,12
heat shock 70kDa protein 9 (mortalin)	HSPA9	1,12
cyclin G associated kinase	GAK	1,12
late cornified envelope 3C	LCE3C	1,12
prominin 2	PROM2	1,12
doublecortin domain containing 2B	DCDC2B	1,12
Transcript Identified by AceView, Entrez Gene ID(s) 816	CAMK2B	1,12
family with sequence similarity 231, member C; family with sequence similarity 231, member A	FAM231C; FAM231A	1,12
tumor necrosis factor receptor superfamily, member 4	TNFRSF4	1,12
Transcript Identified by AceView, Entrez Gene ID(s) 9697	TRAM2	1,12
zinc finger CCCH-type containing 10	ZC3H10	1,12
Transcript Identified by AceView, Entrez Gene ID(s) 100133204; Salzman2013 ANTISENSE, CDS, coding, downstream_end, intronic, OVCODE, OVERLAPTX, OVEXON, UTR3 best transcript NM_032303; chromosome 9 open reading frame 147; chromosome 9 open reading frame 147 [Source:HGNC Symbol;Acc:HGNC:31438]	C9orf147; HSDL2	1,12
WD repeat and HMG-box DNA binding protein 1	WDHD1	1,12
hydroxymethylbilane synthase	HMBS	1,12
phosphatidylinositol glycan anchor biosynthesis class A	PIGA	1,12
crystallin gamma A	CRYGA	1,12
potassium channel subfamily M regulatory beta subunit 2	KCNMB2	1,12
La ribonucleoprotein domain family, member 4B	LARP4B	1,12
ret finger protein-like 2	RFPL2	1,12
zinc finger protein 766; microRNA 643	ZNF766; MIR643	1,12
microRNA 7162; golgin A2 pseudogene 6; mitochondrial poly(A) polymerase	MIR7162; GOLGA2P6; MTPAP	1,12
B-cell CLL/lymphoma 11A (zinc finger protein)	BCL11A	1,12
BH3-like motif containing, cell death inducer; mir-100-let-7a-2 cluster host gene	BLID; MIR100HG	1,12
NIMA-related kinase 11	NEK11	1,12
ATPase, Cu++ transporting, alpha polypeptide	ATP7A	1,12
protease, serine, 12 (neurotrypsin, motopsin)	PRSS12	1,12
zinc finger protein 551	ZNF551	1,12
NOP2/Sun domain family, member 5 pseudogene 1	NSUN5P1	1,12
phospholipase A2, group IVF	PLA2G4F	1,12

ADAM metallopeptidase with thrombospondin type 1 motif 16	ADAMTS16	1,12
death-domain associated protein	DAXX	1,12
urotensin 2B	UTS2B	1,12
splA/ryanodine receptor domain and SOCS box containing 4	SPSB4	1,12
SPATA31 subfamily D, member 4	SPATA31D4	1,12
formyl peptide receptor 2	FPR2	1,12
MAP/microtubule affinity-regulating kinase 4	MARK4	1,12
synaptotagmin XII	SYT12	1,12
chromosome 9 open reading frame 142	C9orf142	1,12
chromogranin A	CHGA	1,12
G protein-coupled receptor 89A	GPR89A	1,12
Rho GTPase activating protein 10	ARHGAP10	1,12
human immunodeficiency virus type I enhancer binding protein 3	HIVEP3	1,12
regulating synaptic membrane exocytosis 3	RIMS3	1,12
apoptosis resistant E3 ubiquitin protein ligase 1	AREL1	1,12
solute carrier family 44, member 5	SLC44A5	1,12
potassium channel, voltage gated shaker related subfamily A, member 7	KCNA7	1,12
thiamine triphosphatase	THTPA	1,12
Transcript Identified by AceView, Entrez Gene ID(s) 8328; novel transcript	RP11-295G24.4; GFI1B	1,12
olfactory receptor, family 4, subfamily A, member 16	OR4A16	1,12
FAST kinase domains 2	FASTKD2	1,12
PRAME family member 25	PRAMEF25	1,12
ADP-ribose/CDP-alcohol diphosphatase, manganese-dependent	ADPRM	1,12
retinoic acid early transcript 1G	RAET1G	1,12
olfactory receptor, family 51, subfamily B, member 6	OR51B6	1,12
solute carrier family 9, subfamily B (NHA1, cation proton antiporter 1), member 1	SLC9B1	1,12
protease, serine 46	PRSS46	1,12
peptidylglycine alpha-amidating monooxygenase	PAM	1,12
B-cell CLL/lymphoma 7A	BCL7A	1,12
ribosomal L1 domain containing 1	RSL1D1	1,12
patched 1	PTCH1	1,12
protein phosphatase 1, regulatory subunit 21	PPP1R21	1,12
olfactory receptor, family 51, subfamily D, member 1	OR51D1	1,12
REM2 and RAB-like small GTPase 1	RSG1	1,12
anaphase promoting complex subunit 7	ANAPC7	1,12
SH2 domain containing 6	SH2D6	1,12
olfactory receptor, family 51, subfamily I, member 2	OR51I2	1,12

dpy-30 histone methyltransferase complex regulatory subunit	DPY30	1,12
proteasome 26S subunit, non-ATPase 11	PSMD11	1,12
solute carrier family 17 (organic anion transporter), member 3; histone cluster 1, H2a, pseudogene 2	SLC17A3; HIST1H2APS2	1,12
zinc finger protein 621	ZNF621	1,12
peptidoglycan recognition protein 4	PGLYRP4	1,12
ST6 (alpha-N-acetyl-neuraminyl-2,3-beta-galactosyl-1,3)-N-acetylgalactosaminide alpha-2,6-sialyltransferase 1	ST6GALNAC1	1,12
beaded filament structural protein 2, phakinin	BFSP2	1,12
hydroxysteroid (11-beta) dehydrogenase 1-like; ribosomal protein L36	HSD11B1L; RPL36	1,12
kelch repeat and BTB (POZ) domain containing 12	KBTBD12	1,12
MT-RNR2-like 3	MTRNR2L3	1,12
doublecortin domain containing 1	DCDC1	1,12
armadillo repeat containing 7	ARMC7	1,12
RAB2A, member RAS oncogene family	RAB2A	1,12
chromosome 8 open reading frame 34	C8orf34	1,12
homeobox A1	HOXA1	1,12
serine/arginine repetitive matrix 4	SRRM4	1,12
cutA divalent cation tolerance homolog (E. coli)	CUTA	1,12
zinc finger protein 35	ZNF35	1,12
elongation factor RNA polymerase II-like 3	ELL3	1,12
olfactory receptor, family 52, subfamily J, member 3	OR52J3	1,12
chondroitin polymerizing factor 2; microRNA 671	CHPF2; MIR671	1,12
mesoderm posterior bHLH transcription factor 1	MESP1	1,12
trace amine associated receptor 9 (gene/pseudogene)	TAAR9	1,12
olfactory receptor, family 1, subfamily L, member 4	OR1L4	1,12
zinc finger protein 121	ZNF121	1,12
testis expressed 26	TEX26	1,12
prokineticin 1	PROK1	1,12
myosin, heavy chain 8, skeletal muscle, perinatal	MYH8	1,12
solute carrier family 35, member F1	SLC35F1	1,12
chromosome 19 open reading frame 57	C19orf57	1,12
SHC SH2-domain binding protein 1-like	SHCBP1L	1,12
mitogen-activated protein kinase kinase kinase 5	MAP3K5	1,12
olfactomedin 2	OLFM2	1,12
histone deacetylase 10	HDAC10	1,12
urotensin 2 receptor	UTS2R	1,12
plexin domain containing 2	PLXDC2	1,12
macrophage scavenger receptor 1	MSR1	1,12
apoptotic chromatin condensation inducer 1	ACIN1	1,12
ankyrin repeat and ubiquitin domain containing 1	ANKUB1	1,12
spermatogenesis associated 20	SPATA20	1,12

methyl-CpG binding domain protein 2	MBD2	1,12
tRNA methyltransferase 10B	TRMT10B	1,12
integrin linked kinase	ILK	1,12
late cornified envelope 1A	LCE1A	1,12
Fc receptor-like 1	FCRL1	1,12
CDP-diacylglycerol synthase 1	CDS1	1,12
transglutaminase 6	TGM6	1,12
regulating synaptic membrane exocytosis 4	RIMS4	1,12
leucine rich repeat containing 29	LRRC29	1,12
leukocyte immunoglobulin-like receptor, subfamily A (with TM domain), member 6	LILRA6	1,12
Transcript Identified by AceView, Entrez Gene ID(s) 3670	ISL1	1,12
NFKB activating protein-like	NKAPL	1,12
neurofilament, heavy polypeptide	NEFH	1,12
achaete-scute family bHLH transcription factor 5	ASCL5	1,12
family with sequence similarity 184, member A	FAM184A	1,12
zinc finger protein 417	ZNF417	1,12
protein phosphatase 1, regulatory subunit 3G	PPP1R3G	1,12
alkB homolog 1, histone H2A dioxygenase	ALKBH1	1,12
phosducin like 2	PDCL2	1,12
Ras suppressor protein 1	RSU1	1,12
growth differentiation factor 3	GDF3	1,12
zinc finger, BED-type containing 1	ZBED1	1,12
dystrobrevin beta	DTNB	1,12
plastin 3	PLS3	1,12
pleckstrin homology domain containing, family G (with RhoGef domain) member 5	PLEKHG5	1,12
chromosome 2 open reading frame 83	C2orf83	1,12
enamelin	ENAM	1,12
spermatid maturation 1	SPEM1	1,12
amelotin	AMTN	1,12
family with sequence similarity 171, member B	FAM171B	1,12
synovial sarcoma translocation gene on chromosome 18-like 1	SS18L1	1,12
leucine-rich pentatricopeptide repeat containing	LRPPRC	1,12
family with sequence similarity 27-like	FAM27L	1,12
thyroid hormone receptor interactor 12	TRIP12	1,12
transmembrane protein 150B	TMEM150B	1,12
FLJ33360 protein; FLJ33360 protein [Source:EntrezGene;Acc:401172]; putative novel transcript	FLJ33360; CTD-2324F15.2	1,12
phosphoglucomutase 5	PGM5	1,12
chymotrypsin C (caldecrin)	CTRC	1,12
olfactory receptor, family 1, subfamily L, member 8	OR1L8	1,12

bromodomain containing 8	BRD8	1,12
forkhead-associated (FHA) phosphopeptide binding domain 1	FHAD1	1,12
vanin 2	VNN2	1,12
clock circadian regulator	CLOCK	1,12
matrix metallopeptidase 12	MMP12	1,12
matrix metallopeptidase 21	MMP21	1,11
CD2-associated protein	CD2AP	1,11
acyl-CoA synthetase bubblegum family member 1	ACSBG1	1,11
coiled-coil domain containing 84	CCDC84	1,11
coiled-coil domain containing 141	CCDC141	1,11
polymerase (RNA) II (DNA directed) polypeptide A, 220kDa	POLR2A	1,11
THO complex 3	THOC3	1,11
dehydrogenase/reductase (SDR family) member 7	DHRS7	1,11
Kazal-type serine peptidase inhibitor domain 1	KAZALD1	1,11
Homo sapiens TP53 target 3D (TP53TG3D), transcript variant 2, non-coding RNA.; TP53-target gene 3 protein; Homo sapiens TP53 target 3, mRNA (cDNA clone MGC:119889 IMAGE:40015196), complete cds.; Homo sapiens TP53 target 3, mRNA (cDNA clone MGC:119888 IMAGE:40015195), complete cds.	TP53TG3D; LOC102723655; TP53TG3; AC136612.1	1,11
myeloperoxidase	MPO	1,11
cilia and flagella associated protein 77	CFAP77	1,11
Epstein-Barr virus induced 3	EBI3	1,11
ameloblastin	AMBN	1,11
family with sequence similarity 134, member B	FAM134B	1,11
UMODL1 antisense RNA 1	UMODL1-AS1	1,11
mediator complex subunit 21	MED21	1,11
splicing regulatory glutamine/lysine-rich protein 1	SREK1	1,11
POTE ankyrin domain family, member C	POTEC	1,11
RPTOR independent companion of MTOR, complex 2	RICTOR	1,11
opsin 5	OPN5	1,11
RAB6A, member RAS oncogene family	RAB6A	1,11
methyltransferase like 1	METTL1	1,11
cartilage oligomeric matrix protein	COMP	1,11
tumor necrosis factor receptor superfamily, member 11b	TNFRSF11B	1,11
JAZF zinc finger 1	JAZF1	1,11
ral guanine nucleotide dissociation stimulator-like 3	RGL3	1,11
Transcript Identified by AceView, Entrez Gene ID(s) 3551	IKBKB	1,11
uncharacterized LOC100129083; Transcript Identified by AceView, Entrez Gene ID(s) 100129083; novel transcript, antisense to SIGLEC10	LOC100129083; CTD-2616J11.2	1,11

marginal zone B and B1 cell-specific protein	MZB1	1,11
selectin L	SELL	1,11
SMG7 nonsense mediated mRNA decay factor	SMG7	1,11
TPTE and PTEN homologous inositol lipid phosphatase pseudogene; Transcript Identified by AceView, Entrez Gene ID(s) 1454; 400927	LOC400927; CSNK1E	1,11
actin gamma 1	ACTG1	1,11
adaptor-related protein complex 3, sigma 1 subunit	AP3S1	1,11
ubiquitin conjugating enzyme E2Q family member 2-like	UBE2Q2L	1,11
serum amyloid A-like 1	SAAL1	1,11
cysteine and histidine rich domain containing 1	CHORDC1	1,11
mitochondrial ribosomal protein L33	MRPL33	1,11
vasoactive intestinal peptide	VIP	1,11
interaction protein for cytohesin exchange factors 1	IPCEF1	1,11
transcription factor AP-2 delta (activating enhancer binding protein 2 delta)	TFAP2D	1,11
parathyroid hormone 2	PTH2	1,11
family with sequence similarity 25, member C	FAM25C	1,11
defensin, beta 124	DEFB124	1,11
SEC14-like lipid binding 4	SEC14L4	1,11
ADP-ribosyltransferase 5	ART5	1,11
peripherin	PRPH	1,11
Ras association and DIL domains	RADIL	1,11
ARP2 actin-related protein 2 homolog (yeast)	ACTR2	1,11
v-maf avian musculoaponeurotic fibrosarcoma oncogene homolog G	MAFG	1,11
retina and anterior neural fold homeobox 2	RAX2	1,11
gap junction protein beta 7	GJB7	1,11
Transcript Identified by AceView, Entrez Gene ID(s) 11031	RAB31	1,11
solute carrier organic anion transporter family, member 2A1	SLCO2A1	1,11
Aly/REF export factor	ALYREF	1,11
Rap guanine nucleotide exchange factor 2	RAPGEF2	1,11
olfactory receptor, family 10, subfamily A, member 2	OR10A2	1,11
coiled-coil domain containing 84	CCDC84	1,11
double C2-like domains, beta	DOC2B	1,11
adhesion G protein-coupled receptor F4	ADGRF4	1,11
zinc finger protein 7	ZNF7	1,11
solute carrier family 8 (sodium/calcium exchanger), member 1	SLC8A1	1,11
kalirin, RhoGEF kinase	KALRN	1,11
required for meiotic nuclear division 1 homolog	RMND1	1,11
fibroblast growth factor receptor-like 1	FGFRL1	1,11

latent transforming growth factor beta binding protein 3	LTBP3	1,11
adiponectin receptor 1	ADIPOR1	1,11
long intergenic non-protein coding RNA 1600	LINC01600	1,11
chromosome 4 open reading frame 46	C4orf46	1,11
late cornified envelope 4A	LCE4A	1,11
golgi-associated, gamma adaptin ear containing, ARF binding protein 3	GGA3	1,11
tankyrase 1 binding protein 1	TNKS1BP1	1,11
membrane-spanning 4-domains, subfamily A, member 13	MS4A13	1,11
dermatan sulfate epimerase-like	DSEL	1,11
SH3-domain binding protein 2	SH3BP2	1,11
chromosome 2 open reading frame 61	C2orf61	1,11
phosphorylase kinase, gamma 2 (testis)	PHKG2	1,11
collagen, type XI, alpha 1	COL11A1	1,11
membrane metallo-endopeptidase-like 1	MMEL1	1,11
olfactory receptor, family 5, subfamily B, member 21	OR5B21	1,11
inositol-trisphosphate 3-kinase B	ITPKB	1,11
RUSC1 antisense RNA 1	RUSC1-AS1	1,11
lymphocyte-specific protein 1; microRNA 7847	LSP1; MIR7847	1,11
WD repeat domain 75	WDR75	1,11
UDP-glucose 6-dehydrogenase	UGDH	1,11
flavin containing monooxygenase 2	FMO2	1,11
dopamine receptor D1	DRD1	1,11
gonadotropin releasing hormone receptor	GNRHR	1,11
sema domain, seven thrombospondin repeats (type 1 and type 1-like), transmembrane domain (TM) and short cytoplasmic domain, (semaphorin) 5B	SEMA5B	1,11
Usher syndrome 2A (autosomal recessive, mild)	USH2A	1,11
IKAROS family zinc finger 5	IKZF5	1,11
leucine rich repeat neuronal 3	LRRN3	1,11
N-acetylated alpha-linked acidic dipeptidase-like 2	NAALADL2	1,11
chromosome 5 open reading frame 51	C5orf51	1,11
transmembrane protein 130	TMEM130	1,11
microtubule-associated protein, RP/EB family, member 1	MAPRE1	1,11
phospholipase A2, group X	PLA2G10	1,11
neuroglobin	NGB	1,11
dynein, axonemal, light intermediate chain 1	DNALI1	1,11
chromosome 16 open reading frame 72-like	LOC389895	1,11
CNDP dipeptidase 2 (metallopeptidase M20 family)	CNDP2	1,11
Rho GTPase activating protein 6	ARHGAP6	1,11
Memczak2013 ANTISENSE, coding, INTERNAL, UTR3 best transcript NM_014310	RASD2	1,11

oral cancer overexpressed 1	ORAOV1	1,11
DEAD (Asp-Glu-Ala-Asp) box helicase 21	DDX21	1,11
olfactory receptor, family 4, subfamily D, member 10	OR4D10	1,11
oncomodulin	OCM	1,11
double homeobox 4 like 24; double homeobox 4 like 23	DUX4L24; DUX4L23	1,11
double homeobox 4 like 22; double homeobox 4 like 21	DUX4L22; DUX4L21	1,11
double homeobox 4 like 10; double homeobox 4 like 11	DUX4L10; DUX4L11	1,11
BCL6 corepressor	BCOR	1,11
dihydrouridine synthase 4-like	DUS4L	1,11
NADP-dependent oxidoreductase domain containing 1	NOXRED1	1,11
ribosomal protein S6 kinase, 90kDa, polypeptide 1	RPS6KA1	1,11
ubiquitin specific peptidase 32	USP32	1,11
prickle homolog 1	PRICKLE1	1,11
	AC208162.1	1,11
phosphodiesterase 10A	PDE10A	1,11
prostaglandin E receptor 4 (subtype EP4)	PTGER4	1,11
interleukin 36, gamma	IL36G	1,11
AT-hook transcription factor	AKNA	1,11
PYD (pyrin domain) containing 1	PYDC1	1,11
collagen, type I, alpha 2	COL1A2	1,11
fibrillarin	FBL	1,11
Transcript Identified by AceView, Entrez Gene ID(s) 84620	ST6GAL2	1,11
fasciculation and elongation protein zeta 1	FEZ1	1,11
transmembrane BAX inhibitor motif containing 1; microRNA 6513	TMBIM1; MIR6513	1,11
interleukin 22 receptor, alpha 2	IL22RA2	1,11
zinc finger protein 155	ZNF155	1,11
aarF domain containing kinase 4	ADCK4	1,11
Bardet-Biedl syndrome 4	BBS4	1,11
Memczak2013 ALT_ACCEPTOR, ALT_DONOR, coding, INTERNAL, intronic best transcript NM_002806	PSMC6	1,11
peptidyl-tRNA hydrolase 2	PTRH2	1,11
molybdenum cofactor synthesis 1	MOCS1	1,11
receptor accessory protein 3	REEP3	1,11
synaptotagmin IV	SYT4	1,11
Transcript Identified by AceView, Entrez Gene ID(s) 196527	ANO6	1,11
RAD52 homolog, DNA repair protein	RAD52	1,11
leucine rich repeat containing 8 family, member E	LRRC8E	1,11
RUN and FYVE domain containing 1	RUFY1	1,11
MT-RNR2-like 9	MTRNR2L9	1,11

parvalbumin	PVALB	1,11
5-hydroxytryptamine (serotonin) receptor 3A, ionotropic	HTR3A	1,11
ankyrin repeat domain 31	ANKRD31	1,11
dorsal root ganglia homeobox	DRGX	1,11
defensin, beta 132	DEFB132	1,11
Memczak2013 ANTISENSE, coding, INTERNAL, intronic best transcript NM_003335	UBA7	1,11
centriolar coiled coil protein 110kDa	CCP110	1,11
KIAA0907; small Cajal body-specific RNA 4	KIAA0907; SCARNA4	1,11
synaptophysin	SYP	1,11
adhesion G protein-coupled receptor G7	ADGRG7	1,11
potassium channel, two pore domain subfamily K, member 12	KCNK12	1,11
Rho-related BTB domain containing 3	RHOBTB3	1,11
histone deacetylase 1	HDAC1	1,11
frizzled class receptor 7	FZD7	1,11
basonuclin 1	BNC1	1,11
chromosome 11 open reading frame 73	C11orf73	1,11
lectin, galactoside-binding, soluble, 12	LGALS12	1,11
Rieske (Fe-S) domain containing	RFESD	1,11
MAGE family member B6	MAGEB6	1,11
neuroblastoma breakpoint family, member 3	NBPF3	1,11
uncharacterized LOC100287728; Transcript Identified by AceView; novel transcript	LOC100287728; RP11-85L21.4; jerva	1,11
lysozyme-like 4	LYZL4	1,11
surfactant protein B	SFTPB	1,11
chromosome 8 open reading frame 89	C8orf89	1,11
dynein, axonemal, heavy chain 12	DNAH12	1,11
Rho GDP dissociation inhibitor (GDI) gamma	ARHGDIG	1,11
SR-related CTD-associated factor 4	SCAF4	1,11
olfactory receptor, family 1, subfamily K, member 1	OR1K1	1,11
ficolin (collagen/fibrinogen domain containing) 3	FCN3	1,11
hydroxysteroid (17-beta) dehydrogenase 2	HSD17B2	1,11
zinc finger protein 474	ZNF474	1,11
KIAA2012	KIAA2012	1,11
RNA binding motif protein 28	RBM28	1,11
kringle containing transmembrane protein 2	KREMEN2	1,11
COMM domain containing 8	COMMD8	1,11
serpin peptidase inhibitor, clade B (ovalbumin), member 12	SERPINB12	1,11
neuro-oncological ventral antigen 2	NOVA2	1,11
PAP associated domain containing 4	PAPD4	1,11
GPR158 antisense RNA 1	GPR158-AS1	1,11

polypeptide N-acetylgalactosaminyltransferase 5	GALNT5	1,11
Sad1 and UNC84 domain containing 1	SUN1	1,11
olfactory receptor, family 6, subfamily C, member 1	OR6C1	1,11
SRY box 7	SOX7	1,11
interleukin 34	IL34	1,11
ALX homeobox 1	ALX1	1,11
olfactory receptor, family 56, subfamily A, member 1	OR56A1	1,11
Memczak2013 ANTISENSE, coding, INTERNAL, intronic best transcript NM_006947	SRP72	1,11
G protein-coupled receptor 45	GPR45	1,11
transmembrane protein 178B	TMEM178B	1,11
transcription factor CP2-like 1	TFCP2L1	1,11
unc-5 netrin receptor C	UNC5C	1,11
ADP-ribosylation factor like GTPase 11	ARL11	1,11
Zhang2013 ALT_ACCEPTOR, ALT_DONOR, coding, INTERNAL, intronic best transcript NM_001033549	BABAM1	1,11
Jeck2013 ALT_ACCEPTOR, ALT_DONOR, coding, INTERNAL, intronic best transcript NM_014629	ARHGEF10	1,11
lipin 3	LPIN3	1,11
zinc finger CCCH-type containing 6	ZC3H6	1,11
actin filament associated protein 1-like 1	AFAP1L1	1,11
olfactory receptor, family 51, subfamily E, member 2	OR51E2	1,11
basic charge, Y-linked, 2B	BPY2B	1,11
olfactory receptor, family 1, subfamily J, member 2	OR1J2	1,11
zinc finger protein 549	ZNF549	1,11
olfactory receptor, family 10, subfamily A, member 5	OR10A5	1,11
Transcript Identified by AceView, Entrez Gene ID(s) 10587	TXNRD2	1,11
ribonuclease, RNase A family, 2 (liver, eosinophil-derived neurotoxin)	RNASE2	1,11
transcription factor 15 (basic helix-loop-helix)	TCF15	1,11
F-box and leucine-rich repeat protein 16	FBXL16	1,11
transmembrane protein 196	TMEM196	1,11
cyclin G2	CCNG2	1,11
neuronal PAS domain protein 1	NPAS1	1,11
LSM4 homolog, U6 small nuclear RNA and mRNA degradation associated	LSM4	1,11
thioredoxin reductase 3 neighbor; thioredoxin reductase 3	TXNRD3NB; TXNRD3	1,11
HCLS1 binding protein 3	HS1BP3	1,11
TOX high mobility group box family member 2	TOX2	1,11
family with sequence similarity 175, member A	FAM175A	1,11
membrane-spanning 4-domains, subfamily A, member 3 (hematopoietic cell-specific)	MS4A3	1,11
kallikrein related peptidase 8	KLK8	1,11

Bardet-Biedl syndrome 7	BBS7	1,11
solute carrier family 25 (S-adenosylmethionine carrier), member 26	SLC25A26	1,11
transmembrane protein 151A	TMEM151A	1,11
ELOVL fatty acid elongase 5	ELOVL5	1,11
zinc finger protein 568	ZNF568	1,11
spinster homolog 2 (Drosophila)	SPNS2	1,11
ubiquitin protein ligase E3B	UBE3B	1,11
spindle apparatus coiled-coil protein 1	SPDL1	1,11
glutamate receptor, metabotropic 4	GRM4	1,11
hes-related family bHLH transcription factor with YRPW motif-like	HEYL	1,11
junctional adhesion molecule 2	JAM2	1,11
F-box protein 40	FBXO40	1,11
transmembrane protein 35	TMEM35	1,11
SCAN domain containing 2 pseudogene	SCAND2P	1,11
interleukin 13	IL13	1,11
EF-hand calcium binding domain 10	EFCAB10	1,11
cancer/testis antigen 62	CT62	1,11
receptor accessory protein 5	REEP5	1,10
5-hydroxytryptamine (serotonin) receptor 6, G protein-coupled	HTR6	1,10
polypeptide N-acetylgalactosaminyltransferase-like 6	GALNTL6	1,10
hepatitis A virus cellular receptor 2	HAVCR2	1,10
ligand dependent nuclear receptor interacting factor 1	LRIF1	1,10
solute carrier family 17 (acidic sugar transporter), member 5	SLC17A5	1,10
pannexin 2	PANX2	1,10
olfactory receptor, family 9, subfamily A, member 4	OR9A4	1,10
cytohesin 4	CYTH4	1,10
Transcript Identified by AceView, Entrez Gene ID(s) 9107	MTMR6	1,10
YKT6 v-SNARE homolog (S. cerevisiae)	YKT6	1,10
family with sequence similarity 47, member B	FAM47B	1,10
multimerin 2	MMRN2	1,10
potassium channel, inwardly rectifying subfamily J, member 14	KCNJ14	1,10
zinc finger protein 571	ZNF571	1,10
ribosomal RNA processing 1	RRP1	1,10
dual oxidase maturation factor 1	DUOXA1	1,10
chibby homolog 3 (Drosophila)	CBY3	1,10
ankyrin repeat domain 19, pseudogene	ANKRD19P	1,10
acetoacetyl-CoA synthetase pseudogene 1	AACSP1	1,10
chymotrypsin-like elastase family, member 2B	CELA2B	1,10
chromosome 5 open reading frame 28	C5orf28	1,10

aquaporin 5	AQP5	1,10
spermatogenesis associated 22	SPATA22	1,10
Rho GTPase activating protein 25	ARHGAP25	1,10
vimentin	VIM	1,10
Memczak2013 ALT_ACCEPTOR, ALT_DONOR, coding, INTERNAL, intronic best transcript NM_138799	MBOAT2	1,10
protein phosphatase 2, catalytic subunit, alpha isozyme	PPP2CA	1,10
interleukin 6	IL6	1,10
kelch domain containing 8A	KLHDC8A	1,10
tRNA-yW synthesizing protein 5	TYW5	1,10
olfactory receptor, family 2, subfamily B, member 2	OR2B2	1,10
glycoprotein integral membrane 1	GINM1	1,10
HGF activator	HGFAC	1,10
hematopoietic prostaglandin D synthase	HPGDS	1,10
BTB (POZ) domain containing 19	BTBD19	1,10
long intergenic non-protein coding RNA 1270	LINC01270	1,10
unc-45 myosin chaperone B	UNC45B	1,10
kelch-like family member 10	KLHL10	1,10
megalencephalic leukoencephalopathy with subcortical cysts 1	MLC1	1,10
acid sensing ion channel family member 5	ASIC5	1,10
adhesion G protein-coupled receptor A2	ADGRA2	1,10
potassium channel, two pore domain subfamily K, member 1	KCNK1	1,10
alcohol dehydrogenase 1C (class I), gamma polypeptide	ADH1C	1,10
zinc finger protein 197	ZNF197	1,10
sushi, von Willebrand factor type A, EGF and pentraxin domain containing 1	SVEP1	1,10
solute carrier family 45, member 2	SLC45A2	1,10
glycine-N-acyltransferase-like 3	GLYATL3	1,10
RAB44, member RAS oncogene family	RAB44	1,10
insulin-like growth factor 1 (somatomedin C)	IGF1	1,10
S-antigen; retina and pineal gland (arrestin)	SAG	1,10
chromosome 6 open reading frame 229	C6orf229	1,10
family with sequence similarity 124 member B	FAM124B	1,10
transmembrane protein 220	TMEM220	1,10
organic solute carrier partner 1	OSCP1	1,10
coiled-coil domain containing 187	CCDC187	1,10
TBC1 domain family, member 10C	TBC1D10C	1,10
GATA binding protein 1 (globin transcription factor 1)	GATA1	1,10
uridine-cytidine kinase 2; microRNA 3658	UCK2; MIR3658	1,10
WNT1 inducible signaling pathway protein 1	WISP1	1,10
mucosal vascular addressin cell adhesion molecule 1	MADCAM1	1,10
ATPase, Ca++ transporting, plasma membrane 3	ATP2B3	1,10
SLX4 structure-specific endonuclease subunit	SLX4	1,10

adaptor-related protein complex 4, sigma 1 subunit	AP4S1	1,10
dpy-19-like 2 (C. elegans)	DPY19L2	1,10
ubiquitin conjugating enzyme E2Q family-like 1	UBE2QL1	1,10
UBA-like domain containing 1	UBALD1	1,10
UTP18 small subunit (SSU) processome component	UTP18	1,10
Zhang2013 ALT_ACCEPTOR, ALT_DONOR, coding, INTERNAL, intronic best transcript NM_001164446	C6orf132	1,10
WD repeat and FYVE domain containing 3	WDFY3	1,10
BARX homeobox 2	BARX2	1,10
fission, mitochondrial 1	FIS1	1,10
ZFP42 zinc finger protein	ZFP42	1,10
dishevelled associated activator of morphogenesis 2	DAAM2	1,10
breast carcinoma amplified sequence 4	BCAS4	1,10
general transcription factor IIH subunit 5	GTF2H5	1,10
hydroxysteroid (17-beta) dehydrogenase 10	HSD17B10	1,10
zinc finger and SCAN domain containing 1	ZSCAN1	1,10
lysine (K)-specific methyltransferase 2B	KMT2B	1,10
prolyl 4-hydroxylase, transmembrane (endoplasmic reticulum)	P4HTM	1,10
axonemal dynein light chain domain containing 1	AXDND1	1,10
SEH1-like nucleoporin	SEH1L	1,10
interactor of little elongation complex ELL subunit 2	ICE2	1,10
dual specificity phosphatase 27 (putative)	DUSP27	1,10
peroxisomal biogenesis factor 26	PEX26	1,10
G protein-coupled receptor 1	GPR1	1,10
solute carrier organic anion transporter family, member 3A1	SLCO3A1	1,10
N-deacetylase/N-sulfotransferase (heparan glucosaminyl) 3	NDST3	1,10
ZFP82 zinc finger protein	ZFP82	1,10
olfactory receptor, family 4, subfamily K, member 17	OR4K17	1,10
WD repeat domain 43; small nucleolar RNA, C/D box 53	WDR43; SNORD53	1,10
ATPase, class II, type 9A	ATP9A	1,10
chromosome 9 open reading frame 24	C9orf24	1,10
RAB GTPase activating protein 1-like	RABGAP1L	1,10
zinc finger protein 679	ZNF679	1,10
cyclin L1	CCNL1	1,10
mitochondrial ribosomal protein L37	MRPL37	1,10
family with sequence similarity 120A	FAM120A	1,10
NOP16 nucleolar protein	NOP16	1,10
RIC3 acetylcholine receptor chaperone	RIC3	1,10
CKLF-like MARVEL transmembrane domain containing 7	CMTM7	1,10
thymidine kinase 2, mitochondrial	TK2	1,10
peptidase inhibitor 3, skin-derived	PI3	1,10

transmembrane protein 8C	TMEM8C	1,10
1-aminocyclopropane-1-carboxylate synthase (inactive)-like	ACCSL	1,10
major histocompatibility complex, class II, DR beta 5	HLA-DRB5	1,10
tubulin, alpha 8	TUBA8	1,10
Rho family GTPase 2	RND2	1,10
glial fibrillary acidic protein	GFAP	1,10
chromosome 11 open reading frame 95	C11orf95	1,10
chromosome 17 open reading frame 107	C17orf107	1,10
NADH dehydrogenase (ubiquinone) 1, subcomplex unknown, 1, 6kDa	NDUFC1	1,10
nucleus accumbens associated 1, BEN and BTB (POZ) domain containing	NACC1	1,10
transcobalamin I (vitamin B12 binding protein, R binder family)	TCN1	1,10
patched domain containing 1	PTCHD1	1,10
olfactory receptor, family 4, subfamily M, member 2	OR4M2	1,10
tumor necrosis factor (ligand) superfamily, member 18	TNFSF18	1,10
olfactory receptor, family 10, subfamily AG, member 1	OR10AG1	1,10
defensin, beta 4B	DEFB4B	1,10
SATB homeobox 1	SATB1	1,10
N-acetyltransferase domain containing 1	NATD1	1,10
butyrophilin-like 8	BTNL8	1,10
dipeptidyl-peptidase 9	DPP9	1,10
BRICK1, SCAR/WAVE actin-nucleating complex subunit	BRK1	1,10
dynein regulatory complex subunit 1	DRC1	1,10
nuclear RNA export factor 2B; nuclear RNA export factor 2	NXF2B; NXF2	1,10
vesicle amine transport 1-like	VAT1L	1,10
BEN domain containing 6	BEND6	1,10
cytochrome P450, family 27, subfamily C, polypeptide 1	CYP27C1	1,10
nudE neurodevelopment protein 1-like 1	NDEL1	1,10
EPH receptor A6	EPHA6	1,10
neuroblastoma breakpoint family, member 8	NBPF8	1,10
G protein-coupled bile acid receptor 1	GPBAR1	1,10
glutamate receptor, ionotropic, AMPA 1	GRIA1	1,10
ER membrane protein complex subunit 7	EMC7	1,10
olfactory receptor, family 7, subfamily D, member 2	OR7D2	1,10
keratin associated protein 9-2	KRTAP9-2	1,10
phosphodiesterase 5A, cGMP-specific	PDE5A	1,10
mediator of cell motility 1	MEMO1	1,10
lysozyme-like 1	LYZL1	1,10
NK3 homeobox 2	NKX3-2	1,10

Jeck2013 ALT_ACCEPTOR, ALT_DONOR, coding, INTERNAL, intronic best transcript NM_001014797; Transcript Identified by AceView	KCNMA1; swamu	1,10
ret proto-oncogene	RET	1,10
matrix extracellular phosphoglycoprotein	MEPE	1,10
cullin 2	CUL2	1,10
serine carboxypeptidase 1	SCPEP1	1,10
trafficking protein particle complex 4	TRAPPC4	1,10
methyltransferase like 9	METTL9	1,10
ankyrin repeat domain 30B	ANKRD30B	1,10
GIPC PDZ domain containing family, member 3	GIPC3	1,10
osteocrin	OSTN	1,10
N-acylsphingosine amidohydrolase (acid ceramidase) 1	ASAH1	1,10
complement component 3a receptor 1	C3AR1	1,10
UDP glycosyltransferase 3 family, polypeptide A1	UGT3A1	1,10
G-rich RNA sequence binding factor 1	GRSF1	1,10
shadow of prion protein homolog (zebrafish)	SPRN	1,10
tumor protein p63 regulated 1	TPRG1	1,10
ADAM metallopeptidase with thrombospondin type 1 motif 13	ADAMTS13	1,10
chaperonin containing TCP1, subunit 7 (eta)	CCT7	1,10
ghrelin/obestatin prepropeptide	GHRL	1,10
Memczak2013 ANTISENSE, coding, INTERNAL, intronic best transcript NM_021931	DHX35	1,10
F-box protein 48	FBXO48	1,10
PR domain containing 13	PRDM13	1,10
transglutaminase 4	TGM4	1,10
cornifelin	CNFN	1,10
zinc finger protein 211	ZNF211	1,10
uncharacterized LOC285150; uncharacterized LOC285150 [Source:EntrezGene;Acc:285150]; novel transcript	FLJ33534; AC062028.1	1,10
lipoxygenase homology domains 1	LOXHD1	1,10
sorting nexin family member 27	SNX27	1,10
tripartite motif containing 64C	TRIM64C	1,10
taste receptor, type 1, member 2	TAS1R2	1,10
family with sequence similarity 205, member A	FAM205A	1,10
HLA complex group 22	HCG22	1,10
MORN repeat containing 1	MORN1	1,10
LIM domain and actin binding 1	LIMA1	1,10
tyrosine 3-monooxygenase/tryptophan 5-monooxygenase activation protein, epsilon pseudogene 7	YWHAEP7	1,10
EPB41L4A antisense RNA 2 (head to head)	EPB41L4A-AS2	1,10
basonuclin 2	BNC2	1,10
cytotoxic and regulatory T-cell molecule	CRTAM	1,10

ATP synthase mitochondrial F1 complex assembly factor 2	ATPAF2	1,10
target of EGR1, member 1 (nuclear)	TOE1	1,10
aquaporin 1 (Colton blood group)	AQP1	1,10
polymerase (DNA directed), gamma 2, accessory subunit	POLG2	1,10
carbonyl reductase 3	CBR3	1,10
out at first homolog	OAF	1,10
tropomodulin 4 (muscle)	TMOD4	1,10
zinc finger and BTB domain containing 8B	ZBTB8B	1,10
testis expressed 13B	TEX13B	1,10
Transcript Identified by AceView, Entrez Gene ID(s) 9246	UBE2L6	1,10
tripartite motif containing 39	TRIM39	1,10
olfactory receptor, family 13, subfamily C, member 8	OR13C8	1,10
paraoxonase 3	PON3	1,10
immunoglobulin superfamily, DCC subclass, member 4	IGDCC4	1,10
Memczak2013 ALT_ACCEPTOR, ALT_DONOR, coding, INTERNAL, intronic best transcript NM_014502	PRPF19	1,10
Memczak2013 ALT_ACCEPTOR, ALT_DONOR, coding, INTERNAL, intronic best transcript NM_001170794	BACH2	1,10
ubiquilin 3	UBQLN3	1,10
guanylate cyclase activator 1C	GUCA1C	1,10
retinoic acid induced 2	RAI2	1,10
SHANK-associated RH domain interactor	SHARPIN	1,10
telomeric repeat binding factor 2, interacting protein	TERF2IP	1,10
odorant binding protein 2B	OBP2B	1,10
kinesin family member 20B	KIF20B	1,10
Transcript Identified by AceView, Entrez Gene ID(s) 8850	KAT2B	1,10
Rab interacting lysosomal protein	RILP	1,10
Transcript Identified by AceView, Entrez Gene ID(s) 5885	RAD21	1,10
serpin peptidase inhibitor, clade B (ovalbumin), member 7	SERPINB7	1,10
interleukin 2	IL2	1,10
slingshot protein phosphatase 1	SSH1	1,10
inverted formin, FH2 and WH2 domain containing	INF2	1,10
dihydrodiol dehydrogenase (dimeric)	DHDH	1,10
BLOC1S1-RDH5 readthrough; Uncharacterized protein [Source:UniProtKB/TrEMBL;Acc:F8VNQ1]	BLOC1S1-RDH5; RP11-644F5.10	1,10
zinc finger protein 592	ZNF592	1,10
PRAME family member 27	PRAMEF27	1,10
zinc finger protein 414	ZNF414	1,10
golgin A6 family-like 2	GOLGA6L2	1,10

zinc finger, C3HC-type containing 1	ZC3HC1	1,10
ZFP2 zinc finger protein	ZFP2	1,10
protease, serine, 54	PRSS54	1,10
centrosomal protein 104kDa	CEP104	1,10
regulating synaptic membrane exocytosis 2	RIMS2	1,10
T-cell leukemia homeobox 2	TLX2	1,10
zinc finger protein 501	ZNF501	1,10
CTD nuclear envelope phosphatase 1 regulatory subunit 1	CNEP1R1	1,10
potassium channel, inwardly rectifying subfamily J, member 11	KCNJ11	1,10
metastasis suppressor 1	MTSS1	1,10
tousled-like kinase 1	TLK1	1,10
Kruppel-like factor 13	KLF13	1,10
heat shock transcription factor 4; F-box and leucine-rich repeat protein 8	HSF4; FBXL8	1,10
solute carrier family 7, member 14	SLC7A14	1,10
NUT family member 2G	NUTM2G	1,10
stimulated by retinoic acid 13	STRA13	1,10
tektin 1	TEKT1	1,10
synaptotagmin V	SYT5	1,10
long intergenic non-protein coding RNA 1276	LINC01276	1,10
somatostatin receptor 2	SSTR2	1,10
Transcript Identified by AceView, Entrez Gene ID(s) 669	BPGM	1,10
sclerostin domain containing 1	SOSTDC1	1,10
checkpoint with forkhead and ring finger domains, E3 ubiquitin protein ligase	CHFR	1,10
zona pellucida-like domain containing 1	ZPLD1	1,10
RNA binding motif protein, Y-linked, family 1, member D; RNA binding motif protein, Y-linked, family 1, member B; RNA binding motif protein, Y-linked, family 1, member A1	RBMY1D; RBMY1B; RBMY1A1	1,10
zinc finger protein 518A	ZNF518A	1,10
calcineurin-like phosphoesterase domain containing 1	CPPED1	1,09
RAS protein activator like 3	RASAL3	1,09
taste receptor, type 2, member 8	TAS2R8	1,09
C-type lectin domain family 4, member F	CLEC4F	1,09
small integral membrane protein 10	SMIM10	1,09
par-3 family cell polarity regulator	PARD3	1,09
lysine (K)-specific demethylase 3A	KDM3A	1,09
nephrosis 1, congenital, Finnish type (nephrin)	NPHS1	1,09
olfactory receptor, family 5, subfamily AU, member 1	OR5AU1	1,09
proprotein convertase subtilisin/kexin type 7	PCSK7	1,09
eyes shut homolog (Drosophila)	EYS	1,09
ferritin, heavy polypeptide-like 17	FTHL17	1,09

ADAM-like, decysin 1	ADAMDEC1	1,09
stathmin-like 4	STMN4	1,09
chromosome 2 open reading frame 16	C2orf16	1,09
cactin, spliceosome C complex subunit	CACTIN	1,09
orthopedia homeobox	OTP	1,09
chromosome 20 open reading frame 195	C20orf195	1,09
exostosin-like glycosyltransferase 1	EXTL1	1,09
zinc finger CCCH-type containing 14	ZC3H14	1,09
ankyrin repeat, family A (RFXANK-like), 2	ANKRA2	1,09
aldo-keto reductase family 7-like (gene/pseudogene)	AKR7L	1,09
centrosomal protein 44kDa	CEP44	1,09
serine/threonine kinase 36	STK36	1,09
PIF1 5-to-3 DNA helicase	PIF1	1,09
WW domain containing E3 ubiquitin protein ligase 2	WWP2	1,09
G protein-coupled receptor 148	GPR148	1,09
ubiquitin protein ligase E3 component n-recognin 7 (putative)	UBR7	1,09
apoptosis enhancing nuclease	AEN	1,09
PH domain and leucine rich repeat protein phosphatase 2	PHLPP2	1,09
aquaporin 2 (collecting duct)	AQP2	1,09
5-hydroxytryptamine (serotonin) receptor 2A, G protein-coupled	HTR2A	1,09
BCL2-like 14 (apoptosis facilitator)	BCL2L14	1,09
Memczak2013 ALT_DONOR, coding, INTERNAL, intronic best transcript NM_198268	HIPK1	1,09
SUV3-like helicase	SUPV3L1	1,09
G protein-coupled receptor 108; microRNA 6791	GPR108; MIR6791	1,09
ankyrin repeat domain 55	ANKRD55	1,09
lectin, galactoside-binding, soluble, 4	LGALS4	1,09
BR serine/threonine kinase 1	BRSK1	1,09
Wilms tumor 1	WT1	1,09
BAALC antisense RNA 2	BAALC-AS2	1,09
protein phosphatase, Mg2+/Mn2+ dependent, 1G	PPM1G	1,09
Transcript Identified by AceView, Entrez Gene ID(s) 2646	GCKR	1,09
retinal degeneration 3-like	RD3L	1,09
RAS p21 protein activator 3	RASA3	1,09
aryl hydrocarbon receptor nuclear translocator	ARNT	1,09
Transcript Identified by AceView, Entrez Gene ID(s) 57181	SLC39A10	1,09
chemokine (C-C motif) ligand 18	CCL18	1,09
ligand of numb-protein X 1, E3 ubiquitin protein ligase	LNX1	1,09
spermatogenesis associated 8	SPATA8	1,09

Transcript Identified by AceView, Entrez Gene ID(s) 26508	HEYL	1,09
crystallin beta A1	CRYBA1	1,09
zinc finger protein 579	ZNF579	1,09
NADH dehydrogenase (ubiquinone) 1 alpha subcomplex, 10, 42kDa	NDUFA10	1,09
sulfotransferase family 1C member 3	SULT1C3	1,09
hydroxyacyl-CoA dehydrogenase/3-ketoacyl-CoA thiolase/enoyl-CoA hydratase (trifunctional protein), alpha subunit	HADHA	1,09
fibroblast activation protein alpha	FAP	1,09
Homo sapiens neuropeptide Y receptor Y4 (NPY4R), transcript variant 2, mRNA.; neuropeptide Y receptor type 4-like; Homo sapiens pancreatic polypeptide receptor 1, mRNA (cDNA clone MGC:116895 IMAGE:40005502), complete cds.; Homo sapiens pancreatic polypeptide receptor 1, mRNA (cDNA clone MGC:116897 IMAGE:40005506), complete cds.; novel transcript	NPY4R; CH17-360D5.1; PPYR1	1,09
anterior gradient 3, protein disulphide isomerase family member	AGR3	1,09
pre-mRNA processing factor 6	PRPF6	1,09
torsin family 1, member B (torsin B)	TOR1B	1,09
mitochondrial genome maintenance exonuclease 1	MGME1	1,09
olfactory receptor, family 2, subfamily AT, member 4	OR2AT4	1,09
syncollin	SYCN	1,09
nucleoporin 37kDa	NUP37	1,09
interleukin 1 receptor associated kinase 2	IRAK2	1,09
nebulette	NEBL	1,09
complement component 1, q subcomponent-like 4	C1QL4	1,09
solute carrier family 34 (type II sodium/phosphate cotransporter), member 3	SLC34A3	1,09
ubiquitin specific peptidase 3	USP3	1,09
heat shock transcription factor family member 5	HSF5	1,09
ral guanine nucleotide dissociation stimulator-like 4	RGL4	1,09
phosphatidylinositol glycan anchor biosynthesis class G	PIGG	1,09
RNA binding motif protein 46	RBM46	1,09
solute carrier family 45, member 1	SLC45A1	1,09
dual specificity phosphatase and pro isomerase domain containing 1	DUPD1	1,09
ATP6V1G2-DDX39B readthrough (NMD candidate)	ATP6V1G2-DDX39B	1,09
TP53 target 3C	TP53TG3C	1,09
small integral membrane protein 18	SMIM18	1,09

calcium channel, voltage-dependent, L type, alpha 1C subunit	CACNA1C	1,09
PX domain containing 1	PXDC1	1,09
zinc finger, CCHC domain containing 12	ZCCHC12	1,09
2-deoxynucleoside 5-phosphate N-hydrolase 1	DNPH1	1,09
solute carrier family 25 (S-adenosylmethionine carrier), member 26	SLC25A26	1,09
Memczak2013 ALT_ACCEPTOR, ALT_DONOR, coding, INTERNAL, intronic best transcript NM_001170794	BACH2	1,09
solute carrier family 8 (sodium/calcium exchanger), member 3	SLC8A3	1,09
elastin	ELN	1,09
G antigen 2B; G antigen 2A; G antigen 2C	GAGE2B; GAGE2A; GAGE2C	1,09
peptidyl arginine deiminase, type III	PADI3	1,09
ATP binding cassette subfamily A member 6	ABCA6	1,09
netrin G2	NTNG2	1,09
POTE ankyrin domain family, member D	POTED	1,09
chromosome 3 open reading frame 84	C3orf84	1,09
aldo-keto reductase family 7, member A2	AKR7A2	1,09
RAB, member of RAS oncogene family-like 2A	RABL2A	1,09
intercellular adhesion molecule 5	ICAM5	1,09
toll-like receptor 9	TLR9	1,09
zinc finger and SCAN domain containing 20	ZSCAN20	1,09
complement factor H-related 3	CFHR3	1,09
oxidized low density lipoprotein (lectin-like) receptor 1	OLR1	1,09
transmembrane protease, serine 9	TMPRSS9	1,09
free fatty acid receptor 4	FFAR4	1,09
signal peptide peptidase like 3	SPPL3	1,09
eukaryotic translation initiation factor 2 alpha kinase 4	EIF2AK4	1,09
ATP binding cassette subfamily A member 9	ABCA9	1,09
regulator of microtubule dynamics 3	RMDN3	1,09
polyhomeotic homolog 1 (Drosophila)	PHC1	1,09
olfactory receptor, family 5, subfamily J, member 2	OR5J2	1,09
netrin 4	NTN4	1,09
family with sequence similarity 26, member D	FAM26D	1,09
ets variant 7	ETV7	1,09
homeodomain interacting protein kinase 4	HIPK4	1,09
ESX homeobox 1	ESX1	1,09
HCK proto-oncogene, Src family tyrosine kinase	HCK	1,09
CD300 molecule-like family member f	CD300LF	1,09
small integral membrane protein 15	SMIM15	1,09
TraB domain containing 2A	TRABD2A	1,09
glucokinase (hexokinase 4)	GCK	1,09

Jeck2013 ALT_ACCEPTOR, ALT_DONOR, coding, INTERNAL, intronic best transcript NM_017660	GATAD2A	1,09
caveolin 3	CAV3	1,09
microfibrillar associated protein 3 like	MFAP3L	1,09
olfactory receptor, family 52, subfamily M, member 1	OR52M1	1,09
protein phosphatase, Mg2+/Mn2+ dependent, 1A	PPM1A	1,09
family with sequence similarity 170, member A	FAM170A	1,09
motilin	MLN	1,09
keratinocyte associated protein 3	KRTCAP3	1,09
family with sequence similarity 71, member C	FAM71C	1,09
Transcript Identified by AceView, Entrez Gene ID(s) 79658	ARHGAP10	1,09
adaptor-related protein complex 4, beta 1 subunit	AP4B1	1,09
leucine rich repeat containing 41	LRRC41	1,09
kinesin family member 19	KIF19	1,09
origin recognition complex subunit 4	ORC4	1,09
dihydropyrimidinase-like 4	DPYSL4	1,09
H2B histone family, member W, testis-specific	H2BFWT	1,09
extended synaptotagmin-like protein 2	ESYT2	1,09
S100 calcium binding protein A8	S100A8	1,09
membrane-spanning 4-domains, subfamily A, member 1	MS4A1	1,09
testis-specific serine kinase 2	TSSK2	1,09
tRNA methyltransferase 10A	TRMT10A	1,09
cytochrome P450, family 2, subfamily F, polypeptide 1	CYP2F1	1,09
nephronophthisis 1 (juvenile)	NPHP1	1,09
NADH dehydrogenase, subunit 1 (complex I)	ND1	1,09
interleukin 5	IL5	1,09
atonal bHLH transcription factor 8	ATOH8	1,09
chromodomain helicase DNA binding protein 8	CHD8	1,09
beta-1,3-N-acetylgalactosaminyltransferase 1 (globoside blood group)	B3GALNT1	1,09
carboxypeptidase, vitellogenic-like	CPVL	1,09
keratin 16, type I	KRT16	1,09
gamma-aminobutyric acid (GABA) B receptor, 2	GABBR2	1,09
uncharacterized LOC149373; Transcript Identified by AceView; novel transcript	LOC149373; RP5-1097F14.3; kosimo	1,09
family with sequence similarity 160, member B1	FAM160B1	1,09
leucine rich repeat transmembrane neuronal 1	LRRTM1	1,09
chromosome 19 open reading frame 47	C19orf47	1,09
signal-regulatory protein beta 2	SIRPB2	1,09
PQ loop repeat containing 3	PQLC3	1,09
ubiquitously expressed prefoldin like chaperone	UXT	1,09
calcium channel flower domain containing 1	CACFD1	1,09

olfactory receptor, family 10, subfamily S, member 1	OR10S1	1,09
transcription elongation factor A (SII)-like 5	TCEAL5	1,09
THUMP domain containing 3	THUMPD3	1,09
thiamin pyrophosphokinase 1	TPK1	1,09
amylase, alpha 1A (salivary); amylase, alpha 1C (salivary)	AMY1A; AMY1C	1,09
chromosome 2 open reading frame 81	C2orf81	1,09
protein tyrosine phosphatase type IVA, member 2	PTP4A2	1,09
DDB1 and CUL4 associated factor 8-like 2	DCAF8L2	1,09
Memczak2013 ANTISENSE, coding, INTERNAL, UTR3 best transcript NM_130847	AMOTL1	1,09
leptin receptor overlapping transcript-like 1	LEPROTL1	1,09
transcription factor 7-like 2 (T-cell specific, HMG-box)	TCF7L2	1,09
ADNP homeobox 2	ADNP2	1,09
protein tyrosine phosphatase, receptor type, T	PTPRT	1,09
RAD52 motif containing 1	RDM1	1,09
mediator complex subunit 1	MED1	1,09
Memczak2013 ANTISENSE, coding, INTERNAL, UTR3 best transcript NM_015354	NUP188	1,09
UDP-GlcNAc:betaGal beta-1,3-N-acetylglucosaminyltransferase 9	B3GNT9	1,09
WNT inhibitory factor 1	WIF1	1,09
coiled-coil domain containing 169	CCDC169	1,09
Salzman2013 ANTISENSE, coding, INTERNAL, intronic best transcript NM_032968	PCDH11X	1,09
mitochondrial rRNA methyltransferase 1	MRM1	1,09
bestrophin 3	BEST3	1,09
coilin	COIL	1,09
zinc finger protein 727	ZNF727	1,09
cerebral dopamine neurotrophic factor	CDNF	1,09
cystatin 9 (testatin)	CST9	1,09
interferon, alpha 21	IFNA21	1,09
Transcript Identified by AceView, Entrez Gene ID(s) 94031	HTRA3	1,09
regulation of nuclear pre-mRNA domain containing 1B	RPRD1B	1,09
membrane associated ring finger 8	MARCH8	1,09
sarcoglycan gamma	SGCG	1,09
EYA transcriptional coactivator and phosphatase 2	EYA2	1,09
transglutaminase 7	TGM7	1,09
spermatogenesis associated 41 (non-protein coding)	SPATA41	1,09
olfactory receptor, family 5, subfamily AS, member 1	OR5AS1	1,09
zinc finger protein 273	ZNF273	1,09
apolipoprotein C-IV	APOC4	1,09
olfactory receptor, family 4, subfamily K, member 13	OR4K13	1,09
mutS homolog 6	MSH6	1,09

transmembrane protein 163	TMEM163	1,09
sarcolemma associated protein	SLMAP	1,09
SPANX family, member N1	SPANXN1	1,09
N(alpha)-acetyltransferase 15, NatA auxiliary subunit	NAA15	1,09
ralA binding protein 1	RALBP1	1,09
inter-alpha-trypsin inhibitor heavy chain family, member 6	ITIH6	1,09
ZFP28 zinc finger protein	ZFP28	1,09
THO complex 3 pseudogene; Homo sapiens THO complex 3, mRNA (cDNA clone MGC:5469 IMAGE:3451612), complete cds.; Homo sapiens THO complex 3, mRNA (cDNA clone MGC:87233 IMAGE:5262880), complete cds.; Salzman2013 ANNOTATED, INTERNAL, ncRNA, OVERLAPTX, OVEXON best transcript NR_003615; Transcript Identified by AceView, Entrez Gene ID(s) 728554	LOC728554; THOC3	1,09
cytochrome c, somatic	CYCS	1,09
wingless-type MMTV integration site family, member 9A	WNT9A	1,09
mitochondrial ribosomal protein L46	MRPL46	1,09
BMS1 ribosome biogenesis factor pseudogene 20; immunoglobulin lambda variable 1-51; immunoglobulin lambda variable 5-52	BMS1P20; IGLV1-51; IGLV5-52	1,09
double homeobox 4 like 14; double homeobox 4 like 15	DUX4L14; DUX4L15	1,09
TBC1 domain family, member 22B	TBC1D22B	1,09
single-strand-selective monofunctional uracil-DNA glycosylase 1	SMUG1	1,09
NSA2 ribosome biogenesis homolog	NSA2	1,09
mediator complex subunit 14	MED14	1,09
interferon (alpha, beta and omega) receptor 2	IFNAR2	1,09
tubulin, alpha 1b	TUBA1B	1,09
Fc fragment of IgG, high affinity Ia, receptor (CD64)	FCGR1A	1,09
MT-RNR2-like 1	MTRNR2L1	1,09
calpain, small subunit 2	CAPNS2	1,09
zinc finger protein 197	ZNF197	1,09
adaptor-related protein complex 1, mu 2 subunit	AP1M2	1,09
hemoglobin, mu	HBM	1,09
Transcript Identified by AceView, Entrez Gene ID(s) 9402; putative novel transcript	RP3-370M22.8; GRAP2	1,09
zinc finger protein 418	ZNF418	1,09
THAP7 antisense RNA 1	THAP7-AS1	1,09
ribosomal protein L36a	RPL36A	1,09
transmembrane protein 161A	TMEM161A	1,09
oxysterol binding protein-like 1A	OSBPL1A	1,09

leiomodin 1 (smooth muscle)	LMOD1	1,09
testis specific protein, Y-linked 3; testis specific protein, Y-linked 4; testis specific protein, Y-linked 8; testis specific protein, Y-linked 1	TSPY3; TSPY4; TSPY8; TSPY1	1,09
long intergenic non-protein coding RNA 612	LINC00612	1,08
TIA1 cytotoxic granule-associated RNA binding protein-like 1	TIAL1	1,08
olfactory receptor, family 2, subfamily T, member 11 (gene/pseudogene)	OR2T11	1,08
microRNA 659; ankyrin repeat domain 54	MIR659; ANKRD54	1,08
arginine and glutamate rich 1	ARGLU1	1,08
IZUMO family member 4	IZUMO4	1,08
general transcription factor IIB	GTF2B	1,08
mitogen-activated protein kinase kinase kinase 19	MAP3K19	1,08
olfactory receptor, family 13, subfamily H, member 1	OR13H1	1,08
NLR family, pyrin domain containing 2	NLRP2	1,08
phospholipase C, zeta 1	PLCZ1	1,08
purinergic receptor P2Y, G-protein coupled, 13	P2RY13	1,08
elastin microfibril interfacer 3	EMILIN3	1,08
class II, major histocompatibility complex, transactivator	CIITA	1,08
Ras association (RalGDS/AF-6) domain family (N-terminal) member 9	RASSF9	1,08
relaxin 1	RLN1	1,08
5-nucleotidase, ecto (CD73)	NT5E	1,08
matrilin 1, cartilage matrix protein	MATN1	1,08
olfactory receptor, family 6, subfamily C, member 6	OR6C6	1,08
amphiphysin	AMPH	1,08
glycerol-3-phosphate dehydrogenase 1	GPD1	1,08
transforming growth factor beta 3	TGFB3	1,08
Sin3A associated protein 18kDa	SAP18	1,08
aurora kinase A interacting protein 1	AURKAIP1	1,08
lysophospholipase I	LYPLA1	1,08
forkhead box B1	FOXB1	1,08
cilia and flagella associated protein 70	CFAP70	1,08
solute carrier family 5 (sodium/monocarboxylate cotransporter), member 12	SLC5A12	1,08
DEAD (Asp-Glu-Ala-Asp) box helicase 42	DDX42	1,08
exportin 5	XPO5	1,08
pentatricopeptide repeat domain 2	PTCD2	1,08
pleckstrin and Sec7 domain containing 2	PSD2	1,08
reticulon 4 receptor-like 1	RTN4RL1	1,08
Memczak2013 ALT_ACCEPTOR, ALT_DONOR, coding, INTERNAL, intronic best transcript NM_031453	FAM107B	1,08

protease, serine, 16 (thymus)	PRSS16	1,08
ATP binding cassette subfamily B member 8	ABCB8	1,08
insulinoma-associated 1	INSM1	1,08
leucine rich repeat containing 32	LRRC32	1,08
family with sequence similarity 218, member A	FAM218A	1,08
putative novel transcript; Transcript Identified by AceView, Entrez Gene ID(s) 6048	XXbac-BPG154L12.4; RNF5	1,08
ornithine decarboxylase antizyme 1	OAZ1	1,08
SPT16 homolog, facilitates chromatin remodeling subunit	SUPT16H	1,08
receptor (G protein-coupled) activity modifying protein 3	RAMP3	1,08
integral membrane protein 2C	ITM2C	1,08
T-box 18	TBX18	1,08
EGF-like-domain, multiple 7	EGFL7	1,08
ring finger protein 151	RNF151	1,08
NCK-associated protein 1-like	NCKAP1L	1,08
NADH dehydrogenase (ubiquinone) complex I, assembly factor 6	NDUFAF6	1,08
chromosome 11 open reading frame 16	C11orf16	1,08
WD repeat domain 64	WDR64	1,08
chromosome 1 open reading frame 68	C1orf68	1,08
phosphodiesterase 6A, cGMP-specific, rod, alpha	PDE6A	1,08
cell adhesion molecule 4	CADM4	1,08
collagen, type XIV, alpha 1	COL14A1	1,08
peptidyl arginine deiminase, type I	PADI1	1,08
RALY RNA binding protein-like	RALYL	1,08
ankyrin and armadillo repeat containing	ANKAR	1,08
olfactory receptor, family 10, subfamily G, member 8	OR10G8	1,08
nudix hydrolase 5	NUDT5	1,08
growth regulation by estrogen in breast cancer-like	GREB1L	1,08
iodotyrosine deiodinase	IYD	1,08
thioredoxin domain containing 2 (spermatozoa)	TXNDC2	1,08
Yip1 interacting factor homolog B (S. cerevisiae)	YIF1B	1,08
SH3-domain GRB2-like endophilin B1	SH3GLB1	1,08
ATPase, class V, type 10B	ATP10B	1,08
Transcript Identified by AceView, Entrez Gene ID(s) 7182	NR2C2	1,08
PRAME family member 5	PRAMEF5	1,08
zinc finger protein 44	ZNF44	1,08
interferon, alpha 4	IFNA4	1,08
zinc finger protein 132	ZNF132	1,08
ADP-ribosylation factor interacting protein 1	ARFIP1	1,08
fibroblast growth factor 5	FGF5	1,08

ATR serine/threonine kinase	ATR	1,08
kinesin family member 6	KIF6	1,08
late cornified envelope 3A	LCE3A	1,08
chemokine (C-C motif) ligand 21	CCL21	1,08
splA/ryanodine receptor domain and SOCS box containing 2	SPSB2	1,08
nuclear factor of kappa light polypeptide gene enhancer in B-cells inhibitor, delta	NFKBID	1,08
olfactory receptor, family 2, subfamily Y, member 1	OR2Y1	1,08
ras-related C3 botulinum toxin substrate 3 (rho family, small GTP binding protein Rac3)	RAC3	1,08
ring finger protein 223	RNF223	1,08
sirtuin 2	SIRT2	1,08
integrator complex subunit 5	INTS5	1,08
proteasome 26S subunit, ATPase 4 [Source:HGNC Symbol;Acc:HGNC:9551]	PSMC4	1,08
chromosome 7 open reading frame 71	C7orf71	1,08
early growth response 4	EGR4	1,08
ADAMTS like 1	ADAMTSL1	1,08
potassium channel, voltage gated KQT-like subfamily Q, member 1	KCNQ1	1,08
NADH dehydrogenase, subunit 4L (complex I); NADH dehydrogenase, subunit 4 (complex I)	ND4L; ND4	1,08
leiomodin 3 (fetal)	LMOD3	1,08
Transcript Identified by AceView, Entrez Gene ID(s) 1837	DTNA	1,08
RAB6B, member RAS oncogene family	RAB6B	1,08
solute carrier family 25, member 44	SLC25A44	1,08
achaete-scute family bHLH transcription factor 2	ASCL2	1,08
TRHDE antisense RNA 1	TRHDE-AS1	1,08
Memczak2013 ALT_ACCEPTOR, ALT_DONOR, coding, INTERNAL, intronic best transcript NM_001025108	AFF3	1,08
Transcript Identified by AceView, Entrez Gene ID(s) 50515	CHST11	1,08
ankyrin repeat domain 22	ANKRD22	1,08
calmodulin 2 (phosphorylase kinase, delta)	CALM2	1,08
tight junction associated protein 1 (peripheral)	TJAP1	1,08
C1q and tumor necrosis factor related protein 8	C1QTNF8	1,08
teneurin transmembrane protein 4	TENM4	1,08
chromosome 3 open reading frame 62	C3orf62	1,08
succinate dehydrogenase complex, subunit C, integral membrane protein, 15kDa	SDHC	1,08
chromosome 5 open reading frame 67	C5orf67	1,08
olfactory receptor, family 56, subfamily B, member 4	OR56B4	1,08
ubiquilin 4	UBQLN4	1,08

laminin, alpha 1	LAMA1	1,08
osteoclast stimulating factor 1	OSTF1	1,08
CWC15 spliceosome-associated protein	CWC15	1,08
heart and neural crest derivatives expressed 1	HAND1	1,08
BCL2-like 14 (apoptosis facilitator)	BCL2L14	1,08
armadillo repeat containing, X-linked 5	ARMCX5	1,08
transmembrane protein 42; microRNA 564	TMEM42; MIR564	1,08
phospholipid phosphatase related 4	PLPPR4	1,08
chromosome transmission fidelity factor 8	CHTF8	1,08
chromosome 1 open reading frame 226	C1orf226	1,08
family with sequence similarity 46, member C	FAM46C	1,08
family with sequence similarity 159, member B	FAM159B	1,08
Transcript Identified by AceView, Entrez Gene ID(s) 50515	CHST11	1,08
casein kappa	CSN3	1,08
spermatogenesis associated 6	SPATA6	1,08
interleukin 1 alpha	IL1A	1,08
cyclic nucleotide binding domain containing 2	CNBD2	1,08
c-Maf inducing protein	CMIP	1,08
carnosine synthase 1	CARNS1	1,08
potassium channel, inwardly rectifying subfamily J, member 2	KCNJ2	1,08
protein phosphatase, Mg2+/Mn2+ dependent, 1B	PPM1B	1,08
transmembrane protein 108	TMEM108	1,08
centromere protein L	CENPL	1,08
Transcript Identified by AceView, Entrez Gene ID(s) 23355	VPS8	1,08
chromosome 1 open reading frame 21	C1orf21	1,08
TGFB-induced factor homeobox 2-like, X-linked	TGIF2LX	1,08
p21 protein (Cdc42/Rac)-activated kinase 6; BUB1 mitotic checkpoint serine/threonine kinase B	PAK6; BUB1B	1,08
chaperonin containing TCP1, subunit 3 (gamma)	CCT3	1,08
neuralized E3 ubiquitin protein ligase 1	NEURL1	1,08
olfactory receptor, family 5, subfamily G, member 5 pseudogene; olfactory receptor, family 5, subfamily G, member 3 (gene/pseudogene)	OR5G5P; OR5G3	1,08
trefoil factor 3	TFF3	1,08
calpain 13	CAPN13	1,08
coiled-coil domain containing 89	CCDC89	1,08
ADAM metallopeptidase with thrombospondin type 1 motif 14	ADAMTS14	1,08
gon-4-like (C. elegans)	GON4L	1,08
family with sequence similarity 71, member F2	FAM71F2	1,08
PRAME family member 11	PRAMEF11	1,08

germinal center-associated, signaling and motility	GCSAM	1,08
centrin 1	CETN1	1,08
WAS protein homolog associated with actin, golgi membranes and microtubules	WHAMM	1,08
EF-hand calcium binding domain 8	EFCAB8	1,08
cullin 9	CUL9	1,08
GRIP and coiled-coil domain containing 1	GCC1	1,08
somatomedin B and thrombospondin type 1 domain containing	SBSPON	1,08
LSM5 homolog, U6 small nuclear RNA and mRNA degradation associated	LSM5	1,08
potassium channel, two pore domain subfamily K, member 17	KCNK17	1,08
fibronectin type III domain containing 7	FNDC7	1,08
ribosome binding factor A (putative); RBFA downstream neighbor (non-protein coding)	RBFA; RBFADN	1,08
sema domain, immunoglobulin domain (Ig), short basic domain, secreted, (semaphorin) 3D	SEMA3D	1,08
proline rich 14	PRR14	1,08
phosphodiesterase 1B, calmodulin-dependent	PDE1B	1,08
tumor protein p53 inducible nuclear protein 2	TP53INP2	1,08
NOP2/Sun domain family, member 6	NSUN6	1,08
olfactory receptor, family 1, subfamily S, member 2	OR1S2	1,08
oxoglutarate (alpha-ketoglutarate) dehydrogenase (lipoamide)	OGDH	1,08
complement component 9	C9	1,08
elaC ribonuclease Z 1	ELAC1	1,08
TATA box binding protein associated factor 3	TAF3	1,08
protein tyrosine phosphatase, receptor type, O	PTPRO	1,08
synaptotagmin-like 5	SYTL5	1,08
BCL2-like 14 (apoptosis facilitator)	BCL2L14	1,08
mitochondrial translational initiation factor 2	MTIF2	1,08
chromosome 15 open reading frame 59	C15orf59	1,08
glucosaminyl (N-acetyl) transferase family member 7	GCNT7	1,08
mitogen-activated protein kinase kinase kinase 10	MAP3K10	1,08
arginine-fifty homeobox	ARGFX	1,08
protocadherin 20	PCDH20	1,08
glycine receptor alpha 4	GLRA4	1,08
zinc finger, DHHC-type containing 3	ZDHHC3	1,08
junctophilin 1	JPH1	1,08
neuronal differentiation 2	NEUROD2	1,08
adenosine monophosphate deaminase 1	AMPD1	1,08
olfactory receptor, family 10, subfamily H, member 4	OR10H4	1,08
integrin beta 5	ITGB5	1,08

olfactory receptor, family 52, subfamily B, member 4 (gene/pseudogene)	OR52B4	1,08
phenylethanolamine N-methyltransferase	PNMT	1,08
Transcript Identified by AceView, Entrez Gene ID(s) 5987	TRIM27	1,08
activating transcription factor 2	ATF2	1,08
erythroblast membrane-associated protein (Scianna blood group)	ERMAP	1,08
neurensin 1	NRSN1	1,08
Kv channel interacting protein 3, calsenilin	KCNIP3	1,08
Memczak2013 ALT_ACCEPTOR, ALT_DONOR, coding, INTERNAL, intronic best transcript NM_014938	MLXIP	1,08
BTB (POZ) domain containing 17	BTBD17	1,08
core-binding factor, runt domain, alpha subunit 2; translocated to, 3	CBFA2T3	1,08
galanin/GMAP prepropeptide	GAL	1,08
tRNA isopentenyltransferase 1	TRIT1	1,08
X-linked Kx blood group related, X-linked	XKRX	1,08
zinc finger, C3H1-type containing	ZFC3H1	1,08
promyelocytic leukemia	PML	1,08
potassium channel, inwardly rectifying subfamily J, member 3	KCNJ3	1,08
AKNA domain containing 1	AKNAD1	1,08
dynein, axonemal, heavy chain 5	DNAH5	1,08
UDP-Gal:betaGlcNAc beta 1,3-galactosyltransferase 5	B3GALT5	1,08
taste receptor, type 2, member 4	TAS2R4	1,08
olfactory receptor, family 2, subfamily W, member 5 (gene/pseudogene)	OR2W5	1,08
ribosomal protein S4, X-linked	RPS4X	1,08
alcohol dehydrogenase 1A (class I), alpha polypeptide	ADH1A	1,08
cAMP responsive element binding protein 3-like 1	CREB3L1	1,08
long intergenic non-protein coding RNA 1559	LINC01559	1,08
major histocompatibility complex, class II, DQ alpha 1	HLA-DQA1	1,08
family with sequence similarity 46, member D	FAM46D	1,08
olfactory receptor, family 8, subfamily D, member 4	OR8D4	1,08
gap junction protein delta 2	GJD2	1,08
ribosomal protein S6 kinase, 90kDa, polypeptide 1	RPS6KA1	1,08
frizzled class receptor 10	FZD10	1,08
neuronal tyrosine-phosphorylated phosphoinositide-3-kinase adaptor 1	NYAP1	1,08
A kinase (PRKA) anchor protein 6	AKAP6	1,08
transcription factor 19	TCF19	1,08
transmembrane protein 253	TMEM253	1,08
POC1 centriolar protein B; POC1B-GALNT4 readthrough; polypeptide N-acetylgalactosaminyltransferase 4	POC1B; POC1B-GALNT4; GALNT4	1,08

KIAA1024-like	KIAA1024L	1,08
family with sequence similarity 53, member A	FAM53A	1,08
TBK1 binding protein 1	TBKBP1	1,08
GLI pathogenesis-related 1 like 2	GLIPR1L2	1,08
NK2 homeobox 2	NKX2-2	1,08
mastermind-like transcriptional coactivator 1	MAML1	1,08
PIK3CD antisense RNA 1	PIK3CD-AS1	1,08
luteinizing hormone beta polypeptide	LHB	1,08
ankyrin repeat domain 49	ANKRD49	1,08
outer dense fiber of sperm tails 3-like 1	ODF3L1	1,08
fatty acyl-CoA reductase 2	FAR2	1,08
ADP-ribosylation factor GTPase activating protein 2	ARFGAP2	1,08
tryptophan rich basic protein	WRB	1,08
interleukin 1 receptor-like 1	IL1RL1	1,08
neuropeptide Y receptor Y5	NPY5R	1,08
ADAM metallopeptidase with thrombospondin type 1 motif 13	ADAMTS13	1,08
translocation associated membrane protein 2	TRAM2	1,08
placenta specific 4	PLAC4	1,08
sorting nexin 32	SNX32	1,08
renalase, FAD-dependent amine oxidase	RNLS	1,08
decorin	DCN	1,08
Transcript Identified by AceView, Entrez Gene ID(s) 23355	VPS8	1,08
synaptotagmin I	SYT1	1,08
synaptotagmin XVI	SYT16	1,08
Zic family member 1	ZIC1	1,08
Salzman2013 ALT_ACCEPTOR, ALT_DONOR, coding, INTERNAL, intronic best transcript NM_007112	THBS3	1,08
tumor protein p73	TP73	1,08
ubiquitin specific peptidase 35	USP35	1,08
interleukin 1 family, member 10 (theta)	IL1F10	1,08
MAM domain containing glycosylphosphatidylinositol anchor 2	MDGA2	1,08
solute carrier family 1 (neutral amino acid transporter), member 5	SLC1A5	1,08
vomeronasal 1 receptor 3 (gene/pseudogene)	VN1R3	1,08
UDP-glucose pyrophosphorylase 2	UGP2	1,08
zinc finger protein 623	ZNF623	1,08
ribosomal protein S28	RPS28	1,07
fibrillin 2	FBN2	1,07
PRAME family member 6 [Source:HGNC Symbol;Acc:HGNC:30583]	PRAMEF6	1,07
PRAME family member 6 [Source:HGNC Symbol;Acc:HGNC:30583]	PRAMEF6	1,07

t-SNARE domain containing 1	TSNARE1	1,07
serpin peptidase inhibitor, clade B (ovalbumin), member 13	SERPINB13	1,07
carbonic anhydrase VII	CA7	1,07
Rho GTPase activating protein 39	ARHGAP39	1,07
zinc finger protein 575	ZNF575	1,07
bactericidal/permeability-increasing protein	BPI	1,07
BTB (POZ) domain containing 6	BTBD6	1,07
B-cell scaffold protein with ankyrin repeats 1	BANK1	1,07
MLX interacting protein	MLXIP	1,07
homeobox D8	HOXD8	1,07
Jeck2013 ALT_ACCEPTOR, ALT_DONOR, coding, INTERNAL, intronic best transcript NM_004996	ABCC1	1,07
sarcospan	SSPN	1,07
carboxyl ester lipase	CEL	1,07
Zhang2013 ALT_ACCEPTOR, ALT_DONOR, coding, INTERNAL, intronic best transcript NM_002882	RANBP1	1,07
olfactory receptor, family 52, subfamily D, member 1	OR52D1	1,07
family with sequence similarity 27, member E3	FAM27E3	1,07
rhophilin associated tail protein 1	ROPN1	1,07
regenerating islet-derived 3 gamma	REG3G	1,07
solute carrier family 22 (organic cation/zwitterion transporter), member 4	SLC22A4	1,07
melanocortin 5 receptor	MC5R	1,07
plexin C1	PLXNC1	1,07
small integral membrane protein 10 like 1	SMIM10L1	1,07
bromodomain adjacent to zinc finger domain 2A	BAZ2A	1,07
coiled-coil domain containing 22	CCDC22	1,07
calcium/calmodulin-dependent protein kinase IG	CAMK1G	1,07
G protein-coupled receptor 183	GPR183	1,07
thyroid hormone receptor interactor 11	TRIP11	1,07
ankyrin repeat and SOCS box containing 6	ASB6	1,07
transmembrane protein 132D	TMEM132D	1,07
transcription factor AP-2 beta (activating enhancer binding protein 2 beta)	TFAP2B	1,07
homer scaffolding protein 3	HOMER3	1,07
lymphoid-restricted membrane protein	LRMP	1,07
uncharacterized LOC100144595; novel transcript; Transcript Identified by AceView	LOC100144595; AC009227.2; tikure	1,07
transmembrane 4 L six family member 1	TM4SF1	1,07
UDP-GlcNAc:betaGal beta-1,3-N-acetylglucosaminyltransferase 8	B3GNT8	1,07
ankyrin repeat and SOCS box containing 15	ASB15	1,07

olfactory receptor, family 1, subfamily D, member 4 (gene/pseudogene)	OR1D4	1,07
lipase A, lysosomal acid, cholesterol esterase	LIPA	1,07
proline rich 18	PRR18	1,07
RNA binding motif protein, X-linked 2	RBMX2	1,07
family with sequence similarity 177, member A1	FAM177A1	1,07
BCL2-like 14 (apoptosis facilitator)	BCL2L14	1,07
phospholipid scramblase family, member 5	PLSCR5	1,07
GINS complex subunit 3 (Psf3 homolog)	GINS3	1,07
dual specificity tyrosine-(Y)-phosphorylation regulated kinase 1B	DYRK1B	1,07
chemokine (C-C motif) ligand 3	CCL3	1,07
protein phosphatase 6, regulatory subunit 1	PPP6R1	1,07
zinc finger protein 208	ZNF208	1,07
chemokine (C-C motif) ligand 17	CCL17	1,07
G antigen 12C; G antigen 12E; G antigen 12H	GAGE12C; GAGE12E; GAGE12H	1,07
zinc finger protein 665	ZNF665	1,07
lysine (K)-specific demethylase 4E	KDM4E	1,07
FERM domain containing 8	FRMD8	1,07
lectin, galactoside-binding-like	LGALSL	1,07
solute carrier family 26 (anion exchanger), member 7	SLC26A7	1,07
ADAM metallopeptidase with thrombospondin type 1 motif 3	ADAMTS3	1,07
cyclin-dependent kinase 15	CDK15	1,07
EF-hand calcium binding domain 6	EFCAB6	1,07
PRAME family member 12	PRAMEF12	1,07
sperm flagellar 2	SPEF2	1,07
forkhead box R1	FOXR1	1,07
keratin associated protein 4-2	KRTAP4-2	1,07
retinitis pigmentosa 1-like 1	RP1L1	1,07
RAS-like, family 10, member A	RASL10A	1,07
rabenosyn, RAB effector	RBSN	1,07
zyg-11 family member A, cell cycle regulator	ZYG11A	1,07
Transcript Identified by AceView, Entrez Gene ID(s) 57448	BIRC6	1,07
hydroxyacylglutathione hydrolase-like	HAGHL	1,07
homeobox C13	HOXC13	1,07
proline/histidine/glycine-rich 1	PHGR1	1,07
zinc finger protein 793	ZNF793	1,07
cyclin-dependent kinase-like 2 (CDC2-related kinase)	CDKL2	1,07
retinol dehydrogenase 5 (11-cis/9-cis)	RDH5	1,07
SOGA family member 3; KIAA0408	SOGA3; KIAA0408	1,07

TATA box binding protein (TBP)-associated factor, RNA polymerase I, C, 110kDa	TAF1C	1,07
transmembrane protein 190	TMEM190	1,07
sorting nexin family member 30	SNX30	1,07
YTH N(6)-methyladenosine RNA binding protein 3	YTHDF3	1,07
olfactory receptor, family 13, subfamily C, member 9	OR13C9	1,07
calcium binding protein 2	CABP2	1,07
cyclin-dependent kinase 5, regulatory subunit 2 (p39)	CDK5R2	1,07
keratin 28, type I	KRT28	1,07
methyltransferase like 13	METTL13	1,07
macrophage stimulating 1 receptor	MST1R	1,07
testis expressed sequence 13-like	LOC100129520	1,07
mitogen-activated protein kinase kinase kinase kinase 3	MAP4K3	1,07
Memczak2013 ANTISENSE, INTERNAL, intronic, ncRNA best transcript NR_024568	FBXO31	1,07
double C2-like domains, alpha	DOC2A	1,07
zonadhesin (gene/pseudogene)	ZAN	1,07
uncharacterized LOC101927572; Memczak2013 ANTISENSE, CDS, coding, INTERNAL, intronic best transcript NM_015526; novel transcript, antisense to CLIP3 and THAP8	LOC101927572; AC002116.7; CLIP3	1,07
StAR-related lipid transfer domain containing 5	STARD5	1,07
BCL2-like 14 (apoptosis facilitator)	BCL2L14	1,07
dynein, axonemal, intermediate chain 1	DNAI1	1,07
tigger transposable element derived 6	TIGD6	1,07
FERM and PDZ domain containing 2	FRMPD2	1,07
chromosome 9 open reading frame 91	C9orf91	1,07
proline/arginine-rich end leucine-rich repeat protein	PRELP	1,07
solute carrier family 26, member 10	SLC26A10	1,07
murine retrovirus integration site 1 homolog	MRVI1	1,07
chromosome 6 open reading frame 118	C6orf118	1,07
zinc finger protein 469	ZNF469	1,07
ALG9, alpha-1,2-mannosyltransferase	ALG9	1,07
nucleosome assembly protein 1-like 2	NAP1L2	1,07
mediator of DNA-damage checkpoint 1	MDC1	1,07
proteasome 26S subunit, non-ATPase 7	PSMD7	1,07
Memczak2013 ALT_ACCEPTOR, ALT_DONOR, coding, INTERNAL, intronic best transcript NM_014957	DENND3	1,07
chemokine (C-C motif) ligand 4-like 2; chemokine (C-C motif) ligand 4-like 1	CCL4L2; CCL4L1	1,07
deleted in azoospermia 3	DAZ3	1,07
dipeptidyl-peptidase 7	DPP7	1,07
homeobox A5	HOXA5	1,07
pyridine nucleotide-disulphide oxidoreductase domain 2; microRNA 1287	PYROXD2; MIR1287	1,07

solute carrier family 22 (organic anion/urate transporter), member 13	SLC22A13	1,07
cystatin 9-like	CST9L	1,07
adherens junctions associated protein 1	AJAP1	1,07
S100 calcium binding protein Z	S100Z	1,07
Memczak2013 ALT_ACCEPTOR, ALT_DONOR, coding, INTERNAL, intronic best transcript NM_022172	PC	1,07
aminopeptidase puromycin sensitive	NPEPPS	1,07
testis-specific serine kinase 3	TSSK3	1,07
zinc finger protein 257	ZNF257	1,07
Memczak2013 ANTISENSE, CDS, coding, INTERNAL best transcript NM_133502	ZNF274	1,07
interleukin 27	IL27	1,07
keratin 86, type II	KRT86	1,07
PRAME family member 19 [Source:EntrezGene;Acc:645414]	PRAMEF19	1,07
exonuclease 3-5 domain containing 1	EXD1	1,07
NACHT and WD repeat domain containing 1	NWD1	1,07
collagen, type XXII, alpha 1	COL22A1	1,07
aquaporin 12B	AQP12B	1,07
ankyrin repeat and SOCS box containing 10	ASB10	1,07
3-hydroxymethyl-3-methylglutaryl-CoA lyase	HMGCL	1,07
MAS-related GPR, member X1	MRGPRX1	1,07
interleukin 16	IL16	1,07
cytochrome b5 domain containing 1	CYB5D1	1,07
grainyhead-like transcription factor 3	GRHL3	1,07
synaptonemal complex protein 2	SYCP2	1,07
KIAA0226-like	KIAA0226L	1,07
leucine rich repeat containing 74A	LRRC74A	1,07
junctophilin 3	JPH3	1,07
hypocretin (orexin) receptor 2	HCRTR2	1,07
transmembrane protein 175	TMEM175	1,07
coiled-coil domain containing 57	CCDC57	1,07
syntaxin 6	STX6	1,07
stimulator of chondrogenesis 1	SCRG1	1,07
arachidonate 15-lipoxygenase	ALOX15	1,07
tripartite motif family like 1	TRIML1	1,07
formin homology 2 domain containing 1	FHOD1	1,07
thyroid hormone receptor interactor 6; microRNA 6875	TRIP6; MIR6875	1,07
cornichon family AMPA receptor auxiliary protein 3	CNIH3	1,07
zinc finger protein 214	ZNF214	1,07
cingulin	CGN	1,07
insulin-like 3 (Leydig cell)	INSL3	1,07
melatonin receptor 1A	MTNR1A	1,07
zinc finger protein 561	ZNF561	1,07

guanylate cyclase 1, soluble, alpha 3	GUCY1A3	1,07
HtrA serine peptidase 4	HTRA4	1,07
eukaryotic translation initiation factor 2D	EIF2D	1,07
olfactory receptor family 10 subfamily A member 6 (gene/pseudogene) [Source:HGNC Symbol;Acc:HGNC:15132]	OR10A6	1,07
dynein, axonemal, heavy chain 7	DNAH7	1,07
proteasome subunit alpha 1	PSMA1	1,07
ethanolamine kinase 2	ETNK2	1,07
olfactory receptor, family 2, subfamily M, member 4	OR2M4	1,07
Rho GTPase activating protein 20	ARHGAP20	1,07
karyopherin alpha 2 (RAG cohort 1, importin alpha 1)	KPNA2	1,07
Jeck2013 ANTISENSE, CDS, coding, INTERNAL, intronic, OVCODE, OVEXON best transcript NM_017491	WDR1	1,07
integral membrane protein 2A	ITM2A	1,07
lysine (K)-specific methyltransferase 2C	KMT2C	1,07
BCL2 associated athanogene 6	BAG6	1,07
glycophorin E (MNS blood group)	GYPE	1,07
nuclear cap binding protein subunit 2	NCBP2	1,07
histone cluster 1, H4h	HIST1H4H	1,07
family with sequence similarity 163, member B	FAM163B	1,07
terminal uridylyl transferase 1, U6 snRNA-specific	TUT1	1,07
chromosome 10 open reading frame 55	C10orf55	1,07
atypical chemokine receptor 4	ACKR4	1,07
caspase recruitment domain family, member 11	CARD11	1,07
SAM pointed domain containing ETS transcription factor	SPDEF	1,07
POU class 2 associating factor 1	POU2AF1	1,07
potassium channel, voltage gated KQT-like subfamily Q, member 2	KCNQ2	1,07
corneodesmosin	CDSN	1,07
spindle and kinetochore associated complex subunit 2	SKA2	1,07
membrane associated ring finger 1	MARCH1	1,07
olfactory receptor, family 13, subfamily F, member 1	OR13F1	1,07
serine palmitoyltransferase, long chain base subunit 1	SPTLC1	1,07
angiopoietin like 6	ANGPTL6	1,07
zinc finger, BED-type containing 1; dehydrogenase/reductase (SDR family) X-linked	ZBED1; DHRSX	1,07
taste receptor, type 1, member 1	TAS1R1	1,07
Memczak2013 ANTISENSE, coding, INTERNAL, intronic best transcript NM_001098633	AKT1S1	1,07
teashirt zinc finger homeobox 2	TSHZ2	1,07
zinc finger protein 813	ZNF813	1,07
ankyrin repeat domain 27 (VPS9 domain)	ANKRD27	1,07
coiled-coil domain containing 159	CCDC159	1,07
family with sequence similarity 84, member A	FAM84A	1,07

amine oxidase, copper containing 1	AOC1	1,07
luteinizing hormone/choriogonadotropin receptor	LHCGR	1,07
hyperpolarization activated cyclic nucleotide gated potassium channel 3	HCN3	1,07
solute carrier family 41, member 3	SLC41A3	1,07
cell adhesion molecule 2	CADM2	1,07
synuclein beta	SNCB	1,07
DEAD (Asp-Glu-Ala-Asp) box polypeptide 4	DDX4	1,07
pro-platelet basic protein	PPBP	1,07
methyl-CpG binding domain protein 5	MBD5	1,07
collagen, type XXIV, alpha 1	COL24A1	1,07
FBJ murine osteosarcoma viral oncogene homolog B	FOSB	1,07
family with sequence similarity 180, member A	FAM180A	1,07
growth hormone secretagogue receptor	GHSR	1,07
visual system homeobox 1	VSX1	1,07
MCF.2 cell line derived transforming sequence-like 2	MCF2L2	1,07
podocan-like 1	PODNL1	1,07
NADH dehydrogenase (ubiquinone) 1 alpha subcomplex, 10, 42kDa	NDUFA10	1,07
nephroblastoma overexpressed	NOV	1,07
tumor necrosis factor	TNF	1,07
mannose-binding lectin (protein A) 1, pseudogene; BMS1 ribosome biogenesis factor pseudogene 21	MBL1P; BMS1P21	1,07
Rho GTPase activating protein 22	ARHGAP22	1,07
family with sequence similarity 234, member A; Rho GDP dissociation inhibitor (GDI) gamma	FAM234A; ARHGDIG	1,07
UDP glycosyltransferase 3 family, polypeptide A2	UGT3A2	1,07
serine/threonine kinase receptor associated protein	STRAP	1,07
Transcript Identified by AceView, Entrez Gene ID(s) 79781	IQCA1	1,07
fibroblast growth factor 19	FGF19	1,07
four and a half LIM domains 1	FHL1	1,07
butyrophilin-like 9	BTNL9	1,07
GLIS family zinc finger 1	GLIS1	1,07
phosphatidylinositol-5-phosphate 4-kinase, type II, alpha	PIP4K2A	1,07
eukaryotic translation initiation factor 4E family member 2	EIF4E2	1,07
sorting nexin 11	SNX11	1,07
neural cell adhesion molecule 1	NCAM1	1,07
pancreatic and duodenal homeobox 1	PDX1	1,07
mesogenin 1	MSGN1	1,07
F-box and leucine-rich repeat protein 22	FBXL22	1,07
family with sequence similarity 83, member B	FAM83B	1,07

peptidyl-prolyl cis-trans isomerase A-like 4G; Peptidyl-prolyl cis-trans isomerase [Source:UniProtKB/TrEMBL;Acc:A0A075B767]	LOC105371242; AC243756.1	1,07
bone morphogenetic protein 4	BMP4	1,07
chromosome transmission fidelity factor 18	CHTF18	1,07
Transcript Identified by AceView, Entrez Gene ID(s) 60468	BACH2	1,07
secretory carrier membrane protein 2	SCAMP2	1,07
midline 1	MID1	1,07
immunoglobulin superfamily, member 5	IGSF5	1,07
leucine rich repeat containing 31	LRRC31	1,07
tRNA phosphotransferase 1	TRPT1	1,07
zinc finger protein 197	ZNF197	1,07
autophagy related 16-like 1	ATG16L1	1,07
coiled-coil domain containing 152	CCDC152	1,07
protein phosphatase 1, regulatory subunit 16A	PPP1R16A	1,07
ring finger protein 181	RNF181	1,07
replication factor C subunit 3	RFC3	1,07
KRTAP5-1/KRTAP5-2 antisense RNA 1	KRTAP5-AS1	1,07
PMS1 homolog 2, mismatch repair system component	PMS2	1,07
coiled-coil domain containing 175	CCDC175	1,07
microfibrillar associated protein 3	MFAP3	1,07
solute carrier family 6 (neurotransmitter transporter), member 13	SLC6A13	1,07
cytochrome c oxidase subunit VIa polypeptide 2	COX6A2	1,07
transmembrane protein 255B	TMEM255B	1,07
tripartite motif containing 43	TRIM43	1,07
core 1 synthase, glycoprotein-N-acetylgalactosamine 3-beta-galactosyltransferase 1	C1GALT1	1,07
lactate dehydrogenase A-like 6A	LDHAL6A	1,07
immunoglobulin superfamily, member 9	IGSF9	1,07
ATP synthase, H+ transporting, mitochondrial Fo complex subunit D	ATP5H	1,06
MAGE family member B5	MAGEB5	1,06
lipocalin 2	LCN2	1,06
SH3-domain GRB2-like (endophilin) interacting protein 1	SGIP1	1,06
proteasome 26S subunit, non-ATPase 3	PSMD3	1,06
poly(ADP-ribose) polymerase family member 16	PARP16	1,06
transcription factor 20 (AR1)	TCF20	1,06
transcription factor 20 (AR1)	TCF20	1,06
polymerase (RNA) I polypeptide E	POLR1E	1,06
cholinergic receptor, nicotinic gamma	CHRNG	1,06
Memczak2013 ALT_ACCEPTOR, ALT_DONOR, coding, INTERNAL, intronic best transcript NM_014713	LAPTM4A	1,06

chromosome 7 open reading frame 57	C7orf57	1,06
sperm flagellar 1	SPEF1	1,06
neuropeptides B/W receptor 1	NPBWR1	1,06
potassium channel, voltage gated shaker related subfamily A, member 5	KCNA5	1,06
keratin 79, type II	KRT79	1,06
discoidin domain receptor tyrosine kinase 2	DDR2	1,06
homeobox C6; homeobox C4; homeobox C5	HOXC6; HOXC4; HOXC5	1,06
leucine-rich repeats and calponin homology (CH) domain containing 2	LRCH2	1,06
ubiquitin A-52 residue ribosomal protein fusion product 1	UBA52	1,06
ATPase, H+ transporting, lysosomal 42kDa, V1 subunit C2	ATP6V1C2	1,06
polypeptide N-acetylgalactosaminyltransferase 11	GALNT11	1,06
scleraxis bHLH transcription factor	SCX	1,06
cancer/testis antigen 83	CT83	1,06
KIAA1644	KIAA1644	1,06
T-cell acute lymphocytic leukemia 1	TAL1	1,06
small integral membrane protein 7; mediator complex subunit 26	SMIM7; MED26	1,06
retinitis pigmentosa GTPase regulator interacting protein 1	RPGRIP1	1,06
mannose receptor, C type 1	MRC1	1,06
Transcript Identified by AceView, Entrez Gene ID(s) 90485	ZNF835	1,06
Memczak2013 ANTISENSE, coding, INTERNAL, intronic best transcript NM_001040442	FABP6	1,06
CKLF-CMTM1 readthrough	CKLF-CMTM1	1,06
tripartite motif containing 49C	TRIM49C	1,06
zinc finger and BTB domain containing 9	ZBTB9	1,06
nebulin-related anchoring protein	NRAP	1,06
HORMA domain containing 2	HORMAD2	1,06
chronic lymphocytic leukemia up-regulated 1 opposite strand	CLLU1OS	1,06
proline rich 23A	PRR23A	1,06
chromosome 5 open reading frame 60	C5orf60	1,06
mitochondrial ribosomal protein S7	MRPS7	1,06
phospholipase C, beta 4	PLCB4	1,06
deiodinase, iodothyronine, type III	DIO3	1,06
death associated protein like 1; olfactory receptor, family 7, subfamily E, member 89 pseudogene	DAPL1; OR7E89P	1,06
zinc finger protein 138	ZNF138	1,06
Fanconi anemia complementation group I	FANCI	1,06

CD300c molecule	CD300C	1,06
UNC homeobox	UNCX	1,06
RNA binding motif protein 42	RBM42	1,06
stathmin domain containing 1	STMND1	1,06
ADIRF antisense RNA 1 [Source:HGNC Symbol;Acc:HGNC:45127]; Transcript Identified by AceView, Entrez Gene ID(s) 79812; ADIRF antisense RNA 1; novel transcript, antisense to ADIRF	ADIRF-AS1; RP11-96C23.15; MMRN2	1,06
golgin A6 family, member B	GOLGA6B	1,06
mitochondrial calcium uptake 2	MICU2	1,06
zinc finger protein 408	ZNF408	1,06
neuron navigator 1	NAV1	1,06
adenylate cyclase 3	ADCY3	1,06
peptide YY	PYY	1,06
kinesin family member 26A	KIF26A	1,06
leishmanolysin-like (metallopeptidase M8 family)	LMLN	1,06
triadin	TRDN	1,06
transmembrane protease, serine 7	TMPRSS7	1,06
NLR family, pyrin domain containing 7	NLRP7	1,06
protein phosphatase 1, regulatory subunit 37	PPP1R37	1,06
G-patch domain containing 3	GPATCH3	1,06
lipase, family member J	LIPJ	1,06
solute carrier family 22 (organic cation transporter), member 2	SLC22A2	1,06
FEZ family zinc finger 2	FEZF2	1,06
nucleoporin 62kDa C-terminal like	NUP62CL	1,06
xylulokinase homolog (H. influenzae)	XYLB	1,06
xin actin binding repeat containing 1	XIRP1	1,06
DnaJ (Hsp40) homolog, subfamily C, member 11	DNAJC11	1,06
janus kinase and microtubule interacting protein 1	JAKMIP1	1,06
neuronal PAS domain protein 4	NPAS4	1,06
pentraxin 4, long	PTX4	1,06
taste receptor, type 2, member 9	TAS2R9	1,06
keratin associated protein 21-3	KRTAP21-3	1,06
HOXB cluster antisense RNA 3	HOXB-AS3	1,06
exportin 1	XPO1	1,06
exocyst complex component 2	EXOC2	1,06
glutamine-rich 1	QRICH1	1,06
COMM domain containing 7	COMMD7	1,06
outer dense fiber of sperm tails 3B	ODF3B	1,06
dynein, cytoplasmic 1, light intermediate chain 1	DYNC1LI1	1,06
bradykinin receptor B1	BDKRB1	1,06
family with sequence similarity 71, member F1	FAM71F1	1,06
alkaline phosphatase, intestinal	ALPI	1,06
zinc finger protein 197	ZNF197	1,06

LYR motif containing 2	LYRM2	1,06
ARMCX5-GPRASP2 readthrough	ARMCX5-GPRASP2	1,06
olfactory receptor, family 7, subfamily G, member 2	OR7G2	1,06
zinc finger, C2HC-type containing 1C	ZC2HC1C	1,06
TSR2, 20S rRNA accumulation, homolog (S. cerevisiae)	TSR2	1,06
Zhang2013 ALT_ACCEPTOR, ALT_DONOR, coding, INTERNAL, intronic best transcript NM_012398	PIP5K1C	1,06
zinc finger protein 106	ZNF106	1,06
sema domain, immunoglobulin domain (Ig), transmembrane domain (TM) and short cytoplasmic domain, (semaphorin) 4A	SEMA4A	1,06
coiled-coil domain containing 13	CCDC13	1,06
prostate and testis expressed 3	PATE3	1,06
suppressor of variegation 3-9 homolog 1 (Drosophila)	SUV39H1	1,06
G protein-coupled receptor 161	GPR161	1,06
calcium and integrin binding family member 3	CIB3	1,06
NADH dehydrogenase (ubiquinone) Fe-S protein 2, 49kDa (NADH-coenzyme Q reductase)	NDUFS2	1,06
vacuolar protein sorting 13 homolog D (S. cerevisiae)	VPS13D	1,06
ribosomal protein L7a	RPL7A	1,06
tankyrase, TRF1-interacting ankyrin-related ADP-ribose polymerase 2	TNKS2	1,06
solute carrier family 22 (organic anion/urate transporter), member 12	SLC22A12	1,06
sonic hedgehog	SHH	1,06
transmembrane protease, serine 2	TMPRSS2	1,06
olfactory receptor, family 2, subfamily T, member 35	OR2T35	1,06
ring finger protein 222	RNF222	1,06
UTP6, small subunit (SSU) processome component, homolog (yeast)	UTP6	1,06
musculoskeletal, embryonic nuclear protein 1	MUSTN1	1,06
transglutaminase 4	TGM4	1,06
golgi-associated, gamma adaptin ear containing, ARF binding protein 2	GGA2	1,06
chronic lymphocytic leukemia up-regulated 1	CLLU1	1,06
transcription factor AP-2 alpha (activating enhancer binding protein 2 alpha)	TFAP2A	1,06
von Willebrand factor A domain containing 5B1	VWA5B1	1,06
huntingtin interacting protein K	HYPK	1,06
ceramide synthase 6	CERS6	1,06
double homeobox 4 like 8	DUX4L8	1,06
solute carrier family 17 (vesicular glutamate transporter), member 7	SLC17A7	1,06
paired-like homeodomain 2	PITX2	1,06

t-complex 10-like 2	TCP10L2	1,06
sema domain, seven thrombospondin repeats (type 1 and type 1-like), transmembrane domain (TM) and short cytoplasmic domain, (semaphorin) 5A	SEMA5A	1,06
spermatogenesis associated 19	SPATA19	1,06
olfactory receptor, family 52, subfamily E, member 2	OR52E2	1,06
solute carrier family 2 (facilitated glucose transporter), member 6 [Source:HGNC Symbol;Acc:HGNC:11011]	SLC2A6	1,06
tubulin folding cofactor D	TBCD	1,06
chromosome 2 open reading frame 57	C2orf57	1,06
ATPase, H+ transporting, lysosomal accessory protein 1-like	ATP6AP1L	1,06
developmental pluripotency associated 3	DPPA3	1,06
SPATA31 subfamily A, member 1	SPATA31A1	1,06
olfactory receptor, family 2, subfamily T, member 27	OR2T27	1,06
family with sequence similarity 131, member B	FAM131B	1,06
acetylserotonin O-methyltransferase; A kinase (PRKA) anchor protein 17A	ASMT; AKAP17A	1,06
Homo sapiens acetylserotonin O-methyltransferase (ASMT), transcript variant 1, mRNA.; Homo sapiens acetylserotonin O-methyltransferase (ASMT), transcript variant 3, mRNA.; acetylserotonin O-methyltransferase [Source:HGNC Symbol;Acc:HGNC:750]; Homo sapiens acetylserotonin O-methyltransferase, mRNA (cDNA clone MGC:2145 IMAGE:3356470), complete cds.; Transcript Identified by AceView, Entrez Gene ID(s) 438; 8227, RefSeq ID(s) NM_001171038	ASMT; SFRS17AandASMT	1,06
potassium channel, sodium activated subfamily T, member 1	KCNT1	1,06
anaplastic lymphoma receptor tyrosine kinase	ALK	1,06
Memczak2013 ALT_ACCEPTOR, ALT_DONOR, coding, INTERNAL, intronic best transcript NM_003036; Transcript Identified by AceView	SKI; kerlabu	1,06
Fer3-like bHLH transcription factor	FERD3L	1,06
maestro heat-like repeat family member 2B	MROH2B	1,06
family with sequence similarity 104, member B	FAM104B	1,06
regulator of G-protein signaling 9	RGS9	1,06
collagen, type IX, alpha 2	COL9A2	1,06
defensin, alpha 6, Paneth cell-specific	DEFA6	1,06
keratin associated protein 20-3	KRTAP20-3	1,06
olfactory receptor, family 5, subfamily D, member 18	OR5D18	1,06
ring finger protein 165	RNF165	1,06
zinc finger protein 197	ZNF197	1,06
cholinergic receptor, muscarinic 2	CHRM2	1,06
family with sequence similarity 166, member B	FAM166B	1,06
MORC family CW-type zinc finger 3	MORC3	1,06

leucine rich repeat containing 26	LRRC26	1,06
nitric oxide synthase 1 (neuronal) adaptor protein	NOS1AP	1,06
sorting nexin 18	SNX18	1,06
coiled-coil and C2 domain containing 2A	CC2D2A	1,06
TP73 antisense RNA 1	TP73-AS1	1,06
mitogen-activated protein kinase 10	MAPK10	1,06
olfactory receptor, family 10, subfamily A, member 6 (gene/pseudogene)	OR10A6	1,06
olfactory receptor family 10 subfamily A member 6 (gene/pseudogene) [Source:HGNC Symbol;Acc:HGNC:15132]	OR10A6	1,06
acidic repeat containing	ACRC	1,06
KN motif and ankyrin repeat domains 3	KANK3	1,06
ssu-2 homolog (C. elegans)	SSUH2	1,06
Memczak2013 ALT_ACCEPTOR, ALT_DONOR, coding, INTERNAL, intronic best transcript NM_001190457	CORO2B	1,06
spectrin repeat containing, nuclear envelope 2	SYNE2	1,06
oculocutaneous albinism II	OCA2	1,06
hedgehog acyltransferase	HHAT	1,06
cerebellin 2 precursor	CBLN2	1,06
oxidative stress responsive 1	OXSR1	1,06
family with sequence similarity 212, member B	FAM212B	1,06
cholinergic receptor, muscarinic 5	CHRM5	1,06
zinc finger protein 462	ZNF462	1,06
BEN domain containing 2	BEND2	1,06
LIM domain only 3 (rhombotin-like 2)	LMO3	1,06
family with sequence similarity 73, member B	FAM73B	1,06
mesoderm development candidate 1	MESDC1	1,06
PRAME family member 6 [Source:HGNC Symbol;Acc:HGNC:30583]	PRAMEF6	1,06
lens intrinsic membrane protein 2	LIM2	1,06
interleukin 12 receptor, beta 2	IL12RB2	1,06
homeobox B7	HOXB7	1,06
CREB regulated transcription coactivator 1	CRTC1	1,06
potassium channel, two pore domain subfamily K, member 7	KCNK7	1,06
enhancer of polycomb homolog 2 (Drosophila)	EPC2	1,06
coiled-coil domain containing 110	CCDC110	1,06
neuropeptide FF receptor 2	NPFFR2	1,06
downstream neighbor of SON; crystallin zeta like 1	DONSON; CRYZL1	1,06
testis expressed 29	TEX29	1,06
keratin 32, type I	KRT32	1,06
NIM1 serine/threonine protein kinase	NIM1K	1,06
opioid receptor, delta 1	OPRD1	1,06

eukaryotic translation initiation factor 4E family member 3	EIF4E3	1,06
cytokine receptor-like factor 2	CRLF2	1,06
coronin, actin binding protein, 1A	CORO1A	1,06
opsin 4	OPN4	1,06
NSE2/MMS21 homolog, SMC5-SMC6 complex SUMO ligase	NSMCE2	1,06
melanoma associated antigen (mutated) 1	MUM1	1,06
Synthetic construct Homo sapiens clone IMAGE:100069019, MGC:199030 methyl-CpG binding domain protein 3-like 2 (MBD3L2) mRNA, encodes complete protein.; novel transcript	MBD3L2; CTB-25J19.1	1,06
eukaryotic translation initiation factor 3, subunit M	EIF3M	1,06
glycoprotein Ib (platelet), beta polypeptide; septin 5	GP1BB; SEPT5	1,06
Transcript Identified by AceView, Entrez Gene ID(s) 26147	PHF19	1,06
chromosome 8 open reading frame 58	C8orf58	1,06
Homo sapiens olfactory receptor, family 4, subfamily M, member 2 (OR4M2), mRNA.; Homo sapiens olfactory receptor, family 4, subfamily M, member 2, mRNA (cDNA clone MGC:168600 IMAGE:9020977), complete cds.	OR4M2; AC171558.2	1,06
nei-like DNA glycosylase 2	NEIL2	1,06
olfactory receptor, family 5, subfamily C, member 1	OR5C1	1,06
Transcript Identified by AceView, Entrez Gene ID(s) 116840	CNTROB	1,06
family with sequence similarity 207, member A	FAM207A	1,06
RasGEF domain family member 1C	RASGEF1C	1,06
Eukaryotic translation initiation factor 3 subunit F [Source:UniProtKB/Swiss-Prot;Acc:O00303]	EIF3F	1,06
transmembrane protein 2	TMEM2	1,06
NK2 homeobox 5	NKX2-5	1,06
solute carrier family 34 (type II sodium/phosphate cotransporter), member 2	SLC34A2	1,06
multiple EGF-like-domains 11	MEGF11	1,06
WD repeat domain 60	WDR60	1,06
neurexin 3	NRXN3	1,06
arachidonate 5-lipoxygenase	ALOX5	1,06
microtubule associated serine/threonine kinase 1	MAST1	1,06
endothelin 2	EDN2	1,06
long intergenic non-protein coding RNA 336	LINC00336	1,06
EPH receptor A10	EPHA10	1,06
Memczak2013 ANTISENSE, CDS, coding, INTERNAL best transcript NM_033054	MYO1G	1,06

splA/ryanodine receptor domain and SOCS box containing 3	SPSB3	1,06
adhesion G protein-coupled receptor V1	ADGRV1	1,06
methyltransferase like 24	METTL24	1,06
SPATA31 subfamily C, member 2	SPATA31C2	1,06
uncharacterized LOC100129924; Transcript Identified by AceView, Entrez Gene ID(s) 79078; novel transcript	LOC100129924; C1orf50; RP5-994D16.11	1,06
membrane-spanning 4-domains, subfamily A, member 4E	MS4A4E	1,06
cilia and flagella associated protein 53	CFAP53	1,06
WD repeat domain 38	WDR38	1,06
adenosine A1 receptor	ADORA1	1,06
chymotrypsin-like elastase family, member 2A	CELA2A	1,06
uncharacterized serine/threonine-protein kinase SgK494; Synthetic construct Homo sapiens clone IMAGE:100064171, MGC:193254 uncharacterized serine/threonine-protein kinase SgK494 (FLJ25006) mRNA, encodes complete protein.; Salzman2013 ANNOTATED, CDS, coding, INTERNAL, OVCODE, OVERLAPTX, OVEXON best transcript NM_001174103; Transcript Identified by AceView, Entrez Gene ID(s) 124923; 9703; 645851; 10615; uncharacterized serine/threonine-protein kinase SgK494 [Source:EntrezGene;Acc:124923]; Uncharacterized protein [Source:UniProtKB/TrEMBL;Acc:J3KTE0]	SGK494; FLJ25006; KIAA0100andSGK494andSPAG5; RP11-192H23.4	1,06
Homo sapiens uncharacterized LOC388692 (LOC388692), long non-coding RNA.; Homo sapiens family with sequence similarity 231, member D (FAM231D), long non-coding RNA.; novel transcript	LOC388692; FAM231D; RP11-403I13.7; RP11-403I13.8	1,06
lipopolysaccharide binding protein	LBP	1,06
ubiquitin conjugating enzyme E2D 2	UBE2D2	1,06
collagen, type XXIII, alpha 1	COL23A1	1,06
kinesin family member 7	KIF7	1,06
unc-80 homolog (C. elegans)	UNC80	1,06
DEAD (Asp-Glu-Ala-Asp) box helicase 25	DDX25	1,06
cystatin-like 1	CSTL1	1,06
protease, serine, 37	PRSS37	1,06
FERM domain containing 1	FRMD1	1,06
leucine rich repeat containing 10B	LRRC10B	1,06
zinc finger protein, FOG family member 2	ZFPM2	1,06
trichohyalin like 1	TCHHL1	1,06
ankyrin repeat and death domain containing 1B	ANKDD1B	1,06
protein phosphatase 4, catalytic subunit	PPP4C	1,06
Fc receptor-like 3	FCRL3	1,06
DEAD (Asp-Glu-Ala-Asp) box polypeptide 53	DDX53	1,06

olfactory receptor, family 5, subfamily H, member 15; olfactory receptor, family 5, subfamily H, member 5 pseudogene	OR5H15; OR5H5P	1,06
forkhead box R2	FOXR2	1,06
hyaluronan synthase 2	HAS2	1,06
olfactory receptor, family 5, subfamily AR, member 1 (gene/pseudogene)	OR5AR1	1,06
3-oxoacid CoA-transferase 2 pseudogene 1	OXCT2P1	1,06
septin 3	sept-03	1,06
olfactory receptor, family 2, subfamily AG, member 2	OR2AG2	1,06
nucleoredoxin	NXN	1,06
neuropeptide B	NPB	1,06
transcription factor AP-2 gamma (activating enhancer binding protein 2 gamma)	TFAP2C	1,06
tetratricopeptide repeat domain 36	TTC36	1,06
G protein-coupled receptor 82	GPR82	1,06
LysM, putative peptidoglycan-binding, domain containing 2	LYSMD2	1,06
transmembrane and immunoglobulin domain containing 1	TMIGD1	1,06
TATA box binding protein associated factor 7 like	TAF7L	1,06
tubulin polyglutamylase complex subunit 1	TPGS1	1,06
EPH receptor B1	EPHB1	1,06
polymerase (RNA) III (DNA directed) polypeptide K, 12.3 kDa	POLR3K	1,06
Transcript Identified by AceView, Entrez Gene ID(s) 51105	PHF20L1	1,06
Jeck2013 ALT_ACCEPTOR, ALT_DONOR, coding, INTERNAL, intronic best transcript NM_001014797	KCNMA1	1,06
beta-1,3-glucuronyltransferase 2	B3GAT2	1,06
acid sensing ion channel 2	ASIC2	1,06
hyaluronan and proteoglycan link protein 2	HAPLN2	1,06
Memczak2013 ANTISENSE, CDS, coding, INTERNAL, UTR5 best transcript NM_001199573	TRIM22	1,05
P antigen family, member 2B	PAGE2B	1,05
POU class 5 homeobox 1B	POU5F1B	1,05
TBC1 domain family, member 3C	TBC1D3C	1,05
ankyrin repeat and death domain containing 1A	ANKDD1A	1,05
Jeck2013 ALT_ACCEPTOR, ALT_DONOR, coding, INTERNAL, intronic best transcript NM_015589	SAMD4A	1,05
dynein, axonemal, intermediate chain 2	DNAI2	1,05
Transcript Identified by AceView, Entrez Gene ID(s) 64405	CDH22	1,05
PPARGC1 and ESRR induced regulator, muscle 1	PERM1	1,05
ADP-ribosylation factor like GTPase 14 effector protein	ARL14EP	1,05

SPANX family, member N2	SPANXN2	1,05
family with sequence similarity 104, member A	FAM104A	1,05
family with sequence similarity 57, member B	FAM57B	1,05
transmembrane protein 183A	TMEM183A	1,05
vitrin	VIT	1,05
H6 family homeobox 1	HMX1	1,05
RAB25, member RAS oncogene family	RAB25	1,05
interleukin 17B	IL17B	1,05
trichoplein, keratin filament binding	TCHP	1,05
olfactory receptor, family 5, subfamily M, member 11	OR5M11	1,05
MOB family member 4, phocein	MOB4	1,05
Zhang2013 ALT_DONOR, coding, INTERNAL, intronic, OVERLAPTX, OVEXON best transcript NM_016648	LARP7	1,05
rhophilin associated tail protein 1-like	ROPN1L	1,05
small ArfGAP 1	SMAP1	1,05
synaptonemal complex protein 1	SYCP1	1,05
erythrocyte membrane protein band 4.2	EPB42	1,05
mediator complex subunit 21	MED21	1,05
cyclin and CBS domain divalent metal cation transport mediator 4	CNNM4	1,05
TP53RK binding protein	TPRKB	1,05
hyaluronan synthase 3	HAS3	1,05
roundabout guidance receptor 3	ROBO3	1,05
CUE domain containing 2	CUEDC2	1,05
zinc finger, DHHC-type containing 8	ZDHHC8	1,05
zinc finger, MYND-type containing 11	ZMYND11	1,05
cyclin G2	CCNG2	1,05
coiled-coil domain containing 62	CCDC62	1,05
surfeit 6	SURF6	1,05
PRAME family member 18	PRAMEF18	1,05
zinc finger protein 329	ZNF329	1,05
signal recognition particle receptor (docking protein)	SRPR	1,05
coiled-coil domain containing 120	CCDC120	1,05
chemerin chemokine-like receptor 1	CMKLR1	1,05
T-box 20	TBX20	1,05
NADH dehydrogenase (ubiquinone) complex I, assembly factor 6	NDUFAF6	1,05
major facilitator superfamily domain containing 12	MFSD12	1,05
calpain 10	CAPN10	1,05
CDC42 effector protein (Rho GTPase binding) 5	CDC42EP5	1,05
HKR1, GLI-Kruppel zinc finger family member	HKR1	1,05
arginine/serine-rich coiled-coil 2	RSRC2	1,05
zinc finger protein 98	ZNF98	1,05
retinol binding protein 3, interstitial	RBP3	1,05
chromosome 17 open reading frame 77	C17orf77	1,05

chromosome 7 open reading frame 33	C7orf33	1,05
POU class 3 homeobox 1	POU3F1	1,05
chromosome 15 open reading frame 39	C15orf39	1,05
proteasome subunit alpha 7	PSMA7	1,05
Rhox homeobox family, member 1	RHOXF1	1,05
coiled-coil domain containing 88C	CCDC88C	1,05
ribosomal protein L28; microRNA 6805	RPL28; MIR6805	1,05
glutamate receptor, ionotropic, delta 2	GRID2	1,05
olfactory receptor, family 14, subfamily C, member 36	OR14C36	1,05
interferon regulatory factor 7	IRF7	1,05
C-type lectin domain family 2, member L	CLEC2L	1,05
zinc finger protein 674	ZNF674	1,05
cytochrome c oxidase assembly factor 6	COA6	1,05
putative deoxyuridine 5-triphosphate nucleotidohydrolase-like protein FLJ16323; novel transcript; Transcript Identified by AceView	LOC100506422; RP11-80I3.1; tayamu	1,05
myelin transcription factor 1-like	MYT1L	1,05
potassium channel, inwardly rectifying subfamily J, member 1	KCNJ1	1,05
peroxisomal biogenesis factor 2	PEX2	1,05
general transcription factor IIIC subunit 4	GTF3C4	1,05
T-cell immunoglobulin and mucin domain containing 4	TIMD4	1,05
ubiquitin conjugating enzyme E2G 1	UBE2G1	1,05
DnaJ (Hsp40) homolog, subfamily B, member 7	DNAJB7	1,05
zinc finger protein 17	ZNF17	1,05
potassium channel, subfamily U, member 1	KCNU1	1,05
cartilage intermediate layer protein, nucleotide pyrophosphohydrolase	CILP	1,05
glypican 5	GPC5	1,05
zinc finger protein 268	ZNF268	1,05
cysteine-rich PDZ-binding protein	CRIPT	1,05
KIAA0930	KIAA0930	1,05
ring finger protein 7	RNF7	1,05
Src homology 2 domain containing transforming protein D	SHD	1,05
replication protein A4	RPA4	1,05
olfactory receptor, family 51, subfamily L, member 1	OR51L1	1,05
fibronectin leucine rich transmembrane protein 1	FLRT1	1,05
prokineticin 2	PROK2	1,05
olfactory receptor, family 7, subfamily G, member 3	OR7G3	1,05
Transcript Identified by AceView, Entrez Gene ID(s) 287	ANK2	1,05
ADAMTSL4 antisense RNA 1	ADAMTSL4-AS1	1,05
interferon, gamma	IFNG	1,05
interferon, alpha 8	IFNA8	1,05
patatin-like phospholipase domain containing 5	PNPLA5	1,05

uncharacterized LOC400661; novel transcript	LOC400661; AC034110.1	1,05
myocilin, trabecular meshwork inducible glucocorticoid response	MYOC	1,05
doublecortin domain containing 1	DCDC1	1,05
interleukin 36 receptor antagonist	IL36RN	1,05
F-box and leucine-rich repeat protein 3	FBXL3	1,05
zinc finger protein 311	ZNF311	1,05
P antigen family, member 5 (prostate associated)	PAGE5	1,05
enolase-phosphatase 1	ENOPH1	1,05
protein associated with topoisomerase II homolog 2 (yeast)	PATL2	1,05
TBC1 domain family, member 3	TBC1D3	1,05
coatomer protein complex subunit zeta 1	COPZ1	1,05
Rho-associated, coiled-coil containing protein kinase 1	ROCK1	1,05
family with sequence similarity 19 (chemokine (C-C motif)-like), member A3	FAM19A3	1,05
bladder cancer associated protein	BLCAP	1,05
solute carrier family 30 (zinc transporter), member 4	SLC30A4	1,05
chromosome 1 open reading frame 35	C1orf35	1,05
adaptor-related protein complex 2, alpha 2 subunit	AP2A2	1,05
RAS guanyl releasing protein 2 (calcium and DAG-regulated)	RASGRP2	1,05
oligosaccharyltransferase complex subunit 4 (non-catalytic)	OST4	1,05
leukocyte immunoglobulin-like receptor, subfamily B (with TM and ITIM domains), member 2	LILRB2	1,05
KIAA1211	KIAA1211	1,05
dopamine receptor D2	DRD2	1,05
polymerase (RNA) II (DNA directed) polypeptide B, 140kDa	POLR2B	1,05
Zhang2013 ALT_ACCEPTOR, ALT_DONOR, coding, INTERNAL, intronic, OVERLAPTX best transcript NM_017550	MIER2	1,05
nuclear protein 2, transcriptional regulator	NUPR2	1,05
EYA transcriptional coactivator and phosphatase 1	EYA1	1,05
ATH1, acid trehalase-like 1 (yeast)	ATHL1	1,05
cytochrome P450, family 2, subfamily C, polypeptide 18	CYP2C18	1,05
KH and NYN domain containing	KHNYN	1,05
FtsJ RNA methyltransferase homolog 2 (E. coli)	FTSJ2	1,05
Salzman2013 ALT_ACCEPTOR, ALT_DONOR, coding, INTERNAL, intronic best transcript NM_012215	MGEA5	1,05
outer dense fiber of sperm tails 3-like 2	ODF3L2	1,05
androgen-induced 1	AIG1	1,05
zinc finger protein 649	ZNF649	1,05

general transcription factor IIH subunit 2	GTF2H2	1,05
	LRIG1	1,05
cell division cycle 27	CDC27	1,05
HEN1 methyltransferase homolog 1 (Arabidopsis)	HENMT1	1,05
solute carrier family 17 (vesicular glutamate transporter), member 6	SLC17A6	1,05
ARL2-SNX15 readthrough (NMD candidate)	ARL2-SNX15	1,05
ankyrin repeat domain 6	ANKRD6	1,05
zinc finger protein 502	ZNF502	1,05
chromosome 10 open reading frame 71	C10orf71	1,05
DEAH (Asp-Glu-Ala-His) box polypeptide 16	DHX16	1,05
zinc finger protein 285	ZNF285	1,05
A kinase (PRKA) anchor protein 3	AKAP3	1,05
granulysin	GNLY	1,05
guanine nucleotide binding protein (G protein), alpha inhibiting activity polypeptide 3	GNAI3	1,05
oculocerebrorenal syndrome of Lowe	OCRL	1,05
chromosome 4 open reading frame 48	C4orf48	1,05
alanyl-tRNA synthetase	AARS	1,05
TAF9B RNA polymerase II, TATA box binding protein (TBP)-associated factor, 31kDa	TAF9B	1,05
CD53 molecule	CD53	1,05
ELMO/CED-12 domain containing 1	ELMOD1	1,05
uncharacterized LOC102467081; novel transcript, antisense to GZMK; Transcript Identified by AceView	LOC102467081; CTD-2313F11.1; slygee	1,05
mitogen-activated protein kinase 4	MAPK4	1,05
G protein-coupled receptor associated sorting protein 2	GPRASP2	1,05
hephaestin	HEPH	1,05
chromosome 2 open reading frame 73	C2orf73	1,05
coiled-coil domain containing 3	CCDC3	1,05
family with sequence similarity 105, member A	FAM105A	1,05
coiled-coil domain containing 168	CCDC168	1,05
stereocilin	STRC	1,05
T-cell leukemia homeobox 3	TLX3	1,05
NME/NM23 family member 9	NME9	1,05
Scm-like with four mbt domains 2	SFMBT2	1,05
mitochondrial ribosomal protein S25	MRPS25	1,05
protein kinase C, epsilon	PRKCE	1,05
Transcript Identified by AceView, Entrez Gene ID(s) 219333	USP12	1,05
natural cytotoxicity triggering receptor 2	NCR2	1,05
transforming growth factor beta regulator 1	TBRG1	1,05
tetratricopeptide repeat domain 31	TTC31	1,05
zinc finger, DHHC-type containing 13	ZDHHC13	1,05

neurotrophin 3	NTF3	1,05
acyl-CoA thioesterase 4	ACOT4	1,05
Transcript Identified by AceView, Entrez Gene ID(s) 51056	LAP3	1,05
galactosidase beta 1 like 3	GLB1L3	1,05
SLIT and NTRK-like family, member 2	SLITRK2	1,05
long intergenic non-protein coding RNA 471	LINC00471	1,05
ST6 beta-galactosamide alpha-2,6-sialyltranferase 2	ST6GAL2	1,05
protocadherin beta 11	PCDHB11	1,05
olfactory receptor, family 4, subfamily M, member 1	OR4M1	1,05
fem-1 homolog c (C. elegans)	FEM1C	1,05
pleiomorphic adenoma gene-like 1; hydatidiform mole associated and imprinted (non-protein coding)	PLAGL1; HYMAI	1,05
synapsin II	SYN2	1,05
variable charge, X-linked 2	VCX2	1,05
phosphodiesterase 1C, calmodulin-dependent 70kDa	PDE1C	1,05
claudin 7	CLDN7	1,05
myosin, light chain 3, alkali; ventricular, skeletal, slow	MYL3	1,05
vaccinia related kinase 1	VRK1	1,05
lipid droplet associated hydrolase	LDAH	1,05
ADP-ribosylation factor like GTPase 6	ARL6	1,05
solute carrier organic anion transporter family, member 2B1	SLCO2B1	1,05
olfactory receptor, family 5, subfamily M, member 8	OR5M8	1,05
phosphatidylinositol-4,5-bisphosphate 3-kinase, catalytic subunit beta	PIK3CB	1,05
Memczak2013 ALT_ACCEPTOR, ALT_DONOR, coding, INTERNAL, intronic best transcript NM_001076787	TP53I11	1,05
peptidylprolyl isomerase E (cyclophilin E)	PPIE	1,05
FAT atypical cadherin 2	FAT2	1,05
actin, beta	ACTB	1,05
cysteine and glycine-rich protein 1	CSRP1	1,05
KIAA1143 [Source:HGNC Symbol;Acc:HGNC:29198]	KIAA1143	1,05
chromodomain helicase DNA binding protein 3	CHD3	1,05
cholinergic receptor, nicotinic alpha 5	CHRNA5	1,05
olfactory receptor, family 52, subfamily B, member 6	OR52B6	1,05
calcyphosine	CAPS	1,05
P antigen family, member 2 (prostate associated)	PAGE2	1,05
testis-specific serine kinase 1B	TSSK1B	1,05
TBC1D3P1-DHX40P1 readthrough transcribed pseudogene	TBC1D3P1-DHX40P1	1,05
Rho guanine nucleotide exchange factor 15	ARHGEF15	1,05
uncharacterized LOC101928327; novel transcript	LOC101928327; AC007557.1	1,05
relaxin 2	RLN2	1,05

olfactory receptor, family 3, subfamily A, member 4 pseudogene; olfactory receptor, family 3, subfamily A, member 5 pseudogene	OR3A4P; OR3A5P	1,05
HEPACAM family member 2	HEPACAM2	1,05
zinc finger protein 705G	ZNF705G	1,05
tetratricopeptide repeat domain 29	TTC29	1,05
coiled-coil domain containing 85A	CCDC85A	1,05
distal-less homeobox 6	DLX6	1,05
tRNA methyltransferase 44 homolog (S. cerevisiae)	TRMT44	1,05
mitochondrial ribosomal protein L32	MRPL32	1,05
intraflagellar transport 43	IFT43	1,05
ADP-ribosylation factor like GTPase 5B	ARL5B	1,05
forkhead box D3	FOXD3	1,05
phosphodiesterase 6D, cGMP-specific, rod, delta	PDE6D	1,05
chromosome 12 open reading frame 65	C12orf65	1,05
adhesion G protein-coupled receptor F2	ADGRF2	1,05
myocyte enhancer factor 2A	MEF2A	1,05
colipase, pancreatic	CLPS	1,05
coiled-coil domain containing 83	CCDC83	1,05
pterin-4 alpha-carbinolamine dehydratase/dimerization cofactor of hepatocyte nuclear factor 1 alpha	PCBD1	1,05
calcium channel, voltage-dependent, L type, alpha 1S subunit	CACNA1S	1,05
ERBB receptor feedback inhibitor 1	ERRFI1	1,05
multiciliate differentiation and DNA synthesis associated cell cycle protein	MCIDAS	1,05
pleckstrin homology domain containing, family G (with RhoGef domain) member 1	PLEKHG1	1,05
tripartite motif containing 49D1; tripartite motif containing 49D2	TRIM49D1; TRIM49D2	1,05
keratin associated protein 5-6	KRTAP5-6	1,05
apolipoprotein B mRNA editing enzyme, catalytic polypeptide 1	APOBEC1	1,05
STARD3 N-terminal like	STARD3NL	1,05
NADH dehydrogenase (ubiquinone) 1 alpha subcomplex, 6, 14kDa	NDUFA6	1,05
charged multivesicular body protein 6	CHMP6	1,05
migration and invasion inhibitory protein	MIIP	1,05
MAS-related GPR, member D	MRGPRD	1,05
coiled-coil domain containing 188	CCDC188	1,05
protein kinase C and casein kinase substrate in neurons 2	PACSIN2	1,05
centriolin	CNTRL	1,05
sperm associated antigen 11A; sperm associated antigen 11B	SPAG11A; SPAG11B	1,05

zinc finger protein 202	ZNF202	1,05
ATM interactor	ATMIN	1,05
lipoma HMGIC fusion partner-like 1	LHFPL1	1,05
cyclin-dependent kinase 5	CDK5	1,05
SPATA31 subfamily E, member 1	SPATA31E1	1,05
olfactory receptor, family 6, subfamily S, member 1	OR6S1	1,05
trafficking protein particle complex 11	TRAPPC11	1,05
cytochrome c oxidase assembly homolog 15 (yeast)	COX15	1,05
protein phosphatase 2, regulatory subunit B, beta	PPP2R5B	1,05
zinc finger protein 668	ZNF668	1,05
CD300e molecule	CD300E	1,05
tumor suppressor candidate 5	TUSC5	1,05
neuronal differentiation 1	NEUROD1	1,05
aldehyde dehydrogenase 1 family, member A2	ALDH1A2	1,05
phosphodiesterase 8B	PDE8B	1,05
cytochrome P450, family 4, subfamily B, polypeptide 1	CYP4B1	1,05
cylicin, basic protein of sperm head cytoskeleton 1	CYLC1	1,05
DEAD/H (Asp-Glu-Ala-Asp/His) box polypeptide 26B	DDX26B	1,05
autophagy related 16-like 1	ATG16L1	1,05
wingless-type MMTV integration site family, member 16	WNT16	1,05
biogenesis of lysosomal organelles complex-1, subunit 6, pallidin	BLOC1S6	1,05
ubiquitin-conjugating enzyme E2F (putative)	UBE2F	1,05
protein tyrosine phosphatase, non-receptor type 9	PTPN9	1,05
angiopoietin 4	ANGPT4	1,05
H6 family homeobox 3	HMX3	1,05
ring finger protein, transmembrane 1	RNFT1	1,05
olfactory receptor, family 4, subfamily D, member 5	OR4D5	1,05
chromosome 2 open reading frame 40	C2orf40	1,05
solute carrier family 38, member 8	SLC38A8	1,05
tubulin polymerization promoting protein	TPPP	1,05
myosin regulatory light chain interacting protein; microRNA 4639	MYLIP; MIR4639	1,05
guanine nucleotide binding protein (G protein), alpha transducing activity polypeptide 1	GNAT1	1,05
Homo sapiens golgin A6 family-like 4 (GOLGA6L4), mRNA.; golgin A6 family-like 4; golgin A6 family-like 4 [Source:HGNC Symbol;Acc:HGNC:27256]	GOLGA6L4	1,05
Down syndrome cell adhesion molecule	DSCAM	1,05
ATP binding cassette subfamily G member 1	ABCG1	1,05
homeobox B5	HOXB5	1,05
reticulocalbin 1, EF-hand calcium binding domain	RCN1	1,05
adenomatosis polyposis coli 2	APC2	1,05
placenta specific 9	PLAC9	1,05

sialic acid binding Ig-like lectin 16 (gene/pseudogene)	SIGLEC16	1,05
chromosome 16 open reading frame 71	C16orf71	1,05
retinol binding protein 7, cellular	RBP7	1,05
PPAN-P2RY11 readthrough; purinergic receptor P2Y, G-protein coupled, 11; peter pan homolog (Drosophila)	PPAN-P2RY11; P2RY11; PPAN	1,05
immunoglobulin mu binding protein 2	IGHMBP2	1,05
creatine kinase, muscle	CKM	1,05
neuropeptide W	NPW	1,05
aldo-keto reductase family 1, member C8, pseudogene	AKR1C8P	1,05
Memczak2013 ALT_ACCEPTOR, ALT_DONOR, coding, INTERNAL, intronic best transcript NM_022720	DGCR8	1,05
ribosomal protein S4, Y-linked 2	RPS4Y2	1,05
cytochrome c oxidase assembly factor 7 (putative)	COA7	1,05
translocase of inner mitochondrial membrane 23 homolog B (yeast)	TIMM23B	1,05
wingless-type MMTV integration site family, member 2B	WNT2B	1,05
prohibitin	PHB	1,05
La ribonucleoprotein domain family, member 4	LARP4	1,05
Rap guanine nucleotide exchange factor 3	RAPGEF3	1,05
olfactory receptor, family 8, subfamily J, member 1	OR8J1	1,04
chromosome 22 open reading frame 15	C22orf15	1,04
transmembrane protein 222	TMEM222	1,04
urocanate hydratase 1	UROC1	1,04
Memczak2013 ALT_ACCEPTOR, ALT_DONOR, coding, INTERNAL, intronic best transcript NM_001142393	NEDD9	1,04
jumonji, AT rich interactive domain 2	JARID2	1,04
Memczak2013 ALT_ACCEPTOR, ALT_DONOR, coding, INTERNAL, intronic best transcript NM_000655	SELL	1,04
Olfactory receptor 4N4 [Source:UniProtKB/Swiss-Prot;Acc:Q8N0Y3]	AC171558.1	1,04
defensin, beta 119	DEFB119	1,04
signal transducer and activator of transcription 5A	STAT5A	1,04
platelet-activating factor acetylhydrolase 1b, catalytic subunit 3 (29kDa)	PAFAH1B3	1,04
chromosome 1 open reading frame 64	C1orf64	1,04
DENN/MADD domain containing 4B	DENND4B	1,04
armadillo repeat containing 4	ARMC4	1,04
ring finger protein 168, E3 ubiquitin protein ligase	RNF168	1,04
hedgehog acyltransferase	HHAT	1,04
N-sulfoglucosamine sulfohydrolase	SGSH	1,04
ZNF252P antisense RNA 1	ZNF252P-AS1	1,04
adaptor-related protein complex 1, gamma 2 subunit	AP1G2	1,04
lysophosphatidic acid receptor 1	LPAR1	1,04
NLR family, pyrin domain containing 12	NLRP12	1,04

nuclear receptor subfamily 0, group B, member 1	NR0B1	1,04
centrosomal protein 72kDa	CEP72	1,04
olfactory receptor, family 5, subfamily W, member 2	OR5W2	1,04
gastrin-releasing peptide	GRP	1,04
chromosome 6 open reading frame 15	C6orf15	1,04
APITD1-CORT readthrough; cortistatin; apoptosis-inducing, TAF9-like domain 1	APITD1-CORT; CORT; APITD1	1,04
chromosome 16 open reading frame 86	C16orf86	1,04
thymidine phosphorylase; SCO2 cytochrome c oxidase assembly protein	TYMP; SCO2	1,04
chromosome 7 open reading frame 66	C7orf66	1,04
potassium channel, two pore domain subfamily K, member 18	KCNK18	1,04
Transcript Identified by AceView, Entrez Gene ID(s) 50515	CHST11	1,04
transglutaminase 4	TGM4	1,04
SPANX family, member N3	SPANXN3	1,04
CSAG family, member 3	CSAG3	1,04
endothelin converting enzyme 2	ECE2	1,04
Transcript Identified by AceView, Entrez Gene ID(s) 51074	APIP	1,04
defensin, beta 103A; defensin, beta 103B	DEFB103A; DEFB103B	1,04
fermitin family member 3	FERMT3	1,04
Transcript Identified by AceView, Entrez Gene ID(s) 131034	CPNE4	1,04
guanine nucleotide binding protein (G protein), gamma 10	GNG10	1,04
fer-1-like family member 5	FER1L5	1,04
protein phosphatase 2, regulatory subunit B, gamma	PPP2R2C	1,04
EF-hand calcium binding domain 8	EFCAB8	1,04
cleavage and polyadenylation specific factor 3-like; microRNA 6727	CPSF3L; MIR6727	1,04
RAB4B, member RAS oncogene family; MIA-RAB4B readthrough (NMD candidate); RAB4B-EGLN2 readthrough (NMD candidate)	RAB4B; MIA-RAB4B; RAB4B-EGLN2	1,04
olfactory receptor, family 1, subfamily A, member 1	OR1A1	1,04
AT rich interactive domain 3C (BRIGHT-like)	ARID3C	1,04
CASK interacting protein 2	CASKIN2	1,04
adenylate cyclase 9	ADCY9	1,04
glycine-N-acyltransferase	GLYAT	1,04
aspartate dehydrogenase domain containing	ASPDH	1,04
ankyrin repeat domain 60	ANKRD60	1,04
cytoplasmic linker associated protein 2	CLASP2	1,04
clathrin, heavy chain (Hc)	CLTC	1,04

olfactory receptor, family 2, subfamily V, member 2	OR2V2	1,04
ceramide kinase like	CERKL	1,04
purinergic receptor P2X, ligand gated ion channel, 1	P2RX1	1,04
myoneurin	MYNN	1,04
coiled-coil domain containing 185	CCDC185	1,04
serine/arginine repetitive matrix 5	SRRM5	1,04
HPN antisense RNA 1	HPN-AS1	1,04
family with sequence similarity 25, member G; family with sequence similarity 25, member C; protein FAM25	FAM25G; FAM25C; FAM25BP	1,04
FRMPD3 antisense RNA 1	FRMPD3-AS1	1,04
slit guidance ligand 1; ARHGAP19-SLIT1 readthrough (NMD candidate)	SLIT1; ARHGAP19-SLIT1	1,04
ADP-ribosylation factor like GTPase 13B	ARL13B	1,04
ankyrin repeat domain 34C	ANKRD34C	1,04
Memczak2013 ANTISENSE, coding, INTERNAL, intronic best transcript NM_080760	DACH1	1,04
crystallin mu	CRYM	1,04
phosphodiesterase 4D interacting protein	PDE4DIP	1,04
SMG5 nonsense mediated mRNA decay factor	SMG5	1,04
integrin beta 7	ITGB7	1,04
neuropeptide Y receptor Y2	NPY2R	1,04
extracellular leucine-rich repeat and fibronectin type III domain containing 2	ELFN2	1,04
otogelin-like	OTOGL	1,04
piwi-like RNA-mediated gene silencing 1	PIWIL1	1,04
hedgehog acyltransferase	HHAT	1,04
CaM kinase-like vesicle-associated	CAMKV	1,04
lysine (K)-specific methyltransferase 5B	KMT5B	1,04
transforming growth factor beta 2; TGFB2 overlapping transcript 1	TGFB2; TGFB2-OT1	1,04
oxysterol binding protein-like 5	OSBPL5	1,04
myotubularin related protein 8	MTMR8	1,04
cyclin B3	CCNB3	1,04
kelch-like family member 29	KLHL29	1,04
ankyrin repeat and SOCS box containing 5	ASB5	1,04
intraflagellar transport 122	IFT122	1,04
protein arginine methyltransferase 5	PRMT5	1,04
solute carrier family 10, member 4	SLC10A4	1,04
N-ethylmaleimide-sensitive factor attachment protein, beta	NAPB	1,04
integrin alpha FG-GAP repeat containing 2	ITFG2	1,04
Rho GTPase activating protein 28	ARHGAP28	1,04
kallikrein related peptidase 7	KLK7	1,04
glutamate receptor, ionotropic, AMPA 3	GRIA3	1,04

retinol dehydrogenase 8 (all-trans)	RDH8	1,04
glycine receptor alpha 1	GLRA1	1,04
vacuolar protein sorting 37 homolog C (S. cerevisiae)	VPS37C	1,04
chromosome 17 open reading frame 74	C17orf74	1,04
v-myb avian myeloblastosis viral oncogene homolog-like 2	MYBL2	1,04
ATPase type 13A5	ATP13A5	1,04
transcription factor 21	TCF21	1,04
BVES antisense RNA 1	BVES-AS1	1,04
WAP four-disulfide core domain 10A	WFDC10A	1,04
chromosome X open reading frame 36	CXorf36	1,04
corin, serine peptidase	CORIN	1,04
human immunodeficiency virus type I enhancer binding protein 1	HIVEP1	1,04
tripartite motif containing 64B	TRIM64B	1,04
calcium responsive transcription factor	CARF	1,04
zinc finger protein 669	ZNF669	1,04
X antigen family, member 1E; X antigen family, member 1B	XAGE1E; XAGE1B	1,04
solute carrier family 35 (CMP-sialic acid transporter), member A1	SLC35A1	1,04
dephospho-CoA kinase domain containing	DCAKD	1,04
Transcript Identified by AceView, Entrez Gene ID(s) 4179	CD46	1,04
interferon-induced protein with tetratricopeptide repeats 2	IFIT2	1,04
discs, large homolog 1 (Drosophila)	DLG1	1,04
interleukin 1 receptor associated kinase 4	IRAK4	1,04
exosome component 2	EXOSC2	1,04
hydroxymethylbilane synthase	HMBS	1,04
synaptotagmin X	SYT10	1,04
LUC7-like 3 pre-mRNA splicing factor	LUC7L3	1,04
CDC42 effector protein (Rho GTPase binding) 3	CDC42EP3	1,04
Jeck2013 ANTISENSE, coding, INTERNAL, OVEXON, UTR3 best transcript NM_020746	MAVS	1,04
complement factor H-related 5	CFHR5	1,04
solute carrier family 25, member 39	SLC25A39	1,04
receptor accessory protein 1	REEP1	1,04
potassium channel tetramerization domain containing 4	KCTD4	1,04
olfactory receptor, family 10, subfamily A, member 3	OR10A3	1,04
NACHT and WD repeat domain containing 2	NWD2	1,04
solute carrier family 12 (sodium/chloride transporter), member 3	SLC12A3	1,04
PRAME family member 25	PRAMEF25	1,04
meningioma (disrupted in balanced translocation) 1	MN1	1,04

GTPase, IMAP family member 7	GIMAP7	1,04
succinate dehydrogenase complex subunit B, iron sulfur (Ip)	SDHB	1,04
structural maintenance of chromosomes 1B	SMC1B	1,04
protocadherin 12	PCDH12	1,04
chromosome 3 open reading frame 30	C3orf30	1,04
CTAGE family, member 6	CTAGE6	1,04
heparan sulfate (glucosamine) 3-O-sulfotransferase 6	HS3ST6	1,04
superoxide dismutase 3, extracellular	SOD3	1,04
synaptosome associated protein 91kDa	SNAP91	1,04
Transcript Identified by AceView, Entrez Gene ID(s) 23126	POGZ	1,04
dipeptidase 2	DPEP2	1,04
methyltransferase like 11B	METTL11B	1,04
coiled-coil serine rich protein 1	CCSER1	1,04
PR domain containing 12	PRDM12	1,04
chromosome 11 open reading frame 21	C11orf21	1,04
radial spoke head 9 homolog (Chlamydomonas)	RSPH9	1,04
WD repeat domain 24	WDR24	1,04
lymphocyte antigen 6 complex, locus G6C	LY6G6C	1,04
espin-like	ESPNL	1,04
keratin 5, type II	KRT5	1,04
DnaJ (Hsp40) homolog, subfamily C, member 5; microRNA 941-1; microRNA 941-2; microRNA 941-3; microRNA 941-4; microRNA 941-5	DNAJC5; MIR941-1; MIR941-2; MIR941-3; MIR941-4; MIR941-5	1,04
solute carrier family 2 (facilitated glucose transporter), member 12	SLC2A12	1,04
SPT2 chromatin protein domain containing 1	SPTY2D1	1,04
ADP-ribosylation factor like GTPase 9	ARL9	1,04
uncharacterized LOC79160; novel transcript	LOC79160; RP5-907D15.4	1,04
Niemann-Pick disease, type C2; microRNA 4709	NPC2; MIR4709	1,04
hyaluronan and proteoglycan link protein 3	HAPLN3	1,04
arginine vasopressin	AVP	1,04
family with sequence similarity 170, member B	FAM170B	1,04
Transcript Identified by AceView, Entrez Gene ID(s) 10265; novel transcript	CTD-3032H12.2; IRX5	1,04
chromosome 17 open reading frame 64	C17orf64	1,04
defensin, beta 107A; defensin, beta 107B	DEFB107A; DEFB107B	1,04
carnitine O-octanoyltransferase	CROT	1,04
parathyroid hormone 1 receptor	PTH1R	1,04
piccolo presynaptic cytomatrix protein	PCLO	1,04

minichromosome maintenance domain containing 2	MCMDC2	1,04
glycophorin A (MNS blood group)	GYPA	1,04
coiled-coil domain containing 129	CCDC129	1,04
zinc finger protein 480	ZNF480	1,04
translocase of inner mitochondrial membrane 21 homolog (yeast)	TIMM21	1,04
taste receptor, type 2, member 60	TAS2R60	1,04
complement component 1, q subcomponent-like 1	C1QL1	1,04
B-cell CLL/lymphoma 6, member B	BCL6B	1,04
protein kinase, cAMP-dependent, regulatory, type II, alpha	PRKAR2A	1,04
zinc finger protein 234	ZNF234	1,04
olfactory receptor, family 6, subfamily C, member 3	OR6C3	1,04
tubulin tyrosine ligase-like family member 1	TTLL1	1,04
leucine rich repeat containing 55	LRRC55	1,04
IQ motif containing F2	IQCF2	1,04
calcium homeostasis endoplasmic reticulum protein	CHERP	1,04
calcium channel, voltage-dependent, L type, alpha 1F subunit	CACNA1F	1,04
bactericidal/permeability-increasing protein	BPI	1,04
forkhead box L1	FOXL1	1,04
potassium channel, voltage gated subfamily E regulatory beta subunit 1	KCNE1	1,04
natural cytotoxicity triggering receptor 3	NCR3	1,04
G protein-coupled receptor, class C, group 5, member B	GPRC5B	1,04
heat shock protein, alpha-crystallin-related, B9	HSPB9	1,04
chromosome 19 open reading frame 25	C19orf25	1,04
forkhead box N4	FOXN4	1,04
IQ motif containing with AAA domain 1	IQCA1	1,04
olfactory receptor, family 5, subfamily K, member 4	OR5K4	1,04
interferon-induced protein 44	IFI44	1,04
coiled-coil domain containing 150	CCDC150	1,04
DnaJ (Hsp40) homolog, subfamily B, member 6	DNAJB6	1,04
secretory carrier membrane protein 3	SCAMP3	1,04
F-box and leucine-rich repeat protein 7	FBXL7	1,04
zinc finger protein 57	ZNF57	1,04
methyltransferase like 5	METTL5	1,04
proline rich 12	PRR12	1,04
fukutin related protein	FKRP	1,04
coiled-coil domain containing 85B	CCDC85B	1,04
gamma-aminobutyric acid (GABA) A receptor, alpha 2	GABRA2	1,04
BTG family, member 2	BTG2	1,04
secretin receptor	SCTR	1,04
acid phosphatase, testicular	ACPT	1,04
DnaJ (Hsp40) homolog, subfamily C, member 17	DNAJC17	1,04

synovial sarcoma translocation, chromosome 18	SS18	1,04
ribosomal protein S6 kinase, 90kDa, polypeptide 1	RPS6KA1	1,04
membrane bound transcription factor peptidase, site 2; YY2 transcription factor	MBTPS2; YY2	1,04
HHIP-like 1	HHIPL1	1,04
protein prenyltransferase alpha subunit repeat containing 1	PTAR1	1,04
discs, large homolog 5 (Drosophila)	DLG5	1,04
SERTA domain containing 4	SERTAD4	1,04
chromosome 1 open reading frame 158	C1orf158	1,04
ring finger protein 220	RNF220	1,04
NRSN2 antisense RNA 1	NRSN2-AS1	1,04
acyl-CoA synthetase medium-chain family member 6	ACSM6	1,04
solute carrier family 18 (vesicular acetylcholine transporter), member 3	SLC18A3	1,04
chromosome 17 open reading frame 82	C17orf82	1,04
HIG1 hypoxia inducible domain family, member 1B	HIGD1B	1,04
histamine receptor H1	HRH1	1,04
hydroxysteroid (17-beta) dehydrogenase 6	HSD17B6	1,04
SEC14-like lipid binding 5	SEC14L5	1,04
zinc finger and SCAN domain containing 30	ZSCAN30	1,04
clavesin 1	CLVS1	1,04
novel transcript; Transcript Identified by AceView, Entrez Gene ID(s) 64116	RP11-499E18.1; SLC39A8	1,04
proline rich 23 domain containing 2	PRR23D2	1,04
glutathione peroxidase 3	GPX3	1,04
zinc finger protein 273	ZNF273	1,04
Transcript Identified by AceView, Entrez Gene ID(s) 64327	LMBR1	1,04
transmembrane protein 171	TMEM171	1,04
leucine rich repeat containing 3B	LRRC3B	1,04
chorionic gonadotropin, beta polypeptide 5; chorionic gonadotropin, beta polypeptide 8	CGB5; CGB8	1,04
ALG13, UDP-N-acetylglucosaminyltransferase subunit	ALG13	1,04
keratin 1, type II	KRT1	1,04
solute carrier family 2 (facilitated glucose transporter), member 7	SLC2A7	1,04
non-specific cytotoxic cell receptor protein 1 homolog (zebrafish)	NCCRP1	1,04
deleted in azoospermia-like	DAZL	1,04
dynein, axonemal, assembly factor 5	DNAAF5	1,04
5-hydroxytryptamine (serotonin) receptor 1B, G protein-coupled	HTR1B	1,04
Usher syndrome 1G (autosomal recessive)	USH1G	1,04
leucine rich repeat containing 15	LRRC15	1,04

zinc finger protein 19	ZNF19	1,04
reticulon 3	RTN3	1,04
chromosome 1 open reading frame 146	C1orf146	1,04
chorionic gonadotropin, beta polypeptide 2	CGB2	1,04
polymerase (RNA) I polypeptide A	POLR1A	1,04
cancer/testis antigen 2	CTAG2	1,04
BCL2-like 14 (apoptosis facilitator)	BCL2L14	1,04
purinergic receptor P2X, ligand gated ion channel, 7	P2RX7	1,04
tRNA-histidine guanylyltransferase 1-like	THG1L	1,04
deoxyribonuclease II beta	DNASE2B	1,04
prostate cancer susceptibility candidate 2	PRAC2	1,04
cathepsin S	CTSS	1,04
GA binding protein transcription factor alpha subunit	GABPA	1,04
zinc finger, DHHC-type containing 22	ZDHHC22	1,04
protein disulfide isomerase-like, testis expressed	PDILT	1,04
shisa family member 9	SHISA9	1,04
cAMP-regulated phosphoprotein 19kDa	ARPP19	1,04
negative regulator of ubiquitin-like proteins 1	NUB1	1,04
olfactory receptor, family 4, subfamily F, member 4; olfactory receptor, family 4, subfamily F, member 17	OR4F4; OR4F17	1,04
neuropeptide VF precursor	NPVF	1,04
tyrosyl-DNA phosphodiesterase 1	TDP1	1,04
zinc finger, DHHC-type containing 3	ZDHHC3	1,04
RNA guanylyltransferase and 5-phosphatase	RNGTT	1,04
cadherin, EGF LAG seven-pass G-type receptor 3; microRNA 4793	CELSR3; MIR4793	1,04
kelch-like family member 9	KLHL9	1,04
syntaphilin	SNPH	1,04
androgen-dependent TFPI-regulating protein	ADTRP	1,04
pyrroline-5-carboxylate reductase-like	PYCRL	1,04
Memczak2013 ANTISENSE, CDS, coding, INTERNAL best transcript NM_020175	DUS3L	1,04
chloride intracellular channel 2	CLIC2	1,04
solute carrier family 9, subfamily A (NHE3, cation proton antiporter 3), member 3	SLC9A3	1,04
CDC42 small effector 2	CDC42SE2	1,04
HSPE1-MOB4 readthrough	HSPE1-MOB4	1,04
olfactory receptor, family 2, subfamily M, member 2	OR2M2	1,04
Fc receptor-like 6	FCRL6	1,04
early B-cell factor 1	EBF1	1,04
arachidonate 15-lipoxygenase, type B	ALOX15B	1,04
Memczak2013 ALT_ACCEPTOR, ALT_DONOR, coding, INTERNAL, intronic best transcript NM_003502	AXIN1	1,04
zinc finger protein 502	ZNF502	1,04
p21 protein (Cdc42/Rac)-activated kinase 3	PAK3	1,04

zinc finger protein 324B	ZNF324B	1,03
chromosome 1 open reading frame 220	C1orf220	1,03
MIR670 host gene; hydroxysteroid (17-beta) dehydrogenase 12	MIR670HG; HSD17B12	1,03
olfactory receptor, family 7, subfamily E, member 24	OR7E24	1,03
gap junction protein alpha 8	GJA8	1,03
Memczak2013 ANTISENSE, CDS, coding, INTERNAL best transcript NM_001005417	B4GALT2	1,03
autophagy related 16-like 2	ATG16L2	1,03
keratin associated protein 10-6	KRTAP10-6	1,03
helicase-like transcription factor	HLTF	1,03
ring finger protein 135	RNF135	1,03
G protein-coupled receptor 31	GPR31	1,03
zinc finger and BTB domain containing 48	ZBTB48	1,03
zinc finger protein 227	ZNF227	1,03
ZFP37 zinc finger protein	ZFP37	1,03
keratin associated protein 3-3	KRTAP3-3	1,03
kin of IRRE like (Drosophila)	KIRREL	1,03
chromosome 9 open reading frame 66	C9orf66	1,03
G protein-coupled receptor 68	GPR68	1,03
Na+/K+ transporting ATPase interacting 4	NKAIN4	1,03
amidohydrolase domain containing 2	AMDHD2	1,03
aldehyde dehydrogenase 3 family, member A2	ALDH3A2	1,03
ataxin 3	ATXN3	1,03
ankyrin repeat and IBR domain containing 1	ANKIB1	1,03
cathepsin O	CTSO	1,03
serine active site containing 1	SERAC1	1,03
long intergenic non-protein coding RNA 1219	LINC01219	1,03
Transcript Identified by AceView, Entrez Gene ID(s) 4684	NCAM1	1,03
polypeptide N-acetylgalactosaminyltransferase 12	GALNT12	1,03
BUB1 mitotic checkpoint serine/threonine kinase B	BUB1B	1,03
neurogenin 1	NEUROG1	1,03
NME/NM23 nucleoside diphosphate kinase 3	NME3	1,03
killer cell lectin-like receptor subfamily B, member 1	KLRB1	1,03
ZFP30 zinc finger protein	ZFP30	1,03
RBM14-RBM4 readthrough	RBM14-RBM4	1,03
nebulin	NEB	1,03
aristaless related homeobox	ARX	1,03
ABO blood group (transferase A, alpha 1-3-N-acetylgalactosaminyltransferase; transferase B, alpha 1-3-galactosyltransferase) [Source:HGNC Symbol;Acc:HGNC:79]	ABO	1,03
long intergenic non-protein coding RNA 1189	LINC01189	1,03
zinc finger protein 879	ZNF879	1,03

LSM12 homolog	LSM12	1,03
DLC1 Rho GTPase activating protein	DLC1	1,03
RNA binding motif protein 6	RBM6	1,03
SEC14 and spectrin domains 1	SESTD1	1,03
chromosome 6 open reading frame 222	C6orf222	1,03
keratin 82, type II	KRT82	1,03
FANCD2 opposite strand	FANCD2OS	1,03
lymphocyte antigen 6 complex, locus G5C	LY6G5C	1,03
NADH dehydrogenase (ubiquinone) complex I, assembly factor 4	NDUFAF4	1,03
neural EGFL like 2	NELL2	1,03
adrenoceptor beta 3	ADRB3	1,03
coiled-coil glutamate rich protein 2	CCER2	1,03
sperm acrosome associated 4	SPACA4	1,03
spleen tyrosine kinase	SYK	1,03
aldolase A, fructose-bisphosphate	ALDOA	1,03
tumor susceptibility 101	TSG101	1,03
regulator of G-protein signaling 19	RGS19	1,03
GA binding protein transcription factor, beta subunit 2	GABPB2	1,03
tryptophan 2,3-dioxygenase	TDO2	1,03
protein phosphatase 1, regulatory subunit 32	PPP1R32	1,03
calcium/calmodulin-dependent protein kinase II alpha	CAMK2A	1,03
keratin 83, type II	KRT83	1,03
olfactory receptor, family 5, subfamily I, member 1	OR5I1	1,03
tetratricopeptide repeat domain 9C	TTC9C	1,03
ATPase, Ca++ transporting, cardiac muscle, slow twitch 2	ATP2A2	1,03
adrenoceptor alpha 2A	ADRA2A	1,03
calicin	CCIN	1,03

protocadherin gamma subfamily C, 3; protocadherin gamma subfamily A, 12; protocadherin gamma subfamily B, 4; protocadherin gamma subfamily A, 8; protocadherin gamma subfamily A, 1; protocadherin gamma subfamily A, 10; protocadherin gamma subfamily A, 11; protocadherin gamma subfamily A, 2; protocadherin gamma subfamily A, 3; protocadherin gamma subfamily A, 4; protocadherin gamma subfamily A, 5; protocadherin gamma subfamily A, 6; protocadherin gamma subfamily A, 7; protocadherin gamma subfamily A, 9; protocadherin gamma subfamily B, 1; protocadherin gamma subfamily B, 2; protocadherin gamma subfamily B, 3; protocadherin gamma subfamily B, 5; protocadherin gamma subfamily B, 6; protocadherin gamma subfamily B, 7; protocadherin gamma subfamily C, 4; protocadherin gamma subfamily C, 5	PCDHGC3; PCDHGA12; PCDHGB4; PCDHGA8; PCDHGA1; PCDHGA10; PCDHGA11; PCDHGA2; PCDHGA3; PCDHGA4; PCDHGA5; PCDHGA6; PCDHGA7; PCDHGA9; PCDHGB1; PCDHGB2; PCDHGB3; PCDHGB5; PCDHGB6; PCDHGB7; PCDHGC4; PCDHGC5	1,03
chromosome 10 open reading frame 99	C10orf99	1,03
killer cell lectin-like receptor subfamily F, member 1	KLRF1	1,03
synaptophysin-like 1	SYPL1	1,03
vascular cell adhesion molecule 1	VCAM1	1,03
cell division cycle associated 2	CDCA2	1,03
ST8 alpha-N-acetyl-neuraminide alpha-2,8-sialyltransferase 4	ST8SIA4	1,03
ADAM metallopeptidase with thrombospondin type 1 motif 13	ADAMTS13	1,03
cerebellin 4 precursor	CBLN4	1,03
S-phase cyclin A-associated protein in the ER	SCAPER	1,03
mastermind-like transcriptional coactivator 3	MAML3	1,03
uncharacterized LOC100130451; novel transcript (LOC100130451); novel transcript	LOC100130451; AC079610.2; RP11-105N14.2	1,03
carbonic anhydrase I	CA1	1,03
homeobox B2	HOXB2	1,03
protocadherin-related 15	PCDH15	1,03

selenoprotein M; selenoprotein M [Source:EntrezGene;Acc:140606]; Salzman2013 ANNOTATED, CDS, coding, OVCODE, OVERLAPTX, OVEXON, UTR3 best transcript NM_080430; Salzman2013 ANNOTATED, CDS, coding, OVCODE, OVERLAPTX, OVEXON, UTR3, UTR5 best transcript NM_080430; Transcript Identified by AceView, Entrez Gene ID(s) 140606	SELM	1,03
kelch domain containing 4	KLHDC4	1,03
RIMS binding protein 2	RIMBP2	1,03
ADAM metallopeptidase domain 21	ADAM21	1,03
calcitonin-related polypeptide beta	CALCB	1,03
Salzman2013 ANTISENSE, coding, INTERNAL, intronic best transcript NM_001153	ANXA4	1,03
nuclear factor of activated T-cells, cytoplasmic, calcineurin-dependent 2 interacting protein; microRNA 4517	NFATC2IP; MIR4517	1,03
zinc finger protein 705A; family with sequence similarity 66, member C	ZNF705A; FAM66C	1,03
ankyrin repeat domain 28	ANKRD28	1,03
Cbp/p300-interacting transactivator, with Glu/Asp rich carboxy-terminal domain, 4	CITED4	1,03
DNA nucleotidylexotransferase	DNTT	1,03
protein phosphatase 1, regulatory (inhibitor) subunit 14A	PPP1R14A	1,03
caspase 1	CASP1	1,03
zinc finger, MYM-type 5	ZMYM5	1,03
abhydrolase domain containing 6	ABHD6	1,03
trophinin	TRO	1,03
NOP2 nucleolar protein	NOP2	1,03
testis specific protein, Y-linked 3	TSPY3	1,03
mitochondrial ribosomal protein L55	MRPL55	1,03
unc-51 like kinase 4	ULK4	1,03
nuclear receptor binding factor 2	NRBF2	1,03
chromosome 15 open reading frame 56	C15orf56	1,03
FYVE, RhoGEF and PH domain containing 5	FGD5	1,03
family with sequence similarity 200, member A	FAM200A	1,03
P antigen family, member 4 (prostate associated)	PAGE4	1,03
ripply transcriptional repressor 1	RIPPLY1	1,03
MAX interactor 1, dimerization protein	MXI1	1,03
EPH receptor A5	EPHA5	1,03
SCO-spondin	SSPO	1,03
KIAA0319	KIAA0319	1,03
polypyrimidine tract binding protein 2	PTBP2	1,03
SIN3 transcription regulator family member B	SIN3B	1,03

calpain 15; microRNA 5587	CAPN15; MIR5587	1,03
chromosome 1 open reading frame 229	C1orf229	1,03
MAGE family member A6	MAGEA6	1,03
Memczak2013 ALT_ACCEPTOR, ALT_DONOR, coding, INTERNAL, intronic best transcript NM_001099272	BTBD9	1,03
cholinergic receptor, nicotinic beta 2	CHRNB2	1,03
N-acetyltransferase 14 (GCN5-related, putative)	NAT14	1,03
ribosomal RNA processing 15 homolog	RRP15	1,03
UL16 binding protein 2	ULBP2	1,03
VANGL planar cell polarity protein 2	VANGL2	1,03
scavenger receptor class A, member 5	SCARA5	1,03
potassium channel, voltage gated Shal related subfamily D, member 3	KCND3	1,03
parathyroid hormone 2 receptor	PTH2R	1,03
KIAA1755	KIAA1755	1,03
neurexophilin and PC-esterase domain family, member 2	NXPE2	1,03
chemokine (C-X-C motif) receptor 5	CXCR5	1,03
protein kinase, membrane associated tyrosine/threonine 1	PKMYT1	1,03
RAB9A, member RAS oncogene family	RAB9A	1,03
trans-golgi network vesicle protein 23 homolog A (S. cerevisiae)	TVP23A	1,03
CCR4-NOT transcription complex subunit 3	CNOT3	1,03
dynein assembly factor with WDR repeat domains 1	DAW1	1,03
germinal center-associated, signaling and motility-like	GCSAML	1,03
synaptoporin	SYNPR	1,03
glutamate receptor, ionotropic, N-methyl-D-aspartate 3B	GRIN3B	1,03
zinc finger protein 790	ZNF790	1,03
chromosome 5 open reading frame 58	C5orf58	1,03
fibronectin type III domain containing 5	FNDC5	1,03
coiled-coil domain containing 84	CCDC84	1,03
calcium/calmodulin-dependent serine protein kinase (MAGUK family)	CASK	1,03
long intergenic non-protein coding RNA 92	LINC00092	1,03
collagen, type XIX, alpha 1	COL19A1	1,03
major histocompatibility complex, class I, G	HLA-G	1,03
Transcript Identified by AceView, Entrez Gene ID(s) 56890	MDM1	1,03
fucosyltransferase 9 (alpha (1,3) fucosyltransferase)	FUT9	1,03
phosphoglycerate mutase 2 (muscle)	PGAM2	1,03
keratin 25, type I	KRT25	1,03
C-reactive protein, pentraxin-related	CRP	1,03

leucine rich repeat containing 38	LRRC38	1,03
hypoxanthine phosphoribosyltransferase 1	HPRT1	1,03
BAH domain and coiled-coil containing 1	BAHCC1	1,03
Transcript Identified by AceView, Entrez Gene ID(s) 60672	MIIP	1,03
transient receptor potential cation channel, subfamily C, member 3	TRPC3	1,03
optic atrophy 1 (autosomal dominant)	OPA1	1,03
transmembrane protein 207	TMEM207	1,03
ribosomal protein L32; small nucleolar RNA, H/ACA box 7A	RPL32; SNORA7A	1,03
cysteine rich C-terminal 1	CRCT1	1,03
developing brain homeobox 2	DBX2	1,03
solute carrier organic anion transporter family, member 6A1	SLCO6A1	1,03
anaphase promoting complex subunit 1	ANAPC1	1,03
CTAGE family, member 8	CTAGE8	1,03
PYCARD antisense RNA 1	PYCARD-AS1	1,03
zinc finger, DHHC-type containing 3	ZDHHC3	1,03
KRAB-A domain containing 2	KRBA2	1,03
V-set and transmembrane domain containing 1	VSTM1	1,03
putative homeodomain transcription factor 2	PHTF2	1,03
retbindin	RTBDN	1,03
myelin regulatory factor-like	MYRFL	1,03
ring finger protein 225	RNF225	1,03
Norrie disease (pseudoglioma)	NDP	1,03
olfactory receptor, family 6, subfamily K, member 2	OR6K2	1,03
solute carrier family 4 (sodium bicarbonate cotransporter), member 5	SLC4A5	1,03
galactosylceramidase	GALC	1,03
nuclear cap binding subunit 3	NCBP3	1,03
zinc finger protein 501 [Source:HGNC Symbol;Acc:HGNC:23717]	ZNF501	1,03
eukaryotic elongation factor 2 lysine methyltransferase	EEF2KMT	1,03
Zhang2013 ALT_ACCEPTOR, ALT_DONOR, coding, INTERNAL, intronic best transcript NM_001670	ARVCF	1,03
olfactory receptor, family 4, subfamily X, member 2 (gene/pseudogene)	OR4X2	1,03
leukocyte receptor tyrosine kinase	LTK	1,03
zinc finger protein 428	ZNF428	1,03
Memczak2013 ANTISENSE, coding, INTERNAL, intronic best transcript NM_175900; novel transcript, antisense to C16orf54	AC009133.17; C16orf54	1,03
sphingomyelin phosphodiesterase 4, neutral membrane (neutral sphingomyelinase-3)	SMPD4	1,03

glycosylphosphatidylinositol anchored high density lipoprotein binding protein 1	GPIHBP1	1,03
nicotinate phosphoribosyltransferase	NAPRT	1,03
olfactory receptor, family 51, subfamily G, member 2	OR51G2	1,03
neuropeptide Y receptor Y4	NPY4R	1,03
histone cluster 1, H1d	HIST1H1D	1,03
olfactory receptor, family 4, subfamily D, member 2	OR4D2	1,03
olfactory receptor, family 2, subfamily W, member 1	OR2W1	1,03
myosin, heavy chain 14, non-muscle	MYH14	1,03
family with sequence similarity 162, member B	FAM162B	1,03
deoxyhypusine synthase	DHPS	1,03
tubby like protein 2	TULP2	1,03
polymeric immunoglobulin receptor	PIGR	1,03
desmoplakin	DSP	1,03
olfactory receptor, family 4, subfamily A, member 15	OR4A15	1,03
WAP four-disulfide core domain 8	WFDC8	1,03
serum amyloid A2; SAA2-SAA4 readthrough; serum amyloid A4, constitutive	SAA2; SAA2-SAA4; SAA4	1,03
ATPase, H+ transporting, lysosomal 56/58kDa, V1 subunit B1	ATP6V1B1	1,03
ubiquilin 2	UBQLN2	1,03
zinc finger (CCCH type), RNA binding motif and serine/arginine rich 2	ZRSR2	1,03
F-box protein 41	FBXO41	1,03
zinc finger protein 598	ZNF598	1,03
CD8b molecule	CD8B	1,03
long intergenic non-protein coding RNA 552	LINC00552	1,03
NAD(P)H dehydrogenase, quinone 2	NQO2	1,03
ST6 (alpha-N-acetyl-neuraminyl-2,3-beta-galactosyl-1,3)-N-acetylgalactosaminide alpha-2,6-sialyltransferase 4	ST6GALNAC4	1,03
zinc finger protein 420	ZNF420	1,03
zinc finger protein 707	ZNF707	1,03
neuromedin S	NMS	1,03
chemokine (C-X-C motif) ligand 9	CXCL9	1,03
serine/threonine kinase-like domain containing 1 [Source:HGNC Symbol;Acc:HGNC:28669]	STKLD1	1,03
protocadherin beta 3	PCDHB3	1,03
Transcript Identified by AceView, Entrez Gene ID(s) 8453; novel transcript	CUL2; RP11-297A16.2	1,03
cadherin 3, type 1, P-cadherin (placental)	CDH3	1,03
adipogenin	ADIG	1,03
CD207 molecule, langerin	CD207	1,03
endogenous retrovirus group W, member 1	ERVW-1	1,03
cytochrome P450, family 4, subfamily X, polypeptide 1	CYP4X1	1,03

cytochrome c oxidase subunit VIb polypeptide 2 (testis)	COX6B2	1,03
BTB (POZ) domain containing 18	BTBD18	1,03
potassium channel tetramerization domain containing 1	KCTD1	1,03
potassium channel, calcium activated intermediate/small conductance subfamily N alpha, member 2	KCNN2	1,03
peptidylprolyl isomerase A (cyclophilin A)-like 4C	PPIAL4C	1,03
POM121 and ZP3 fusion	POMZP3	1,03
mitochondrial ribosomal protein S30	MRPS30	1,03
adhesion molecule with Ig-like domain 1	AMIGO1	1,03
Zhang2013 ALT_ACCEPTOR, ALT_DONOR, coding, INTERNAL, intronic best transcript NM_002482	NASP	1,03
transmembrane protein 42	TMEM42	1,03
interleukin 12B	IL12B	1,03
protein phosphatase 1, regulatory subunit 3F	PPP1R3F	1,03
arachidonate 12-lipoxygenase, 12R type	ALOX12B	1,03
shisa family member 8	SHISA8	1,03
zinc finger with KRAB and SCAN domains 7	ZKSCAN7	1,03
lemur tyrosine kinase 2	LMTK2	1,03
centromere protein C	CENPC	1,03
PHD finger protein 6	PHF6	1,03
Sin3A associated protein 25kDa	SAP25	1,03
Rho guanine nucleotide exchange factor 35	ARHGEF35	1,03
olfactory receptor, family 9, subfamily Q, member 2	OR9Q2	1,03
polypeptide N-acetylgalactosaminyltransferase 8	GALNT8	1,03
olfactory receptor, family 6, subfamily X, member 1	OR6X1	1,03
PR domain containing 15	PRDM15	1,03
zinc finger, DHHC-type containing 3	ZDHHC3	1,03
protocadherin beta 4	PCDHB4	1,03
leucine rich repeat containing 18	LRRC18	1,03
cathepsin F	CTSF	1,03
keratin 37, type I	KRT37	1,03
v-myb avian myeloblastosis viral oncogene homolog-like 1	MYBL1	1,03
solute carrier family 7 (anionic amino acid transporter), member 13	SLC7A13	1,03
centrosomal protein 131kDa	CEP131	1,03
major histocompatibility complex, class I-related	MR1	1,03
peptidyl arginine deiminase, type IV	PADI4	1,03
doublesex and mab-3 related transcription factor 2	DMRT2	1,03
misato 1, mitochondrial distribution and morphology regulator; misato family member 2, pseudogene	MSTO1; MSTO2P	1,03
keratin associated protein 22-1	KRTAP22-1	1,03
wingless-type MMTV integration site family, member 10B	WNT10B	1,03

neuronal tyrosine-phosphorylated phosphoinositide-3-kinase adaptor 2	NYAP2	1,03
abhydrolase domain containing 16B	ABHD16B	1,03
chromosome 11 open reading frame 70	C11orf70	1,03
SYNJ2BP-COX16 readthrough; COX16 cytochrome c oxidase assembly homolog; synaptojanin 2 binding protein	SYNJ2BP-COX16; COX16; SYNJ2BP	1,03
ADAM metallopeptidase domain 17	ADAM17	1,03
transmembrane protein 225	TMEM225	1,03
myosin, heavy chain 13, skeletal muscle	MYH13	1,03
solute carrier family 13 (sodium-dependent dicarboxylate transporter), member 2	SLC13A2	1,03
interleukin 21	IL21	1,03
RAB3 GTPase activating protein subunit 2 (non-catalytic); aurora kinase A pseudogene 1	RAB3GAP2; AURKAPS1	1,03
CTAGE family, member 9	CTAGE9	1,03
R3H domain containing-like	R3HDML	1,03
homeobox B13	HOXB13	1,03
BarH-like homeobox 1	BARHL1	1,03
transmembrane protein 8B	TMEM8B	1,03
chromosome 6 open reading frame 25	C6orf25	1,03
DnaJ (Hsp40) homolog, subfamily C, member 28	DNAJC28	1,03
EPH receptor B2	EPHB2	1,03
ankyrin repeat domain 40	ANKRD40	1,03
RNA binding motif protein 39	RBM39	1,03
SET and MYND domain containing 4	SMYD4	1,03
FOXL2 neighbor	FOXL2NB	1,03
cadherin 26	CDH26	1,03
crystallin gamma B	CRYGB	1,03
chromosome 8 open reading frame 74	C8orf74	1,03
prune homolog 2 (Drosophila)	PRUNE2	1,03
immunoglobulin superfamily, member 3	IGSF3	1,03
frequently rearranged in advanced T-cell lymphomas 1	FRAT1	1,03
chemokine (C-X-C motif) ligand 10	CXCL10	1,03
lipoma HMGIC fusion partner-like 2	LHFPL2	1,03
transmembrane protein 206	TMEM206	1,03
casein kinase 2, alpha 1 polypeptide	CSNK2A1	1,03
ubiquitin specific peptidase 39	USP39	1,03
zygote arrest 1-like	ZAR1L	1,03
zinc finger and BTB domain containing 32	ZBTB32	1,03
signal peptide, CUB domain, EGF-like 1	SCUBE1	1,03
lectin, mannose-binding, 1 like	LMAN1L	1,03
translation machinery associated 7 homolog	TMA7	1,03
G protein-coupled receptor associated sorting protein 1	GPRASP1	1,02

Homo sapiens acetylserotonin O-methyltransferase (ASMT), transcript variant 2, mRNA.	ASMT	1,02
ST3 beta-galactoside alpha-2,3-sialyltransferase 3; microRNA 6079	ST3GAL3; MIR6079	1,02
zinc finger protein 223	ZNF223	1,02
WNK lysine deficient protein kinase 4	WNK4	1,02
ATPase, Ca++ transporting, cardiac muscle, fast twitch 1	ATP2A1	1,02
potassium channel, voltage gated subfamily E regulatory beta subunit 1	KCNE1	1,02
homeobox C10	HOXC10	1,02
Rho guanine nucleotide exchange factor 33	ARHGEF33	1,02
ribosomal RNA processing 1B	RRP1B	1,02
seizure related 6 homolog (mouse)-like	SEZ6L	1,02
ankyrin repeat and ubiquitin domain containing 1	ANKUB1	1,02
transmembrane protein 229A	TMEM229A	1,02
Transcript Identified by AceView, Entrez Gene ID(s) 23122	CLASP2	1,02
ribosomal protein S6 kinase, 90kDa, polypeptide 1	RPS6KA1	1,02
tektin 2 (testicular)	TEKT2	1,02
solute carrier family 37 (glucose-6-phosphate transporter), member 1	SLC37A1	1,02
suppression of tumorigenicity 18, zinc finger	ST18	1,02
IgLON family member 5	IGLON5	1,02
CMT1A duplicated region transcript 15	CDRT15	1,02
triggering receptor expressed on myeloid cells-like 1	TREML1	1,02
tripartite motif containing 62	TRIM62	1,02
zinc finger and AT hook domain containing	ZFAT	1,02
olfactory receptor, family 2, subfamily C, member 3; GCSAML antisense RNA 1	OR2C3; GCSAML-AS1	1,02
kelch-like family member 40	KLHL40	1,02
SPANX family, member N5	SPANXN5	1,02
scratch family zinc finger 2	SCRT2	1,02
family with sequence similarity 109, member B	FAM109B	1,02
tolloid like 2	TLL2	1,02
trafficking protein particle complex 3-like	TRAPPC3L	1,02
defensin, alpha 3, neutrophil-specific	DEFA3	1,02
family with sequence similarity 220, member A	FAM220A	1,02
WEE1 homolog 2 (S. pombe)	WEE2	1,02
dynein, axonemal, light chain 4	DNAL4	1,02
acyl-CoA dehydrogenase family, member 8	ACAD8	1,02
myogenic factor 5	MYF5	1,02
ets variant 1	ETV1	1,02
insulin receptor substrate 4	IRS4	1,02
histidine decarboxylase	HDC	1,02

RNA binding motif protein 14	RBM14	1,02
solute carrier family 26 (anion exchanger), member 4	SLC26A4	1,02
EPH receptor A2	EPHA2	1,02
Homo sapiens synaptotagmin XV (SYT15), transcript variant a, mRNA.; Homo sapiens synaptotagmin XV (SYT15), transcript variant b, mRNA.; Homo sapiens synaptotagmin XV, mRNA (cDNA clone MGC:164845 IMAGE:40147906), complete cds.; Synaptotagmin-15 [Source:UniProtKB/Swiss-Prot;Acc:Q9BQS2]	SYT15; ABC7-42404400C24.1	1,02
phospholipase A2, group IIF	PLA2G2F	1,02
proteoglycan 2, bone marrow (natural killer cell activator, eosinophil granule major basic protein)	PRG2	1,02
chromosome 18 open reading frame 21	C18orf21	1,02
T-box 22	TBX22	1,02
uncharacterized LOC100130880; novel transcript	LOC100130880; AC022173.2	1,02
uncharacterized LOC101928093; novel transcript	LOC101928093; RP11-779O18.1	1,02
G protein-coupled receptor 180	GPR180	1,02
exocyst complex component 3-like 1	EXOC3L1	1,02
BPI fold containing family B, member 4	BPIFB4	1,02
HEAT repeat containing 9	HEATR9	1,02
dual specificity phosphatase 2	DUSP2	1,02
microfibrillar associated protein 3 like	MFAP3L	1,02
glycine receptor alpha 2	GLRA2	1,02
minichromosome maintenance complex component 3 associated protein	MCM3AP	1,02
1-acylglycerol-3-phosphate O-acyltransferase 1; microRNA 6721	AGPAT1; MIR6721	1,02
adenylate kinase 1	AK1	1,02
thyroid peroxidase	TPO	1,02
protein tyrosine phosphatase domain containing 1	PTPDC1	1,02
F-box protein 47	FBXO47	1,02
peroxisome proliferator-activated receptor gamma, coactivator-related 1	PPRC1	1,02
solute carrier family 32 (GABA vesicular transporter), member 1	SLC32A1	1,02
wingless-type MMTV integration site family, member 7A	WNT7A	1,02
transmembrane protein 249	TMEM249	1,02
potassium channel, inwardly rectifying subfamily J, member 4	KCNJ4	1,02
growth arrest and DNA-damage-inducible, gamma	GADD45G	1,02
proteasome (prosome, macropain) subunit, beta type, 11	PSMB11	1,02

uncharacterized LOC339059; novel transcript, antisense to FAM38A	LOC339059; RP5-1142A6.8	1,02
THUMP domain containing 2	THUMPD2	1,02
long intergenic non-protein coding RNA 885	LINC00885	1,02
solute carrier family 9, member C2 (putative)	SLC9C2	1,02
diacylglycerol kinase, delta 130kDa	DGKD	1,02
F-box and WD repeat domain containing 9	FBXW9	1,02
vav 1 guanine nucleotide exchange factor	VAV1	1,02
hemochromatosis type 2 (juvenile)	HFE2	1,02
ribosomal protein S28	RPS28	1,02
Transcript Identified by AceView, Entrez Gene ID(s) 80232	WDR26	1,02
anaphase promoting complex subunit 11	ANAPC11	1,02
mucin 4, cell surface associated	MUC4	1,02
carboxylesterase 5A	CES5A	1,02
fibrinogen C domain containing 1	FIBCD1	1,02
aldehyde dehydrogenase 8 family, member A1	ALDH8A1	1,02
olfactory receptor, family 6, subfamily N, member 1	OR6N1	1,02
serine/threonine kinase 11 interacting protein	STK11IP	1,02
2,4-dienoyl-CoA reductase 2, peroxisomal	DECR2	1,02
ribosomal protein S4, Y-linked 1	RPS4Y1	1,02
solute carrier family 25, member 35	SLC25A35	1,02
tumor protein D52	TPD52	1,02
suppression of tumorigenicity 13 (colon carcinoma) (Hsp70 interacting protein)	ST13	1,02
nucleotide-binding oligomerization domain containing 2	NOD2	1,02
defensin, beta 103A	DEFB103A	1,02
translocator protein (18kDa)	TSPO	1,02
engulfment and cell motility 2	ELMO2	1,02
mediator complex subunit 7	MED7	1,02
zygote arrest 1	ZAR1	1,02
chromosome 9 open reading frame 163	C9orf163	1,02
solute carrier family 22, member 14	SLC22A14	1,02
spindlin 1	SPIN1	1,02
SET-like protein	SETSIP	1,02
small nuclear RNA activating complex polypeptide 3	SNAPC3	1,02
Transcript Identified by AceView, Entrez Gene ID(s) 149297; novel transcript	RP11-9L18.3; FAM78B	1,02
ring finger protein 215	RNF215	1,02
transglutaminase 4	TGM4	1,02
neuroblastoma breakpoint family, member 7	NBPF7	1,02
olfactory receptor, family 2, subfamily M, member 5	OR2M5	1,02
protein phosphatase 6, catalytic subunit	PPP6C	1,02
perilipin 4	PLIN4	1,02
T-cell lymphoma invasion and metastasis 1	TIAM1	1,02

ubiquinol-cytochrome c reductase, complex III subunit XI	UQCR11	1,02
angiotensin I converting enzyme	ACE	1,02
apolipoprotein A-II	APOA2	1,02
G protein-coupled receptor 62	GPR62	1,02
SHQ1, H/ACA ribonucleoprotein assembly factor	SHQ1	1,02
transmembrane 6 superfamily member 1	TM6SF1	1,02
glyceraldehyde-3-phosphate dehydrogenase	GAPDH	1,02
keratin 27, type I	KRT27	1,02
muscular LMNA-interacting protein	MLIP	1,02
myosin IH	MYO1H	1,02
zinc finger and SCAN domain containing 31	ZSCAN31	1,02
SEC14-like lipid binding 6	SEC14L6	1,02
guanine deaminase	GDA	1,02
polymerase (DNA directed), epsilon, catalytic subunit	POLE	1,02
iroquois homeobox 6	IRX6	1,02
zinc finger, CCHC domain containing 18	ZCCHC18	1,02
chromosome 16 open reading frame 97	C16orf97	1,02
defensin, beta 123	DEFB123	1,02
microtubule associated tumor suppressor candidate 2	MTUS2	1,02
ankyrin 3, node of Ranvier (ankyrin G)	ANK3	1,02
myotubularin related protein 7	MTMR7	1,02
chromosome 1 open reading frame 228	C1orf228	1,02
tubulin, gamma complex associated protein 2	TUBGCP2	1,02
Memczak2013 ALT_ACCEPTOR, ALT_DONOR, coding, INTERNAL, intronic best transcript NM_003369	UVRAG	1,02
calmodulin-like 3	CALML3	1,02
family with sequence similarity 177, member B	FAM177B	1,02
olfactory receptor, family 10, subfamily AD, member 1	OR10AD1	1,02
olfactory receptor, family 5, subfamily K, member 2	OR5K2	1,02
protocadherin 8	PCDH8	1,02
LysM, putative peptidoglycan-binding, domain containing 1	LYSMD1	1,02
chromosome 1 open reading frame 141	C1orf141	1,02
X-ray repair complementing defective repair in Chinese hamster cells 2	XRCC2	1,02
coiled-coil domain containing 39	CCDC39	1,02
CUB and Sushi multiple domains 3	CSMD3	1,02
PWP2 periodic tryptophan protein homolog (yeast)	PWP2	1,02
chymotrypsinogen B2	CTRB2	1,02
ORAI calcium release-activated calcium modulator 2	ORAI2	1,02
adenylate kinase 9	AK9	1,02
Memczak2013 ANTISENSE, INTERNAL, ncRNA best transcript NR_003587	MYO15B	1,02
receptor tyrosine kinase-like orphan receptor 2	ROR2	1,02
casein kinase 2, alpha prime polypeptide	CSNK2A2	1,02

RNA binding motif protein 41	RBM41	1,02
acyl-CoA thioesterase 8	ACOT8	1,02
B-cell CLL/lymphoma 7C	BCL7C	1,02
transmembrane protein 104	TMEM104	1,02
ubiquitin conjugating enzyme E2B	UBE2B	1,02
G protein-coupled receptor 35	GPR35	1,02
Transcript Identified by AceView, Entrez Gene ID(s) 4211	MEIS1	1,02
dispatched homolog 2 (Drosophila)	DISP2	1,02
UDP glycosyltransferase 8	UGT8	1,02
microseminoprotein, prostate associated	MSMP	1,02
mutated in colorectal cancers	MCC	1,02
phosphoribosyl pyrophosphate synthetase 1-like 1	PRPS1L1	1,02
dynactin 1	DCTN1	1,02
peptidylprolyl isomerase A (cyclophilin A)-like 4E	PPIAL4E	1,02
protease, serine, 33	PRSS33	1,02
testis specific protein, Y-linked 2	TSPY2	1,02
D-tyrosyl-tRNA deacylase 1	DTD1	1,02
LIM homeobox 4	LHX4	1,02
coiled-coil domain containing 88B	CCDC88B	1,02
maestro heat-like repeat family member 7; tetratricopeptide repeat domain 4; MROH7-TTC4 readthrough (NMD candidate)	MROH7; TTC4; MROH7-TTC4	1,02
exonuclease 3-5 domain containing 3	EXD3	1,02
retinoschisin 1	RS1	1,02
transient receptor potential cation channel, subfamily M, member 1	TRPM1	1,02
chromosome 19 open reading frame 38	C19orf38	1,02
leucine rich repeat and fibronectin type III domain containing 4	LRFN4	1,02
ubiquitin-conjugating enzyme E2Z	UBE2Z	1,02
calcium channel, voltage-dependent, gamma subunit 2	CACNG2	1,02
mitochondrial methionyl-tRNA formyltransferase	MTFMT	1,02
CD1b molecule	CD1B	1,02
TBC1 domain family, member 13	TBC1D13	1,02
Sp9 transcription factor	SP9	1,02
CCHC-type zinc finger, nucleic acid binding protein	CNBP	1,02
cytochrome P450, family 1, subfamily B, polypeptide 1	CYP1B1	1,02
taste receptor, type 2, member 40	TAS2R40	1,02
adenosine deaminase domain containing 1	ADAD1	1,02
guanine nucleotide binding protein (G protein), alpha 15 (Gq class)	GNA15	1,02
acid phosphatase, prostate	ACPP	1,02
glycophorin B (MNS blood group)	GYPB	1,02
DEAD (Asp-Glu-Ala-Asp) box polypeptide 39A	DDX39A	1,02

calcium channel, voltage-dependent, gamma subunit 7	CACNG7	1,02
Fc fragment of IgG, high affinity Ib, receptor (CD64)	FCGR1B	1,02
far upstream element (FUSE) binding protein 3	FUBP3	1,02
inositol polyphosphate-5-phosphatase D	INPP5D	1,02
carboxypeptidase X (M14 family), member 1	CPXM1	1,02
prostate cancer susceptibility candidate 1	PRAC1	1,02
monoglyceride lipase	MGLL	1,02
calpastatin	CAST	1,02
long intergenic non-protein coding RNA 982	LINC00982	1,02
KIAA1429	KIAA1429	1,02
KIAA1211-like	KIAA1211L	1,02
cyclic nucleotide gated channel beta 1	CNGB1	1,02
zinc finger protein 485	ZNF485	1,02
Memczak2013 ANTISENSE, coding, INTERNAL, intronic best transcript NM_052931	SLAMF6	1,02
putative uncharacterized protein MGC39545; novel transcript, antisense to FEZ1	LOC403312; AP000708.1	1,02
Sp7 transcription factor	SP7	1,02
striatin interacting protein 1	STRIP1	1,02
variable charge, X-linked; variable charge, X-linked 3A	VCX; VCX3A	1,02
zinc finger protein 627	ZNF627	1,02
Transcript Identified by AceView, Entrez Gene ID(s) 128209; novel transcript	RP5-1198O20.4; KLF17	1,02
ISY1 splicing factor homolog	ISY1	1,02
DnaJ (Hsp40) homolog, subfamily C, member 5 beta	DNAJC5B	1,02
family with sequence similarity 132, member A	FAM132A	1,02
RAS guanyl releasing protein 4	RASGRP4	1,02
basic leucine zipper transcription factor, ATF-like 3	BATF3	1,02
transmembrane protease, serine 11B	TMPRSS11B	1,02
uncharacterized LOC100131496; novel transcript, antisense to ZMYND8	LOC100131496; RP4-569M23.5	1,02
interferon, lambda receptor 1	IFNLR1	1,02
ribosomal protein L7a	RPL7A	1,02
nuclear factor of kappa light polypeptide gene enhancer in B-cells inhibitor-like 1	NFKBIL1	1,02
tRNA selenocysteine 1 associated protein 1	TRNAU1AP	1,02
transmembrane protein 252	TMEM252	1,02
chromosome 6 open reading frame 203	C6orf203	1,02
P antigen family, member 1 (prostate associated)	PAGE1	1,02
ninjurin 2	NINJ2	1,02
fibroblast growth factor 22	FGF22	1,02
teneurin transmembrane protein 1	TENM1	1,02
chromosome 9 open reading frame 114	C9orf114	1,02
activity-regulated cytoskeleton-associated protein	ARC	1,02
kelch-like family member 28	KLHL28	1,02

peptidase inhibitor 15	PI15	1,02
Tu translation elongation factor, mitochondrial; microRNA 4721	TUFM; MIR4721	1,02
orofacial cleft 1 candidate 1	OFCC1	1,02
casein kinase 2, alpha 3 polypeptide	CSNK2A3	1,02
tight junction protein 1	TJP1	1,02
RanBP-type and C3HC4-type zinc finger containing 1	RBCK1	1,02
lemur tyrosine kinase 3	LMTK3	1,02
cytochrome P450, family 2, subfamily D, polypeptide 6	CYP2D6	1,02
ganglioside induced differentiation associated protein 1	GDAP1	1,02
glioma tumor suppressor candidate region gene 1	GLTSCR1	1,02
prostate and breast cancer overexpressed 1	PBOV1	1,02
chromosome 19 open reading frame 67	C19orf67	1,02
IL2-inducible T-cell kinase	ITK	1,02
cytochrome c oxidase assembly factor 1 homolog	COA1	1,02
acid sensing ion channel 3	ASIC3	1,02
zinc finger protein 728	ZNF728	1,02
alanine and arginine rich domain containing protein	AARD	1,02
myosin phosphatase Rho interacting protein	MPRIP	1,02
sorting nexin 19	SNX19	1,02
fibronectin type III and SPRY domain containing 1	FSD1	1,02
KIAA2022	KIAA2022	1,02
antioxidant 1 copper chaperone	ATOX1	1,02
zinc finger protein 284	ZNF284	1,02
amyloid beta (A4) precursor protein-binding, family B, member 3; microRNA 6831	APBB3; MIR6831	1,02
TNFSF12-TNFSF13 readthrough	TNFSF12-TNFSF13	1,02
Transcript Identified by AceView, Entrez Gene ID(s) 9695	EDEM1	1,02
GRAM domain containing 1B	GRAMD1B	1,02
placenta expressed transcript 1	PLET1	1,02
chromosome 17 open reading frame 80	C17orf80	1,02
syntaxin binding protein 1	STXBP1	1,02
cytochrome b-245, alpha polypeptide	CYBA	1,02
small proline-rich protein 2F	SPRR2F	1,02
chromosome 7 open reading frame 69	C7orf69	1,02
nuclear receptor subfamily 4, group A, member 1	NR4A1	1,02
motilin receptor	MLNR	1,02
zinc finger protein 681	ZNF681	1,02
chromosome 11 open reading frame 53	C11orf53	1,02
PRAME family member 25	PRAMEF25	1,02
carbonic anhydrase XIV	CA14	1,02
caspase 12 (gene/pseudogene)	CASP12	1,02
RNA binding motif protein 20	RBM20	1,02

v-crk avian sarcoma virus CT10 oncogene homolog	CRK	1,02
chromosome 1 open reading frame 204	C1orf204	1,02
hemoglobin, epsilon 1	HBE1	1,02
Ras converting CAAX endopeptidase 1; chromosome 11 open reading frame 80	RCE1; C11orf80	1,02
cholinergic receptor, nicotinic beta 3	CHRNB3	1,02
neurofilament, light polypeptide	NEFL	1,02
chromosome 11 open reading frame 91	C11orf91	1,02
metallophosphoesterase domain containing 1	MPPED1	1,02
ureidoimidazoline (2-oxo-4-hydroxy-4-carboxy-5-) decarboxylase	URAD	1,02
proline rich 21	PRR21	1,02
ribosomal protein S6 kinase, 90kDa, polypeptide 1	RPS6KA1	1,02
transmembrane protein 47	TMEM47	1,02
olfactory receptor, family 2, subfamily V, member 1	OR2V1	1,02
senataxin	SETX	1,02
ATP synthase, H+ transporting, mitochondrial Fo complex subunit G	ATP5L	1,02
tudor domain containing 6	TDRD6	1,02
cytokine like 1	CYTL1	1,02
chromosome X open reading frame 67	CXorf67	1,02
NK2 homeobox 3	NKX2-3	1,02
synaptonemal complex central element protein 1-like	SYCE1L	1,02
potassium channel, voltage gated eag related subfamily H, member 8	KCNH8	1,02
potassium channel, inwardly rectifying subfamily J, member 12	KCNJ12	1,02
transmembrane protein 235	TMEM235	1,02
toll-like receptor 2	TLR2	1,02
proline rich Gla (G-carboxyglutamic acid) 1	PRRG1	1,02
sperm associated antigen 16	SPAG16	1,02
NADH dehydrogenase (ubiquinone) 1 alpha subcomplex, 10, 42kDa	NDUFA10	1,02
azurocidin 1	AZU1	1,02
glutamate receptor, ionotropic, delta 2 (Grid2) interacting protein	GRID2IP	1,02
integrin alpha L	ITGAL	1,02
spermatogenesis and centriole associated 1	SPATC1	1,02
cholinergic receptor, muscarinic 3	CHRM3	1,02
methenyltetrahydrofolate synthetase domain containing	MTHFSD	1,02
golgin A6 family-like 22	GOLGA6L22	1,02
transmembrane and immunoglobulin domain containing 2	TMIGD2	1,02
Myb/SANT-like DNA-binding domain containing 1	MSANTD1	1,02

family with sequence similarity 50, member B	FAM50B	1,02
butyrophilin, subfamily 2, member A1	BTN2A1	1,02
RAS-like, family 11, member B	RASL11B	1,02
glycine receptor alpha 3	GLRA3	1,02
RAB3 GTPase activating protein subunit 1 (catalytic)	RAB3GAP1	1,02
coiled-coil domain containing 27	CCDC27	1,01
oxysterol binding protein-like 2	OSBPL2	1,01
Fc receptor-like 5	FCRL5	1,01
v-kit Hardy-Zuckerman 4 feline sarcoma viral oncogene homolog	KIT	1,01
tetraspanin 2	TSPAN2	1,01
teashirt zinc finger homeobox 3	TSHZ3	1,01
protein inhibitor of activated STAT 1	PIAS1	1,01
eukaryotic translation initiation factor 4A1; small nucleolar RNA, C/D box 10; small nucleolar RNA, H/ACA box 67; small nucleolar RNA, H/ACA box 48	EIF4A1; SNORD10; SNORA67; SNORA48	1,01
uncharacterized LOC388780; putative novel transcript	LOC388780; RP11-128M1.1	1,01
matrix metallopeptidase 8	MMP8	1,01
RAD51 recombinase	RAD51	1,01
homeobox D13	HOXD13	1,01
methionyl aminopeptidase 1	METAP1	1,01
aminoacylase 1	ACY1	1,01
PRAME family member 25; PRAME family member 26	PRAMEF25; PRAMEF26	1,01
fizzy/cell division cycle 20 related 1	FZR1	1,01
cat eye syndrome chromosome region, candidate 1	CECR1	1,01
G protein-coupled receptor 174	GPR174	1,01
spermatogenesis associated 33	SPATA33	1,01
kelch domain containing 1	KLHDC1	1,01
homeobox A7	HOXA7	1,01
phospholipase B domain containing 1	PLBD1	1,01
zinc finger protein 26	ZNF26	1,01
Mediterranean fever	MEFV	1,01
dehydrogenase/reductase (SDR family) member 3; microRNA 6730	DHRS3; MIR6730	1,01
chromosome 20 open reading frame 85	C20orf85	1,01
pentraxin 3, long	PTX3	1,01
zinc finger protein 609	ZNF609	1,01
Jeck2013 ANTISENSE, coding, INTERNAL, intronic best transcript NM_005336	HDLBP	1,01
uncharacterized LOC100652929; novel transcript; uncharacterized LOC100652929 [Source:EntrezGene;Acc:100652929]	HP09025; CTD-2116F7.1	1,01

sorting nexin 20	SNX20	1,01
radical S-adenosyl methionine domain containing 2	RSAD2	1,01
RAS-like, family 12	RASL12	1,01
toll-like receptor 10	TLR10	1,01
N(alpha)-acetyltransferase 11, NatA catalytic subunit	NAA11	1,01
RNA binding protein S1, serine-rich domain	RNPS1	1,01
arginyl aminopeptidase (aminopeptidase B)	RNPEP	1,01
death-associated protein kinase 2	DAPK2	1,01
deleted in lymphocytic leukemia, 7	DLEU7	1,01
G protein-coupled receptor 176	GPR176	1,01
protamine 3	PRM3	1,01
indolethylamine N-methyltransferase	INMT	1,01
phosphofructokinase, muscle	PFKM	1,01
methylmalonic aciduria (cobalamin deficiency) cblD type, with homocystinuria	MMADHC	1,01
FYVE, RhoGEF and PH domain containing 3	FGD3	1,01
transient receptor potential cation channel, subfamily M, member 5	TRPM5	1,01
secretoglobin, family 1D, member 2	SCGB1D2	1,01
leucine rich repeat containing 10	LRRC10	1,01
alkaline phosphatase, liver/bone/kidney	ALPL	1,01
vacuolar protein sorting 33 homolog B (yeast)	VPS33B	1,01
cofilin 1 (non-muscle)	CFL1	1,01
WD repeat domain 91	WDR91	1,01
docking protein 2	DOK2	1,01
KIAA1143	KIAA1143	1,01
zinc finger protein 391	ZNF391	1,01
RAB34, member RAS oncogene family	RAB34	1,01
kelch-like family member 34	KLHL34	1,01
Ras association (RalGDS/AF-6) domain family member 1	RASSF1	1,01
WD repeat domain 11	WDR11	1,01
proline rich 33	PRR33	1,01
sparc/osteonectin, cwcv and kazal-like domains proteoglycan (testican) 3	SPOCK3	1,01
phosphatidylinositol-4-phosphate 5-kinase, type I, gamma	PIP5K1C	1,01
monocyte to macrophage differentiation-associated 2	MMD2	1,01
LIM homeobox 5	LHX5	1,01
zinc finger with KRAB and SCAN domains 7	ZKSCAN7	1,01
elaC ribonuclease Z 2	ELAC2	1,01
ankyrin repeat domain 35	ANKRD35	1,01
phospholipase A2, group VI (cytosolic, calcium-independent)	PLA2G6	1,01
armadillo repeat containing 10	ARMC10	1,01

tetratricopeptide repeat domain 27; microRNA 4765	TTC27; MIR4765	1,01
growth hormone releasing hormone	GHRH	1,01
telomere maintenance 2	TELO2	1,01
major histocompatibility complex, class II, DQ alpha 2	HLA-DQA2	1,01
small lysine-rich protein 1	SMKR1	1,01
Transcript Identified by AceView, Entrez Gene ID(s) 640	BLK	1,01
hyaluronan and proteoglycan link protein 4	HAPLN4	1,01
sarcalumenin	SRL	1,01
X-prolyl aminopeptidase (aminopeptidase P) 2, membrane-bound	XPNPEP2	1,01
DEAD (Asp-Glu-Ala-Asp) box polypeptide 54	DDX54	1,01
corticotropin releasing hormone receptor 1	CRHR1	1,01
zinc finger protein 273	ZNF273	1,01
CWC22 homolog, spliceosome-associated protein	CWC22	1,01
iroquois homeobox 2	IRX2	1,01
cytochrome c oxidase subunit VIIa polypeptide 1 (muscle)	COX7A1	1,01
G protein-coupled receptor 158	GPR158	1,01
excision repair cross-complementation group 4	ERCC4	1,01
retinal pigment epithelium-derived rhodopsin homolog	RRH	1,01
olfactory receptor, family 7, subfamily A, member 5	OR7A5	1,01
WD repeat domain 78	WDR78	1,01
retinaldehyde binding protein 1	RLBP1	1,01
golgin A7 family, member B	GOLGA7B	1,01
olfactory receptor, family 2, subfamily A, member 25	OR2A25	1,01
transmembrane protein 11	TMEM11	1,01
GRAM domain containing 1A	GRAMD1A	1,01
chromodomain helicase DNA binding protein 4	CHD4	1,01
neural EGFL like 1	NELL1	1,01
solute carrier family 4 (anion exchanger), member 3	SLC4A3	1,01
chromosome 20 open reading frame 144	C20orf144	1,01
smoothelin-like 1	SMTNL1	1,01
epithelial splicing regulatory protein 1	ESRP1	1,01
olfactory receptor, family 52, subfamily N, member 4 (gene/pseudogene)	OR52N4	1,01
KH homology domain containing 1	KHDC1	1,01
Transcript Identified by AceView, Entrez Gene ID(s) 1785	DNM2	1,01
NIMA-related kinase 5	NEK5	1,01
basic helix-loop-helix family, member e23	BHLHE23	1,01
RNA pseudouridylate synthase domain containing 3	RPUSD3	1,01
olfactory receptor, family 5, subfamily K, member 3	OR5K3	1,01
transmembrane protein 229B	TMEM229B	1,01
Memczak2013 ANTISENSE, coding, INTERNAL, intronic best transcript NM_016463	CXXC5	1,01

SPECC1L-ADORA2A readthrough (NMD candidate)	SPECC1L-ADORA2A	1,01
MSS51 mitochondrial translational activator	MSS51	1,01
twisted gastrulation BMP signaling modulator 1	TWSG1	1,01
leucine rich repeat containing 37, member A5, pseudogene	LRRC37A5P	1,01
radical S-adenosyl methionine domain containing 1	RSAD1	1,01
keratin associated protein 13-4	KRTAP13-4	1,01
pituitary tumor-transforming 1 interacting protein	PTTG1IP	1,01
zinc finger with KRAB and SCAN domains 7	ZKSCAN7	1,01
solute carrier family 4 (sodium bicarbonate cotransporter), member 4	SLC4A4	1,01
glycine-N-acyltransferase-like 2	GLYATL2	1,01
GDNF family receptor alpha 2	GFRA2	1,01
potassium channel, voltage gated modifier subfamily G, member 1	KCNG1	1,01
2-oxoglutarate and iron-dependent oxygenase domain containing 3	OGFOD3	1,01
lacritin	LACRT	1,01
zinc finger protein 846	ZNF846	1,01
ArfGAP with dual PH domains 1	ADAP1	1,01
fibronectin type III and SPRY domain containing 2	FSD2	1,01
Zhang2013 ALT_ACCEPTOR, ALT_DONOR, coding, INTERNAL, intronic best transcript NM_014675	CROCC	1,01
IGF2 antisense RNA	IGF2-AS	1,01
K(lysine) acetyltransferase 2A	KAT2A	1,01
single-pass membrane protein with coiled-coil domains 2	SMCO2	1,01
interferon, lambda 2	IFNL2	1,01
peptidase (mitochondrial processing) alpha	PMPCA	1,01
natriuretic peptide C	NPPC	1,01
tubby like protein 1	TULP1	1,01
leucine rich repeat and fibronectin type III domain containing 3	LRFN3	1,01
relaxin 3	RLN3	1,01
cilia and flagella associated protein 36	CFAP36	1,01
neuron specific gene family member 1; Neuron-specific protein family member 1 [Source:UniProtKB/Swiss-Prot;Acc:P42857]	NSG1; D4S234E	1,01
SPT6 homolog, histone chaperone	SUPT6H	1,01
replication factor C subunit 4	RFC4	1,01
NADH dehydrogenase (ubiquinone) 1 alpha subcomplex, 10, 42kDa	NDUFA10	1,01
polycystic kidney and hepatic disease 1 (autosomal recessive)	PKHD1	1,01

wingless-type MMTV integration site family, member 7B	WNT7B	1,01
TLR4 interactor with leucine-rich repeats	TRIL	1,01
snail family zinc finger 3	SNAI3	1,01
hippocalcin like 4	HPCAL4	1,01
cholinergic receptor, nicotinic beta 4	CHRNB4	1,01
lymphotoxin alpha	LTA	1,01
variable charge, X-linked 3B; variable charge, X-linked	VCX3B; VCX	1,01
keratin associated protein 25-1	KRTAP25-1	1,01
stomatin	STOM	1,01
acrosin	ACR	1,01
serpin peptidase inhibitor, clade B (ovalbumin), member 11 (gene/pseudogene)	SERPINB11	1,01
cystatin 8 (cystatin-related epididymal specific)	CST8	1,01
Memczak2013 ALT_ACCEPTOR, ALT_DONOR, coding, INTERNAL, intronic best transcript NM_015986	CRLF3	1,01
fucosyltransferase 5 (alpha (1,3) fucosyltransferase)	FUT5	1,01
ectonucleotide pyrophosphatase/phosphodiesterase 6	ENPP6	1,01
ventral anterior homeobox 1	VAX1	1,01
KN motif and ankyrin repeat domains 2	KANK2	1,01
guanosine monophosphate reductase	GMPR	1,01
PRAME family member 19	PRAMEF19	1,01
RAD51L3-RFFL readthrough; HCG2039718, isoform CRA_g; Uncharacterized protein [Source:UniProtKB/TrEMBL;Acc:K7EN88]	RAD51L3-RFFL	1,01
radial spoke head 10 homolog B2 (Chlamydomonas); radial spoke head 10 homolog B (Chlamydomonas)	RSPH10B2; RSPH10B	1,01
inositol polyphosphate-5-phosphatase D	INPP5D	1,01
tau tubulin kinase 1	TTBK1	1,01
myotilin	MYOT	1,01
ADAMTS like 2	ADAMTSL2	1,01
potassium channel, voltage gated shaker related subfamily A, member 4	KCNA4	1,01
zinc finger protein 608	ZNF608	1,01
INO80 complex subunit D	INO80D	1,01
ribosomal protein L12	RPL12	1,01
MAGE family member A5	MAGEA5	1,01
MPL proto-oncogene, thrombopoietin receptor	MPL	1,01
ADP-ribosylation factor like GTPase 2	ARL2	1,01
keratin associated protein 9-9	KRTAP9-9	1,01
nibrin	NBN	1,01
single immunoglobulin and toll-interleukin 1 receptor (TIR) domain	SIGIRR	1,01
transmembrane protein 176B	TMEM176B	1,01
chromosome 7 open reading frame 77	C7orf77	1,01

kallikrein related peptidase 14	KLK14	1,01
chordin-like 1	CHRDL1	1,01
homeobox D9	HOXD9	1,01
coiled-coil glutamate rich protein 1	CCER1	1,01
archaelysin family metallopeptidase 1	AMZ1	1,01
mitochondrial ribosomal protein S16	MRPS16	1,01
reprimo-like	RPRML	1,01
CCR4-NOT transcription complex subunit 10	CNOT10	1,01
lipocalin-like 1	LCNL1	1,01
cilia and flagella associated protein 52	CFAP52	1,01
S-antigen; retina and pineal gland (arrestin)	SAG	1,01
CMT1A duplicated region transcript 15-like 2	CDRT15L2	1,01
microtubule associated protein 1 light chain 3 gamma	MAP1LC3C	1,01
BPI fold containing family A, member 1	BPIFA1	1,01
small vasohibin binding protein	SVBP	1,01
general transcription factor IIIC subunit 2	GTF3C2	1,01
von Willebrand factor C domain containing 2	VWC2	1,01
SET and MYND domain containing 1	SMYD1	1,01
ephrin-B3	EFNB3	1,01
G protein-coupled receptor 156	GPR156	1,01
trophoblast glycoprotein-like	TPBGL	1,01
2-oxoglutarate and iron-dependent oxygenase domain containing 1	OGFOD1	1,01
bone morphogenetic protein 3	BMP3	1,01
neuroblastoma breakpoint family, member 26	NBPF26	1,01
ATPase, H+/K+ exchanging, beta polypeptide	ATP4B	1,01
MAGE family member B17	MAGEB17	1,01
DEAD (Asp-Glu-Ala-Asp) box polypeptide 49	DDX49	1,01
arachidonate 12-lipoxygenase	ALOX12	1,01
amyloid beta (A4) precursor protein-binding, family A, member 1	APBA1	1,01
long intergenic non-protein coding RNA 1590; small integral membrane protein 8	LINC01590; SMIM8	1,01
maestro heat-like repeat family member 9	MROH9	1,01
MT-RNR2-like 4	MTRNR2L4	1,01
potassium channel, two pore domain subfamily K, member 10	KCNK10	1,01
PNN-interacting serine/arginine-rich protein	PNISR	1,01
zinc finger with KRAB and SCAN domains 7	ZKSCAN7	1,01
SPT20 homolog, SAGA complex component-like 2	SUPT20HL2	1,01
coiled-coil domain containing 166	CCDC166	1,01
dehydrogenase/reductase (SDR family) member 12	DHRS12	1,01
mitogen-activated protein kinase 15	MAPK15	1,01
calpain 6	CAPN6	1,01
tripartite motif containing 60	TRIM60	1,01

adenylate cyclase 4	ADCY4	1,01
ubiquitin specific peptidase 17-like family member 4	USP17L4	1,01
killer cell lectin-like receptor subfamily D, member 1	KLRD1	1,01
protein tyrosine phosphatase, receptor type, B	PTPRB	1,01
chromosome 1 open reading frame 95	C1orf95	1,01
gamma-glutamyl hydrolase (conjugase, folylpolygammaglutamyl hydrolase)	GGH	1,01
nuclear receptor coactivator 3	NCOA3	1,01
thymic stromal lymphopoietin	TSLP	1,01
JMJD1C antisense RNA 1	JMJD1C-AS1	1,01
regulatory factor X, 3 (influences HLA class II expression)	RFX3	1,01
pre-B lymphocyte 3	VPREB3	1,01
chymotrypsin-like elastase family, member 1	CELA1	1,01
coiled-coil domain containing 17	CCDC17	1,01
chemokine (C-C motif) ligand 4-like 2; chemokine (C-C motif) ligand 4; chemokine (C-C motif) ligand 4-like 1	CCL4L2; CCL4; CCL4L1	1,01
N-acetyltransferase 8B (GCN5-related, putative, gene/pseudogene)	NAT8B	1,01
X-linked Kx blood group related 7	XKR7	1,01
topoisomerase I binding, arginine/serine-rich, E3 ubiquitin protein ligase	TOPORS	1,01
mediator complex subunit 27	MED27	1,01
ADAM metallopeptidase domain 7	ADAM7	1,01
olfactory receptor, family 5, subfamily P, member 2	OR5P2	1,01
cannabinoid receptor interacting protein 1	CNRIP1	1,01
mediator complex subunit 25; microRNA 6800	MED25; MIR6800	1,01
Memczak2013 ANTISENSE, coding, INTERNAL, UTR3 best transcript NM_001428	ENO1	1,01
dual oxidase 2	DUOX2	1,01
chromosome 16 open reading frame 90	C16orf90	1,01
seizure related 6 homolog (mouse)-like 2	SEZ6L2	1,01
PARK2 co-regulated like	PACRGL	1,01
ring finger protein 152	RNF152	1,01
IQ motif containing F6	IQCF6	1,01
chromosome 11 open reading frame 65	C11orf65	1,01
MAGE family member B3	MAGEB3	1,01
zinc finger and BTB domain containing 45	ZBTB45	1,01
SLIT and NTRK-like family, member 5	SLITRK5	1,01
branched chain amino-acid transaminase 2, mitochondrial	BCAT2	1,01
Memczak2013 ANTISENSE, CDS, coding, upstream_start, UTR3, UTR5 best transcript NM_003523	HIST1H2BE	1,01
catenin, beta like 1	CTNNBL1	1,01

lipoma HMGIC fusion partner	LHFP	1,01
synaptosome associated protein 47kDa	SNAP47	1,01
chromosome 9 open reading frame 173	C9orf173	1,01
zinc finger protein 251	ZNF251	1,01
glyoxylate reductase 1 homolog (Arabidopsis)	GLYR1	1,01
chromosome 2 open reading frame 71	C2orf71	1,01
signal recognition particle 54kDa	SRP54	1,01
adhesion G protein-coupled receptor L3	ADGRL3	1,01
zinc finger protein 502	ZNF502	1,01
family with sequence similarity 47, member C	FAM47C	1,01
coagulation factor XIII, A1 polypeptide	F13A1	1,01
serine/threonine kinase-like domain containing 1 [Source:HGNC Symbol;Acc:HGNC:28669]	STKLD1	1,01
disrupted in renal carcinoma 1	DIRC1	1,01
gap junction protein alpha 10	GJA10	1,01
tudor domain containing 12	TDRD12	1,01
RNA binding motif protein 7	RBM7	1,01
olfactory receptor, family 52, subfamily W, member 1	OR52W1	1,01
glutamate receptor, ionotropic, kainate 3	GRIK3	1,01
cancer/testis antigen 55	CT55	1,01
testis expressed 9	TEX9	1,01
Jeck2013 ANTISENSE, coding, INTERNAL, intronic best transcript NM_016263	FZR1	1,01
adenylosuccinate synthase	ADSS	1,01
chromosome 19 open reading frame 44	C19orf44	1,01
tetratricopeptide repeat domain 25	TTC25	1,01
calcium channel flower domain containing 1	CACFD1	1,01
forkhead box P4; microRNA 4641	FOXP4; MIR4641	1,01
purinergic receptor P2X, ligand gated ion channel, 6	P2RX6	1,01
coiled-coil-helix-coiled-coil-helix domain containing 3	CHCHD3	1,01
ribosomal protein S25	RPS25	1,01
LY6/PLAUR domain containing 2	LYPD2	1,01
Jeck2013 ALT_ACCEPTOR, ALT_DONOR, coding, INTERNAL, intronic best transcript NM_152900	MAGI3	1,01
Memczak2013 ANTISENSE, CDS, coding, INTERNAL, intronic best transcript NM_001077401	ACVRL1	1,01
fatty acid amide hydrolase	FAAH	1,01
tRNA-yW synthesizing protein 1 homolog (S. cerevisiae)	TYW1	1,01
spindlin family, member 4	SPIN4	1,01
vitelline membrane outer layer 1 homolog (chicken)	VMO1	1,01
coiled-coil domain containing 102B	CCDC102B	1,01
myosin light chain 1	MYL1	1,01
cysteine-rich secretory protein 3	CRISP3	1,01
cadherin 18, type 2	CDH18	1,01
NOP2/Sun domain family, member 7	NSUN7	1,01

chromosome 11 open reading frame 40	C11orf40	1,01
uncharacterized LOC389199; Transcript Identified by AceView, Entrez Gene ID(s) 389199; novel transcript	LOC389199; AC097381.1	1,01
periaxin	PRX	1,01
topoisomerase (DNA) II binding protein 1	TOPBP1	1,01
lumican	LUM	1,01
transmembrane epididymal protein 1	TEDDM1	1,01
testis and ovary specific PAZ domain containing 1	TOPAZ1	1,01
fumarylacetoacetate hydrolase domain containing 2A	FAHD2A	1,01
HOP homeobox	HOPX	1,01
ubiquitin-conjugating enzyme E2U (putative)	UBE2U	1,01
zinc finger, BED-type containing 5	ZBED5	1,01
La ribonucleoprotein domain family, member 1	LARP1	1,01
Yes-associated protein 1	YAP1	1,01
olfactory receptor, family 2, subfamily B, member 3	OR2B3	1,01
solute carrier family 34 (type II sodium/phosphate cotransporter), member 1	SLC34A1	1,01
glycine-N-acyltransferase-like 1	GLYATL1	1,01
transient receptor potential cation channel, subfamily M, member 7	TRPM7	1,01
G1 to S phase transition 1	GSPT1	1,01
nucleolar protein 4	NOL4	1,01
harbinger transposase derived 1	HARBI1	1,01
carboxymethylenebutenolidase homolog (Pseudomonas)	CMBL	1,01
GVQW motif containing 2	GVQW2	1,01
very low density lipoprotein receptor	VLDLR	1,01
Transcript Identified by AceView, Entrez Gene ID(s) 23126	POGZ	1,01
coiled-coil domain containing 18	CCDC18	1,01
stearoyl-CoA desaturase 5	SCD5	1,01
dynein, axonemal, heavy chain 2	DNAH2	1,01
zinc finger protein 691	ZNF691	1,01
polo-like kinase 5	PLK5	1,01
BTG3 associated nuclear protein	BANP	1,01
Zhang2013 ALT_ACCEPTOR, ALT_DONOR, coding, INTERNAL, intronic best transcript NM_052909	PLEKHG4B	1,01
LINE-1 type transposase domain containing 1	L1TD1	1,01
engulfment and cell motility 3	ELMO3	1,01
zinc finger protein 544	ZNF544	1,01
Transcript Identified by AceView, Entrez Gene ID(s) 84954; novel transcript, antisense to STAP2	AC007292.7; MPND	1,01
protein tyrosine phosphatase, receptor type, f polypeptide (PTPRF), interacting protein (liprin), alpha 2	PPFIA2	1,01
cyclin-dependent kinase 2 associated protein 2	CDK2AP2	1,00

poliovirus receptor related immunoglobulin domain containing	PVRIG	1,00
WW domain binding protein 1	WBP1	1,00
olfactory receptor, family 11, subfamily H, member 2	OR11H2	1,00
chromosome 9 open reading frame 106	C9orf106	1,00
neurexophilin and PC-esterase domain family, member 4	NXPE4	1,00
proline rich 23B	PRR23B	1,00
sodium channel, non voltage gated 1 beta subunit	SCNN1B	1,00
receptor (TNFRSF)-interacting serine-threonine kinase 1	RIPK1	1,00
olfactory receptor, family 10, subfamily G, member 2	OR10G2	1,00
keratin associated protein 9-3	KRTAP9-3	1,00
solute carrier family 5 (sodium/sugar cotransporter), member 10	SLC5A10	1,00
solute carrier family 16 (monocarboxylate transporter), member 1	SLC16A1	1,00
small nuclear ribonucleoprotein polypeptide B	SNRPB2	1,00
arrestin domain containing 5	ARRDC5	1,00
olfactory receptor, family 2, subfamily D, member 2	OR2D2	1,00
SMAD family member 5	SMAD5	1,00
DnaJ (Hsp40) homolog, subfamily B, member 13	DNAJB13	1,00
guanylate binding protein 2, interferon-inducible	GBP2	1,00
lysozyme-like 2	LYZL2	1,00
AF4/FMR2 family, member 3	AFF3	1,00
transmembrane protein 155	TMEM155	1,00
transmembrane protein 115	TMEM115	1,00
inositol polyphosphate-5-phosphatase D	INPP5D	1,00
DIM1 dimethyladenosine transferase 1 homolog	DIMT1	1,00
receptor (chemosensory) transporter protein 2	RTP2	1,00
tumor protein p53 regulated apoptosis inducing protein 1	TP53AIP1	1,00
Transcript Identified by AceView, Entrez Gene ID(s) 9045	RPL14	1,00
leucine rich repeat and fibronectin type III domain containing 1	LRFN1	1,00
ISY1-RAB43 readthrough	ISY1-RAB43	1,00
sterile alpha motif domain containing 11	SAMD11	1,00
Transcript Identified by AceView, Entrez Gene ID(s) 51752	ERAP1	1,00
zinc finger protein 195	ZNF195	1,00
parkin RBR E3 ubiquitin protein ligase	PARK2	1,00
glutamate rich 4	ERICH4	1,00
somatostatin receptor 5	SSTR5	1,00
YTH N(6)-methyladenosine RNA binding protein 2	YTHDF2	1,00
TRAF family member-associated NFKB activator	TANK	1,00

lectin, galactoside-binding, soluble, 13	LGALS13	1,00
cilia and flagella associated protein 69	CFAP69	1,00
Memczak2013 ANTISENSE, coding, INTERNAL, UTR3 best transcript NM_005188	CBL	1,00
olfactory receptor, family 6, subfamily Q, member 1 (gene/pseudogene)	OR6Q1	1,00
chromosome 10 open reading frame 95	C10orf95	1,00
olfactory receptor, family 1, subfamily D, member 5	OR1D5	1,00
ILK associated serine/threonine phosphatase	ILKAP	1,00
diacylglycerol kinase, delta 130kDa	DGKD	1,00
serine/threonine kinase-like domain containing 1 [Source:HGNC Symbol;Acc:HGNC:28669]	STKLD1	1,00
NME/NM23 family member 5	NME5	1,00
chondroitin sulfate proteoglycan 4 pseudogene 1, Y-linked	CSPG4P1Y	1,00
CTR9 homolog, Paf1/RNA polymerase II complex component	CTR9	1,00
tumor protein p63 regulated 1	TPRG1	1,00
ADAM metallopeptidase with thrombospondin type 1 motif 15	ADAMTS15	1,00
hydroxysteroid (17-beta) dehydrogenase 1	HSD17B1	1,00
chymotrypsin-like elastase family, member 3B	CELA3B	1,00
TNF receptor-associated factor 3 interacting protein 1	TRAF3IP1	1,00
acrosin binding protein	ACRBP	1,00
Memczak2013 ALT_ACCEPTOR, ALT_DONOR, coding, INTERNAL, intronic best transcript NM_001172639	CELF1	1,00
transcription factor 3	TCF3	1,00
absent in melanoma 1-like	AIM1L	1,00
zinc finger protein 716	ZNF716	1,00
heterogeneous nuclear ribonucleoprotein C (C1/C2)	HNRNPC	1,00
olfactory receptor, family 2, subfamily AG, member 1 (gene/pseudogene)	OR2AG1	1,00
CLK4-associating serine/arginine rich protein	CLASRP	1,00
zinc finger protein 175	ZNF175	1,00
S100 calcium binding protein A7A	S100A7A	1,00
NANOG neighbor homeobox	NANOGNB	1,00
sphingosine-1-phosphate receptor 2	S1PR2	1,00
family with sequence similarity 132, member B	FAM132B	1,00
HRAS-like suppressor 2	HRASLS2	1,00
DEAD (Asp-Glu-Ala-Asp) box polypeptide 43	DDX43	1,00
zinc finger, DHHC-type containing 3	ZDHHC3	1,00
solute carrier family 43, member 3	SLC43A3	1,00
ribosomal protein L38	RPL38	1,00
interleukin 1 receptor-like 2	IL1RL2	1,00
visual system homeobox 2	VSX2	1,00

coiled-coil domain containing 28B	CCDC28B	1,00
long intergenic non-protein coding RNA 272	LINC00272	1,00
Transcript Identified by AceView, Entrez Gene ID(s) 8220	DGCR14	1,00
mitochondrial ribosomal protein S28	MRPS28	1,00
solute carrier family 25, member 41	SLC25A41	1,00
claudin 9	CLDN9	1,00
Transcript Identified by AceView, Entrez Gene ID(s) 11193	WBP4	1,00
Transcript Identified by AceView, Entrez Gene ID(s) 153572	IRX2	1,00
intraflagellar transport 52	IFT52	1,00
kinesin family member 26B	KIF26B	1,00
BTB (POZ) domain containing 16	BTBD16	1,00
interleukin 11 receptor, alpha	IL11RA	1,00
DEAH (Asp-Glu-Ala-His) box polypeptide 40	DHX40	1,00
mitochondrial ribosomal protein S23	MRPS23	1,00
integrin beta 1 binding protein 1	ITGB1BP1	1,00
chymotrypsin-like	CTRL	1,00
endogenous Bornavirus-like nucleoprotein 2	EBLN2	1,00
BPI fold containing family A, member 2	BPIFA2	1,00
defensin, alpha 1B; defensin, alpha 1; defensin, alpha 3, neutrophil-specific	DEFA1B; DEFA1; DEFA3	1,00
defensin, alpha 1; defensin, alpha 1B	DEFA1; DEFA1B	1,00
carnosine synthase 1	CARNS1	1,00
zinc finger protein 91	ZNF91	1,00
purinergic receptor P2Y, G-protein coupled, 14	P2RY14	1,00
general transcription factor IIA 1-like; STON1-GTF2A1L readthrough; stonin 1	GTF2A1L; STON1-GTF2A1L; STON1	1,00
immunoresponsive 1 homolog (mouse)	IRG1	1,00
neuropeptides B/W receptor 2	NPBWR2	1,00
fibroblast growth factor 23	FGF23	1,00
EF-hand calcium binding domain 8	EFCAB8	1,00
ATP binding cassette subfamily B member 1	ABCB1	1,00
PIK3CD antisense RNA 2	PIK3CD-AS2	1,00
tumor necrosis factor (ligand) superfamily, member 11	TNFSF11	1,00
laminin, gamma 3	LAMC3	1,00
forkhead box E1	FOXE1	1,00
toll-like receptor 7	TLR7	1,00
pleckstrin and Sec7 domain containing	PSD	1,00
hydroxymethylbilane synthase	HMBS	1,00
valyl-tRNA synthetase	VARS	1,00
IGF like family receptor 1	IGFLR1	1,00
solute carrier family 4, sodium bicarbonate transporter, member 10	SLC4A10	1,00

dynamin 3	DNM3	1,00
TOR signaling pathway regulator	TIPRL	1,00
mitochondrial transcription termination factor 4	MTERF4	1,00
relaxin/insulin-like family peptide receptor 1	RXFP1	1,00
NAD kinase	NADK	1,00
heterogeneous nuclear ribonucleoprotein C-like 3 [Source:HGNC Symbol;Acc:HGNC:51235]	HNRNPCL3	1,00
leucine rich repeat transmembrane neuronal 3	LRRTM3	1,00
adhesion G protein-coupled receptor A1	ADGRA1	1,00
major histocompatibility complex, class II, DR beta 1	HLA-DRB1	1,00
MAGE family member C1	MAGEC1	1,00
methyltransferase like 18	METTL18	1,00
EPH receptor B1	EPHB1	1,00
matrix metallopeptidase 27	MMP27	1,00
clarin 2	CLRN2	1,00
RNA binding motif protein 15B	RBM15B	1,00
IQ motif containing F5	IQCF5	1,00
cardiomyopathy associated 5	CMYA5	1,00
NK2 homeobox 8	NKX2-8	1,00
LYR motif containing 9	LYRM9	1,00
family with sequence similarity 129, member C	FAM129C	1,00
ADP-ribosylation factor 1; microRNA 3620	ARF1; MIR3620	1,00
listerin E3 ubiquitin protein ligase 1	LTN1	1,00
cartilage intermediate layer protein 2	CILP2	1,00
aldo-keto reductase family 1, member C2	AKR1C2	1,00
protein phosphatase 6, regulatory subunit 3	PPP6R3	1,00
testis expressed 12	TEX12	1,00
Transcript Identified by AceView, Entrez Gene ID(s) 91782	CHMP7	1,00
olfactory receptor, family 52, subfamily B, member 2	OR52B2	1,00
defensin, beta 107B; defensin, beta 107A	DEFB107B; DEFB107A	1,00
mutL homolog 3	MLH3	1,00
TBC1 domain family, member 3L; TBC1 domain family, member 3E	TBC1D3L; TBC1D3E	1,00
guanylate binding protein 4	GBP4	1,00
endonuclease/exonuclease/phosphatase family domain containing 1	EEPD1	1,00
testis expressed 40	TEX40	1,00
LGALS8 antisense RNA 1	LGALS8-AS1	1,00
CD6 molecule	CD6	1,00
maestro	MRO	1,00
guanylate binding protein family, member 6	GBP6	1,00
EDAR-associated death domain	EDARADD	1,00
chondroitin sulfate proteoglycan 5 (neuroglycan C)	CSPG5	1,00

RAB6C-like; Synthetic construct Homo sapiens clone IMAGE:100016523, MGC:184337 RAB6C-like (LOC150786) mRNA, encodes complete protein.	WTH3DI; LOC150786	1,00
glycine receptor beta	GLRB	1,00
forkhead box F2	FOXF2	1,00
chemokine (C-C motif) receptor 8	CCR8	1,00
peptidyl arginine deiminase, type IV	PADI4	1,00
pleckstrin homology domain interacting protein	PHIP	1,00
methyltransferase like 16	METTL16	1,00
Jeck2013 ALT_ACCEPTOR, ALT_DONOR, coding, INTERNAL, intronic best transcript NM_002737	PRKCA	1,00
prickle homolog 3	PRICKLE3	1,00
uncharacterized LOC400553; novel transcript	LOC400553; RP11-863P13.4	1,00
natural killer cell granule protein 7	NKG7	1,00
nucleoredoxin-like 1	NXNL1	1,00
family with sequence similarity 111, member B	FAM111B	1,00
MORC family CW-type zinc finger 1	MORC1	1,00
potassium channel, two pore domain subfamily K, member 4	KCNK4	1,00
phosphatidylinositol glycan anchor biosynthesis class W	PIGW	1,00
LSM2 homolog, U6 small nuclear RNA and mRNA degradation associated	LSM2	1,00
cilia and flagella associated protein 206	CFAP206	1,00
defensin, beta 109, pseudogene 1B	DEFB109P1B	1,00
zinc finger, DHHC-type containing 3	ZDHHC3	1,00
synaptotagmin VI	SYT6	1,00
limbic system-associated membrane protein	LSAMP	1,00
NADPH oxidase, EF-hand calcium binding domain 5; sperm equatorial segment protein 1	NOX5; SPESP1	1,00
TBC1 domain containing kinase	TBCK	1,00
leucine rich repeat containing 63	LRRC63	1,00
pre-B-cell leukemia homeobox 1	PBX1	1,00
DMRT-like family A2	DMRTA2	1,00
K(lysine) acetyltransferase 6A	KAT6A	1,00
zinc finger protein 708	ZNF708	1,00
midline 2	MID2	1,00
Transcript Identified by AceView, Entrez Gene ID(s) 123016	TTC8	1,00
keratin associated protein 15-1	KRTAP15-1	1,00
mediator complex subunit 19	MED19	1,00
activating transcription factor 1	ATF1	1,00
major facilitator superfamily domain containing 2B	MFSD2B	1,00
testis expressed 101	TEX101	1,00
claudin 17	CLDN17	1,00

kelch-like family member 41	KLHL41	1,00
esterase D	ESD	1,00
Zhang2013 ALT_DONOR, coding, INTERNAL, intronic best transcript NM_001006	RPS3A	1,00
chromosome 10 open reading frame 142	C10orf142	1,00
ABL proto-oncogene 1, non-receptor tyrosine kinase	ABL1	1,00
paraneoplastic Ma antigen family member 6A	PNMA6A	1,00
chloride channel, voltage-sensitive 1	CLCN1	1,00
transmembrane protein 125	TMEM125	1,00
GTP-binding protein 8 (putative)	GTPBP8	1,00
testis specific protein, Y-linked 10	TSPY10	1,00
A kinase (PRKA) anchor protein 4	AKAP4	1,00
sarcoglycan delta	SGCD	1,00
Memczak2013 ANTISENSE, CDS, coding, INTERNAL best transcript NM_020979	SH2B2	1,00
cytochrome P450, family 4, subfamily B, polypeptide 1	CYP4B1	1,00
Transcript Identified by AceView, Entrez Gene ID(s) 11273	ATXN2L	1,00
lipocalin 10	LCN10	1,00
leucine rich repeat containing 3C	LRRC3C	1,00
zinc finger, CW type with PWWP domain 1	ZCWPW1	1,00
homeobox A4	HOXA4	1,00
fibronectin type III and ankyrin repeat domains 1	FANK1	1,00
Memczak2013 ALT_ACCEPTOR, ALT_DONOR, coding, INTERNAL, intronic best transcript NM_015130	TBC1D9	1,00
olfactory receptor, family 52, subfamily E, member 8	OR52E8	1,00
golgi-associated, olfactory signaling regulator	GFY	1,00
Transcript Identified by AceView, Entrez Gene ID(s) 5992	RFX4	1,00
myomesin 2	MYOM2	1,00
tetratricopeptide repeat domain 16	TTC16	1,00
peptidase M20 domain containing 1	PM20D1	1,00
mitogen-activated protein kinase kinase kinase 15	MAP3K15	1,00
HBS1-like translational GTPase	HBS1L	1,00
NK6 homeobox 3	NKX6-3	1,00
olfactory receptor, family 1, subfamily D, member 2	OR1D2	1,00
chromodomain helicase DNA binding protein 5	CHD5	1,00
Memczak2013 ANTISENSE, CDS, coding, INTERNAL best transcript NM_002265	KPNB1	1,00
inositol polyphosphate-5-phosphatase B	INPP5B	1,00
long intergenic non-protein coding RNA 311	LINC00311	1,00
retinoic acid receptor responder (tazarotene induced) 3	RARRES3	1,00
methionine adenosyltransferase II, beta	MAT2B	1,00
WD repeat domain 49	WDR49	1,00
prostate and testis expressed 2	PATE2	1,00

leucine rich repeat containing 40	LRRC40	1,00
succinate receptor 1	SUCNR1	1,00
tRNA methyltransferase 13 homolog (S. cerevisiae)	TRMT13	1,00
ADP-ribosylation factor like GTPase 10	ARL10	1,00
ciliogenesis associated TTC17 interacting protein	CATIP	1,00
nicotinamide nucleotide transhydrogenase	NNT	1,00
HERV-H LTR-associating 1	HHLA1	1,00
RAB11 family interacting protein 2 (class I)	RAB11FIP2	1,00
Transcript Identified by AceView, Entrez Gene ID(s) 79677	SMC6	1,00
acyl-CoA binding domain containing 6	ACBD6	1,00
TIMELESS interacting protein; ribosomal protein L9 pseudogene 25	TIPIN; RPL9P25	1,00
catenin (cadherin-associated protein), alpha 2	CTNNA2	1,00
IKAROS family zinc finger 4	IKZF4	1,00
AhpC/TSA antioxidant enzyme domain containing 1	AAED1	1,00
Purkinje cell protein 4	PCP4	1,00
complement factor H	CFH	1,00
ribosomal protein L41	RPL41	1,00
family with sequence similarity 179, member A	FAM179A	1,00
keratin 4, type II	KRT4	1,00
metastasis associated in colon cancer 1	MACC1	1,00
G protein-coupled receptor 85	GPR85	1,00
DAN domain family member 5, BMP antagonist	DAND5	1,00
tripartite motif containing 46	TRIM46	1,00
tigger transposable element derived 5	TIGD5	1,00
small proline-rich protein 1B	SPRR1B	1,00
phospholipid phosphatase 7 (inactive)	PLPP7	1,00
ATP binding cassette subfamily A member 5	ABCA5	1,00
calcium channel, voltage-dependent, gamma subunit 4	CACNG4	1,00
ribulose-5-phosphate-3-epimerase	RPE	1,00
Memczak2013 ALT_ACCEPTOR, ALT_DONOR, coding, INTERNAL, intronic best transcript NM_017931	TTC38	1,00
coiled-coil domain containing 149	CCDC149	1,00
contactin associated protein-like 4	CNTNAP4	1,00
polycystic kidney disease 2-like 2	PKD2L2	1,00
KIAA1210	KIAA1210	1,00
EF-hand and coiled-coil domain containing 1	EFCC1	1,00
neuroblastoma RAS viral (v-ras) oncogene homolog	NRAS	1,00
gamma-aminobutyric acid (GABA) A receptor, gamma 2	GABRG2	1,00
suppressor APC domain containing 2	SAPCD2	1,00
neurotrophic tyrosine kinase, receptor, type 1	NTRK1	1,00
Transcript Identified by AceView, Entrez Gene ID(s) 5604	MAP2K1	1,00

hypocretin (orexin) receptor 1	HCRTR1	1,00
LEM domain containing 1	LEMD1	1,00
follicle stimulating hormone receptor	FSHR	1,00
peptidyl arginine deiminase, type VI	PADI6	1,00
olfactory receptor, family 4, subfamily S, member 2	OR4S2	1,00
Memczak2013 ANTISENSE, CDS, coding, INTERNAL best transcript NM_014931	PPP6R1	1,00
SRY box 30	SOX30	1,00
Transcript Identified by AceView, Entrez Gene ID(s) 150590; 51601; 51263; chromosome 2 open reading frame 15 [Source:HGNC Symbol;Acc:HGNC:28436]	C2orf15andLIPT1andMRPL30; C2orf15	1,00
immunoglobulin superfamily, member 6	IGSF6	1,00
crystallin beta B1	CRYBB1	1,00
long intergenic non-protein coding RNA 303	LINC00303	1,00
notch 1	NOTCH1	1,00
riboflavin kinase	RFK	1,00
ADAM metallopeptidase domain 23	ADAM23	1,00
MKRN2 opposite strand	MKRN2OS	1,00
folliculogenesis specific bHLH transcription factor	FIGLA	1,00
serine peptidase inhibitor, Kazal type 6	SPINK6	1,00
diacylglycerol kinase, beta 90kDa	DGKB	1,00
folate receptor 2 (fetal)	FOLR2	1,00
K(lysine) acetyltransferase 6B	KAT6B	1,00
cytochrome P450, family 2, subfamily D, polypeptide 6	CYP2D6	1,00
UTP20 small subunit (SSU) processome component	UTP20	1,00
protein kinase, Y-linked, pseudogene	PRKY	1,00
olfactory receptor, family 11, subfamily H, member 1	OR11H1	1,00
coiled-coil domain containing 54	CCDC54	1,00
chemokine (C-X-C motif) ligand 2	CXCL2	1,00
keratin 2, type II	KRT2	1,00
olfactory receptor, family 10, subfamily J, member 1	OR10J1	1,00
POU class 6 homeobox 2	POU6F2	1,00
OTU deubiquitinase 5	OTUD5	1,00
olfactory receptor, family 51, subfamily I, member 1	OR51I1	1,00
Sjogren syndrome nuclear autoantigen 1	SSNA1	1,00
canopy FGF signaling regulator 1	CNPY1	1,00
transmembrane protein 174	TMEM174	1,00
deltex 2, E3 ubiquitin ligase	DTX2	1,00
cytochrome P450, family 4, subfamily A, polypeptide 11	CYP4A11	1,00
TGFB-induced factor homeobox 2-like, Y-linked	TGIF2LY	1,00
dual specificity phosphatase and pro isomerase domain containing 1	DUPD1	1,00
dehydrogenase/reductase (SDR family) member 7B	DHRS7B	1,00
lysophosphatidic acid receptor 3	LPAR3	1,00

potassium channel, two pore domain subfamily K, member 15	KCNK15	1,00
NADH dehydrogenase (ubiquinone) 1 alpha subcomplex, 9, 39kDa	NDUFA9	1,00
plasmolipin	PLLP	1,00
LMBR1 domain containing 1	LMBRD1	1,00
orthodenticle homeobox 2	OTX2	1,00
gremlin 1, DAN family BMP antagonist [Source:HGNC Symbol;Acc:HGNC:2001]	GREM1	1,00
tumor necrosis factor (ligand) superfamily, member 12	TNFSF12	1,00
SV2 related protein	SVOP	1,00
cell cycle and apoptosis regulator 2	CCAR2	1,00
nuclear mitotic apparatus protein 1	NUMA1	1,00
solute carrier family 27 (fatty acid transporter), member 1	SLC27A1	1,00
Kruppel-like factor 4 (gut)	KLF4	1,00
NUT family member 2F	NUTM2F	1,00
WSC domain containing 2	WSCD2	1,00
TATA box binding protein associated factor 15	TAF15	1,00
olfactory receptor, family 8, subfamily H, member 1	OR8H1	1,00
piggyBac transposable element derived 4	PGBD4	1,00
wingless-type MMTV integration site family, member 11	WNT11	1,00
transcription elongation factor A (SII) N-terminal and central domain containing	TCEANC	1,00
REX1, RNA exonuclease 1 homolog-like 10, pseudogene	REXO1L10P	1,00
interleukin 37	IL37	1,00
reticulon 4 interacting protein 1	RTN4IP1	1,00
acyl-CoA binding domain containing 5	ACBD5	1,00
zinc finger and BTB domain containing 6	ZBTB6	1,00
HRAS-like suppressor family, member 5	HRASLS5	1,00
zinc finger protein 321, pseudogene	ZNF321P	1,00
chromosome 2 open reading frame 70	C2orf70	1,00
casein beta	CSN2	1,00
zinc finger protein 677	ZNF677	1,00
T-box 15	TBX15	1,00
phospholipase D family, member 5	PLD5	1,00
CD164 sialomucin-like 2	CD164L2	1,00
transcription factor EC	TFEC	1,00
huntingtin-associated protein 1	HAP1	1,00
transcription elongation regulator 1	TCERG1	1,00
interleukin 4	IL4	1,00
adrenoceptor alpha 1A	ADRA1A	1,00
leucine rich repeat containing 7	LRRC7	1,00

Memczak2013 ALT_DONOR, coding, INTERNAL, intronic best transcript NM_021078	KAT2A	1,00
meiosis specific with coiled-coil domain	MEIOC	1,00
leucine rich repeat containing 53	LRRC53	1,00
SPHK1 interactor, AKAP domain containing	SPHKAP	1,00
hedgehog acyltransferase-like	HHATL	1,00
long intergenic non-protein coding RNA 961	LINC00961	1,00
poly(ADP-ribose) polymerase family member 6	PARP6	1,00
MAGE family member A10	MAGEA10	1,00
golgin A6 family-like 9	GOLGA6L9	0,99
serine peptidase inhibitor, Kazal type 9	SPINK9	0,99
defensin, beta 130	DEFB130	0,99
defensin, beta 130	DEFB130	0,99
GRIK1 antisense RNA 2; BTB and CNC homology 1, basic leucine zipper transcription factor 1	GRIK1-AS2; BACH1	0,99
complement factor H-related 4	CFHR4	0,99
DnaJ (Hsp40) homolog, subfamily B, member 14	DNAJB14	0,99
diacylglycerol kinase, delta 130kDa	DGKD	0,99
selenoprotein K; selenoprotein K [Source:EntrezGene;Acc:58515]; Transcript Identified by AceView, Entrez Gene ID(s) 58515, RefSeq ID(s) NM_021237	SELK	0,99
Janus kinase and microtubule interacting protein 3	JAKMIP3	0,99
chromosome 7 open reading frame 72	C7orf72	0,99
Na+/K+ transporting ATPase interacting 1	NKAIN1	0,99
non-SMC condensin I complex subunit G	NCAPG	0,99
NADH dehydrogenase (ubiquinone) 1 alpha subcomplex, 10, 42kDa	NDUFA10	0,99
arylsulfatase family, member H	ARSH	0,99
apelin receptor early endogenous ligand	APELA	0,99
Kv channel interacting protein 1	KCNIP1	0,99
erythrocyte membrane protein band 4.1 like 4A	EPB41L4A	0,99
otopetrin 2	OTOP2	0,99
angiopoietin like 4	ANGPTL4	0,99
phosphodiesterase 2A, cGMP-stimulated	PDE2A	0,99
zinc finger, matrin-type 1	ZMAT1	0,99
StAR-related lipid transfer domain containing 6	STARD6	0,99
zinc finger protein 653	ZNF653	0,99
transmembrane protein 30B	TMEM30B	0,99
arrestin 3, retinal (X-arrestin)	ARR3	0,99
queuine tRNA-ribosyltransferase 1	QTRT1	0,99
collagen, type XIII, alpha 1	COL13A1	0,99
DENN/MADD domain containing 4A	DENND4A	0,99
Nance-Horan syndrome (congenital cataracts and dental anomalies)	NHS	0,99

coiled-coil domain containing 115	CCDC115	0,99
chromosome 7 open reading frame 13	C7orf13	0,99
adrenomedullin 5 (putative); carnitine palmitoyltransferase 1C	ADM5; CPT1C	0,99
fucosyltransferase 7 (alpha (1,3) fucosyltransferase)	FUT7	0,99
family with sequence similarity 127, member B	FAM127B	0,99
apolipoprotein A-IV	APOA4	0,99
BCL2-like 11 (apoptosis facilitator)	BCL2L11	0,99
FER1L6 antisense RNA 1	FER1L6-AS1	0,99
GDNF antisense RNA 1 (head to head)	GDNF-AS1	0,99
fucosyltransferase 2 (secretor status included)	FUT2	0,99
lamin tail domain containing 2	LMNTD2	0,99
neural cell adhesion molecule 2	NCAM2	0,99
synaptosome associated protein 29kDa	SNAP29	0,99
cytosolic thiouridylase subunit 1	CTU1	0,99
coiled-coil domain containing 91	CCDC91	0,99
cyclin I	CCNI	0,99
leucine rich repeat containing 43	LRRC43	0,99
Transcript Identified by AceView, Entrez Gene ID(s) 257415	FAM133B	0,99
GTP-binding protein 10 (putative)	GTPBP10	0,99
WD repeat domain, phosphoinositide interacting 2	WIPI2	0,99
DnaJ (Hsp40) homolog, subfamily C, member 10	DNAJC10	0,99
synaptonemal complex central element protein 1; shadow of prion protein homolog (zebrafish) pseudogene 1	SYCE1; SPRNP1	0,99
eukaryotic translation initiation factor 3, subunit C; eukaryotic translation initiation factor 3, subunit C-like	EIF3C; EIF3CL	0,99
engrailed homeobox 1	EN1	0,99
TAO kinase 1; microRNA 4523	TAOK1; MIR4523	0,99
secretoglobin, family 1A, member 1 (uteroglobin)	SCGB1A1	0,99
prostaglandin F receptor (FP)	PTGFR	0,99
olfactory receptor, family 4, subfamily D, member 11	OR4D11	0,99
enhancer of yellow 2 homolog (Drosophila)	ENY2	0,99
astrotactin 2	ASTN2	0,99
N-glycanase 1	NGLY1	0,99
muscle, skeletal, receptor tyrosine kinase	MUSK	0,99
mesenchyme homeobox 1	MEOX1	0,99
olfactory receptor, family 10, subfamily J, member 3	OR10J3	0,99
trafficking protein particle complex 3	TRAPPC3	0,99
PRAME family member 8	PRAMEF8	0,99
chemokine-like factor; CKLF-CMTM1 readthrough	CKLF; CKLF-CMTM1	0,99
KIAA1468	KIAA1468	0,99
chromosome 1 open reading frame 87	C1orf87	0,99

bone morphogenetic protein/retinoic acid inducible neural-specific 3	BRINP3	0,99
zinc finger and BTB domain containing 37	ZBTB37	0,99
endo/exonuclease (5-3), endonuclease G-like	EXOG	0,99
transglutaminase 4	TGM4	0,99
ribosomal protein S6 kinase, 90kDa, polypeptide 1	RPS6KA1	0,99
interleukin 21 receptor	IL21R	0,99
ADAMTS like 4	ADAMTSL4	0,99
Eukaryotic translation initiation factor 3 subunit F [Source:UniProtKB/Swiss-Prot;Acc:O00303]	EIF3F	0,99
Jeck2013 ALT_ACCEPTOR, ALT_DONOR, coding, INTERNAL, intronic best transcript NM_134261	RORA	0,99
ZNF625-ZNF20 readthrough (NMD candidate); zinc finger protein 625	ZNF625-ZNF20; ZNF625	0,99
guanylate cyclase activator 1B (retina)	GUCA1B	0,99
potassium channel tetramerization domain containing 11	KCTD11	0,99
Transcript Identified by AceView, Entrez Gene ID(s) 342184	FMN1	0,99
copine IV	CPNE4	0,99
taste receptor, type 1, member 3	TAS1R3	0,99
retrotransposon gag domain containing 4	RGAG4	0,99
peroxidasin like	PXDNL	0,99
5-hydroxytryptamine (serotonin) receptor 1E, G protein-coupled	HTR1E	0,99
zinc finger family member 767, pseudogene	ZNF767P	0,99
zinc finger protein 141	ZNF141	0,99
copine VI (neuronal)	CPNE6	0,99
chromosome 2 open reading frame 66	C2orf66	0,99
cilia and flagella associated 54	CFAP54	0,99
TM4SF1 antisense RNA 1	TM4SF1-AS1	0,99
ectonucleotide pyrophosphatase/phosphodiesterase 2	ENPP2	0,99
choline kinase beta	CHKB	0,99
tubulin, epsilon 1	TUBE1	0,99
ras homolog family member H	RHOH	0,99
KIAA0125	KIAA0125	0,99
Obg-like ATPase 1	OLA1	0,99
DnaJ (Hsp40) homolog, subfamily B, member 8	DNAJB8	0,99
methyltransferase like 16	METTL16	0,99
olfactory receptor, family 52, subfamily N, member 1	OR52N1	0,99
family with sequence similarity 65, member C	FAM65C	0,99
nuclear speckle splicing regulatory protein 1; microRNA 423	NSRP1; MIR423	0,99
artemin	ARTN	0,99
small ubiquitin-like modifier 1	SUMO1	0,99

olfactory receptor, family 11, subfamily H, member 4	OR11H4	0,99
TBC1 domain family, member 20	TBC1D20	0,99
AHNAK nucleoprotein	AHNAK	0,99
matrix Gla protein	MGP	0,99
S-antigen; retina and pineal gland (arrestin)	SAG	0,99
membrane protein, palmitoylated 4	MPP4	0,99
SNAP-associated protein	SNAPIN	0,99
tripartite motif containing 43B	TRIM43B	0,99
WNK lysine deficient protein kinase 1	WNK1	0,99
transmembrane protein 164	TMEM164	0,99
proteasome (prosome, macropain) assembly chaperone 1	PSMG1	0,99
activin A receptor type IB	ACVR1B	0,99
coiled-coil domain containing 66	CCDC66	0,99
zinc finger, SWIM-type containing 4	ZSWIM4	0,99
protein phosphatase 1, regulatory subunit 16A	PPP1R16A	0,99
zinc finger protein 846	ZNF846	0,99
proline-rich protein BstNI subfamily 1	PRB1	0,99
transmembrane protein 179	TMEM179	0,99
dickkopf-like 1	DKKL1	0,99
amyloid beta (A4) precursor protein-binding, family B, member 1 (Fe65)	APBB1	0,99
Transcript Identified by AceView, Entrez Gene ID(s) 116442	RAB39B	0,99
small nuclear ribonucleoprotein polypeptide A	SNRPA	0,99
RAR-related orphan receptor B	RORB	0,99
olfactory receptor, family 52, subfamily H, member 1	OR52H1	0,99
NLR family, CARD domain containing 3	NLRC3	0,99
solute carrier family 6 (neutral amino acid transporter), member 17	SLC6A17	0,99
SPATA31 subfamily A, member 5; SPATA31 subfamily A, member 7	SPATA31A5; SPATA31A7	0,99
chromosome 10 open reading frame 67	C10orf67	0,99
Memczak2013 ALT_ACCEPTOR, ALT_DONOR, coding, INTERNAL, intronic best transcript NM_016836	RBMS1	0,99
T cell activation inhibitor, mitochondrial	TCAIM	0,99
transmembrane protease, serine 11E	TMPRSS11E	0,99
	LRIG1	0,99
enoyl-CoA hydratase domain containing 3	ECHDC3	0,99
PHD finger protein 24	PHF24	0,99
receptor (chemosensory) transporter protein 3	RTP3	0,99
NADH dehydrogenase (ubiquinone) 1, alpha/beta subcomplex, 1, 8kDa	NDUFAB1	0,99
G protein-coupled receptor 37 (endothelin receptor type B-like)	GPR37	0,99

major intrinsic protein of lens fiber	MIP	0,99
mitogen-activated protein kinase kinase 3	MAP2K3	0,99
DEK proto-oncogene	DEK	0,99
derlin 2	DERL2	0,99
diencephalon/mesencephalon homeobox 1	DMBX1	0,99
ubiquilin-like	UBQLNL	0,99
tetraspanin 19	TSPAN19	0,99
large tumor suppressor kinase 1	LATS1	0,99
peptidylprolyl isomerase A (cyclophilin A) pseudogene 30	PPIAP30	0,99
IKAROS family zinc finger 3	IKZF3	0,99
engrailed homeobox 2	EN2	0,99
keratin associated protein 6-1	KRTAP6-1	0,99
methyltransferase like 2B	METTL2B	0,99
interferon induced transmembrane protein 10	IFITM10	0,99
ubiquitin B	UBB	0,99
uncharacterized LOC103611081; uncharacterized LOC100507431; Transcript Identified by AceView; novel transcript	LOC103611081; LOC100507431; RP11-890B15.3; RP11-890B15.2; klerglaw	0,99
DEAH (Asp-Glu-Ala-His) box polypeptide 37	DHX37	0,99
G protein-coupled receptor 139	GPR139	0,99
chloride channel accessory 4	CLCA4	0,99
chromosome 22 open reading frame 23	C22orf23	0,99
zinc finger protein 80	ZNF80	0,99
glycoprotein M6B	GPM6B	0,99
chromosome 4 open reading frame 47	C4orf47	0,99
cAMP responsive element binding protein 3-like 4	CREB3L4	0,99
factor interacting with PAPOLA and CPSF1	FIP1L1	0,99
olfactory receptor, family 5, subfamily P, member 2	OR5P2	0,99
kelch repeat and BTB (POZ) domain containing 3	KBTBD3	0,99
mal, T-cell differentiation protein-like	MALL	0,99
adhesion G protein-coupled receptor F1	ADGRF1	0,99
myosin, heavy chain 11, smooth muscle	MYH11	0,99
transmembrane BAX inhibitor motif containing 6	TMBIM6	0,99
signal peptidase complex subunit 1	SPCS1	0,99
3-hydroxymethyl-3-methylglutaryl-CoA lyase-like 1	HMGCLL1	0,99
beta-site APP-cleaving enzyme 2	BACE2	0,99
hydroxymethylbilane synthase	HMBS	0,99
colony stimulating factor 2 (granulocyte-macrophage)	CSF2	0,99
potassium channel, voltage gated subfamily A regulatory beta subunit 3	KCNAB3	0,99
olfactory receptor, family 4, subfamily D, member 1	OR4D1	0,99
maelstrom spermatogenic transposon silencer	MAEL	0,99

desmoglein 3	DSG3	0,99
CDP-diacylglycerol synthase 2	CDS2	0,99
alkaline ceramidase 1	ACER1	0,99
uncharacterized LOC102724323; Transcript Identified by AceView, Entrez Gene ID(s) 220972; novel transcript	LOC102724323; RP11-67C2.2; MARCH8	0,99
Memczak2013 ALT_ACCEPTOR, ALT_DONOR, coding, INTERNAL, intronic best transcript NM_181558	RFC3	0,99
olfactory receptor, family 2, subfamily B, member 11	OR2B11	0,99
leucine-rich repeat kinase 2	LRRK2	0,99
Ras homolog enriched in brain	RHEB	0,99
methionine sulfoxide reductase B2	MSRB2	0,99
F-box protein 7	FBXO7	0,99
jun D proto-oncogene	JUND	0,99
phospholipase A2 inhibitor and LY6/PLAUR domain containing	PINLYP	0,99
regulation of nuclear pre-mRNA domain containing 2	RPRD2	0,99
family with sequence similarity 198, member A	FAM198A	0,99
B-box and SPRY domain containing	BSPRY	0,99
keratin associated protein 5-10	KRTAP5-10	0,99
protein phosphatase 1, regulatory subunit 15B	PPP1R15B	0,99
interferon regulatory factor 4	IRF4	0,99
membrane associated ring finger 11	MARCH11	0,99
cortactin binding protein 2	CTTNBP2	0,99
coiled-coil domain containing 36	CCDC36	0,99
protocadherin 18	PCDH18	0,99
olfactory receptor, family 6, subfamily V, member 1	OR6V1	0,99
DnaJ (Hsp40) homolog, subfamily C, member 18	DNAJC18	0,99
FK506 binding protein 9	FKBP9	0,99
T-box 3	TBX3	0,99
clathrin, heavy chain-like 1	CLTCL1	0,99
proteasome (prosome, macropain) assembly chaperone 3	PSMG3	0,99
5-hydroxytryptamine (serotonin) receptor 2B, G protein-coupled	HTR2B	0,99
spermatogenesis and oogenesis specific basic helix-loop-helix 1	SOHLH1	0,99
carbonic anhydrase III	CA3	0,99
C-type lectin domain family 3, member A	CLEC3A	0,99
otolin 1	OTOL1	0,99
olfactory receptor, family 4, subfamily Q, member 3	OR4Q3	0,99
Memczak2013 ALT_ACCEPTOR, ALT_DONOR, coding, INTERNAL, intronic best transcript NM_001193571; Transcript Identified by AceView	CSRP1; markobu	0,99
LIM homeobox transcription factor 1, alpha	LMX1A	0,99

glutamate receptor interacting protein 1	GRIP1	0,99
left-right determination factor 1	LEFTY1	0,99
coiled-coil domain containing 186; microRNA 2110	CCDC186; MIR2110	0,99
GRB2-associated binding protein 3	GAB3	0,99
interferon, epsilon; MIR31 host gene	IFNE; MIR31HG	0,99
EF-hand domain (C-terminal) containing 2	EFHC2	0,99
eukaryotic translation initiation factor 4A3	EIF4A3	0,99
solute carrier family 39 (zinc transporter), member 12	SLC39A12	0,99
REX1, RNA exonuclease 1 homolog-like 1, pseudogene; REX1, RNA exonuclease 1 homolog-like 3, pseudogene	REXO1L1P; REXO1L3P	0,99
ZFP91-CNTF readthrough (NMD candidate)	ZFP91-CNTF	0,99
hydroxysteroid (17-beta) dehydrogenase 8	HSD17B8	0,99
copine family member IX	CPNE9	0,99
ALG1, chitobiosyldiphosphodolichol beta-mannosyltransferase-like 2	ALG1L2	0,99
ELKS/RAB6-interacting/CAST family member 1	ERC1	0,99
chromosome 6 open reading frame 58	C6orf58	0,99
PRAME family member 7	PRAMEF7	0,99
zinc finger protein 35	ZNF35	0,99
Transcript Identified by AceView, Entrez Gene ID(s) 55278	QRSL1	0,99
zinc finger, DHHC-type containing 24	ZDHHC24	0,99
sodium channel, voltage gated, type IV beta subunit	SCN4B	0,99
ankyrin repeat and sterile alpha motif domain containing 1A	ANKS1A	0,99
telomeric repeat binding factor 2	TERF2	0,99
small integral membrane protein 4	SMIM4	0,99
zinc finger protein 833, pseudogene	ZNF833P	0,99
matrix metallopeptidase 23B; matrix metallopeptidase 23A (pseudogene)	MMP23B; MMP23A	0,99
NLR family, pyrin domain containing 6	NLRP6	0,99
prosaposin-like 1 (gene/pseudogene)	PSAPL1	0,99
transmembrane protein 215	TMEM215	0,99
C2 calcium-dependent domain containing 4B	C2CD4B	0,99
Synthetic construct Homo sapiens clone IMAGE:100063377, MGC:190767 ribosomal protein L41 (RPL41) mRNA, encodes complete protein.; 60S ribosomal protein L41 [Source:UniProtKB/Swiss-Prot;Acc:P62945]	RPL41	0,99
EF-hand calcium binding domain 14	EFCAB14	0,99
family with sequence similarity 159, member A	FAM159A	0,99
Yip1 domain family member 4	YIPF4	0,99
activating transcription factor 6	ATF6	0,99
zinc finger, FYVE domain containing 27	ZFYVE27	0,99

protease, serine 41	PRSS41	0,99
protein inhibitor of activated STAT 2	PIAS2	0,99
chromosome 3 open reading frame 22	C3orf22	0,99
methyltransferase like 3	METTL3	0,99
C5orf66 antisense RNA 1	C5orf66-AS1	0,99
DEAD (Asp-Glu-Ala-Asp) box helicase 5; microRNA 3064; microRNA 5047	DDX5; MIR3064; MIR5047	0,99
solute carrier family 25 (S-adenosylmethionine carrier), member 26	SLC25A26	0,99
NADH dehydrogenase (ubiquinone) 1 alpha subcomplex, 4-like 2	NDUFA4L2	0,99
nudix hydrolase 10	NUDT10	0,99
ribonuclease, RNase A family, 8	RNASE8	0,99
doublecortin domain containing 2C	DCDC2C	0,99
pleckstrin and Sec7 domain containing 4	PSD4	0,99
novel transcript; Transcript Identified by AceView, Entrez Gene ID(s) 5690	RP4-728D4.2; PSMB2	0,99
S100 calcium binding protein G	S100G	0,99
secretogranin II	SCG2	0,99
testis expressed 28	TEX28	0,99
C-type lectin domain family 5, member A	CLEC5A	0,99
ubiquitin-conjugating enzyme E2O	UBE2O	0,99
regulator of G-protein signaling 22	RGS22	0,99
cadherin 9, type 2 (T1-cadherin)	CDH9	0,99
calcium/calmodulin-dependent protein kinase IV	CAMK4	0,99
HAUS augmin like complex subunit 3; polymerase (DNA directed) nu	HAUS3; POLN	0,99
chromosome 1 open reading frame 122	C1orf122	0,99
endo-beta-N-acetylglucosaminidase	ENGASE	0,99
MT-RNR2-like 5	MTRNR2L5	0,99
polypeptide N-acetylgalactosaminyltransferase 18	GALNT18	0,99
Synthetic construct Homo sapiens clone IMAGE:100063377, MGC:190767 ribosomal protein L41 (RPL41) mRNA, encodes complete protein.	RPL41	0,99
sorting nexin 17	SNX17	0,99
zinc finger protein 546	ZNF546	0,99
protein kinase C, eta	PRKCH	0,99
B-cell translocation gene 4	BTG4	0,99
SRY box 21	SOX21	0,99
solute carrier family 25, member 45	SLC25A45	0,99
transmembrane and coiled-coil domains 2	TMCO2	0,99
PRAME family member 25	PRAMEF25	0,99
PRAME family member 25	PRAMEF25	0,99
FAM83H antisense RNA 1 (head to head)	FAM83H-AS1	0,99
zinc finger with KRAB and SCAN domains 7	ZKSCAN7	0,99

3-hydroxyacyl-CoA dehydratase 3	HACD3	0,99
olfactory receptor, family 7, subfamily C, member 1	OR7C1	0,99
MICAL C-terminal like	MICALCL	0,99
N-6 adenine-specific DNA methyltransferase 2 (putative)	N6AMT2	0,99
bromodomain and PHD finger containing 3	BRPF3	0,99
E4F transcription factor 1	E4F1	0,99
zinc finger with KRAB and SCAN domains 7	ZKSCAN7	0,99
iroquois homeobox 4	IRX4	0,99
leucine rich repeat containing 37, member A4, pseudogene	LRRC37A4P	0,99
F-box protein 15	FBXO15	0,99
wingless-type MMTV integration site family, member 1	WNT1	0,99
SPATA31 subfamily A, member 7; SPATA31 subfamily A, member 5	SPATA31A7; SPATA31A5	0,99
apolipoprotein C-II	APOC2	0,99
actinin, alpha 2	ACTN2	0,99
adenylate cyclase 2 (brain)	ADCY2	0,99
zinc finger protein 148	ZNF148	0,99
phospholipid phosphatase 6	PLPP6	0,99
piwi-like RNA-mediated gene silencing 3	PIWIL3	0,99
olfactory receptor, family 8, subfamily B, member 2	OR8B2	0,99
zinc finger protein 806	ZNF806	0,99
steroidogenic acute regulatory protein	STAR	0,99
sodium channel, non voltage gated 1 delta subunit	SCNN1D	0,99
eukaryotic translation initiation factor 4A2; small nucleolar RNA, H/ACA box 63; small nucleolar RNA, C/D box 2; small nucleolar RNA, H/ACA box 4; small nucleolar RNA, H/ACA box 81; microRNA 1248	EIF4A2; SNORA63; SNORD2; SNORA4; SNORA81; MIR1248	0,99
eva-1 homolog B (C. elegans)	EVA1B	0,99
gastrulation brain homeobox 1	GBX1	0,99
TRAF-interacting protein with forkhead-associated domain, family member B	TIFAB	0,99
olfactory receptor, family 8, subfamily J, member 3	OR8J3	0,99
GPRIN family member 3	GPRIN3	0,99
gap junction protein beta 5	GJB5	0,99
neurotrophin 4	NTF4	0,99
deoxyribonuclease I	DNASE1	0,99
zinc finger, BED-type containing 6; zinc finger CCCH-type containing 11A	ZBED6; ZC3H11A	0,99
anoctamin 10	ANO10	0,99
proteasome subunit alpha 6	PSMA6	0,99
phosphoinositide-3-kinase interacting protein 1	PIK3IP1	0,98

trafficking protein particle complex 4	TRAPPC4	0,98
golgi SNAP receptor complex member 1	GOSR1	0,98
nanos homolog 3 (Drosophila)	NANOS3	0,98
TAF4 RNA polymerase II, TATA box binding protein (TBP)-associated factor, 135kDa	TAF4	0,98
cripto, FRL-1, cryptic family 1	CFC1	0,98
mannosidase, alpha, class 1C, member 1	MAN1C1	0,98
IGF like family member 3	IGFL3	0,98
ribosomal protein L36a	RPL36A	0,98
cholinergic receptor, nicotinic alpha 4	CHRNA4	0,98
DSN1 homolog, MIS12 kinetochore complex component	DSN1	0,98
LIM domain binding 3	LDB3	0,98
T-box 18	TBX18	0,98
protein arginine methyltransferase 8	PRMT8	0,98
potassium channel, voltage gated modifier subfamily G, member 4	KCNG4	0,98
double homeobox 4	DUX4	0,98
sialic acid binding Ig-like lectin 1, sialoadhesin	SIGLEC1	0,98
mannosidase, alpha, class 2C, member 1	MAN2C1	0,98
transmembrane protease, serine 13	TMPRSS13	0,98
zinc finger protein 776	ZNF776	0,98
hydroxymethylbilane synthase	HMBS	0,98
torsin A interacting protein 1	TOR1AIP1	0,98
purine-rich element binding protein B; microRNA 4657	PURB; MIR4657	0,98
protein phosphatase 1, regulatory (inhibitor) subunit 1B	PPP1R1B	0,98
polycystic kidney and hepatic disease 1 (autosomal recessive)-like 1	PKHD1L1	0,98
inducible T-cell co-stimulator ligand	ICOSLG	0,98
zinc finger protein 197	ZNF197	0,98
cilia and flagella associated protein 57	CFAP57	0,98
zinc finger protein 440	ZNF440	0,98
MAGE family member A4	MAGEA4	0,98
zinc finger protein 423	ZNF423	0,98
immunoglobulin lambda-like polypeptide 5; microRNA 5571; immunoglobulin lambda constant 1 (Mcg marker); immunoglobulin lambda constant 2 (Kern-Oz- marker); immunoglobulin lambda variable 2-5 (pseudogene); immunoglobulin lambda variable 3-1; immunoglobulin lambda joining 1	IGLL5; MIR5571; IGLC1; IGLC2; IGLV2-5; IGLV3-1; IGLJ1	0,98
DEAD (Asp-Glu-Ala-Asp) box polypeptide 51	DDX51	0,98
aryl hydrocarbon receptor interacting protein-like 1	AIPL1	0,98
olfactory receptor, family 4, subfamily A, member 8 (gene/pseudogene) [Source:HGNC Symbol;Acc:HGNC:15165]	OR4A8	0,98
actin, alpha, cardiac muscle 1	ACTC1	0,98

SIX homeobox 5	SIX5	0,98
embryonal Fyn-associated substrate	EFS	0,98
Jeck2013 ALT_ACCEPTOR, ALT_DONOR, coding, INTERNAL, intronic best transcript NM_057159; Salzman2013 ALT_ACCEPTOR, ALT_DONOR, coding, INTERNAL, intronic best transcript NM_057159	LPAR1	0,98
CACTIN antisense RNA 1	CACTIN-AS1	0,98
matrix metallopeptidase 17 (membrane-inserted)	MMP17	0,98
interleukin 24	IL24	0,98
tetraspanin 32	TSPAN32	0,98
Transcript Identified by AceView, Entrez Gene ID(s) 54874	FNBP1L	0,98
mucin 20, cell surface associated; succinate dehydrogenase complex subunit A, flavoprotein pseudogene 2; microRNA 570; long intergenic non-protein coding RNA 969	MUC20; SDHAP2; MIR570; LINC00969	0,98
katanin p60 subunit A-like 2	KATNAL2	0,98
macrophage expressed 1	MPEG1	0,98
KIAA0586	KIAA0586	0,98
protein tyrosine phosphatase, receptor type, f polypeptide (PTPRF), interacting protein (liprin), alpha 3	PPFIA3	0,98
WDR86 antisense RNA 1	WDR86-AS1	0,98
mitochondrial ribosomal protein S33	MRPS33	0,98
Salzman2013 ALT_ACCEPTOR, ALT_DONOR, coding, INTERNAL, intronic best transcript NM_006989	RASA4	0,98
lysine (K)-specific demethylase 7A	KDM7A	0,98
Transcript Identified by AceView, Entrez Gene ID(s) 57545	CC2D2A	0,98
proline rich 35	PRR35	0,98
transmembrane (C-terminal) protease, serine 12	TMPRSS12	0,98
Transcript Identified by AceView, Entrez Gene ID(s) 7486	WRN	0,98
transcription factor 7 (T-cell specific, HMG-box)	TCF7	0,98
Rho GTPase activating protein 11B; Rho GTPase activating protein 11A	ARHGAP11B; ARHGAP11A	0,98
ribosomal protein S6 kinase, 90kDa, polypeptide 1	RPS6KA1	0,98
delta-like 3 (Drosophila)	DLL3	0,98
von Willebrand factor A domain containing 3B	VWA3B	0,98
NLR family, pyrin domain containing 5	NLRP5	0,98
membrane associated guanylate kinase, WW and PDZ domain containing 2	MAGI2	0,98
platelet factor 4 variant 1	PF4V1	0,98
solute carrier family 7, member 4	SLC7A4	0,98
family with sequence similarity 63, member B	FAM63B	0,98

solute carrier family 47 (multidrug and toxin extrusion), member 2	SLC47A2	0,98
PTC7 protein phosphatase homolog	PPTC7	0,98
nuclear receptor subfamily 4, group A, member 2	NR4A2	0,98
tripartite motif containing 44	TRIM44	0,98
splicing factor 3b subunit 5	SF3B5	0,98
PILR alpha associated neural protein	PIANP	0,98
olfactory receptor, family 5, subfamily T, member 3	OR5T3	0,98
chemokine (C-C motif) ligand 11	CCL11	0,98
ELAV like neuron-specific RNA binding protein 2	ELAVL2	0,98
mutY DNA glycosylase	MUTYH	0,98
forkhead box D1	FOXD1	0,98
hyperpolarization activated cyclic nucleotide gated potassium channel 4	HCN4	0,98
family with sequence similarity 231, member A	FAM231A	0,98
zinc finger protein 630	ZNF630	0,98
TBC1 domain family, member 32	TBC1D32	0,98
methyl-CpG binding domain protein 3-like 1	MBD3L1	0,98
leucine-rich repeats and IQ motif containing 1	LRRIQ1	0,98
chromosome 11 open reading frame 85	C11orf85	0,98
MAS-related GPR, member X2	MRGPRX2	0,98
thyrotropin-releasing hormone receptor	TRHR	0,98
MARVEL domain containing 3	MARVELD3	0,98
transient receptor potential cation channel, subfamily C, member 6	TRPC6	0,98
cilia and flagella associated protein 44	CFAP44	0,98
keratin associated protein 4-7	KRTAP4-7	0,98
chromosome 20 open reading frame 197	C20orf197	0,98
cilia and flagella associated protein 47	CFAP47	0,98
ATP binding cassette subfamily C member 8	ABCC8	0,98
SRY box 3	SOX3	0,98
tousled-like kinase 2	TLK2	0,98
alcohol dehydrogenase 1B (class I), beta polypeptide	ADH1B	0,98
zinc finger protein 729	ZNF729	0,98
nardilysin convertase; microRNA 761	NRDC; MIR761	0,98
F11 antisense RNA 1	F11-AS1	0,98
metallo-beta-lactamase domain containing 1	MBLAC1	0,98
ribosomal protein S6 kinase, 90kDa, polypeptide 1	RPS6KA1	0,98
zinc finger protein 367	ZNF367	0,98
zinc finger protein 180	ZNF180	0,98
MAGE family member L2	MAGEL2	0,98
Memczak2013 ALT_ACCEPTOR, ALT_DONOR, coding, INTERNAL, intronic best transcript NM_000969	RPL5	0,98
protein kinase, cAMP-dependent, regulatory, type II, beta	PRKAR2B	0,98

amine oxidase, copper containing 2 (retina-specific)	AOC2	0,98
RIMS binding protein 3	RIMBP3	0,98
Memczak2013 ANTISENSE, coding, INTERNAL, intronic best transcript NM_014265	ADAM28	0,98
phosphotyrosine interaction domain containing 1	PID1	0,98
chromosome 6 open reading frame 163	C6orf163	0,98
polymerase (DNA directed), mu; microRNA 6838	POLM; MIR6838	0,98
dynein, axonemal, heavy chain 1	DNAH1	0,98
gametocyte specific factor 1-like	GTSF1L	0,98
Na+/K+ transporting ATPase interacting 3	NKAIN3	0,98
regulator of G-protein signaling 6	RGS6	0,98
polymerase (DNA directed), delta 1, catalytic subunit	POLD1	0,98
FEZ family zinc finger 1	FEZF1	0,98
H2A histone family, member J	H2AFJ	0,98
sperm acrosome associated 3	SPACA3	0,98
guanine nucleotide binding protein (G protein), gamma 3	GNG3	0,98
actinin, alpha 4	ACTN4	0,98
BPI fold containing family C	BPIFC	0,98
taste receptor, type 2, member 39	TAS2R39	0,98
glutaminase	GLS	0,98
ATP synthase F0 subunit 8; ATP synthase F0 subunit 6; cytochrome c oxidase III	ATP8; ATP6; COX3	0,98
olfactory receptor, family 4, subfamily C, member 6	OR4C6	0,98
zinc finger, BED-type containing 9	ZBED9	0,98
olfactory receptor, family 51, subfamily B, member 2 (gene/pseudogene)	OR51B2	0,98
apolipoprotein B mRNA editing enzyme, catalytic polypeptide-like 4 (putative)	APOBEC4	0,98
long intergenic non-protein coding RNA 1235	LINC01235	0,98
musculin	MSC	0,98
transmembrane protein 240	TMEM240	0,98
Transcript Identified by AceView, Entrez Gene ID(s) 3836	KPNA1	0,98
growth differentiation factor 9	GDF9	0,98
spermatogenesis associated 16	SPATA16	0,98
synapse differentiation inducing 1	SYNDIG1	0,98
guanine nucleotide binding protein (G protein), alpha inhibiting activity polypeptide 2	GNAI2	0,98
keratin 6C, type II	KRT6C	0,98
defensin, beta 113	DEFB113	0,98
ADAM metallopeptidase with thrombospondin type 1 motif 13	ADAMTS13	0,98
olfactory receptor, family 2, subfamily L, member 5	OR2L5	0,98
progressive rod-cone degeneration	PRCD	0,98

PDZ domain containing ring finger 4	PDZRN4	0,98
protein tyrosine phosphatase, receptor type, D	PTPRD	0,98
tumor suppressor candidate 2	TUSC2	0,98
eukaryotic translation initiation factor 5A-like 1	EIF5AL1	0,98
S-antigen; retina and pineal gland (arrestin)	SAG	0,98
gamma-aminobutyric acid (GABA) A receptor, alpha 6	GABRA6	0,98
phosphatidylinositol-4,5-bisphosphate 3-kinase, catalytic subunit gamma	PIK3CG	0,98
aldo-keto reductase family 7, member A3 (aflatoxin aldehyde reductase)	AKR7A3	0,98
E2F-associated phosphoprotein	EAPP	0,98
shisa family member 7	SHISA7	0,98
taste receptor, type 2, member 38	TAS2R38	0,98
SMAD family member 2	SMAD2	0,98
dehydrogenase/reductase (SDR family) member 7C	DHRS7C	0,98
family with sequence similarity 89, member A; microRNA 1182	FAM89A; MIR1182	0,98
metal response element binding transcription factor 2	MTF2	0,98
APC membrane recruitment protein 3	AMER3	0,98
cytokine induced apoptosis inhibitor 1	CIAPIN1	0,98
seizure related 6 homolog (mouse)	SEZ6	0,98
glutamate receptor, ionotropic, N-methyl D-aspartate 2C	GRIN2C	0,98
zinc finger, CW type with PWWP domain 2	ZCWPW2	0,98
NADH dehydrogenase (ubiquinone) 1 beta subcomplex, 9, 22kDa	NDUFB9	0,98
neurocan	NCAN	0,98
coiled-coil domain containing 184	CCDC184	0,98
calpain 9	CAPN9	0,98
sclerostin	SOST	0,98
phospholipase A2 receptor 1	PLA2R1	0,98
transmembrane protein 187	TMEM187	0,98
Kruppel-like factor 17	KLF17	0,98
proteoglycan 4	PRG4	0,98
ephrin-A5	EFNA5	0,98
histone cluster 1, H2bh	HIST1H2BH	0,98
carboxypeptidase A3 (mast cell)	CPA3	0,98
IBA57 homolog, iron-sulfur cluster assembly	IBA57	0,98
interleukin 17F	IL17F	0,98
dual specificity phosphatase 12	DUSP12	0,98
thioredoxin domain containing 12 (endoplasmic reticulum); KTI12 chromatin associated homolog	TXNDC12; KTI12	0,98
sterile alpha motif domain containing 10	SAMD10	0,98
ribosomal protein L41 pseudogene 1	RPL41P1	0,98

novel transcript, sense overlapping TAF10; Transcript Identified by AceView, Entrez Gene ID(s) 6881	RP11-732A19.2; TAF10	0,98
teneurin transmembrane protein 3	TENM3	0,98
BCL2-like 2	BCL2L2	0,98
solute carrier family 24 (sodium/potassium/calcium exchanger), member 3	SLC24A3	0,98
IZUMO family member 3	IZUMO3	0,98
LysM, putative peptidoglycan-binding, domain containing 4	LYSMD4	0,98
ankyrin-repeat and fibronectin type III domain containing 1	ANKFN1	0,98
potassium channel tetramerization domain containing 16	KCTD16	0,98
alpha 1,4-galactosyltransferase	A4GALT	0,98
cyclic nucleotide gated channel alpha 3	CNGA3	0,98
histidine rich calcium binding protein	HRC	0,98
cytochrome b-245, beta polypeptide	CYBB	0,98
paired immunoglobin-like type 2 receptor alpha	PILRA	0,98
lipoprotein, Lp(a)-like 2, pseudogene	LPAL2	0,98
RNA binding motif protein 5	RBM5	0,98
PRAME family member 20	PRAMEF20	0,98
olfactory receptor, family 4, subfamily D, member 6	OR4D6	0,98
epididymal peptidase inhibitor	EPPIN	0,98
adenylate cyclase activating polypeptide 1 (pituitary) receptor type I	ADCYAP1R1	0,98
chromosome 12 open reading frame 40	C12orf40	0,98
abhydrolase domain containing 1	ABHD1	0,98
poly(ADP-ribose) polymerase family member 9	PARP9	0,98
sialic acid binding Ig-like lectin 14; sialic acid binding Ig-like lectin 5	SIGLEC14; SIGLEC5	0,98
ferric-chelate reductase 1	FRRS1	0,98
quinoid dihydropteridine reductase	QDPR	0,98
dual oxidase 1	DUOX1	0,98
Transcript Identified by AceView, Entrez Gene ID(s) 9320	TRIP12	0,98
mediator complex subunit 24; microRNA 6884	MED24; MIR6884	0,98
atlastin GTPase 1	ATL1	0,98
neuregulin 4	NRG4	0,98
major histocompatibility complex, class II, DQ beta 2	HLA-DQB2	0,98
Memczak2013 ANTISENSE, CDS, coding, INTERNAL best transcript NM_181786	HKR1	0,98
Fc fragment of IgA receptor	FCAR	0,98
solute carrier family 4, sodium bicarbonate cotransporter, member 9	SLC4A9	0,98
alanine--glyoxylate aminotransferase 2	AGXT2	0,98

WD repeat domain 97	WDR97	0,98
prostaglandin I2 (prostacyclin) synthase	PTGIS	0,98
novel transcript, antisense to C16orf53 and MVP; PAXIP1 associated glutamate-rich protein 1 [Source:HGNC Symbol;Acc:HGNC:28707]	AC009133.12; PAGR1	0,98
doublesex and mab-3 related transcription factor 3	DMRT3	0,98
autophagy related 9B	ATG9B	0,98
neurotrimin	NTM	0,98
ladybird homeobox 1	LBX1	0,98
NUT midline carcinoma, family member 1	NUTM1	0,98
ribonuclease, RNase A family, 12 (non-active); ribonuclease, RNase A family, 11 (non-active)	RNASE12; RNASE11	0,98
defensin, beta 106B; defensin, beta 106A	DEFB106B; DEFB106A	0,98
Transcript Identified by AceView, Entrez Gene ID(s) 65980	BRD9	0,98
DTW domain containing 1	DTWD1	0,98
RDH10 antisense RNA 1	RDH10-AS1	0,98
brain-derived neurotrophic factor	BDNF	0,98
WAS protein family homolog 1	WASH1	0,98
PDZ and LIM domain 1	PDLIM1	0,98
carcinoembryonic antigen-related cell adhesion molecule 6 (non-specific cross reacting antigen)	CEACAM6	0,98
neuroblastoma breakpoint family, member 4	NBPF4	0,98
potassium channel tetramerization domain containing 8	KCTD8	0,98
schlafen family member 12-like	SLFN12L	0,98
chromosome 2 open reading frame 88	C2orf88	0,98
matrix metallopeptidase 24 (membrane-inserted)	MMP24	0,98
HAND2 antisense RNA 1 (head to head)	HAND2-AS1	0,98
family with sequence similarity 131, member A	FAM131A	0,98
F-box protein 3	FBXO3	0,98
complement component (3b/4b) receptor 1 (Knops blood group)	CR1	0,98
zinc finger protein 711	ZNF711	0,98
leucine rich colipase-like 1	LRCOL1	0,98
family with sequence similarity 53, member C	FAM53C	0,98
ST8 alpha-N-acetyl-neuraminide alpha-2,8-sialyltransferase 5	ST8SIA5	0,98
Wolf-Hirschhorn syndrome candidate 1-like 1	WHSC1L1	0,98
ATPase, H+ transporting, lysosomal V0 subunit a2	ATP6V0A2	0,98
leucine rich repeat and Ig domain containing 1	LINGO1	0,98
potassium channel, voltage gated KQT-like subfamily Q, member 4	KCNQ4	0,98
limb and CNS expressed 1	LIX1	0,98
formin 1	FMN1	0,98

CDC like kinase 1	CLK1	0,98
cytochrome P450, family 4, subfamily Z, polypeptide 1	CYP4Z1	0,98
keratin associated protein 19-8	KRTAP19-8	0,98
transmembrane protein 176A	TMEM176A	0,98
solute carrier family 30 (zinc transporter), member 8	SLC30A8	0,98
PRELI domain containing 1	PRELID1	0,98
Memczak2013 ANTISENSE, coding, INTERNAL, intronic best transcript NM_012399	PITPNB	0,98
CDKN2A interacting protein N-terminal like	CDKN2AIPNL	0,98
family with sequence similarity 187, member B	FAM187B	0,98
Jeck2013 ALT_ACCEPTOR, ALT_DONOR, coding, INTERNAL, intronic best transcript NM_032531	KIRREL3	0,98
cyclin-dependent kinase 14	CDK14	0,98
nuclear transcription factor, X-box binding-like 1	NFXL1	0,98
deltex 1, E3 ubiquitin ligase	DTX1	0,98
chromosome 11 open reading frame 74	C11orf74	0,98
relaxin/insulin-like family peptide receptor 2	RXFP2	0,98
sex comb on midleg-like 1 (Drosophila)	SCML1	0,98
DEAH (Asp-Glu-Ala-His) box polypeptide 34	DHX34	0,98
activity-dependent neuroprotector homeobox	ADNP	0,98
polypeptide N-acetylgalactosaminyltransferase 3	GALNT3	0,98
collagen, type XXV, alpha 1	COL25A1	0,98
growth differentiation factor 5	GDF5	0,98
olfactory receptor, family 13, subfamily J, member 1	OR13J1	0,98
DEAD (Asp-Glu-Ala-Asp) box helicase 6	DDX6	0,98
zinc finger protein 705E	ZNF705E	0,98
leucine rich repeat neuronal 2	LRRN2	0,98
coiled-coil domain containing 116	CCDC116	0,98
solute carrier family 35, member G6	SLC35G6	0,98
tripartite motif containing 23	TRIM23	0,98
major facilitator superfamily domain containing 2A	MFSD2A	0,98
proline rich 19	PRR19	0,98
small integral membrane protein 23	SMIM23	0,98
myosin light chain 10	MYL10	0,98
hemoglobin, delta	HBD	0,98
DAZ interacting zinc finger protein 3	DZIP3	0,98
Transcript Identified by AceView, Entrez Gene ID(s) 149111; novel transcript	CNIH3; RP11-449J1.1	0,98
NMD3 ribosome export adaptor	NMD3	0,98
notch 3	NOTCH3	0,98
Transcript Identified by AceView, Entrez Gene ID(s) 149840	C20orf196	0,98
phosphoribosylglycinamide formyltransferase, phosphoribosylglycinamide synthetase, phosphoribosylaminoimidazole synthetase	GART	0,98

epithelial stromal interaction 1 (breast)	EPSTI1	0,98
origin recognition complex subunit 2	ORC2	0,98
dendrin	DDN	0,98
Transcript Identified by AceView, Entrez Gene ID(s) 1997; 100128628	ELF1	0,98
olfactory receptor, family 4, subfamily A, member 5	OR4A5	0,98
tribbles pseudokinase 3	TRIB3	0,98
kinase suppressor of ras 1	KSR1	0,98
basic helix-loop-helix domain containing, class B, 9	BHLHB9	0,98
protein tyrosine phosphatase, receptor type, N	PTPRN	0,98
carbohydrate (N-acetylgalactosamine 4-0) sulfotransferase 8	CHST8	0,98
transmembrane protein 213	TMEM213	0,98
glutamic-oxaloacetic transaminase 2, mitochondrial	GOT2	0,98
MAGE family member B18	MAGEB18	0,98
CDK5 regulatory subunit associated protein 1	CDK5RAP1	0,98
chorionic gonadotropin, beta polypeptide 7	CGB7	0,98
MT-RNR2-like 7	MTRNR2L7	0,98
long intergenic non-protein coding RNA 173	LINC00173	0,98
cyclin-dependent kinase 2 interacting protein	CINP	0,98
LIM and senescent cell antigen-like domains 3-like	LIMS3L	0,98
C1q and tumor necrosis factor related protein 1	C1QTNF1	0,98
pleckstrin homology domain containing, family A (phosphoinositide binding specific) member 3	PLEKHA3	0,98
chromosome 3 open reading frame 80	C3orf80	0,98
proteasome subunit beta 10	PSMB10	0,98
dopamine beta-hydroxylase (dopamine beta-monooxygenase)	DBH	0,98
cell division cycle 73	CDC73	0,98
nucleoporin 85kDa	NUP85	0,98
B-cell translocation gene 1, anti-proliferative; long intergenic non-protein coding RNA 1619	BTG1; LINC01619	0,98
zinc finger protein 407	ZNF407	0,98
wingless-type MMTV integration site family, member 9B	WNT9B	0,98
GLI family zinc finger 3	GLI3	0,98
intraflagellar transport 88	IFT88	0,98
claudin 18	CLDN18	0,98
Fc fragment of IgE, low affinity II, receptor for (CD23)	FCER2	0,98
microseminoprotein, beta-	MSMB	0,98
uromodulin-like 1	UMODL1	0,98
cat eye syndrome chromosome region, candidate 6	CECR6	0,98
killer cell immunoglobulin-like receptor, two domains, long cytoplasmic tail, 4	KIR2DL4	0,98
Tctex1 domain containing 4	TCTEX1D4	0,98

kelch repeat and BTB (POZ) domain containing 13	KBTBD13	0,98
neuroblastoma breakpoint family, member 12	NBPF12	0,98
protein tyrosine phosphatase, receptor type, C-associated protein	PTPRCAP	0,98
coenzyme Q6 monooxygenase	COQ6	0,98
SEBOX homeobox	SEBOX	0,98
coiled-coil domain containing 43	CCDC43	0,98
Salzman2013 ANNOTATED, coding, OVEXON, UTR5 best transcript NM_001077664	URGCP	0,98
endoplasmic reticulum-golgi intermediate compartment 1	ERGIC1	0,98
transient receptor potential cation channel, subfamily V, member 5	TRPV5	0,98
succinate-CoA ligase, ADP-forming, beta subunit	SUCLA2	0,98
SPARC like 1	SPARCL1	0,98
mucin 5AC, oligomeric mucus/gel-forming	MUC5AC	0,98
dual specificity phosphatase 23	DUSP23	0,98
glutamate receptor interacting protein 2	GRIP2	0,98
myogenin (myogenic factor 4)	MYOG	0,98
C2 calcium-dependent domain containing 4C	C2CD4C	0,98
protein phosphatase 1, regulatory subunit 9B	PPP1R9B	0,98
olfactory receptor, family 4, subfamily C, member 15	OR4C15	0,98
surfactant protein C	SFTPC	0,98
transmembrane protein 147	TMEM147	0,98
guanine nucleotide binding protein (G protein), gamma transducing activity polypeptide 2	GNGT2	0,98
olfactory receptor, family 4, subfamily K, member 1	OR4K1	0,98
chromosome 6 open reading frame 99	C6orf99	0,98
G protein-coupled receptor 63	GPR63	0,98
chemokine (C-C motif) ligand 2	CCL2	0,98
glycine-N-acyltransferase-like 1	GLYATL1	0,98
coiled-coil domain containing 97	CCDC97	0,97
proprotein convertase subtilisin/kexin type 5	PCSK5	0,97
prostaglandin E receptor 1	PTGER1	0,97
guanylate cyclase 2F, retinal	GUCY2F	0,97
fibronectin type III domain containing 8	FNDC8	0,97
chromosome 1 open reading frame 74	C1orf74	0,97
ATPase, class I, type 8B, member 4	ATP8B4	0,97
F-box protein 42	FBXO42	0,97
actin, alpha 2, smooth muscle, aorta	ACTA2	0,97
WD repeat containing, antisense to TP53	WRAP53	0,97
potassium channel, voltage gated eag related subfamily H, member 4	KCNH4	0,97
Scm-like with four mbt domains 1	SFMBT1	0,97
AE binding protein 1; microRNA 4649	AEBP1; MIR4649	0,97

Transcript Identified by AceView, Entrez Gene ID(s) 2326	FMO1	0,97
seryl-tRNA synthetase 2, mitochondrial	SARS2	0,97
coiled-coil domain containing 158	CCDC158	0,97
coenzyme Q2 4-hydroxybenzoate polyprenyltransferase	COQ2	0,97
olfactory receptor, family 5, subfamily R, member 1 (gene/pseudogene)	OR5R1	0,97
statherin	STATH	0,97
joining chain of multimeric IgA and IgM	JCHAIN	0,97
ATPase, aminophospholipid transporter (APLT), class I, type 8A, member 1	ATP8A1	0,97
carcinoembryonic antigen-related cell adhesion molecule 19	CEACAM19	0,97
single-pass membrane protein with coiled-coil domains 1	SMCO1	0,97
UL16 binding protein 1	ULBP1	0,97
RasGEF domain family member 1A	RASGEF1A	0,97
Transcript Identified by AceView, Entrez Gene ID(s) 4928	NUP98	0,97
phytanoyl-CoA dioxygenase domain containing 1	PHYHD1	0,97
chromosome 17 open reading frame 67	C17orf67	0,97
coiled-coil domain containing 71-like	CCDC71L	0,97
protein tyrosine phosphatase type IVA, member 1	PTP4A1	0,97
dihydroorotate dehydrogenase (quinone)	DHODH	0,97
nipsnap homolog 3A (C. elegans); nipsnap homolog 3B (C. elegans)	NIPSNAP3A; NIPSNAP3B	0,97
FK506 binding protein 1B	FKBP1B	0,97
chymotrypsin-like elastase family, member 3A	CELA3A	0,97
Nik related kinase	NRK	0,97
ADAM metallopeptidase with thrombospondin type 1 motif 13	ADAMTS13	0,97
solute carrier family 6 (proline IMINO transporter), member 20	SLC6A20	0,97
troponin T type 2 (cardiac)	TNNT2	0,97
olfactory receptor, family 9, subfamily G, member 4	OR9G4	0,97
zinc finger CCCH-type containing 3	ZC3H3	0,97
FIC domain containing	FICD	0,97
cathepsin W	CTSW	0,97
phosphodiesterase 4C, cAMP-specific	PDE4C	0,97
meteorin, glial cell differentiation regulator	METRN	0,97
actin-related protein 2/3 complex inhibitor	ARPIN	0,97
Meckel syndrome, type 1	MKS1	0,97
solute carrier family 4, sodium bicarbonate cotransporter, member 7	SLC4A7	0,97
isthmin 2	ISM2	0,97

nuclear receptor subfamily 3, group C, member 2	NR3C2	0,97
adhesion G protein-coupled receptor E1	ADGRE1	0,97
pleckstrin homology domain containing, family D (with coiled-coil domains) member 1	PLEKHD1	0,97
THAP domain containing, apoptosis associated protein 3	THAP3	0,97
hemogen	HEMGN	0,97
chromosome 1 open reading frame 137	C1orf137	0,97
prolactin-induced protein	PIP	0,97
sarcoma antigen 1	SAGE1	0,97
chromosome 12 open reading frame 80	C12orf80	0,97
POU class 4 homeobox 3	POU4F3	0,97
ADAM metallopeptidase domain 11	ADAM11	0,97
tripartite motif containing 48	TRIM48	0,97
guanylate cyclase activator 2B (uroguanylin)	GUCA2B	0,97
olfactory receptor, family 5, subfamily AL, member 1 (gene/pseudogene)	OR5AL1	0,97
ataxin 3-like	ATXN3L	0,97
long intergenic non-protein coding RNA 1140	LINC01140	0,97
RAS (RAD and GEM)-like GTP binding 2	REM2	0,97
olfactory receptor, family 11, subfamily H, member 7 (gene/pseudogene)	OR11H7	0,97
ataxin 7-like 2	ATXN7L2	0,97
pre-B-cell leukemia homeobox 1	PBX1	0,97
exosome component 7	EXOSC7	0,97
WD repeat domain 17	WDR17	0,97
MAGE family member B10	MAGEB10	0,97
hydroxy-delta-5-steroid dehydrogenase, 3 beta- and steroid delta-isomerase 1	HSD3B1	0,97
canopy FGF signaling regulator 3	CNPY3	0,97
TOPORS antisense RNA 1; GVQW motif containing 1	TOPORS-AS1; GVQW1	0,97
zymogen granule protein 16	ZG16	0,97
killer cell immunoglobulin-like receptor, three domains, X1	KIR3DX1	0,97
Src-like-adaptor 2	SLA2	0,97
eosinophil peroxidase	EPX	0,97
COP9 signalosome subunit 7B	COPS7B	0,97
Jeck2013 ANTISENSE, coding, INTERNAL, intronic best transcript NM_022765	MICAL1	0,97
interferon, alpha 5	IFNA5	0,97
VENT homeobox	VENTX	0,97
smoothelin-like 2	SMTNL2	0,97
lamin tail domain containing 1	LMNTD1	0,97
Kv channel interacting protein 4	KCNIP4	0,97
mucin 6, oligomeric mucus/gel-forming	MUC6	0,97

folate hydrolase (prostate-specific membrane antigen) 1	FOLH1	0,97
KIAA1324	KIAA1324	0,97
chromosome 1 open reading frame 162	C1orf162	0,97
caspase recruitment domain family, member 14	CARD14	0,97
solute carrier family 6 (neurotransmitter transporter), member 7	SLC6A7	0,97
PHD finger protein 5A	PHF5A	0,97
oligodendrocyte transcription factor 1	OLIG1	0,97
family with sequence similarity 13, member C	FAM13C	0,97
CXADR-like membrane protein	CLMP	0,97
ankyrin repeat domain 66	ANKRD66	0,97
enhancer of polycomb homolog 1 (Drosophila)	EPC1	0,97
family with sequence similarity 212, member B	FAM212B	0,97
family with sequence similarity 3, member D	FAM3D	0,97
ectodysplasin A receptor	EDAR	0,97
neuregulin 2	NRG2	0,97
growth arrest-specific 2 like 1	GAS2L1	0,97
long intergenic non-protein coding RNA 1429	LINC01429	0,97
CDGSH iron sulfur domain 1	CISD1	0,97
uncharacterized LOC102724279; Transcript Identified by AceView; novel transcript	LOC102724279; AC004791.2; nimure	0,97
keratin associated protein 12-4	KRTAP12-4	0,97
keratin associated protein 5-5	KRTAP5-5	0,97
chromosome 14 open reading frame 39	C14orf39	0,97
protein phosphatase 3, catalytic subunit, beta isozyme	PPP3CB	0,97
WSC domain containing 1	WSCD1	0,97
NEDD4 binding protein 2-like 2	N4BP2L2	0,97
tetraspanin 13	TSPAN13	0,97
activin A receptor type IL	ACVRL1	0,97
coiled-coil domain containing 154	CCDC154	0,97
t-complex 10	TCP10	0,97
ATP binding cassette subfamily F member 3	ABCF3	0,97
coagulation factor II (thrombin) receptor-like 3	F2RL3	0,97
long intergenic non-protein coding RNA 1565	LINC01565	0,97
AE binding protein 2	AEBP2	0,97
proline-rich coiled-coil 2B	PRRC2B	0,97

TATA box binding protein associated factor 1D; small nucleolar RNA, H/ACA box 8; small nucleolar RNA, H/ACA box 18; small nucleolar RNA, H/ACA box 40; small nucleolar RNA, H/ACA box 1; small nucleolar RNA, H/ACA box 25; small nucleolar RNA, H/ACA box 32; small nucleolar RNA, C/D box 5; microRNA 1304	TAF1D; SNORA8; SNORA18; SNORA40; SNORA1; SNORA25; SNORA32; SNORD5; MIR1304	0,97
cytochrome b	CYTB	0,97
Zhang2013 ALT_ACCEPTOR, ALT_DONOR, coding, INTERNAL, intronic best transcript NM_022720	DGCR8	0,97
protein kinase N1	PKN1	0,97
uncharacterized LOC101929372; novel transcript	LOC101929372; AC004019.13	0,97
FGFR1 oncogene partner	FGFR1OP	0,97
mitochondrial ribosomal protein S18B	MRPS18B	0,97
peptidyl arginine deiminase, type II	PADI2	0,97
protein tyrosine phosphatase, non-receptor type 22 (lymphoid)	PTPN22	0,97
ceramide synthase 2	CERS2	0,97
olfactory receptor family 4 subfamily K member 3 (gene/pseudogene) [Source:HGNC Symbol;Acc:HGNC:14731]	OR4K3	0,97
two pore segment channel 2	TPCN2	0,97
zinc finger protein 276	ZNF276	0,97
CBP80/20-dependent translation initiation factor; microRNA 4743	CTIF; MIR4743	0,97
phospholipase A2, group VII (platelet-activating factor acetylhydrolase, plasma)	PLA2G7	0,97
tachykinin receptor 3	TACR3	0,97
coiled-coil domain containing 105	CCDC105	0,97
von Willebrand factor A domain containing 3A	VWA3A	0,97
LYR motif containing 4	LYRM4	0,97
IQ motif containing G	IQCG	0,97
putative UPF0607 protein ENSP00000383783	LOC100131107	0,97
minichromosome maintenance complex binding protein	MCMBP	0,97
DnaJ (Hsp40) homolog, subfamily C, member 27	DNAJC27	0,97
leukocyte immunoglobulin-like receptor, subfamily B (with TM and ITIM domains), member 5	LILRB5	0,97
transmembrane protein 61	TMEM61	0,97
tubulin tyrosine ligase-like family member 9	TTLL9	0,97
olfactory receptor, family 1, subfamily Q, member 1	OR1Q1	0,97
synapsin III	SYN3	0,97
Transcript Identified by AceView, Entrez Gene ID(s) 9320	TRIP12	0,97

cytohesin 1 interacting protein	CYTIP	0,97
chromosome 6 open reading frame 226	C6orf226	0,97
ADP-ribosylation factor like GTPase 14 effector protein like	ARL14EPL	0,97
carbohydrate (keratan sulfate Gal-6) sulfotransferase 1	CHST1	0,97
prolyl-tRNA synthetase 2, mitochondrial (putative)	PARS2	0,97
chemokine (C-X-C motif) ligand 11	CXCL11	0,97
myosin VIIA and Rab interacting protein	MYRIP	0,97
OTU deubiquitinase, ubiquitin aldehyde binding 1	OTUB1	0,97
potassium channel, voltage gated modifier subfamily V, member 1	KCNV1	0,97
RAP2C, member of RAS oncogene family	RAP2C	0,97
solute carrier family 13 (sodium/sulfate symporter), member 4	SLC13A4	0,97
growth differentiation factor 10	GDF10	0,97
surfeit 6	SURF6	0,97
small integral membrane protein 10 like 2B	SMIM10L2B	0,97
interleukin 1 receptor associated kinase 3; microRNA 6502	IRAK3; MIR6502	0,97
ATP13A5 antisense RNA 1	ATP13A5-AS1	0,97
t-complex-associated-testis-expressed 1	TCTE1	0,97
follistatin-like 5	FSTL5	0,97
androgen receptor	AR	0,97
family with sequence similarity 183, member A	FAM183A	0,97
Transcript Identified by AceView, Entrez Gene ID(s) 25788	RAD54B	0,97
neuroblastoma breakpoint family, member 19	NBPF19	0,97
CDKN2A interacting protein	CDKN2AIP	0,97
TSC22 domain family, member 3	TSC22D3	0,97
N(alpha)-acetyltransferase 20, NatB catalytic subunit	NAA20	0,97
diphosphoinositol pentakisphosphate kinase 1	PPIP5K1	0,97
formin 2; alcohol dehydrogenase 5 (class III), chi polypeptide, pseudogene 3	FMN2; ADH5P3	0,97
cytokine receptor-like factor 3	CRLF3	0,97
zinc finger, CCHC domain containing 9	ZCCHC9	0,97
leucine-rich single-pass membrane protein 2	LSMEM2	0,97
transmembrane protein 75	TMEM75	0,97
prolactin	PRL	0,97
TWIST neighbor	TWISTNB	0,97
CD80 molecule	CD80	0,97
hydroxysteroid (11-beta) dehydrogenase 1	HSD11B1	0,97
glucosidase, beta (bile acid) 2	GBA2	0,97
zinc finger protein 136	ZNF136	0,97
Sad1 and UNC84 domain containing 5	SUN5	0,97
LKAAEAR motif containing 1	LKAAEAR1	0,97

uncharacterized LOC149950; novel transcript	LOC149950; RP11-410N8.4	0,97
gap junction protein gamma 2	GJC2	0,97
protein kinase C, gamma	PRKCG	0,97
growth arrest-specific 2 like 2	GAS2L2	0,97
olfactory receptor, family 2, subfamily T, member 34	OR2T34	0,97
protease, serine, 23	PRSS23	0,97
INTS3 and NABP interacting protein	INIP	0,97
membrane-spanning 4-domains, subfamily A, member 4A	MS4A4A	0,97
gamma-aminobutyric acid (GABA) A receptor, alpha 1	GABRA1	0,97
sphingosine-1-phosphate lyase 1	SGPL1	0,97
dishevelled-binding antagonist of beta-catenin 3	DACT3	0,97
olfactory receptor, family 52, subfamily N, member 5	OR52N5	0,97
otoconin 90	OC90	0,97
desmoglein 4	DSG4	0,97
potassium channel, voltage gated eag related subfamily H, member 1	KCNH1	0,97
bestrophin 4	BEST4	0,97
phosphatidylserine synthase 1	PTDSS1	0,97
chromosome 5 open reading frame 24	C5orf24	0,97
crystallin beta B3	CRYBB3	0,97
breakpoint cluster region	BCR	0,97
zinc finger protein 618	ZNF618	0,97
rhophilin, Rho GTPase binding protein 1	RHPN1	0,97
hydroxymethylbilane synthase	HMBS	0,97
Transcript Identified by AceView, Entrez Gene ID(s) 130888	FBXO36	0,97
MOB kinase activator 3C	MOB3C	0,97
CD4 molecule	CD4	0,97
Memczak2013 ALT_ACCEPTOR, ALT_DONOR, coding, INTERNAL, intronic best transcript NM_014661	FAM53B	0,97
uncharacterized protein ENSP00000383407-like; Transcript Identified by AceView; HCG1818297; Protein LOC388813 [Source:UniProtKB/TrEMBL;Acc:A0A087WSY0]	LOC388813; bafor; AF165138.7	0,97
ovo-like zinc finger 2	OVOL2	0,97
Transcript Identified by AceView, Entrez Gene ID(s) 171586	ABHD3	0,97
olfactory receptor, family 2, subfamily D, member 3	OR2D3	0,97
family with sequence similarity 131, member C	FAM131C	0,97
sel-1 suppressor of lin-12-like 2 (C. elegans)	SEL1L2	0,97
homeobox D10	HOXD10	0,97
RNA binding motif protein, X-linked-like 3	RBMXL3	0,97
interferon, alpha 17	IFNA17	0,97

family with sequence similarity 181, member B	FAM181B	0,97
EMSY BRCA2-interacting transcriptional repressor	EMSY	0,97
neuronal differentiation 6	NEUROD6	0,97
nuclear receptor interacting protein 2	NRIP2	0,97
5-hydroxytryptamine (serotonin) receptor 3D, ionotropic	HTR3D	0,97
leucine-rich repeats and IQ motif containing 4	LRRIQ4	0,97
olfactory receptor, family 2, subfamily A, member 14; olfactory receptor, family 2, subfamily A, member 2	OR2A14; OR2A2	0,97
ATPase type 13A4	ATP13A4	0,97
Zhang2013 ALT_ACCEPTOR, ALT_DONOR, coding, INTERNAL, intronic best transcript NM_004582	RABGGTB	0,97
family with sequence similarity 71, member E2	FAM71E2	0,97
ADP-ribosylation factor like GTPase 13A	ARL13A	0,97
thioredoxin-related transmembrane protein 3	TMX3	0,97
zinc finger CCCH-type containing 7A	ZC3H7A	0,97
RNA binding protein, fox-1 homolog (C. elegans) 2	RBFOX2	0,97
B-cell acute lymphoblastic leukemia expressed	BLACE	0,97
regulatory factor X, 1 (influences HLA class II expression)	RFX1	0,97
Ellis van Creveld protein	EVC	0,97
interferon induced transmembrane protein 5	IFITM5	0,97
NADH dehydrogenase (ubiquinone) 1 alpha subcomplex, 10, 42kDa	NDUFA10	0,97
regulated endocrine-specific protein 18	RESP18	0,97
EF-hand calcium binding domain 9	EFCAB9	0,97
keratin associated protein 10-5	KRTAP10-5	0,97
zinc finger protein 707	ZNF707	0,97
chromosome 11 open reading frame 88	C11orf88	0,97
eukaryotic translation initiation factor 4E family member 1B	EIF4E1B	0,97
peptidase M20 domain containing 2	PM20D2	0,97
meiotic kinetochore factor	MEIKIN	0,97
PR domain containing 16	PRDM16	0,97
chromosome 20 open reading frame 202	C20orf202	0,97
defensin, beta 126	DEFB126	0,97
zinc finger, SWIM-type containing 3	ZSWIM3	0,97
MyoD family inhibitor domain containing	MDFIC	0,97
retinoid X receptor beta	RXRB	0,97
major facilitator superfamily domain containing 6-like	MFSD6L	0,97
Transcript Identified by AceView, Entrez Gene ID(s) 5257	PHKB	0,97
Memczak2013 ANTISENSE, coding, INTERNAL, intronic best transcript NM_001146694	KDM4C	0,97
tubulin folding cofactor E-like	TBCEL	0,97
apolipoprotein L, 4	APOL4	0,97

LY6/PLAUR domain containing 6	LYPD6	0,97
lymphocyte antigen 6 complex, locus K	LY6K	0,97
fibrillarin-like 1	FBLL1	0,97
RAB3A interacting protein (rabin3)-like 1	RAB3IL1	0,97
dynein, axonemal, heavy chain 10 opposite strand	DNAH10OS	0,97
general transcription factor IIF subunit 1	GTF2F1	0,97
transmembrane protein 200A	TMEM200A	0,97
ST8 alpha-N-acetyl-neuraminide alpha-2,8-sialyltransferase 2	ST8SIA2	0,97
Transcript Identified by AceView, Entrez Gene ID(s) 10060	ABCC9	0,97
solute carrier family 4, sodium bicarbonate cotransporter, member 8	SLC4A8	0,97
family with sequence similarity 78, member B	FAM78B	0,97
kin of IRRE like 2 (Drosophila)	KIRREL2	0,97
transmembrane protein 248	TMEM248	0,97
potassium channel, two pore domain subfamily K, member 9	KCNK9	0,97
karyopherin (importin) beta 1	KPNB1	0,97
solute carrier family 22, member 20	SLC22A20	0,97
zinc finger protein 709	ZNF709	0,97
bombesin-like receptor 3	BRS3	0,97
sulfatase modifying factor 1	SUMF1	0,97
bone marrow stromal cell antigen 2	BST2	0,97
calcitonin-related polypeptide alpha	CALCA	0,97
zinc finger protein 513	ZNF513	0,97
olfactory receptor, family 2, subfamily W, member 3	OR2W3	0,97
developmentally regulated GTP binding protein 1	DRG1	0,97
leucine rich repeat containing 72	LRRC72	0,97
BCL2-like 14 (apoptosis facilitator)	BCL2L14	0,97
fibroblast growth factor 8 (androgen-induced)	FGF8	0,97
CD86 molecule	CD86	0,97
fer-1-like family member 6	FER1L6	0,97
transmembrane protein 218	TMEM218	0,97
armadillo repeat containing, X-linked 2	ARMCX2	0,97
kelch repeat and BTB (POZ) domain containing 4	KBTBD4	0,97
retinol dehydrogenase 13 (all-trans/9-cis)	RDH13	0,97
LDL receptor related protein 8	LRP8	0,97
nucleoporin 155kDa	NUP155	0,97
interferon regulatory factor 1	IRF1	0,97
Jeck2013 ANTISENSE, coding, INTERNAL, intronic best transcript NM_001103184	FMN1	0,97
cadherin 7, type 2	CDH7	0,97
lipoyl(octanoyl) transferase 2 (putative)	LIPT2	0,97
synuclein alpha interacting protein	SNCAIP	0,97

small integral membrane protein 10 like 2A	SMIM10L2A	0,97
ring finger protein, LIM domain interacting	RLIM	0,97
hedgehog acyltransferase	HHAT	0,97
YLP motif containing 1	YLPM1	0,97
chondroitin sulfate proteoglycan 4	CSPG4	0,97
vasoactive intestinal peptide receptor 2	VIPR2	0,97
interleukin 12A	IL12A	0,97
coronin, actin binding protein, 2A	CORO2A	0,97
synaptonemal complex protein 2-like	SYCP2L	0,97
shisa family member 2	SHISA2	0,97
carboxypeptidase B1 (tissue)	CPB1	0,97
cytochrome P450, family 20, subfamily A, polypeptide 1	CYP20A1	0,97
glycogen synthase 2 (liver)	GYS2	0,97
hepatoma-derived growth factor, related protein 3; Hepatoma-derived growth factor-related protein 3 [Source:UniProtKB/Swiss-Prot;Acc:Q9Y3E1]; Transcript Identified by AceView, Entrez Gene ID(s) 50810; Jeck2013, Memczak2013, Salzman2013 ANNOTATED, CDS, coding, INTERNAL, OVCODE, OVEXON best transcript NM_016073; Jeck2013 ALT_ACCEPTOR, ALT_DONOR, coding, INTERNAL, intronic best transcript NM_016073; Salzman2013 ANNOTATED, CDS, coding, INTERNAL, OVCODE, OVEXON best transcript NM_016073	HDGFRP3	0,97
ATR interacting protein; three prime repair exonuclease 1	ATRIP; TREX1	0,97
SMG1 phosphatidylinositol 3-kinase-related kinase	SMG1	0,97
solute carrier family 23, member 3	SLC23A3	0,97
glutamate receptor, ionotropic, delta 1	GRID1	0,97
myotubularin related protein 3	MTMR3	0,97
CWF19-like 2, cell cycle control (S. pombe)	CWF19L2	0,97
zinc finger protein 487	ZNF487	0,97
single-pass membrane protein with coiled-coil domains 3	SMCO3	0,97
ubiquitin specific peptidase 9, Y-linked; testis-specific transcript, Y-linked 15 (non-protein coding)	USP9Y; TTTY15	0,97
keratin associated protein 17-1	KRTAP17-1	0,97
NHL repeat containing 4; phosphatidylinositol glycan anchor biosynthesis class Q	NHLRC4; PIGQ	0,97
growth associated protein 43	GAP43	0,97
WD repeat domain 86	WDR86	0,97
chromosome 22 open reading frame 31	C22orf31	0,97
Transcript Identified by AceView, Entrez Gene ID(s) 23355	VPS8	0,97
C-type lectin domain family 6, member A	CLEC6A	0,97

coiled-coil domain containing 189	CCDC189	0,97
four and a half LIM domains 5	FHL5	0,97
ring finger protein 186	RNF186	0,97
cadherin 19, type 2	CDH19	0,97
OMA1 zinc metallopeptidase	OMA1	0,97
uncharacterized LOC401052; Salzman2013 ANNOTATED, coding, INTERNAL, OVERLAPTX, OVEXON, UTR5 best transcript NM_001008737; novel transcript; Transcript Identified by AceView, Entrez Gene ID(s) 401052; 55831	LOC401052; AC022007.5; TMEM111	0,97
olfactory receptor, family 10, subfamily P, member 1	OR10P1	0,97
chromosome 9 open reading frame 139	C9orf139	0,97
inositol polyphosphate-5-phosphatase D	INPP5D	0,97
ATPase, Na+/K+ transporting, alpha 3 polypeptide	ATP1A3	0,97
fructose-1,6-bisphosphatase 1	FBP1	0,97
tensin 1	TNS1	0,97
progesterone immunomodulatory binding factor 1	PIBF1	0,97
LRP2 binding protein	LRP2BP	0,97
dual specificity tyrosine-(Y)-phosphorylation regulated kinase 1B	DYRK1B	0,97
tripartite motif containing 11; microRNA 6742	TRIM11; MIR6742	0,97
aldehyde dehydrogenase 1 family, member A1	ALDH1A1	0,97
secreted frizzled-related protein 1	SFRP1	0,97
GTPase, IMAP family member 8	GIMAP8	0,97
islet amyloid polypeptide	IAPP	0,97
leucine zipper, down-regulated in cancer 1	LDOC1	0,97
zinc finger, SWIM-type containing 7	ZSWIM7	0,97
ribosomal protein S6 kinase, 90kDa, polypeptide 1	RPS6KA1	0,97
transmembrane protein 210	TMEM210	0,97
SH3 and cysteine rich domain 2	STAC2	0,97
excision repair cross-complementation group 6; ERCC6-PGBD3 readthrough; piggyBac transposable element derived 3	ERCC6; ERCC6-PGBD3; PGBD3	0,97
serine/arginine-rich splicing factor 6	SRSF6	0,97
keratin associated protein 9-4	KRTAP9-4	0,97
bone morphogenetic protein 2	BMP2	0,97
heme oxygenase 2	HMOX2	0,97
FUN14 domain containing 1	FUNDC1	0,97
fibroblast growth factor binding protein 3	FGFBP3	0,97
proline rich 29	PRR29	0,97
CCAAT/enhancer binding protein (C/EBP), epsilon	CEBPE	0,97
odorant binding protein 2B	OBP2B	0,97
FGR proto-oncogene, Src family tyrosine kinase	FGR	0,97
myosin, heavy chain 7, cardiac muscle, beta	MYH7	0,97

signal transducer and activator of transcription 4	STAT4	0,97
DNA (cytosine-5-)-methyltransferase 3-like	DNMT3L	0,97
heart and neural crest derivatives expressed 2	HAND2	0,97
dipeptidyl-peptidase 3	DPP3	0,97
tolloid like 1	TLL1	0,97
teratocarcinoma-derived growth factor 1	TDGF1	0,97
sirtuin 6	SIRT6	0,97
G protein-coupled receptor 37 like 1	GPR37L1	0,96
methyltransferase like 2A	METTL2A	0,96
kelch-like family member 30	KLHL30	0,96
adrenoceptor beta 2, surface	ADRB2	0,96
olfactory receptor, family 1, subfamily I, member 1	OR1I1	0,96
solute carrier family 16, member 11	SLC16A11	0,96
parathyroid hormone	PTH	0,96
Zhang2013 ALT_ACCEPTOR, ALT_DONOR, coding, INTERNAL, intronic best transcript NM_001127	AP1B1	0,96
solute carrier family 35, member A4	SLC35A4	0,96
calcium channel, voltage-dependent, R type, alpha 1E subunit	CACNA1E	0,96
tyrosine 3-monooxygenase/tryptophan 5-monooxygenase activation protein, epsilon	YWHAE	0,96
interferon, alpha 13	IFNA13	0,96
ankyrin repeat domain 7	ANKRD7	0,96
interleukin 25	IL25	0,96
gamma-aminobutyric acid (GABA) A receptor, delta	GABRD	0,96
zinc finger, DHHC-type containing 11B	ZDHHC11B	0,96
keratin 78, type II	KRT78	0,96
carbohydrate (N-acetylglucosamine 6-O) sulfotransferase 5	CHST5	0,96
adhesion G protein-coupled receptor L1	ADGRL1	0,96
dynein, axonemal, heavy chain 9	DNAH9	0,96
coiled-coil domain containing 144A	CCDC144A	0,96
serine palmitoyltransferase, long chain base subunit 2	SPTLC2	0,96
cholinergic receptor, nicotinic alpha 10; nucleoporin 98kDa	CHRNA10; NUP98	0,96
zona pellucida glycoprotein 4	ZP4	0,96
sterile alpha motif domain containing 13	SAMD13	0,96
ankyrin repeat domain 24	ANKRD24	0,96
cullin 3	CUL3	0,96
long intergenic non-protein coding RNA 1205	LINC01205	0,96
myosin, heavy chain 1, skeletal muscle, adult	MYH1	0,96
contactin 4	CNTN4	0,96
chromosome 14 open reading frame 178	C14orf178	0,96
ankyrin repeat domain 34B	ANKRD34B	0,96
tumor necrosis factor receptor superfamily, member 8	TNFRSF8	0,96

glucose-6-phosphatase, catalytic, 2	G6PC2	0,96
POU6F2 antisense RNA 2 [Source:HGNC Symbol;Acc:HGNC:21887]; Salzman2013 ANTISENSE, coding, INTERNAL, intronic, OVERLAPTX best transcript NM_007252; novel transcript, antisense to POU6F2	POU6F2-AS2; POU6F2; AC011292.2	0,96
uncharacterized LOC338797; novel transcript	LOC338797; RP13-507P19.2; RP13-507P19.1	0,96
Rh blood group, D antigen	RHD	0,96
ribonuclease P/MRP 14kDa subunit	RPP14	0,96
potassium channel tetramerization domain containing 9	KCTD9	0,96
disco-interacting protein 2 homolog B	DIP2B	0,96
katanin p80 (WD repeat containing) subunit B 1	KATNB1	0,96
CD48 molecule	CD48	0,96
intercellular adhesion molecule 3	ICAM3	0,96
T-cell acute lymphocytic leukemia 2	TAL2	0,96
FERM and PDZ domain containing 3	FRMPD3	0,96
growth hormone 2	GH2	0,96
LIM homeobox 2	LHX2	0,96
protein tyrosine phosphatase, receptor type, E	PTPRE	0,96
dual specificity tyrosine-(Y)-phosphorylation regulated kinase 1B	DYRK1B	0,96
Memczak2013 ANTISENSE, coding, INTERNAL, intronic best transcript NM_033360	KRAS	0,96
mutS homolog 4	MSH4	0,96
fucosyltransferase 4 (alpha (1,3) fucosyltransferase, myeloid-specific)	FUT4	0,96
thrombomodulin	THBD	0,96
transmembrane 7 superfamily member 3	TM7SF3	0,96
APOC4-APOC2 readthrough (NMD candidate); apolipoprotein C-II	APOC4-APOC2; APOC2	0,96
keratin 77, type II	KRT77	0,96
myosin IB	MYO1B	0,96
prokineticin receptor 2	PROKR2	0,96
Homo sapiens polyhomeotic homolog 1 (Drosophila), mRNA (cDNA clone MGC:87926 IMAGE:5788132), complete cds.; Homo sapiens polyhomeotic homolog 1 (Drosophila), mRNA (cDNA clone MGC:189745 IMAGE:9057069), complete cds.	PHC1	0,96
glutamate receptor, metabotropic 5	GRM5	0,96
fatty acid binding protein 9, testis	FABP9	0,96
dynamin 2	DNM2	0,96
formin 1	FMN1	0,96
schlafen family member 12	SLFN12	0,96
zinc finger protein 705C, pseudogene	ZNF705CP	0,96
gamma-aminobutyric acid (GABA) A receptor, pi	GABRP	0,96

Charcot-Leyden crystal galectin	CLC	0,96
EPH receptor B3	EPHB3	0,96
cytokine receptor-like factor 2	CRLF2	0,96
enolase 1, (alpha)	ENO1	0,96
replication protein A1	RPA1	0,96
zinc finger protein 671	ZNF671	0,96
phosphofurin acidic cluster sorting protein 2	PACS2	0,96
zinc finger protein 774	ZNF774	0,96
chimerin 2	CHN2	0,96
defensin, alpha 5, Paneth cell-specific	DEFA5	0,96
MOB kinase activator 2	MOB2	0,96
KRAB box domain containing 1	KRBOX1	0,96
phosphoglucomutase 2	PGM2	0,96
uncharacterized LOC497048; uncharacterized LOC497048 [Source:EntrezGene;Acc:497048]; putative novel transcript	KU-MEL-3; RP4-529N6.1	0,96
mitochondrial translational release factor 1	MTRF1	0,96
polyribonucleotide nucleotidyltransferase 1	PNPT1	0,96
CD33 molecule	CD33	0,96
chorionic gonadotropin, beta polypeptide 1	CGB1	0,96
G protein-coupled receptor 182	GPR182	0,96
nucleolar protein with MIF4G domain 1	NOM1	0,96
prepronociceptin	PNOC	0,96
zinc finger protein 154	ZNF154	0,96
sema domain, immunoglobulin domain (Ig), short basic domain, secreted, (semaphorin) 3E	SEMA3E	0,96
DPP9 antisense RNA 1	DPP9-AS1	0,96
transmembrane protein 81	TMEM81	0,96
immunoglobulin superfamily, member 22	IGSF22	0,96
olfactory receptor, family 2, subfamily L, member 13	OR2L13	0,96
protease, serine 27	PRSS27	0,96
membrane-spanning 4-domains, subfamily A, member 7	MS4A7	0,96
sodium channel, non voltage gated 1 beta subunit	SCNN1B	0,96
keratin associated protein 4-3	KRTAP4-3	0,96
membrane metallo-endopeptidase	MME	0,96
guanylate cyclase activator 1A (retina)	GUCA1A	0,96
ELOVL fatty acid elongase 3	ELOVL3	0,96
chorionic somatomammotropin hormone 1 (placental lactogen)	CSH1	0,96
chromosome 1 open reading frame 195	C1orf195	0,96
transmembrane 4 L six family member 20	TM4SF20	0,96
CD28 molecule	CD28	0,96
small G protein signaling modulator 1	SGSM1	0,96

wingless-type MMTV integration site family, member 10A	WNT10A	0,96
olfactory receptor, family 4, subfamily C, member 11	OR4C11	0,96
solute carrier family 16 (monocarboxylate transporter), member 1	SLC16A1	0,96
TBC1D22A antisense RNA 1	TBC1D22A-AS1	0,96
transcription elongation factor B polypeptide 3C (elongin A3)	TCEB3C	0,96
heat shock 10kDa protein 1	HSPE1	0,96
prohibitin 2; small Cajal body-specific RNA 12	PHB2; SCARNA12	0,96
BTB and CNC homology 1, basic leucine zipper transcription factor 2	BACH2	0,96
chromosome 1 open reading frame 185	C1orf185	0,96
ALX homeobox 3	ALX3	0,96
small nuclear ribonucleoprotein D1 polypeptide	SNRPD1	0,96
HORMA domain containing 1	HORMAD1	0,96
Memczak2013 ALT_ACCEPTOR, ALT_DONOR, INTERNAL, intronic, ncRNA best transcript NR_023348	GAS8	0,96
interleukin 15 receptor, alpha	IL15RA	0,96
Homo sapiens uncharacterized LOC100133669 (LOC100133669), long non-coding RNA.; Transcript Identified by AceView, Entrez Gene ID(s) 100133669; novel transcript	LOC100133669; RP11-273G15.2	0,96
tetratricopeptide repeat domain 9B	TTC9B	0,96
uncharacterized LOC105377348; Salzman2013 ANTISENSE, coding, INTERNAL, intronic, OVERLAPTX best transcript NM_181890; novel transcript, antisense UBE2D3; Transcript Identified by AceView	LOC105377348; RP11-10L12.4; UBE2D3; pugo	0,96
transition protein 2 (during histone to protamine replacement)	TNP2	0,96
MLX interacting protein	MLXIP	0,96
catenin (cadherin-associated protein), alpha 3	CTNNA3	0,96
H1 histone family, member 0	H1F0	0,96
uroplakin 3B	UPK3B	0,96
fibroblast growth factor 6	FGF6	0,96
chromosome 3 open reading frame 58	C3orf58	0,96
hedgehog interacting protein	HHIP	0,96
dystrotelin	DYTN	0,96
gremlin 1, DAN family BMP antagonist	GREM1	0,96
sema domain, immunoglobulin domain (Ig), transmembrane domain (TM) and short cytoplasmic domain, (semaphorin) 4F	SEMA4F	0,96
GRB2 associated regulator of MAPK1 2	GAREM2	0,96
prokineticin receptor 1	PROKR1	0,96
A kinase (PRKA) anchor protein 10	AKAP10	0,96

kynurenine 3-monooxygenase (kynurenine 3-hydroxylase)	KMO	0,96
coagulation factor XII (Hageman factor)	F12	0,96
component of oligomeric golgi complex 5	COG5	0,96
cholinergic receptor, muscarinic 3	CHRM3	0,96
chromosome 17 open reading frame 98	C17orf98	0,96
calcium channel, voltage-dependent, beta subunit associated regulatory protein	CBARP	0,96
apolipoprotein O-like	APOOL	0,96
F11 receptor	F11R	0,96
PR domain containing 14	PRDM14	0,96
heterogeneous nuclear ribonucleoprotein H3 (2H9)	HNRNPH3	0,96
interleukin 2 receptor, alpha	IL2RA	0,96
Holliday junction recognition protein	HJURP	0,96
cytochrome c oxidase subunit I	COX1	0,96
glucagon-like peptide 1 receptor	GLP1R	0,96
uncharacterized LOC400558; Transcript Identified by AceView, Entrez Gene ID(s) 400558; novel transcript	LOC400558; RP11-46C24.3	0,96
psoriasis susceptibility 1 candidate 1	PSORS1C1	0,96
phospholipase A2, group IIC	PLA2G2C	0,96
Memczak2013 ANTISENSE, CDS, coding, INTERNAL best transcript NM_007277	EXOC3	0,96
Transcript Identified by AceView, Entrez Gene ID(s) 9361	LONP1	0,96
proprotein convertase subtilisin/kexin type 1	PCSK1	0,96
calcium binding protein 4	CABP4	0,96
phosphatase and actin regulator 1	PHACTR1	0,96
bromodomain containing 7	BRD7	0,96
coiled-coil domain containing 33	CCDC33	0,96
high mobility group box 4	HMGB4	0,96
unc-13 homolog C (C. elegans)	UNC13C	0,96
Transcript Identified by AceView, Entrez Gene ID(s) 23040	MYT1L	0,96
KH domain containing 3-like, subcortical maternal complex member	KHDC3L	0,96
leukocyte immunoglobulin-like receptor, subfamily A (with TM domain), member 5	LILRA5	0,96
insulin-degrading enzyme	IDE	0,96
zona pellucida binding protein 2	ZPBP2	0,96
olfactory receptor, family 51, subfamily J, member 1 (gene/pseudogene)	OR51J1	0,96
SR-related CTD-associated factor 1	SCAF1	0,96
ribosomal protein L41 pseudogene 2	RPL41P2	0,96
regulator of G-protein signaling 8	RGS8	0,96
NLR family, pyrin domain containing 8	NLRP8	0,96

major histocompatibility complex, class II, DP beta 1	HLA-DPB1	0,96
zinc finger, MYND-type containing 10	ZMYND10	0,96
Transcript Identified by AceView, Entrez Gene ID(s) 2066	ERBB4	0,96
IQ motif containing with AAA domain 1 like	IQCA1L	0,96
GTPase, IMAP family member 4	GIMAP4	0,96
Jeck2013 ALT_ACCEPTOR, ALT_DONOR, coding, INTERNAL, intronic best transcript NM_005270	GLI2	0,96
somatostatin receptor 4	SSTR4	0,96
zinc finger protein 540; ZNF571 antisense RNA 1	ZNF540; ZNF571-AS1	0,96
solute carrier family 39 (zinc transporter), member 2	SLC39A2	0,96
leucine-rich repeats and calponin homology (CH) domain containing 3	LRCH3	0,96
DIRAS family, GTP-binding RAS-like 2	DIRAS2	0,96
nucleosome assembly protein 1-like 5	NAP1L5	0,96
radial spoke head 14 homolog (Chlamydomonas)	RSPH14	0,96
multiple PDZ domain protein	MPDZ	0,96
calcyclin binding protein	CACYBP	0,96
major histocompatibility complex, class II, DQ beta 1	HLA-DQB1	0,96
RAS (RAD and GEM)-like GTP-binding 1	REM1	0,96
chromosome 2 open reading frame 88	C2orf88	0,96
bone morphogenetic protein 8a	BMP8A	0,96
thymus, brain and testes associated	TBATA	0,96
cadherin 12, type 2 (N-cadherin 2)	CDH12	0,96
Rh blood group, CcEe antigens	RHCE	0,96
death effector domain containing	DEDD	0,96
iron-sulfur cluster assembly enzyme	ISCU	0,96
phospholipase C, eta 2	PLCH2	0,96
mago homolog B, exon junction complex core component	MAGOHB	0,96
serine peptidase inhibitor, Kunitz type, 2	SPINT2	0,96
RAB19, member RAS oncogene family	RAB19	0,96
olfactory receptor, family 2, subfamily T, member 1	OR2T1	0,96
chromosome 15 open reading frame 53	C15orf53	0,96
Rho guanine nucleotide exchange factor 38	ARHGEF38	0,96
nicotinamide phosphoribosyltransferase	NAMPT	0,96
transcription elongation factor A (SII)-like 2	TCEAL2	0,96
mex-3 RNA binding family member C	MEX3C	0,96
PRAME family member 8	PRAMEF8	0,96
chromosome 5 open reading frame 47	C5orf47	0,96
bone morphogenetic protein 10	BMP10	0,96
tryptase gamma 1	TPSG1	0,96
K(lysine) acetyltransferase 6B	KAT6B	0,96
aldo-keto reductase family 1, member C4	AKR1C4	0,96

C-type lectin domain family 2, member D	CLEC2D	0,96
olfactory receptor, family 5, subfamily M, member 3	OR5M3	0,96
p53-responsive gene 1	PRG1	0,96
ADAM metallopeptidase domain 8	ADAM8	0,96
ribosomal protein S18	RPS18	0,96
multiple EGF-like-domains 6	MEGF6	0,96
BMP binding endothelial regulator	BMPER	0,96
interleukin 22	IL22	0,96
potassium channel, voltage gated Shaw related subfamily C, member 4	KCNC4	0,96
urotensin 2	UTS2	0,96
CCCTC-binding factor (zinc finger protein)	CTCF	0,96
inositol polyphosphate-5-phosphatase D	INPP5D	0,96
X antigen family, member 2	XAGE2	0,96
chromosome 4 open reading frame 17	C4orf17	0,96
Transcript Identified by AceView, Entrez Gene ID(s) 3707	ITPKB	0,96
TSEN15 tRNA splicing endonuclease subunit	TSEN15	0,96
neurturin	NRTN	0,96
olfactory receptor, family 5, subfamily T, member 1	OR5T1	0,96
zinc finger with KRAB and SCAN domains 8	ZKSCAN8	0,96
Transcript Identified by AceView, Entrez Gene ID(s) 79041	TMEM38A	0,96
peptidylprolyl isomerase A (cyclophilin A)	PPIA	0,96
solute carrier family 28 (concentrative nucleoside transporter), member 2	SLC28A2	0,96
calpain 14	CAPN14	0,96
X antigen family, member 1B; X antigen family, member 1E	XAGE1B; XAGE1E	0,96
interleukin 12 receptor, beta 1	IL12RB1	0,96
sialic acid binding Ig-like lectin 7	SIGLEC7	0,96
NADH dehydrogenase (ubiquinone) 1 alpha subcomplex, 6, 14kDa	NDUFA6	0,96
IQ motif containing C	IQCC	0,96
GC-rich promoter binding protein 1	GPBP1	0,96
ERGIC and golgi 3	ERGIC3	0,96
mediator complex subunit 13	MED13	0,96
myosin XVA	MYO15A	0,96
WW domain containing adaptor with coiled-coil	WAC	0,96
leukocyte cell derived chemotaxin 1	LECT1	0,96
olfactory receptor, family 1, subfamily M, member 1	OR1M1	0,96
calcium release activated channel regulator 2A	CRACR2A	0,96
tumor necrosis factor receptor superfamily, member 10d, decoy with truncated death domain	TNFRSF10D	0,96

solute carrier family 25 (S-adenosylmethionine carrier), member 26	SLC25A26	0,96
insulin-like growth factor 2 mRNA binding protein 1	IGF2BP1	0,96
metallothionein-like 5, testis-specific (tesmin)	MTL5	0,96
keratin associated protein 9-7	KRTAP9-7	0,96
MT-RNR2-like 6	MTRNR2L6	0,96
even-skipped homeobox 2	EVX2	0,96
DEAD (Asp-Glu-Ala-Asp) box polypeptide 28	DDX28	0,96
olfactory receptor, family 6, subfamily B, member 2	OR6B2	0,96
protein phosphatase 2, regulatory subunit B, delta	PPP2R2D	0,96
prenylcysteine oxidase 1 like	PCYOX1L	0,96
par-6 family cell polarity regulator alpha	PARD6A	0,96
nuclear fragile X mental retardation protein interacting protein 2	NUFIP2	0,96
Transcript Identified by AceView, Entrez Gene ID(s) 6764	ST5	0,96
protease, serine, 48	PRSS48	0,96
Ras-like without CAAX 2	RIT2	0,96
KIAA1549	KIAA1549	0,96
glucosaminyl (N-acetyl) transferase 6 [Source:HGNC Symbol;Acc:HGNC:21623]	GCNT6	0,96
glutaredoxin, cysteine rich 1	GRXCR1	0,96
pogo transposable element with ZNF domain	POGZ	0,96
X-linked Kx blood group related, Y-linked 2; X-linked Kx blood group related, Y-linked	XKRY2; XKRY	0,96
CD160 molecule	CD160	0,96
Memczak2013 ANTISENSE, CDS, coding, INTERNAL, UTR3 best transcript NM_001127457	CRY2	0,96
retinoblastoma binding protein 8; microRNA 4741	RBBP8; MIR4741	0,96
histone cluster 1, H4l	HIST1H4L	0,96
phosphatidylinositol-4-phosphate 3-kinase, catalytic subunit type 2 alpha	PIK3C2A	0,96
TBC1 domain family, member 3L	TBC1D3L	0,96
cerebellin 1 precursor	CBLN1	0,96
colipase-like 2	CLPSL2	0,96
peptidylprolyl isomerase (cyclophilin)-like 2	PPIL2	0,96
replication factor C subunit 2	RFC2	0,96
olfactory receptor, family 5, subfamily A, member 1	OR5A1	0,96
ring finger protein 138, E3 ubiquitin protein ligase	RNF138	0,96
olfactory receptor, family 6, subfamily T, member 1	OR6T1	0,96
FIC domain containing	FICD	0,96
calcium channel, voltage-dependent, alpha 2/delta subunit 3	CACNA2D3	0,96
karyopherin alpha 5 (importin alpha 6)	KPNA5	0,96
cystatin D	CST5	0,96

T-box 5	TBX5	0,96
toll-like receptor 1	TLR1	0,96
chromosome X open reading frame 51A; chromosome X open reading frame 51B	CXorf51A; CXorf51B	0,96
glycerophosphodiester phosphodiesterase domain containing 2	GDPD2	0,96
collagen, type VI, alpha 5	COL6A5	0,96
gamma-aminobutyric acid (GABA) A receptor, rho 1	GABRR1	0,96
neuronal pentraxin I	NPTX1	0,96
ovo-like zinc finger 1	OVOL1	0,96
Transcript Identified by AceView, Entrez Gene ID(s) 23670	TMEM2	0,96
transmembrane protein 116	TMEM116	0,96
zinc finger protein 775	ZNF775	0,96
nephrosis 2, idiopathic, steroid-resistant (podocin)	NPHS2	0,96
dynein regulatory complex subunit 3	DRC3	0,96
chaperonin containing TCP1, subunit 8 (theta)	CCT8	0,96
Zic family member 1	ZIC1	0,96
DNA-damage-inducible transcript 4-like	DDIT4L	0,96
STAU2 antisense RNA 1	STAU2-AS1	0,96
basic charge, Y-linked, 2C	BPY2C	0,96
NADH dehydrogenase (ubiquinone) 1 alpha subcomplex, 6, 14kDa	NDUFA6	0,96
sterile alpha motif domain containing 3	SAMD3	0,96
protein-O-mannosyltransferase 1	POMT1	0,96
olfactory receptor, family 2, subfamily A, member 1	OR2A1	0,96
RAB40A, member RAS oncogene family	RAB40A	0,96
ADAM metallopeptidase with thrombospondin type 1 motif 20	ADAMTS20	0,96
component of oligomeric golgi complex 7	COG7	0,96
taxilin beta	TXLNB	0,96
capping protein (actin filament) muscle Z-line, alpha 3	CAPZA3	0,96
fibrous sheath CABYR binding protein	FSCB	0,96
keratin associated protein 19-2	KRTAP19-2	0,96
uridine phosphorylase 2	UPP2	0,96
oligodendrocytic myelin paranodal and inner loop protein	OPALIN	0,96
ankyrin repeat and SOCS box containing 12	ASB12	0,96
carboxypeptidase A2 (pancreatic)	CPA2	0,96
zinc finger protein 543	ZNF543	0,95
chromosome 1 open reading frame 111	C1orf111	0,95
potassium channel, calcium activated large conductance subfamily M alpha, member 1	KCNMA1	0,95
odorant binding protein 2A	OBP2A	0,95
outer dense fiber of sperm tails 2	ODF2	0,95

tubulin, alpha 1c	TUBA1C	0,95
Transcript Identified by AceView, Entrez Gene ID(s) 146167	SLC38A8	0,95
peptidylprolyl isomerase (cyclophilin)-like 3	PPIL3	0,95
CUB domain containing protein 2	CDCP2	0,95
protein phosphatase 1, regulatory subunit 42	PPP1R42	0,95
Memczak2013 ALT_ACCEPTOR, ALT_DONOR, coding, INTERNAL, intronic best transcript NM_001003800	BICD2	0,95
fibrillin 1	FBN1	0,95
chromosome 22 open reading frame 39; histone cell cycle regulator	C22orf39; HIRA	0,95
chromosome 3 open reading frame 36	C3orf36	0,95
lactate dehydrogenase C	LDHC	0,95
Rap guanine nucleotide exchange factor 1	RAPGEF1	0,95
double homeobox 4	DUX4	0,95
F-box and leucine-rich repeat protein 6	FBXL6	0,95
inhibin beta A	INHBA	0,95
Memczak2013 ANTISENSE, coding, intronic, upstream_start, UTR5 best transcript NM_001161357	FCHO1	0,95
TP53 target 3D	TP53TG3D	0,95
uncharacterized LOC100130705; putative novel transcript	LOC100130705; RP11-309L24.4	0,95
Alport syndrome, mental retardation, midface hypoplasia and elliptocytosis chromosomal region gene 1	AMMECR1	0,95
PX domain containing serine/threonine kinase	PXK	0,95
potassium channel, inwardly rectifying subfamily J, member 16	KCNJ16	0,95
goosecoid homeobox 2	GSC2	0,95
uncharacterized LOC101929319; Salzman2013 ANTISENSE, CDS, coding, INTERNAL, intronic, OVCODE, OVEXON best transcript NM_007115	LOC101929319; TNFAIP6	0,95
olfactory receptor, family 2, subfamily A, member 12	OR2A12	0,95
hemoglobin, beta	HBB	0,95
staufen double-stranded RNA binding protein 1	STAU1	0,95
MX dynamin-like GTPase 2	MX2	0,95
ring finger protein 212	RNF212	0,95
small EDRK-rich factor 1B (centromeric); small EDRK-rich factor 1A (telomeric)	SERF1B; SERF1A	0,95
small EDRK-rich factor 1A (telomeric)	SERF1A	0,95
geminin coiled-coil domain containing	GMNC	0,95
RAS protein activator like 1 (GAP1 like)	RASAL1	0,95
group-specific component (vitamin D binding protein)	GC	0,95
ankyrin repeat domain 37	ANKRD37	0,95
prion protein (testis specific)	PRNT	0,95

trace amine associated receptor 8	TAAR8	0,95
protein kinase, cAMP-dependent, catalytic, gamma	PRKACG	0,95
Memczak2013 ALT_ACCEPTOR, ALT_DONOR, coding, INTERNAL, intronic best transcript NM_018717	MAML3	0,95
chromosome 18 open reading frame 65	C18orf65	0,95
MAP3K12 binding inhibitory protein 1	MBIP	0,95
NADH dehydrogenase (ubiquinone) 1 alpha subcomplex, 8, 19kDa	NDUFA8	0,95
cholinergic receptor, muscarinic 1	CHRM1	0,95
opioid receptor, kappa 1	OPRK1	0,95
RANBP2-like and GRIP domain containing 1; RANBP2-like and GRIP domain containing 2	RGPD1; RGPD2	0,95
Rap guanine nucleotide exchange factor like 1	RAPGEFL1	0,95
small integral membrane protein 6	SMIM6	0,95
secretoglobin, family 3A, member 2	SCGB3A2	0,95
actin-related protein T3	ACTRT3	0,95
myosin binding protein C, cardiac	MYBPC3	0,95
transmembrane protein 200C	TMEM200C	0,95
chromosome 1 open reading frame 234	C1orf234	0,95
transcription elongation factor A (SII)-like 3	TCEAL3	0,95
phosphatidylinositol-4-phosphate 3-kinase, catalytic subunit type 2 gamma	PIK3C2G	0,95
phosphatidylinositol transfer protein, membrane-associated 2	PITPNM2	0,95
mitochondrial antiviral signaling protein	MAVS	0,95
zinc finger protein 93	ZNF93	0,95
GDNF family receptor alpha like	GFRAL	0,95
keratin associated protein 19-3	KRTAP19-3	0,95
integrator complex subunit 3	INTS3	0,95
olfactory receptor, family 51, subfamily F, member 2	OR51F2	0,95
YY1 transcription factor; microRNA 6764	YY1; MIR6764	0,95
beta-carotene oxygenase 2; ribosomal protein S12 pseudogene 21	BCO2; RPS12P21	0,95
deleted in esophageal cancer 1	DEC1	0,95
proline rich 32	PRR32	0,95
MKRN2 opposite strand	MKRN2OS	0,95
enolase family member 4	ENO4	0,95
zinc finger, CCHC domain containing 16	ZCCHC16	0,95
BR serine/threonine kinase 2	BRSK2	0,95
loricrin	LOR	0,95
energy homeostasis associated	ENHO	0,95
Rho guanine nucleotide exchange factor 19	ARHGEF19	0,95
glyceraldehyde-3-phosphate dehydrogenase, spermatogenic	GAPDHS	0,95
coiled-coil-helix-coiled-coil-helix domain containing 6	CHCHD6	0,95

hes family bHLH transcription factor 2	HES2	0,95
potassium channel, two pore domain subfamily K, member 5	KCNK5	0,95
family with sequence similarity 172, member A; POU domain class 5, transcription factor 2; microRNA 2277	FAM172A; POU5F2; MIR2277	0,95
olfactory receptor, family 1, subfamily A, member 2	OR1A2	0,95
family with sequence similarity 60, member A	FAM60A	0,95
cerebellar degeneration related protein 2	CDR2	0,95
reticulon 2	RTN2	0,95
DEAD (Asp-Glu-Ala-Asp) box polypeptide 31	DDX31	0,95
5-hydroxytryptamine (serotonin) receptor 4, G protein-coupled	HTR4	0,95
Fc fragment of IgG, low affinity IIIa, receptor (CD16a)	FCGR3A	0,95
uncharacterized LOC101927844; Transcript Identified by AceView	LOC101927844; serfawbu	0,95
Rhox homeobox family, member 2; Rhox homeobox family, member 2B	RHOXF2; RHOXF2B	0,95
alpha 1,3-galactosyltransferase 2	A3GALT2	0,95
zinc finger CCCH-type containing 15	ZC3H15	0,95
G protein-coupled receptor kinase 7	GRK7	0,95
T-box 4	TBX4	0,95
mex-3 RNA binding family member A	MEX3A	0,95
transmembrane protein 71	TMEM71	0,95
POU class 3 homeobox 3	POU3F3	0,95
adaptor-related protein complex 5, mu 1 subunit	AP5M1	0,95
solute carrier family 35, member F3	SLC35F3	0,95
mediator complex subunit 23	MED23	0,95
pygopus family PHD finger 1	PYGO1	0,95
zinc finger, BED-type containing 4	ZBED4	0,95
iroquois homeobox 1	IRX1	0,95
kallikrein 1	KLK1	0,95
lysyl oxidase-like 1	LOXL1	0,95
ST8 alpha-N-acetyl-neuraminide alpha-2,8-sialyltransferase 1	ST8SIA1	0,95
keratin associated protein 10-11; keratin associated protein 12-3; keratin associated protein 10-12	KRTAP10-11; KRTAP12-3; KRTAP10-12	0,95
zinc finger protein 140	ZNF140	0,95
5-nucleotidase domain containing 4	NT5DC4	0,95
primase and DNA directed polymerase	PRIMPOL	0,95
Transcript Identified by AceView, Entrez Gene ID(s) 387535	HCRP1	0,95
transforming growth factor beta receptor associated protein 1	TGFBRAP1	0,95

mitochondrial ribosomal protein L35	MRPL35	0,95
serologically defined colon cancer antigen 8	SDCCAG8	0,95
mannosyl (beta-1,4-)-glycoprotein beta-1,4-N-acetylglucosaminyltransferase	MGAT3	0,95
proline rich 3	PRR3	0,95
tripartite motif containing 69	TRIM69	0,95
age-related maculopathy susceptibility 2	ARMS2	0,95
transmembrane protein 212	TMEM212	0,95
RWD domain containing 3; transmembrane protein 56; TMEM56-RWDD3 readthrough	RWDD3; TMEM56; TMEM56-RWDD3	0,95
olfactory receptor, family 13, subfamily C, member 5	OR13C5	0,95
solute carrier family 25, member 38	SLC25A38	0,95
sperm adhesion molecule 1 (PH-20 hyaluronidase, zona pellucida binding)	SPAM1	0,95
sidekick cell adhesion molecule 2	SDK2	0,95
guanine nucleotide binding protein-like 1	GNL1	0,95
heterogeneous nuclear ribonucleoprotein C-like 2	HNRNPCL2	0,95
MSANTD3-TMEFF1 readthrough	MSANTD3-TMEFF1	0,95
peroxiredoxin 5	PRDX5	0,95
cyclin Y like 1; microRNA 4775	CCNYL1; MIR4775	0,95
NIMA-related kinase 7	NEK7	0,95
Transcript Identified by AceView, Entrez Gene ID(s) 285596	FAM153A	0,95
isocitrate dehydrogenase 3 (NAD+) alpha	IDH3A	0,95
testis expressed 14	TEX14	0,95
melanin-concentrating hormone receptor 1	MCHR1	0,95
F-box protein 16	FBXO16	0,95
zinc finger RNA binding protein 2	ZFR2	0,95
SRY box 17	SOX17	0,95
olfactory receptor, family 8, subfamily S, member 1	OR8S1	0,95
tubulin tyrosine ligase-like family member 4	TTLL4	0,95
tripartite motif-containing 51	TRIM51	0,95
calcium channel, voltage-dependent, gamma subunit 2	CACNG2	0,95
ubiquinol-cytochrome c reductase core protein I	UQCRC1	0,95
chromosome 5 open reading frame 64	C5orf64	0,95
aprataxin	APTX	0,95
carbonic anhydrase XI	CA11	0,95
divergent-paired related homeobox	DPRX	0,95
thrombospondin type 1 domain containing 7B	THSD7B	0,95
calcium channel, voltage-dependent, gamma subunit 1	CACNG1	0,95
zinc finger protein 536	ZNF536	0,95
ribulose-5-phosphate-3-epimerase-like 1	RPEL1	0,95

transporter 1, ATP-binding cassette, sub-family B (MDR/TAP)	TAP1	0,95
mindbomb E3 ubiquitin protein ligase 1	MIB1	0,95
V-set and transmembrane domain containing 5	VSTM5	0,95
BCL2-like 14 (apoptosis facilitator)	BCL2L14	0,95
UPF0607 protein ENSP00000381418-like; Putative UPF0607 protein ENSP00000381418 [Source:UniProtKB/Swiss-Prot;Acc:A8MU76]	LOC729159; RP11-457D20.2	0,95
G protein-coupled receptor 88	GPR88	0,95
RAD50 interactor 1	RINT1	0,95
solute carrier family 1 (glutamate transporter), member 7	SLC1A7	0,95
polycystic kidney disease 1-like 3	PKD1L3	0,95
SEC16 homolog A, endoplasmic reticulum export factor	SEC16A	0,95
EPH receptor B6	EPHB6	0,95
pinin, desmosome associated protein	PNN	0,95
msh homeobox 2	MSX2	0,95
ADAM metallopeptidase domain 33	ADAM33	0,95
Synthetic construct Homo sapiens clone IMAGE:100063377, MGC:190767 ribosomal protein L41 (RPL41) mRNA, encodes complete protein.	RPL41	0,95
calcitonin receptor	CALCR	0,95
mucin-like 1	MUCL1	0,95
forkhead box I3	FOXI3	0,95
V-set and immunoglobulin domain containing 8	VSIG8	0,95
interleukin 36, alpha	IL36A	0,95
homeobox B9	HOXB9	0,95
lymphocyte antigen 6 complex, locus G6D	LY6G6D	0,95
collagen, type IV, alpha 1	COL4A1	0,95
complement component (3b/4b) receptor 1-like	CR1L	0,95
leucine rich repeat containing 14	LRRC14	0,95
immunoglobulin (CD79A) binding protein 1	IGBP1	0,95
signaling lymphocytic activation molecule family member 1	SLAMF1	0,95
POM121 transmembrane nucleoporin C	POM121C	0,95
perilipin 1	PLIN1	0,95
keratin 7, type II	KRT7	0,95
fibroblast growth factor 14; FGF14 intronic transcript 1	FGF14; FGF14-IT1	0,95
teneurin transmembrane protein 2	TENM2	0,95
solute carrier organic anion transporter family, member 1B7 (non-functional); solute carrier organic anion transporter family, member 1B1	SLCO1B7; SLCO1B1	0,95
CTD nuclear envelope phosphatase 1	CTDNEP1	0,95
frizzled class receptor 8; microRNA 4683	FZD8; MIR4683	0,95
cullin 5	CUL5	0,95

keratin associated protein 20-4	KRTAP20-4	0,95
cation channel, sperm associated 4	CATSPER4	0,95
intelectin 2	ITLN2	0,95
R-spondin 1	RSPO1	0,95
KRR1, small subunit (SSU) processome component, homolog (yeast)	KRR1	0,95
solute carrier family 27 (fatty acid transporter), member 6	SLC27A6	0,95
archaelysin family metallopeptidase 1	AMZ1	0,95
kinesin family member 5C	KIF5C	0,95
apolipoprotein A-I binding protein	APOA1BP	0,95
cytochrome c oxidase subunit VIIIC	COX8C	0,95
epiphycan	EPYC	0,95
interferon, alpha 7	IFNA7	0,95
forkhead box H1	FOXH1	0,95
olfactory receptor, family 6, subfamily K, member 6	OR6K6	0,95
phospholipase C, delta 4	PLCD4	0,95
phosphatidylinositol glycan anchor biosynthesis class X	PIGX	0,95
gap junction protein beta 4	GJB4	0,95
INO80 complex subunit	INO80	0,95
integrin alpha D	ITGAD	0,95
RAB11A, member RAS oncogene family	RAB11A	0,95
Nanog homeobox	NANOG	0,95
lipase, family member K	LIPK	0,95
EGF containing fibulin-like extracellular matrix protein 1	EFEMP1	0,95
CD99 molecule-like 2	CD99L2	0,95
insulin induced gene 1	INSIG1	0,95
raftlin family member 2	RFTN2	0,95
ADP-ribosyltransferase 3	ART3	0,95
ribosomal protein L36a-like	RPL36AL	0,95
mitochondrial ribosomal protein S5	MRPS5	0,95
phosphoinositide-3-kinase, regulatory subunit 4	PIK3R4	0,95
corticotropin releasing hormone binding protein	CRHBP	0,95
LY6/PLAUR domain containing 6B	LYPD6B	0,95
coagulation factor IX	F9	0,95
clusterin-like 1 (retinal)	CLUL1	0,95
GRB2-associated binding protein family, member 4	GAB4	0,95
potassium channel, voltage gated Shab related subfamily B, member 1	KCNB1	0,95
dihydrouridine synthase 1-like	DUS1L	0,95
adenosine monophosphate deaminase 3	AMPD3	0,95
chromosome 12 open reading frame 54	C12orf54	0,95
hemicentin 2	HMCN2	0,95
small proline-rich protein 2G	SPRR2G	0,95
collagen, type VII, alpha 1; microRNA 711	COL7A1; MIR711	0,95

adenosine kinase; mitochondrial ribosomal protein L35 pseudogene 3	ADK; MRPL35P3	0,95
uncharacterized LOC151121; novel transcript	LOC151121; AC079586.1	0,95
cAMP responsive element binding protein-like 2	CREBL2	0,95
even-skipped homeobox 1	EVX1	0,95
butyrobetaine (gamma), 2-oxoglutarate dioxygenase (gamma-butyrobetaine hydroxylase) 1	BBOX1	0,95
dehydrogenase/reductase (SDR family) member 4 like 2	DHRS4L2	0,95
solute carrier family 13 (sodium/sulfate symporter), member 1	SLC13A1	0,95
chloride channel, voltage-sensitive 4	CLCN4	0,95
matrix metallopeptidase 25	MMP25	0,95
PRAME family member 7	PRAMEF7	0,95
long intergenic non-protein coding RNA 269	LINC00269	0,95
EH domain binding protein 1 like 1	EHBP1L1	0,95
Transcript Identified by AceView, Entrez Gene ID(s) 91445	RNF185	0,95
glycoprotein VI (platelet)	GP6	0,95
pyroglutamyl-peptidase I-like	PGPEP1L	0,95
tenascin C	TNC	0,95
glycogenin 1	GYG1	0,95
glutamate receptor, metabotropic 1	GRM1	0,95
BAI1-associated protein 3	BAIAP3	0,95
leucine zipper protein 4	LUZP4	0,95
integrin alpha 4	ITGA4	0,95
nucleoporin 210kDa like; microRNA 5698	NUP210L; MIR5698	0,95
kelch-like family member 32	KLHL32	0,95
aurora kinase A and ninein interacting protein	AUNIP	0,95
FK506 binding protein 15	FKBP15	0,95
paired box 7	PAX7	0,95
leucine-rich repeats and guanylate kinase domain containing	LRGUK	0,95
solute carrier family 16 (monocarboxylate transporter), member 7	SLC16A7	0,95
Transcript Identified by AceView, Entrez Gene ID(s) 6596	HLTF	0,95
fibulin 2	FBLN2	0,95
caspase 2	CASP2	0,95
chromosome 10 open reading frame 2	C10orf2	0,95
myeloid-associated differentiation marker-like 2	MYADML2	0,95

SAGA complex associated factor 29 pseudogene; Transcript Identified by AceView, Entrez Gene ID(s) 388242; Uncharacterized protein [Source:UniProtKB/TrEMBL;Acc:H0YHM3]	LOC388242; LOC613038; RP11-345J4.3	0,95
microspherule protein 1	MCRS1	0,95
myeloid cell nuclear differentiation antigen	MNDA	0,95
ajuba LIM protein	AJUBA	0,95
transmembrane protein 151B	TMEM151B	0,95
Transcript Identified by AceView, Entrez Gene ID(s) 3561; putative novel transcript	RP5-1091N2.9; IL2RG	0,95
HEAT repeat containing 6	HEATR6	0,95
protein phosphatase 2, regulatory subunit B, beta	PPP2R5B	0,95
C2 calcium-dependent domain containing 4D	C2CD4D	0,95
kelch-like family member 13	KLHL13	0,95
Memczak2013 ANTISENSE, coding, INTERNAL, intronic best transcript NM_024829	PLBD1	0,95
hydroxymethylbilane synthase	HMBS	0,95
matrix metallopeptidase 28	MMP28	0,95
tripartite motif containing 27	TRIM27	0,95
keratin 14, type I	KRT14	0,95
osteoclast stimulatory transmembrane protein	OCSTAMP	0,95
Transcript Identified by AceView, Entrez Gene ID(s) 57498; 100216337	KIDINS220	0,95
cytochrome P450, family 26, subfamily C, polypeptide 1	CYP26C1	0,95
TLX1 neighbor	TLX1NB	0,95
synaptic vesicle glycoprotein 2B	SV2B	0,95
rhophilin associated tail protein 1B	ROPN1B	0,95
interleukin 13 receptor, alpha 2	IL13RA2	0,95
histidine rich carboxyl terminus 1	HRCT1	0,95
matrix metallopeptidase 16 (membrane-inserted)	MMP16	0,95
p21 protein (Cdc42/Rac)-activated kinase 4	PAK4	0,95
olfactory receptor, family 4, subfamily F, member 6	OR4F6	0,95
solute carrier family 25 (mitochondrial folate carrier), member 32	SLC25A32	0,95
insulin like growth factor binding protein 5	IGFBP5	0,95
TAR DNA binding protein	TARDBP	0,95
POTE ankyrin domain family, member B3	POTEB3	0,95
transmembrane protein 43	TMEM43	0,95
BOC cell adhesion associated, oncogene regulated	BOC	0,95
transmembrane protease, serine 15	TMPRSS15	0,95
myostatin	MSTN	0,95
tRNA-yW synthesizing protein 1 homolog B (S. cerevisiae)	TYW1B	0,95
cysteine/histidine-rich 1	CYHR1	0,95
G protein-coupled receptor 153	GPR153	0,95

connector enhancer of kinase suppressor of Ras 2	CNKSR2	0,95
leucyl/cystinyl aminopeptidase	LNPEP	0,95
melanoma inhibitory activity; RAB4B, member RAS oncogene family; MIA-RAB4B readthrough (NMD candidate)	MIA; RAB4B; MIA-RAB4B	0,95
G2/M-phase specific E3 ubiquitin protein ligase	G2E3	0,95
Jeck2013 ANTISENSE, CDS, coding, INTERNAL, OVCODE, OVEXON, UTR3 best transcript NM_001013	RPS9	0,95
LIM homeobox 9	LHX9	0,95
origin recognition complex subunit 1	ORC1	0,95
fascin actin-bundling protein 2, retinal	FSCN2	0,95
theg spermatid protein	THEG	0,95
sodium channel, voltage gated, type VII alpha subunit	SCN7A	0,95
schlafen family member 13	SLFN13	0,95
lysocardiolipin acyltransferase 1	LCLAT1	0,95
nuclear receptor subfamily 2, group C, member 2	NR2C2	0,95
CD300 molecule-like family member b	CD300LB	0,95
nascent polypeptide-associated complex alpha subunit	NACA	0,95
RAD51 associated protein 2	RAD51AP2	0,95
chemokine (C-C motif) ligand 13	CCL13	0,95
remodeling and spacing factor 1	RSF1	0,95
NIF3 NGG1 interacting factor 3-like 1	NIF3L1	0,95
histamine receptor H4	HRH4	0,95
RERG/RAS-like	RERGL	0,95
neuropilin (NRP) and tolloid (TLL)-like 1	NETO1	0,95
Wilms tumor 1 interacting protein	WTIP	0,95
coiled-coil domain containing 25	CCDC25	0,95
chloride channel accessory 2	CLCA2	0,95
ciliary neurotrophic factor receptor	CNTFR	0,95
cyclin H	CCNH	0,95
ribosomal protein S25	RPS25	0,95
arylacetamide deacetylase-like 3	AADACL3	0,95
trafficking protein particle complex 4	TRAPPC4	0,95
aspartate-rich 1	DRICH1	0,94
prolyl endopeptidase	PREP	0,94
hydroxymethylbilane synthase	HMBS	0,94
malic enzyme 3, NADP(+)-dependent, mitochondrial	ME3	0,94
zinc finger and BTB domain containing 1	ZBTB1	0,94
sec1 family domain containing 2	SCFD2	0,94
interleukin 17 receptor E-like	IL17REL	0,94
Homo sapiens thymosin beta 15B (TMSB15B), mRNA.; Thymosin beta-15B [Source:UniProtKB/Swiss-Prot;Acc:P0CG35]	TMSB15B; RP11-722G7.1	0,94
fms-related tyrosine kinase 3	FLT3	0,94
testis-specific serine kinase 4	TSSK4	0,94

CD276 molecule	CD276	0,94
guanylate cyclase 1, soluble, beta 3	GUCY1B3	0,94
ubiquitin-conjugating enzyme E2S; ubiquitin conjugating enzyme E2S pseudogene 1	UBE2S; UBE2SP1	0,94
partner and localizer of BRCA2	PALB2	0,94
Zic family member 4	ZIC4	0,94
zinc finger protein 347	ZNF347	0,94
tubulin tyrosine ligase-like family member 8	TTLL8	0,94
thyroid stimulating hormone, beta	TSHB	0,94
chromosome 3 open reading frame 79	C3orf79	0,94
aryl-hydrocarbon receptor nuclear translocator 2; microRNA 5572	ARNT2; MIR5572	0,94
olfactory receptor, family 5, subfamily AC, member 1 (gene/pseudogene)	OR5AC1	0,94
lysophosphatidylcholine acyltransferase 3	LPCAT3	0,94
Transcript Identified by AceView, Entrez Gene ID(s) 389362	PSMG4	0,94
ets variant 3-like	ETV3L	0,94
nuclear cap binding protein subunit 2-like	NCBP2L	0,94
protease, serine, 58	PRSS58	0,94
SH3-binding domain protein 5-like	SH3BP5L	0,94
zinc finger protein 207; microRNA 632	ZNF207; MIR632	0,94
structural maintenance of chromosomes 4	SMC4	0,94
centromere protein M	CENPM	0,94
MAS-related GPR, member F	MRGPRF	0,94
retinol dehydrogenase 16 (all-trans)	RDH16	0,94
ARP6 actin-related protein 6 homolog (yeast)	ACTR6	0,94
NK2 homeobox 4	NKX2-4	0,94
Zhang2013 ALT_ACCEPTOR, ALT_DONOR, coding, INTERNAL, intronic best transcript NM_004110	FDXR	0,94
ARP5 actin-related protein 5 homolog (yeast)	ACTR5	0,94
DEAD (Asp-Glu-Ala-Asp) box polypeptide 23	DDX23	0,94
potassium channel, voltage gated shaker related subfamily A, member 2	KCNA2	0,94
MANSC domain containing 1	MANSC1	0,94
low density lipoprotein receptor class A domain containing 2	LDLRAD2	0,94
solute carrier family 25 (mitochondrial thiamine pyrophosphate carrier), member 19	SLC25A19	0,94
MFNG O-fucosylpeptide 3-beta-N-acetylglucosaminyltransferase	MFNG	0,94
zinc finger protein 264	ZNF264	0,94
gametogenetin	GGN	0,94
inner membrane protein, mitochondrial	IMMT	0,94
TDP-glucose 4,6-dehydratase	TGDS	0,94

UDP-GlcNAc:betaGal beta-1,3-N-acetylglucosaminyltransferase 4	B3GNT4	0,94
crumbs family member 1, photoreceptor morphogenesis associated	CRB1	0,94
IQ motif containing E	IQCE	0,94
butyrophilin-like 2	BTNL2	0,94
solute carrier family 5 (sodium/glucose cotransporter), member 1	SLC5A1	0,94
RAD51 associated protein 1	RAD51AP1	0,94
family with sequence similarity 150, member A	FAM150A	0,94
general transcription factor IIIC subunit 6	GTF3C6	0,94
Jeck2013 ALT_ACCEPTOR, ALT_DONOR, coding, INTERNAL, intronic best transcript NM_006734	HIVEP2	0,94
Transcript Identified by AceView, Entrez Gene ID(s) 347088; adhesion G protein-coupled receptor D2 [Source:HGNC Symbol;Acc:HGNC:18651]	GPR144; ADGRD2	0,94
melanoma inhibitory activity family, member 3	MIA3	0,94
tenascin N	TNN	0,94
OTU domain containing 6B	OTUD6B	0,94
double homeobox A	DUXA	0,94
UDP-Gal:betaGlcNAc beta 1,4- galactosyltransferase, polypeptide 1	B4GALT1	0,94
leukocyte immunoglobulin-like receptor, subfamily A (with TM domain), member 1	LILRA1	0,94
erythrocyte membrane protein band 4.2	EPB42	0,94
radial spoke head 6 homolog A (Chlamydomonas)	RSPH6A	0,94
zinc finger protein 805	ZNF805	0,94
NEDD4 binding protein 3	N4BP3	0,94
interphotoreceptor matrix proteoglycan 2	IMPG2	0,94
filaggrin family member 2	FLG2	0,94
spectrin, beta, erythrocytic; microRNA 7855	SPTB; MIR7855	0,94
kallikrein related peptidase 11	KLK11	0,94
cytochrome c oxidase assembly factor 4 homolog	COA4	0,94
ELAV like neuron-specific RNA binding protein 4	ELAVL4	0,94
phenylalanyl-tRNA synthetase alpha subunit	FARSA	0,94
peptidylprolyl cis/trans isomerase, NIMA-interacting 4	PIN4	0,94
solute carrier family 9, subfamily C (Na+-transporting carboxylic acid decarboxylase), member 1	SLC9C1	0,94
Transcript Identified by AceView, Entrez Gene ID(s) 26051	PPP1R16B	0,94
muskelin 1, intracellular mediator containing kelch motifs	MKLN1	0,94
pyroglutamylated RFamide peptide	QRFP	0,94
coiled-coil domain containing 102A	CCDC102A	0,94
kelch-like family member 23	KLHL23	0,94

olfactory receptor, family 8, subfamily G, member 5	OR8G5	0,94
Eukaryotic translation initiation factor 3 subunit F [Source:UniProtKB/Swiss-Prot;Acc:O00303]	EIF3F	0,94
tectonin beta-propeller repeat containing 1	TECPR1	0,94
cilia and flagella associated protein 65	CFAP65	0,94
CD244 molecule, natural killer cell receptor 2B4	CD244	0,94
MACRO domain containing 2; ribosomal protein S3 pseudogene 1	MACROD2; RPS3P1	0,94
zinc finger protein 35	ZNF35	0,94
STEAP family member 4	STEAP4	0,94
colony stimulating factor 2 receptor, alpha, low-affinity (granulocyte-macrophage)	CSF2RA	0,94
Nipped-B homolog (Drosophila)	NIPBL	0,94
histamine receptor H2	HRH2	0,94
solute carrier family 14 (urea transporter), member 2	SLC14A2	0,94
v-akt murine thymoma viral oncogene homolog 3	AKT3	0,94
chromosome 12 open reading frame 29	C12orf29	0,94
mitochondrial ribosomal protein L45	MRPL45	0,94
proapoptotic nucleolar protein 1	PANO1	0,94
kinesin family member 27	KIF27	0,94
Transcript Identified by AceView, Entrez Gene ID(s) 23378	RRP8	0,94
synaptic vesicle glycoprotein 2A	SV2A	0,94
serine peptidase inhibitor, Kazal type 4	SPINK4	0,94
MAGE family member A3	MAGEA3	0,94
TP53 target 5	TP53TG5	0,94
pellino E3 ubiquitin protein ligase family member 3	PELI3	0,94
synapse differentiation inducing 1-like	SYNDIG1L	0,94
LMO7 downstream neighbor	LMO7DN	0,94
prothymosin, alpha	PTMA	0,94
NADH dehydrogenase (ubiquinone) complex I, assembly factor 5	NDUFAF5	0,94
tetratricopeptide repeat domain 7B	TTC7B	0,94
hydroxysteroid (17-beta) dehydrogenase 13	HSD17B13	0,94
GPN-loop GTPase 2	GPN2	0,94
synaptotagmin II	SYT2	0,94
protein phosphatase 1, regulatory (inhibitor) subunit 1A	PPP1R1A	0,94
leukotriene C4 synthase	LTC4S	0,94
gasdermin A	GSDMA	0,94
mitochondrial ribosome-associated GTPase 1	MTG1	0,94
retinoic acid early transcript 1E	RAET1E	0,94
sorcin	SRI	0,94
neuromedin U receptor 1	NMUR1	0,94
chimerin 2	CHN2	0,94
olfactory receptor, family 13, subfamily D, member 1	OR13D1	0,94

chromosome 15 open reading frame 48; microRNA 147b	C15orf48; MIR147B	0,94
lysosomal protein transmembrane 4 beta	LAPTM4B	0,94
transmembrane protein 110	TMEM110	0,94
dickkopf WNT signaling pathway inhibitor 4	DKK4	0,94
adducin 3 (gamma)	ADD3	0,94
goosecoid homeobox	GSC	0,94
neurobeachin like 1	NBEAL1	0,94
cannabinoid receptor 2	CNR2	0,94
Transcript Identified by AceView, Entrez Gene ID(s) 8425	LTBP4	0,94
signal transducing adaptor family member 1	STAP1	0,94
chromosome 10 open reading frame 131	C10orf131	0,94
neurexophilin and PC-esterase domain family, member 1	NXPE1	0,94
Homo sapiens XK, Kell blood group complex subunit-related, Y-linked 2 (XKRY2), mRNA.; Homo sapiens XK, Kell blood group complex subunit-related, Y-linked (XKRY), mRNA.; Homo sapiens XK, Kell blood group complex subunit-related, Y-linked, mRNA (cDNA clone MGC:164514 IMAGE:40146905), complete cds.; Homo sapiens XK, Kell blood group complex subunit-related, Y-linked, mRNA (cDNA clone MGC:164516 IMAGE:40146907), complete cds.; Homo sapiens XK, Kell blood group complex subunit-related, Y-linked 2, mRNA (cDNA clone MGC:164532 IMAGE:40146923), complete cds.; Homo sapiens XK, Kell blood group complex subunit-related, Y-linked 2, mRNA (cDNA clone MGC:164534 IMAGE:40146925), complete cds.; Synthetic construct Homo sapiens clone IMAGE:100015836, MGC:183189 XK, Kell blood group complex subunit-related, Y-linked 2 (XKRY2) mRNA, encodes complete protein.; Synthetic construct Homo sapiens clone IMAGE:100016528, MGC:184271 XK, Kell blood group complex subunit-related, Y-linked (XKRY) mRNA, encodes complete protein.; Transcript Identified by AceView, Entrez Gene ID(s) 353515, RefSeq ID(s) NM_004677, NM_001002906	XKRY2; XKRY	0,94
ovo-like zinc finger 3	OVOL3	0,94
Transcript Identified by AceView, Entrez Gene ID(s) 375775	PNPLA7	0,94
NRDE-2, necessary for RNA interference, domain containing	NRDE2	0,94
keratin 33A, type I	KRT33A	0,94
myelodysplastic syndrome 2 translocation associated	MDS2	0,94

leucine-rich repeat, immunoglobulin-like and transmembrane domains 1	LRIT1	0,94
peptidyl arginine deiminase, type VI	PADI6	0,94
zinc finger protein 735	ZNF735	0,94
Zhang2013 ALT_ACCEPTOR, ALT_DONOR, coding, INTERNAL, intronic best transcript NM_020338	ZMIZ1	0,94
G-patch domain and KOW motifs	GPKOW	0,94
keratocan	KERA	0,94
mitochondrial ribosomal protein L47	MRPL47	0,94
IQ motif containing F5 pseudogene; Transcript Identified by AceView, Entrez Gene ID(s) 401067	IQCF4; IQCF3	0,94
mucin 7, secreted	MUC7	0,94
pleckstrin homology domain containing, family A (phosphoinositide binding specific) member 4	PLEKHA4	0,94
potassium voltage-gated channel, modifier subfamily S, member 2	KCNS2	0,94
spectrin, alpha, erythrocytic 1	SPTA1	0,94
protein phosphatase 2, regulatory subunit B, gamma	PPP2R5C	0,94
neurogenin 3	NEUROG3	0,94
RNA binding region (RNP1, RRM) containing 3	RNPC3	0,94
cholinergic receptor, nicotinic alpha 4	CHRNA4	0,94
testis expressed 22	TEX22	0,94
ring finger protein 180	RNF180	0,94
Memczak2013 ANTISENSE, CDS, coding, INTERNAL best transcript NM_012335	MYO1F	0,94
protocadherin alpha 9; protocadherin alpha subfamily C, 1; protocadherin alpha subfamily C, 2; protocadherin alpha 1; protocadherin alpha 10; protocadherin alpha 11; protocadherin alpha 12; protocadherin alpha 13; protocadherin alpha 2; protocadherin alpha 3; protocadherin alpha 4; protocadherin alpha 5; protocadherin alpha 6; protocadherin alpha 7; protocadherin alpha 8; protocadherin alpha 14 pseudogene	PCDHA9; PCDHAC1; PCDHAC2; PCDHA1; PCDHA10; PCDHA11; PCDHA12; PCDHA13; PCDHA2; PCDHA3; PCDHA4; PCDHA5; PCDHA6; PCDHA7; PCDHA8; PCDHA14	0,94
lactate dehydrogenase A-like 6B	LDHAL6B	0,94
chromosome 6 open reading frame 223	C6orf223	0,94
mixed lineage kinase 4; Mitogen-activated protein kinase kinase kinase MLK4 [Source:UniProtKB/Swiss-Prot;Acc:Q5TCX8]; Salzman2013 ANNOTATED, CDS, coding, INTERNAL, OVCODE, OVEXON best transcript NM_032435	KIAA1804; MLK4	0,94

abhydrolase domain containing 12B	ABHD12B	0,94
chromosome 16 open reading frame 82	C16orf82	0,94
myozenin 1	MYOZ1	0,94
interferon-induced protein 44-like	IFI44L	0,94
endogenous retrovirus group MER34, member 1	ERVMER34-1	0,94
KIAA1456	KIAA1456	0,94
phosphatidylinositol 4-kinase type 2 beta	PI4K2B	0,94
kelch-like family member 15	KLHL15	0,94
nuclear receptor interacting protein 3	NRIP3	0,94
proteinase 3	PRTN3	0,94
family with sequence similarity 126, member A	FAM126A	0,94
uncharacterized LOC286238; Transcript Identified by AceView, Entrez Gene ID(s) 286238, RefSeq ID(s) NM_001100111; novel transcript	LOC286238; RP13-60M5.2	0,94
FRAS1 related extracellular matrix protein 2	FREM2	0,94
double homeobox 3	DUX3	0,94
chromosome 14 open reading frame 159	C14orf159	0,94
glycoprotein M6A	GPM6A	0,94
NLR family, pyrin domain containing 4	NLRP4	0,94
zinc finger protein 483	ZNF483	0,94
claudin 25	CLDN25	0,94
G protein-coupled receptor 21; RAB GTPase activating protein 1	GPR21; RABGAP1	0,94
transmembrane protein 78	TMEM78	0,94
androglobin	ADGB	0,94
zinc finger protein 780A	ZNF780A	0,94
ring finger protein 207	RNF207	0,94
olfactory receptor, family 1, subfamily P, member 1 (gene/pseudogene)	OR1P1	0,94
Spi-C transcription factor (Spi-1/PU.1 related)	SPIC	0,94
transmembrane protein with EGF-like and two follistatin-like domains 2	TMEFF2	0,94
LIM homeobox 8	LHX8	0,94
SECIS binding protein 2	SECISBP2	0,94
chromosome 21 open reading frame 58	C21orf58	0,94
zinc finger protein 784	ZNF784	0,94
GAR1 homolog, ribonucleoprotein	GAR1	0,94
copine VII	CPNE7	0,94
Jeck2013 ALT_ACCEPTOR, ALT_DONOR, coding, INTERNAL, intronic best transcript NM_058175	COL6A2	0,94
solute carrier family 12 (sodium/potassium/chloride transporter), member 1	SLC12A1	0,94
keratin associated protein 6-2	KRTAP6-2	0,94
alpha hemoglobin stabilizing protein	AHSP	0,94
F-box protein 24	FBXO24	0,94

uncharacterized LOC401357; novel piRNA host transcript	LOC401357; RP13-580B18.4	0,94
l(3)mbt-like 2 (Drosophila)	L3MBTL2	0,94
G protein-coupled receptor 34	GPR34	0,94
zinc finger protein 536	ZNF536	0,94
NADH dehydrogenase (ubiquinone) flavoprotein 1, 51kDa	NDUFV1	0,94
colony stimulating factor 2 receptor, alpha, low-affinity (granulocyte-macrophage)	CSF2RA	0,94
sema domain, immunoglobulin domain (Ig), short basic domain, secreted, (semaphorin) 3G	SEMA3G	0,94
zinc finger protein 676	ZNF676	0,94
novel transcript; Transcript Identified by AceView, Entrez Gene ID(s) 7099	RP11-281A20.2; TLR4	0,94
regulator of G-protein signaling 13	RGS13	0,94
cell division cycle associated 7-like	CDCA7L	0,94
torsin A interacting protein 2	TOR1AIP2	0,94
regulatory factor X, 4 (influences HLA class II expression)	RFX4	0,94
microfibrillar associated protein 1	MFAP1	0,94
interleukin 17 receptor A	IL17RA	0,94
interferon (alpha, beta and omega) receptor 1	IFNAR1	0,94
trichoplein, keratin filament binding	TCHP	0,94
complement factor H-related 2	CFHR2	0,94
cleavage and polyadenylation specific factor 3	CPSF3	0,94
Transcript Identified by AceView, Entrez Gene ID(s) 84193	SETD3	0,94
chromosome 12 open reading frame 77	C12orf77	0,94
otopetrin 1	OTOP1	0,94
PRAME family member 25	PRAMEF25	0,94
methionine sulfoxide reductase A	MSRA	0,94
actin-like 10	ACTL10	0,94
Transcript Identified by AceView, Entrez Gene ID(s) 51755	CDK12	0,94
heterogeneous nuclear ribonucleoprotein A1-like 2	HNRNPA1L2	0,94
ring finger protein 20, E3 ubiquitin protein ligase	RNF20	0,94
myosin light chain 7	MYL7	0,94
schlafen family member 14	SLFN14	0,94
NOP9 nucleolar protein	NOP9	0,94
ankyrin repeat domain 30B-like	ANKRD30BL	0,94
translocase of outer mitochondrial membrane 20 homolog (yeast)-like	TOMM20L	0,94
speedy/RINGO cell cycle regulator family member E16 [Source:HGNC Symbol;Acc:HGNC:51512]	SPDYE16	0,94
mesenchyme homeobox 2	MEOX2	0,94

defensin, beta 112	DEFB112	0,94
C-type lectin domain family 17, member A	CLEC17A	0,94
metaxin 1	MTX1	0,94
EF-hand calcium binding domain 3	EFCAB3	0,94
estrogen-related receptor beta	ESRRB	0,94
BPI fold containing family A, member 3	BPIFA3	0,94
chromosome 2 open reading frame 48	C2orf48	0,94
Transcript Identified by AceView, Entrez Gene ID(s) 1791	DNTT	0,94
Transcript Identified by AceView, Entrez Gene ID(s) 79922; novel transcript	CTB-75G16.3; MRM1	0,94
zinc finger protein 225	ZNF225	0,94
RAD21 antisense RNA 1	RAD21-AS1	0,94
myosin light chain kinase 2	MYLK2	0,94
phosphoribosyl pyrophosphate synthetase 2	PRPS2	0,94
propionyl-CoA carboxylase alpha subunit	PCCA	0,94
zinc finger protein 883	ZNF883	0,94
immunoglobulin superfamily, member 11	IGSF11	0,94
Transcript Identified by AceView, Entrez Gene ID(s) 3082	HGF	0,94
peroxiredoxin 6	PRDX6	0,94
zinc finger protein 8	ZNF8	0,94
cytochrome P450, family 27, subfamily B, polypeptide 1	CYP27B1	0,94
2-oxoglutarate and iron-dependent oxygenase domain containing 2	OGFOD2	0,94
BARX homeobox 1	BARX1	0,94
transmembrane protein 72	TMEM72	0,94
cyclin-dependent kinase 16	CDK16	0,94
ubiquitin specific peptidase 48	USP48	0,94
interferon, alpha 1	IFNA1	0,94
KIAA2026	KIAA2026	0,94
chromosome 2 open reading frame 54	C2orf54	0,94
defensin, beta 121	DEFB121	0,94
NADH dehydrogenase (ubiquinone) 1 alpha subcomplex, 10, 42kDa	NDUFA10	0,94
nidogen 2 (osteonidogen)	NID2	0,94
citrate synthase	CS	0,94
interferon, lambda 3	IFNL3	0,94
delta-like 2 homolog (Drosophila)	DLK2	0,94
apoptotic peptidase activating factor 1	APAF1	0,94
FERM domain containing 7	FRMD7	0,94
Zhang2013 ALT_ACCEPTOR, ALT_DONOR, coding, INTERNAL, intronic best transcript NM_001006641	SLC25A25	0,94
pleckstrin homology domain containing, family G (with RhoGef domain) member 7	PLEKHG7	0,94

tumor necrosis factor (ligand) superfamily, member 8	TNFSF8	0,94
spectrin repeat containing, nuclear envelope 2	SYNE2	0,94
eukaryotic translation initiation factor 3, subunit C; eukaryotic translation initiation factor 3, subunit C-like	EIF3C; EIF3CL	0,94
long intergenic non-protein coding RNA 266-1	LINC00266-1	0,94
FtsJ homolog 3 (E. coli)	FTSJ3	0,94
EEF1E1-BLOC1S5 readthrough (NMD candidate)	EEF1E1-BLOC1S5	0,94
carboxypeptidase O	CPO	0,94
myosin, heavy chain 4, skeletal muscle	MYH4	0,94
hydroxyprostaglandin dehydrogenase 15-(NAD)	HPGD	0,94
dual serine/threonine and tyrosine protein kinase	DSTYK	0,94
transmembrane protein 89	TMEM89	0,94
TRK-fused gene	TFG	0,94
actinin, alpha 3 (gene/pseudogene)	ACTN3	0,94
Memczak2013 ANTISENSE, CDS, coding, INTERNAL best transcript NM_156039	CSF3R	0,94
chromosome 19 open reading frame 68	C19orf68	0,94
olfactory receptor, family 8, subfamily U, member 1; olfactory receptor, family 8, subfamily U, member 8	OR8U1; OR8U8	0,94
NIPA-like domain containing 4	NIPAL4	0,94
CAP-GLY domain containing linker protein 2	CLIP2	0,94
zinc finger CCCH-type domain-containing-like	LOC441155	0,94
stromal cell-derived factor 2-like 1	SDF2L1	0,94
diphthamine biosynthesis 6	DPH6	0,94
MAGE family member A9B; MAGE family member A9	MAGEA9B; MAGEA9	0,94
SENP3-EIF4A1 readthrough (NMD candidate)	SENP3-EIF4A1	0,94
SH2 domain containing 7	SH2D7	0,94
ATPase, Na+/K+ transporting, alpha 1 polypeptide	ATP1A1	0,94
beta-carotene oxygenase 1	BCO1	0,94
prefoldin subunit 5	PFDN5	0,94
phospholipase C-like 1	PLCL1	0,94
Fc fragment of IgG, low affinity IIIb, receptor (CD16b)	FCGR3B	0,94
GDNF family receptor alpha 4	GFRA4	0,94
heparanase 2 (inactive)	HPSE2	0,94
hypoxia up-regulated 1	HYOU1	0,94
vascular endothelial growth factor C	VEGFC	0,94
wingless-type MMTV integration site family, member 4	WNT4	0,94
cytokine receptor-like factor 3	CRLF3	0,94
BRCA2 and CDKN1A interacting protein	BCCIP	0,94
chemokine (C-C motif) ligand 19	CCL19	0,94
Transcript Identified by AceView, Entrez Gene ID(s) 91445	RNF185	0,94
chromosome 2 open reading frame 49	C2orf49	0,93
dachshund family transcription factor 2	DACH2	0,93

PCOLCE antisense RNA 1	PCOLCE-AS1	0,93
oncomodulin 2	OCM2	0,93
dedicator of cytokinesis 10	DOCK10	0,93
zinc finger, FYVE domain containing 28	ZFYVE28	0,93
inhibitor of Bruton agammaglobulinemia tyrosine kinase	IBTK	0,93
zinc finger protein 574	ZNF574	0,93
FXYD domain containing ion transport regulator 6; FXYD6-FXYD2 readthrough; FXYD domain containing ion transport regulator 2	FXYD6; FXYD6-FXYD2; FXYD2	0,93
tRNA methyltransferase 10C, mitochondrial RNase P subunit	TRMT10C	0,93
olfactory receptor, family 2, subfamily Z, member 1	OR2Z1	0,93
calcium channel, voltage-dependent, N type, alpha 1B subunit	CACNA1B	0,93
Salzman2013 ANTISENSE, CDS, coding, INTERNAL, intronic, OVCODE, OVERLAPTX, OVEXON best transcript NM_001014809	CRMP1	0,93
thrombopoietin	THPO	0,93
Sys1 golgi trafficking protein	SYS1	0,93
phospholipase C, eta 1	PLCH1	0,93
Rho GTPase activating protein 36	ARHGAP36	0,93
microtubule-associated protein 10	MAP10	0,93
Memczak2013 ALT_ACCEPTOR, ALT_DONOR, coding, INTERNAL, intronic best transcript NM_152701	ABCA13	0,93
carcinoembryonic antigen-related cell adhesion molecule 4	CEACAM4	0,93
zinc finger protein 654	ZNF654	0,93
SH2 domain containing 1A	SH2D1A	0,93
chromosome 7 open reading frame 61	C7orf61	0,93
neuropeptide FF receptor 1	NPFFR1	0,93
Transcript Identified by AceView, Entrez Gene ID(s) 56895	AGPAT4	0,93
Jeck2013 ALT_ACCEPTOR, ALT_DONOR, coding, INTERNAL, intronic best transcript NM_001080396	FAM155A	0,93
Transcript Identified by AceView, Entrez Gene ID(s) 1488	CTBP2	0,93
Memczak2013 ALT_ACCEPTOR, ALT_DONOR, coding, INTERNAL, intronic best transcript NM_015032	PDS5B	0,93
olfactory receptor, family 10, subfamily R, member 2	OR10R2	0,93
protein kinase, AMP-activated, alpha 1 catalytic subunit	PRKAA1	0,93
fibroblast growth factor 3	FGF3	0,93
malate dehydrogenase 1B	MDH1B	0,93
RAS p21 protein activator (GTPase activating protein) 1	RASA1	0,93
pseudopodium-enriched atypical kinase 1	PEAK1	0,93

transforming growth factor alpha	TGFA	0,93
zinc finger protein 99	ZNF99	0,93
SERTA domain containing 4	SERTAD4	0,93
adenylate cyclase 8 (brain)	ADCY8	0,93
transcription elongation factor A (SII)-like 6	TCEAL6	0,93
phospholipid phosphatase related 3	PLPPR3	0,93
SET domain containing 1A	SETD1A	0,93
Memczak2013 ALT_ACCEPTOR, ALT_DONOR, coding, INTERNAL, intronic best transcript NM_052847	GNG7	0,93
solute carrier family 28 (concentrative nucleoside transporter), member 1	SLC28A1	0,93
killer cell lectin-like receptor subfamily G, member 1	KLRG1	0,93
N-terminal EF-hand calcium binding protein 2	NECAB2	0,93
uncharacterized LOC339862; novel transcript; Transcript Identified by AceView	LOC339862; RP11-320G10.1; skerdorbo	0,93
intracisternal A particle-promoted polypeptide	IPP	0,93
P antigen family, member 3 (prostate associated)	PAGE3	0,93
transmembrane protein 132E	TMEM132E	0,93
guanine nucleotide binding protein (G protein), gamma 8	GNG8	0,93
coiled-coil domain containing 60	CCDC60	0,93
interferon, lambda 1	IFNL1	0,93
chromosome 17 open reading frame 50	C17orf50	0,93
heparanase	HPSE	0,93
cellular retinoic acid binding protein 1	CRABP1	0,93
URI1, prefoldin-like chaperone	URI1	0,93
Memczak2013 ANTISENSE, CDS, coding, INTERNAL best transcript NM_005120	MED12	0,93
PDZ domain containing ring finger 3	PDZRN3	0,93
plakophilin 1	PKP1	0,93
solute carrier family 9, subfamily A (NHE5, cation proton antiporter 5), member 5	SLC9A5	0,93
Transcript Identified by AceView, Entrez Gene ID(s) 8896	BUD31	0,93
chemokine (C-X-C motif) ligand 12	CXCL12	0,93
plasmalemma vesicle associated protein	PLVAP	0,93
guanine nucleotide binding protein (G protein), beta 5	GNB5	0,93
bone morphogenetic protein/retinoic acid inducible neural-specific 2	BRINP2	0,93
somatostatin receptor 1	SSTR1	0,93
schlafen family member 11	SLFN11	0,93
protein kinase D1	PRKD1	0,93
kinesin light chain 3	KLC3	0,93
testis expressed 11	TEX11	0,93

limb development membrane protein 1	LMBR1	0,93
histocompatibility (minor) HB-1	HMHB1	0,93
DEP domain containing 5	DEPDC5	0,93
membrane-spanning 4-domains, subfamily A, member 6E	MS4A6E	0,93
Memczak2013 ALT_ACCEPTOR, ALT_DONOR, coding, INTERNAL, intronic best transcript NM_021141	XRCC5	0,93
ring finger protein 17	RNF17	0,93
poly(A) binding protein, cytoplasmic 1-like 2A	PABPC1L2A	0,93
eukaryotic translation initiation factor 3, subunit A	EIF3A	0,93
coiled-coil and C2 domain containing 1A	CC2D1A	0,93
nucleoporin 43kDa	NUP43	0,93
NADH dehydrogenase (ubiquinone) Fe-S protein 8, 23kDa (NADH-coenzyme Q reductase); microRNA 4691; microRNA 7113	NDUFS8; MIR4691; MIR7113	0,93
tumor suppressing subtransferable candidate 4	TSSC4	0,93
transmembrane protein 41B	TMEM41B	0,93
kinesin family member 12	KIF12	0,93
presenilin 2	PSEN2	0,93
leucine rich repeat and fibronectin type III domain containing 3	LRFN3	0,93
one cut homeobox 2	ONECUT2	0,93
CXXC finger protein 4	CXXC4	0,93
gastrokine 2	GKN2	0,93
opticin	OPTC	0,93
Memczak2013 ANTISENSE, CDS, coding, INTERNAL best transcript NM_017519	ARID1B	0,93
Homo sapiens chromosome X open reading frame 51A (CXorf51A), mRNA.; Homo sapiens chromosome X open reading frame 51B (CXorf51B), mRNA.; Transcript Identified by AceView, Entrez Gene ID(s) 100129239, RefSeq ID(s) NM_001144064; chromosome X open reading frame 51A [Source:HGNC Symbol;Acc:HGNC:30533]; chromosome X open reading frame 51	CXorf51A; CXorf51B; CXorf51	0,93
RUN and FYVE domain containing 2	RUFY2	0,93
defensin, beta 105B; defensin, beta 105A	DEFB105B; DEFB105A	0,93
B melanoma antigen; B melanoma antigen family, member 4; B melanoma antigen family, member 3; B melanoma antigen family, member 2; B melanoma antigen family, member 5	BAGE; BAGE4; BAGE3; BAGE2; BAGE5	0,93
kelch-like family member 38	KLHL38	0,93
hypoxia up-regulated 1	HYOU1	0,93
olfactory receptor, family 1, subfamily B, member 1 (gene/pseudogene)	OR1B1	0,93

ATP synthase, H+ transporting, mitochondrial F1 complex, beta polypeptide	ATP5B	0,93
	LRIG1	0,93
FCH domain only 2	FCHO2	0,93
adenosine deaminase, RNA-specific, B2 (inactive)	ADARB2	0,93
vomeronasal 1 receptor 5 (gene/pseudogene)	VN1R5	0,93
phospholipase C, gamma 1; ribosomal protein L23a pseudogene 81	PLCG1; RPL23AP81	0,93
glycosyltransferase 6 domain containing 1	GLT6D1	0,93
kinetochore-localized astrin/SPAG5 binding protein	KNSTRN	0,93
immunoglobulin-like domain containing receptor 2	ILDR2	0,93
ubiquitin specific peptidase 51	USP51	0,93
speedy/RINGO cell cycle regulator family member E1	SPDYE1	0,93
5-hydroxytryptamine (serotonin) receptor 1A, G protein-coupled	HTR1A	0,93
sperm mitochondria-associated cysteine-rich protein	SMCP	0,93
olfactory receptor, family 1, subfamily J, member 4	OR1J4	0,93
zinc finger protein 648	ZNF648	0,93
solute carrier family 25, member 46	SLC25A46	0,93
RGD motif, leucine rich repeats, tropomodulin domain and proline-rich containing	RLTPR	0,93
guanylate binding protein 7	GBP7	0,93
CKLF-like MARVEL transmembrane domain containing 1	CMTM1	0,93
zinc finger protein 546	ZNF546	0,93
sushi domain containing 4	SUSD4	0,93
tonsoku-like, DNA repair protein	TONSL	0,93
SET domain containing 6	SETD6	0,93
flavin containing monooxygenase 6 pseudogene	FMO6P	0,93
calcium/calmodulin-dependent protein kinase II inhibitor 2	CAMK2N2	0,93
ribonuclease, RNase A family, 10 (non-active)	RNASE10	0,93
gamma-glutamylamine cyclotransferase	GGACT	0,93
KH domain containing, RNA binding, signal transduction associated 2	KHDRBS2	0,93
lipase, gastric	LIPF	0,93
R-spondin 4	RSPO4	0,93
desmocollin 1	DSC1	0,93
Memczak2013 ANTISENSE, CDS, coding, INTERNAL best transcript NM_198525	KIF7	0,93
microRNA 5195; immunoglobulin heavy variable 5-78 (pseudogene)	MIR5195; IGHV5-78	0,93
RAB24, member RAS oncogene family	RAB24	0,93

Homo sapiens uncharacterized LOC150527 (TISP43), transcript variant 1, non-coding RNA.; Homo sapiens uncharacterized LOC150527 (TISP43), transcript variant 2, non-coding RNA.; Homo sapiens uncharacterized LOC646743 (LOC646743), non-coding RNA.; Transcript Identified by AceView, Entrez Gene ID(s) 150527; Transcript Identified by AceView, Entrez Gene ID(s) 150527, RefSeq ID(s) NR_027313; uncharacterized LOC150527 [Source:EntrezGene;Acc:150527]	TISP43; LOC646743; LOC150527	0,93
cytochrome c oxidase subunit VIa polypeptide 1	COX6A1	0,93
Fas ligand (TNF superfamily, member 6)	FASLG	0,93
olfactory receptor, family 4, subfamily Q, member 2 (gene/pseudogene)	OR4Q2	0,93
ankyrin repeat domain 45	ANKRD45	0,93
LUC7-like	LUC7L	0,93
defensin, beta 135	DEFB135	0,93
solute carrier family 35, member F4	SLC35F4	0,93
transmembrane 6 superfamily member 2	TM6SF2	0,93
RAN guanine nucleotide release factor	RANGRF	0,93
potassium channel, voltage gated Shaw related subfamily C, member 3	KCNC3	0,93
cell cycle associated protein 1	CAPRIN1	0,93
PR domain containing 5	PRDM5	0,93
methyl-CpG binding domain protein 3-like 3	MBD3L3	0,93
poly(ADP-ribose) polymerase family member 15	PARP15	0,93
N-terminal EF-hand calcium binding protein 3	NECAB3	0,93
pyrin domain containing 2	PYDC2	0,93
polymerase (RNA) mitochondrial (DNA directed)	POLRMT	0,93
coiled-coil domain containing 40	CCDC40	0,93
autophagy related 16-like 1	ATG16L1	0,93
ADAM metallopeptidase domain 2	ADAM2	0,93
poly(A) binding protein interacting protein 2B	PAIP2B	0,93
MANSC domain containing 1	MANSC1	0,93
growth factor independent 1B transcription repressor	GFI1B	0,93
NME/NM23 family member 8	NME8	0,93
claudin 34	CLDN34	0,93
WD repeat domain 33	WDR33	0,93
family with sequence similarity 78, member A	FAM78A	0,93
K(lysine) acetyltransferase 6B	KAT6B	0,93
regulator of G-protein signaling 4	RGS4	0,93
odorant binding protein 2B	OBP2B	0,93
zinc finger and BTB domain containing 25	ZBTB25	0,93
coiled-coil domain containing 86	CCDC86	0,93
spermatogenesis associated 45	SPATA45	0,93

potassium channel, voltage gated eag related subfamily H, member 6	KCNH6	0,93
chromosome 10 open reading frame 62	C10orf62	0,93
cannabinoid receptor 1 (brain)	CNR1	0,93
GRIP and coiled-coil domain containing 2	GCC2	0,93
cementum protein 1	CEMP1	0,93
olfactory marker protein	OMP	0,93
DEAD (Asp-Glu-Ala-Asp) box polypeptide 55	DDX55	0,93
Sp3 transcription factor	SP3	0,93
homeobox C8	HOXC8	0,93
Memczak2013 ANTISENSE, CDS, coding, INTERNAL best transcript NM_006901	MYO9A	0,93
zinc finger protein 41	ZNF41	0,93
long intergenic non-protein coding RNA 602	LINC00602	0,93
drosha, ribonuclease type III	DROSHA	0,93
neuritin 1-like	NRN1L	0,93
tyrosinase-related protein 1	TYRP1	0,93
CD226 molecule	CD226	0,93
Transcript Identified by AceView, Entrez Gene ID(s) 8242	KDM5C	0,93
ATP synthase, H+ transporting, mitochondrial Fo complex subunit B1	ATP5F1	0,93
ariadne RBR E3 ubiquitin protein ligase 2	ARIH2	0,93
suppressor of cytokine signaling 6	SOCS6	0,93
ubiquitin interaction motif containing 1	UIMC1	0,93
ring finger protein 224	RNF224	0,93
heat shock protein, alpha-crystallin-related, B6	HSPB6	0,93
angiopoietin 1	ANGPT1	0,93
family with sequence similarity 91, member A1	FAM91A1	0,93
klotho	KL	0,93
phosphohistidine phosphatase 1	PHPT1	0,93
intraflagellar transport 80	IFT80	0,93
solute carrier family 5 (sodium/monocarboxylate cotransporter), member 8	SLC5A8	0,93
protein phosphatase 1, regulatory subunit 36	PPP1R36	0,93
ubiquitin specific peptidase 40	USP40	0,93
solute carrier family 36, member 3	SLC36A3	0,93
family with sequence similarity 83, member C	FAM83C	0,93
CKLF-like MARVEL transmembrane domain containing 2	CMTM2	0,93
calbindin 1	CALB1	0,93
glucosidase, alpha; neutral C	GANC	0,93
solute carrier family 4 (anion exchanger), member 2	SLC4A2	0,93
solute carrier family 7 (amino acid transporter light chain, L system), member 5	SLC7A5	0,93
S-antigen; retina and pineal gland (arrestin)	SAG	0,93

thioredoxin domain containing 8 (spermatozoa)	TXNDC8	0,93
H1 histone family, member O, oocyte-specific	H1FOO	0,93
CD37 molecule	CD37	0,93
Memczak2013 ANTISENSE, coding, INTERNAL, intronic best transcript NM_002072; Transcript Identified by AceView	GNAQ; skeyplorbu	0,93
neuronal PAS domain protein 3	NPAS3	0,93
calcium channel, voltage-dependent, gamma subunit 3	CACNG3	0,93
clathrin heavy chain linker domain containing 1	CLHC1	0,93
BTB (POZ) domain containing 8	BTBD8	0,93
guanine nucleotide binding protein (G protein), gamma 5	GNG5	0,93
chorionic gonadotropin, beta polypeptide; chorionic gonadotropin, beta polypeptide 5	CGB; CGB5	0,93
G protein-coupled receptor, class C, group 5, member D	GPRC5D	0,93
La ribonucleoprotein domain family, member 7	LARP7	0,93
gastrin	GAST	0,93
SRSF protein kinase 3	SRPK3	0,93
SOS Ras/Rac guanine nucleotide exchange factor 1	SOS1	0,93
zinc finger protein 112	ZNF112	0,93
V-set domain containing T cell activation inhibitor 1	VTCN1	0,93
zinc finger protein 814	ZNF814	0,93
CD3e molecule, epsilon (CD3-TCR complex)	CD3E	0,93
alkB homolog 4, lysine demthylase	ALKBH4	0,93
T-cell, immune regulator 1, ATPase, H+ transporting, lysosomal V0 subunit A3	TCIRG1	0,93
myosin, heavy chain 6, cardiac muscle, alpha	MYH6	0,93
Mov10 RISC complex RNA helicase like 1	MOV10L1	0,93
hedgehog acyltransferase	HHAT	0,93
fatty acid desaturase 6	FADS6	0,93
Salzman2013 ANTISENSE, coding, INTERNAL, intronic best transcript NM_001034845	GALNTL6	0,93
ubiquitin specific peptidase 29	USP29	0,93
family with sequence similarity 227, member A	FAM227A	0,93
uncharacterized LOC100133091; Salzman2013 ANNOTATED, INTERNAL, ncRNA, OVEXON best transcript NR_029411; Transcript Identified by AceView, Entrez Gene ID(s) 554248; 100133091, RefSeq ID(s) NR_029411	LOC100133091	0,93
l(3)mbt-like 3 (Drosophila)	L3MBTL3	0,93
ribosomal RNA processing 36	RRP36	0,93
CD1c molecule	CD1C	0,93
Transcript Identified by AceView, Entrez Gene ID(s) 23518	R3HDM1	0,93
alanyl-tRNA synthetase 2, mitochondrial	AARS2	0,93

solute carrier family 35, member F4	SLC35F4	0,93
oxysterol binding protein	OSBP	0,93
coiled-coil domain containing 107	CCDC107	0,93
olfactory receptor, family 6, subfamily B, member 3	OR6B3	0,93
peptidylprolyl isomerase (cyclophilin)-like 1	PPIL1	0,93
transmembrane protease, serine 5	TMPRSS5	0,93
colorectal neoplasia differentially expressed (non-protein coding)	CRNDE	0,93
Jeck2013 ALT_ACCEPTOR, ALT_DONOR, coding, INTERNAL, intronic best transcript NM_178423	HDAC9	0,93
SCY1-like, kinase-like 3	SCYL3	0,93
protein tyrosine phosphatase, non-receptor type 6	PTPN6	0,93
X antigen family, member 3	XAGE3	0,93
acyl-CoA wax alcohol acyltransferase 2	AWAT2	0,93
Zhang2013 ALT_ACCEPTOR, ALT_DONOR, coding, INTERNAL, intronic best transcript NM_032447; Transcript Identified by AceView	FBN3; mumari	0,93
pyrroline-5-carboxylate reductase family, member 2; microRNA 6741	PYCR2; MIR6741	0,93
granzyme H	GZMH	0,93
oocyte secreted protein 2	OOSP2	0,93
DLC1 Rho GTPase activating protein	DLC1	0,93
nuclear receptor subfamily 1, group H, member 3	NR1H3	0,93
coiled-coil domain containing 191	CCDC191	0,93
centrosomal protein 78kDa	CEP78	0,93
SPT20 homolog, SAGA complex component-like 1	SUPT20HL1	0,93
Memczak2013 ALT_ACCEPTOR, ALT_DONOR, coding, INTERNAL, intronic best transcript NM_001193522	FAM65A	0,93
potassium channel tetramerization domain containing 19	KCTD19	0,93
histone cluster 1, H3i	HIST1H3I	0,93
chromosome 8 open reading frame 87	C8orf87	0,93
homeobox D12	HOXD12	0,93
solute carrier family 46, member 3	SLC46A3	0,93
CGG triplet repeat binding protein 1	CGGBP1	0,93
centrosomal protein 19kDa	CEP19	0,93
CUB and Sushi multiple domains 2	CSMD2	0,93
MT-RNR2-like 8; microRNA 4485	MTRNR2L8; MIR4485	0,93
adipogenesis regulatory factor; ankyrin repeat and GTPase domain Arf GTPase activating protein 11; BMS1 ribosome biogenesis factor pseudogene 3	ADIRF; AGAP11; BMS1P3	0,93
growth hormone releasing hormone receptor	GHRHR	0,93
leukocyte immunoglobulin-like receptor, subfamily B (with TM and ITIM domains), member 4	LILRB4	0,93

transmembrane protein 238	TMEM238	0,93
kelch-like family member 24	KLHL24	0,93
UDP-GlcNAc:betaGal beta-1,3-N-acetylglucosaminyltransferase 5	B3GNT5	0,93
chromosome 11 open reading frame 97	C11orf97	0,93
kinetochore associated 1	KNTC1	0,93
ubiquitin protein ligase E3A	UBE3A	0,93
HIG1 hypoxia inducible domain family, member 1A	HIGD1A	0,93
profilin family, member 4	PFN4	0,93
zinc finger protein 692	ZNF692	0,93
toll-like receptor 8	TLR8	0,93
ligand of numb-protein X 2	LNX2	0,93
oligodendrocyte lineage transcription factor 2	OLIG2	0,92
chloride intracellular channel 6	CLIC6	0,92
pancreatic lipase-related protein 3	PNLIPRP3	0,92
valosin containing protein	VCP	0,92
cytidine monophosphate (UMP-CMP) kinase 1, cytosolic	CMPK1	0,92
olfactory receptor, family 51, subfamily A, member 7	OR51A7	0,92
KRAB-A domain containing 1	KRBA1	0,92
adhesion G protein-coupled receptor E3	ADGRE3	0,92
RAB11 family interacting protein 1 (class I)	RAB11FIP1	0,92
sodium leak channel, non selective	NALCN	0,92
solute carrier family 28 (concentrative nucleoside transporter), member 3	SLC28A3	0,92
homer scaffolding protein 1	HOMER1	0,92
amelogenin, X-linked	AMELX	0,92
solute carrier family 2 (facilitated glucose transporter), member 11	SLC2A11	0,92
RNA binding motif protein, X-linked-like 2	RBMXL2	0,92
filamin B, beta	FLNB	0,92
O-linked N-acetylglucosamine (GlcNAc) transferase	OGT	0,92
E2F transcription factor 2	E2F2	0,92
DNAJC25-GNG10 readthrough	DNAJC25-GNG10	0,92
ankyrin repeat and kinase domain containing 1	ANKK1	0,92
myogenic factor 6 (herculin)	MYF6	0,92
proline rich 15	PRR15	0,92
tumor necrosis factor receptor superfamily, member 10b	TNFRSF10B	0,92
taste receptor, type 2, member 20	TAS2R20	0,92
death-domain associated protein	DAXX	0,92
solute carrier family 17, member 4	SLC17A4	0,92
NDC1 transmembrane nucleoporin	NDC1	0,92
fibroblast growth factor binding protein 2	FGFBP2	0,92
olfactory receptor, family 2, subfamily F, member 2	OR2F2	0,92
taxilin gamma	TXLNG	0,92

SERTA domain containing 2	SERTAD2	0,92
E74-like factor 1 (ets domain transcription factor)	ELF1	0,92
prostate androgen-regulated mucin-like protein 1	PARM1	0,92
sosondowah ankyrin repeat domain family member D	SOWAHD	0,92
chromosome 12 open reading frame 73	C12orf73	0,92
zinc finger, FYVE domain containing 19	ZFYVE19	0,92
lipolysis stimulated lipoprotein receptor	LSR	0,92
family with sequence similarity 107, member A	FAM107A	0,92
signal recognition particle 72kDa	SRP72	0,92
ring finger protein 182	RNF182	0,92
Memczak2013 ANTISENSE, CDS, coding, INTERNAL best transcript NM_000250	MPO	0,92
Transcript Identified by AceView, Entrez Gene ID(s) 55315	SLC29A3	0,92
dynein heavy chain -like pseudogene; TEC	LOC730668; CITF22-92A6.2	0,92
family with sequence similarity 129, member A	FAM129A	0,92
WD repeat domain 83	WDR83	0,92
olfactory receptor, family 5, subfamily H, member 14	OR5H14	0,92
activating signal cointegrator 1 complex subunit 1	ASCC1	0,92
zinc finger protein 316	ZNF316	0,92
leucyl-tRNA synthetase	LARS	0,92
WAP four-disulfide core domain 9	WFDC9	0,92
defensin, beta 109, pseudogene 1B	DEFB109P1B	0,92
wingless-type MMTV integration site family, member 3	WNT3	0,92
poly(A) binding protein, cytoplasmic 1-like 2B	PABPC1L2B	0,92
tropomodulin 2 (neuronal)	TMOD2	0,92
potassium channel, two pore domain subfamily K, member 6	KCNK6	0,92
interferon regulatory factor 6	IRF6	0,92
neuroblastoma breakpoint family, member 14	NBPF14	0,92
chorionic gonadotropin, beta polypeptide 8	CGB8	0,92
3-hydroxyacyl-CoA dehydratase 4	HACD4	0,92
testis-specific serine kinase 6	TSSK6	0,92
neuroblastoma breakpoint family, member 10	NBPF10	0,92
Transcript Identified by AceView, Entrez Gene ID(s) 153562	MARVELD2	0,92
Salzman2013 ALT_ACCEPTOR, ALT_DONOR, coding, INTERNAL, intronic best transcript NM_152513	MEI1	0,92
sodium channel, voltage gated, type II beta subunit	SCN2B	0,92
tumor protein, translationally-controlled 1; small nucleolar RNA, H/ACA box 31	TPT1; SNORA31	0,92
chromosome 1 open reading frame 167	C1orf167	0,92
heparan sulfate 6-O-sulfotransferase 2	HS6ST2	0,92

Transcript Identified by AceView, Entrez Gene ID(s) 92691	TMEM169	0,92
gonadotropin releasing hormone 1	GNRH1	0,92
cancer/testis antigen 1B; cancer/testis antigen 1A	CTAG1B; CTAG1A	0,92
zinc finger RNA binding protein	ZFR	0,92
v-ral simian leukemia viral oncogene homolog A (ras related)	RALA	0,92
cytochrome c oxidase subunit VIIb2	COX7B2	0,92
neurogenin 2	NEUROG2	0,92
nuclear receptor corepressor 1	NCOR1	0,92
von Hippel-Lindau tumor suppressor-like	VHLL	0,92
gamma-aminobutyric acid (GABA) A receptor, theta	GABRQ	0,92
potassium voltage-gated channel, modifier subfamily S, member 1	KCNS1	0,92
olfactory receptor, family 51, subfamily A, member 2	OR51A2	0,92
RUN domain containing 3B	RUNDC3B	0,92
Transcript Identified by AceView, Entrez Gene ID(s) 23126	POGZ	0,92
carbohydrate (N-acetylglucosamine 6-O) sulfotransferase 4	CHST4	0,92
spermidine/spermine N1-acetyltransferase family member 2	SAT2	0,92
immunoglobulin superfamily, member 10	IGSF10	0,92
surfactant protein C	SFTPC	0,92
V-set and transmembrane domain containing 2B	VSTM2B	0,92
glutaminase 2 (liver, mitochondrial)	GLS2	0,92
NHS-like 2	NHSL2	0,92
indolethylamine N-methyltransferase; family with sequence similarity 188, member B; INMT-FAM188B readthrough (NMD candidate)	INMT; FAM188B; INMT-FAM188B	0,92
lipase, endothelial	LIPG	0,92
methyl-CpG binding domain protein 3-like 5	MBD3L5	0,92
olfactory receptor, family 10, subfamily A, member 4	OR10A4	0,92
PRAME family member 2	PRAMEF2	0,92
potassium channel, sodium activated subfamily T, member 2	KCNT2	0,92
thymocyte expressed, positive selection associated 1	TESPA1	0,92
gap junction protein alpha 5	GJA5	0,92
adaptor-related protein complex 3, delta 1 subunit	AP3D1	0,92
nuclear factor of activated T-cells, cytoplasmic, calcineurin-dependent 1	NFATC1	0,92
proteasome 26S subunit, ATPase 5	PSMC5	0,92
G protein-coupled receptor 12	GPR12	0,92
chromosome 3 open reading frame 18	C3orf18	0,92

solute carrier family 20 (phosphate transporter), member 2	SLC20A2	0,92
RAD50 homolog, double strand break repair protein	RAD50	0,92
olfactory receptor, family 5, subfamily D, member 14	OR5D14	0,92
cytochrome P450, family 2, subfamily C, polypeptide 19	CYP2C19	0,92
zinc finger, CCHC domain containing 10	ZCCHC10	0,92
potassium channel, inwardly rectifying subfamily J, member 15	KCNJ15	0,92
olfactory receptor, family 1, subfamily L, member 3	OR1L3	0,92
microsomal glutathione S-transferase 3	MGST3	0,92
translocase of inner mitochondrial membrane 8 homolog A (yeast)	TIMM8A	0,92
toll-like receptor 5	TLR5	0,92
tripartite motif containing 40	TRIM40	0,92
ADAM metallopeptidase domain 29	ADAM29	0,92
claudin 14	CLDN14	0,92
male-specific lethal 2 homolog (Drosophila)	MSL2	0,92
butyrophilin, subfamily 1, member A1	BTN1A1	0,92
zinc finger protein 687	ZNF687	0,92
erb-b2 receptor tyrosine kinase 2	ERBB2	0,92
wingless-type MMTV integration site family, member 8B	WNT8B	0,92
ubiquitin-like with PHD and ring finger domains 2, E3 ubiquitin protein ligase	UHRF2	0,92
G protein-coupled receptor 150	GPR150	0,92
Fas apoptotic inhibitory molecule 2	FAIM2	0,92
protein phosphatase 1, catalytic subunit, beta isozyme	PPP1CB	0,92
prefoldin subunit 2	PFDN2	0,92
transient receptor potential cation channel, subfamily A, member 1	TRPA1	0,92
chondrolectin	CHODL	0,92
interleukin 17A	IL17A	0,92
sorting nexin 25	SNX25	0,92
acylglycerol kinase	AGK	0,92
interleukin 31	IL31	0,92
TBC1 domain family, member 3E	TBC1D3E	0,92
scavenger receptor class F, member 1	SCARF1	0,92
piggyBac transposable element derived 2	PGBD2	0,92
scratch family zinc finger 1	SCRT1	0,92
transient receptor potential cation channel, subfamily C, member 5	TRPC5	0,92
UPF3 regulator of nonsense transcripts homolog A (yeast)	UPF3A	0,92
prolactin releasing hormone	PRLH	0,92
nucleotide binding protein 2	NUBP2	0,92

EPH receptor A3	EPHA3	0,92
ribosomal protein L24	RPL24	0,92
fibroblast growth factor 10	FGF10	0,92
RAD51 paralog D	RAD51D	0,92
RNA binding motif protein 12B	RBM12B	0,92
aldo-keto reductase family 1, member E2	AKR1E2	0,92
protein kinase, AMP-activated, gamma 3 non-catalytic subunit	PRKAG3	0,92
family with sequence similarity 209, member A	FAM209A	0,92
solute carrier family 12, member 9	SLC12A9	0,92
myosin XVI	MYO16	0,92
olfactory receptor, family 1, subfamily L, member 6	OR1L6	0,92
olfactory receptor, family 6, subfamily F, member 1; olfactory receptor, family 14, subfamily A, member 2	OR6F1; OR14A2	0,92
nuclear transcription factor Y subunit beta	NFYB	0,92
coenzyme Q9	COQ9	0,92
MMS19 homolog, cytosolic iron-sulfur assembly component	MMS19	0,92
family with sequence similarity 126, member B	FAM126B	0,92
synaptonemal complex central element protein 3	SYCE3	0,92
dynein, light chain, LC8-type 1	DYNLL1	0,92
forkhead box B2	FOXB2	0,92
CD1a molecule	CD1A	0,92
G protein-coupled receptor 6	GPR6	0,92
Rho guanine nucleotide exchange factor 4	ARHGEF4	0,92
FSHD region gene 1	FRG1	0,92
transmembrane protein 221	TMEM221	0,92
CD47 molecule	CD47	0,92
SH3-domain GRB2-like 2	SH3GL2	0,92
hypoxia up-regulated 1	HYOU1	0,92
microtubule-associated protein 1 light chain 3 beta 2	MAP1LC3B2	0,92
S-antigen; retina and pineal gland (arrestin)	SAG	0,92
lon peptidase 2, peroxisomal	LONP2	0,92
sema domain, transmembrane domain (TM), and cytoplasmic domain, (semaphorin) 6D	SEMA6D	0,92
Homo sapiens MGC57346-CRHR1 readthrough (MGC57346-CRHR1), transcript variant 5, mRNA.; Homo sapiens MGC57346-CRHR1 readthrough (MGC57346-CRHR1), transcript variant 6, mRNA.; novel transcript	MGC57346-CRHR1; RP11-105N13.4	0,92
family with sequence similarity 64, member A	FAM64A	0,92
paired box 9	PAX9	0,92
phosphodiesterase 10A	PDE10A	0,92
cilia and flagella associated protein 157	CFAP157	0,92
ubiquitin conjugating enzyme E2G 2	UBE2G2	0,92
zinc finger protein 343	ZNF343	0,92

acyl-CoA thioesterase 12	ACOT12	0,92
chymotrypsinogen B1	CTRB1	0,92
olfactory receptor, family 4, subfamily F, member 21	OR4F21	0,92
ribosomal protein S6 kinase, 90kDa, polypeptide 1	RPS6KA1	0,92
receptor accessory protein 4	REEP4	0,92
novel transcript; Transcript Identified by AceView, Entrez Gene ID(s) 79634	AC010894.3; SCRN3	0,92
chromosome 1 open reading frame 145	C1orf145	0,92
calcyon neuron-specific vesicular protein	CALY	0,92
lethal giant larvae homolog 1 (Drosophila)	LLGL1	0,92
TBC1 domain family, member 25	TBC1D25	0,92
PRAME family member 13	PRAMEF13	0,92
REC114 meiotic recombination protein	REC114	0,92
Jeck2013 ANTISENSE, coding, INTERNAL, intronic best transcript NM_173688	NKAIN3	0,92
leucine-rich repeats and death domain containing 1	LRRD1	0,92
olfactory receptor, family 2, subfamily T, member 5	OR2T5	0,92
solute carrier family 6 (neurotransmitter transporter), member 1	SLC6A1	0,92
contactin associated protein-like 2	CNTNAP2	0,92
endothelial cell-specific molecule 1	ESM1	0,92
carcinoembryonic antigen-related cell adhesion molecule 18	CEACAM18	0,92
LINGO1 antisense RNA 1	LINGO1-AS1	0,92
proline rich 25	PRR25	0,92
TBC1 domain family, member 29	TBC1D29	0,92
dual specificity phosphatase 15	DUSP15	0,92
potassium channel tetramerization domain containing 12	KCTD12	0,92
collapsin response mediator protein 1	CRMP1	0,92
family with sequence similarity 71, member B	FAM71B	0,92
chromatin assembly factor 1, subunit B (p60)	CHAF1B	0,92
rogdi homolog	ROGDI	0,92
cold shock domain containing E1, RNA binding	CSDE1	0,92
Transcript Identified by AceView, Entrez Gene ID(s) 108	ADCY2	0,92
olfactory receptor, family 2, subfamily S, member 2 (gene/pseudogene)	OR2S2	0,92
interleukin 17D	IL17D	0,92
cytochrome P450, family 2, subfamily C, polypeptide 8	CYP2C8	0,92
SERTA domain containing 4	SERTAD4	0,92
G protein-coupled receptor 146	GPR146	0,92
keratin associated protein 19-1	KRTAP19-1	0,92
trans-2,3-enoyl-CoA reductase-like	TECRL	0,92
septin 11	sept-11	0,92
spectrin, beta, non-erythrocytic 5	SPTBN5	0,92

eukaryotic translation elongation factor 1 epsilon 1	EEF1E1	0,92
SEC14-like lipid binding 3	SEC14L3	0,92
serine/arginine-rich splicing factor 11	SRSF11	0,92
keratin associated protein 26-1	KRTAP26-1	0,92
anoctamin 5	ANO5	0,92
testis expressed 261	TEX261	0,92
actin-related protein T1	ACTRT1	0,92
ciliary rootlet coiled-coil, rootletin	CROCC	0,92
leucine-rich repeat, immunoglobulin-like and transmembrane domains 2	LRIT2	0,92
fibroblast growth factor 16	FGF16	0,92
cysteine-rich secretory protein 2	CRISP2	0,92
protocadherin beta 15	PCDHB15	0,92
transducin (beta)-like 1 X-linked receptor 1	TBL1XR1	0,92
chromosome 7 open reading frame 26	C7orf26	0,92
free fatty acid receptor 2	FFAR2	0,92
tigger transposable element derived 7	TIGD7	0,92
UBX domain protein 6	UBXN6	0,92
coiled-coil domain containing 127	CCDC127	0,92
neuron-derived neurotrophic factor	NDNF	0,92
aldo-keto reductase family 1, member A1 (aldehyde reductase)	AKR1A1	0,92
guanylate cyclase 2D, membrane (retina-specific)	GUCY2D	0,92
tripartite motif containing 56	TRIM56	0,92
TIMP metallopeptidase inhibitor 4	TIMP4	0,92
chromosome 9 open reading frame 92	C9orf92	0,92
tubulin, gamma 1	TUBG1	0,92
profilin 3	PFN3	0,92
notch 4	NOTCH4	0,92
secretoglobin, family 2A, member 1	SCGB2A1	0,92
actin, beta-like 2	ACTBL2	0,92
transcription elongation factor B polypeptide 3C-like; transcription elongation factor B polypeptide 3C (elongin A3)	TCEB3CL; TCEB3C	0,92
cerberus 1, DAN family BMP antagonist	CER1	0,92
Memczak2013 ALT_ACCEPTOR, ALT_DONOR, coding, INTERNAL, intronic best transcript NM_001130110	SETBP1	0,92
ABHD14A-ACY1 readthrough	ABHD14A-ACY1	0,92
protein tyrosine kinase 6	PTK6	0,92
RAS p21 protein activator 3	RASA3	0,92
small integral membrane protein 8	SMIM8	0,92
retinal outer segment membrane protein 1	ROM1	0,92
major histocompatibility complex, class I, A	HLA-A	0,92
paraoxonase 1	PON1	0,92
protocadherin 11 Y-linked	PCDH11Y	0,92

secretin	SCT	0,92
zinc finger protein 441	ZNF441	0,92
ribosomal protein, large, P2; small nucleolar RNA, H/ACA box 52	RPLP2; SNORA52	0,92
homeobox C9	HOXC9	0,92
family with sequence similarity 149, member A	FAM149A	0,92
bone morphogenetic protein 8b	BMP8B	0,92
pregnancy up-regulated nonubiquitous CaM kinase	PNCK	0,92
UTP23, small subunit (SSU) processome component, homolog (yeast)	UTP23	0,92
MIR205 host gene; microRNA 205	MIR205HG; MIR205	0,92
von Willebrand factor A domain containing 5B2	VWA5B2	0,92
acid sensing ion channel family member 4	ASIC4	0,92
leucine rich repeat transmembrane neuronal 2	LRRTM2	0,92
phosphate regulating endopeptidase homolog, X-linked	PHEX	0,92
NAD synthetase 1	NADSYN1	0,92
iron-sulfur cluster assembly 1	ISCA1	0,92
ribosomal protein L41 pseudogene 5	RPL41P5	0,92
carbonic anhydrase X	CA10	0,92
cadherin, EGF LAG seven-pass G-type receptor 1	CELSR1	0,92
PHD finger protein 21B	PHF21B	0,92
deoxyribonuclease I-like 2	DNASE1L2	0,92
EP300 interacting inhibitor of differentiation 2	EID2	0,92
MRE11 homolog A, double strand break repair nuclease	MRE11A	0,92
adhesion G protein-coupled receptor G2	ADGRG2	0,92
zona pellucida binding protein	ZPBP	0,92
transient receptor potential cation channel, subfamily M, member 8	TRPM8	0,92
density-regulated protein	DENR	0,92
cAMP-regulated phosphoprotein 21kDa	ARPP21	0,92
coiled-coil domain containing 140	CCDC140	0,92
C3 and PZP-like, alpha-2-macroglobulin domain containing 8	CPAMD8	0,92
Transcript Identified by AceView, Entrez Gene ID(s) 4952	OCRL	0,92
interferon, lambda 4 (gene/pseudogene)	IFNL4	0,92
t-complex-associated-testis-expressed 3	TCTE3	0,92
SIX homeobox 2	SIX2	0,92
CD247 molecule	CD247	0,92
serine incorporator 4	SERINC4	0,92
autophagy related 101	ATG101	0,92
cholecystokinin A receptor	CCKAR	0,92
Memczak2013 ALT_ACCEPTOR, ALT_DONOR, coding, INTERNAL, intronic best transcript NM_170721	MSI2	0,92

protein phosphatase 3, regulatory subunit B, alpha	PPP3R1	0,92
coiled-coil domain containing 174	CCDC174	0,92
zinc finger, GATA-like protein 1	ZGLP1	0,91
glutamine and serine rich 1	QSER1	0,91
olfactory receptor, family 6, subfamily C, member 75	OR6C75	0,91
peroxisomal biogenesis factor 11 gamma	PEX11G	0,91
MGAT4 family, member D	MGAT4D	0,91
protein tyrosine phosphatase, non-receptor type 13 (APO-1/CD95 (Fas)-associated phosphatase)	PTPN13	0,91
THO complex 5	THOC5	0,91
SCP2 sterol-binding domain containing 1	SCP2D1	0,91
leucine rich repeat containing 37A	LRRC37A	0,91
nucleolin	NCL	0,91
uncharacterized LOC100128554; Transcript Identified by AceView, Entrez Gene ID(s) 144678; 100128554; novel transcript	LOC100128554; RP5-944M2.3	0,91
diacylglycerol kinase, delta 130kDa	DGKD	0,91
poly(A) binding protein, nuclear 1-like (cytoplasmic)	PABPN1L	0,91
Transcript Identified by AceView, Entrez Gene ID(s) 57553	MICAL3	0,91
sphingosine-1-phosphate receptor 4	S1PR4	0,91
homeobox A11	HOXA11	0,91
MIR22 host gene	MIR22HG	0,91
GLI pathogenesis-related 1 like 1	GLIPR1L1	0,91
ZFP91 zinc finger protein	ZFP91	0,91
interleukin 36, beta	IL36B	0,91
eukaryotic translation initiation factor 3, subunit L	EIF3L	0,91
Memczak2013 ANTISENSE, CDS, coding, INTERNAL, intronic best transcript NM_004579	MAP4K2	0,91
Transcript Identified by AceView, Entrez Gene ID(s) 161436	EML5	0,91
olfactory receptor, family 11, subfamily L, member 1	OR11L1	0,91
DnaJ (Hsp40) homolog, subfamily C, member 16	DNAJC16	0,91
Transcript Identified by AceView, Entrez Gene ID(s) 7837	PXDN	0,91
prostate and testis expressed 1	PATE1	0,91
tudor domain containing 9	TDRD9	0,91
SH3 and multiple ankyrin repeat domains 2	SHANK2	0,91
Rieske (Fe-S) domain containing	RFESD	0,91
G protein-coupled receptor 152	GPR152	0,91
olfactory receptor, family 13, subfamily C, member 2	OR13C2	0,91
sema domain, immunoglobulin domain (Ig), short basic domain, secreted, (semaphorin) 3A	SEMA3A	0,91
FERM and PDZ domain containing 2B, pseudogene; FERM and PDZ domain containing 2	FRMPD2B; FRMPD2	0,91

oligophrenin 1	OPHN1	0,91
zinc finger protein 775	ZNF775	0,91
adenomatous polyposis coli	APC	0,91
olfactory receptor, family 51, subfamily T, member 1	OR51T1	0,91
DEAD (Asp-Glu-Ala-Asp) box polypeptide 19B	DDX19B	0,91
ribosomal protein S6 kinase, 90kDa, polypeptide 6	RPS6KA6	0,91
ER membrane protein complex subunit 1	EMC1	0,91
Jeck2013 ANTISENSE, coding, INTERNAL, OVEXON, UTR3 best transcript NM_001039690	CHTF8	0,91
microRNA 650; immunoglobulin lambda variable 3-9 (gene/pseudogene); immunoglobulin lambda variable 2-8	MIR650; IGLV3-9; IGLV2-8	0,91
transmembrane protein 232	TMEM232	0,91
CSNK1G2 antisense RNA 1	CSNK1G2-AS1	0,91
FCH and double SH3 domains 2	FCHSD2	0,91
butyrophilin, subfamily 2, member A2	BTN2A2	0,91
transcription elongation factor A (SII)-like 4	TCEAL4	0,91
ubiquitin conjugating enzyme E2E 1	UBE2E1	0,91
GIMAP family P-loop NTPase domain containing 1	GIMD1	0,91
tachykinin receptor 2	TACR2	0,91
taste receptor, type 2, member 7	TAS2R7	0,91
cytochrome P450, family 2, subfamily B, polypeptide 6	CYP2B6	0,91
NADH dehydrogenase (ubiquinone) 1 alpha subcomplex, 7, 14.5kDa	NDUFA7	0,91
KH homology domain containing 1-like	KHDC1L	0,91
NADH dehydrogenase (ubiquinone) 1 beta subcomplex, 5, 16kDa	NDUFB5	0,91
Transcript Identified by AceView, Entrez Gene ID(s) 23250	ATP11A	0,91
DENN/MADD domain containing 1A	DENND1A	0,91
zyg-11 related, cell cycle regulator	ZER1	0,91
zinc finger protein 488	ZNF488	0,91
bradykinin receptor B2	BDKRB2	0,91
MORC family CW-type zinc finger 2	MORC2	0,91
peroxisomal membrane protein 4	PXMP4	0,91
chromosome 20 open reading frame 173	C20orf173	0,91
FERM domain containing 4B	FRMD4B	0,91
histatin 3	HTN3	0,91
family with sequence similarity 217, member A	FAM217A	0,91
synemin, intermediate filament protein	SYNM	0,91
DDB1 and CUL4 associated factor 6	DCAF6	0,91
plasminogen receptor, C-terminal lysine transmembrane protein	PLGRKT	0,91
Morf4 family associated protein 1	MRFAP1	0,91

TP53 target 3B; TP53 target 3; TP53 target 3C	TP53TG3B; TP53TG3; TP53TG3C	0,91
Memczak2013 ALT_ACCEPTOR, ALT_DONOR, coding, INTERNAL, intronic best transcript NM_001040033	CD53	0,91
T cell receptor associated transmembrane adaptor 1	TRAT1	0,91
sodium channel, voltage gated, type X alpha subunit	SCN10A	0,91
SIX homeobox 3	SIX3	0,91
chromosome 14 open reading frame 37	C14orf37	0,91
negative regulator of reactive oxygen species	NRROS	0,91
olfactory receptor, family 52, subfamily R, member 1 (gene/pseudogene)	OR52R1	0,91
A kinase (PRKA) anchor protein 11	AKAP11	0,91
Transcript Identified by AceView, Entrez Gene ID(s) 205564	SENP5	0,91
uncharacterized LOC115110; novel transcript	LOC115110; RP3-395M20.8	0,91
solute carrier family 17 (vesicular glutamate transporter), member 8	SLC17A8	0,91
olfactory receptor, family 2, subfamily T, member 4	OR2T4	0,91
TAP binding protein-like	TAPBPL	0,91
transglutaminase 1	TGM1	0,91
vitamin K epoxide reductase complex subunit 1 like 1	VKORC1L1	0,91
Williams-Beuren syndrome chromosome region 28	WBSCR28	0,91
Transcript Identified by AceView, Entrez Gene ID(s) 65977	PLEKHA3	0,91
glutathione S-transferase pi 1	GSTP1	0,91
UBX domain protein 2A	UBXN2A	0,91
proline rich, lacrimal 1	PROL1	0,91
dynein, axonemal, heavy chain 17	DNAH17	0,91
PDZ and LIM domain 3	PDLIM3	0,91
WW domain binding protein 4	WBP4	0,91
homeobox D11	HOXD11	0,91
germ cell associated 1	GSG1	0,91
desmoglein 1	DSG1	0,91
Transcript Identified by AceView, Entrez Gene ID(s) 10659	CELF2	0,91
peptidylprolyl cis/trans isomerase, NIMA-interacting 1	PIN1	0,91
GATA binding protein 5	GATA5	0,91
glypican 4	GPC4	0,91
Jeck2013 ALT_ACCEPTOR, ALT_DONOR, coding, INTERNAL, intronic best transcript NM_001080396	FAM155A	0,91
olfactory receptor, family 1, subfamily G, member 1	OR1G1	0,91
long intergenic non-protein coding RNA 935	LINC00935	0,91
coiled-coil domain containing 96	CCDC96	0,91

tetratricopeptide repeat domain 19	TTC19	0,91
coiled-coil domain containing 47	CCDC47	0,91
Transcript Identified by AceView, Entrez Gene ID(s) 8615	USO1	0,91
tumor protein p63	TP63	0,91
ribonuclease, RNase A family, k6	RNASE6	0,91
interleukin 1 receptor accessory protein-like 2	IL1RAPL2	0,91
glutamate rich 2	ERICH2	0,91
doublesex and mab-3 related transcription factor 1	DMRT1	0,91
serine/arginine-rich splicing factor 5	SRSF5	0,91
ATP binding cassette subfamily F member 1	ABCF1	0,91
Memczak2013 ALT_ACCEPTOR, coding, INTERNAL, intronic best transcript NM_001080443	KIF18B	0,91
solute carrier family 25 (mitochondrial carrier: glutamate), member 22	SLC25A22	0,91
chemokine (C-C motif) ligand 25	CCL25	0,91
zinc finger with KRAB and SCAN domains 4	ZKSCAN4	0,91
family with sequence similarity 47, member E; FAM47E-STBD1 readthrough; starch binding domain 1	FAM47E; FAM47E-STBD1; STBD1	0,91
chromosome 4 open reading frame 19	C4orf19	0,91
repulsive guidance molecule family member a	RGMA	0,91
transducin-like enhancer of split 6	TLE6	0,91
major histocompatibility complex, class II, DO alpha	HLA-DOA	0,91
5-hydroxytryptamine (serotonin) receptor 5A, G protein-coupled	HTR5A	0,91
kelch repeat and BTB (POZ) domain containing 11	KBTBD11	0,91
uncharacterized LOC101928034; Salzman2013 ANTISENSE, CDS, coding, INTERNAL, intronic, OVCODE, OVEXON, UTR5 best transcript NM_002960	LOC101928034; S100A3	0,91
chromosome 9 open reading frame 153	C9orf153	0,91
cytochrome b5 reductase-like	CYB5RL	0,91
protein phosphatase 2, regulatory subunit A, beta	PPP2R1B	0,91
chromosome 1 open reading frame 61	C1orf61	0,91
methyltransferase like 12; small nucleolar RNA, H/ACA box 57	METTL12; SNORA57	0,91
stathmin-like 3	STMN3	0,91
otopetrin 3	OTOP3	0,91
Jrk helix-turn-helix protein	JRK	0,91
Memczak2013 ALT_ACCEPTOR, ALT_DONOR, coding, INTERNAL, intronic best transcript NM_018482	ASAP1	0,91
CDC42 small effector 2	CDC42SE2	0,91
uncharacterized 100631378; Transcript Identified by AceView, Entrez Gene ID(s) 728853; novel transcript	LOC100631378; AC016582.2; LOC728853	0,91
hydroxymethylbilane synthase	HMBS	0,91

PAS domain containing serine/threonine kinase	PASK	0,91
uncharacterized LOC283731; novel transcript	LOC283731; RP11-247C2.2	0,91
LDL receptor related protein 5 like	LRP5L	0,91
GTPase, IMAP family member 6	GIMAP6	0,91
teashirt zinc finger homeobox 1	TSHZ1	0,91
phosphatidylinositol transfer protein, membrane-associated 1	PITPNM1	0,91
formin binding protein 4	FNBP4	0,91
carbonic anhydrase VI	CA6	0,91
chromosome 7 open reading frame 76; split hand/foot malformation (ectrodactyly) type 1	C7orf76; SHFM1	0,91
microtubule associated protein 6	MAP6	0,91
zinc finger protein 789	ZNF789	0,91
carcinoembryonic antigen-related cell adhesion molecule 21	CEACAM21	0,91
perilipin 5	PLIN5	0,91
PR domain containing 10	PRDM10	0,91
olfactory receptor, family 11, subfamily A, member 1	OR11A1	0,91
high mobility group box 1	HMGB1	0,91
interleukin 10 receptor, beta	IL10RB	0,91
solute carrier family 25 (mitochondrial carrier; ornithine transporter) member 2	SLC25A2	0,91
keratin associated protein 10-9	KRTAP10-9	0,91
heterogeneous nuclear ribonucleoprotein C-like 1	HNRNPCL1	0,91
mitochondrial ribosomal protein L38	MRPL38	0,91
SET nuclear proto-oncogene	SET	0,91
APC membrane recruitment protein 2	AMER2	0,91
potassium channel, voltage gated shaker related subfamily A, member 1	KCNA1	0,91
major histocompatibility complex, class I, F	HLA-F	0,91
olfactory receptor, family 9, subfamily I, member 1	OR9I1	0,91
integrin beta like 1	ITGBL1	0,91
F-box protein 36	FBXO36	0,91
growth differentiation factor 2	GDF2	0,91
NFKB activating protein	NKAP	0,91
F-box and WD repeat domain containing 12	FBXW12	0,91
ADAM metallopeptidase domain 12	ADAM12	0,91
retina and anterior neural fold homeobox	RAX	0,91
dihydrofolate reductase like 1	DHFRL1	0,91
nuclear factor, erythroid 2-like 1	NFE2L1	0,91
synaptotagmin III	SYT3	0,91
tudor and KH domain containing	TDRKH	0,91
solute carrier family 6 (neutral amino acid transporter), member 19	SLC6A19	0,91

killer cell lectin-like receptor subfamily A pseudogene 1	KLRAP1	0,91
ras responsive element binding protein 1	RREB1	0,91
SUMO1/sentrin specific peptidase 5	SENP5	0,91
chromosome 4 open reading frame 51	C4orf51	0,91
motile sperm domain containing 3	MOSPD3	0,91
CUGBP, Elav-like family member 1	CELF1	0,91
long intergenic non-protein coding RNA 1496	LINC01496	0,91
oligodendrocyte myelin glycoprotein	OMG	0,91
ras homolog family member V	RHOV	0,91
transcription factor 25 (basic helix-loop-helix)	TCF25	0,91
Transcript Identified by AceView, Entrez Gene ID(s) 143686	SESN3	0,91
cytochrome P450, family 26, subfamily B, polypeptide 1	CYP26B1	0,91
taxilin alpha	TXLNA	0,91
chromosome X open reading frame 40B	CXorf40B	0,91
cathepsin Z	CTSZ	0,91
C-type lectin domain family 10, member A	CLEC10A	0,91
StAR-related lipid transfer domain containing 5	STARD5	0,91
heterogeneous nuclear ribonucleoprotein C-like 4 [Source:HGNC Symbol;Acc:HGNC:51333]	HNRNPCL4	0,91
iron-sulfur cluster assembly 2	ISCA2	0,91
chromosome 1 open reading frame 159	C1orf159	0,91
trophinin associated protein	TROAP	0,91
succinyl-CoA:glutarate-CoA transferase	SUGCT	0,91
poly(A)-specific ribonuclease (PARN)-like domain containing 1	PNLDC1	0,91
dual oxidase maturation factor 2	DUOXA2	0,91
forkhead box I2	FOXI2	0,91
mitogen-activated protein kinase 7	MAPK7	0,91
NHS-like 2	NHSL2	0,91
coiled-coil domain containing 137	CCDC137	0,91
family with sequence similarity 109, member A	FAM109A	0,91
proprotein convertase subtilisin/kexin type 1 inhibitor	PCSK1N	0,91
calcium-sensing receptor	CASR	0,91
ES cell expressed Ras	ERAS	0,91
endothelin receptor type B	EDNRB	0,91
NADH dehydrogenase (ubiquinone) 1 alpha subcomplex, 10, 42kDa	NDUFA10	0,91
chromosome 22 open reading frame 42	C22orf42	0,91
mitochondrial ribosomal protein L28	MRPL28	0,91
guanine nucleotide binding protein (G protein), q polypeptide	GNAQ	0,91
ubiquinol-cytochrome c reductase hinge protein	UQCRH	0,91
dual specificity phosphatase 26 (putative)	DUSP26	0,91
cytochrome P450, family 2, subfamily C, polypeptide 9	CYP2C9	0,91

ubiquinol-cytochrome c reductase complex assembly factor 1	UQCC1	0,91
centromere protein B	CENPB	0,91
erb-b2 receptor tyrosine kinase 4	ERBB4	0,91
relaxin/insulin-like family peptide receptor 3	RXFP3	0,91
forkhead box D4	FOXD4	0,91
olfactory receptor, family 4, subfamily N, member 2	OR4N2	0,91
zinc finger protein 280D	ZNF280D	0,91
lysozyme-like 6	LYZL6	0,91
signal sequence receptor, beta (translocon-associated protein beta)	SSR2	0,91
anthrax toxin receptor-like	ANTXRL	0,91
N-acetyltransferase 8 (GCN5-related, putative)	NAT8	0,91
glycoprotein Ib (platelet), alpha polypeptide	GP1BA	0,91
Transcript Identified by AceView, Entrez Gene ID(s) 2098	ESD	0,91
methionine sulfoxide reductase A	MSRA	0,91
coiled-coil domain containing 178	CCDC178	0,91
lymphocyte antigen 6 complex, locus G6F	LY6G6F	0,91
HLA complex group 27 (non-protein coding)	HCG27	0,91
SWI/SNF related, matrix associated, actin dependent regulator of chromatin, subfamily a-like 1	SMARCAL1	0,91
nuclear receptor subfamily 2, group E, member 3	NR2E3	0,91
diacylglycerol O-acyltransferase 2	DGAT2	0,91
keratin associated protein 19-7	KRTAP19-7	0,91
methyltransferase like 20	METTL20	0,91
replication protein A2	RPA2	0,91
olfactory receptor, family 4, subfamily C, member 3	OR4C3	0,91
pyroglutamylated RFamide peptide receptor	QRFPR	0,91
docking protein 3	DOK3	0,91
ubiquitin specific peptidase 10	USP10	0,91
pannexin 3	PANX3	0,91
adrenoceptor alpha 2B	ADRA2B	0,91
ribosomal L24 domain containing 1	RSL24D1	0,91
S-antigen; retina and pineal gland (arrestin)	SAG	0,91
cytochrome c oxidase subunit Va	COX5A	0,91
regulator of G-protein signaling like 1	RGSL1	0,91
O-6-methylguanine-DNA methyltransferase	MGMT	0,91
membrane-spanning 4-domains, subfamily A, member 2	MS4A2	0,91
outer dense fiber of sperm tails 4	ODF4	0,91
phospholipase C-like 2; microRNA 3714	PLCL2; MIR3714	0,91
NOP2/Sun domain family, member 3	NSUN3	0,91
apolipoprotein B mRNA editing enzyme, catalytic polypeptide-like 3D	APOBEC3D	0,91

synaptosome associated protein 47kDa	SNAP47	0,91
reprimo, TP53 dependent G2 arrest mediator candidate	RPRM	0,91
family with sequence similarity 83, member E	FAM83E	0,91
NADH dehydrogenase (ubiquinone) 1 beta subcomplex, 7, 18kDa	NDUFB7	0,91
5-hydroxytryptamine (serotonin) receptor 2C, G protein-coupled	HTR2C	0,91
Homo sapiens POTE ankyrin domain family, member B3 (POTEB3), transcript variant 1, mRNA.; Homo sapiens POTE ankyrin domain family, member B, mRNA (cDNA clone MGC:119371 IMAGE:40006483), complete cds.; Homo sapiens POTE ankyrin domain family, member B, mRNA (cDNA clone MGC:119372 IMAGE:40006485), complete cds.; Homo sapiens POTE ankyrin domain family, member B, mRNA (cDNA clone MGC:119373 IMAGE:40006489), complete cds.; Homo sapiens POTE ankyrin domain family, member B, mRNA (cDNA clone MGC:156116 IMAGE:40006490), complete cds.	POTEB3; POTEB; AC136352.4	0,91
ubiquitin specific peptidase 26	USP26	0,91
sushi domain containing 1	SUSD1	0,91
RAB40A, member RAS oncogene family-like	RAB40AL	0,91
olfactory receptor, family 4, subfamily E, member 2	OR4E2	0,91
sulfatase 1	SULF1	0,91
DnaJ (Hsp40) homolog, subfamily C, member 7	DNAJC7	0,91
KIF1 binding protein	KIF1BP	0,91
phospholipase C, epsilon 1	PLCE1	0,91
protein disulfide isomerase family A, member 2	PDIA2	0,91
Memczak2013 ALT_ACCEPTOR, ALT_DONOR, coding, INTERNAL, intronic best transcript NM_022480	KLHL25	0,91
SAM domain and HD domain 1	SAMHD1	0,91
transcription factor B2, mitochondrial	TFB2M	0,91
olfactory receptor, family 5, subfamily AP, member 2	OR5AP2	0,91
Memczak2013 ANTISENSE, coding, INTERNAL, UTR3 best transcript NM_000565	IL6R	0,91
nodal growth differentiation factor	NODAL	0,91
retinitis pigmentosa 9 (autosomal dominant)	RP9	0,91
heat shock 70kD protein 12B	HSPA12B	0,91
makorin ring finger protein 2	MKRN2	0,91
tripartite motif containing 26	TRIM26	0,91
golgi reassembly stacking protein 2	GORASP2	0,91
odorant binding protein 2B	OBP2B	0,91
ubiquinol-cytochrome c reductase core protein II	UQCRC2	0,91
salt-inducible kinase 1	SIK1	0,91
transmembrane protein 100	TMEM100	0,91
stathmin 2	STMN2	0,91

family with sequence similarity 163, member A	FAM163A	0,91
prostaglandin-endoperoxide synthase 1 (prostaglandin G/H synthase and cyclooxygenase)	PTGS1	0,91
ubiquitin-like 4B	UBL4B	0,91
cortexin 3	CTXN3	0,91
hydroxysteroid (17-beta) dehydrogenase 3	HSD17B3	0,91
Transcript Identified by AceView, Entrez Gene ID(s) 23081	KDM4C	0,91
ATP11A upstream neighbor	ATP11AUN	0,91
pancreatic lipase	PNLIP	0,91
cat eye syndrome chromosome region, candidate 2	CECR2	0,91
kelch domain containing 9	KLHDC9	0,91
Rhox homeobox family, member 2B; Rhox homeobox family, member 2	RHOXF2B; RHOXF2	0,91
haloacid dehalogenase-like hydrolase domain containing 2	HDHD2	0,91
thyroid hormone receptor associated protein 3	THRAP3	0,90
Rho guanine nucleotide exchange factor 37	ARHGEF37	0,90
lipocalin 1	LCN1	0,90
dual adaptor of phosphotyrosine and 3-phosphoinositides	DAPP1	0,90
potassium channel, voltage gated modifier subfamily F, member 1	KCNF1	0,90
keratin associated protein 29-1	KRTAP29-1	0,90
leucine rich repeat containing 17	LRRC17	0,90
HGH1 homolog	HGH1	0,90
fructosamine 3 kinase related protein	FN3KRP	0,90
chloride channel accessory 1	CLCA1	0,90
ELKS/RAB6-interacting/CAST family member 2	ERC2	0,90
tyrosylprotein sulfotransferase 2	TPST2	0,90
chromosome 3 open reading frame 17	C3orf17	0,90
solute carrier family 25 (carnitine/acylcarnitine translocase), member 20	SLC25A20	0,90
NME/NM23 nucleoside diphosphate kinase 1; NME1-NME2 readthrough; NME/NM23 nucleoside diphosphate kinase 2	NME1; NME1-NME2; NME2	0,90
zinc finger protein 419	ZNF419	0,90
chromosome 12 open reading frame 66	C12orf66	0,90
protein phosphatase 1, regulatory subunit 12C	PPP1R12C	0,90
olfactory receptor, family 4, subfamily K, member 2	OR4K2	0,90
olfactory receptor, family 7, subfamily C, member 2	OR7C2	0,90
amyloid beta precursor protein (cytoplasmic tail) binding protein 2	APPBP2	0,90
MAP-kinase activating death domain	MADD	0,90
ariadne homolog 2 opposite strand	ARIH2OS	0,90

diacylglycerol kinase, delta 130kDa	DGKD	0,90
COP9 signalosome subunit 7A	COPS7A	0,90
dermcidin	DCD	0,90
lysophosphatidic acid receptor 4	LPAR4	0,90
secretoglobin, family 1C, member 1	SCGB1C1	0,90
U2 small nuclear RNA auxiliary factor 1	U2AF1	0,90
RPL36A-HNRNPH2 readthrough	RPL36A-HNRNPH2	0,90
mitochondrial ribosomal protein S12	MRPS12	0,90
dopamine receptor D3	DRD3	0,90
olfactory receptor, family 2, subfamily L, member 8 (gene/pseudogene)	OR2L8	0,90
Fc fragment of IgG, low affinity IIb, receptor (CD32)	FCGR2B	0,90
cytochrome b5 type B (outer mitochondrial membrane)	CYB5B	0,90
tubulin folding cofactor C	TBCC	0,90
phosducin like	PDCL	0,90
long intergenic non-protein coding RNA 649	LINC00649	0,90
tripartite motif containing 49B	TRIM49B	0,90
translocase of inner mitochondrial membrane 8 homolog B (yeast)	TIMM8B	0,90
synovial sarcoma translocation gene on chromosome 18-like 2	SS18L2	0,90
ribosomal protein S6 kinase-like 1	RPS6KL1	0,90
low density lipoprotein receptor class A domain containing 1	LDLRAD1	0,90
trinucleotide repeat containing 18	TNRC18	0,90
FCH domain only 1	FCHO1	0,90
small nuclear ribonucleoprotein, U5 200kDa subunit	SNRNP200	0,90
RAB32, member RAS oncogene family	RAB32	0,90
CD300 molecule-like family member g	CD300LG	0,90
ST8 alpha-N-acetyl-neuraminide alpha-2,8-sialyltransferase 1	ST8SIA1	0,90
uncharacterized LOC100134391; novel transcript	LOC100134391; CTD-2532D12.4	0,90
FERM domain containing 6	FRMD6	0,90
chromosome 9 open reading frame 135	C9orf135	0,90
EF-hand domain family, member B	EFHB	0,90
solute carrier family 22, member 24	SLC22A24	0,90
zinc finger protein 625	ZNF625	0,90
DPY30 domain containing 1	DYDC1	0,90
MARCKS-like 1	MARCKSL1	0,90
solute carrier family 25 (mitochondrial carrier; oxoglutarate carrier), member 11	SLC25A11	0,90
sperm acrosome associated 6	SPACA6	0,90
myogenic differentiation 1	MYOD1	0,90

cholecystokinin B receptor	CCKBR	0,90
actin-related protein T2	ACTRT2	0,90
opsin 1 (cone pigments), long-wave-sensitive	OPN1LW	0,90
zinc finger protein 354B	ZNF354B	0,90
solute carrier family 16 (monocarboxylate transporter), member 1	SLC16A1	0,90
von Willebrand factor D and EGF domains	VWDE	0,90
paired box 2	PAX2	0,90
v-ets avian erythroblastosis virus E26 oncogene homolog	ERG	0,90
olfactory receptor, family 14, subfamily I, member 1	OR14I1	0,90
keratin associated protein 7-1 (gene/pseudogene)	KRTAP7-1	0,90
cadherin 22, type 2	CDH22	0,90
Memczak2013 ALT_ACCEPTOR, ALT_DONOR, coding, INTERNAL, intronic best transcript NM_004152	OAZ1	0,90
Rh family, C glycoprotein	RHCG	0,90
RNA polymerase II associated protein 2	RPAP2	0,90
claudin 23	CLDN23	0,90
RIO kinase 2	RIOK2	0,90
TBC1 domain family, member 21	TBC1D21	0,90
cyclin T2	CCNT2	0,90
sorting nexin 12	SNX12	0,90
apolipoprotein L, 2	APOL2	0,90
family with sequence similarity 101, member A	FAM101A	0,90
olfactory receptor, family 52, subfamily Z, member 1 (gene/pseudogene)	OR52Z1	0,90
keratin 3, type II	KRT3	0,90
parvin, gamma	PARVG	0,90
diacylglycerol kinase, iota	DGKI	0,90
WD repeat domain 12	WDR12	0,90
PLAC8-like 1	PLAC8L1	0,90
WD repeat domain 36	WDR36	0,90
protein phosphatase, Mg2+/Mn2+ dependent, 1L	PPM1L	0,90
REC8 meiotic recombination protein	REC8	0,90
selectin P (granule membrane protein 140kDa, antigen CD62)	SELP	0,90
peripherin 2 (retinal degeneration, slow)	PRPH2	0,90
myosin binding protein C, slow type	MYBPC1	0,90
serine peptidase inhibitor, Kazal type 7 (putative)	SPINK7	0,90
G1 to S phase transition 2	GSPT2	0,90
splicing factor, suppressor of white-apricot family	SFSWAP	0,90
G protein-coupled receptor 15	GPR15	0,90
zinc finger, B-box domain containing	ZBBX	0,90
bone morphogenetic protein 15	BMP15	0,90

tripartite motif containing 63, E3 ubiquitin protein ligase	TRIM63	0,90
neuronal pentraxin II	NPTX2	0,90
polymerase (RNA) II (DNA directed) polypeptide D	POLR2D	0,90
lipase, member I	LIPI	0,90
isoprenoid synthase domain containing	ISPD	0,90
Jeck2013 ALT_DONOR, coding, INTERNAL, intronic best transcript NM_147780	CTSB	0,90
olfactory receptor, family 4, subfamily C, member 13	OR4C13	0,90
bifunctional apoptosis regulator	BFAR	0,90
heterogeneous nuclear ribonucleoprotein H1 (H)	HNRNPH1	0,90
cytochrome P450, family 2, subfamily D, polypeptide 6	CYP2D6	0,90
N-acetyltransferase 16 (GCN5-related, putative)	NAT16	0,90
G protein-coupled receptor 78	GPR78	0,90
long intergenic non-protein coding RNA 1620	LINC01620	0,90
zinc finger protein 431; vomeronasal 1 receptor 82 pseudogene	ZNF431; VN1R82P	0,90
superoxide dismutase 1, soluble	SOD1	0,90
Rho guanine nucleotide exchange factor 16	ARHGEF16	0,90
Fanconi anemia complementation group E	FANCE	0,90
Transcript Identified by AceView, Entrez Gene ID(s) 51633	OTUD6B	0,90
chemokine (C-C motif) receptor 3	CCR3	0,90
coiled-coil domain containing 7	CCDC7	0,90
cytochrome c oxidase subunit VIIb	COX7B	0,90
transient receptor potential cation channel, subfamily V, member 6	TRPV6	0,90
protein tyrosine phosphatase, receptor type, H	PTPRH	0,90
small integral membrane protein 22	SMIM22	0,90
tumor necrosis factor, alpha-induced protein 8-like 3	TNFAIP8L3	0,90
atonal bHLH transcription factor 1	ATOH1	0,90
receptor-associated protein of the synapse	RAPSN	0,90
phosphodiesterase 1A, calmodulin-dependent	PDE1A	0,90
solute carrier family 35, member E2	SLC35E2	0,90
heparan sulfate 6-O-sulfotransferase 3	HS6ST3	0,90
ornithine decarboxylase antizyme 3	OAZ3	0,90
abhydrolase domain containing 15	ABHD15	0,90
olfactory receptor, family 4, subfamily K, member 15	OR4K15	0,90
circadian associated repressor of transcription	CIART	0,90
KIAA0232	KIAA0232	0,90
ring finger protein 212B	RNF212B	0,90
interferon, kappa	IFNK	0,90
tudor domain containing 15	TDRD15	0,90
ankyrin repeat domain 63	ANKRD63	0,90
arginine vasopressin receptor 1A	AVPR1A	0,90

chromosome X open reading frame 49B	CXorf49B	0,90
F-box protein 27	FBXO27	0,90
acetylcholinesterase (Yt blood group)	ACHE	0,90
Memczak2013 ANTISENSE, coding, INTERNAL, intronic best transcript NM_001198696	AIFM2	0,90
crumbs family member 2	CRB2	0,90
histamine receptor H3	HRH3	0,90
solute carrier family 31 (copper transporter), member 2	SLC31A2	0,90
collectin sub-family member 12	COLEC12	0,90
Memczak2013 ANTISENSE, coding, INTERNAL, intronic best transcript NM_020235	BBX	0,90
equatorin, sperm acrosome associated	EQTN	0,90
opsin 1 (cone pigments), medium-wave-sensitive	OPN1MW	0,90
dynein, axonemal, heavy chain 3	DNAH3	0,90
coiled-coil domain containing 130	CCDC130	0,90
nucleotide binding protein 1	NUBP1	0,90
ABI family, member 3	ABI3	0,90
leucine zipper, down-regulated in cancer 1-like	LDOC1L	0,90
gamma-aminobutyric acid (GABA) A receptor, alpha 3	GABRA3	0,90
dopamine receptor D4	DRD4	0,90
Transcript Identified by AceView, Entrez Gene ID(s) 79844	ZDHHC11	0,90
MIS18 kinetochore protein A	MIS18A	0,90
solute carrier family 35, member D3	SLC35D3	0,90
taste receptor, type 2, member 3	TAS2R3	0,90
podocan	PODN	0,90
chemokine (C-C motif) receptor 9	CCR9	0,90
mitochondrial transcription termination factor 3	MTERF3	0,90
YME1-like 1 ATPase	YME1L1	0,90
septin 14	sept-14	0,90
v-maf avian musculoaponeurotic fibrosarcoma oncogene homolog A	MAFA	0,90
tripartite motif containing 58	TRIM58	0,90
calcium homeostasis modulator 3	CALHM3	0,90
mab-21-like 1 (C. elegans)	MAB21L1	0,90
chromosome 11 open reading frame 96	C11orf96	0,90
olfactory receptor, family 52, subfamily K, member 1	OR52K1	0,90
NK2 homeobox 6	NKX2-6	0,90
opioid receptor, mu 1	OPRM1	0,90
cadherin 4, type 1, R-cadherin (retinal)	CDH4	0,90
forkhead box D4-like 5	FOXD4L5	0,90
deiodinase, iodothyronine, type II	DIO2	0,90
cyclin-dependent kinase inhibitor 2D (p19, inhibits CDK4)	CDKN2D	0,90
solute carrier family 35, member G5	SLC35G5	0,90

E1A binding protein p300; microRNA 1281	EP300; MIR1281	0,90
autophagy related 4C, cysteine peptidase	ATG4C	0,90
BLK proto-oncogene, Src family tyrosine kinase	BLK	0,90
chemokine (C-X-C motif) ligand 13	CXCL13	0,90
mitochondrial ribosomal protein S22	MRPS22	0,90
ectonucleotide pyrophosphatase/phosphodiesterase 4 (putative)	ENPP4	0,90
achaete-scute family bHLH transcription factor 1	ASCL1	0,90
kyphoscoliosis peptidase	KY	0,90
Jeck2013 ALT_ACCEPTOR, ALT_DONOR, coding, INTERNAL, intronic best transcript NM_006909	RASGRF2	0,90
CD69 molecule	CD69	0,90
TBC1 domain family, member 23	TBC1D23	0,90
calcium channel, voltage-dependent, alpha 2/delta subunit 2	CACNA2D2	0,90
ArfGAP with GTPase domain, ankyrin repeat and PH domain 9; BMS1 ribosome biogenesis factor pseudogene 6	AGAP9; BMS1P6	0,90
solute carrier family 1 (neuronal/epithelial high affinity glutamate transporter, system Xag), member 1	SLC1A1	0,90
hypocretin (orexin) neuropeptide precursor	HCRT	0,90
cytoplasmic linker associated protein 1	CLASP1	0,90
trichohyalin	TCHH	0,90
MCF.2 cell line derived transforming sequence	MCF2	0,90
forkhead box D2	FOXD2	0,90
BIN3 intronic transcript 1	BIN3-IT1	0,90
kinocilin	KNCN	0,90
Transcript Identified by AceView, Entrez Gene ID(s) 79719	AAGAB	0,90
acyl-CoA thioesterase 11	ACOT11	0,90
transmembrane protein 88B	TMEM88B	0,90
odorant binding protein 2B	OBP2B	0,90
small G protein signaling modulator 2	SGSM2	0,90
keratin associated protein 24-1	KRTAP24-1	0,90
Memczak2013 ALT_ACCEPTOR, ALT_DONOR, coding, INTERNAL, intronic best transcript NM_006586	CNPY3	0,90
elongator acetyltransferase complex subunit 3	ELP3	0,90
Memczak2013 ANTISENSE, coding, INTERNAL, UTR3 best transcript NM_006378	SEMA4D	0,90
collagen, type X, alpha 1	COL10A1	0,90
ERI1 exoribonuclease family member 3; ERI3 intronic transcript 1	ERI3; ERI3-IT1	0,90
polymerase (RNA) III (DNA directed) polypeptide H (22.9kD)	POLR3H	0,90
kinesin family member 4B	KIF4B	0,90

cell adhesion molecule L1-like	CHL1	0,90
alcohol dehydrogenase 7 (class IV), mu or sigma polypeptide	ADH7	0,90
Ca++-dependent secretion activator	CADPS	0,90
RAB12, member RAS oncogene family	RAB12	0,90
membrane-spanning 4-domains, subfamily A, member 18	MS4A18	0,90
zinc finger protein 527	ZNF527	0,90
tetratricopeptide repeat domain 30B	TTC30B	0,90
proteasome 26S subunit, ATPase 4 [Source:HGNC Symbol;Acc:HGNC:9551]	PSMC4	0,90
golgi brefeldin A resistant guanine nucleotide exchange factor 1	GBF1	0,90
sulfotransferase family 1A member 2	SULT1A2	0,90
insulin; INS-IGF2 readthrough	INS; INS-IGF2	0,90
leucine rich repeat containing 47	LRRC47	0,90
synaptotagmin IX	SYT9	0,90
transmembrane protein 150A	TMEM150A	0,90
transmembrane protein 200B	TMEM200B	0,90
testis expressed 30	TEX30	0,90
EGF-like-domain, multiple 6	EGFL6	0,90
egl-9 family hypoxia-inducible factor 3	EGLN3	0,90
chromosome 1 open reading frame 168	C1orf168	0,90
insulin like growth factor binding protein-like 1	IGFBPL1	0,90
zinc finger protein 521	ZNF521	0,90
protein-L-isoaspartate (D-aspartate) O-methyltransferase domain containing 1	PCMTD1	0,90
TBC1 domain family, member 17; microRNA 4750	TBC1D17; MIR4750	0,90
frequently rearranged in advanced T-cell lymphomas 2	FRAT2	0,90
ubiquitin-like modifier activating enzyme 6	UBA6	0,90
meiotic double-stranded break formation protein 4	MEI4	0,90
dynein regulatory complex subunit 7	DRC7	0,90
pepsinogen 4, group I (pepsinogen A)	PGA4	0,90
complement component (3d/Epstein Barr virus) receptor 2	CR2	0,90
solute carrier family 45, member 4	SLC45A4	0,90
F-box and leucine-rich repeat protein 15	FBXL15	0,90
zinc finger protein 524	ZNF524	0,90
olfactory receptor, family 11, subfamily H, member 6	OR11H6	0,90
RANBP2-like and GRIP domain containing 2; RANBP2-like and GRIP domain containing 1	RGPD2; RGPD1	0,90
opsin 1 (cone pigments), short-wave-sensitive	OPN1SW	0,90
C-type lectin domain family 4, member C	CLEC4C	0,90
prolactin releasing hormone receptor	PRLHR	0,90

jagged 2	JAG2	0,90
family with sequence similarity 19 (chemokine (C-C motif)-like), member A2	FAM19A2	0,90
family with sequence similarity 189, member A1	FAM189A1	0,90
acyl-CoA thioesterase 6	ACOT6	0,90
potassium channel, two pore domain subfamily K, member 13	KCNK13	0,90
makorin ring finger protein 1	MKRN1	0,90
SET binding factor 1	SBF1	0,90
kinesin family member 23	KIF23	0,90
aspartate beta-hydroxylase domain containing 2	ASPHD2	0,90
transmembrane protein 191C	TMEM191C	0,90
protease, serine, 2 (trypsin 2)	PRSS2	0,90
adiponectin, C1Q and collagen domain containing	ADIPOQ	0,90
proteasome subunit alpha 4	PSMA4	0,90
major facilitator superfamily domain containing 4	MFSD4	0,90
oxytocin/neurophysin I prepropeptide	OXT	0,90
K(lysine) acetyltransferase 6B	KAT6B	0,90
vesicle transport through interaction with t-SNAREs 1B	VTI1B	0,90
Bruton agammaglobulinemia tyrosine kinase	BTK	0,90
long intergenic non-protein coding RNA 1359	LINC01359	0,90
Mab-21 domain containing 2	MB21D2	0,90
epsin 3	EPN3	0,90
small ubiquitin-like modifier 3	SUMO3	0,90
melanocortin 1 receptor (alpha melanocyte stimulating hormone receptor)	MC1R	0,90
kelch-like family member 6	KLHL6	0,90
macrophage receptor with collagenous structure	MARCO	0,90
dihydrouridine synthase 2	DUS2	0,90
nucleophosmin/nucleoplasmin 2	NPM2	0,90
Transcript Identified by AceView, Entrez Gene ID(s) 375775	PNPLA7	0,90
retinal degeneration 3	RD3	0,90
transmembrane protein 247	TMEM247	0,90
Transcript Identified by AceView, Entrez Gene ID(s) 54715	A2BP1	0,90
ALX homeobox 4	ALX4	0,90
ubiquitin associated and SH3 domain containing A	UBASH3A	0,90
clathrin interactor 1	CLINT1	0,90
intercellular adhesion molecule 4 (Landsteiner-Wiener blood group)	ICAM4	0,90
ubiquitin specific peptidase 49	USP49	0,90
CD84 molecule	CD84	0,90
family with sequence similarity 157, member A	FAM157A	0,90
DDB1 and CUL4 associated factor 8	DCAF8	0,90

zinc finger protein 584	ZNF584	0,90
ALG10B, alpha-1,2-glucosyltransferase	ALG10B	0,90
zinc finger protein 705D	ZNF705D	0,90
DDB1 and CUL4 associated factor 12-like 1	DCAF12L1	0,90
Transcript Identified by AceView, Entrez Gene ID(s) 7082	TJP1	0,90
olfactory receptor, family 3, subfamily A, member 2	OR3A2	0,90
Transcript Identified by AceView, Entrez Gene ID(s) 23028	KDM1A	0,90
adenomatosis polyposis coli down-regulated 1-like	APCDD1L	0,90
olfactory receptor, family 4, subfamily D, member 9	OR4D9	0,90
Jeck2013 ANTISENSE, coding, INTERNAL, intronic best transcript NM_001130100	KIFC3	0,90
ubiquitin-like 4A	UBL4A	0,90
phosphoribosylformylglycinamidine synthase	PFAS	0,90
amyloid beta (A4) precursor protein-binding, family A, member 2	APBA2	0,90
transducer of ERBB2, 2	TOB2	0,90
cyclin-dependent kinase 12	CDK12	0,90
zinc finger protein 546	ZNF546	0,90
DiGeorge syndrome critical region gene 5 (non-protein coding); DiGeorge syndrome critical region gene 9 (non-protein coding)	DGCR5; DGCR9	0,90
Jeck2013 ANTISENSE, CDS, coding, INTERNAL, intronic, OVCODE, OVEXON best transcript NM_058175	COL6A2	0,90
RNA binding motif protein 26	RBM26	0,90
transmembrane protein 80	TMEM80	0,90
stromal antigen 2	STAG2	0,90
Memczak2013 ANTISENSE, CDS, coding, INTERNAL, UTR3 best transcript NM_001080533	UNC119B	0,90
GRB2-related adaptor protein 2	GRAP2	0,90
zinc finger protein 446	ZNF446	0,90
SUMO1/sentrin/SMT3 specific peptidase 2	SENP2	0,90
TGF-beta activated kinase 1/MAP3K7 binding protein 2	TAB2	0,90
zinc finger protein 837	ZNF837	0,90
glutamyl aminopeptidase (aminopeptidase A)	ENPEP	0,90
TBC1 domain family, member 3B; TBC1 domain family, member 3H; TBC1 domain family, member 3I; TBC1 domain family, member 3F; TBC1 domain family, member 3G	TBC1D3B; TBC1D3H; TBC1D3I; TBC1D3F; TBC1D3G	0,90
ENTH domain containing 1	ENTHD1	0,90
sperm acrosome associated 1	SPACA1	0,89
zinc finger with KRAB and SCAN domains 7	ZKSCAN7	0,89
gliomedin	GLDN	0,89

RAN binding protein 17	RANBP17	0,89
speedy/RINGO cell cycle regulator family member E6; speedy/RINGO cell cycle regulator family member E2	SPDYE6; SPDYE2	0,89
regenerating islet-derived family, member 4	REG4	0,89
vomeronasal 1 receptor 4	VN1R4	0,89
regulator of G-protein signaling 17	RGS17	0,89
olfactory receptor, family 7, subfamily A, member 10	OR7A10	0,89
tyrosine aminotransferase	TAT	0,89
Transcript Identified by AceView, Entrez Gene ID(s) 10777	ARPP21	0,89
SPO11 meiotic protein covalently bound to DSB	SPO11	0,89
MAGE family member E1	MAGEE1	0,89
C1GALT1-specific chaperone 1 like	C1GALT1C1L	0,89
Transcript Identified by AceView, Entrez Gene ID(s) 84173	ELMOD3	0,89
SRA stem-loop interacting RNA binding protein	SLIRP	0,89
caspase 6	CASP6	0,89
chondroitin sulfate proteoglycan 4 pseudogene 1, Y-linked; dynamin 1 pseudogene 24	CSPG4P1Y; DNM1P24	0,89
ribosomal protein S6 kinase, 90kDa, polypeptide 1	RPS6KA1	0,89
Memczak2013 ANTISENSE, CDS, coding, INTERNAL best transcript NM_006623	PHGDH	0,89
runt-related transcription factor 3	RUNX3	0,89
zinc finger protein 215	ZNF215	0,89
DEAD (Asp-Glu-Ala-Asp) box polypeptide 19A	DDX19A	0,89
inositol 1,4,5-trisphosphate receptor interacting protein-like 2	ITPRIPL2	0,89
bone marrow stromal cell antigen 1	BST1	0,89
eukaryotic translation initiation factor 2, subunit 2 beta, 38kDa	EIF2S2	0,89
arginine vasopressin receptor 1B	AVPR1B	0,89
sulfotransferase family 6B member 1	SULT6B1	0,89
TBCC domain containing 1	TBCCD1	0,89
Cbp/p300-interacting transactivator, with Glu/Asp rich carboxy-terminal domain, 1	CITED1	0,89
keratin associated protein 16-1	KRTAP16-1	0,89
uncharacterized LOC101927550; Salzman2013 ANTISENSE, CDS, coding, INTERNAL, intronic, OVCODE, OVERLAPTX, OVEXON best transcript NM_020429; novel transcript	LOC101927550; AC004893.11; SMURF1	0,89
ankyrin repeat and SOCS box containing 18	ASB18	0,89
protocadherin 11 X-linked	PCDH11X	0,89
adenosine deaminase domain containing 2	ADAD2	0,89
tumor necrosis factor receptor superfamily, member 21	TNFRSF21	0,89
KIAA1328	KIAA1328	0,89

MRVI1 antisense RNA 1	MRVI1-AS1	0,89
TBC1 domain family, member 31	TBC1D31	0,89
dihydropyrimidinase-like 3	DPYSL3	0,89
msh homeobox 1	MSX1	0,89
dachsous cadherin-related 1	DCHS1	0,89
RBBP8 N-terminal like	RBBP8NL	0,89
spermatogenesis associated 3	SPATA3	0,89
leucine-rich repeats and transmembrane domains 1	LRTM1	0,89
netrin 3	NTN3	0,89
solute carrier family 5 (sodium/glucose cotransporter), member 2	SLC5A2	0,89
SMAD family member 1	SMAD1	0,89
cyclic nucleotide gated channel alpha 1	CNGA1	0,89
ATPase, Na+/K+ transporting, beta 2 polypeptide	ATP1B2	0,89
major histocompatibility complex, class II, DR alpha	HLA-DRA	0,89
mitochondrial ribosomal protein L15	MRPL15	0,89
hydroxymethylbilane synthase	HMBS	0,89
UDP-GlcNAc:betaGal beta-1,3-N-acetylglucosaminyltransferase 6	B3GNT6	0,89
transmembrane protein 259	TMEM259	0,89
cholesterol 25-hydroxylase	CH25H	0,89
stimulated by retinoic acid 8	STRA8	0,89
ankyrin repeat domain 61	ANKRD61	0,89
solute carrier family 25, member 47	SLC25A47	0,89
STT3A, subunit of the oligosaccharyltransferase complex (catalytic)	STT3A	0,89
upstream binding protein 1 (LBP-1a)	UBP1	0,89
solute carrier family 52 (riboflavin transporter), member 1	SLC52A1	0,89
growth arrest-specific 7	GAS7	0,89
poly(A) polymerase beta	PAPOLB	0,89
coiled-coil domain containing 167	CCDC167	0,89
secretoglobin, family 3A, member 1	SCGB3A1	0,89

Homo sapiens A kinase (PRKA) anchor protein 17A (AKAP17A), transcript variant 1, mRNA.; Homo sapiens A kinase (PRKA) anchor protein 17A (AKAP17A), transcript variant 2, non-coding RNA.; A kinase (PRKA) anchor protein 17A [Source:HGNC Symbol;Acc:HGNC:18783]; Homo sapiens splicing factor, arginine/serine-rich 17A, mRNA (cDNA clone MGC:39904 IMAGE:5217247), complete cds.; Homo sapiens splicing factor, arginine/serine-rich 17A, mRNA (cDNA clone MGC:125365 IMAGE:40007662), complete cds.; Homo sapiens splicing factor, arginine/serine-rich 17A, mRNA (cDNA clone MGC:125366 IMAGE:40007663), complete cds.; Jeck2013 ANNOTATED, INTERNAL, ncRNA, OVCODE, OVEXON best transcript NR_027383	AKAP17A; SFRS17A	0,89
zinc finger, DHHC-type containing 3	ZDHHC3	0,89
Homo sapiens TBC1 domain family, member 3H (TBC1D3H), mRNA.; Homo sapiens TBC1 domain family, member 3G (TBC1D3G), mRNA.; Homo sapiens TBC1 domain family, member 3I (TBC1D3I), mRNA.; Homo sapiens TBC1 domain family member-like (LOC101060389), mRNA.; Homo sapiens TBC1 domain family, member 3C, mRNA (cDNA clone MGC:87891 IMAGE:5165385), complete cds.; Homo sapiens TBC1 domain family, member 3, mRNA (cDNA clone MGC:87893 IMAGE:5742850), complete cds.	TBC1D3H; TBC1D3G; TBC1D3I; LOC101060389; TBC1D3C; TBC1D3	0,89
L-2-hydroxyglutarate dehydrogenase	L2HGDH	0,89
SURP and G-patch domain containing 1	SUGP1	0,89
Memczak2013 ALT_ACCEPTOR, ALT_DONOR, INTERNAL, intronic, ncRNA best transcript NR_045602	GALNT14	0,89
RAB41, member RAS oncogene family	RAB41	0,89
hypoxia up-regulated 1	HYOU1	0,89
tudor domain containing 5	TDRD5	0,89
zinc finger, matrin-type 4	ZMAT4	0,89
exonuclease 5	EXO5	0,89
testis specific protein, Y-linked 8	TSPY8	0,89
nucleoporin 88kDa	NUP88	0,89
secretory carrier membrane protein 1	SCAMP1	0,89
tripartite motif containing 61	TRIM61	0,89
ybeY metallopeptidase (putative)	YBEY	0,89
mediator complex subunit 8	MED8	0,89
ADAM metallopeptidase with thrombospondin type 1 motif 13	ADAMTS13	0,89
chromosome 8 open reading frame 76	C8orf76	0,89
olfactory receptor, family 10, subfamily G, member 3	OR10G3	0,89

chromosome 2 open reading frame 91	C2orf91	0,89
DMRT-like family C1; family with sequence similarity 226, member B (non-protein coding); family with sequence similarity 226, member A (non-protein coding); DMRT-like family C1B	DMRTC1; FAM226B; FAM226A; DMRTC1B	0,89
defensin, beta 4A	DEFB4A	0,89
Hermansky-Pudlak syndrome 6	HPS6	0,89
family with sequence similarity 178, member B	FAM178B	0,89
collectin subfamily member 11	COLEC11	0,89
spindle and centriole associated protein 1	SPICE1	0,89
family with sequence similarity 120B	FAM120B	0,89
developing brain homeobox 1	DBX1	0,89
RANBP2-like and GRIP domain containing 5; RANBP2-like and GRIP domain containing 8	RGPD5; RGPD8	0,89
olfactory receptor, family 4, subfamily K, member 14	OR4K14	0,89
integrin alpha FG-GAP repeat containing 2	ITFG2	0,89
chromosome X open reading frame 38	CXorf38	0,89
zinc finger protein 493	ZNF493	0,89
fucosidase, alpha-L- 2, plasma	FUCA2	0,89
NLR family, CARD domain containing 4	NLRC4	0,89
Memczak2013 ALT_ACCEPTOR, ALT_DONOR, coding, INTERNAL, intronic best transcript NM_001145114	RRP12	0,89
pro-melanin-concentrating hormone	PMCH	0,89
ribosomal protein S17	RPS17	0,89
major facilitator superfamily domain containing 14C	MFSD14C	0,89
heterogeneous nuclear ribonucleoprotein H1 (H)	HNRNPH1	0,89
myotubularin related protein 10	MTMR10	0,89
outer dense fiber of sperm tails 3	ODF3	0,89
frizzled class receptor 6	FZD6	0,89
chromosome 1 open reading frame 101	C1orf101	0,89
chloride channel, voltage-sensitive Kb	CLCNKB	0,89
chromosome 10 open reading frame 53	C10orf53	0,89
Jeck2013 ANTISENSE, CDS, coding, INTERNAL, intronic, OVCODE, OVEXON best transcript NM_002473	MYH9	0,89
biogenesis of lysosomal organelles complex-1, subunit 1	BLOC1S1	0,89
N-acetylgalactosaminidase, alpha-	NAGA	0,89
CKLF-like MARVEL transmembrane domain containing 6	CMTM6	0,89
zinc and ring finger 3	ZNRF3	0,89
dual specificity tyrosine-(Y)-phosphorylation regulated kinase 1B	DYRK1B	0,89
checkpoint kinase 2	CHEK2	0,89
chromodomain protein, Y-linked, 2A	CDY2A	0,89
vertebrae development associated	VRTN	0,89
paraspeckle component 1	PSPC1	0,89

proteasome (prosome, macropain) assembly chaperone 2	PSMG2	0,89
endogenous retrovirus group FRD, member 1	ERVFRD-1	0,89
tumor necrosis factor receptor superfamily, member 14	TNFRSF14	0,89
developmental pluripotency associated 2	DPPA2	0,89
ribosomal protein S19 binding protein 1	RPS19BP1	0,89
RAD9 checkpoint clamp component A	RAD9A	0,89
gasdermin B	GSDMB	0,89
peptidyl arginine deiminase, type IV	PADI4	0,89
potassium channel, voltage gated modifier subfamily G, member 2	KCNG2	0,89
solute carrier family 6 (neurotransmitter transporter), member 3	SLC6A3	0,89
oxidase (cytochrome c) assembly 1-like	OXA1L	0,89
long intergenic non-protein coding RNA 615	LINC00615	0,89
leucine rich repeat and Ig domain containing 2	LINGO2	0,89
asparaginase	ASPG	0,89
tetratricopeptide repeat domain 30A	TTC30A	0,89
family with sequence similarity 193, member B	FAM193B	0,89
transmembrane 9 superfamily protein member 4	TM9SF4	0,89
tumor necrosis factor receptor superfamily, member 17	TNFRSF17	0,89
secretoglobin, family 1C, member 2	SCGB1C2	0,89
D4, zinc and double PHD fingers family 1	DPF1	0,89
phosphoribosyl pyrophosphate synthetase 1	PRPS1	0,89
estrogen receptor 1	ESR1	0,89
RAS guanyl releasing protein 1 (calcium and DAG-regulated)	RASGRP1	0,89
U2 small nuclear RNA auxiliary factor 2	U2AF2	0,89
forkhead box E3	FOXE3	0,89
SH3 domain binding kinase 1	SBK1	0,89
neuregulin 3	NRG3	0,89
integrin alpha 9	ITGA9	0,89
MAX dimerization protein 3	MXD3	0,89
G protein-coupled receptor 155	GPR155	0,89
GAS8 antisense RNA 1	GAS8-AS1	0,89
serine peptidase inhibitor, Kazal type 13 (putative)	SPINK13	0,89
DnaJ (Hsp40) homolog, subfamily A, member 2	DNAJA2	0,89
proenkephalin	PENK	0,89
PDZ domain containing 7	PDZD7	0,89
branched chain amino-acid transaminase 1, cytosolic	BCAT1	0,89
mucin 17, cell surface associated	MUC17	0,89
zinc finger, C2HC-type containing 1B	ZC2HC1B	0,89
ribosomal protein S6 kinase, 90kDa, polypeptide 1	RPS6KA1	0,89
translocase of inner mitochondrial membrane 44 homolog (yeast)	TIMM44	0,89

aralkylamine N-acetyltransferase	AANAT	0,89
putative uncharacterized protein FLJ37770-like; Transcript Identified by AceView, Entrez Gene ID(s) 387790; novel transcript	LOC100506127; RP11-111M22.2; LOC387790	0,89
parkinson protein 7	PARK7	0,89
myopalladin	MYPN	0,89
MAGEA10-MAGEA5 readthrough; novel transcript	MAGEA10-MAGEA5; RP11-1007I13.4	0,89
myosin, heavy chain 7B, cardiac muscle, beta	MYH7B	0,89
polypyrimidine tract binding protein 1; microRNA 4745	PTBP1; MIR4745	0,89
family with sequence similarity 212, member A	FAM212A	0,89
thrombospondin 2	THBS2	0,89
solute carrier family 24 (sodium/potassium/calcium exchanger), member 1	SLC24A1	0,89
tumor necrosis factor (ligand) superfamily, member 4	TNFSF4	0,89
Memczak2013 ANTISENSE, CDS, coding, INTERNAL best transcript NM_002957	RXRA	0,89
hydroxymethylbilane synthase	HMBS	0,89
heat shock protein 90kDa alpha (cytosolic), class B member 1	HSP90AB1	0,89
keratin 84, type II	KRT84	0,89
rhomboid, veinlet-like 1 (Drosophila)	RHBDL1	0,89
potassium channel, voltage gated shaker related subfamily A, member 6; polypeptide N-acetylgalactosaminyltransferase 8	KCNA6; GALNT8	0,89
HMP19 protein; Neuron-specific protein family member 2 [Source:UniProtKB/Swiss-Prot;Acc:Q9Y328]; Transcript Identified by AceView, Entrez Gene ID(s) 51617	HMP19	0,89
HECT and RLD domain containing E3 ubiquitin protein ligase 3	HERC3	0,89
SERTA domain containing 4	SERTAD4	0,89
neurotensin receptor 2	NTSR2	0,89
mitochondrial poly(A) polymerase	MTPAP	0,89
thrombospondin type 1 domain containing 1	THSD1	0,89
transmembrane protein 95	TMEM95	0,89
Kruppel-like factor 8	KLF8	0,89
testis specific protein, Y-linked 1	TSPY1	0,89
tudor domain containing 1	TDRD1	0,89
patatin-like phospholipase domain containing 7	PNPLA7	0,89
IKAROS family zinc finger 2	IKZF2	0,89
chromosome 5 open reading frame 56	C5orf56	0,89
UDP-Gal:betaGlcNAc beta 1,3-galactosyltransferase 1	B3GALT1	0,89
TXK tyrosine kinase	TXK	0,89
arginine vasopressin receptor 2	AVPR2	0,89

protein tyrosine phosphatase, non-receptor type 5 (striatum-enriched)	PTPN5	0,89
transmembrane protein 205	TMEM205	0,89
glutamate rich 6	ERICH6	0,89
protocadherin 9	PCDH9	0,89
SLIT and NTRK-like family, member 6	SLITRK6	0,89
baculoviral IAP repeat containing 2	BIRC2	0,89
integrin beta 1 binding protein (melusin) 2	ITGB1BP2	0,89
PSMB8 antisense RNA 1 (head to head)	PSMB8-AS1	0,89
apolipoprotein B mRNA editing enzyme, catalytic polypeptide-like 3A	APOBEC3A	0,89
Memczak2013 ALT_ACCEPTOR, ALT_DONOR, coding, INTERNAL, intronic best transcript NM_002865	RAB2A	0,89
serine/arginine repetitive matrix 3	SRRM3	0,89
olfactory receptor, family 5, subfamily H, member 1	OR5H1	0,89
olfactory receptor, family 1, subfamily J, member 1	OR1J1	0,89
chromatin assembly factor 1, subunit A (p150)	CHAF1A	0,89
regulator of G-protein signaling 7 binding protein	RGS7BP	0,89
CLOCK-interacting pacemaker	CIPC	0,89
chromosome 2 open reading frame 80	C2orf80	0,89
noggin	NOG	0,89
mitochondrial ribosomal protein L44	MRPL44	0,89
heterogeneous nuclear ribonucleoprotein U (scaffold attachment factor A)	HNRNPU	0,89
tubby like protein 4	TULP4	0,89
zinc finger protein 385B	ZNF385B	0,89
filamin A interacting protein 1	FILIP1	0,89
K(lysine) acetyltransferase 6B	KAT6B	0,89
ubiquitin conjugating enzyme E2K	UBE2K	0,89
intraflagellar transport 57	IFT57	0,89
death inducer-obliterator 1	DIDO1	0,89
poly(rC) binding protein 2; PCBP2 overlapping transcript 1	PCBP2; PCBP2-OT1	0,89
Memczak2013 ALT_ACCEPTOR, ALT_DONOR, coding, INTERNAL, intronic best transcript NM_001190821	SMAD7	0,89
G protein-coupled receptor 171	GPR171	0,89
C-type lectin domain family 4, member G	CLEC4G	0,89
arrestin domain containing 1	ARRDC1	0,89
trimethylguanosine synthase 1	TGS1	0,89
family with sequence similarity 92, member A1	FAM92A1	0,89
cytochrome P450, family 2, subfamily A, polypeptide 6	CYP2A6	0,89
molybdenum cofactor synthesis 2	MOCS2	0,89
zinc finger protein 333	ZNF333	0,89
calmodulin-like 5	CALML5	0,89
pseudouridylate synthase 1	PUS1	0,89

dachshund family transcription factor 1	DACH1	0,89
hydroxyacyl-CoA dehydrogenase/3-ketoacyl-CoA thiolase/enoyl-CoA hydratase (trifunctional protein), beta subunit	HADHB	0,89
eukaryotic translation initiation factor 4E nuclear import factor 1	EIF4ENIF1	0,89
phospholipase C, delta 1	PLCD1	0,89
fibronectin type III and SPRY domain containing 1-like	FSD1L	0,89
PRAME family member 16; PRAME family member 17	PRAMEF16; PRAMEF17	0,89
CDC42 small effector 2	CDC42SE2	0,89
tripartite motif containing 67	TRIM67	0,89
l(3)mbt-like 1 (Drosophila)	L3MBTL1	0,89
zinc finger protein 683	ZNF683	0,89
neuroblastoma breakpoint family, member 11	NBPF11	0,89
cartilage associated protein	CRTAP	0,89
NLR family, pyrin domain containing 10	NLRP10	0,89
cell division cycle 20B	CDC20B	0,89
phosphomevalonate kinase	PMVK	0,89
leucine rich repeat containing 4	LRRC4	0,89
aquaporin 4	AQP4	0,89
fucose-1-phosphate guanylyltransferase	FPGT	0,89
growth factor receptor bound protein 14	GRB14	0,89
dynein, axonemal, assembly factor 3	DNAAF3	0,89
short chain dehydrogenase/reductase family 9C, member 7	SDR9C7	0,89
Na+/K+ transporting ATPase interacting 2	NKAIN2	0,89
Synthetic construct Homo sapiens clone IMAGE:100062553, MGC:190557 B melanoma antigen family, member 5 (BAGE5) mRNA, encodes complete protein.; B melanoma antigen family, member 5 [Source:EntrezGene;Acc:85316]	BAGE5	0,89
charged multivesicular body protein 2B	CHMP2B	0,89
olfactory receptor, family 3, subfamily A, member 3	OR3A3	0,89
zinc finger protein 250	ZNF250	0,89
transmembrane protein 132C	TMEM132C	0,89
Transcript Identified by AceView, Entrez Gene ID(s) 5747	PTK2	0,89
solute carrier family 6 (neurotransmitter transporter), member 5	SLC6A5	0,89
chromosome 16 open reading frame 91	C16orf91	0,89
keratin associated protein 10-4	KRTAP10-4	0,89
integrin beta 6	ITGB6	0,89
smu-1 suppressor of mec-8 and unc-52 homolog (C. elegans)	SMU1	0,89

natriuretic peptide B	NPPB	0,89
chromosome 10 open reading frame 120	C10orf120	0,89
translocase of inner mitochondrial membrane 9 homolog (yeast)	TIMM9	0,89
glutamine rich 2	QRICH2	0,89
protein kinase (cAMP-dependent, catalytic) inhibitor alpha	PKIA	0,89
myoregulin	MRLN	0,89
Jeck2013 ALT_ACCEPTOR, ALT_DONOR, coding, INTERNAL, intronic best transcript NM_001160133	KCNQ5	0,89
kinase non-catalytic C-lobe domain (KIND) containing 1	KNDC1	0,89
tRNA nucleotidyl transferase, CCA-adding, 1	TRNT1	0,89
tumor necrosis factor receptor superfamily, member 25	TNFRSF25	0,89
FLT3-interacting zinc finger 1	FIZ1	0,89
ets variant 2	ETV2	0,89
PHD finger protein 19	PHF19	0,89
aminocarboxymuconate semialdehyde decarboxylase	ACMSD	0,89
titin	TTN	0,89
S1 RNA binding domain 1	SRBD1	0,89
cadherin 20, type 2	CDH20	0,89
TAF5-like RNA polymerase II, p300/CBP-associated factor (PCAF)-associated factor, 65kDa	TAF5L	0,89
RAD9-HUS1-RAD1 interacting nuclear orphan 1	RHNO1	0,89
epoxide hydrolase 3	EPHX3	0,89
olfactory receptor, family 10, subfamily A, member 7	OR10A7	0,89
ring finger protein 150	RNF150	0,89
GTP binding protein 6 (putative)	GTPBP6	0,89
leucine rich repeat containing 73	LRRC73	0,89
olfactory receptor, family 1, subfamily E, member 3 (gene/pseudogene)	OR1E3	0,89
Jeck2013 ALT_ACCEPTOR, ALT_DONOR, coding, INTERNAL, intronic best transcript NM_004787	SLIT2	0,89
neuralized E3 ubiquitin protein ligase 2	NEURL2	0,89
Memczak2013 ANTISENSE, coding, INTERNAL, intronic best transcript NM_006721	ADK	0,89
transforming growth factor, beta-induced, 68kDa	TGFBI	0,89
mitochondrial ribosomal protein S18C	MRPS18C	0,89
transition protein 1 (during histone to protamine replacement)	TNP1	0,89
synaptotagmin XIV	SYT14	0,89
chromosome 10 open reading frame 25	C10orf25	0,89
gem nuclear organelle associated protein 8	GEMIN8	0,89
transmembrane protein 257	TMEM257	0,89
transmembrane protein 145	TMEM145	0,89
protein arginine methyltransferase 9	PRMT9	0,89

Fc receptor, IgA, IgM, high affinity	FCAMR	0,89
zinc finger protein 532	ZNF532	0,88
	PRAMEF9	0,88
fibroblast growth factor 17	FGF17	0,88
keratin associated protein 8-1	KRTAP8-1	0,88
hornerin	HRNR	0,88
echinoderm microtubule associated protein like 3	EML3	0,88
lipoprotein lipase	LPL	0,88
tetratricopeptide repeat domain 23	TTC23	0,88
chemokine (C-C motif) ligand 14; chemokine (C-C motif) ligand 15; CCL15-CCL14 readthrough (NMD candidate)	CCL14; CCL15; CCL15-CCL14	0,88
actin-like 9	ACTL9	0,88
coiled-coil domain containing 87	CCDC87	0,88
HTR5A antisense RNA 1	HTR5A-AS1	0,88
dyslexia susceptibility 1 candidate 1	DYX1C1	0,88
forkhead box D4-like 3	FOXD4L3	0,88
Zhang2013 ALT_ACCEPTOR, ALT_DONOR, coding, INTERNAL, intronic best transcript NM_001145297	EXOC7	0,88
methionine sulfoxide reductase B1	MSRB1	0,88
potassium channel, inwardly rectifying subfamily J, member 6	KCNJ6	0,88
Transcript Identified by AceView, Entrez Gene ID(s) 7881	KCNAB1	0,88
heat shock transcription factor, Y-linked 2; heat shock transcription factor, Y-linked 1	HSFY2; HSFY1	0,88
guanylyl cyclase domain containing 1	GUCD1	0,88
FRY like transcription coactivator	FRYL	0,88
neurexophilin 1	NXPH1	0,88
NHP2 ribonucleoprotein	NHP2	0,88
pancreas specific transcription factor, 1a	PTF1A	0,88
olfactory receptor, family 13, subfamily G, member 1	OR13G1	0,88
nucleosome assembly protein 1-like 4	NAP1L4	0,88
topoisomerase (DNA) III alpha	TOP3A	0,88
MAS1 proto-oncogene like, G protein-coupled receptor	MAS1L	0,88
B-cell CLL/lymphoma 11B (zinc finger protein)	BCL11B	0,88
phosphodiesterase 4C, cAMP-specific	PDE4C	0,88
Memczak2013 ALT_DONOR, coding, INTERNAL, intronic best transcript NM_001421	ELF4	0,88
chromosome 16 open reading frame 78	C16orf78	0,88
alpha-1,4-N-acetylglucosaminyltransferase	A4GNT	0,88
POM121 transmembrane nucleoporin	POM121	0,88
ectonucleotide pyrophosphatase/phosphodiesterase 7	ENPP7	0,88
exostosin glycosyltransferase 1	EXT1	0,88
olfactory receptor, family 6, subfamily C, member 4	OR6C4	0,88
peptidyl arginine deiminase, type IV	PADI4	0,88

zona pellucida-like domain containing 1	ZPLD1	0,88
olfactory receptor, family 11, subfamily G, member 2	OR11G2	0,88
major histocompatibility complex, class I, L (pseudogene)	HLA-L	0,88
transducin-like enhancer of split 2	TLE2	0,88
Kruppel-like factor 15	KLF15	0,88
PDS5 cohesin associated factor A	PDS5A	0,88
sorting nexin family member 21	SNX21	0,88
Transcript Identified by AceView, Entrez Gene ID(s) 288	ANK3	0,88
leukocyte cell-derived chemotaxin 2	LECT2	0,88
interferon regulatory factor 2 binding protein 1	IRF2BP1	0,88
speedy/RINGO cell cycle regulator family member E2; speedy/RINGO cell cycle regulator family member E2B	SPDYE2; SPDYE2B	0,88
speedy/RINGO cell cycle regulator family member E2B	SPDYE2B	0,88
Jrk helix-turn-helix protein	JRK	0,88
ubiquitin specific peptidase 21	USP21	0,88
insulinoma-associated 2	INSM2	0,88
dopamine receptor D5	DRD5	0,88
transient receptor potential cation channel, subfamily M, member 2	TRPM2	0,88
secretoglobin, family 2B, member 2	SCGB2B2	0,88
T cell-interacting, activating receptor on myeloid cells 1	TARM1	0,88
phenylalanine hydroxylase	PAH	0,88
syntaxin 1B	STX1B	0,88
ubiquitin specific peptidase 43	USP43	0,88
FLYWCH family member 2	FLYWCH2	0,88
ankyrin repeat domain 34A	ANKRD34A	0,88
Src homology 2 domain containing E	SHE	0,88
lysine (K)-specific demethylase 4D	KDM4D	0,88
chromodomain protein, Y-linked, 1	CDY1	0,88
nucleosome assembly protein 1-like 1	NAP1L1	0,88
choline O-acetyltransferase	CHAT	0,88
PDZ domain containing 2	PDZD2	0,88
tumor suppressor candidate 1	TUSC1	0,88
gamma-glutamyltransferase 7	GGT7	0,88
ISG15 ubiquitin-like modifier	ISG15	0,88
meiotic nuclear divisions 1	MND1	0,88
naked cuticle homolog 2 (Drosophila)	NKD2	0,88
pre-mRNA processing factor 38A	PRPF38A	0,88
tripartite motif containing 9	TRIM9	0,88
sentan, cilia apical structure protein	SNTN	0,88
ripply transcriptional repressor 2	RIPPLY2	0,88
serine peptidase inhibitor, Kazal type 5	SPINK5	0,88
mitochondrial ribosomal protein L3	MRPL3	0,88
transmembrane 9 superfamily member 3	TM9SF3	0,88

interleukin enhancer binding factor 2	ILF2	0,88
HscB mitochondrial iron-sulfur cluster co-chaperone	HSCB	0,88
phosphatidylinositol-specific phospholipase C, X domain containing 3	PLCXD3	0,88
TROVE domain family, member 2	TROVE2	0,88
sterile alpha motif domain containing 7	SAMD7	0,88
excision repair cross-complementation group 3	ERCC3	0,88
cyclic nucleotide binding domain containing 1	CNBD1	0,88
Memczak2013 ANTISENSE, CDS, coding, INTERNAL best transcript NM_153280; Transcript Identified by AceView	UBA1; tydee	0,88
solute carrier family 22 (organic anion transporter), member 8	SLC22A8	0,88
collagen, type XVI, alpha 1	COL16A1	0,88
chromosome 9 open reading frame 57	C9orf57	0,88
phospholipase D family, member 6	PLD6	0,88
poly(A) binding protein, cytoplasmic 5	PABPC5	0,88
phosphoglycerate kinase 2	PGK2	0,88
TP53 target 3B	TP53TG3B	0,88
Transcript Identified by AceView, Entrez Gene ID(s) 138050	HGSNAT	0,88
SWI/SNF related, matrix associated, actin dependent regulator of chromatin, subfamily d, member 1	SMARCD1	0,88
upstream binding transcription factor, RNA polymerase I	UBTF	0,88
Zhang2013 ALT_ACCEPTOR, ALT_DONOR, coding, INTERNAL, intronic best transcript NM_025176	NINL	0,88
ribosomal protein L4	RPL4	0,88
chaperonin containing TCP1, subunit 5 (epsilon)	CCT5	0,88
kinase insert domain receptor	KDR	0,88
ARFGEF family member 3	ARFGEF3	0,88
tachykinin 4 (hemokinin)	TAC4	0,88
forkhead box P3	FOXP3	0,88
plexin A4	PLXNA4	0,88
Memczak2013 ALT_ACCEPTOR, ALT_DONOR, coding, INTERNAL, intronic best transcript NM_006454	MXD4	0,88
ankyrin repeat domain 30A	ANKRD30A	0,88
RNA 2,3-cyclic phosphate and 5-OH ligase	RTCB	0,88
glutathione S-transferase mu 5	GSTM5	0,88
junctional sarcoplasmic reticulum protein 1	JSRP1	0,88
butyrylcholinesterase	BCHE	0,88
SSU72 homolog, RNA polymerase II CTD phosphatase	SSU72	0,88
taste receptor, type 2, member 13	TAS2R13	0,88
methyl-CpG binding domain protein 3-like 4	MBD3L4	0,88
BRX1, biogenesis of ribosomes	BRIX1	0,88
adhesion G protein-coupled receptor G4	ADGRG4	0,88

MYB binding protein (P160) 1a	MYBBP1A	0,88
Transcript Identified by AceView, Entrez Gene ID(s) 6777	STAT5B	0,88
Rho GTPase activating protein 11B	ARHGAP11B	0,88
family with sequence similarity 161, member A	FAM161A	0,88
aspartoacylase	ASPA	0,88
CPX chromosome region, candidate 1	CPXCR1	0,88
G protein-coupled receptor 26	GPR26	0,88
mesoderm induction early response 1, family member 2	MIER2	0,88
family with sequence similarity 180, member B	FAM180B	0,88
cryptochrome circadian clock 2	CRY2	0,88
chromosome 19 open reading frame 60	C19orf60	0,88
SET and MYND domain containing 2	SMYD2	0,88
adhesion G protein-coupled receptor L4	ADGRL4	0,88
makorin ring finger protein 3	MKRN3	0,88
solute carrier family 26 (anion exchanger), member 6; microRNA 6824	SLC26A6; MIR6824	0,88
CTP synthase 2	CTPS2	0,88
superoxide dismutase 2, mitochondrial	SOD2	0,88
leucine rich repeat and Ig domain containing 3	LINGO3	0,88
growth hormone 1	GH1	0,88
olfactory receptor, family 5, subfamily F, member 1	OR5F1	0,88
islet cell autoantigen 1	ICA1	0,88
phospholipase A2, group IIE	PLA2G2E	0,88
SPATA31 subfamily A, member 6	SPATA31A6	0,88
kelch-like family member 33	KLHL33	0,88
zinc finger protein 48	ZNF48	0,88
K(lysine) acetyltransferase 6B	KAT6B	0,88
phospholipase A2, group XIIA	PLA2G12A	0,88
solute carrier family 7 (cationic amino acid transporter, y+ system), member 1	SLC7A1	0,88
kinesin family member 1A	KIF1A	0,88
methylmalonic aciduria (cobalamin deficiency) cblA type	MMAA	0,88
SLIT and NTRK-like family, member 4	SLITRK4	0,88
recombination activating gene 2	RAG2	0,88
Homo sapiens testis specific protein, Y-linked 3, mRNA (cDNA clone MGC:103998 IMAGE:30915406), complete cds.; Homo sapiens similar to testis specific protein, Y-linked 1, mRNA (cDNA clone MGC:150413 IMAGE:40120433), complete cds.; Homo sapiens testis specific protein, Y-linked 3, mRNA (cDNA clone MGC:150414 IMAGE:40120435), complete cds.	TSPY3; LOC728395	0,88
SET and MYND domain containing 3	SMYD3	0,88
transcription elongation factor, mitochondrial	TEFM	0,88

CDC like kinase 2	CLK2	0,88
nei-like DNA glycosylase 1	NEIL1	0,88
olfactory receptor, family 1, subfamily N, member 1	OR1N1	0,88
kinesin family member 15	KIF15	0,88
coiled-coil domain containing 12	CCDC12	0,88
Sjogren syndrome antigen B (autoantigen La)	SSB	0,88
chromosome 11 open reading frame 94	C11orf94	0,88
cylicin, basic protein of sperm head cytoskeleton 2	CYLC2	0,88
long intergenic non-protein coding RNA 1219	LINC01219	0,88
olfactory receptor, family 6, subfamily C, member 70	OR6C70	0,88
phosphatidylinositol glycan anchor biosynthesis class K	PIGK	0,88
uncharacterized LOC100505478; putative novel transcript	LOC100505478; RP11-402G3.3	0,88
olfactory receptor, family 7, subfamily D, member 4	OR7D4	0,88
adaptor-related protein complex 3, mu 2 subunit	AP3M2	0,88
shisa family member 6	SHISA6	0,88
MAM domain containing glycosylphosphatidylinositol anchor 1	MDGA1	0,88
Down syndrome critical region 4	DSCR4	0,88
meiosis 1 associated protein	M1AP	0,88
proline-rich transmembrane protein 3	PRRT3	0,88
claudin 10	CLDN10	0,88
testis specific 10 interacting protein	TSGA10IP	0,88
ALG1, chitobiosyldiphosphodolichol beta-mannosyltransferase-like	ALG1L	0,88
insulin receptor-related receptor	INSRR	0,88
cytochrome c oxidase subunit Vb	COX5B	0,88
ribonuclease, RNase A family, 13 (non-active)	RNASE13	0,88
small nuclear ribonucleoprotein, U11/U12 25kDa subunit	SNRNP25	0,88
fibulin 7	FBLN7	0,88
nucleolar protein 4-like	NOL4L	0,88
Memczak2013 ALT_ACCEPTOR, ALT_DONOR, coding, INTERNAL, intronic best transcript NM_173683; novel transcript	AF131215.3; XKR6	0,88
zinc finger protein 628	ZNF628	0,88
ring finger and CCCH-type domains 1	RC3H1	0,88
glial cells missing homolog 1 (Drosophila)	GCM1	0,88
peptidylprolyl isomerase A (cyclophilin A)-like 4G	PPIAL4G	0,88
testis highly expressed protein 5; novel transcript; Transcript Identified by AceView	THEG5; AC011525.4; pleysey	0,88
paired related homeobox 1	PRRX1	0,88
chloride channel, voltage-sensitive Ka	CLCNKA	0,88
TGF-beta activated kinase 1/MAP3K7 binding protein 3	TAB3	0,88

fibrosin-like 1	FBRSL1	0,88
olfactory receptor, family 6, subfamily N, member 2	OR6N2	0,88
ATPase, H+ transporting, lysosomal accessory protein 2	ATP6AP2	0,88
MT-RNR2-like 10	MTRNR2L10	0,88
tripartite motif containing 45	TRIM45	0,88
submaxillary gland androgen regulated protein 3B	SMR3B	0,88
nyctalopin	NYX	0,88
centrosomal protein 295kDa	CEP295	0,88
forkhead box L1-like; novel transcript; Transcript Identified by AceView	WI2-2373I1.2; pajoyby	0,88
R3H domain containing 2	R3HDM2	0,88
Transcript Identified by AceView, Entrez Gene ID(s) 5825	ABCD3	0,88
neuroligin 3	NLGN3	0,88
URB1 ribosome biogenesis 1 homolog (S. cerevisiae)	URB1	0,88
regulatory factor X-associated ankyrin-containing protein	RFXANK	0,88
ferritin mitochondrial	FTMT	0,88
platelet derived growth factor D	PDGFD	0,88
epithelial mitogen	EPGN	0,88
prostaglandin-endoperoxide synthase 2 (prostaglandin G/H synthase and cyclooxygenase)	PTGS2	0,88
transmembrane protein 14A	TMEM14A	0,88
MAGE family member A12; CSAG family, member 4 (pseudogene)	MAGEA12; CSAG4	0,88
hydrolethalus syndrome 1	HYLS1	0,88
succinate-CoA ligase, GDP-forming, beta subunit	SUCLG2	0,88
baculoviral IAP repeat containing 8	BIRC8	0,88
ret finger protein-like 1	RFPL1	0,88
phosphatidylinositol glycan anchor biosynthesis class L	PIGL	0,88
gamma-aminobutyric acid (GABA) A receptor, rho 2	GABRR2	0,88
hyaluronan-mediated motility receptor (RHAMM)	HMMR	0,88
S-antigen; retina and pineal gland (arrestin)	SAG	0,88
olfactory receptor, family 4, subfamily L, member 1	OR4L1	0,88
glycyl-tRNA synthetase	GARS	0,88
family with sequence similarity 71, member A	FAM71A	0,88
F-box protein 2	FBXO2	0,88
DIS3 homolog, exosome endoribonuclease and 3-5 exoribonuclease	DIS3	0,88
MAGE family member B1; MAGE family member B4	MAGEB1; MAGEB4	0,88
KRIT1, ankyrin repeat containing	KRIT1	0,88
HIG1 hypoxia inducible domain family, member 1C	HIGD1C	0,88
scinderin	SCIN	0,88
olfactory receptor, family 5, subfamily L, member 2	OR5L2	0,88

hypoxia up-regulated 1	HYOU1	0,88
CD1e molecule	CD1E	0,88
Transcript Identified by AceView, Entrez Gene ID(s) 23122	CLASP2	0,88
CD248 molecule, endosialin	CD248	0,88
homeobox A6	HOXA6	0,88
family with sequence similarity 69, member C	FAM69C	0,88
ankyrin 1, erythrocytic	ANK1	0,88
Fc fragment of IgE, high affinity I, receptor for; alpha polypeptide	FCER1A	0,88
Transcript Identified by AceView, Entrez Gene ID(s) 93624	TADA2B	0,88
Jeck2013 ALT_ACCEPTOR, ALT_DONOR, coding, INTERNAL, intronic best transcript NM_015049	TRAK2	0,88
fat mass and obesity associated	FTO	0,88
Jeck2013 ALT_ACCEPTOR, ALT_DONOR, coding, INTERNAL, intronic best transcript NM_006042	HS3ST3A1	0,88
autocrine motility factor receptor, E3 ubiquitin protein ligase	AMFR	0,88
lymphocyte cytosolic protein 2	LCP2	0,88
adenosine deaminase, tRNA-specific 2	ADAT2	0,88
long intergenic non-protein coding RNA 1342	LINC01342	0,88
cell division cycle 6	CDC6	0,88
ring finger and SPRY domain containing 1	RSPRY1	0,88
leucine zipper, putative tumor suppressor 1	LZTS1	0,88
olfactory receptor, family 10, subfamily H, member 5	OR10H5	0,88
C9orf135 antisense RNA 1 (head to head)	C9orf135-AS1	0,88
zinc finger protein 862	ZNF862	0,88
collagen, type VIII, alpha 2	COL8A2	0,88
arginine-glutamic acid dipeptide (RE) repeats	RERE	0,88
Jeck2013 ALT_DONOR, coding, INTERNAL, intronic best transcript NM_004515	ILF2	0,88
increased sodium tolerance 1 homolog (yeast)	IST1	0,88
deafness, autosomal recessive 31	DFNB31	0,88
olfactory receptor, family 56, subfamily A, member 3	OR56A3	0,88
ectonucleoside triphosphate diphosphohydrolase 4; lysyl oxidase-like 2	ENTPD4; LOXL2	0,88
homeobox B6	HOXB6	0,88
DEAD (Asp-Glu-Ala-Asp) box polypeptide 60-like	DDX60L	0,88
elastase, neutrophil expressed	ELANE	0,88
NADPH oxidase 4	NOX4	0,88
SH3 and multiple ankyrin repeat domains 1	SHANK1	0,88
cytochrome P450, family 4, subfamily F, polypeptide 22	CYP4F22	0,88
WDYHV motif containing 1	WDYHV1	0,88

spastic paraplegia 7 (pure and complicated autosomal recessive)	SPG7	0,88
erythrocyte membrane protein band 4.2	EPB42	0,88
fucosyltransferase 10 (alpha (1,3) fucosyltransferase)	FUT10	0,88
glutathione peroxidase 6	GPX6	0,88
Transcript Identified by AceView, Entrez Gene ID(s) 108	ADCY2	0,88
peptidyl arginine deiminase, type I	PADI1	0,88
5-azacytidine induced 2	AZI2	0,88
contactin associated protein-like 5	CNTNAP5	0,88
hedgehog acyltransferase	HHAT	0,88
podocalyxin-like 2	PODXL2	0,88
abhydrolase domain containing 17C	ABHD17C	0,88
nucleoredoxin-like 2	NXNL2	0,88
AT rich interactive domain 1B (SWI1-like)	ARID1B	0,88
potassium channel, voltage gated modifier subfamily V, member 2	KCNV2	0,88
fibroblast growth factor 7	FGF7	0,88
sodium channel, voltage gated, type V alpha subunit	SCN5A	0,88
polymerase (DNA directed), delta 2, accessory subunit	POLD2	0,88
chromosome 9 open reading frame 172	C9orf172	0,88
myeloid-derived growth factor	MYDGF	0,88
apoptosis-associated tyrosine kinase	AATK	0,88
olfactory receptor, family 51, subfamily B, member 4	OR51B4	0,88
serum response factor	SRF	0,88
regulator of G protein signaling 9 binding protein	RGS9BP	0,88
neurofascin	NFASC	0,88
golgin A1	GOLGA1	0,88
stromal interaction molecule 2	STIM2	0,88
novel transcript; olfactory receptor, family 5, subfamily H, member 8 (gene/pseudogene) [Source:HGNC Symbol;Acc:HGNC:14773]	RP11-325B23.2; OR5H8	0,88
p53-induced death domain protein 1	PIDD1	0,87
microRNA 6809; tensin 1	MIR6809; TNS1	0,87
DDB1 and CUL4 associated factor 16	DCAF16	0,87
olfactory receptor, family 5, subfamily B, member 2	OR5B2	0,87
Rho guanine nucleotide exchange factor 1	ARHGEF1	0,87
2-aminoethanethiol (cysteamine) dioxygenase	ADO	0,87
gap junction protein delta 4	GJD4	0,87
siah E3 ubiquitin protein ligase family member 3	SIAH3	0,87
novel transcript; Transcript Identified by AceView, Entrez Gene ID(s) 8559; RP11-295P9.3 (from geneSymbol)	RP11-295P9.3; PRPF18	0,87
fatty acid binding protein 7, brain	FABP7	0,87
structural maintenance of chromosomes 6	SMC6	0,87
BTB (POZ) domain containing 7	BTBD7	0,87

nucleoporin like 2	NUPL2	0,87
ADAM metallopeptidase domain 20	ADAM20	0,87
zinc finger protein 593	ZNF593	0,87
TP53 target 3B	TP53TG3B	0,87
Homo sapiens TP53 target 3B (TP53TG3B), transcript variant 1, mRNA.; Homo sapiens TP53 target 3C (TP53TG3C), transcript variant 1, mRNA.; Homo sapiens TP53 target 3 (TP53TG3), transcript variant 1, mRNA.; Homo sapiens TP53 target 3 (TP53TG3), transcript variant 3, non-coding RNA.; Homo sapiens TP53 target 3B (TP53TG3B), transcript variant 3, non-coding RNA.; Homo sapiens TP53 target 3 (TP53TG3), transcript variant 4, non-coding RNA.; Homo sapiens TP53 target 3C (TP53TG3C), transcript variant 3, non-coding RNA.; TP53-target gene 3 protein; Homo sapiens TP53 target 3, mRNA (cDNA clone MGC:119889 IMAGE:40015196), complete cds.; Homo sapiens TP53 target 3, mRNA (cDNA clone MGC:119888 IMAGE:40015195), complete cds.; TP53-target gene 3 protein [Source:UniProtKB/Swiss-Prot;Acc:Q9ULZ0]	TP53TG3B; TP53TG3C; TP53TG3; LOC102723655; RP11-23E10.6	0,87
Homo sapiens TP53 target 3B (TP53TG3B), transcript variant 1, mRNA.; Homo sapiens TP53 target 3C (TP53TG3C), transcript variant 1, mRNA.; Homo sapiens TP53 target 3 (TP53TG3), transcript variant 1, mRNA.; Homo sapiens TP53 target 3 (TP53TG3), transcript variant 3, non-coding RNA.; Homo sapiens TP53 target 3B (TP53TG3B), transcript variant 3, non-coding RNA.; Homo sapiens TP53 target 3 (TP53TG3), transcript variant 4, non-coding RNA.; Homo sapiens TP53 target 3C (TP53TG3C), transcript variant 3, non-coding RNA.; TP53-target gene 3 protein; Homo sapiens TP53 target 3, mRNA (cDNA clone MGC:119889 IMAGE:40015196), complete cds.; Homo sapiens TP53 target 3, mRNA (cDNA clone MGC:119888 IMAGE:40015195), complete cds.; Transcript Identified by AceView; TP53-target gene 3 protein [Source:UniProtKB/Swiss-Prot;Acc:Q9ULZ0]	TP53TG3B; TP53TG3C; TP53TG3; LOC102723655; hotema; RP11-1277H1.5	0,87
acyl-CoA synthetase medium-chain family member 4	ACSM4	0,87
heterogeneous nuclear ribonucleoprotein C-like 3; heterogeneous nuclear ribonucleoprotein C-like 4	HNRNPCL3; HNRNPCL4	0,87
zinc finger protein 586	ZNF586	0,87
G protein-coupled receptor 101	GPR101	0,87
Zhang2013 ALT_DONOR, coding, INTERNAL, intronic best transcript NM_007184	NISCH	0,87
WD repeat domain 35	WDR35	0,87
tau tubulin kinase 2	TTBK2	0,87

transcription elongation factor A (SII) N-terminal and central domain containing	TCEANC	0,87
solute carrier family 6, member 16	SLC6A16	0,87
zinc finger protein 573	ZNF573	0,87
platelet derived growth factor C	PDGFC	0,87
ficolin (collagen/fibrinogen domain containing) 1	FCN1	0,87
zinc finger protein 764	ZNF764	0,87
neuropeptide Y	NPY	0,87
solute carrier family 10 (sodium/bile acid cotransporter), member 6	SLC10A6	0,87
olfactory receptor, family 5, subfamily D, member 16	OR5D16	0,87
solute carrier family 15 (oligopeptide transporter), member 2	SLC15A2	0,87
palmdelphin	PALMD	0,87
BCL2-like 14 (apoptosis facilitator)	BCL2L14	0,87
Wiskott-Aldrich syndrome	WAS	0,87
StAR-related lipid transfer domain containing 9	STARD9	0,87
family with sequence similarity 174, member B	FAM174B	0,87
retinoid X receptor gamma	RXRG	0,87
forkhead box N1	FOXN1	0,87
spermidine synthase	SRM	0,87
TBC1 domain family, member 3 pseudogene 5	TBC1D3P5	0,87
MAN1B1 antisense RNA 1 (head to head)	MAN1B1-AS1	0,87
pleckstrin homology domain containing, family A member 5	PLEKHA5	0,87
ATP synthase, H+ transporting, mitochondrial F1 complex, O subunit	ATP5O	0,87
tetratricopeptide repeat domain 23-like	TTC23L	0,87
progestin and adipoQ receptor family member IV	PAQR4	0,87
MAGI family member, X-linked	MAGIX	0,87
transglutaminase 4	TGM4	0,87
proteasome subunit alpha 8	PSMA8	0,87
coiled-coil domain containing 148	CCDC148	0,87
cell wall biogenesis 43 C-terminal homolog	CWH43	0,87
sidekick cell adhesion molecule 1	SDK1	0,87
amylase, alpha 2A (pancreatic)	AMY2A	0,87
nuclear protein, ataxia-telangiectasia locus	NPAT	0,87
SPATA31 subfamily A, member 3	SPATA31A3	0,87
general transcription factor IIH subunit 4	GTF2H4	0,87
kinesin family member 15	KIF15	0,87
keratin associated protein 10-3	KRTAP10-3	0,87
Transcript Identified by AceView, Entrez Gene ID(s) 4345; novel transcript	CD200; RP11-90K6.1	0,87
NCK adaptor protein 1	NCK1	0,87
perilipin 2	PLIN2	0,87

serine-rich and transmembrane domain containing 1	SERTM1	0,87
discs, large (Drosophila) homolog-associated protein 1	DLGAP1	0,87
pregnancy up-regulated nonubiquitous CaM kinase	PNCK	0,87
derlin 3	DERL3	0,87
SIGLEC family like 1	SIGLECL1	0,87
complement component 1, q subcomponent-like 3	C1QL3	0,87
xylosyltransferase II	XYLT2	0,87
RPL17-C18orf32 readthrough	RPL17-C18orf32	0,87
glutamyl-prolyl-tRNA synthetase	EPRS	0,87
chromosome 1 open reading frame 43	C1orf43	0,87
dihydropyrimidinase	DPYS	0,87
Zhang2013 ALT_ACCEPTOR, ALT_DONOR, coding, INTERNAL, intronic best transcript NM_002520	NPM1	0,87
chromosome 6 open reading frame 62	C6orf62	0,87
clusterin; microRNA 6843	CLU; MIR6843	0,87
adhesion G protein-coupled receptor F5	ADGRF5	0,87
ribosomal protein L39-like	RPL39L	0,87
proline rich 23C	PRR23C	0,87
serum amyloid A1	SAA1	0,87
TatD DNase domain containing 2; ghrelin opposite strand/antisense RNA	TATDN2; GHRLOS	0,87
chondroitin sulfate synthase 3	CHSY3	0,87
tigger transposable element derived 2	TIGD2	0,87
5-nucleotidase, cytosolic IB; NT5C1B-RDH14 readthrough; retinol dehydrogenase 14 (all-trans/9-cis/11-cis)	NT5C1B; NT5C1B-RDH14; RDH14	0,87
cleavage stimulation factor, 3 pre-RNA, subunit 1	CSTF1	0,87
diacylglycerol kinase theta	DGKQ	0,87
Zhang2013 ALT_ACCEPTOR, ALT_DONOR, coding, INTERNAL, intronic best transcript NM_003934	FUBP3	0,87
eukaryotic translation initiation factor 4 gamma, 2; small nucleolar RNA, C/D box 97	EIF4G2; SNORD97	0,87
transmembrane protein 132B	TMEM132B	0,87
Transcript Identified by AceView, Entrez Gene ID(s) 10915	TCERG1	0,87
eukaryotic translation initiation factor 2-alpha kinase 1	EIF2AK1	0,87
epididymal protein 3A	EDDM3A	0,87
inositol polyphosphate-5-phosphatase D	INPP5D	0,87
basic transcription factor 3	BTF3	0,87
dual specificity tyrosine-(Y)-phosphorylation regulated kinase 1B	DYRK1B	0,87
collectin sub-family member 10 (C-type lectin)	COLEC10	0,87
fragile site, folic acid type, rare, fra(10)(q23.3) or fra(10)(q24.2) candidate 1	FRA10AC1	0,87
lysyl oxidase-like 3	LOXL3	0,87

MLX interacting protein	MLXIP	0,87
myosin, heavy chain 2, skeletal muscle, adult	MYH2	0,87
sperm associated antigen 6	SPAG6	0,87
glucagon receptor	GCGR	0,87
zinc finger protein 292	ZNF292	0,87
L-3-hydroxyproline dehydratase (trans-)	L3HYPDH	0,87
pleckstrin homology domain containing, family A (phosphoinositide binding specific) member 1	PLEKHA1	0,87
SMAD family member 4	SMAD4	0,87
phosphopantothenoylcysteine synthetase; coiled-coil domain containing 30	PPCS; CCDC30	0,87
S-antigen; retina and pineal gland (arrestin)	SAG	0,87
lin-52 DREAM MuvB core complex component	LIN52	0,87
N(alpha)-acetyltransferase 10, NatA catalytic subunit	NAA10	0,87
uncoupling protein 1 (mitochondrial, proton carrier)	UCP1	0,87
ADP-ribosylation factor guanine nucleotide-exchange factor 2 (brefeldin A-inhibited)	ARFGEF2	0,87
zinc finger CCCH-type containing 8	ZC3H8	0,87
olfactory receptor, family 5, subfamily A, member 2	OR5A2	0,87
protease, serine 45	PRSS45	0,87
proline-serine-threonine phosphatase interacting protein 1	PSTPIP1	0,87
chromosome 1 open reading frame 112	C1orf112	0,87
cell adhesion molecule 3	CADM3	0,87
early B-cell factor 4	EBF4	0,87
lysine (K)-specific demethylase 2B	KDM2B	0,87
glutathione S-transferase mu 2 (muscle)	GSTM2	0,87
killer cell immunoglobulin-like receptor, two domains, short cytoplasmic tail, 5; killer cell immunoglobulin-like receptor, three domains, long cytoplasmic tail, 2; killer cell immunoglobulin-like receptor, two domains, long cytoplasmic tail, 1; killer cell immunoglobulin-like receptor, three domains, long cytoplasmic tail, 3; killer cell immunoglobulin-like receptor, three domains, long cytoplasmic tail, 1; killer cell immunoglobulin-like receptor, two domains, short cytoplasmic tail, 4; killer cell immunoglobulin-like receptor, two domains, long cytoplasmic tail, 3; killer cell immunoglobulin-like receptor, three domains, short cytoplasmic tail, 1; killer cell immunoglobulin-like receptor, two domains, short cytoplasmic tail, 2; killer cell immunoglobulin-like receptor, two domains, pseudogene 1; killer cell immunoglobulin-like receptor, three domains, pseudogene 1	KIR2DS5; KIR3DL2; KIR2DL1; KIR3DL3; KIR3DL1; KIR2DS4; KIR2DL3; KIR3DS1; KIR2DS2; KIR2DP1; KIR3DP1	0,87
olfactomedin 3	OLFM3	0,87

long intergenic non-protein coding RNA 550	LINC00550	0,87
fibroblast growth factor (acidic) intracellular binding protein	FIBP	0,87
CASK interacting protein 1	CASKIN1	0,87
cadherin 6, type 2, K-cadherin (fetal kidney)	CDH6	0,87
transporter 2, ATP-binding cassette, sub-family B (MDR/TAP)	TAP2	0,87
GM2 ganglioside activator	GM2A	0,87
Jeck2013 ALT_DONOR, coding, INTERNAL, intronic best transcript NM_032134	QRICH2	0,87
gem nuclear organelle associated protein 4	GEMIN4	0,87
guanylate cyclase 1, soluble, alpha 2	GUCY1A2	0,87
protein kinase C, delta binding protein	PRKCDBP	0,87
nudix hydrolase 13	NUDT13	0,87
dual specificity phosphatase 21	DUSP21	0,87
purinergic receptor P2Y, G-protein coupled, 10	P2RY10	0,87
B9 protein domain 2	B9D2	0,87
SON DNA binding protein; microRNA 6501	SON; MIR6501	0,87
decapping exoribonuclease	DXO	0,87
defensin, beta 104A; defensin, beta 104B	DEFB104A; DEFB104B	0,87
solute carrier family 11 (proton-coupled divalent metal ion transporter), member 1	SLC11A1	0,87
guanine nucleotide binding protein, alpha transducing 3	GNAT3	0,87
Jeck2013 ALT_ACCEPTOR, ALT_DONOR, coding, INTERNAL, intronic best transcript NM_005119	THRAP3	0,87
solute carrier family 25 (mitochondrial iron transporter), member 28	SLC25A28	0,87
bestrophin 2	BEST2	0,87
SR-related CTD-associated factor 8; T-cell lymphoma invasion and metastasis 2	SCAF8; TIAM2	0,87
SPANX family, member N4	SPANXN4	0,87
phosphatidylinositol 4-kinase, catalytic, alpha	PI4KA	0,87
potassium channel tetramerization domain containing 20	KCTD20	0,87
tubulin polymerization-promoting protein family member 3	TPPP3	0,87
necdin, MAGE family member	NDN	0,87
netrin 5	NTN5	0,87
KIRREL3 antisense RNA 3	KIRREL3-AS3	0,87
ADP-ribosylation factor like GTPase 6 interacting protein 1	ARL6IP1	0,87
olfactory receptor, family 51, subfamily A, member 4	OR51A4	0,87
novel transcript, sense overlapping TTC17; Transcript Identified by AceView, Entrez Gene ID(s) 55761	RP11-484D2.4; TTC17	0,87

solute carrier family 25, member 53	SLC25A53	0,87
ST8 alpha-N-acetyl-neuraminide alpha-2,8-sialyltransferase 3	ST8SIA3	0,87
intermediate filament family orphan 1	IFFO1	0,87
D site of albumin promoter (albumin D-box) binding protein	DBP	0,87
thymosin beta 15B	TMSB15B	0,87
M-phase phosphoprotein 9	MPHOSPH9	0,87
tubulin folding cofactor A	TBCA	0,87
protein serine kinase H2	PSKH2	0,87
zinc finger protein 74	ZNF74	0,87
solute carrier family 9, subfamily A (NHE4, cation proton antiporter 4), member 4	SLC9A4	0,87
FPGT-TNNI3K readthrough; fucose-1-phosphate guanylyltransferase	FPGT-TNNI3K; FPGT	0,87
transmembrane protein 225-like; Jeck2013 ALT_ACCEPTOR, ALT_DONOR, ncRNA, OVCODE, OVEXON, upstream_start best transcript TCONS_l2_00026125; Salzman2013 ANNOTATED, INTERNAL, ncRNA, OVCODE, OVEXON best transcript TCONS_l2_00026126; novel transcript; Transcript Identified by AceView, Entrez Gene ID(s) 79027	GS1-259H13.2; TCONS_l2_00026125; TCONS_l2_00026126; ZNF655	0,87
GTP binding protein 1	GTPBP1	0,87
Memczak2013 ANTISENSE, coding, INTERNAL, UTR3 best transcript NM_014737	RASSF2	0,87
bromodomain adjacent to zinc finger domain 2B	BAZ2B	0,87
transmembrane channel like 3	TMC3	0,87
villin-like	VILL	0,87
olfactory receptor, family 10, subfamily A, member 6 (gene/pseudogene)	OR10A6	0,87
THO complex 7	THOC7	0,87
MLX interacting protein	MLXIP	0,87
maltase-glucoamylase	MGAM	0,87
calcium/calmodulin-dependent protein kinase kinase 1, alpha	CAMKK1	0,87
phospholipase A2, group IVA (cytosolic, calcium-dependent)	PLA2G4A	0,87
family with sequence similarity 169, member B	FAM169B	0,87
DEAH (Asp-Glu-Ala-His) box polypeptide 29	DHX29	0,87
YjeF N-terminal domain containing 3	YJEFN3	0,87
CD164 molecule, sialomucin	CD164	0,87
toll interacting protein	TOLLIP	0,87
programmed cell death 6	PDCD6	0,87
chitinase 1 (chitotriosidase)	CHIT1	0,87
N(alpha)-acetyltransferase 25, NatB auxiliary subunit	NAA25	0,87

RAB3A interacting protein	RAB3IP	0,87
Jeck2013 ALT_ACCEPTOR, ALT_DONOR, coding, INTERNAL, intronic best transcript NM_000127; Transcript Identified by AceView	EXT1; spawla	0,87
cap methyltransferase 1	CMTR1	0,87
calcyphosine 2	CAPS2	0,87
protein inhibitor of activated STAT 2	PIAS2	0,87
zona pellucida glycoprotein 1 (sperm receptor)	ZP1	0,87
grainyhead-like transcription factor 1	GRHL1	0,87
lactase	LCT	0,87
BCL2L2-PABPN1 readthrough	BCL2L2-PABPN1	0,87
cyclin-dependent kinase-like 5	CDKL5	0,87
transmembrane protein 68	TMEM68	0,87
cell division cycle 5-like	CDC5L	0,87
HemK methyltransferase family member 1	HEMK1	0,87
ankyrin repeat domain 65	ANKRD65	0,87
pleiotropic regulator 1	PLRG1	0,87
zinc finger protein 645	ZNF645	0,87
ELK4, ETS-domain protein (SRF accessory protein 1)	ELK4	0,87
TSPY-like 5	TSPYL5	0,87
peptidyl arginine deiminase, type IV	PADI4	0,87
UHRF1 binding protein 1-like	UHRF1BP1L	0,87
cancer antigen 1	CAGE1	0,87
chromosome 9 open reading frame 69	C9orf69	0,87
chromosome 9 open reading frame 40	C9orf40	0,87
apolipoprotein E	APOE	0,87
DEAH (Asp-Glu-Ala-His) box polypeptide 8	DHX8	0,87
chromosome 20 open reading frame 27	C20orf27	0,87
proline-rich nuclear receptor coactivator 1	PNRC1	0,87
uncoupling protein 3 (mitochondrial, proton carrier)	UCP3	0,87
clarin 3	CLRN3	0,87
EPPIN-WFDC6 readthrough	EPPIN-WFDC6	0,87
butyrophilin, subfamily 2, member A3, pseudogene	BTN2A3P	0,87
steroid-5-alpha-reductase, alpha polypeptide 2 (3-oxo-5 alpha-steroid delta 4-dehydrogenase alpha 2)	SRD5A2	0,87
asporin	ASPN	0,87
general transcription factor IIIC subunit 1	GTF3C1	0,87
zinc finger protein 627	ZNF627	0,87
ER lipid raft associated 2	ERLIN2	0,87
NADH dehydrogenase (ubiquinone) 1 alpha subcomplex, 6, 14kDa	NDUFA6	0,87
izumo sperm-egg fusion 1	IZUMO1	0,87
neuroblastoma breakpoint family, member 20	NBPF20	0,87
transmembrane protein 86A	TMEM86A	0,87
poly(A) binding protein, nuclear 1	PABPN1	0,87

heterochromatin protein 1, binding protein 3	HP1BP3	0,87
ATP5J2-PTCD1 readthrough; pentatricopeptide repeat domain 1	ATP5J2-PTCD1; PTCD1	0,87
PRAME family member 33, pseudogene	PRAMEF33P	0,87
NLR family, pyrin domain containing 14	NLRP14	0,87
sulfotransferase family 1A member 1	SULT1A1	0,87
arylacetamide deacetylase-like 4	AADACL4	0,87
G protein-coupled receptor 42 (gene/pseudogene)	GPR42	0,87
ATP binding cassette subfamily A member 10	ABCA10	0,87
Rho GTPase activating protein 15	ARHGAP15	0,87
TRPC5 opposite strand	TRPC5OS	0,87
ADAM metallopeptidase with thrombospondin type 1 motif 5	ADAMTS5	0,87
Transcript Identified by AceView, Entrez Gene ID(s) 83699	SH3BGRL2	0,87
LIM homeobox 3	LHX3	0,87
HUS1 checkpoint clamp component B	HUS1B	0,87
nuclear receptor subfamily 2, group C, member 1	NR2C1	0,87
taste receptor, type 2, member 31	TAS2R31	0,87
siah E3 ubiquitin protein ligase 1	SIAH1	0,87
family with sequence similarity 174, member B	FAM174B	0,87
H2.0-like homeobox	HLX	0,87
Memczak2013 ANTISENSE, coding, INTERNAL, intronic best transcript NM_004544	NDUFA10	0,87
olfactory receptor, family 1, subfamily N, member 2	OR1N2	0,87
ATP binding cassette subfamily A member 8	ABCA8	0,87
G protein-coupled receptor 84	GPR84	0,87
polymerase (DNA-directed), delta interacting protein 3	POLDIP3	0,87
trace amine associated receptor 6	TAAR6	0,87
dpy-19-like 3 (C. elegans)	DPY19L3	0,87
chromosome 15 open reading frame 32	C15orf32	0,87
proline rich 22	PRR22	0,87
claudin 24	CLDN24	0,87
small nuclear ribonucleoprotein D2 polypeptide	SNRPD2	0,87
inositol hexakisphosphate kinase 1	IP6K1	0,87
EGF-like, fibronectin type III and laminin G domains	EGFLAM	0,87
Jeck2013 ANTISENSE, CDS, coding, INTERNAL, OVCODE, OVEXON best transcript NM_001042476	CARHSP1	0,87
ceramide-1-phosphate transfer protein	CPTP	0,87
cytidine and dCMP deaminase domain containing 1	CDADC1	0,87
cysteine conjugate-beta lyase 2; RNA binding motif protein, X-linked-like 1	CCBL2; RBMXL1	0,87
guanine nucleotide binding protein (G protein), gamma 11	GNG11	0,87
surfactant protein A1	SFTPA1	0,87

Transcript Identified by AceView, Entrez Gene ID(s) 63892	THADA	0,87
poly(ADP-ribose) polymerase 1	PARP1	0,87
chromosome 11 open reading frame 45	C11orf45	0,87
extracellular matrix protein 2, female organ and adipocyte specific	ECM2	0,87
ubiquitin protein ligase E3 component n-recognin 2	UBR2	0,86
ankyrin repeat domain 23	ANKRD23	0,86
lysine (K)-specific methyltransferase 2E	KMT2E	0,86
t-complex 10-like 2	TCP10L2	0,86
GATA binding protein 2	GATA2	0,86
atlastin GTPase 3	ATL3	0,86
taste receptor, type 2, member 41	TAS2R41	0,86
phosphatidylinositol-3,4,5-trisphosphate-dependent Rac exchange factor 2	PREX2	0,86
leucine rich repeat containing 4B	LRRC4B	0,86
Rho GTPase activating protein 29	ARHGAP29	0,86
olfactory receptor, family 5, subfamily P, member 3	OR5P3	0,86
aquaporin 9	AQP9	0,86
family with sequence similarity 215, member A (non-protein coding)	FAM215A	0,86
spermatogenesis and centriole associated 1-like	SPATC1L	0,86
caspase 14	CASP14	0,86
eomesodermin	EOMES	0,86
ubiquitin specific peptidase 26	USP26	0,86
Ras association (RalGDS/AF-6) domain family (N-terminal) member 7	RASSF7	0,86
sialic acid binding Ig-like lectin 12 (gene/pseudogene)	SIGLEC12	0,86
sterile alpha and TIR motif containing 1	SARM1	0,86
cilia and flagella associated protein 43	CFAP43	0,86
transmembrane protein 74	TMEM74	0,86
keratin associated protein 5-9	KRTAP5-9	0,86
additional sex combs like transcriptional regulator 3	ASXL3	0,86
actin-like 7A	ACTL7A	0,86
calcium channel, voltage-dependent, beta 2 subunit	CACNB2	0,86
isopentenyl-diphosphate delta isomerase 2	IDI2	0,86
ecotropic viral integration site 5-like	EVI5L	0,86
serine peptidase inhibitor, Kazal type 8 (putative)	SPINK8	0,86
solute carrier organic anion transporter family, member 1A2	SLCO1A2	0,86
kallikrein related peptidase 12	KLK12	0,86
methyl-CpG binding domain protein 3-like 2	MBD3L2	0,86
NODAL modulator 3 [Source:HGNC Symbol;Acc:HGNC:25242]; novel transcript, antisense to NOMO3; RP11-517A5.4 (from geneSymbol)	RP11-517A5.5; NOMO3; RP11-517A5.4	0,86

POU class 6 homeobox 1	POU6F1	0,86
DEP domain containing 4	DEPDC4	0,86
SPEG complex locus	SPEG	0,86
speedy/RINGO cell cycle regulator family member A	SPDYA	0,86
Transcript Identified by AceView, Entrez Gene ID(s) 9088	PKMYT1	0,86
DEAD (Asp-Glu-Ala-Asp) box polypeptide 50	DDX50	0,86
Memczak2013 ALT_ACCEPTOR, ALT_DONOR, coding, INTERNAL, intronic best transcript NM_003574	VAPA	0,86
carbohydrate (N-acetylglucosamine 6-O) sulfotransferase 7	CHST7	0,86
cyclic nucleotide gated channel alpha 4	CNGA4	0,86
Transcript Identified by AceView, Entrez Gene ID(s) 4591	TRIM37	0,86
tripartite motif containing 59	TRIM59	0,86
ubiquinol-cytochrome c reductase hinge protein like	UQCRHL	0,86
stomatin (EPB72)-like 3	STOML3	0,86
RPGRIP1-like	RPGRIP1L	0,86
4-hydroxyphenylpyruvate dioxygenase-like	HPDL	0,86
Ellis van Creveld syndrome 2	EVC2	0,86
nuclear receptor binding SET domain protein 1	NSD1	0,86
heterogeneous nuclear ribonucleoprotein C-like 2 [Source:HGNC Symbol;Acc:HGNC:48813]	HNRNPCL2	0,86
Homo sapiens similar to testis specific protein, Y-linked 1, mRNA (cDNA clone MGC:150413 IMAGE:40120433), complete cds.; Homo sapiens testis specific protein, Y-linked 3, mRNA (cDNA clone MGC:150414 IMAGE:40120435), complete cds.	LOC728395; TSPY3	0,86
Memczak2013 ANTISENSE, CDS, coding, INTERNAL best transcript NM_000714	TSPO	0,86
autophagy related 10	ATG10	0,86
guanine nucleotide binding protein (G protein) alpha 12	GNA12	0,86
Transcript Identified by AceView, Entrez Gene ID(s) 55701	RP11-998D10.7; FLJ10357	0,86
proline synthetase co-transcribed homolog (bacterial)	PROSC	0,86
pleckstrin homology-like domain, family B, member 1; microRNA 6716	PHLDB1; MIR6716	0,86
zinc finger protein 385D	ZNF385D	0,86
long intergenic non-protein coding RNA 851	LINC00851	0,86
collagen, type XXVIII, alpha 1	COL28A1	0,86
chromosome 8 open reading frame 31	C8orf31	0,86
thymosin beta 15a	TMSB15A	0,86
ADAM metallopeptidase with thrombospondin type 1 motif 7	ADAMTS7	0,86
caudal type homeobox 4	CDX4	0,86

FEV (ETS oncogene family)	FEV	0,86
importin 9	IPO9	0,86
helicase with zinc finger 2, transcriptional coactivator	HELZ2	0,86
T-cell leukemia/lymphoma 1A	TCL1A	0,86
potassium channel, voltage gated Shal related subfamily D, member 2	KCND2	0,86
proline rich 27	PRR27	0,86
family with sequence similarity 197, Y-linked, member 1	FAM197Y1	0,86
pre-mRNA processing factor 4B	PRPF4B	0,86
matrilin 4	MATN4	0,86
solute carrier family 3 (amino acid transporter heavy chain), member 1	SLC3A1	0,86
Memczak2013 ANTISENSE, CDS, coding, INTERNAL best transcript NM_002649	PIK3CG	0,86
methylthioadenosine phosphorylase	MTAP	0,86
unc-5 family C-terminal like	UNC5CL	0,86
ABO blood group (transferase A, alpha 1-3-N-acetylgalactosaminyltransferase; transferase B, alpha 1-3-galactosyltransferase)	ABO	0,86
piwi-like RNA-mediated gene silencing 2	PIWIL2	0,86
calcium release activated channel regulator 2B	CRACR2B	0,86
zinc finger protein 780A	ZNF780A	0,86
complement factor H-related 2	CFHR2	0,86
lectin, galactoside-binding, soluble, 16	LGALS16	0,86
Transcript Identified by AceView, Entrez Gene ID(s) 2043	EPHA4	0,86
symplekin	SYMPK	0,86
BCL2-like 14 (apoptosis facilitator)	BCL2L14	0,86
regulator of G-protein signaling 21	RGS21	0,86
replication factor C subunit 5	RFC5	0,86
Zhang2013 ALT_DONOR, coding, INTERNAL, intronic best transcript NM_002482	NASP	0,86
trafficking protein particle complex 13	TRAPPC13	0,86
gephyrin	GPHN	0,86
capping protein (actin filament) muscle Z-line, alpha 2	CAPZA2	0,86
cilia and flagella associated protein 161	CFAP161	0,86
C-type lectin domain family 1, member B	CLEC1B	0,86
importin 13	IPO13	0,86
Memczak2013 ALT_DONOR, coding, INTERNAL, intronic best transcript NM_014739	BCLAF1	0,86
CD200 receptor 1 like	CD200R1L	0,86
proprotein convertase subtilisin/kexin type 4	PCSK4	0,86
TNF receptor-associated factor 2	TRAF2	0,86
Rho guanine nucleotide exchange factor 7	ARHGEF7	0,86

trace amine associated receptor 5	TAAR5	0,86
chromosome 14 open reading frame 177	C14orf177	0,86
metadherin	MTDH	0,86
PHD and ring finger domains 1	PHRF1	0,86
transmembrane protein 186	TMEM186	0,86
calcium-binding protein, spermatid-specific 1	CABS1	0,86
cadherin-related 23	CDH23	0,86
keratin associated protein 13-1	KRTAP13-1	0,86
phospholipase D family, member 3	PLD3	0,86
eukaryotic translation elongation factor 1 gamma; microRNA 3654	EEF1G; MIR3654	0,86
proteasome subunit alpha 3	PSMA3	0,86
family with sequence similarity 69, member B	FAM69B	0,86
thiosulfate sulfurtransferase (rhodanese)-like domain containing 2	TSTD2	0,86
mechanistic target of rapamycin (serine/threonine kinase)	MTOR	0,86
src kinase associated phosphoprotein 1	SKAP1	0,86
Transcript Identified by AceView, Entrez Gene ID(s) 10978; novel transcript, sense overlapping CLP1	CLP1; AP000662.4	0,86
chaperonin containing TCP1, subunit 2 (beta)	CCT2	0,86
origin recognition complex subunit 6	ORC6	0,86
SKI/DACH domain containing 1	SKIDA1	0,86
Transcript Identified by AceView, Entrez Gene ID(s) 10580	SORBS1	0,86
adhesion G protein-coupled receptor B3	ADGRB3	0,86
fructose-1,6-bisphosphatase 2	FBP2	0,86
eukaryotic translation initiation factor 3, subunit B	EIF3B	0,86
Fc receptor-like A	FCRLA	0,86
family with sequence similarity 24, member A	FAM24A	0,86
mucin 16, cell surface associated	MUC16	0,86
Werner syndrome, RecQ helicase-like	WRN	0,86
pterin-4 alpha-carbinolamine dehydratase/dimerization cofactor of hepatocyte nuclear factor 1 alpha (TCF1) 2	PCBD2	0,86
brain expressed X-linked 5	BEX5	0,86
tetratricopeptide repeat domain 6	TTC6	0,86
ring finger protein 130	RNF130	0,86
non-SMC condensin II complex subunit G2	NCAPG2	0,86
olfactory receptor, family 10, subfamily X, member 1 (gene/pseudogene)	OR10X1	0,86
ribosomal protein S10	RPS10	0,86
transcription factor Dp family, member 3	TFDP3	0,86
MAS-related GPR, member X3	MRGPRX3	0,86
lin-54 DREAM MuvB core complex component	LIN54	0,86
ribosomal protein L9	RPL9	0,86

ring finger protein 149	RNF149	0,86
keratin associated protein 11-1	KRTAP11-1	0,86
DAZ associated protein 1	DAZAP1	0,86
Fc receptor-like 2	FCRL2	0,86
Transcript Identified by AceView, Entrez Gene ID(s) 5800	PTPRO	0,86
synaptotagmin-like 2	SYTL2	0,86
solute carrier family 37 (glucose-6-phosphate transporter), member 1	SLC37A1	0,86
retinoblastoma-like 1	RBL1	0,86
MON1 secretory trafficking family member A	MON1A	0,86
protein phosphatase 2C-like domain containing 1	PP2D1	0,86
kelch-like family member 35	KLHL35	0,86
epididymal sperm binding protein 1	ELSPBP1	0,86
squamous cell carcinoma antigen recognized by T-cells 1	SART1	0,86
family with sequence similarity 43, member B	FAM43B	0,86
secretogranin V	SCG5	0,86
NADH dehydrogenase (ubiquinone) 1 alpha subcomplex, 1, 7.5kDa	NDUFA1	0,86
tetraspanin 11	TSPAN11	0,86
caspase 10	CASP10	0,86
Transcript Identified by AceView, Entrez Gene ID(s) 80271	ITPKC	0,86
mesoderm posterior bHLH transcription factor 2	MESP2	0,86
triggering receptor expressed on myeloid cells-like 4	TREML4	0,86
ATPase inhibitory factor 1	ATPIF1	0,86
RIMS binding protein 3C	RIMBP3C	0,86
structural maintenance of chromosomes 1A	SMC1A	0,86
phospholipase D family, member 4	PLD4	0,86
family with sequence similarity 217, member A	FAM217A	0,86
olfactory receptor, family 6, subfamily M, member 1	OR6M1	0,86
regulator of G-protein signaling 12	RGS12	0,86
protein kinase C and casein kinase substrate in neurons 1	PACSIN1	0,86
fibroblast growth factor 12	FGF12	0,86
multiple coagulation factor deficiency 2	MCFD2	0,86
kinesin family member 5A	KIF5A	0,86
hedgehog acyltransferase	HHAT	0,86
Rho-related BTB domain containing 2	RHOBTB2	0,86
WAP four-disulfide core domain 11	WFDC11	0,86
NADH dehydrogenase (ubiquinone) 1 alpha subcomplex, 10, 42kDa	NDUFA10	0,86
NK1 homeobox 2	NKX1-2	0,86
chromosome 16 open reading frame 96	C16orf96	0,86

ER degradation enhancer, mannosidase alpha-like 1	EDEM1	0,86
endoplasmic reticulum oxidoreductase beta	ERO1B	0,86
transmembrane protein 126A	TMEM126A	0,86
RNA binding motif, single stranded interacting protein 1; microRNA 4785	RBMS1; MIR4785	0,86
DEAF1 transcription factor	DEAF1	0,86
taste receptor, type 2, member 42	TAS2R42	0,86
phenazine biosynthesis-like protein domain containing	PBLD	0,86
ADAM metallopeptidase with thrombospondin type 1 motif 13	ADAMTS13	0,86
immunoglobulin superfamily containing leucine-rich repeat 2	ISLR2	0,86
protein kinase, X-linked	PRKX	0,86
sosondowah ankyrin repeat domain family member C	SOWAHC	0,86
protein phosphatase, Mg2+/Mn2+ dependent, 1E	PPM1E	0,86
NODAL modulator 2	NOMO2	0,86
aldo-keto reductase family 1, member C3	AKR1C3	0,86
radial spoke head 10 homolog B2 (Chlamydomonas)	RSPH10B2	0,86
kallikrein related peptidase 5	KLK5	0,86
nucleoporin 88kDa	NUP88	0,86
lysine-rich nucleolar protein 1	KNOP1	0,86
topoisomerase (DNA) I, mitochondrial	TOP1MT	0,86
family with sequence similarity 201, member A	FAM201A	0,86
ubiquitin-fold modifier 1	UFM1	0,86
zinc finger protein 502	ZNF502	0,86
dipeptidase 3	DPEP3	0,86
mago homolog, exon junction complex core component	MAGOH	0,86
PML-RARA regulated adaptor molecule 1	PRAM1	0,86
basic transcription factor 3-like 4	BTF3L4	0,86
armadillo repeat containing, X-linked 1	ARMCX1	0,86
synapse defective 1, Rho GTPase, homolog 1 (C. elegans)	SYDE1	0,86
Memczak2013 ALT_ACCEPTOR, ALT_DONOR, coding, INTERNAL, intronic best transcript NM_020397	CAMK1D	0,86
peptidase D	PEPD	0,86
Memczak2013 ALT_ACCEPTOR, ALT_DONOR, coding, INTERNAL, intronic best transcript NM_003664	AP3B1	0,86
Rho GTPase activating protein 8	ARHGAP8	0,86
contactin associated protein-like 3	CNTNAP3	0,86
chromosome 15 open reading frame 62	C15orf62	0,86
zinc finger protein 44	ZNF44	0,86
zinc finger, AN1-type domain 4	ZFAND4	0,86
chromosome 9 open reading frame 131	C9orf131	0,86
SIX homeobox 6	SIX6	0,86
major histocompatibility complex, class II, DO beta	HLA-DOB	0,86

mitogen-activated protein kinase 6	MAPK6	0,86
C-type lectin domain family 2, member B	CLEC2B	0,86
formyl peptide receptor 1	FPR1	0,86
adrenoceptor beta 1	ADRB1	0,86
deleted in malignant brain tumors 1	DMBT1	0,86
chemokine (C-X-C motif) receptor 6	CXCR6	0,86
arginyl-tRNA synthetase	RARS	0,86
BMS1 ribosome biogenesis factor	BMS1	0,86
solute carrier family 16 (monocarboxylate transporter), member 8	SLC16A8	0,86
phosphodiesterase 4B, cAMP-specific	PDE4B	0,86
long intergenic non-protein coding RNA 1561	LINC01561	0,86
cell division cycle 34	CDC34	0,86
methyltransferase like 10	METTL10	0,86
tetratricopeptide repeat domain 22	TTC22	0,86
interleukin 3	IL3	0,86
killer cell lectin-like receptor subfamily F, member 2	KLRF2	0,86
abnormal spindle microtubule assembly	ASPM	0,86
pseudouridine 5-phosphatase	PUDP	0,86
protein O-linked mannose N-acetylglucosaminyltransferase 2 (beta 1,4-)	POMGNT2	0,86
NADPH oxidase organizer 1	NOXO1	0,86
tigger transposable element derived 3	TIGD3	0,86
galectin-related inter-fiber protein	GRIFIN	0,86
Williams-Beuren syndrome chromosome region 17	WBSCR17	0,86
proline, glutamate and leucine rich protein 1	PELP1	0,86
kinesin family member 18A	KIF18A	0,86
interferon, beta 1, fibroblast	IFNB1	0,86
ceramide synthase 1; growth differentiation factor 1	CERS1; GDF1	0,86
peripheral myelin protein 2	PMP2	0,86
aminoadipate-semialdehyde dehydrogenase-phosphopantetheinyl transferase	AASDHPPT	0,86
NFU1 iron-sulfur cluster scaffold	NFU1	0,86
chemokine (C-C motif) ligand 27	CCL27	0,86
Dmx-like 1	DMXL1	0,86
autophagy related 4D, cysteine peptidase	ATG4D	0,86
family with sequence similarity 3, member A	FAM3A	0,86
centromere protein K	CENPK	0,86
kallikrein related peptidase 9	KLK9	0,86
hypermethylated in cancer 1	HIC1	0,86
src-related kinase lacking C-terminal regulatory tyrosine and N-terminal myristylation sites	SRMS	0,86
Memczak2013 ANTISENSE, CDS, coding, INTERNAL, UTR3 best transcript NM_022727	TRMT2A	0,86

NADH dehydrogenase (ubiquinone) 1 alpha subcomplex, 13	NDUFA13	0,86
solute carrier family 35, member E2B	SLC35E2B	0,86
contactin 6	CNTN6	0,86
transmembrane protein 161B	TMEM161B	0,86
dual specificity phosphatase 7	DUSP7	0,86
Transcript Identified by AceView, Entrez Gene ID(s) 55222	LRRC20	0,85
adenosine A2b receptor	ADORA2B	0,85
opsin 1 (cone pigments), medium-wave-sensitive; opsin 1 (cone pigments), medium-wave-sensitive 2; opsin 1 (cone pigments), medium-wave-sensitive 3	OPN1MW; OPN1MW2; OPN1MW3	0,85
myozenin 3	MYOZ3	0,85
chromosome 6 open reading frame 183	C6orf183	0,85
eukaryotic elongation factor 2 kinase	EEF2K	0,85
hyaluronan binding protein 2	HABP2	0,85
upstream binding transcription factor, RNA polymerase I-like 1	UBTFL1	0,85
hydroxymethylbilane synthase	HMBS	0,85
zinc finger protein 785	ZNF785	0,85
Transcript Identified by AceView, Entrez Gene ID(s) 240	ALOX5	0,85
kinesin family member 22	KIF22	0,85
MYCBP associated protein	MYCBPAP	0,85
tankyrase, TRF1-interacting ankyrin-related ADP-ribose polymerase	TNKS	0,85
family with sequence similarity 166, member A	FAM166A	0,85
dermatopontin	DPT	0,85
thyroglobulin	TG	0,85
beclin 2	BECN2	0,85
ribosomal protein L7a	RPL7A	0,85
FAD-dependent oxidoreductase domain containing 1	FOXRED1	0,85
Rho GTPase activating protein 35	ARHGAP35	0,85
tubulin folding cofactor B	TBCB	0,85
ankyrin repeat domain 18A; family with sequence similarity 95, member C	ANKRD18A; FAM95C	0,85
zinc finger protein 16	ZNF16	0,85
cholinergic receptor, nicotinic alpha 1	CHRNA1	0,85
transmembrane protein 25	TMEM25	0,85
HECT and RLD domain containing E3 ubiquitin protein ligase 3	HERC3	0,85
xanthine dehydrogenase	XDH	0,85
secreted phosphoprotein 2	SPP2	0,85
NmrA-like family domain containing 1	NMRAL1	0,85
Zhang2013 ALT_ACCEPTOR, ALT_DONOR, coding, INTERNAL, intronic best transcript NM_030653	DDX11	0,85

Y box binding protein 2	YBX2	0,85
Salzman2013 ALT_ACCEPTOR, ALT_DONOR, coding, INTERNAL, intronic best transcript NM_015092	SMG1	0,85
succinate dehydrogenase complex subunit D, integral membrane protein	SDHD	0,85
zinc finger protein 564	ZNF564	0,85
zinc finger, AN1-type domain 1	ZFAND1	0,85
olfactory receptor, family 12, subfamily D, member 2 (gene/pseudogene)	OR12D2	0,85
coiled-coil domain containing 182	CCDC182	0,85
SLAM family member 6	SLAMF6	0,85
zinc finger protein 398	ZNF398	0,85
contactin 2 (axonal)	CNTN2	0,85
tectorin alpha	TECTA	0,85
transient receptor potential cation channel, subfamily C, member 4 associated protein	TRPC4AP	0,85
transmembrane protease, serine 4	TMPRSS4	0,85
superoxide dismutase 3, extracellular	SOD3	0,85
nucleotide binding protein-like	NUBPL	0,85
1-acylglycerol-3-phosphate O-acyltransferase 5	AGPAT5	0,85
tetratricopeptide repeat domain 28	TTC28	0,85
spondin 2, extracellular matrix protein	SPON2	0,85
uncharacterized LOC100506142; Memczak2013 ANTISENSE, coding, INTERNAL, intronic best transcript NM_012249; novel transcript	LOC100506142; RP11-417F21.1; RHOQ	0,85
transmembrane channel like 1	TMC1	0,85
chromosome 17 open reading frame 51	C17orf51	0,85
adaptor-related protein complex 3, beta 1 subunit	AP3B1	0,85
PDLIM1 interacting kinase 1 like	PDIK1L	0,85
chromosome 14 open reading frame 142	C14orf142	0,85
replication timing regulatory factor 1	RIF1	0,85
adhesion G protein-coupled receptor B1	ADGRB1	0,85
deoxyhypusine hydroxylase/monooxygenase	DOHH	0,85
Transcript Identified by AceView, Entrez Gene ID(s) 8470	SORBS2	0,85
maestro heat-like repeat family member 5	MROH5	0,85
olfactory receptor, family 13, subfamily A, member 1	OR13A1	0,85
RAB14, member RAS oncogene family	RAB14	0,85
olfactory receptor, family 51, subfamily V, member 1	OR51V1	0,85
shroom family member 2	SHROOM2	0,85
stem-loop binding protein	SLBP	0,85
cilia and flagella associated protein 99	CFAP99	0,85
CD40 ligand	CD40LG	0,85
PRAME family member 8	PRAMEF8	0,85
premelanosome protein	PMEL	0,85

UDP-GlcNAc:betaGal beta-1,3-N-acetylglucosaminyltransferase-like 1	B3GNTL1	0,85
zinc finger protein 429	ZNF429	0,85
gamma-aminobutyric acid (GABA) A receptor, beta 1	GABRB1	0,85
DND microRNA-mediated repression inhibitor 1	DND1	0,85
5-nucleotidase, cytosolic IA	NT5C1A	0,85
structural maintenance of chromosomes flexible hinge domain containing 1	SMCHD1	0,85
solute carrier family 25 (aspartate/glutamate carrier), member 13	SLC25A13	0,85
transmembrane protein 82	TMEM82	0,85
short stature homeobox	SHOX	0,85
T-cell immunoreceptor with Ig and ITIM domains	TIGIT	0,85
MLX, MAX dimerization protein	MLX	0,85
dipeptidyl-peptidase 6	DPP6	0,85
transmembrane protein 52	TMEM52	0,85
solute carrier family 25 (S-adenosylmethionine carrier), member 26	SLC25A26	0,85
Fanconi anemia complementation group G	FANCG	0,85
intraflagellar transport 81	IFT81	0,85
MMS22-like, DNA repair protein	MMS22L	0,85
S100 calcium binding protein A12	S100A12	0,85
transcription elongation factor A (SII), 1	TCEA1	0,85
chromosome 4 open reading frame 27	C4orf27	0,85
mex-3 RNA binding family member B	MEX3B	0,85
RAB40C, member RAS oncogene family	RAB40C	0,85
melanocortin 3 receptor	MC3R	0,85
olfactory receptor, family 6, subfamily K, member 3	OR6K3	0,85
clavesin 2	CLVS2	0,85
dynein, axonemal, heavy chain 10	DNAH10	0,85
NADH dehydrogenase (ubiquinone) 1 alpha subcomplex, 2, 8kDa	NDUFA2	0,85
DEAD (Asp-Glu-Ala-Asp) box polypeptide 60	DDX60	0,85
secreted LY6/PLAUR domain containing 1	SLURP1	0,85
BRF1, RNA polymerase III transcription initiation factor 90 kDa subunit	BRF1	0,85
POU class 3 homeobox 2	POU3F2	0,85
protein kinase C, beta	PRKCB	0,85
zinc finger and BTB domain containing 44	ZBTB44	0,85
zinc finger and BTB domain containing 7C	ZBTB7C	0,85
cathelicidin antimicrobial peptide	CAMP	0,85
	LRIG1	0,85
olfactory receptor, family 6, subfamily C, member 65	OR6C65	0,85
BEN domain containing 4	BEND4	0,85
family with sequence similarity 83, member F	FAM83F	0,85

WAP four-disulfide core domain 12	WFDC12	0,85
nucleophosmin (nucleolar phosphoprotein B23, numatrin)	NPM1	0,85
coagulation factor III (thromboplastin, tissue factor)	F3	0,85
MAP6 domain containing 1	MAP6D1	0,85
retinoic acid receptor responder (tazarotene induced) 1	RARRES1	0,85
bone morphogenetic protein 5	BMP5	0,85
Fanconi anemia complementation group F	FANCF	0,85
translocase of outer mitochondrial membrane 6 homolog (yeast); prickle homolog 4	TOMM6; PRICKLE4	0,85
WD repeat domain 88	WDR88	0,85
proline-rich coiled-coil 2C	PRRC2C	0,85
zinc finger protein 696	ZNF696	0,85
papillary renal cell carcinoma (translocation-associated)	PRCC	0,85
brain-enriched guanylate kinase-associated	BEGAIN	0,85
aryl hydrocarbon receptor nuclear translocator-like	ARNTL	0,85
cortexin 1	CTXN1	0,85
STX16-NPEPL1 readthrough (NMD candidate)	STX16-NPEPL1	0,85
pleckstrin	PLEK	0,85
mucin 3A, cell surface associated	MUC3A	0,85
lysine (K)-specific demethylase 4A	KDM4A	0,85
zinc finger protein 791	ZNF791	0,85
selectin E	SELE	0,85
SLIT-ROBO Rho GTPase activating protein 2C	SRGAP2C	0,85
RAP1A, member of RAS oncogene family	RAP1A	0,85
translocase of outer mitochondrial membrane 22 homolog (yeast)	TOMM22	0,85
glutamate receptor, ionotropic, N-methyl D-aspartate 1	GRIN1	0,85
centrosomal protein 250kDa	CEP250	0,85
paired box 3	PAX3	0,85
family with sequence similarity 69, member A	FAM69A	0,85
calcium channel, voltage-dependent, alpha 2/delta subunit 1	CACNA2D1	0,85
zinc finger protein 12	ZNF12	0,85
membrane-spanning 4-domains, subfamily A, member 8	MS4A8	0,85
stromal antigen 3-like 1 (pseudogene)	STAG3L1	0,85
claudin 12	CLDN12	0,85
olfactory receptor, family 4, subfamily F, member 15	OR4F15	0,85
ring finger protein 39	RNF39	0,85
twist family bHLH transcription factor 2	TWIST2	0,85
zinc finger protein 672	ZNF672	0,85
low density lipoprotein receptor class A domain containing 3	LDLRAD3	0,85
doublecortin-like kinase 3	DCLK3	0,85

family with sequence similarity 81, member B	FAM81B	0,85
forkhead box L2	FOXL2	0,85
required for meiotic nuclear division 5 homolog B	RMND5B	0,85
uncharacterized LOC285692; novel transcript; Transcript Identified by AceView, Entrez Gene ID(s) 285692; 50834	LOC285692; CTD-2143L24.1; TAS2R1	0,85
solute carrier family 38, member 10	SLC38A10	0,85
ADP-ribosylation factor like GTPase 14	ARL14	0,85
outer dense fiber of sperm tails 1	ODF1	0,85
myosin light chain 5	MYL5	0,85
potassium channel, inwardly rectifying subfamily J, member 18	KCNJ18	0,85
chordin-like 2	CHRDL2	0,85
deoxythymidylate kinase	DTYMK	0,85
Transcript Identified by AceView, Entrez Gene ID(s) 79868	ALG13	0,85
integrin alpha 8	ITGA8	0,85
solute carrier family 13 (sodium-dependent dicarboxylate transporter), member 3; TP53 regulating kinase	SLC13A3; TP53RK	0,85
tyrosine kinase, non-receptor, 1	TNK1	0,85
ATP synthase, H+ transporting, mitochondrial Fo complex subunit F6	ATP5J	0,85
olfactory receptor, family 10, subfamily G, member 4	OR10G4	0,85
transmembrane protein 55B	TMEM55B	0,85
zinc finger protein 236	ZNF236	0,85
MGAT4 family, member C	MGAT4C	0,85
PRAME family member 2	PRAMEF2	0,85
Transcript Identified by AceView, Entrez Gene ID(s) 63899	NSUN3	0,85
amyloid beta (A4) precursor protein-binding, family B, member 3	APBB3	0,85
signal-induced proliferation-associated 1 like 3	SIPA1L3	0,85
forkhead box O3	FOXO3	0,85
CTAGE family, member 8; CTAGE family, member 4	CTAGE8; CTAGE4	0,85
diacylglycerol kinase, delta 130kDa	DGKD	0,85
zinc finger CCCH-type containing 7B	ZC3H7B	0,85
HECT and RLD domain containing E3 ubiquitin protein ligase 5	HERC5	0,85
uncharacterized LOC101928137; novel transcript	LOC101928137; RP11-314D7.2	0,85
cysteinyl leukotriene receptor 1	CYSLTR1	0,85
embryonic ectoderm development; microRNA 6755	EED; MIR6755	0,85
nescient helix-loop-helix 1	NHLH1	0,85
RE1-silencing transcription factor	REST	0,85

KIAA1143 [Source:HGNC Symbol;Acc:HGNC:29198]	KIAA1143	0,85
long intergenic non-protein coding RNA 282	LINC00282	0,85
coatomer protein complex subunit zeta 2	COPZ2	0,85
mitochondrial ribosomal protein S14	MRPS14	0,85
heterogeneous nuclear ribonucleoprotein C-like 1	HNRNPCL1	0,85
inturned planar cell polarity protein	INTU	0,85
solute carrier family 39 (zinc transporter), member 4	SLC39A4	0,85
tectonic family member 1	TCTN1	0,85
neuroligin 2	NLGN2	0,85
zinc finger protein 771	ZNF771	0,85
uroplakin 2	UPK2	0,85
solute carrier family 52 (riboflavin transporter), member 3	SLC52A3	0,85
inositol-trisphosphate 3-kinase A	ITPKA	0,85
Memczak2013 ALT_ACCEPTOR, ALT_DONOR, coding, INTERNAL, intronic best transcript NM_001098512	PRKG1	0,85
ADAM metallopeptidase with thrombospondin type 1 motif 13	ADAMTS13	0,85
peroxisomal biogenesis factor 5-like	PEX5L	0,85
ring finger protein 113A	RNF113A	0,85
low density lipoprotein receptor; microRNA 6886	LDLR; MIR6886	0,85
IKAROS family zinc finger 1	IKZF1	0,85
prospero homeobox 2	PROX2	0,85
multiple EGF-like-domains 10	MEGF10	0,85
synaptotagmin XIII	SYT13	0,85
CUGBP, Elav-like family member 4	CELF4	0,85
zinc finger and BTB domain containing 26	ZBTB26	0,85
interleukin 5 receptor, alpha	IL5RA	0,85
ryanodine receptor 1 (skeletal)	RYR1	0,85
GRB10 interacting GYF protein 1	GIGYF1	0,85
trafficking protein particle complex 4	TRAPPC4	0,85
abhydrolase domain containing 16A	ABHD16A	0,85
cyclin-dependent kinase 13	CDK13	0,85
B-cell CLL/lymphoma 2	BCL2	0,85
p53-induced death domain protein 1	PIDD1	0,85
Memczak2013 ANTISENSE, coding, INTERNAL, intronic best transcript NM_003940	USP13	0,85
deltex 3 like, E3 ubiquitin ligase	DTX3L	0,85
keratin associated protein 9-8	KRTAP9-8	0,85
translocase of outer mitochondrial membrane 40 homolog (yeast)-like; microRNA 5187	TOMM40L; MIR5187	0,85
RAB33A, member RAS oncogene family	RAB33A	0,85
disco-interacting protein 2 homolog A	DIP2A	0,85
chitinase, acidic	CHIA	0,85
DNA-damage inducible 1 homolog 1	DDI1	0,85

BCL2-associated transcription factor 1	BCLAF1	0,85
ribosomal protein S2; small nucleolar RNA, H/ACA box 64; small nucleolar RNA, H/ACA box 10	RPS2; SNORA64; SNORA10	0,85
p21 protein (Cdc42/Rac)-activated kinase 7	PAK7	0,85
olfactory receptor, family 5, subfamily M, member 10	OR5M10	0,85
G protein-coupled receptor 25	GPR25	0,85
keratin associated protein 10-10	KRTAP10-10	0,85
peroxiredoxin 1	PRDX1	0,85
protein phosphatase 1, regulatory subunit 3E	PPP1R3E	0,85
nucleosome assembly protein 1-like 6	NAP1L6	0,84
solute carrier family 39 (zinc transporter), member 8	SLC39A8	0,84
nucleophosmin/nucleoplasmin 3	NPM3	0,84
coiled-coil domain containing 15	CCDC15	0,84
myosin IIIA	MYO3A	0,84
resistin	RETN	0,84
hyaluronan synthase 1	HAS1	0,84
collagen, type V, alpha 3	COL5A3	0,84
olfactory receptor, family 5, subfamily AC, member 2	OR5AC2	0,84
transcriptional adaptor 3	TADA3	0,84
chondroadherin	CHAD	0,84
endothelin 3	EDN3	0,84
CD163 molecule	CD163	0,84
Transcript Identified by AceView, Entrez Gene ID(s) 100288237; 7700	ZNF141	0,84
ankyrin repeat and LEM domain containing 1	ANKLE1	0,84
aggrecan	ACAN	0,84
otogelin	OTOG	0,84
family with sequence similarity 101, member A	FAM101A	0,84
Homo sapiens TBC1 domain family, member 3H (TBC1D3H), mRNA.; Homo sapiens TBC1 domain family, member 3L (TBC1D3L), mRNA.; Homo sapiens TBC1 domain family, member 3I (TBC1D3I), mRNA.; Homo sapiens TBC1 domain family member-like (LOC101060389), mRNA.; TBC1 domain family member-like; Homo sapiens TBC1 domain family, member 3C, mRNA (cDNA clone MGC:87891 IMAGE:5165385), complete cds.; Homo sapiens TBC1 domain family, member 3, mRNA (cDNA clone MGC:87893 IMAGE:5742850), complete cds.; TBC1 domain family, member 3D [Source:HGNC Symbol;Acc:HGNC:28944]	TBC1D3H; TBC1D3L; TBC1D3I; LOC101060389; TBC1D3C; TBC1D3; TBC1D3D	0,84
hedgehog acyltransferase	HHAT	0,84
mitogen-activated protein kinase kinase kinase kinase 1	MAP4K1	0,84
GDP-mannose pyrophosphorylase A	GMPPA	0,84
RALY heterogeneous nuclear ribonucleoprotein	RALY	0,84
PARP1 binding protein	PARPBP	0,84

nuclear pore complex interacting protein family, member A8	NPIPA8	0,84
thymocyte selection-associated high mobility group box	TOX	0,84
C1q and tumor necrosis factor related protein 4	C1QTNF4	0,84
sperm associated antigen 8	SPAG8	0,84
pleckstrin homology domain containing, family A member 7	PLEKHA7	0,84
angiotensin I converting enzyme 2	ACE2	0,84
N-acetyltransferase 10 (GCN5-related)	NAT10	0,84
family with sequence similarity 19 (chemokine (C-C motif)-like), member A1	FAM19A1	0,84
protein tyrosine phosphatase, receptor type, Q	PTPRQ	0,84
Transcript Identified by AceView, Entrez Gene ID(s) 26084	SGEF	0,84
intraflagellar transport 140	IFT140	0,84
homeobox A13	HOXA13	0,84
secernin 2	SCRN2	0,84
DnaJ (Hsp40) homolog, subfamily C, member 5 gamma	DNAJC5G	0,84
5-methyltetrahydrofolate-homocysteine methyltransferase reductase	MTRR	0,84
indoleamine 2,3-dioxygenase 2	IDO2	0,84
COX19 cytochrome c oxidase assembly factor	COX19	0,84
mitochondrial ribosomal protein L2	MRPL2	0,84
olfactory receptor, family 6, subfamily C, member 2	OR6C2	0,84
guanine nucleotide binding protein (G protein), alpha activating activity polypeptide O	GNAO1	0,84
RNA binding motif protein 25	RBM25	0,84
hydroxymethylbilane synthase	HMBS	0,84
semenogelin II	SEMG2	0,84
left-right determination factor 2	LEFTY2	0,84
abhydrolase domain containing 13	ABHD13	0,84
histone cluster 3, H2a	HIST3H2A	0,84
dual specificity tyrosine-(Y)-phosphorylation regulated kinase 1B	DYRK1B	0,84
PCF11 cleavage and polyadenylation factor subunit	PCF11	0,84
microtubule associated tumor suppressor 1	MTUS1	0,84
solute carrier family 9, subfamily A (NHE9, cation proton antiporter 9), member 9	SLC9A9	0,84
branched chain keto acid dehydrogenase E1, beta polypeptide	BCKDHB	0,84
olfactory receptor, family 2, subfamily F, member 1 (gene/pseudogene)	OR2F1	0,84
v-mos Moloney murine sarcoma viral oncogene homolog	MOS	0,84
PEST proteolytic signal containing nuclear protein	PCNP	0,84

polypeptide N-acetylgalactosaminyltransferase 9	GALNT9	0,84
zinc finger, imprinted 3	ZIM3	0,84
N-acetylated alpha-linked acidic dipeptidase-like 2	NAALADL2	0,84
tubulin polymerization-promoting protein family member 2	TPPP2	0,84
WD repeat domain 82	WDR82	0,84
synaptotagmin VIII	SYT8	0,84
2,3-bisphosphoglycerate mutase	BPGM	0,84
endoplasmic reticulum protein 44	ERP44	0,84
DiGeorge syndrome critical region gene 6	DGCR6	0,84
carbonic anhydrase VB, mitochondrial	CA5B	0,84
Transcript Identified by AceView, Entrez Gene ID(s) 56474	CTPS2	0,84
chromosome 17 open reading frame 99	C17orf99	0,84
fatty acid binding protein 4, adipocyte	FABP4	0,84
cytochrome P450, family 1, subfamily A, polypeptide 2	CYP1A2	0,84
cerebellin 2 precursor	CBLN2	0,84
long intergenic non-protein coding RNA 260	LINC00260	0,84
trafficking protein particle complex 9	TRAPPC9	0,84
exocyst complex component 4	EXOC4	0,84
zinc finger protein 638	ZNF638	0,84
ring finger protein 212	RNF212	0,84
UDP-Gal:betaGlcNAc beta 1,3-galactosyltransferase 4	B3GALT4	0,84
methyl-CpG binding protein 2	MECP2	0,84
peptidylprolyl isomerase A (cyclophilin A)-like 4F; peptidylprolyl isomerase A (cyclophilin A)-like 4D	PPIAL4F; PPIAL4D	0,84
interleukin 17C	IL17C	0,84
WD repeat domain 3	WDR3	0,84
parathymosin	PTMS	0,84
ubiquitin specific peptidase 42	USP42	0,84
synaptopodin 2	SYNPO2	0,84
Transcript Identified by AceView, Entrez Gene ID(s) 79598	CEP97	0,84

hepatoma-derived growth factor-related protein 2; Hepatoma-derived growth factor-related protein 2 [Source:UniProtKB/Swiss-Prot;Acc:Q7Z4V5]; Salzman2013 ANNOTATED, CDS, coding, OVCODE, OVEXON, UTR5 best transcript NM_001001520; Salzman2013 ANNOTATED, CDS, coding, OVCODE, OVERLAPTX, OVEXON, UTR3, UTR5 best transcript NM_001001520; Salzman2013 ANNOTATED, CDS, coding, INTERNAL, OVCODE, OVEXON best transcript NM_001001520; Salzman2013 ALT_ACCEPTOR, CDS, coding, INTERNAL, intronic, OVCODE, OVEXON best transcript NM_001001520; Salzman2013 ANNOTATED, CDS, coding, OVCODE, OVERLAPTX, OVEXON, UTR3 best transcript NM_001001520; Salzman2013 ALT_DONOR, CDS, coding, INTERNAL, OVCODE, OVERLAPTX, OVEXON, UTR3 best transcript NM_001001520	HDGFRP2	0,84
mitochondrial ribosomal protein L42	MRPL42	0,84
nucleoporin 54kDa	NUP54	0,84
ribosomal protein S25	RPS25	0,84
PDZ domain containing 4	PDZD4	0,84
olfactory receptor, family 5, subfamily D, member 13 (gene/pseudogene)	OR5D13	0,84
YTH domain containing 1	YTHDC1	0,84
lysine (K)-specific demethylase 5A	KDM5A	0,84
S-antigen; retina and pineal gland (arrestin)	SAG	0,84
kallikrein related peptidase 4	KLK4	0,84
nuclear GTPase, germinal center associated	NUGGC	0,84
contactin 3 (plasmacytoma associated)	CNTN3	0,84
phospholipase D2	PLD2	0,84
solute carrier family 1 (glial high affinity glutamate transporter), member 2	SLC1A2	0,84
nth-like DNA glycosylase 1	NTHL1	0,84
D-amino-acid oxidase	DAO	0,84
Zhang2013 ALT_ACCEPTOR, ALT_DONOR, coding, INTERNAL, intronic best transcript NM_002953	RPS6KA1	0,84
3-hydroxyacyl-CoA dehydratase 2	HACD2	0,84
sarcolipin	SLN	0,84
polymerase (RNA) II (DNA directed) polypeptide F; microRNA 4534; microRNA 6820	POLR2F; MIR4534; MIR6820	0,84
phosphatase and actin regulator 4	PHACTR4	0,84
zinc finger protein 404	ZNF404	0,84
adducin 1 (alpha)	ADD1	0,84
hydroxymethylbilane synthase	HMBS	0,84
WD repeat domain 93	WDR93	0,84

glutamate receptor, metabotropic 3	GRM3	0,84
copine III	CPNE3	0,84
phosphodiesterase 8A	PDE8A	0,84
UDP glucuronosyltransferase 2 family, polypeptide B17	UGT2B17	0,84
zinc finger with KRAB and SCAN domains 2	ZKSCAN2	0,84
WEE1 G2 checkpoint kinase	WEE1	0,84
zinc finger protein 660	ZNF660	0,84
sorting nexin 5	SNX5	0,84
C-type lectin domain family 2, member A	CLEC2A	0,84
putative homeodomain transcription factor 1	PHTF1	0,84
InaF-motif containing 2	INAFM2	0,84
small EDRK-rich factor 2	SERF2	0,84
Transcript Identified by AceView, Entrez Gene ID(s) 729475	RAD51AP2	0,84
autoimmune regulator	AIRE	0,84
solute carrier family 1 (glutamate/neutral amino acid transporter), member 4	SLC1A4	0,84
ubiquitin conjugating enzyme E2 variant 2	UBE2V2	0,84
chromosome 5 open reading frame 45	C5orf45	0,84
chromosome 9 open reading frame 50	C9orf50	0,84
Jeck2013 ALT_ACCEPTOR, ALT_DONOR, coding, INTERNAL, intronic best transcript NM_001100164	PHACTR2	0,84
testis expressed 36	TEX36	0,84
olfactory receptor, family 13, subfamily C, member 4	OR13C4	0,84
proline rich 15-like	PRR15L	0,84
SLIT and NTRK-like family, member 1	SLITRK1	0,84
mitochondrial ribosomal protein S35	MRPS35	0,84
canopy FGF signaling regulator 4	CNPY4	0,84
signal transducer and activator of transcription 5B	STAT5B	0,84
EP300 interacting inhibitor of differentiation 2B	EID2B	0,84
Ts translation elongation factor, mitochondrial	TSFM	0,84
pre T-cell antigen receptor alpha	PTCRA	0,84
family with sequence similarity 83, member E	FAM83E	0,84
mitochondrial translational release factor 1-like	MTRF1L	0,84
sex comb on midleg-like 2 (Drosophila)	SCML2	0,84
IQ motif containing B1	IQCB1	0,84
testis expressed 13A	TEX13A	0,84
DDB1 and CUL4 associated factor 12-like 2	DCAF12L2	0,84
Memczak2013 ANTISENSE, CDS, coding, INTERNAL, intronic best transcript NM_004475	FLOT2	0,84
parvin, beta	PARVB	0,84
coiled-coil domain containing 38	CCDC38	0,84
huntingtin interacting protein 1 related	HIP1R	0,84
Memczak2013 ALT_ACCEPTOR, ALT_DONOR, coding, INTERNAL, intronic best transcript NM_001164162	PPP6R3	0,84

NADH dehydrogenase (ubiquinone) complex I, assembly factor 2	NDUFAF2	0,84
zinc finger protein 385D	ZNF385D	0,84
E74-like factor 2 (ets domain transcription factor)	ELF2	0,84
basigin (Ok blood group)	BSG	0,84
signal-regulatory protein delta	SIRPD	0,84
SIL1 nucleotide exchange factor	SIL1	0,84
kidney associated antigen 1	KAAG1	0,84
CUGBP, Elav-like family member 5	CELF5	0,84
pepsinogen 3, group I (pepsinogen A); pepsinogen 4, group I (pepsinogen A)	PGA3; PGA4	0,84
HOXA cluster antisense RNA 3	HOXA-AS3	0,84
INO80B-WBP1 readthrough (NMD candidate)	INO80B-WBP1	0,84
C-type lectin domain family 12, member B	CLEC12B	0,84
family with sequence similarity 209, member B	FAM209B	0,84
ribosomal protein L18	RPL18	0,84
microsomal glutathione S-transferase 1	MGST1	0,84
MYCBP-associated, testis expressed 1	MAATS1	0,84
solute carrier family 35, member E3	SLC35E3	0,84
DCN1, defective in cullin neddylation 1, domain containing 5	DCUN1D5	0,84
zinc finger protein 467	ZNF467	0,84
olfactory receptor, family 51, subfamily E, member 1	OR51E1	0,84
centrosomal protein 95kDa	CEP95	0,84
ATP binding cassette subfamily D member 1	ABCD1	0,84
FYN binding protein	FYB	0,84
mitochondrial ribosomal protein L36	MRPL36	0,84
peptidyl arginine deiminase, type I	PADI1	0,84
CDGSH iron sulfur domain 2	CISD2	0,84
jumonji domain containing 4	JMJD4	0,84
keratin associated protein 13-2	KRTAP13-2	0,84
homocysteine-inducible, endoplasmic reticulum stress-inducible, ubiquitin-like domain member 1	HERPUD1	0,84
F-box protein 44	FBXO44	0,84
zinc finger protein 320	ZNF320	0,84
homeobox A2	HOXA2	0,84
poly(A)-specific ribonuclease	PARN	0,84
echinoderm microtubule associated protein like 1	EML1	0,84
hexamethylene bis-acetamide inducible 1	HEXIM1	0,84
dual specificity tyrosine-(Y)-phosphorylation regulated kinase 2	DYRK2	0,84
5-oxoprolinase (ATP-hydrolysing)	OPLAH	0,84
interleukin 26	IL26	0,84
retinal pigment epithelium-specific protein 65kDa	RPE65	0,84

sphingomyelin phosphodiesterase 3, neutral membrane (neutral sphingomyelinase II)	SMPD3	0,84
ER membrane protein complex subunit 6	EMC6	0,84
SET domain containing 2	SETD2	0,84
defensin, beta 115	DEFB115	0,84
ST8 alpha-N-acetyl-neuraminide alpha-2,8-sialyltransferase 6	ST8SIA6	0,84
docking protein 1	DOK1	0,84
coiled-coil domain containing 78	CCDC78	0,84
ALG12, alpha-1,6-mannosyltransferase	ALG12	0,84
apolipoprotein L, 5	APOL5	0,84
WD repeat, sterile alpha motif and U-box domain containing 1	WDSUB1	0,84
lecithin retinol acyltransferase (phosphatidylcholine--retinol O-acyltransferase)	LRAT	0,84
BRCA1 associated protein 1	BAP1	0,84
tripartite motif containing 64	TRIM64	0,84
GRIP1 associated protein 1	GRIPAP1	0,84
solute carrier family 35 (GDP-fucose transporter), member C1	SLC35C1	0,84
C1q and tumor necrosis factor related protein 7	C1QTNF7	0,84
complement component 1, q subcomponent, C chain	C1QC	0,84
potassium channel, inwardly rectifying subfamily J, member 9	KCNJ9	0,84
ubiquitin specific peptidase 50	USP50	0,84
GINS complex subunit 3 (Psf3 homolog)	GINS3	0,84
ribosomal RNA processing 8, methyltransferase, homolog (yeast)	RRP8	0,84
ribosomal protein L10; small nucleolar RNA, H/ACA box 70	RPL10; SNORA70	0,84
mitochondrial ribosome-associated GTPase 2	MTG2	0,84
Mdm1 nuclear protein	MDM1	0,84
long intergenic non-protein coding RNA 1225	LINC01225	0,84
growth factor receptor bound protein 2	GRB2	0,84
ADP-ribosylhydrolase like 2	ADPRHL2	0,84
COMM domain containing 9	COMMD9	0,84
cleavage and polyadenylation specific factor 7	CPSF7	0,84
Ral GEF with PH domain and SH3 binding motif 2	RALGPS2	0,84
signal-induced proliferation-associated 1 like 1	SIPA1L1	0,84
ribosomal protein L8; microRNA 6850	RPL8; MIR6850	0,84
amyloid beta (A4) precursor protein-binding, family A, member 1	APBA1	0,84
zinc finger, MYM-type 4	ZMYM4	0,84
pecanex-like 4 (Drosophila)	PCNXL4	0,84
protocadherin beta 1	PCDHB1	0,84

olfactory receptor, family 2, subfamily L, member 3	OR2L3	0,84
CD38 molecule	CD38	0,84
forkhead box O6	FOXO6	0,83
SEC24 homolog B, COPII coat complex component	SEC24B	0,83
olfactory receptor, family 8, subfamily B, member 3	OR8B3	0,83
ribosomal protein L26	RPL26	0,83
GS homeobox 2	GSX2	0,83
adenylate kinase 7	AK7	0,83
short chain dehydrogenase/reductase family 39U, member 1	SDR39U1	0,83
chromosome 16 open reading frame 13	C16orf13	0,83
small integral membrane protein 1 (Vel blood group)	SMIM1	0,83
family with sequence similarity 26, member E	FAM26E	0,83
chromosome 11 open reading frame 42	C11orf42	0,83
cytoplasmic polyadenylation element binding protein 3	CPEB3	0,83
maltase-glucoamylase	MGAM	0,83
estrogen-related receptor alpha	ESRRA	0,83
family with sequence similarity 153, member C, pseudogene	FAM153C	0,83
WD repeat domain 89	WDR89	0,83
PDZ and LIM domain 2 (mystique)	PDLIM2	0,83
RBPJ interacting and tubulin associated 1	RITA1	0,83
adhesion G protein-coupled receptor L3	ADGRL3	0,83
tubulin, gamma 2	TUBG2	0,83
SMC5-SMC6 complex localization factor 1	SLF1	0,83
chromosome 1 open reading frame 109	C1orf109	0,83
protein kinase C, theta	PRKCQ	0,83
retinoblastoma-like 2	RBL2	0,83
exocyst complex component 6B	EXOC6B	0,83
Transcript Identified by AceView, Entrez Gene ID(s) 1997; 100128628	ELF1	0,83
ubiquitin protein ligase E3C	UBE3C	0,83
phospholipid phosphatase related 5	PLPPR5	0,83
ATP binding cassette subfamily G member 4	ABCG4	0,83
neuronatin	NNAT	0,83
kinase D-interacting substrate 220kDa	KIDINS220	0,83
Transcript Identified by AceView, Entrez Gene ID(s) 4702	NDUFA8	0,83
ribosomal protein S11; small nucleolar RNA, C/D box 35B	RPS11; SNORD35B	0,83
solute carrier family 35, member G3	SLC35G3	0,83
UDP-Gal:betaGlcNAc beta 1,3-galactosyltransferase 2	B3GALT2	0,83
olfactory receptor, family 8, subfamily B, member 4 (gene/pseudogene)	OR8B4	0,83
growth differentiation factor 6	GDF6	0,83

atypical chemokine receptor 3	ACKR3	0,83
translocase of inner mitochondrial membrane 50 homolog (S. cerevisiae)	TIMM50	0,83
kelch-like family member 26	KLHL26	0,83
Rho GTPase activating protein 11B	ARHGAP11B	0,83
ribosomal protein L37	RPL37	0,83
DiGeorge syndrome critical region gene 6-like	DGCR6L	0,83
Transcript Identified by AceView, Entrez Gene ID(s) 10485; novel transcript	C1orf61; RP11-98G7.1	0,83
gap junction protein beta 2	GJB2	0,83
ATPase, class VI, type 11A	ATP11A	0,83
mannosidase, alpha, class 2B, member 1	MAN2B1	0,83
neuronal PAS domain protein 2	NPAS2	0,83
protease, serine, 56	PRSS56	0,83
nuclear receptor binding protein 1	NRBP1	0,83
lipoprotein, Lp(a)	LPA	0,83
impact RWD domain protein	IMPACT	0,83
adenylate kinase 2	AK2	0,83
cerebral cavernous malformation 2-like	CCM2L	0,83
cytochrome P450, family 7, subfamily B, polypeptide 1	CYP7B1	0,83
chromosome 3 open reading frame 14	C3orf14	0,83
zinc finger and SCAN domain containing 12	ZSCAN12	0,83
growth arrest and DNA-damage-inducible, gamma interacting protein 1	GADD45GIP1	0,83
olfactory receptor, family 4, subfamily K, member 5	OR4K5	0,83
coatomer protein complex subunit epsilon	COPE	0,83
lactoperoxidase	LPO	0,83
leucine-rich repeat, immunoglobulin-like and transmembrane domains 3	LRIT3	0,83
solute carrier family 16, member 9	SLC16A9	0,83
zinc finger protein 780A	ZNF780A	0,83
pentatricopeptide repeat domain 1	PTCD1	0,83
Memczak2013 ALT_ACCEPTOR, ALT_DONOR, coding, INTERNAL, intronic best transcript NM_004972	JAK2	0,83
exosome component 3	EXOSC3	0,83
phosphatidylinositol glycan anchor biosynthesis class U	PIGU	0,83
nuclear transcription factor Y subunit alpha	NFYA	0,83
insulin-like growth factor binding protein, acid labile subunit	IGFALS	0,83
progesterone receptor	PGR	0,83
serine/threonine kinase 19	STK19	0,83
olfactory receptor, family 52, subfamily I, member 1	OR52I1	0,83
leptin receptor; leptin receptor overlapping transcript	LEPR; LEPROT	0,83
cholinergic receptor, nicotinic epsilon	CHRNE	0,83
glutathione peroxidase 3	GPX3	0,83

Zhang2013 ALT_ACCEPTOR, ALT_DONOR, coding, INTERNAL, intronic best transcript NM_014238	KSR1	0,83
NADH dehydrogenase (ubiquinone) 1 alpha subcomplex, 10, 42kDa	NDUFA10	0,83
CART prepropeptide	CARTPT	0,83
olfactory receptor, family 6, subfamily J, member 1 (gene/pseudogene)	OR6J1	0,83
acidic nuclear phosphoprotein 32 family member D	ANP32D	0,83
nuclear transcription factor Y subunit gamma	NFYC	0,83
deoxyribonuclease II, lysosomal	DNASE2	0,83
nuclear pore complex interacting protein family, member A7	NPIPA7	0,83
chromosome 21 open reading frame 62	C21orf62	0,83
diacylglycerol kinase, delta 130kDa	DGKD	0,83
RAB3A, member RAS oncogene family	RAB3A	0,83
Transcript Identified by AceView, Entrez Gene ID(s) 8502	PKP4	0,83
crumbs family member 3	CRB3	0,83
chromosome 19 open reading frame 71	C19orf71	0,83
neurofibromin 1	NF1	0,83
ANKH inorganic pyrophosphate transport regulator	ANKH	0,83
tyrosyl-tRNA synthetase 2, mitochondrial	YARS2	0,83
protein tyrosine phosphatase, receptor type, f polypeptide (PTPRF), interacting protein (liprin), alpha 1	PPFIA1	0,83
BRICHOS domain containing 5	BRICD5	0,83
collagen, type XX, alpha 1	COL20A1	0,83
collagen, type XVIII, alpha 1	COL18A1	0,83
RNA binding motif (RNP1, RRM) protein 3	RBM3	0,83
olfactory receptor, family 8, subfamily D, member 1	OR8D1	0,83
zinc finger, MIZ-type containing 1	ZMIZ1	0,83
formin like 3	FMNL3	0,83
natural cytotoxicity triggering receptor 1	NCR1	0,83
long intergenic non-protein coding RNA 273	LINC00273	0,83
SRR1 domain containing	SRRD	0,83
pre-mRNA processing factor 19	PRPF19	0,83
mitogen-activated protein kinase 9	MAPK9	0,83
dynein, light chain, roadblock-type 2	DYNLRB2	0,83
tripartite motif containing 31	TRIM31	0,83
transmembrane protein 123	TMEM123	0,83
pepsinogen 5, group I (pepsinogen A)	PGA5	0,83
basic leucine zipper and W2 domains 2	BZW2	0,83
surfeit 1	SURF1	0,83
U2 small nuclear RNA auxiliary factor 1	U2AF1	0,83
Memczak2013 ANTISENSE, CDS, coding, INTERNAL, intronic best transcript NM_139235	NOL6	0,83

transmembrane protein 168	TMEM168	0,83
activating signal cointegrator 1 complex subunit 3	ASCC3	0,83
carbohydrate (chondroitin 4) sulfotransferase 12	CHST12	0,83
steroid 5 alpha-reductase 3	SRD5A3	0,83
receptor (chemosensory) transporter protein 4	RTP4	0,83
uncharacterized LOC401081; uncharacterized LOC401081 [Source:EntrezGene;Acc:401081]; novel transcript	FLJ22763; RP11-59E19.1	0,83
zinc finger and BTB domain containing 47	ZBTB47	0,83
anti-silencing function 1B histone chaperone	ASF1B	0,83
tudor domain containing 10	TDRD10	0,83
methyltransferase like 15	METTL15	0,83
protease, serine, 8	PRSS8	0,83
follistatin like 1; microRNA 198	FSTL1; MIR198	0,83
galactokinase 2	GALK2	0,83
TRPM8 channel-associated factor 2	TCAF2	0,83
Transcript Identified by AceView, Entrez Gene ID(s) 126231	ZNF573	0,83
surfeit 1	SURF1	0,83
trehalase	TREH	0,83
oxytocin receptor	OXTR	0,83
glycine dehydrogenase (decarboxylating)	GLDC	0,83
family with sequence similarity 186, member A	FAM186A	0,83
APH1A gamma secretase subunit	APH1A	0,83
ALG10, alpha-1,2-glucosyltransferase	ALG10	0,83
Transcript Identified by AceView, Entrez Gene ID(s) 7248	TSC1	0,83
phosphatidylinositol 4-kinase, catalytic, alpha pseudogene 2	PI4KAP2	0,83
RNA binding protein, fox-1 homolog (C. elegans) 3	RBFOX3	0,83
PMS1 homolog 2, mismatch repair system component pseudogene 3	PMS2P3	0,83
HLA complex group 9 (non-protein coding)	HCG9	0,83
proteasome 26S subunit, ATPase 6	PSMC6	0,83
SRC kinase signaling inhibitor 1	SRCIN1	0,83
TAR (HIV-1) RNA binding protein 2	TARBP2	0,83
chromosome 22 open reading frame 34	C22orf34	0,83
proline rich 30	PRR30	0,83
docking protein 4	DOK4	0,83
CoA synthase	COASY	0,83
FUN14 domain containing 2	FUNDC2	0,83
kin of IRRE like 3 (Drosophila)	KIRREL3	0,83
exocyst complex component 3	EXOC3	0,83

uncharacterized protein LOC158434 [Source:RefSeq peptide;Acc:NP_001243337]; uncharacterized LOC158434; Protein LOC158434 [Source:UniProtKB/TrEMBL;Acc:A0A087X204]; novel transcript; Transcript Identified by AceView	RP11-569G13.3; LOC158434; setora	0,83
amyloid P component, serum	APCS	0,83
zinc finger protein 713	ZNF713	0,83
tripartite motif containing 49	TRIM49	0,83
Transcript Identified by AceView, Entrez Gene ID(s) 51011	FAHD2A	0,83
forkhead box S1	FOXS1	0,83
NUT family member 2A	NUTM2A	0,83
THAP domain containing 7	THAP7	0,83
glutathione peroxidase 5	GPX5	0,83
plexin A1	PLXNA1	0,83
solute carrier family 25 (mitochondrial carrier; dicarboxylate transporter), member 10	SLC25A10	0,83
Fas apoptotic inhibitory molecule	FAIM	0,83
neuroblastoma amplified sequence	NBAS	0,83
ovochymase 1	OVCH1	0,83
regulator of G-protein signaling 1	RGS1	0,83
family with sequence similarity 231, member B	FAM231B	0,83
sucrase-isomaltase (alpha-glucosidase)	SI	0,83
late cornified envelope 6A	LCE6A	0,83
eukaryotic translation elongation factor 1 delta (guanine nucleotide exchange protein)	EEF1D	0,83
ubiquitin-conjugating enzyme E2R 2	UBE2R2	0,83
ribonuclease H2, subunit B	RNASEH2B	0,83
gem nuclear organelle associated protein 2	GEMIN2	0,83
guanine nucleotide binding protein (G protein), gamma 13	GNG13	0,83
interferon, alpha 16	IFNA16	0,83
coenzyme Q7 homolog, ubiquinone (yeast)	COQ7	0,83
serine/threonine kinase 31	STK31	0,83
CD1d molecule	CD1D	0,83
Memczak2013 ANTISENSE, CDS, coding, INTERNAL best transcript NM_030821	PLA2G12A	0,83
leucine rich repeat containing 56	LRRC56	0,83
IQ motif and ubiquitin domain containing	IQUB	0,83
neuritin 1	NRN1	0,83
wingless-type MMTV integration site family member 2	WNT2	0,83
chromosome 3 open reading frame 70	C3orf70	0,83
fms-related tyrosine kinase 1	FLT1	0,83
DCC netrin 1 receptor	DCC	0,83
actin-like 6B	ACTL6B	0,83

cystatin E/M	CST6	0,83
solute carrier family 14 (urea transporter), member 1 (Kidd blood group)	SLC14A1	0,83
trafficking protein particle complex 4	TRAPPC4	0,83
carcinoembryonic antigen-related cell adhesion molecule 7	CEACAM7	0,83
UBE2F-SCLY readthrough (NMD candidate)	UBE2F-SCLY	0,83
leukocyte immunoglobulin-like receptor, subfamily B (with TM and ITIM domains), member 1	LILRB1	0,83
natriuretic peptide A	NPPA	0,83
kelch-like family member 11	KLHL11	0,83
adipocyte plasma membrane associated protein	APMAP	0,83
folate hydrolase 1B	FOLH1B	0,83
NSE4 homolog A, SMC5-SMC6 complex component	NSMCE4A	0,83
myosin, heavy chain 15	MYH15	0,83
TEA domain family member 3	TEAD3	0,83
protein tyrosine phosphatase, receptor-type, Z polypeptide 1	PTPRZ1	0,83
Era-like 12S mitochondrial rRNA chaperone 1	ERAL1	0,83
olfactory receptor, family 2, subfamily T, member 10	OR2T10	0,83
apolipoprotein C-I	APOC1	0,83
roundabout guidance receptor 2	ROBO2	0,83
zinc finger protein 2	ZNF2	0,83
chromosome 8 open reading frame 44	C8orf44	0,83
family with sequence similarity 167, member A	FAM167A	0,83
solute carrier family 25 (S-adenosylmethionine carrier), member 26	SLC25A26	0,83
late cornified envelope 5A	LCE5A	0,83
solute carrier family 35, member A5	SLC35A5	0,83
olfactory receptor, family 9, subfamily Q, member 1	OR9Q1	0,83
polymerase (RNA) II (DNA directed) polypeptide M	POLR2M	0,83
phosphoinositide-interacting regulator of transient receptor potential channels	PIRT	0,83
ST6 (alpha-N-acetyl-neuraminyl-2,3-beta-galactosyl-1,3)-N-acetylgalactosaminide alpha-2,6-sialyltransferase 3	ST6GALNAC3	0,83
protocadherin 19	PCDH19	0,83
ubiquitin-conjugating enzyme E2L 6	UBE2L6	0,83
zinc finger protein 287	ZNF287	0,83
contactin 5	CNTN5	0,83
PR domain containing 6	PRDM6	0,83
olfactory receptor, family 1, subfamily E, member 2	OR1E2	0,83
olfactory receptor, family 5, subfamily B, member 12	OR5B12	0,83
carcinoembryonic antigen-related cell adhesion molecule 16	CEACAM16	0,83

CREB binding protein	CREBBP	0,83
AGPAT4 intronic transcript 1	AGPAT4-IT1	0,83
zinc finger and SCAN domain containing 16	ZSCAN16	0,83
keratin associated protein 13-3	KRTAP13-3	0,83
CD8a molecule	CD8A	0,83
RCSD domain containing 1	RCSD1	0,83
glutamate receptor, ionotropic, AMPA 4	GRIA4	0,83
HIG1 hypoxia inducible domain family, member 2A	HIGD2A	0,83
zinc finger and BTB domain containing 49	ZBTB49	0,83
OTU deubiquitinase 6A	OTUD6A	0,83
mannosyl (alpha-1,6-)-glycoprotein beta-1,6-N-acetyl-glucosaminyltransferase, isozyme B	MGAT5B	0,83
oxidoreductase-like domain containing 1	OXLD1	0,83
X-ray repair complementing defective repair in Chinese hamster cells 6	XRCC6	0,83
CKLF-like MARVEL transmembrane domain containing 5	CMTM5	0,83
RAP2A, member of RAS oncogene family	RAP2A	0,83
mucin 22	MUC22	0,83
fumarylacetoacetate hydrolase domain containing 2B	FAHD2B	0,83
ribosomal protein L7a	RPL7A	0,82
zinc finger protein 804B	ZNF804B	0,82
family with sequence similarity 111, member A	FAM111A	0,82
phosphatidylethanolamine-binding protein 4	PEBP4	0,82
olfactory receptor, family 10, subfamily J, member 5	OR10J5	0,82
pleckstrin and Sec7 domain containing 3	PSD3	0,82
ring finger protein 8, E3 ubiquitin protein ligase	RNF8	0,82
interactor of little elongation complex ELL subunit 1	ICE1	0,82
dedicator of cytokinesis 2	DOCK2	0,82
family with sequence similarity 153, member B	FAM153B	0,82
2-5-oligoadenylate synthetase-like	OASL	0,82
CCCTC-binding factor (zinc finger protein)-like	CTCFL	0,82
heme binding protein 1	HEBP1	0,82
aldo-keto reductase family 1, member C1	AKR1C1	0,82
NADH dehydrogenase (ubiquinone) flavoprotein 3, 10kDa	NDUFV3	0,82
KIAA0825	KIAA0825	0,82
secretoglobin, family 1D, member 1	SCGB1D1	0,82
N-acetyltransferase 9 (GCN5-related, putative)	NAT9	0,82
DENN/MADD domain containing 4C	DENND4C	0,82
prostaglandin E receptor 3 (subtype EP3)	PTGER3	0,82
chromosome 7 open reading frame 62	C7orf62	0,82
Transcript Identified by AceView, Entrez Gene ID(s) 51684	SUFU	0,82
membrane associated ring finger 9	MARCH9	0,82
small nuclear ribonucleoprotein, U1 70kDa subunit	SNRNP70	0,82

chondroadherin-like	CHADL	0,82
Transcript Identified by AceView, Entrez Gene ID(s) 79572	ATP13A3	0,82
glycerol-3-phosphate dehydrogenase 1-like	GPD1L	0,82
septin 9	sept-09	0,82
Transcript Identified by AceView, Entrez Gene ID(s) 6502	SKP2	0,82
signal sequence receptor, gamma (translocon-associated protein gamma)	SSR3	0,82
myelin oligodendrocyte glycoprotein	MOG	0,82
formin 1	FMN1	0,82
ankyrin repeat and SOCS box containing 8	ASB8	0,82
spalt-like transcription factor 3	SALL3	0,82
zinc finger and BTB domain containing 16	ZBTB16	0,82
SAM and SH3 domain containing 1	SASH1	0,82
TEN1 CST complex subunit; cyclin-dependent kinase 3; TEN1-CDK3 readthrough (NMD candidate)	TEN1; CDK3; TEN1-CDK3	0,82
TAO kinase 2	TAOK2	0,82
neudesin neurotrophic factor	NENF	0,82
egl-9 family hypoxia-inducible factor 1	EGLN1	0,82
deoxyribonuclease I-like 3	DNASE1L3	0,82
methyltransferase like 14	METTL14	0,82
CTAGE family, member 15	CTAGE15	0,82
lin-7 homolog B (C. elegans)	LIN7B	0,82
cerebellar degeneration related protein 1	CDR1	0,82
deafness, autosomal recessive 59	DFNB59	0,82
tripartite motif containing 7	TRIM7	0,82
Homo sapiens GTP binding protein 6 (putative) (GTPBP6), mRNA.; GTP binding protein 6 (putative) [Source:HGNC Symbol;Acc:HGNC:30189]; Homo sapiens GTP binding protein 6 (putative), mRNA (cDNA clone MGC:1170 IMAGE:3528598), complete cds.; Salzman2013 ANNOTATED, CDS, coding, INTERNAL, OVCODE, OVERLAPTX, OVEXON best transcript NM_012227	GTPBP6	0,82
adhesion molecule with Ig-like domain 3	AMIGO3	0,82
zinc finger protein 589	ZNF589	0,82
poly(ADP-ribose) polymerase family member 10	PARP10	0,82
myosin light chain kinase family member 4	MYLK4	0,82
tumor protein p53	TP53	0,82
uncharacterized LOC101928877; novel transcript; Transcript Identified by AceView, Entrez Gene ID(s) 291	LOC101928877; RP11-386B13.3; SLC25A4	0,82
asparagine synthetase domain containing 1	ASNSD1	0,82
dishevelled segment polarity protein 1; microRNA 6808	DVL1; MIR6808	0,82

zinc finger protein 45	ZNF45	0,82
SERTA domain containing 4	SERTAD4	0,82
SEC31 homolog A, COPII coat complex component	SEC31A	0,82
tripartite motif containing 66	TRIM66	0,82
transmembrane protein 38B	TMEM38B	0,82
zinc finger, DHHC-type containing 23	ZDHHC23	0,82
gem nuclear organelle associated protein 7	GEMIN7	0,82
mitochondrial ribosomal protein L10	MRPL10	0,82
olfactory receptor, family 2, subfamily H, member 2	OR2H2	0,82
solute carrier family 22 (organic cation/carnitine transporter), member 16	SLC22A16	0,82
myosin IIIB	MYO3B	0,82
Jeck2013 ANTISENSE, CDS, coding, INTERNAL, intronic, OVCODE, OVEXON best transcript NM_012335	MYO1F	0,82
methylcrotonoyl-CoA carboxylase 1	MCCC1	0,82
sushi domain containing 3	SUSD3	0,82
solute carrier family 1 (glial high affinity glutamate transporter), member 3	SLC1A3	0,82
S100P binding protein	S100PBP	0,82
zinc finger protein 98	ZNF98	0,82
zinc finger protein 787	ZNF787	0,82
nuclear prelamin A recognition factor	NARF	0,82
NODAL modulator 1	NOMO1	0,82
syntrophin, beta 2 (dystrophin-associated protein A1, 59kDa, basic component 2)	SNTB2	0,82
glucosidase, alpha; acid	GAA	0,82
polyamine modulated factor 1 binding protein 1	PMFBP1	0,82
Memczak2013 ALT_ACCEPTOR, ALT_DONOR, coding, INTERNAL, intronic best transcript NM_203487	PCDH9	0,82
transmembrane and coiled-coil domain family 1	TMCC1	0,82
unc-5 netrin receptor D	UNC5D	0,82
ubiquitin specific peptidase 44	USP44	0,82
calcium and integrin binding family member 2	CIB2	0,82
chromosome 10 open reading frame 126	C10orf126	0,82
MAF1 homolog, negative regulator of RNA polymerase III	MAF1	0,82
phosphatidylinositol glycan anchor biosynthesis class O	PIGO	0,82
hydroxy-delta-5-steroid dehydrogenase, 3 beta- and steroid delta-isomerase 2	HSD3B2	0,82
zinc finger protein 474-like	LOC100505841	0,82
pre-B lymphocyte 1	VPREB1	0,82
speedy/RINGO cell cycle regulator family member E3	SPDYE3	0,82
CASP2 and RIPK1 domain containing adaptor with death domain	CRADD	0,82

Transcript Identified by AceView, Entrez Gene ID(s) 10580	SORBS1	0,82
polypeptide N-acetylgalactosaminyltransferase 15	GALNT15	0,82
Transcript Identified by AceView, Entrez Gene ID(s) 9736	USP34	0,82
Homo sapiens coiled-coil domain containing 101 pseudogene (LOC388242), non-coding RNA.; Homo sapiens SAGA complex associated factor 29 pseudogene (LOC613038), non-coding RNA.; Transcript Identified by AceView, Entrez Gene ID(s) 613038, RefSeq ID(s) NR_002557; Transcript Identified by AceView, Entrez Gene ID(s) 613038; Protein LOC101929849 [Source:UniProtKB/TrEMBL;Acc:H0YHN6]	LOC388242; LOC613038; RP11-347C12.3	0,82
purine-rich element binding protein G	PURG	0,82
endothelin converting enzyme 1	ECE1	0,82
fem-1 homolog b (C. elegans)	FEM1B	0,82
NK3 homeobox 1	NKX3-1	0,82
phosphatidylserine synthase 2	PTDSS2	0,82
IGF like family member 1	IGFL1	0,82
nuclear factor of kappa light polypeptide gene enhancer in B-cells inhibitor, epsilon	NFKBIE	0,82
phosphatidylethanolamine N-methyltransferase	PEMT	0,82
scavenger receptor class B, member 2	SCARB2	0,82
eukaryotic translation initiation factor 3, subunit E	EIF3E	0,82
synaptotagmin binding, cytoplasmic RNA interacting protein	SYNCRIP	0,82
long intergenic non-protein coding RNA 452	LINC00452	0,82
WAP four-disulfide core domain 13	WFDC13	0,82
psoriasis susceptibility 1 candidate 2	PSORS1C2	0,82
surfeit 1	SURF1	0,82
protocadherin beta 7	PCDHB7	0,82
Salzman2013 ALT_ACCEPTOR, ALT_DONOR, coding, INTERNAL, intronic best transcript NM_004540	NCAM2	0,82
testis specific protein, Y-linked 10	TSPY10	0,82
CLPTM1-like	CLPTM1L	0,82
antizyme inhibitor 2	AZIN2	0,82
RAD54 homolog B (S. cerevisiae); fibrinogen silencer binding protein	RAD54B; FSBP	0,82
aldolase B, fructose-bisphosphate	ALDOB	0,82
chloride intracellular channel 5	CLIC5	0,82
rabaptin, RAB GTPase binding effector protein 2	RABEP2	0,82
transketolase	TKT	0,82
Ras-related GTP binding A	RRAGA	0,82

matrin 3; small nucleolar RNA, H/ACA box 74A; small nucleolar RNA host gene 4	MATR3; SNORA74A; SNHG4	0,82
MHC class I polypeptide-related sequence B	MICB	0,82
kelch-like family member 17	KLHL17	0,82
pleckstrin homology domain containing, family S member 1	PLEKHS1	0,82
catsper channel auxiliary subunit beta	CATSPERB	0,82
purinergic receptor P2Y, G-protein coupled, 12	P2RY12	0,82
myeloid/lymphoid or mixed-lineage leukemia; translocated to, 6	MLLT6	0,82
family with sequence similarity 46, member A	FAM46A	0,82
eukaryotic translation initiation factor 4 gamma, 1	EIF4G1	0,82
lysozyme G-like 1	LYG1	0,82
anaphase promoting complex subunit 10	ANAPC10	0,82
protein interacting with cyclin A1	PROCA1	0,82
olfactory receptor, family 2, subfamily L, member 2	OR2L2	0,82
Memczak2013 ANTISENSE, coding, INTERNAL, UTR5 best transcript NM_005583	LYL1	0,82
hes family bHLH transcription factor 3	HES3	0,82
interleukin 18 receptor 1	IL18R1	0,82
fin bud initiation factor homolog (zebrafish)	FIBIN	0,82
uncharacterized LOC100129940; Transcript Identified by AceView; novel transcript, antisense to HMGA2	LOC100129940; flanu; RP11-366L20.2	0,82
ssu-2 homolog (C. elegans)	SSUH2	0,82
Rho GTPase activating protein 15	ARHGAP15	0,82
glycoprotein hormones, alpha polypeptide	CGA	0,82
enhancer of mRNA decapping 4	EDC4	0,82
asparagine-linked glycosylation 1-like 9, pseudogene	ALG1L9P	0,82
adhesion G protein-coupled receptor B2	ADGRB2	0,82
DDB1 and CUL4 associated factor 8-like 1	DCAF8L1	0,82
ets variant 5	ETV5	0,82
small integral membrane protein 17	SMIM17	0,82
protein kinase, cAMP-dependent, catalytic, beta	PRKACB	0,82
Transcript Identified by AceView, Entrez Gene ID(s) 2788	GNG7	0,82
proteasome subunit beta 4	PSMB4	0,82
uncharacterized DKFZp779M0652; Transcript Identified by AceView, Entrez Gene ID(s) 374387, RefSeq ID(s) NR_027134; novel transcript; uncharacterized DKFZp779M0652 [Source:EntrezGene;Acc:374387]	DKFZp779M0652; CTD-2210P24.4	0,82
interleukin 9 receptor	IL9R	0,82

Homo sapiens interleukin 9 receptor (IL9R), transcript variant 1, mRNA.; Homo sapiens interleukin 9 receptor (IL9R), transcript variant 2, mRNA.; interleukin 9 receptor [Source:HGNC Symbol;Acc:HGNC:6030]; Homo sapiens interleukin 9 receptor, mRNA (cDNA clone MGC:59852 IMAGE:6304829), complete cds.; Transcript Identified by AceView, Entrez Gene ID(s) 3581, RefSeq ID(s) NM_002186; Transcript Identified by AceView, Entrez Gene ID(s) 3581	IL9R	0,82
olfactory receptor family 4 subfamily E member 1 (gene/pseudogene) [Source:HGNC Symbol;Acc:HGNC:8296]	OR4E1	0,82
sigma non-opioid intracellular receptor 1	SIGMAR1	0,82
bromodomain PHD finger transcription factor	BPTF	0,82
DENN/MADD domain containing 6A	DENND6A	0,82
hypoxia up-regulated 1	HYOU1	0,82
mitochondrial tRNA translation optimization 1	MTO1	0,82
zinc finger protein 721; ATP binding cassette subfamily A member 11, pseudogene	ZNF721; ABCA11P	0,82
serine peptidase inhibitor, Kazal type 2 (acrosin-trypsin inhibitor)	SPINK2	0,82
mitochondrial ribosomal protein S9	MRPS9	0,82
POU class 5 homeobox 1	POU5F1	0,82
ribosomal protein L7a	RPL7A	0,82
NLR family, CARD domain containing 5	NLRC5	0,82
BUD13 homolog	BUD13	0,82
protein phosphatase 2, regulatory subunit B, beta	PPP2R3B	0,82
protein phosphatase 2, regulatory subunit B, beta	PPP2R3B	0,82
dpy-19-like 1 (C. elegans)	DPY19L1	0,82
protein kinase, AMP-activated, beta 1 non-catalytic subunit	PRKAB1	0,82
deoxyguanosine kinase	DGUOK	0,82
long intergenic non-protein coding RNA 1126	LINC01126	0,82
Myb/SANT-like DNA-binding domain containing 4 with coiled-coils	MSANTD4	0,82
CCZ1 homolog B, vacuolar protein trafficking and biogenesis associated	CCZ1B	0,82
D-tyrosyl-tRNA deacylase 2 (putative)	DTD2	0,82
ADAM metallopeptidase domain 30	ADAM30	0,82
Jeck2013 ALT_ACCEPTOR, ALT_DONOR, coding, INTERNAL, intronic best transcript NM_133459	CCBE1	0,82
lactate dehydrogenase D	LDHD	0,82
neurotrophic tyrosine kinase, receptor, type 3	NTRK3	0,82
zinc finger protein 75D	ZNF75D	0,82
solute carrier family 35, member G1	SLC35G1	0,82

tripartite motif containing 14	TRIM14	0,82
epidermal growth factor receptor pathway substrate 15-like 1	EPS15L1	0,82
colipase-like 1	CLPSL1	0,82
integrator complex subunit 8	INTS8	0,82
olfactory receptor, family 56, subfamily B, member 1	OR56B1	0,82
olfactory receptor, family 51, subfamily F, member 1 (gene/pseudogene)	OR51F1	0,82
chymase 1, mast cell	CMA1	0,82
crystallin beta B2	CRYBB2	0,82
mitochondrial ribosomal protein L43	MRPL43	0,82
ankyrin repeat domain 18B	ANKRD18B	0,82
peptidoglycan recognition protein 2	PGLYRP2	0,82
Transcript Identified by AceView, Entrez Gene ID(s) 26010	SPATS2L	0,82
methylthioribose-1-phosphate isomerase 1	MRI1	0,82
mitogen-activated protein kinase 1	MAPK1	0,82
heparan sulfate (glucosamine) 3-O-sulfotransferase 2	HS3ST2	0,82
melanocortin 4 receptor	MC4R	0,82
ring finger and WD repeat domain 2, E3 ubiquitin protein ligase	RFWD2	0,82
lymphocyte antigen 6 complex, locus E	LY6E	0,82
voltage-dependent anion channel 3	VDAC3	0,82
DEAH (Asp-Glu-Ala-His) box polypeptide 36	DHX36	0,82
defensin, beta 127	DEFB127	0,82
5-nucleotidase, cytosolic IIIA	NT5C3A	0,82
lymphocyte antigen 86	LY86	0,82
Transcript Identified by AceView, Entrez Gene ID(s) 225	ABCD2	0,82
Rho GTPase activating protein 11B	ARHGAP11B	0,82
glutamyl-tRNA synthetase 2, mitochondrial	EARS2	0,82
NADH dehydrogenase (ubiquinone) 1 alpha subcomplex, 3, 9kDa	NDUFA3	0,82
argonaute RISC catalytic component 4	ago-04	0,82
chemokine (C motif) ligand 1	XCL1	0,82
neuroblastoma breakpoint family, member 9	NBPF9	0,82
obscurin, cytoskeletal calmodulin and titin-interacting RhoGEF	OBSCN	0,82
ribosomal protein L15	RPL15	0,81
HCLS1 associated protein X-1	HAX1	0,81
SPATA31 subfamily C, member 1	SPATA31C1	0,81
ribosomal protein L4; small nucleolar RNA, C/D box 16; small nucleolar RNA, C/D box 18A; small nucleolar RNA, C/D box 18B; small nucleolar RNA, C/D box 18C	RPL4; SNORD16; SNORD18A; SNORD18B; SNORD18C	0,81
odd-skipped related transciption factor 1	OSR1	0,81

claudin 16	CLDN16	0,81
solute carrier family 22, member 25	SLC22A25	0,81
olfactory receptor, family 9, subfamily G, member 1; olfactory receptor, family 9, subfamily G, member 9	OR9G1; OR9G9	0,81
membrane protein, palmitoylated 5	MPP5	0,81
sphingosine-1-phosphate receptor 5	S1PR5	0,81
lysine (K)-specific methyltransferase 5A	KMT5A	0,81
c-src tyrosine kinase	CSK	0,81
carbohydrate kinase domain containing	CARKD	0,81
olfactory receptor, family 12, subfamily D, member 3	OR12D3	0,81
SLIT-ROBO Rho GTPase activating protein 2B	SRGAP2B	0,81
ribosomal protein S15	RPS15	0,81
tumor necrosis factor receptor superfamily, member 10c, decoy without an intracellular domain	TNFRSF10C	0,81
solute carrier family 44 (choline transporter), member 2	SLC44A2	0,81
GLI family zinc finger 2	GLI2	0,81
meprin A, alpha (PABA peptide hydrolase)	MEP1A	0,81
F-box and WD repeat domain containing 2	FBXW2	0,81
thyrotropin-releasing hormone	TRH	0,81
ribosomal protein S15a	RPS15A	0,81
PAP associated domain containing 5	PAPD5	0,81
acyl-CoA oxidase 3, pristanoyl	ACOX3	0,81
Homo sapiens CD99 molecule (CD99), transcript variant 2, mRNA.; Homo sapiens CD99 molecule (CD99), transcript variant 3, mRNA.; Homo sapiens CD99 molecule (CD99), transcript variant 1, mRNA.; CD99 molecule [Source:HGNC Symbol;Acc:HGNC:7082]; Homo sapiens CD99 molecule, mRNA (cDNA clone MGC:2282 IMAGE:3161673), complete cds.; Homo sapiens CD99 molecule, mRNA (cDNA clone MGC:3938 IMAGE:2906143), complete cds.; Homo sapiens CD99 molecule, mRNA (cDNA clone MGC:19734 IMAGE:3606974), complete cds.; Homo sapiens CD99 molecule, mRNA (cDNA clone MGC:4214 IMAGE:2957883), complete cds.; Homo sapiens CD99 molecule, mRNA (cDNA clone MGC:14521 IMAGE:4094268), complete cds.; Salzman2013 ANNOTATED, CDS, coding, INTERNAL, OVCODE, OVEXON best transcript NM_002414; Salzman2013 ANNOTATED, CDS, coding, OVCODE, OVEXON, UTR3 best transcript NM_002414	CD99	0,81
RecQ mediated genome instability 2	RMI2	0,81
Zhang2013 ALT_ACCEPTOR, ALT_DONOR, coding, INTERNAL, intronic best transcript NM_198253	TERT	0,81
sperm associated antigen 7	SPAG7	0,81

tenascin R	TNR	0,81
NODAL modulator 3; NODAL modulator 2	NOMO3; NOMO2	0,81
zinc finger protein 91	ZNF91	0,81
splicing factor proline/glutamine-rich	SFPQ	0,81
HtrA serine peptidase 1	HTRA1	0,81
Transcript Identified by AceView, Entrez Gene ID(s) 1803	DPP4	0,81
zinc finger protein 768	ZNF768	0,81
CD2 molecule	CD2	0,81
coiled-coil domain containing 146	CCDC146	0,81
crooked neck pre-mRNA splicing factor 1	CRNKL1	0,81
transmembrane 4 L six family member 18	TM4SF18	0,81
v-maf avian musculoaponeurotic fibrosarcoma oncogene homolog	MAF	0,81
nicotinamide nucleotide adenylyltransferase 2	NMNAT2	0,81
thyroid stimulating hormone receptor	TSHR	0,81
ubiquinol-cytochrome c reductase complex assembly factor 3	UQCC3	0,81
peptidase domain containing associated with muscle regeneration 1	PAMR1	0,81
polyhomeotic homolog 2 (Drosophila); microRNA 3605	PHC2; MIR3605	0,81
Transcript Identified by AceView, Entrez Gene ID(s) 22985	ACIN1	0,81
complement factor H-related 1	CFHR1	0,81
neurotrophin 3	NTF3	0,81
actin, gamma 2, smooth muscle, enteric	ACTG2	0,81
RNA binding motif protein 11	RBM11	0,81
olfactory receptor, family 6, subfamily B, member 1	OR6B1	0,81
UBX domain protein 4	UBXN4	0,81
chemokine (C-X-C motif) ligand 6	CXCL6	0,81
PSMA3 antisense RNA 1	PSMA3-AS1	0,81
oviductal glycoprotein 1	OVGP1	0,81
Transcript Identified by AceView, Entrez Gene ID(s) 285074; 5343	PLGLB1	0,81
clustered mitochondria (cluA/CLU1) homolog	CLUH	0,81
DEXH (Asp-Glu-X-His) box polypeptide 58	DHX58	0,81
endosulfine alpha	ENSA	0,81
family with sequence similarity 110, member A	FAM110A	0,81
olfactory receptor, family 8, subfamily K, member 1	OR8K1	0,81
adaptor-related protein complex 4, epsilon 1 subunit	AP4E1	0,81
serine/arginine repetitive matrix 1	SRRM1	0,81
presenilin associated, rhomboid-like	PARL	0,81
keratin associated protein 4-9	KRTAP4-9	0,81
olfactory receptor, family 5, subfamily P, member 3	OR5P3	0,81
cyclic nucleotide gated channel alpha 2	CNGA2	0,81

ribosomal protein S6 kinase, 70kDa, polypeptide 1	RPS6KB1	0,81
proteasome activator subunit 3	PSME3	0,81
solute carrier family 36 (proton/amino acid symporter), member 2	SLC36A2	0,81
F-box protein 43	FBXO43	0,81
mitochondrial ribosomal protein S24	MRPS24	0,81
Jeck2013 ANTISENSE, INTERNAL, intronic, ncRNA, OVEXON best transcript NR_036569	PILRB	0,81
peroxisomal biogenesis factor 3	PEX3	0,81
5-nucleotidase domain containing 1	NT5DC1	0,81
inositol polyphosphate-5-phosphatase D	INPP5D	0,81
sorting nexin 31	SNX31	0,81
protein phosphatase 2, regulatory subunit B, alpha	PPP2R5A	0,81
MLX interacting protein	MLXIP	0,81
heat shock 27kDa protein 3	HSPB3	0,81
spermatogenesis associated 9	SPATA9	0,81
crystallin gamma C	CRYGC	0,81
zinc finger protein 541	ZNF541	0,81
signal peptide peptidase like 2A	SPPL2A	0,81
uncharacterized LOC651430; uncharacterized LOC651430 [Source:EntrezGene;Acc:651430]; novel transcript	FLJ38576; RP11-173M11.6	0,81
Memczak2013 ANTISENSE, coding, INTERNAL, intronic best transcript NM_014117	C16orf72	0,81
aquarius intron-binding spliceosomal factor	AQR	0,81
Jeck2013 ALT_ACCEPTOR, ALT_DONOR, coding, INTERNAL, intronic best transcript NM_004249	RAB28	0,81
cytochrome P450, family 11, subfamily B, polypeptide 2	CYP11B2	0,81
ST8 alpha-N-acetyl-neuraminide alpha-2,8-sialyltransferase 1	ST8SIA1	0,81
forkhead box P2	FOXP2	0,81
cytochrome P450, family 2, subfamily J, polypeptide 2	CYP2J2	0,81
oocyte expressed protein	OOEP	0,81
chromosome 12 open reading frame 74	C12orf74	0,81
POU class 3 homeobox 4	POU3F4	0,81
ribosomal RNA processing 7 homolog A	RRP7A	0,81
potassium channel subfamily M regulatory beta subunit 1	KCNMB1	0,81
thioredoxin-related transmembrane protein 4	TMX4	0,81
Usher syndrome 1C binding protein 1	USHBP1	0,81
ribosomal protein L7	RPL7	0,81
guanine nucleotide binding protein (G protein), gamma 2	GNG2	0,81
dihydrofolate reductase	DHFR	0,81
forkhead box J1	FOXJ1	0,81

leucine aminopeptidase 3	LAP3	0,81
dendrocyte expressed seven transmembrane protein	DCSTAMP	0,81
resistin like beta	RETNLB	0,81
pelota homolog (Drosophila); integrin alpha 1	PELO; ITGA1	0,81
echinoderm microtubule associated protein like 5	EML5	0,81
Memczak2013 ANTISENSE, coding, INTERNAL, UTR3 best transcript NM_032434	ZNF512	0,81
ribosomal protein L22	RPL22	0,81
C-type lectin domain family 18, member A	CLEC18A	0,81
GTPase activating protein and VPS9 domains 1	GAPVD1	0,81
solute carrier family 12 (potassium/chloride transporter), member 4	SLC12A4	0,81
POU class 2 homeobox 1	POU2F1	0,81
Transcript Identified by AceView, Entrez Gene ID(s) 23175	LPIN1	0,81
small ubiquitin-like modifier 2	SUMO2	0,81
family with sequence similarity 216, member A	FAM216A	0,81
uroporphyrinogen III synthase	UROS	0,81
cornichon family AMPA receptor auxiliary protein 4	CNIH4	0,81
POP5 homolog, ribonuclease P/MRP subunit	POP5	0,81
unc-5 netrin receptor B	UNC5B	0,81
NADH dehydrogenase (ubiquinone) Fe-S protein 1, 75kDa (NADH-coenzyme Q reductase); glycine cleavage system protein H (aminomethyl carrier) pseudogene 3	NDUFS1; GCSHP3	0,81
mediator complex subunit 20	MED20	0,81
hypoxia up-regulated 1	HYOU1	0,81
zinc finger protein 720	ZNF720	0,81
REX4 homolog, 3'-5' exonuclease [Source:HGNC Symbol;Acc:HGNC:12820]	REXO4	0,81
chromosome 7 open reading frame 65	C7orf65	0,81
defensin, beta 105A	DEFB105A	0,81
inositol polyphosphate-5-phosphatase D	INPP5D	0,81
Meis homeobox 1	MEIS1	0,81
family with sequence similarity 9, member A	FAM9A	0,81
protease, serine 50	PRSS50	0,81
regulatory factor X, 2 (influences HLA class II expression)	RFX2	0,81
polymerase (RNA) II (DNA directed) polypeptide J4, pseudogene	POLR2J4	0,81
BRCA1-associated ATM activator 1	BRAT1	0,81
dolichyl-phosphate mannosyltransferase polypeptide 1, catalytic subunit	DPM1	0,81
roundabout guidance receptor 1	ROBO1	0,81
Jeck2013 ALT_ACCEPTOR, ALT_DONOR, coding, INTERNAL, intronic best transcript NM_017774	CDKAL1	0,81

plastin 1	PLS1	0,81
interleukin 1 receptor associated kinase 1; microRNA 718	IRAK1; MIR718	0,81
MTOR associated protein, LST8 homolog	MLST8	0,81
SH2 domain containing 3C	SH2D3C	0,81
POU class 4 homeobox 2	POU4F2	0,81
chromosome 4 open reading frame 50	C4orf50	0,81
Bardet-Biedl syndrome 9	BBS9	0,81
zinc finger protein 470	ZNF470	0,81
kallikrein related peptidase 3	KLK3	0,81
dishevelled segment polarity protein 3	DVL3	0,81
Memczak2013 ANTISENSE, CDS, coding, INTERNAL best transcript NM_014931	PPP6R1	0,81
NYN domain and retroviral integrase containing	NYNRIN	0,81
ermin	ERMN	0,81
RAN, member RAS oncogene family	RAN	0,81
cytochrome b561 family, member D2	CYB561D2	0,81
heterogeneous nuclear ribonucleoprotein D	HNRNPD	0,81
proline rich Gla (G-carboxyglutamic acid) 3 (transmembrane)	PRRG3	0,81
tafazzin	TAZ	0,81
family with sequence similarity 216, member B	FAM216B	0,81
leucine rich repeat containing 45	LRRC45	0,81
carbonic anhydrase XIII	CA13	0,81
ATP/GTP binding protein-like 1	AGBL1	0,81
collagen, type VI, alpha 2	COL6A2	0,81
ectonucleotide pyrophosphatase/phosphodiesterase 5 (putative)	ENPP5	0,81
Transcript Identified by AceView, Entrez Gene ID(s) 3775	KCNK1	0,81
methyl-CpG binding domain protein 6	MBD6	0,81
olfactory receptor, family 8, subfamily K, member 5	OR8K5	0,81
NADPH oxidase activator 1	NOXA1	0,81
dihydropyrimidinase-like 5	DPYSL5	0,81
chromosome 16 open reading frame 59	C16orf59	0,81
glyoxalase I	GLO1	0,81
chromosome 15 open reading frame 43	C15orf43	0,81
secretogranin III	SCG3	0,81
hyaluronan binding protein 4	HABP4	0,81
transcription elongation factor B polypeptide 3B (elongin A2)	TCEB3B	0,81
Memczak2013 ANTISENSE, CDS, coding, INTERNAL best transcript NM_025268	TMEM121	0,81
maestro heat-like repeat family member 6	MROH6	0,81
ribosomal protein S25	RPS25	0,81

Transcript Identified by AceView, Entrez Gene ID(s) 1768	DNAH6	0,81
small nucleolar RNA, C/D box 49A; small nucleolar RNA, C/D box 65; LRRC75A antisense RNA 1	SNORD49A; SNORD65; LRRC75A-AS1	0,81
chromosome 14 open reading frame 169	C14orf169	0,81
transmembrane BAX inhibitor motif containing 4	TMBIM4	0,81
WD repeat and SOCS box containing 1	WSB1	0,81
ring finger protein 41, E3 ubiquitin protein ligase	RNF41	0,81
forkhead box N2	FOXN2	0,81
double homeobox 5	DUX5	0,81
ATPase, H+ transporting, lysosomal 70kDa, V1 subunit A	ATP6V1A	0,81
zinc finger, RAN-binding domain containing 2	ZRANB2	0,81
potassium channel tetramerization domain containing 2	KCTD2	0,81
NK6 homeobox 1	NKX6-1	0,81
tubulin tyrosine ligase-like family member 2	TTLL2	0,81
complement component 5a receptor 1	C5AR1	0,81
UDP-Gal:betaGlcNAc beta 1,4- galactosyltransferase, polypeptide 3	B4GALT3	0,81
ubiquitin specific peptidase 28	USP28	0,81
family with sequence similarity 153, member A	FAM153A	0,81
retrotransposon gag domain containing 1	RGAG1	0,81
GDP-mannose pyrophosphorylase B	GMPPB	0,81
heterogeneous nuclear ribonucleoprotein C-like 4	HNRNPCL4	0,81
hydroxymethylbilane synthase	HMBS	0,81
USO1 vesicle transport factor	USO1	0,81
coenzyme Q5, methyltransferase	COQ5	0,81
leucine rich repeat containing 30	LRRC30	0,81
CUB and zona pellucida-like domains 1	CUZD1	0,81
Memczak2013 ALT_ACCEPTOR, ALT_DONOR, coding, INTERNAL, intronic best transcript NM_020896	OSBPL5	0,81
integrin alpha 11	ITGA11	0,81
ribosomal protein S24	RPS24	0,81
cilia and flagella associated protein 74	CFAP74	0,81
far upstream element (FUSE) binding protein 1	FUBP1	0,81
diacylglycerol kinase, delta 130kDa	DGKD	0,81
family with sequence similarity 133, member A pseudogene	LOC100421561	0,81
melanocortin 2 receptor (adrenocorticotropic hormone)	MC2R	0,81
proteasome (prosome, macropain) assembly chaperone 4	PSMG4	0,81
zinc finger protein 75a	ZNF75A	0,81
gremlin 1, DAN family BMP antagonist [Source:HGNC Symbol;Acc:HGNC:2001]	GREM1	0,81

hyperpolarization activated cyclic nucleotide gated potassium channel 2	HCN2	0,81
DnaJ (Hsp40) homolog, subfamily C, member 21	DNAJC21	0,81
AT rich interactive domain 1A (SWI-like)	ARID1A	0,81
coiled-coil-helix-coiled-coil-helix domain containing 4	CHCHD4	0,81
chromosome 11 open reading frame 87	C11orf87	0,81
calcium channel, voltage-dependent, T type, alpha 1G subunit	CACNA1G	0,81
solute carrier family 25 (glutamate carrier), member 18	SLC25A18	0,81
lipase, hormone-sensitive	LIPE	0,80
corticotropin releasing hormone	CRH	0,80
Transcript Identified by AceView, Entrez Gene ID(s) 153339	TMEM167A	0,80
TPX2, microtubule-associated	TPX2	0,80
chromosome 19 open reading frame 24	C19orf24	0,80
SH3-domain GRB2-like 3	SH3GL3	0,80
Jeck2013 ALT_DONOR, coding, INTERNAL, intronic best transcript NM_006845	KIF2C	0,80
centromere protein I	CENPI	0,80
G protein-coupled receptor kinase 5	GRK5	0,80
leucine rich repeat containing 59	LRRC59	0,80
autophagy related 5	ATG5	0,80
chromosome 2 open reading frame 47	C2orf47	0,80
KIAA1024	KIAA1024	0,80
mitochondrial ribosomal protein L54	MRPL54	0,80
testis development related protein	TDRP	0,80
WDFY family member 4	WDFY4	0,80
COP9 signalosome subunit 2	COPS2	0,80
ribosomal protein L11	RPL11	0,80
transcription factor 24	TCF24	0,80
major histocompatibility complex, class II, DP alpha 1	HLA-DPA1	0,80
centrosomal protein 192kDa	CEP192	0,80
cysteine rich hydrophobic domain 1	CHIC1	0,80
methylenetetrahydrofolate dehydrogenase (NADP+ dependent) 2-like	MTHFD2L	0,80
coiled-coil domain containing 190	CCDC190	0,80
protocadherin 10	PCDH10	0,80
splicing factor 3b subunit 2	SF3B2	0,80
N-deacetylase/N-sulfotransferase (heparan glucosaminyl) 4	NDST4	0,80
RUN and FYVE domain containing 3	RUFY3	0,80
Transcript Identified by AceView, Entrez Gene ID(s) 2890	GRIA1	0,80
chromosome 4 open reading frame 26	C4orf26	0,80

Transcript Identified by AceView, Entrez Gene ID(s) 5747	PTK2	0,80
phosphorylase kinase, beta	PHKB	0,80
slit guidance ligand 2	SLIT2	0,80
olfactory receptor, family 2, subfamily AP, member 1	OR2AP1	0,80
zinc finger protein 35	ZNF35	0,80
GDNF family receptor alpha 3	GFRA3	0,80
dishevelled-binding antagonist of beta-catenin 1	DACT1	0,80
serine/arginine-rich splicing factor 10	SRSF10	0,80
protein phosphatase 1, regulatory subunit 26	PPP1R26	0,80
ribosomal protein L17; small nucleolar RNA, C/D box 58A; small nucleolar RNA, C/D box 58B; small nucleolar RNA, C/D box 58C	RPL17; SNORD58A; SNORD58B; SNORD58C	0,80
CCAAT/enhancer binding protein (C/EBP), delta	CEBPD	0,80
l(3)mbt-like 4 (Drosophila)	L3MBTL4	0,80
isoprenylcysteine carboxyl methyltransferase	ICMT	0,80
keratin associated protein 5-7	KRTAP5-7	0,80
boule homolog, RNA binding protein	BOLL	0,80
Transcript Identified by AceView, Entrez Gene ID(s) 90338	ZNF160	0,80
flavin containing monooxygenase 4	FMO4	0,80
Rh-associated glycoprotein	RHAG	0,80
docking protein 7	DOK7	0,80
PARK2 co-regulated	PACRG	0,80
SWI/SNF related, matrix associated, actin dependent regulator of chromatin, subfamily c, member 1	SMARCC1	0,80
deleted in lymphocytic leukemia 2 (non-protein coding); microRNA 15a; microRNA 16-1; microRNA 3613	DLEU2; MIR15A; MIR16-1; MIR3613	0,80
synaptosome associated protein 25kDa	SNAP25	0,80
odd-skipped related transciption factor 2	OSR2	0,80
C-x(9)-C motif containing 1	CMC1	0,80
prostaglandin D2 synthase 21kDa (brain)	PTGDS	0,80
MNAT CDK-activating kinase assembly factor 1	MNAT1	0,80
late endosomal/lysosomal adaptor, MAPK and MTOR activator 2	LAMTOR2	0,80
junctional sarcoplasmic reticulum protein 1	JSRP1	0,80
chromosome 12 open reading frame 42	C12orf42	0,80
tachykinin receptor 1	TACR1	0,80
leucine rich repeat protein 1; ras homolog family member Q pseudogene 1	LRR1; RHOQP1	0,80
SVOP-like	SVOPL	0,80
Memczak2013 ANTISENSE, CDS, coding, INTERNAL, intronic best transcript NM_178860	SEZ6	0,80

carnosine dipeptidase 1 (metallopeptidase M20 family)	CNDP1	0,80
zinc finger with KRAB and SCAN domains 7	ZKSCAN7	0,80
zinc finger protein 780B	ZNF780B	0,80
phospholipase C, beta 3 (phosphatidylinositol-specific)	PLCB3	0,80
relaxin/insulin-like family peptide receptor 4	RXFP4	0,80
regenerating islet-derived 1 alpha	REG1A	0,80
proteoglycan 3	PRG3	0,80
5-nucleotidase, cytosolic IIIB	NT5C3B	0,80
fibronectin type III domain containing 9	FNDC9	0,80
transmembrane phosphatase with tensin homology	TPTE	0,80
sorting nexin 10	SNX10	0,80
phospholipase C-like 1	PLCL1	0,80
NADH dehydrogenase (ubiquinone) 1 alpha subcomplex, 6, 14kDa	NDUFA6	0,80
kinesin family member 2B	KIF2B	0,80
receptor-interacting serine-threonine kinase 3	RIPK3	0,80
SEC23 interacting protein	SEC23IP	0,80
vasohibin 1	VASH1	0,80
hypoxia inducible lipid droplet-associated	HILPDA	0,80
Transcript Identified by AceView, Entrez Gene ID(s) 1767	DNAH5	0,80
Transcript Identified by AceView, Entrez Gene ID(s) 55040	EPN3	0,80
major facilitator superfamily domain containing 13A	MFSD13A	0,80
Salzman2013 ANNOTATED, coding, OVEXON, UTR5 best transcript NM_001005862	ERBB2	0,80
neurofibromin 2 (merlin)	NF2	0,80
dynein, axonemal, heavy chain 14	DNAH14	0,80
wingless-type MMTV integration site family, member 8A	WNT8A	0,80
wingless-type MMTV integration site family, member 3A	WNT3A	0,80
cell division cycle and apoptosis regulator 1; small nucleolar RNA, C/D box 98	CCAR1; SNORD98	0,80
transmembrane protein 229B	TMEM229B	0,80
chondroitin sulfate synthase 1	CHSY1	0,80
lipoma HMGIC fusion partner-like 3	LHFPL3	0,80
tumor necrosis factor receptor superfamily, member 13B	TNFRSF13B	0,80
olfactory receptor, family 51, subfamily M, member 1	OR51M1	0,80
cyclin-dependent kinase 10	CDK10	0,80
Memczak2013 ALT_ACCEPTOR, ALT_DONOR, coding, INTERNAL, intronic best transcript NM_020820	PREX1	0,80
F-box protein 46	FBXO46	0,80
arginyl aminopeptidase (aminopeptidase B)-like 1	RNPEPL1	0,80

transcription elongation regulator 1-like	TCERG1L	0,80
immunity-related GTPase family, cinema	IRGC	0,80
protease, serine 53	PRSS53	0,80
heterogeneous nuclear ribonucleoprotein K; microRNA 7-1	HNRNPK; MIR7-1	0,80
dual specificity phosphatase 15	DUSP15	0,80
olfactory receptor, family 10, subfamily W, member 1	OR10W1	0,80
testis, prostate and placenta expressed	TEPP	0,80
SET domain containing 9	SETD9	0,80
zinc finger, DBF-type containing 2	ZDBF2	0,80
potassium channel, voltage gated shaker related subfamily A, member 10	KCNA10	0,80
IMP (inosine 5-monophosphate) dehydrogenase 2	IMPDH2	0,80
olfactory receptor, family 10, subfamily G, member 9	OR10G9	0,80
transmembrane protein 191B	TMEM191B	0,80
olfactory receptor, family 4, subfamily B, member 1	OR4B1	0,80
cholinergic receptor, nicotinic alpha 2	CHRNA2	0,80
Homo sapiens uncharacterized LOC102724238 (LOC102724238), long non-coding RNA.; Homo sapiens uncharacterized LOC554249 (LOC554249), long non-coding RNA.; Homo sapiens hypothetical LOC554249, mRNA (cDNA clone MGC:99799 IMAGE:6378300), complete cds.; novel transcript	LOC102724238; LOC554249; RP11-111F5.4	0,80
gelsolin	GSN	0,80
1-aminocyclopropane-1-carboxylate synthase homolog (inactive)	ACCS	0,80
eukaryotic translation elongation factor 1 alpha 1 pseudogene 5	EEF1A1P5	0,80
zinc finger protein 655	ZNF655	0,80
glutathione S-transferase theta 2 (gene/pseudogene); glutathione S-transferase theta 2B (gene/pseudogene)	GSTT2; GSTT2B	0,80
runt-related transcription factor 1; translocated to, 1 (cyclin D-related)	RUNX1T1	0,80
GABA(A) receptor-associated protein like 2	GABARAPL2	0,80
olfactory receptor, family 6, subfamily C, member 74	OR6C74	0,80
Thy-1 cell surface antigen	THY1	0,80
long intergenic non-protein coding RNA 663	LINC00663	0,80
MAM domain containing 4	MAMDC4	0,80
cyclin I family, member 2	CCNI2	0,80
prostaglandin E synthase 2	PTGES2	0,80
cytochrome c oxidase subunit VIIc; microRNA 3607	COX7C; MIR3607	0,80
paraneoplastic Ma antigen 2	PNMA2	0,80
Sec61 translocon gamma subunit	SEC61G	0,80
solute carrier family 16, member 13	SLC16A13	0,80
YY1 associated factor 2	YAF2	0,80

YEATS domain containing 2	YEATS2	0,80
cytochrome P450, family 11, subfamily B, polypeptide 1	CYP11B1	0,80
dishevelled segment polarity protein 2	DVL2	0,80
ring finger protein 139	RNF139	0,80
prodynorphin	PDYN	0,80
protein tyrosine phosphatase, receptor type, U	PTPRU	0,80
ubiquitin-conjugating enzyme E2S	UBE2S	0,80
caspase recruitment domain family, member 18	CARD18	0,80
phosphoglucomutase 5	PGM5	0,80
armadillo repeat containing, X-linked 4	ARMCX4	0,80
NADH dehydrogenase (ubiquinone) Fe-S protein 3, 30kDa (NADH-coenzyme Q reductase)	NDUFS3	0,80
fibroblast growth factor 11	FGF11	0,80
translocase of outer mitochondrial membrane 40 homolog (yeast)	TOMM40	0,80
tumor necrosis factor, alpha-induced protein 3	TNFAIP3	0,80
carboxypeptidase Z; G protein-coupled receptor 78	CPZ; GPR78	0,80
ubiquinol-cytochrome c reductase, complex III subunit X	UQCR10	0,80
protein tyrosine phosphatase, non-receptor type 2	PTPN2	0,80
KH domain containing, RNA binding, signal transduction associated 3	KHDRBS3	0,80
CUGBP, Elav-like family member 2	CELF2	0,80
zinc finger, BED-type containing 5	ZBED5	0,80
C-type lectin domain family 11, member A	CLEC11A	0,80
nuclear assembly factor 1 ribonucleoprotein	NAF1	0,80
hydroxycarboxylic acid receptor 3	HCAR3	0,80
olfactory receptor, family 10, subfamily AC, member 1 (gene/pseudogene) [Source:HGNC Symbol;Acc:HGNC:14758]	OR10AC1	0,80
optic atrophy 3 (autosomal recessive, with chorea and spastic paraplegia)	OPA3	0,80
advanced glycosylation end product-specific receptor	AGER	0,80
uridine monophosphate synthetase	UMPS	0,80
F-box and WD repeat domain containing 8	FBXW8	0,80
RNA binding protein, fox-1 homolog (C. elegans) 1	RBFOX1	0,80
cyclin-dependent kinase-like 4	CDKL4	0,80
B-cell CLL/lymphoma 7B	BCL7B	0,80
chromosome 18 open reading frame 42	C18orf42	0,80
cell division cycle 40	CDC40	0,80
UTP3, small subunit (SSU) processome component, homolog (S. cerevisiae)	UTP3	0,80
polymerase (DNA directed), alpha 1, catalytic subunit	POLA1	0,80
frizzled class receptor 3	FZD3	0,80
dematin actin binding protein	DMTN	0,80
heat shock 70kDa protein 14	HSPA14	0,80

DEAD (Asp-Glu-Ala-Asp) box helicase 17	DDX17	0,80
dimethylglycine dehydrogenase	DMGDH	0,80
eukaryotic translation initiation factor 4H	EIF4H	0,80
heterogeneous nuclear ribonucleoprotein M	HNRNPM	0,80
RNA binding motif protein 17	RBM17	0,80
Memczak2013 ANTISENSE, coding, INTERNAL, intronic best transcript NM_152756	RICTOR	0,80
GPN-loop GTPase 3	GPN3	0,80
checkpoint kinase 1	CHEK1	0,80
transmembrane 9 superfamily member 2	TM9SF2	0,80
mahogunin ring finger 1, E3 ubiquitin protein ligase	MGRN1	0,80
Transcript Identified by AceView, Entrez Gene ID(s) 123355	LRRC28	0,80
TM2 domain containing 2	TM2D2	0,80
telomerase reverse transcriptase	TERT	0,80
ribosomal protein S12	RPS12	0,80
TMEM147 antisense RNA 1	TMEM147-AS1	0,80
small integral membrane protein 11A	SMIM11A	0,80
calcium binding protein 7	CABP7	0,80
olfactory receptor, family 7, subfamily A, member 17	OR7A17	0,80
kelch-like family member 8	KLHL8	0,80
ectonucleoside triphosphate diphosphohydrolase 2	ENTPD2	0,80
small proline-rich protein 4	SPRR4	0,80
signal peptidase complex subunit 3	SPCS3	0,80
RAD54-like 2 (S. cerevisiae)	RAD54L2	0,80
ATP synthase, H+ transporting, mitochondrial Fo complex subunit G2	ATP5L2	0,80
serine/threonine kinase-like domain containing 1 [Source:HGNC Symbol;Acc:HGNC:28669]	STKLD1	0,80
MANSC domain containing 1	MANSC1	0,80
cation channel, sperm associated 2	CATSPER2	0,80
tumor protein p63 regulated 1	TPRG1	0,80
peptidyl arginine deiminase, type I	PADI1	0,79
suppressor of tumorigenicity 20	ST20	0,79
Memczak2013 ANTISENSE, coding, INTERNAL, intronic best transcript NM_152996	ST6GALNAC3	0,79
nitric oxide synthase 2, inducible	NOS2	0,79
MOK protein kinase	MOK	0,79
translocase of inner mitochondrial membrane 17 homolog A (yeast)	TIMM17A	0,79
taxilin gamma pseudogene, Y-linked	TXLNGY	0,79
olfactory receptor, family 8, subfamily J, member 2 (gene/pseudogene)	OR8J2	0,79
TAM41 mitochondrial translocator assembly and maintenance homolog	TAMM41	0,79

phospholamban	PLN	0,79
ribosomal protein L9	RPL9	0,79
ADAM metallopeptidase with thrombospondin type 1 motif 19	ADAMTS19	0,79
complement component 1, q subcomponent binding protein	C1QBP	0,79
zinc finger and BTB domain containing 22	ZBTB22	0,79
splicing factor 3b subunit 3	SF3B3	0,79
trinucleotide repeat containing 6C	TNRC6C	0,79
glycogen synthase 1 (muscle)	GYS1	0,79
NADH dehydrogenase (ubiquinone) 1 alpha subcomplex, 10, 42kDa	NDUFA10	0,79
ZNF436 antisense RNA 1	ZNF436-AS1	0,79
CCZ1 homolog, vacuolar protein trafficking and biogenesis associated; CCZ1 homolog B, vacuolar protein trafficking and biogenesis associated	CCZ1; CCZ1B	0,79
EWS RNA binding protein 1	EWSR1	0,79
long intergenic non-protein coding RNA 1521	LINC01521	0,79
euchromatic histone-lysine N-methyltransferase 1	EHMT1	0,79
chromosome 17 open reading frame 58	C17orf58	0,79
PRAME family member 7; PRAME family member 8	PRAMEF7; PRAMEF8	0,79
polymerase (RNA) III (DNA directed) polypeptide A, 155kDa	POLR3A	0,79
sodium channel, voltage gated, type XI alpha subunit	SCN11A	0,79
keratin associated protein 9-1	KRTAP9-1	0,79
inositol polyphosphate-5-phosphatase D	INPP5D	0,79
phosphatidylinositol glycan anchor biosynthesis class S	PIGS	0,79
chitinase 1 (chitotriosidase)	CHIT1	0,79
cytochrome c oxidase assembly factor 3	COA3	0,79
signal peptide, CUB domain, EGF-like 3	SCUBE3	0,79
ceramide synthase 3	CERS3	0,79
tubulin, gamma complex associated protein 3	TUBGCP3	0,79
G protein-coupled receptor 173	GPR173	0,79
maturin, neural progenitor differentiation regulator homolog (Xenopus)	MTURN	0,79
family with sequence similarity 84, member B	FAM84B	0,79
SRY box 18	SOX18	0,79
NK6 homeobox 2	NKX6-2	0,79
regulator of G-protein signaling 16	RGS16	0,79
chromosome 11 open reading frame 58	C11orf58	0,79
caspase recruitment domain family, member 10	CARD10	0,79
olfactory receptor, family 10, subfamily K, member 1	OR10K1	0,79
chromosome 3 open reading frame 20	C3orf20	0,79
hydroxymethylbilane synthase	HMBS	0,79

PTPN13-like, Y-linked 2; PTPN13-like, Y-linked	PRY2; PRY	0,79
stomatin (EPB72)-like 2	STOML2	0,79
protein-kinase, interferon-inducible double stranded RNA dependent inhibitor, repressor of (P58 repressor)	PRKRIR	0,79
sphingomyelin phosphodiesterase 2, neutral membrane (neutral sphingomyelinase)	SMPD2	0,79
CD101 molecule	CD101	0,79
ADP-ribosylation factor 4	ARF4	0,79
F-box and WD repeat domain containing 11	FBXW11	0,79
ring finger protein 214	RNF214	0,79
glycoprotein A33 (transmembrane)	GPA33	0,79
tubulin, gamma complex associated protein 5	TUBGCP5	0,79
protease, serine 35	PRSS35	0,79
doublecortin-like kinase 2	DCLK2	0,79
zinc finger CCCH-type, antiviral 1	ZC3HAV1	0,79
arylsulfatase family, member J	ARSJ	0,79
Memczak2013 ANTISENSE, coding, INTERNAL, UTR3 best transcript NM_175920	LNPEP	0,79
angio-associated migratory cell protein	AAMP	0,79
forkhead box D4-like 1	FOXD4L1	0,79
Memczak2013 ALT_ACCEPTOR, ALT_DONOR, coding, INTERNAL, intronic best transcript NM_001204883	RAB43	0,79
olfactory receptor, family 5, subfamily B, member 3	OR5B3	0,79
phosphatidylinositol 4-kinase, catalytic, alpha pseudogene 1	PI4KAP1	0,79
cysteine-rich secretory protein 1	CRISP1	0,79
protease, serine, 21 (testisin)	PRSS21	0,79
Memczak2013 ALT_ACCEPTOR, ALT_DONOR, coding, INTERNAL, intronic best transcript NM_001006634	ARHGAP17	0,79
tumor protein p53 inducible protein 13	TP53I13	0,79
CCNT2 antisense RNA 1	CCNT2-AS1	0,79
HAUS augmin like complex subunit 5	HAUS5	0,79
adaptor-related protein complex 5, sigma 1 subunit	AP5S1	0,79
RAD21 cohesin complex component like 1	RAD21L1	0,79
NOP56 ribonucleoprotein; small nucleolar RNA, C/D box 57; small nucleolar RNA, C/D box 56; small nucleolar RNA, H/ACA box 51; small nucleolar RNA, C/D box 110; small nucleolar RNA, C/D box 86; microRNA 1292	NOP56; SNORD57; SNORD56; SNORA51; SNORD110; SNORD86; MIR1292	0,79
chromosome 14 open reading frame 93	C14orf93	0,79
insulin-like 5	INSL5	0,79
G-patch domain containing 8	GPATCH8	0,79
protein phosphatase 1, regulatory subunit 3A	PPP1R3A	0,79

Zhang2013 ALT_ACCEPTOR, ALT_DONOR, coding, INTERNAL, intronic best transcript NM_018198	DNAJC11	0,79
inositol polyphosphate-4-phosphatase type II B	INPP4B	0,79
necdin-like 2, SMC5-SMC6 complex component	NDNL2	0,79
DMRT-like family C2	DMRTC2	0,79
protein phosphatase 4, regulatory subunit 3A	PPP4R3A	0,79
chromosome 16 open reading frame 95	C16orf95	0,79
PYD and CARD domain containing	PYCARD	0,79
zinc finger protein 37A	ZNF37A	0,79
olfactory receptor, family 56, subfamily A, member 5	OR56A5	0,79
chondroitin sulfate N-acetylgalactosaminyltransferase 1	CSGALNACT1	0,79
POC1 centriolar protein A	POC1A	0,79
15 kDa selenoprotein; Salzman2013 ANNOTATED, CDS, coding, INTERNAL, OVCODE, OVEXON best transcript NM_004261; Transcript Identified by AceView, Entrez Gene ID(s) 9403, RefSeq ID(s) NM_203341; Transcript Identified by AceView, Entrez Gene ID(s) 9403, RefSeq ID(s) NM_004261; 15 kDa selenoprotein [Source:EntrezGene;Acc:9403]	sept-15	0,79
KRI1 homolog	KRI1	0,79
patatin-like phospholipase domain containing 4	PNPLA4	0,79
cleavage and polyadenylation specific factor 4	CPSF4	0,79
fibroblast growth factor binding protein 1	FGFBP1	0,79
proliferating cell nuclear antigen	PCNA	0,79
solute carrier family 2 (facilitated glucose transporter), member 6 [Source:HGNC Symbol;Acc:HGNC:11011]	SLC2A6	0,79
solute carrier family 25 (mitochondrial oxoadipate carrier), member 21	SLC25A21	0,79
regulator of chromosome condensation (RCC1) and BTB (POZ) domain containing protein 2	RCBTB2	0,79
out at first homolog	OAF	0,79
golgi transport 1B	GOLT1B	0,79
lipase, family member N	LIPN	0,79
killin, p53-regulated DNA replication inhibitor	KLLN	0,79
carbohydrate (N-acetylglucosamine 6-O) sulfotransferase 6	CHST6	0,79
zinc finger protein 517	ZNF517	0,79
major histocompatibility complex, class I, V (pseudogene)	HLA-V	0,79
WD repeat domain 61	WDR61	0,79
eukaryotic translation initiation factor 2B, subunit 4 delta, 67kDa	EIF2B4	0,79
DEAH (Asp-Glu-Ala-His) box helicase 30	DHX30	0,79
anoctamin 6	ANO6	0,79
ribosomal protein L7a	RPL7A	0,79

olfactory receptor, family 2, subfamily T, member 8	OR2T8	0,79
endothelin converting enzyme-like 1	ECEL1	0,79
glucuronidase, beta	GUSB	0,79
molybdenum cofactor sulfurase	MOCOS	0,79
G protein-coupled receptor 22	GPR22	0,79
pre-mRNA processing factor 39	PRPF39	0,79
olfactory receptor, family 4, subfamily F, member 3	OR4F3	0,79
double zinc ribbon and ankyrin repeat domains 1	DZANK1	0,79
Transcript Identified by AceView, Entrez Gene ID(s) 91694	LONRF1	0,79
thyroid hormone receptor interactor 10	TRIP10	0,79
OTU deubiquitinase 7A	OTUD7A	0,79
galactosidase beta 1; transmembrane protein with metallophosphoesterase domain	GLB1; TMPPE	0,79
WNK lysine deficient protein kinase 2	WNK2	0,79
SH3-domain binding protein 1; pyridoxal (pyridoxine, vitamin B6) phosphatase	SH3BP1; PDXP	0,79
Transcript Identified by AceView, Entrez Gene ID(s) 51128	SAR1B	0,79
adaptor-related protein complex 1 associated regulatory protein	AP1AR	0,79
calcium binding protein 39-like	CAB39L	0,79
double homeobox 1	DUX1	0,79
catechol-O-methyltransferase domain containing 1	COMTD1	0,79
SPATA31 subfamily D, member 1	SPATA31D1	0,79
GLE1 RNA export mediator	GLE1	0,79
granzyme M	GZMM	0,79
glutamic-oxaloacetic transaminase 1-like 1	GOT1L1	0,79
coiled-coil domain containing 155	CCDC155	0,79
RNA binding motif protein 45	RBM45	0,79
Sp4 transcription factor	SP4	0,79
chromosome 20 open reading frame 96	C20orf96	0,79
golgin A2 pseudogene 5	GOLGA2P5	0,79
galactosidase beta 1 like 2	GLB1L2	0,79
zinc finger protein 14	ZNF14	0,79
carbonic anhydrase VIII	CA8	0,79
amylase, alpha 1C (salivary); amylase, alpha 1A (salivary)	AMY1C; AMY1A	0,79
kelch-like family member 3	KLHL3	0,79
calcineurin-like EF-hand protein 1	CHP1	0,79
phosphoinositide-3-kinase, regulatory subunit 5	PIK3R5	0,79
membrane associated ring finger 10	MARCH10	0,79
surfeit 6	SURF6	0,79
family with sequence similarity 120C	FAM120C	0,79
speedy/RINGO cell cycle regulator family member E5	SPDYE5	0,79

RAB37, member RAS oncogene family	RAB37	0,79
adenylate cyclase 10 (soluble)	ADCY10	0,79
chromosome 17 open reading frame 102	C17orf102	0,79
small nuclear ribonucleoprotein D3 polypeptide	SNRPD3	0,79
ash1 (absent, small, or homeotic)-like (Drosophila); microRNA 555	ASH1L; MIR555	0,79
family with sequence similarity 133, member B	FAM133B	0,79
ankyrin repeat and SOCS box containing 11, E3 ubiquitin protein ligase	ASB11	0,79
calsequestrin 1 (fast-twitch, skeletal muscle)	CASQ1	0,79
T-box 10	TBX10	0,79
trafficking protein particle complex 4	TRAPPC4	0,79
olfactory receptor, family 6, subfamily C, member 76	OR6C76	0,79
cytoskeleton associated protein 2-like	CKAP2L	0,79
cytochrome b561 family, member D2	CYB561D2	0,79
succinate dehydrogenase complex assembly factor 1	SDHAF1	0,79
dispatched homolog 2 (Drosophila)	DISP2	0,79
golgin A6 family-like 22; golgin A6 family-like 1	GOLGA6L22; GOLGA6L1	0,79
chromosome 1 open reading frame 174	C1orf174	0,79
zinc and ring finger 4	ZNRF4	0,79
methylenetetrahydrofolate dehydrogenase (NADP+ dependent) 1, methenyltetrahydrofolate cyclohydrolase, formyltetrahydrofolate synthetase	MTHFD1	0,79
yippee like 1	YPEL1	0,79
chromosome 19 open reading frame 54	C19orf54	0,79
Transcript Identified by AceView, Entrez Gene ID(s) 23358	USP24	0,79
gem nuclear organelle associated protein 6	GEMIN6	0,79
extra spindle pole bodies like 1, separase	ESPL1	0,79
recombination signal binding protein for immunoglobulin kappa J region	RBPJ	0,79
solute carrier family 19 (folate transporter), member 1	SLC19A1	0,79
acetoacetyl-CoA synthetase	AACS	0,79
RELT-like 1	RELL1	0,79
G-patch domain containing 1	GPATCH1	0,79
immunoglobin superfamily, member 21	IGSF21	0,79
transmembrane protein 254	TMEM254	0,79
G-patch domain containing 2	GPATCH2	0,79
Transcript Identified by AceView, Entrez Gene ID(s) 118424	UBE2J2	0,79
transmembrane protein 266	TMEM266	0,79
cell division cycle 25C	CDC25C	0,79
activator of basal transcription 1	ABT1	0,79
sel-1 suppressor of lin-12-like (C. elegans)	SEL1L	0,79

ankyrin repeat and SOCS box containing 7	ASB7	0,79
peptidylprolyl isomerase G (cyclophilin G)	PPIG	0,79
kallikrein related peptidase 10	KLK10	0,79
zinc finger protein 546	ZNF546	0,79
tumor necrosis factor, alpha-induced protein 6	TNFAIP6	0,79
gamma-aminobutyric acid (GABA) A receptor, gamma 1	GABRG1	0,79
tripartite motif containing 28	TRIM28	0,79
Eukaryotic translation initiation factor 3 subunit F [Source:UniProtKB/Swiss-Prot;Acc:O00303]	EIF3F	0,79
kelch-like family member 42	KLHL42	0,79
ribosomal protein S13; small nucleolar RNA, C/D box 14B	RPS13; SNORD14B	0,79
transmembrane protein 26	TMEM26	0,79
oligodendrocyte myelin glycoprotein	OMG	0,79
thymosin beta 15B	TMSB15B	0,78
tetratricopeptide repeat domain 5	TTC5	0,78
oxoglutarate dehydrogenase-like	OGDHL	0,78
olfactory receptor, family 13, subfamily C, member 7 (gene/pseudogene) [Source:HGNC Symbol;Acc:HGNC:15102]	OR13C7	0,78
pancreatic lipase-related protein 1	PNLIPRP1	0,78
aurora kinase A	AURKA	0,78
zinc finger protein 445	ZNF445	0,78
diphthamide biosynthesis 3 pseudogene 1	DPH3P1	0,78
ribosomal protein S6 kinase, 90kDa, polypeptide 1	RPS6KA1	0,78
taste receptor, type 2, member 19	TAS2R19	0,78
methionyl aminopeptidase type 1D (mitochondrial)	METAP1D	0,78
F-box and leucine-rich repeat protein 19	FBXL19	0,78
glycine amidinotransferase (L-arginine:glycine amidinotransferase)	GATM	0,78
heterogeneous nuclear ribonucleoprotein L	HNRNPL	0,78
DENN/MADD domain containing 6B	DENND6B	0,78
uncharacterized LOC283710; novel transcript; Transcript Identified by AceView	LOC283710; RP11-16E12.2; rukama	0,78
matrilin 2	MATN2	0,78
phosphorylase kinase, gamma 1 (muscle)	PHKG1	0,78
sec1 family domain containing 1	SCFD1	0,78
heat shock transcription factor 2	HSF2	0,78
glucagon	GCG	0,78
X-linked Kx blood group related 4	XKR4	0,78
tyrosine kinase, non-receptor, 2	TNK2	0,78
TBC1 domain family, member 14	TBC1D14	0,78
cystic fibrosis transmembrane conductance regulator	CFTR	0,78
ras homolog family member T1	RHOT1	0,78

chromosome 11 open reading frame 84	C11orf84	0,78
potassium channel, voltage gated Shaw related subfamily C, member 2	KCNC2	0,78
polymerase (RNA) I polypeptide B	POLR1B	0,78
diacylglycerol kinase, delta 130kDa	DGKD	0,78
hepatoma derived growth factor-like 1	HDGFL1	0,78
lactamase, beta-like 1	LACTBL1	0,78
YY1 associated protein 1	YY1AP1	0,78
cyclin E1	CCNE1	0,78
ribosomal protein L37a	RPL37A	0,78
mucin 1, cell surface associated	MUC1	0,78
cadherin-related family member 3	CDHR3	0,78
aldehyde dehydrogenase 3 family, member B2	ALDH3B2	0,78
transmembrane protein 134	TMEM134	0,78
long intergenic non-protein coding RNA 934	LINC00934	0,78
G protein-coupled receptor kinase 1	GRK1	0,78
cytidine monophosphate (UMP-CMP) kinase 2, mitochondrial	CMPK2	0,78
poly(rC) binding protein 1	PCBP1	0,78
phosphodiesterase 6B, cGMP-specific, rod, beta	PDE6B	0,78
serpin peptidase inhibitor, clade B (ovalbumin), member 6	SERPINB6	0,78
retinal G protein coupled receptor	RGR	0,78
lipin 1; microRNA 548s	LPIN1; MIR548S	0,78
proline rich 5 (renal)	PRR5	0,78
Dmx-like 2	DMXL2	0,78
mitogen-activated protein kinase-activated protein kinase 5; ADAM metallopeptidase domain 1A (pseudogene)	MAPKAPK5; ADAM1A	0,78
chloride channel, nucleotide-sensitive, 1A	CLNS1A	0,78
CTAGE family, member 5	CTAGE5	0,78
ribosomal protein L30	RPL30	0,78
PDZ domain containing 8	PDZD8	0,78
olfactory receptor, family 8, subfamily G, member 1 (gene/pseudogene)	OR8G1	0,78
proline rich 34	PRR34	0,78
KIAA0100	KIAA0100	0,78
interferon regulatory factor 2	IRF2	0,78
doublecortin	DCX	0,78
bromodomain and WD repeat domain containing 1	BRWD1	0,78
kinesin family member 15	KIF15	0,78
gap junction protein alpha 1	GJA1	0,78
Jeck2013 ANTISENSE, CDS, coding, INTERNAL, intronic, OVCODE, OVEXON best transcript NM_015466	PTPN23	0,78

olfactory receptor, family 10, subfamily C, member 1 (gene/pseudogene)	OR10C1	0,78
solute carrier family 17 (organic anion transporter), member 1	SLC17A1	0,78
non-POU domain containing, octamer-binding	NONO	0,78
protein phosphatase 4, regulatory subunit 3B	PPP4R3B	0,78
protease, serine, 57	PRSS57	0,78
cubilin (intrinsic factor-cobalamin receptor)	CUBN	0,78
ribosomal protein SA pseudogene 58; ribosomal protein SA	RPSAP58; RPSA	0,78
zinc finger protein 546	ZNF546	0,78
Memczak2013 ANTISENSE, CDS, coding, INTERNAL, UTR3 best transcript NM_005999	TSNAX	0,78
Transcript Identified by AceView, Entrez Gene ID(s) 10211	FLOT1	0,78
secreted frizzled-related protein 2	SFRP2	0,78
chromosome 14 open reading frame 132	C14orf132	0,78
SOS Ras/Rho guanine nucleotide exchange factor 2	SOS2	0,78
tryptophan rich basic protein	WRB	0,78
protein tyrosine kinase 2 beta	PTK2B	0,78
protease, serine, 3	PRSS3	0,78
sorting nexin 15	SNX15	0,78
olfactory receptor, family 2, subfamily T, member 12	OR2T12	0,78
carcinoembryonic antigen-related cell adhesion molecule 3	CEACAM3	0,78
tumor-associated calcium signal transducer 2	TACSTD2	0,78
transmembrane protein 183B	TMEM183B	0,78
RNA pseudouridylate synthase domain containing 2	RPUSD2	0,78
peroxisomal biogenesis factor 12	PEX12	0,78
brain abundant, membrane attached signal protein 1	BASP1	0,78
hydroxymethylbilane synthase	HMBS	0,78
transcription elongation factor A (SII)-like 7	TCEAL7	0,78
spermatogenesis associated 4	SPATA4	0,78
chromodomain protein, Y-linked, 1B; chromodomain protein, Y-linked, 1	CDY1B; CDY1	0,78
ArfGAP with GTPase domain, ankyrin repeat and PH domain 4	AGAP4	0,78
Memczak2013 ANTISENSE, CDS, coding, INTERNAL, intronic, UTR3 best transcript NM_001099678	LRRC58	0,78
lymphocyte antigen 6 complex, locus G6E (pseudogene)	LY6G6E	0,78
chromosome 14 open reading frame 166	C14orf166	0,78
radial spoke head 4 homolog A (Chlamydomonas)	RSPH4A	0,78
NACC family member 2, BEN and BTB (POZ) domain containing	NACC2	0,78
NOP14 nucleolar protein	NOP14	0,78

cytochrome P450, family 2, subfamily R, polypeptide 1	CYP2R1	0,78
serine/threonine kinase-like domain containing 1	STKLD1	0,78
pleiotrophin	PTN	0,78
HD domain containing 3	HDDC3	0,78
dipeptidyl-peptidase 4	DPP4	0,78
SHC (Src homology 2 domain containing) transforming protein 2	SHC2	0,78
olfactory receptor, family 1, subfamily C, member 1	OR1C1	0,78
dynein, axonemal, assembly factor 2	DNAAF2	0,78
cullin 1	CUL1	0,78
solute carrier family 16 (monocarboxylate transporter), member 1	SLC16A1	0,78
telomeric repeat binding factor (NIMA-interacting) 1	TERF1	0,78
protoporphyrinogen oxidase	PPOX	0,78
olfactomedin like 2B	OLFML2B	0,78
histone cluster 4, H4	HIST4H4	0,78
guanine nucleotide binding protein (G protein), beta polypeptide 4	GNB4	0,78
Rho guanine nucleotide exchange factor 17	ARHGEF17	0,78
cysteine-rich PAK1 inhibitor	CRIPAK	0,78
chromatin target of PRMT1	CHTOP	0,78
chemokine (C motif) ligand 2	XCL2	0,78
transcription factor 7-like 1 (T-cell specific, HMG-box)	TCF7L1	0,78
ZFP92 zinc finger protein	ZFP92	0,78
mex-3 RNA binding family member D	MEX3D	0,78
Memczak2013 ANTISENSE, coding, INTERNAL, intronic best transcript NM_001033556	PPM1B	0,78
decapping enzyme, scavenger	DCPS	0,78
Memczak2013 ALT_ACCEPTOR, ALT_DONOR, coding, INTERNAL, intronic best transcript NM_033028	BBS4	0,78
sperm antigen with calponin homology and coiled-coil domains 1-like	SPECC1L	0,78
Rab interacting lysosomal protein-like 1	RILPL1	0,78
solute carrier family 25 (mitochondrial carrier; phosphate carrier), member 3; small nucleolar RNA, H/ACA box 53	SLC25A3; SNORA53	0,78
protein tyrosine phosphatase, receptor type, S	PTPRS	0,78
melanin-concentrating hormone receptor 2	MCHR2	0,78
exocyst complex component 7; microRNA 6868	EXOC7; MIR6868	0,78
transmembrane protein 202	TMEM202	0,78
SHANK2 antisense RNA 3	SHANK2-AS3	0,78
tetraspanin 16	TSPAN16	0,78
cilia and flagella associated protein 126	CFAP126	0,78
Enah/Vasp-like	EVL	0,78
somatostatin	SST	0,78

aspartyl aminopeptidase	DNPEP	0,78
CD99 molecule	CD99	0,78
transmembrane protein 247	TMEM247	0,78
dual specificity phosphatase 16	DUSP16	0,78
keratin associated protein 4-6	KRTAP4-6	0,78
nuclear receptor coactivator 6	NCOA6	0,78
ST7 antisense RNA 1	ST7-AS1	0,78
Purkinje cell protein 2	PCP2	0,78
solute carrier family 18 (vesicular monoamine transporter), member 1	SLC18A1	0,78
tweety family member 3	TTYH3	0,78
metaxin 3	MTX3	0,78
hydroxy-delta-5-steroid dehydrogenase, 3 beta- and steroid delta-isomerase 7	HSD3B7	0,78
adenylosuccinate lyase	ADSL	0,78
syntaxin 10	STX10	0,78
ubiquitin-like modifier activating enzyme 2	UBA2	0,78
Zhang2013 ALT_ACCEPTOR, ALT_DONOR, coding, INTERNAL, intronic best transcript NM_032737	LMNB2	0,78
cleavage stimulation factor, 3 pre-RNA, subunit 3	CSTF3	0,78
apolipoprotein B mRNA editing enzyme, catalytic polypeptide-like 2	APOBEC2	0,78
translin-associated factor X interacting protein 1	TSNAXIP1	0,78
sprouty RTK signaling antagonist 3	SPRY3	0,78
Homo sapiens sprouty RTK signaling antagonist 3 (SPRY3), transcript variant 1, mRNA.; sprouty homolog 3 (Drosophila) [Source:HGNC Symbol;Acc:HGNC:11271]; Homo sapiens sprouty homolog 3 (Drosophila), mRNA (cDNA clone MGC:97072 IMAGE:7262284), complete cds.; Homo sapiens sprouty homolog 3 (Drosophila), mRNA (cDNA clone MGC:103904 IMAGE:30915293), complete cds.; Homo sapiens sprouty homolog 3 (Drosophila), mRNA (cDNA clone MGC:104063 IMAGE:30915511), complete cds.	SPRY3	0,78
regulator of G-protein signaling 3	RGS3	0,78
SRY box 10	SOX10	0,78
Memczak2013 ALT_ACCEPTOR, ALT_DONOR, coding, INTERNAL, intronic best transcript NM_001142333	RBFOX1	0,78
DEAH (Asp-Glu-Ala-His) box helicase 9	DHX9	0,78
small ArfGAP2	SMAP2	0,78
regulator of chromosome condensation 2	RCC2	0,78
vesicle (multivesicular body) trafficking 1	VTA1	0,78
MKL/myocardin-like 2; TVP23C pseudogene 2	MKL2; TVP23CP2	0,78
lectin, mannose-binding 2	LMAN2	0,78
hemoglobin, gamma G; hemoglobin, gamma A	HBG2; HBG1	0,78

CHKB-CPT1B readthrough (NMD candidate)	CHKB-CPT1B	0,78
dynein heavy chain domain 1	DNHD1	0,78
Transcript Identified by AceView, Entrez Gene ID(s) 153129	SLC38A9	0,78
solute carrier family 25 (mitochondrial carrier; adenine nucleotide translocator), member 6	SLC25A6	0,78
acid phosphatase 1, soluble	ACP1	0,78
dimethylglycine dehydrogenase	DMGDH	0,78
eukaryotic translation initiation factor 4 gamma, 3	EIF4G3	0,78
potassium channel tetramerization domain containing 21	KCTD21	0,78
hedgehog acyltransferase	HHAT	0,78
phospholipase A2, group III	PLA2G3	0,78
olfactory receptor, family 1, subfamily F, member 1	OR1F1	0,78
autophagy related 9A	ATG9A	0,78
KAT8 regulatory NSL complex subunit 3	KANSL3	0,78
KH-type splicing regulatory protein; microRNA 3940	KHSRP; MIR3940	0,78
guanine nucleotide binding protein-like 3 (nucleolar); small nucleolar RNA, C/D box 19B	GNL3; SNORD19B	0,78
chibby homolog 1 (Drosophila)	CBY1	0,78
chromodomain helicase DNA binding protein 9	CHD9	0,78
mal, T-cell differentiation protein	MAL	0,78
claudin domain containing 2	CLDND2	0,78
indian hedgehog	IHH	0,78
gamma-glutamyltransferase light chain 1	GGTLC1	0,78
zinc finger protein 91	ZNF91	0,78
uncharacterized protein MGC27345	MGC27345	0,78
ubiquitin specific peptidase 22	USP22	0,78
DNA replication helicase/nuclease 2	DNA2	0,78
ferredoxin 1	FDX1	0,78
ADP-ribosylation factor 5	ARF5	0,78
dynein, axonemal, light chain 1	DNAL1	0,78
transmembrane protein with EGF-like and two follistatin-like domains 1	TMEFF1	0,78
cytochrome c oxidase subunit VIIa polypeptide 2 like	COX7A2L	0,78
keratin associated protein 19-6	KRTAP19-6	0,78
protein arginine methyltransferase 7	PRMT7	0,78
Vpr (HIV-1) binding protein	VPRBP	0,78
oncoprotein induced transcript 3	OIT3	0,78
ribosomal protein L18a; small nucleolar RNA, H/ACA box 68	RPL18A; SNORA68	0,78
xylosyltransferase I	XYLT1	0,78
NHL repeat containing 2	NHLRC2	0,78
X-linked Kx blood group related 8	XKR8	0,78
BCL2-like 14 (apoptosis facilitator)	BCL2L14	0,78

solute carrier family 25 (mitochondrial carrier; adenine nucleotide translocator), member 6	SLC25A6	0,78
mitochondrial calcium uptake family, member 3	MICU3	0,78
T cell receptor gamma joining 1; T cell receptor gamma constant 2; T cell receptor gamma joining 2; T cell receptor gamma variable 9; T cell receptor gamma constant 1; T cell receptor gamma joining P; T cell receptor gamma joining P2	TRGJ1; TRGC2; TRGJ2; TRGV9; TRGC1; TRGJP; TRGJP2	0,78
ZNF503 antisense RNA 2	ZNF503-AS2	0,78
family with sequence similarity 195, member A	FAM195A	0,78
von Willebrand factor C domain containing protein 2-like	VWC2L	0,78
MAD2 mitotic arrest deficient-like 1 (yeast)	MAD2L1	0,78
zinc finger protein 444	ZNF444	0,78
WD repeat domain 18	WDR18	0,78
keratin 72, type II	KRT72	0,78
zinc finger, ZZ-type with EF-hand domain 1	ZZEF1	0,78
zinc finger protein 563	ZNF563	0,78
glutathione S-transferase alpha 3	GSTA3	0,78
Zic family member 3	ZIC3	0,78
Transcript Identified by AceView, Entrez Gene ID(s) 4642; novel transcript, sense intronic MYO1D	RP11-466A19.5; MYO1D	0,78
ring finger and CCCH-type domains 2	RC3H2	0,77
Memczak2013 ALT_ACCEPTOR, ALT_DONOR, coding, INTERNAL, intronic best transcript NM_139207	NAP1L1	0,77
chromosome 7 open reading frame 73	C7orf73	0,77
protocadherin beta 8	PCDHB8	0,77
heat shock factor binding protein 1	HSBP1	0,77
mannosidase, beta A, lysosomal	MANBA	0,77
RNA binding motif protein 15	RBM15	0,77
tripartite motif containing 2	TRIM2	0,77
UFM1-specific ligase 1	UFL1	0,77
chromosome 16 open reading frame 47	C16orf47	0,77
keratin associated protein 4-5	KRTAP4-5	0,77
eukaryotic translation initiation factor 2, subunit 3 gamma, 52kDa	EIF2S3	0,77
uncharacterized LOC100505549; Salzman2013 ANNOTATED, CDS, coding, OVCODE, OVERLAPTX, OVEXON, UTR3, UTR5 best transcript NM_001242804; novel transcript, antisense to ATP8B1	LOC100505549; RP11-35G9.3	0,77
fibroblast growth factor receptor substrate 2	FRS2	0,77
sushi domain containing 6	SUSD6	0,77
translocated promoter region, nuclear basket protein	TPR	0,77
leucine rich repeat containing 69; microRNA 4661	LRRC69; MIR4661	0,77

novel transcript, antisense to KCNIP4; Transcript Identified by AceView, Entrez Gene ID(s) 133015	RP11-362J17.1; PACRGL	0,77
SHC (Src homology 2 domain containing) transforming protein 3	SHC3	0,77
interleukin 16	IL16	0,77
fibroblast growth factor 21	FGF21	0,77
mucolipin 2	MCOLN2	0,77
prolyl 4-hydroxylase, alpha polypeptide II	P4HA2	0,77
WD repeat domain 46; microRNA 6873	WDR46; MIR6873	0,77
zinc finger, X-linked, duplicated A	ZXDA	0,77
R3H domain and coiled-coil containing 1	R3HCC1	0,77
Transcript Identified by AceView, Entrez Gene ID(s) 10777	ARPP21	0,77
DDRGK domain containing 1	DDRGK1	0,77
leucine rich adaptor protein 1	LURAP1	0,77
chaperonin containing TCP1, subunit 6B (zeta 2)	CCT6B	0,77
sialic acid binding Ig-like lectin 8	SIGLEC8	0,77
autophagy related 4B, cysteine peptidase	ATG4B	0,77
synaptotagmin-like 4	SYTL4	0,77
zinc finger and SCAN domain containing 5B	ZSCAN5B	0,77
zinc finger, BED-type containing 9	ZBED9	0,77
GNAS complex locus	GNAS	0,77
DEAH (Asp-Glu-Ala-Asp/His) box polypeptide 57	DHX57	0,77
transmembrane p24 trafficking protein family member 8	TMED8	0,77
PDX1 C-terminal inhibiting factor 1	PCIF1	0,77
vomeronasal 1 receptor 2	VN1R2	0,77
galanin receptor 1	GALR1	0,77
transformer 2 alpha homolog (Drosophila)	TRA2A	0,77
olfactory receptor, family 1, subfamily E, member 1	OR1E1	0,77
chromosome 14 open reading frame 80	C14orf80	0,77
LIM domain only 1 (rhombotin 1)	LMO1	0,77
zinc finger, MYND-type containing 15	ZMYND15	0,77
cut-like homeobox 2	CUX2	0,77
neuropeptide S receptor 1	NPSR1	0,77
zinc finger protein 546	ZNF546	0,77
lengsin, lens protein with glutamine synthetase domain	LGSN	0,77
exocyst complex component 3-like 4	EXOC3L4	0,77
ATPase family, AAA domain containing 3C	ATAD3C	0,77
archain 1	ARCN1	0,77
actin binding Rho activating protein	ABRA	0,77
ribophorin II	RPN2	0,77
transmembrane and tetratricopeptide repeat containing 3	TMTC3	0,77
olfactory receptor, family 5, subfamily M, member 9	OR5M9	0,77

olfactory receptor, family 56, subfamily A, member 4	OR56A4	0,77
MAP kinase interacting serine/threonine kinase 1	MKNK1	0,77
histone cluster 1, H4f	HIST1H4F	0,77
achaete-scute family bHLH transcription factor 4	ASCL4	0,77
ArfGAP with GTPase domain, ankyrin repeat and PH domain 6	AGAP6	0,77
major facilitator superfamily domain containing 9	MFSD9	0,77
dynein, axonemal, heavy chain 8	DNAH8	0,77
uroplakin 1A	UPK1A	0,77
zinc finger protein 200	ZNF200	0,77
Jeck2013 ALT_ACCEPTOR, ALT_DONOR, coding, INTERNAL, intronic best transcript NM_016836	RBMS1	0,77
SAFB-like, transcription modulator	SLTM	0,77
cystic fibrosis transmembrane conductance regulator	CFTR	0,77
ubiquitin specific peptidase 33	USP33	0,77
TBC1 domain family, member 9B (with GRAM domain)	TBC1D9B	0,77
transmembrane protease, serine 6	TMPRSS6	0,77
microtubule-associated protein 1 light chain 3 beta	MAP1LC3B	0,77
protein tyrosine phosphatase, non-receptor type 20	PTPN20	0,77
zinc finger protein 131	ZNF131	0,77
THAP domain containing 9	THAP9	0,77
complement component 1, r subcomponent	C1R	0,77
adenosylhomocysteinase	AHCY	0,77
dehydrogenase/reductase (SDR family) member 4 like 1	DHRS4L1	0,77
cysteine rich with EGF-like domains 1	CRELD1	0,77
Homo sapiens serine/threonine kinase 19 (STK19), transcript variant 1, mRNA.; Homo sapiens serine/threonine kinase 19 (STK19), transcript variant 2, mRNA.; Homo sapiens serine/threonine kinase 19 (STK19), transcript variant 3, non-coding RNA.; Homo sapiens serine/threonine kinase 19, mRNA (cDNA clone MGC:117388 IMAGE:5165123), complete cds.	STK19	0,77
tRNA methyltransferase O	TRMO	0,77
exosome component 6	EXOSC6	0,77
Transcript Identified by AceView, Entrez Gene ID(s) 55008; novel transcript	RP11-10L7.1; HERC6	0,77
RAB9B, member RAS oncogene family	RAB9B	0,77
fibrinogen-like 2	FGL2	0,77
RNA binding motif protein 12; copine I	RBM12; CPNE1	0,77
lysine (K)-specific methyltransferase 2D	KMT2D	0,77
acireductone dioxygenase 1	ADI1	0,77
polysaccharide biosynthesis domain containing 1	PBDC1	0,77
cadherin 8, type 2	CDH8	0,77
tumor necrosis factor (ligand) superfamily, member 13	TNFSF13	0,77
KIF25 antisense RNA 1	KIF25-AS1	0,77

olfactory receptor, family 4, subfamily C, member 16 (gene/pseudogene)	OR4C16	0,77
chromosome 12 open reading frame 76	C12orf76	0,77
lymphocyte antigen 6 complex, locus E	LY6E	0,77
short chain dehydrogenase/reductase family 16C, member 5	SDR16C5	0,77
zinc finger protein 354C	ZNF354C	0,77
primary cilia formation	PIFO	0,77
Transcript Identified by AceView, Entrez Gene ID(s) 4646	MYO6	0,77
atonal bHLH transcription factor 7	ATOH7	0,77
spastin	SPAST	0,77
inner mitochondrial membrane peptidase subunit 2	IMMP2L	0,77
macrophage erythroblast attacher	MAEA	0,77
phosphodiesterase 4A, cAMP-specific	PDE4A	0,77
transcription factor EB	TFEB	0,77
par-3 family cell polarity regulator beta	PARD3B	0,77
C-type lectin domain family 12, member A	CLEC12A	0,77
EYA transcriptional coactivator and phosphatase 4	EYA4	0,77
storkhead box 2	STOX2	0,77
transient receptor potential cation channel, subfamily C, member 7	TRPC7	0,77
hydroxymethylbilane synthase	HMBS	0,77
Rab acceptor 1 (prenylated)	RABAC1	0,77
serine/threonine kinase 25	STK25	0,77
nuclear export mediator factor	NEMF	0,77
PTPN13-like, Y-linked pseudogene 4	PRYP4	0,77
zinc finger, SWIM-type containing 2	ZSWIM2	0,77
leucine rich repeat containing 74B	LRRC74B	0,77
achaete-scute family bHLH transcription factor 3	ASCL3	0,77
polycomb group ring finger 6	PCGF6	0,77
cysteine/tyrosine-rich 1	CYYR1	0,77
chromosome 1 open reading frame 50	C1orf50	0,77
G protein-coupled receptor 107	GPR107	0,77
peptidyl-tRNA hydrolase domain containing 1	PTRHD1	0,77
polymerase (RNA) II (DNA directed) polypeptide I, 14.5kDa	POLR2I	0,77
protein phosphatase 1, regulatory subunit 27	PPP1R27	0,77
tRNA methyltransferase 11 homolog	TRMT11	0,77
zinc finger protein 658B, pseudogene	ZNF658B	0,77
CAP-GLY domain containing linker protein 3	CLIP3	0,77
castor zinc finger 1	CASZ1	0,77
cytochrome P450, family 46, subfamily A, polypeptide 1	CYP46A1	0,77
Transcript Identified by AceView, Entrez Gene ID(s) 2891	GRIA2	0,77

Transcript Identified by AceView, Entrez Gene ID(s) 26275	HIBCH	0,77
RPS10-NUDT3 readthrough	RPS10-NUDT3	0,77
hydroxyacid oxidase 2 (long chain)	HAO2	0,77
chromosome 19 open reading frame 18	C19orf18	0,77
MCF.2 cell line derived transforming sequence like	MCF2L	0,77
ribonuclease P/MRP 40kDa subunit	RPP40	0,77
peptidylprolyl isomerase A (cyclophilin A)-like 4F	PPIAL4F	0,77
scaffolding protein involved in DNA repair	SPIDR	0,77
high density lipoprotein binding protein	HDLBP	0,77
pleckstrin homology domain containing, family H (with MyTH4 domain) member 3	PLEKHH3	0,77
late cornified envelope 3B	LCE3B	0,77
family with sequence similarity 182, member B	FAM182B	0,77
chromobox homolog 1	CBX1	0,77
elongation factor, RNA polymerase II, 2	ELL2	0,77
zinc finger protein 799	ZNF799	0,77
lysosomal protein transmembrane 4 alpha	LAPTM4A	0,77
LIM and senescent cell antigen-like domains 3-like; LIM and senescent cell antigen-like domains 3	LIMS3L; LIMS3	0,77
RCD1 required for cell differentiation1 homolog (S. pombe)	RQCD1	0,77
chromosome 6 open reading frame 201	C6orf201	0,77
ankyrin repeat and sterile alpha motif domain containing 6	ANKS6	0,77
glycine C-acetyltransferase	GCAT	0,77
Transcript Identified by AceView, Entrez Gene ID(s) 57504	MTA3	0,77
immunoglobulin-like domain containing receptor 1	ILDR1	0,77
ubiquitin associated protein 1 like	UBAP1L	0,77
heterogeneous nuclear ribonucleoprotein C-like 1	HNRNPCL1	0,77
WD repeat domain 45B	WDR45B	0,77
mitochondrial ribosomal protein L22	MRPL22	0,77
heparan sulfate (glucosamine) 3-O-sulfotransferase 1	HS3ST1	0,77
leucine rich repeat containing 36	LRRC36	0,77
ribosomal protein L3; small nucleolar RNA, C/D box 83B; small nucleolar RNA, C/D box 43	RPL3; SNORD83B; SNORD43	0,77
small integral membrane protein 11A	SMIM11A	0,77
latexin	LXN	0,77
Transcript Identified by AceView; Zhang2013 ALT_ACCEPTOR, ALT_DONOR, coding, INTERNAL, intronic best transcript NM_006548	dorsnarby; IGF2BP2	0,77
S-antigen; retina and pineal gland (arrestin)	SAG	0,77
cytoplasmic FMR1 interacting protein 2	CYFIP2	0,77

unc-119 lipid binding chaperone	UNC119	0,77
UFM1-specific peptidase 1 (inactive)	UFSP1	0,77
Transcript Identified by AceView, Entrez Gene ID(s) 10896	OCLM	0,77
protein kinase, cAMP-dependent, regulatory, type I, beta	PRKAR1B	0,77
synaptotagmin XVII	SYT17	0,77
phosphatase and tensin homolog	PTEN	0,77
apolipoprotein B receptor	APOBR	0,77
nicalin	NCLN	0,77
catenin (cadherin-associated protein), alpha 2	CTNNA2	0,77
disco-interacting protein 2 homolog C	DIP2C	0,77
RNA binding motif protein 23	RBM23	0,77
SAP30-like	SAP30L	0,77
zinc finger protein 496	ZNF496	0,77
protein O-linked mannose N-acetylglucosaminyltransferase 1 (beta 1,2-)	POMGNT1	0,77
EPS8-like 2	EPS8L2	0,77
chromosome 1 open reading frame 216	C1orf216	0,77
interleukin 1 receptor accessory protein	IL1RAP	0,77
peroxisomal biogenesis factor 6	PEX6	0,77
guanine nucleotide binding protein (G protein), alpha 14	GNA14	0,77
calsyntenin 3	CLSTN3	0,77
DEAD (Asp-Glu-Ala-Asp) box helicase 1	DDX1	0,77
N(alpha)-acetyltransferase 38, NatC auxiliary subunit	NAA38	0,76
guanine nucleotide binding protein (G protein), gamma transducing activity polypeptide 1	GNGT1	0,76
DnaJ (Hsp40) homolog, subfamily C, member 6	DNAJC6	0,76
Memczak2013 ANTISENSE, CDS, coding, INTERNAL best transcript NM_012433	SF3B1	0,76
nerve growth factor (beta polypeptide)	NGF	0,76
trafficking protein particle complex 12	TRAPPC12	0,76
epidermal growth factor	EGF	0,76
PRAME family member 10	PRAMEF10	0,76
signal recognition particle 9kDa	SRP9	0,76
NUT family member 2D	NUTM2D	0,76
empty spiracles homeobox 1	EMX1	0,76
translocation associated membrane protein 1-like 1	TRAM1L1	0,76
pentatricopeptide repeat domain 3	PTCD3	0,76
amylase, alpha 1B (salivary); amylase, alpha 1A (salivary)	AMY1B; AMY1A	0,76
alpha-2-macroglobulin-like 1	A2ML1	0,76
family with sequence similarity 98, member C	FAM98C	0,76
potassium channel, inwardly rectifying subfamily J, member 13	KCNJ13	0,76

ZFP1 zinc finger protein	ZFP1	0,76
cytoskeleton associated protein 5; small nucleolar RNA, C/D box 67	CKAP5; SNORD67	0,76
4-hydroxy-2-oxoglutarate aldolase 1	HOGA1	0,76
ring finger protein 169	RNF169	0,76
EPH receptor A4	EPHA4	0,76
aldehyde dehydrogenase 1 family, member L1	ALDH1L1	0,76
PRP40 pre-mRNA processing factor 40 homolog A	PRPF40A	0,76
Cbl proto-oncogene C, E3 ubiquitin protein ligase	CBLC	0,76
cripto, FRL-1, cryptic family 1B; cripto, FRL-1, cryptic family 1	CFC1B; CFC1	0,76
olfactory receptor, family 2, subfamily T, member 33	OR2T33	0,76
Salzman2013 ANNOTATED, CDS, coding, INTERNAL, OVCODE, OVEXON best transcript NM_001201380	CNTNAP3B	0,76
chromosome 16 open reading frame 52	C16orf52	0,76
zinc finger protein 77	ZNF77	0,76
proteasome subunit alpha 2	PSMA2	0,76
attractin-like 1	ATRNL1	0,76
ATPase, class VI, type 11C	ATP11C	0,76
transferrin receptor	TFRC	0,76
sodium channel, voltage gated, type III alpha subunit	SCN3A	0,76
ArfGAP with RhoGAP domain, ankyrin repeat and PH domain 1	ARAP1	0,76
structural maintenance of chromosomes 5	SMC5	0,76
calmodulin-lysine N-methyltransferase	CAMKMT	0,76
hydroxymethylbilane synthase	HMBS	0,76
leucine rich repeat containing 58	LRRC58	0,76
hair growth associated	HR	0,76
purinergic receptor P2Y, G-protein coupled, 2	P2RY2	0,76
WD repeat domain 70	WDR70	0,76
aspartyl-tRNA synthetase	DARS	0,76
chromobox homolog 5; microRNA 3198-2	CBX5; MIR3198-2	0,76
thioredoxin reductase 3	TXNRD3	0,76
non imprinted in Prader-Willi/Angelman syndrome 2	NIPA2	0,76
RFNG O-fucosylpeptide 3-beta-N-acetylglucosaminyltransferase	RFNG	0,76
tectonic family member 2	TCTN2	0,76
ubiquitin-like modifier activating enzyme 3	UBA3	0,76
leucine rich repeat containing 71	LRRC71	0,76
phosphoribosyl pyrophosphate amidotransferase	PPAT	0,76
Wilms tumor 1 associated protein	WTAP	0,76
male-specific lethal 1 homolog (Drosophila)	MSL1	0,76
potassium channel tetramerization domain containing 7	KCTD7	0,76
Jeck2013 ALT_ACCEPTOR, ALT_DONOR, coding, INTERNAL, intronic best transcript NM_004529	MLLT3	0,76

nanos homolog 1 (Drosophila)	NANOS1	0,76
PAX3 and PAX7 binding protein 1	PAXBP1	0,76
RASSF1 antisense RNA 1	RASSF1-AS1	0,76
growth factor independent 1 transcription repressor	GFI1	0,76
defensin, beta 110	DEFB110	0,76
RAB1A, member RAS oncogene family	RAB1A	0,76
chromosome 2 open reading frame 27A	C2orf27A	0,76
retinitis pigmentosa 1 (autosomal dominant)	RP1	0,76
purinergic receptor P2X, ligand gated ion channel, 3	P2RX3	0,76
coiled-coil domain containing 177	CCDC177	0,76
nephronophthisis 3 (adolescent)	NPHP3	0,76
cadherin 3, type 1, P-cadherin (placental)	CDH3	0,76
oligodendrocyte transcription factor 3	OLIG3	0,76
vanin 3	VNN3	0,76
solute carrier family 22, member 17	SLC22A17	0,76
proteasome subunit beta 5	PSMB5	0,76
protocadherin beta 9	PCDHB9	0,76
idnK, gluconokinase homolog (E. coli)	IDNK	0,76
B-Raf proto-oncogene, serine/threonine kinase	BRAF	0,76
leucine twenty homeobox	LEUTX	0,76
RAS-like, family 10, member B	RASL10B	0,76
VMA21 vacuolar H+-ATPase homolog (S. cerevisiae)	VMA21	0,76
zinc finger, MYM-type 2	ZMYM2	0,76
zinc finger protein 302	ZNF302	0,76
troponin I type 1 (skeletal, slow)	TNNI1	0,76
chemokine (C-X3-C motif) receptor 1	CX3CR1	0,76
Jeck2013 ANTISENSE, CDS, coding, INTERNAL, OVCODE, OVEXON best transcript NM_130803	MEN1	0,76
angiotensin II receptor, type 2	AGTR2	0,76
protein tyrosine kinase 2	PTK2	0,76
rhophilin, Rho GTPase binding protein 2	RHPN2	0,76
zinc metallopeptidase STE24	ZMPSTE24	0,76
histidine triad nucleotide binding protein 1	HINT1	0,76
limb and CNS expressed 1 like	LIX1L	0,76
interferon, alpha 2	IFNA2	0,76
GTPase, IMAP family member 2	GIMAP2	0,76
fibronectin type III domain containing 1	FNDC1	0,76
dynein, cytoplasmic 2, light intermediate chain 1	DYNC2LI1	0,76
solute carrier organic anion transporter family, member 4A1	SLCO4A1	0,76
eukaryotic translation initiation factor 3, subunit F	EIF3F	0,76
ribosomal protein L14	RPL14	0,76
short stature homeobox	SHOX	0,76

Transcript Identified by AceView; Jeck2013 ALT_ACCEPTOR, ALT_DONOR, coding, INTERNAL, intronic best transcript NM_001003679; putative novel transcript	boyboy; RP4-630A11.3; LEPR	0,76
neuropeptide S	NPS	0,76
ribosomal protein S29	RPS29	0,76
family with sequence similarity 171, member A2	FAM171A2	0,76
sterol O-acyltransferase 1	SOAT1	0,76
ribosomal protein L23a; small nucleolar RNA, C/D box 4B; small nucleolar RNA, C/D box 42B; small nucleolar RNA, C/D box 42A	RPL23A; SNORD4B; SNORD42B; SNORD42A	0,76
polyglutamine binding protein 1	PQBP1	0,76
nicotinamide nucleotide adenylyltransferase 1	NMNAT1	0,76
RFPL3 antisense	RFPL3S	0,76
transmembrane protein 260	TMEM260	0,76
Transcript Identified by AceView, Entrez Gene ID(s) 55604	LRRC16A	0,76
StAR-related lipid transfer domain containing 7	STARD7	0,76
ADAM metallopeptidase with thrombospondin type 1 motif 8	ADAMTS8	0,76
essential meiotic structure-specific endonuclease subunit 2	EME2	0,76
lysine (K)-specific demethylase 8	KDM8	0,76
keratin associated protein 6-3	KRTAP6-3	0,76
	LRIG1	0,76
hydroxymethylbilane synthase	HMBS	0,76
Transcript Identified by AceView, Entrez Gene ID(s) 23126	POGZ	0,76
Jeck2013 ANTISENSE, coding, INTERNAL, intronic best transcript NM_014191	SCN8A	0,76
long intergenic non-protein coding RNA 473	LINC00473	0,76
CD5 molecule-like	CD5L	0,76
REX4 homolog, 3-5 exonuclease	REXO4	0,76
olfactory receptor, family 4, subfamily S, member 1	OR4S1	0,76
mitogen-activated protein kinase 14	MAPK14	0,76
NLR family, pyrin domain containing 3	NLRP3	0,76
SET domain, bifurcated 1	SETDB1	0,76
transmembrane protein 65	TMEM65	0,76
MGAT4 family, member E, pseudogene	MGAT4EP	0,76
jagunal homolog 1	JAGN1	0,76
taste receptor, type 2, member 5	TAS2R5	0,76
cytochrome b5 reductase 3	CYB5R3	0,76
myelin regulatory factor	MYRF	0,76
sterile alpha motif domain containing 1	SAMD1	0,76

pericentrin	PCNT	0,76
TBC1 domain family, member 3K	TBC1D3K	0,76
ribosomal protein S6 kinase, 70kDa, polypeptide 2	RPS6KB2	0,76
LYR motif containing 1	LYRM1	0,76
TraB domain containing	TRABD	0,76
leucine-rich repeat LGI family, member 4	LGI4	0,76
programmed cell death 2	PDCD2	0,76
proline rich 36	PRR36	0,76
BTB (POZ) domain containing 3	BTBD3	0,76
vitamin K epoxide reductase complex subunit 1	VKORC1	0,76
poly(A) binding protein interacting protein 1	PAIP1	0,76
cysteine-rich secretory protein LCCL domain containing 1	CRISPLD1	0,76
nuclear receptor subfamily 1, group I, member 3	NR1I3	0,76
transmembrane protein 109	TMEM109	0,76
Fanconi anemia complementation group C	FANCC	0,76
eukaryotic translation initiation factor 3, subunit E	EIF3E	0,76
ribosomal protein L9 pseudogene 7	RPL9P7	0,76
sorting nexin 33	SNX33	0,76
BCL2/adenovirus E1B 19kDa interacting protein 1	BNIP1	0,76
methylenetetrahydrofolate reductase (NAD(P)H)	MTHFR	0,76
ankyrin repeat domain 9	ANKRD9	0,76
Treacher Collins-Franceschetti syndrome 1	TCOF1	0,76
KDEL (Lys-Asp-Glu-Leu) endoplasmic reticulum protein retention receptor 1	KDELR1	0,76
zinc finger protein 546	ZNF546	0,76
leukotriene A4 hydrolase	LTA4H	0,76
oculomedin	OCLM	0,76
solute carrier family 35, member F5	SLC35F5	0,76
phosphoribosylaminoimidazole carboxylase, phosphoribosylaminoimidazole succinocarboxamide synthetase	PAICS	0,76
uncharacterized LOC101928694; novel transcript, antisense to LIPC	LOC101928694; RP11-355N15.1	0,76
olfactory receptor, family 4, subfamily C, member 12	OR4C12	0,76
C2 calcium-dependent domain containing 2	C2CD2	0,76
diphthamide biosynthesis 5	DPH5	0,76
HECT and RLD domain containing E3 ubiquitin protein ligase family member 6	HERC6	0,76
BRCA1/BRCA2-containing complex subunit 3	BRCC3	0,76
NADH dehydrogenase (ubiquinone) 1 alpha subcomplex, 10, 42kDa	NDUFA10	0,76
Gilles de la Tourette syndrome chromosome region, candidate 1 (non-protein coding)	GTSCR1	0,76
KIAA0368	KIAA0368	0,76

olfactory receptor, family 2, subfamily G, member 3	OR2G3	0,76
MOB kinase activator 1A	MOB1A	0,76
leukocyte immunoglobulin-like receptor, subfamily A (with TM domain), member 2	LILRA2	0,76
serine/threonine kinase 32B	STK32B	0,76
phosphogluconate dehydrogenase	PGD	0,76
histone cluster 1, H2ba	HIST1H2BA	0,76
integrin alpha 2b	ITGA2B	0,76
cytochrome P450, family 26, subfamily A, polypeptide 1	CYP26A1	0,76
CD300 molecule-like family member d	CD300LD	0,75
Memczak2013 ALT_ACCEPTOR, ALT_DONOR, coding, INTERNAL, intronic best transcript NM_212471	PRKAR1A	0,75
hephaestin-like 1	HEPHL1	0,75
SIL1 nucleotide exchange factor	SIL1	0,75
wntless Wnt ligand secretion mediator	WLS	0,75
binder of sperm protein homolog 1	BSPH1	0,75
F-box protein 8	FBXO8	0,75
multiple C2 domains, transmembrane 2	MCTP2	0,75
surfeit 4	SURF4	0,75
chromosome 20 open reading frame 196	C20orf196	0,75
Memczak2013 ANTISENSE, coding, INTERNAL, UTR5 best transcript NM_003494; Transcript Identified by AceView	DYSF; kydor	0,75
lectin, galactoside-binding, soluble, 9C; lectin, galactoside-binding, soluble, 9B	LGALS9C; LGALS9B	0,75
microtubule-associated protein 9	MAP9	0,75
serine/threonine kinase 32A	STK32A	0,75
zinc finger protein 213	ZNF213	0,75
Transcript Identified by AceView, Entrez Gene ID(s) 10082	GPC6	0,75
NK2 homeobox 1	NKX2-1	0,75
homeodomain interacting protein kinase 3	HIPK3	0,75
hydroxymethylbilane synthase	HMBS	0,75
anoctamin 7	ANO7	0,75
calcium and integrin binding 1 (calmyrin)	CIB1	0,75
kelch-like family member 1	KLHL1	0,75
bromo adjacent homology domain containing 1	BAHD1	0,75
GPALPP motifs containing 1	GPALPP1	0,75
RAN binding protein 3	RANBP3	0,75
TEK tyrosine kinase, endothelial	TEK	0,75
abhydrolase domain containing 3	ABHD3	0,75
transmembrane protein 178A	TMEM178A	0,75
heterogeneous nuclear ribonucleoprotein H2 (H)	HNRNPH2	0,75
acyl-CoA dehydrogenase, C-2 to C-3 short chain	ACADS	0,75
WAP four-disulfide core domain 5	WFDC5	0,75

Memczak2013 ANTISENSE, coding, INTERNAL, intronic best transcript NM_001242765	DLGAP1	0,75
exocyst complex component 6	EXOC6	0,75
cation channel, sperm associated 3	CATSPER3	0,75
peroxisomal biogenesis factor 5	PEX5	0,75
thrombospondin-type laminin G domain and EAR repeats	TSPEAR	0,75
ribosomal protein S27	RPS27	0,75
syntaxin 5	STX5	0,75
G protein-coupled receptor, class C, group 5, member B	GPRC5B	0,75
myeloid/lymphoid or mixed-lineage leukemia; translocated to, 10	MLLT10	0,75
ribosomal protein S23	RPS23	0,75
zinc finger and SCAN domain containing 32	ZSCAN32	0,75
ADP-ribosylation factor like GTPase 6 interacting protein 4	ARL6IP4	0,75
histone cluster 3, H3	HIST3H3	0,75
D4, zinc and double PHD fingers, family 3	DPF3	0,75
pecanex homolog (Drosophila)	PCNX	0,75
forkhead box D4-like 4	FOXD4L4	0,75
leucine rich repeat transmembrane neuronal 4	LRRTM4	0,75
unc-13 homolog B (C. elegans)	UNC13B	0,75
ubiquitin conjugating enzyme E2N-like (gene/pseudogene)	UBE2NL	0,75
glucose-6-phosphate isomerase	GPI	0,75
activating transcription factor 7 interacting protein	ATF7IP	0,75
guanine nucleotide binding protein (G protein), beta polypeptide 2-like 1; small nucleolar RNA, C/D box 95; small nucleolar RNA, C/D box 96A	GNB2L1; SNORD95; SNORD96A	0,75
gap junction protein delta 3	GJD3	0,75
intraflagellar transport 27	IFT27	0,75
kinesin family member 15	KIF15	0,75
neuro-oncological ventral antigen 1	NOVA1	0,75
fukutin	FKTN	0,75
heparan-alpha-glucosaminide N-acetyltransferase	HGSNAT	0,75
adrenoceptor alpha 1B	ADRA1B	0,75
tectonin beta-propeller repeat containing 2	TECPR2	0,75
nicotinamide riboside kinase 1	NMRK1	0,75
N(alpha)-acetyltransferase 40, NatD catalytic subunit	NAA40	0,75
platelet/endothelial cell adhesion molecule 1	PECAM1	0,75
RUN and SH3 domain containing 1	RUSC1	0,75
zinc finger protein 500	ZNF500	0,75
NFKB repressing factor	NKRF	0,75
DCN1, defective in cullin neddylation 1, domain containing 2	DCUN1D2	0,75

mitochondrial fission process 1	MTFP1	0,75
ATP binding cassette subfamily A member 13	ABCA13	0,75
ribosomal protein L13a; small nucleolar RNA, C/D box 35A; small nucleolar RNA, C/D box 34; small nucleolar RNA, C/D box 33; small nucleolar RNA, C/D box 32A	RPL13A; SNORD35A; SNORD34; SNORD33; SNORD32A	0,75
poly(A) binding protein, cytoplasmic 4 (inducible form)	PABPC4	0,75
F-box and WD repeat domain containing 10	FBXW10	0,75
ribosomal protein L27	RPL27	0,75
olfactory receptor, family 2, subfamily M, member 3	OR2M3	0,75
fumarylacetoacetate hydrolase domain containing 1	FAHD1	0,75
apelin receptor	APLNR	0,75
EXOC3 antisense RNA 1	EXOC3-AS1	0,75
lipopolysaccharide-induced TNF factor	LITAF	0,75
family with sequence similarity 193, member A	FAM193A	0,75
serine/threonine kinase 35	STK35	0,75
ribosomal RNA processing 12 homolog	RRP12	0,75
Transcript Identified by AceView, Entrez Gene ID(s) 23126	POGZ	0,75
microtubule-associated protein, RP/EB family, member 2	MAPRE2	0,75
ubiquitin-conjugating enzyme E2C	UBE2C	0,75
coiled-coil-helix-coiled-coil-helix domain containing 7	CHCHD7	0,75
ADAM metallopeptidase with thrombospondin type 1 motif 13	ADAMTS13	0,75
cullin-associated and neddylation-dissociated 2 (putative)	CAND2	0,75
family with sequence similarity 102, member B	FAM102B	0,75
mitochondrial ribosomal protein S36	MRPS36	0,75
Memczak2013 ANTISENSE, CDS, coding, INTERNAL best transcript NM_015070	ZC3H13	0,75
Memczak2013 ANTISENSE, coding, INTERNAL, intronic best transcript NM_001165899	PDE4D	0,75
chemokine (C-X-C motif) receptor 1	CXCR1	0,75
Mov10 RISC complex RNA helicase	MOV10	0,75
Rho guanine nucleotide exchange factor 38	ARHGEF38	0,75
cystin 1	CYS1	0,75
protocadherin beta 16	PCDHB16	0,75
SSSCA1 antisense RNA 1 (head to head)	SSSCA1-AS1	0,75
tubulin, gamma complex associated protein 4	TUBGCP4	0,75
eva-1 homolog A (C. elegans)	EVA1A	0,75
ring finger protein 26	RNF26	0,75
myocardin	MYOCD	0,75
zinc finger, MYM-type 1	ZMYM1	0,75

BCL2-associated X protein	BAX	0,75
mediator complex subunit 18	MED18	0,75
zinc finger protein 484	ZNF484	0,75
acid sensing ion channel 1	ASIC1	0,75
stromal antigen 3-like 2 (pseudogene)	STAG3L2	0,75
ubiquitin protein ligase E3D	UBE3D	0,75
SH3 domain containing 19	SH3D19	0,75
myelin protein zero-like 1	MPZL1	0,75
integrin-binding sialoprotein	IBSP	0,75
RNA binding motif protein 44	RBM44	0,75
matrix metallopeptidase 9	MMP9	0,75
Transcript Identified by AceView, Entrez Gene ID(s) 10777	ARPP21	0,75
NADH dehydrogenase (ubiquinone) 1 beta subcomplex, 1, 7kDa	NDUFB1	0,75
RASD family, member 2	RASD2	0,75
structure specific recognition protein 1	SSRP1	0,75
methionyl aminopeptidase 2	METAP2	0,75
Memczak2013 ALT_ACCEPTOR, ALT_DONOR, coding, INTERNAL, intronic best transcript NM_022492	TTC31	0,75
Transcript Identified by AceView, Entrez Gene ID(s) 5019	OXCT1	0,75
ribosomal protein L6	RPL6	0,75
Down syndrome critical region 8	DSCR8	0,75
emopamil binding protein-like	EBPL	0,75
KIAA1191	KIAA1191	0,75
X-linked Kx blood group	XK	0,75
Transcript Identified by AceView, Entrez Gene ID(s) 10450	PPIE	0,75
ring finger protein 5, E3 ubiquitin protein ligase	RNF5	0,75
granzyme K	GZMK	0,75
PACRG antisense RNA 3	PACRG-AS3	0,75
fragile X mental retardation 1	FMR1	0,75
transmembrane protein 204	TMEM204	0,75
spermatogenesis associated 5	SPATA5	0,75
protamine 2	PRM2	0,75
forkhead box G1	FOXG1	0,75
phospholipase A2, group XVI	PLA2G16	0,75
C1q and tumor necrosis factor related protein 3	C1QTNF3	0,75
eukaryotic translation elongation factor 1 alpha 1	EEF1A1	0,75
non-SMC condensin II complex subunit H2	NCAPH2	0,75
Transcript Identified by AceView, Entrez Gene ID(s) 404636	FAM45A	0,75
synaptotagmin XV	SYT15	0,75
proteasome subunit beta 6	PSMB6	0,75

family with sequence similarity 161, member B	FAM161B	0,75
mitochondrial ribosomal protein L4	MRPL4	0,75
fructosamine 3 kinase	FN3K	0,75
ring finger protein 24	RNF24	0,75
butyrophilin-like 10	BTNL10	0,75
cyclin J	CCNJ	0,75
X-linked Kx blood group related 6	XKR6	0,75
adaptor protein, phosphotyrosine interaction, PH domain and leucine zipper containing 2	APPL2	0,75
Jeck2013 ANTISENSE, CDS, coding, INTERNAL, intronic, OVCODE, OVEXON best transcript NM_003131	SRF	0,75
split hand/foot malformation (ectrodactyly) type 1	SHFM1	0,75
zinc finger protein 891	ZNF891	0,75
chromodomain protein, Y-linked, 2B; chromodomain protein, Y-linked, 2A	CDY2B; CDY2A	0,75
transforming growth factor beta regulator 4; small nucleolar RNA, H/ACA box 5B	TBRG4; SNORA5B	0,75
cyclin-dependent kinase 12	CDK12	0,75
olfactory receptor, family 6, subfamily Y, member 1	OR6Y1	0,75
Transcript Identified by AceView, Entrez Gene ID(s) 4674	NAP1L2	0,75
G protein-coupled receptor 52	GPR52	0,75
solute carrier family 2 (facilitated glucose/fructose transporter), member 5	SLC2A5	0,75
astacin-like metallo-endopeptidase (M12 family)	ASTL	0,75
glyoxalase domain containing 4	GLOD4	0,75
zinc finger protein 607	ZNF607	0,75
retinoblastoma binding protein 7	RBBP7	0,75
solute carrier family 15, member 5	SLC15A5	0,75
RIC1 homolog, RAB6A GEF complex partner 1	RIC1	0,75
Fanconi anemia core complex associated protein 100	FAAP100	0,74
BCL2-like 14 (apoptosis facilitator)	BCL2L14	0,74
serrate, RNA effector molecule	SRRT	0,74
SEC62 homolog, preprotein translocation factor	SEC62	0,74
PCI domain containing 2	PCID2	0,74
olfactory receptor, family 6, subfamily C, member 68	OR6C68	0,74
calcium channel flower domain containing 1	CACFD1	0,74
Memczak2013 ANTISENSE, CDS, coding, INTERNAL best transcript NM_006044	HDAC6	0,74
pseudouridylate synthase 7 (putative)	PUS7	0,74
zinc finger protein 263	ZNF263	0,74
MAS-related GPR, member E	MRGPRE	0,74
Cdk5 and Abl enzyme substrate 1	CABLES1	0,74
tripartite motif containing 52	TRIM52	0,74
RAB15, member RAS oncogene family	RAB15	0,74

Transcript Identified by AceView, Entrez Gene ID(s) 22873	DZIP1	0,74
forkhead box M1	FOXM1	0,74
potassium channel, voltage gated subfamily E regulatory beta subunit 4	KCNE4	0,74
aldehyde dehydrogenase 9 family, member A1	ALDH9A1	0,74
magnesium-dependent phosphatase 1; NEDD8-MDP1 readthrough; neural precursor cell expressed, developmentally down-regulated 8; charged multivesicular body protein 4A	MDP1; NEDD8-MDP1; NEDD8; CHMP4A	0,74
NADH dehydrogenase (ubiquinone) Fe-S protein 4, 18kDa (NADH-coenzyme Q reductase)	NDUFS4	0,74
nuclear pore complex interacting protein family, member A7; nuclear pore complex interacting protein family, member A8; polycystic kidney disease 1 (autosomal dominant) pseudogene 1	NPIPA7; NPIPA8; PKD1P1	0,74
RIC8 guanine nucleotide exchange factor B	RIC8B	0,74
FYVE, RhoGEF and PH domain containing 1	FGD1	0,74
replication timing regulatory factor 1	RIF1	0,74
mitochondrial ribosomal protein L39	MRPL39	0,74
POM121 transmembrane nucleoporin-like 12	POM121L12	0,74
gamma-glutamyltransferase 6	GGT6	0,74
RANBP2-like and GRIP domain containing 4	RGPD4	0,74
nudix hydrolase 11	NUDT11	0,74
KH domain containing, RNA binding, signal transduction associated 3	KHDRBS3	0,74
ataxin 10	ATXN10	0,74
ring finger protein 133	RNF133	0,74
nudix hydrolase 22	NUDT22	0,74
hCG1657980; Transcript Identified by AceView, Entrez Gene ID(s) 200726, RefSeq ID(s) NM_001102659; novel transcript	LOC200726; AC010731.4	0,74
ribosomal protein L34	RPL34	0,74
gap junction protein gamma 3	GJC3	0,74
dipeptidyl-peptidase 10 (inactive)	DPP10	0,74
TATA element modulatory factor 1	TMF1	0,74
potassium channel, calcium activated intermediate/small conductance subfamily N alpha, member 3	KCNN3	0,74
armadillo repeat containing, X-linked 6	ARMCX6	0,74
N(alpha)-acetyltransferase 16, NatA auxiliary subunit	NAA16	0,74
chromosome X open reading frame 40A	CXorf40A	0,74
zinc finger protein 286B; forkhead box O3B pseudogene	ZNF286B; FOXO3B	0,74
phosphoribosyl transferase domain containing 1	PRTFDC1	0,74

NADH dehydrogenase (ubiquinone) Fe-S protein 6, 13kDa (NADH-coenzyme Q reductase)	NDUFS6	0,74
ATP synthase, H+ transporting, mitochondrial Fo complex subunit C2 (subunit 9)	ATP5G2	0,74
zinc finger protein 780A	ZNF780A	0,74
transmembrane protein 67	TMEM67	0,74
N-deacetylase/N-sulfotransferase (heparan glucosaminyl) 2	NDST2	0,74
inducible T-cell co-stimulator	ICOS	0,74
histone cluster 1, H2bj	HIST1H2BJ	0,74
protein phosphatase 1, regulatory subunit 3D	PPP1R3D	0,74
zinc finger protein 852	ZNF852	0,74
glycerol-3-phosphate dehydrogenase 2	GPD2	0,74
Rho guanine nucleotide exchange factor 28	ARHGEF28	0,74
TGFB1-induced anti-apoptotic factor 1; myosin XVIIIA	TIAF1; MYO18A	0,74
proline rich 7 (synaptic)	PRR7	0,74
solute carrier family 5 (sodium/multivitamin and iodide cotransporter), member 6	SLC5A6	0,74
synovial apoptosis inhibitor 1, synoviolin	SYVN1	0,74
heat shock factor binding protein 1-like 1	HSBP1L1	0,74
sodium channel, voltage gated, type II alpha subunit	SCN2A	0,74
perilipin 2	PLIN2	0,74
ataxin 7-like 3	ATXN7L3	0,74
ring finger protein 148	RNF148	0,74
olfactory receptor, family 2, subfamily G, member 6	OR2G6	0,74
POTE ankyrin domain family, member B2; POTE ankyrin domain family, member B; POTE ankyrin domain family, member B3	POTEB2; POTEB; POTEB3	0,74
dystrobrevin binding protein 1	DTNBP1	0,74
MPV17 mitochondrial membrane protein-like 2	MPV17L2	0,74
zinc finger protein 620	ZNF620	0,74
SID1 transmembrane family, member 2	SIDT2	0,74
stress-associated endoplasmic reticulum protein family member 2	SERP2	0,74
Transcript Identified by AceView, Entrez Gene ID(s) 80205	CHD9	0,74
uncharacterized LOC399886; Transcript Identified by AceView, Entrez Gene ID(s) 399886; novel transcript	LOC399886; RP11-430H10.2; FLJ41423	0,74
non-SMC condensin II complex subunit D3	NCAPD3	0,74
solute carrier family 16 (monocarboxylate transporter), member 1	SLC16A1	0,74
WAS/WASL interacting protein family, member 1	WIPF1	0,74
leucine, glutamate and lysine rich 1	LEKR1	0,74
proline-rich coiled-coil 1	PRRC1	0,74

cullin 4B	CUL4B	0,74
SH2B adaptor protein 1	SH2B1	0,74
SFT2 domain containing 2	SFT2D2	0,74
ecto-NOX disulfide-thiol exchanger 2	ENOX2	0,74
uronyl-2-sulfotransferase	UST	0,74
WD repeat domain 48	WDR48	0,74
CDC42 effector protein (Rho GTPase binding) 1	CDC42EP1	0,74
tRNA methyltransferase 1 like	TRMT1L	0,74
transcription factor CP2	TFCP2	0,74
secernin 3	SCRN3	0,74
G protein-coupled receptor 32	GPR32	0,74
pyruvate dehydrogenase (lipoamide) alpha 2	PDHA2	0,74
opsin 3	OPN3	0,74
jumonji domain containing 6	JMJD6	0,74
small G protein signaling modulator 3	SGSM3	0,74
GATA binding protein 6	GATA6	0,74
storkhead box 1	STOX1	0,74
chromosome 16 open reading frame 58	C16orf58	0,74
F-box protein 9	FBXO9	0,74
carbonyl reductase 1	CBR1	0,74
serine/threonine kinase 38 like	STK38L	0,74
leucine zipper, putative tumor suppressor family member 3; Leucine zipper putative tumor suppressor 3 [Source:UniProtKB/Swiss-Prot;Acc:O60299]; Salzman2013 ANNOTATED, CDS, coding, OVCODE, OVEXON, UTR5 best transcript NM_014731	LZTS3; ProSAPiP1	0,74
LSM8 homolog, U6 small nuclear RNA associated	LSM8	0,74
nuclear factor of kappa light polypeptide gene enhancer in B-cells inhibitor, zeta	NFKBIZ	0,74
fat storage-inducing transmembrane protein 2	FITM2	0,74
mitogen-activated protein kinase 8	MAPK8	0,74
zinc finger family member 788	ZNF788	0,74
Transcript Identified by AceView, Entrez Gene ID(s) 116441	TM4SF18	0,74
gem nuclear organelle associated protein 5	GEMIN5	0,74
glucoside xylosyltransferase 1	GXYLT1	0,74
BPI fold containing family B, member 3	BPIFB3	0,74
WD and tetratricopeptide repeats 1	WDTC1	0,74
ubiquitin specific peptidase 31	USP31	0,74
v-crk avian sarcoma virus CT10 oncogene homolog-like	CRKL	0,74
family with sequence similarity 45, member A	FAM45A	0,74
mitochondrial translational initiation factor 3	MTIF3	0,74
long intergenic non-protein coding RNA 1549	LINC01549	0,74
olfactory receptor, family 4, subfamily X, member 1 (gene/pseudogene)	OR4X1	0,74

Memczak2013 ALT_ACCEPTOR, ALT_DONOR, coding, INTERNAL, intronic best transcript NM_012479	YWHAG	0,74
transient receptor potential cation channel, subfamily C, member 2-like; Salzman2013 ANNOTATED, INTERNAL, ncRNA, OVEXON best transcript NR_029192; Transcript Identified by AceView, Entrez Gene ID(s) 100133315, RefSeq ID(s) NR_029192; Transcript Identified by AceView, Entrez Gene ID(s) 100133315; Putative short transient receptor potential channel 2-like protein [Source:UniProtKB/Swiss-Prot;Acc:Q6ZNB5]	LOC100133315; RP11-849H4.2	0,74
kaptin (actin binding protein)	KPTN	0,74
DDB1 and CUL4 associated factor 7	DCAF7	0,74
oxidoreductase NAD-binding domain containing 1	OXNAD1	0,74
RFT1 homolog	RFT1	0,74
radial spoke head 1 homolog (Chlamydomonas)	RSPH1	0,74
corepressor interacting with RBPJ, 1	CIR1	0,74
elongator acetyltransferase complex subunit 2	ELP2	0,74
tumor protein p53 inducible protein 11	TP53I11	0,74
golgi phosphoprotein 3-like	GOLPH3L	0,74
ribosomal protein L35	RPL35	0,74
glycosylphosphatidylinositol anchored molecule like	GML	0,74
anaphase promoting complex subunit 16	ANAPC16	0,74
UBA domain containing 1	UBAC1	0,74
secretion associated, Ras related GTPase 1B	SAR1B	0,74
chromosome 20 open reading frame 203	C20orf203	0,74
zinc finger protein 615	ZNF615	0,74
nuclear respiratory factor 1	NRF1	0,74
coatomer protein complex subunit alpha	COPA	0,74
Memczak2013 ALT_ACCEPTOR, ALT_DONOR, coding, INTERNAL, intronic best transcript NM_022479	WBSCR17	0,74
zinc finger and BTB domain containing 46	ZBTB46	0,74
caspase 7	CASP7	0,74
serine dehydratase	SDS	0,74
TSPY-like 4	TSPYL4	0,74
amino-terminal enhancer of split	AES	0,74
mitochondrial ribosomal protein L41	MRPL41	0,74
heterogeneous nuclear ribonucleoprotein D like	HNRNPDL	0,74
reticulocalbin 3, EF-hand calcium binding domain	RCN3	0,74
Memczak2013 ALT_ACCEPTOR, ALT_DONOR, coding, INTERNAL, intronic best transcript NM_001166007	EPB41	0,74
adenosine deaminase-like	ADAL	0,74
MAU2 sister chromatid cohesion factor	MAU2	0,74
NUT family member 2B	NUTM2B	0,74
zinc finger protein 587B	ZNF587B	0,74

Transcript Identified by AceView; Memczak2013 ANTISENSE, CDS, coding, INTERNAL, UTR3 best transcript NM_004526	goyborbu; MCM2	0,74
WD repeat domain 4	WDR4	0,74
TBC1 (tre-2/USP6, BUB2, cdc16) domain family, member 1	TBC1D1	0,74
transmembrane protein 9	TMEM9	0,74
threonyl-tRNA synthetase 2, mitochondrial (putative); microRNA 6878	TARS2; MIR6878	0,74
peptidylprolyl isomerase B (cyclophilin B)	PPIB	0,74
ring finger protein 123	RNF123	0,73
fatty acid binding protein 2, intestinal	FABP2	0,73
claudin 11	CLDN11	0,73
stanniocalcin 2	STC2	0,73
ninjurin 1	NINJ1	0,73
aldehyde dehydrogenase 7 family, member A1	ALDH7A1	0,73
heterogeneous nuclear ribonucleoprotein A1	HNRNPA1	0,73
SEC22 homolog C, vesicle trafficking protein	SEC22C	0,73
signal-regulatory protein alpha	SIRPA	0,73
ubiquitination factor E4B	UBE4B	0,73
zinc finger protein 664	ZNF664	0,73
erythropoietin receptor	EPOR	0,73
kinesin family member 5B	KIF5B	0,73
calnexin	CANX	0,73
dedicator of cytokinesis 8	DOCK8	0,73
ninein-like	NINL	0,73
glutamyl-tRNA(Gln) amidotransferase, subunit C	GATC	0,73
matrix metallopeptidase 11	MMP11	0,73
serine/arginine-rich splicing factor 7	SRSF7	0,73
ribosomal protein L23a pseudogene 5	RPL23AP5	0,73
decapping mRNA 1A	DCP1A	0,73
stromal antigen 3-like 3 (pseudogene); stromal antigen 3-like 2 (pseudogene)	STAG3L3; STAG3L2	0,73
olfactory receptor, family 4, subfamily F, member 5	OR4F5	0,73
olfactory receptor, family 10, subfamily H, member 1	OR10H1	0,73
sushi, nidogen and EGF-like domains 1	SNED1	0,73
ecotropic viral integration site 5	EVI5	0,73
SLAM family member 8	SLAMF8	0,73
InaD-like (Drosophila)	INADL	0,73
caspase 16, pseudogene	CASP16P	0,73
Transcript Identified by AceView, Entrez Gene ID(s) 9793	CKAP5	0,73
zinc finger protein 780A	ZNF780A	0,73
ubiquitin conjugating enzyme E2N	UBE2N	0,73
synaptonemal complex central element protein 2	SYCE2	0,73

chromosome 5 open reading frame 66	C5orf66	0,73
deleted in lung and esophageal cancer 1	DLEC1	0,73
Memczak2013 ALT_ACCEPTOR, ALT_DONOR, coding, INTERNAL, intronic best transcript NM_018460	ARHGAP15	0,73
fibrillin 3	FBN3	0,73
vasohibin 2	VASH2	0,73
poly(A) binding protein interacting protein 2	PAIP2	0,73
minichromosome maintenance complex component 7	MCM7	0,73
major facilitator superfamily domain containing 3	MFSD3	0,73
NADH dehydrogenase (ubiquinone) 1 beta subcomplex, 6, 17kDa	NDUFB6	0,73
RecQ helicase-like 4	RECQL4	0,73
secreted phosphoprotein 1	SPP1	0,73
keratin associated protein 23-1	KRTAP23-1	0,73
eukaryotic translation elongation factor 2; small nucleolar RNA, C/D box 37	EEF2; SNORD37	0,73
aminoadipate-semialdehyde dehydrogenase	AASDH	0,73
armadillo repeat containing 12	ARMC12	0,73
zinc finger protein 565	ZNF565	0,73
leucine rich repeat containing 20	LRRC20	0,73
olfactory receptor, family 52, subfamily L, member 1	OR52L1	0,73
annexin A9	ANXA9	0,73
Transcript Identified by AceView, Entrez Gene ID(s) 57798; novel transcript, antisense to ERVW-1 and PEX1	AC007566.10; GATAD1	0,73
S-antigen; retina and pineal gland (arrestin)	SAG	0,73
protein phosphatase 4, regulatory subunit 1	PPP4R1	0,73
ring finger protein 166	RNF166	0,73
proline-rich basic protein 1	PROB1	0,73
calcium channel flower domain containing 1	CACFD1	0,73
tumor protein p63 regulated 1-like	TPRG1L	0,73
histone deacetylase 11	HDAC11	0,73
ATP synthase, H+ transporting, mitochondrial Fo complex subunit F2	ATP5J2	0,73
FAT atypical cadherin 4	FAT4	0,73
minichromosome maintenance 10 replication initiation factor	MCM10	0,73
family with sequence similarity 199, X-linked	FAM199X	0,73
anoctamin 8	ANO8	0,73
KH domain containing, RNA binding, signal transduction associated 1	KHDRBS1	0,73
zinc finger and SCAN domain containing 29	ZSCAN29	0,73
RWD domain containing 4	RWDD4	0,73
DCN1, defective in cullin neddylation 1, domain containing 4	DCUN1D4	0,73
protein geranylgeranyltransferase type I, beta subunit	PGGT1B	0,73

LHX4 antisense RNA 1; acyl-CoA binding domain containing 6	LHX4-AS1; ACBD6	0,73
tight junction protein 2	TJP2	0,73
zinc finger protein 442	ZNF442	0,73
ADP-ribosylation factor interacting protein 2	ARFIP2	0,73
olfactory receptor, family 2, subfamily AE, member 1	OR2AE1	0,73
DDB1 and CUL4 associated factor 5	DCAF5	0,73
tRNA methyltransferase 61B	TRMT61B	0,73
isoleucyl-tRNA synthetase 2, mitochondrial	IARS2	0,73
carbohydrate (chondroitin 6) sulfotransferase 3	CHST3	0,73
SURP and G-patch domain containing 2	SUGP2	0,73
MIF antisense RNA 1	MIF-AS1	0,73
hematopoietic SH2 domain containing	HSH2D	0,73
agouti related neuropeptide	AGRP	0,73
sperm associated antigen 1	SPAG1	0,73
zinc ribbon domain containing 1	ZNRD1	0,73
Myb-related transcription factor, partner of profilin	MYPOP	0,73
fatty acyl CoA reductase 1	FAR1	0,73
inositol 1,4,5-trisphosphate receptor, type 1	ITPR1	0,73
CXXC finger protein 1	CXXC1	0,73
MALT1 paracaspase	MALT1	0,73
F-box protein 21	FBXO21	0,73
glioma tumor suppressor candidate region gene 2; small nucleolar RNA, C/D box 23	GLTSCR2; SNORD23	0,73
Rho GTPase activating protein 26	ARHGAP26	0,73
Rho GTPase activating protein 32	ARHGAP32	0,73
polycomb group ring finger 2	PCGF2	0,73
exportin, tRNA	XPOT	0,73
calcium channel flower domain containing 1	CACFD1	0,73
Transcript Identified by AceView, Entrez Gene ID(s) 10580	SORBS1	0,73
zinc finger protein 552	ZNF552	0,73
coiled-coil and C2 domain containing 2B	CC2D2B	0,73
heterogeneous nuclear ribonucleoprotein A3; microRNA 4444-2; microRNA 4444-1	HNRNPA3; MIR4444-2; MIR4444-1	0,73
oxysterol binding protein-like 9	OSBPL9	0,73
BCL2 binding component 3; microRNA 3191	BBC3; MIR3191	0,73
zinc finger protein 425	ZNF425	0,73
kinesin family member 15	KIF15	0,73
negative elongation factor complex member A; microRNA 943	NELFA; MIR943	0,73
cilia and flagella associated protein 221	CFAP221	0,73
long intergenic non-protein coding RNA 1089	LINC01089	0,73
SMC5-SMC6 complex localization factor 2	SLF2	0,73

synuclein alpha	SNCA	0,73
Rac GTPase activating protein 1	RACGAP1	0,73
ATPase type 13A1	ATP13A1	0,73
KISS1 receptor	KISS1R	0,73
ankyrin repeat and MYND domain containing 2	ANKMY2	0,73
dicarbonyl/L-xylulose reductase	DCXR	0,73
Memczak2013 ANTISENSE, CDS, coding, INTERNAL, UTR3 best transcript NM_004521	KIF5B	0,73
aquaporin 12A	AQP12A	0,73
GH3 domain containing	GHDC	0,73
transmembrane protein 63B	TMEM63B	0,73
amnion associated transmembrane protein	AMN	0,73
Transcript Identified by AceView, Entrez Gene ID(s) 54875	CNTLN	0,73
melanocortin 2 receptor accessory protein	MRAP	0,73
golgin A3	GOLGA3	0,73
chromosome 2 open reading frame 78	C2orf78	0,73
histone cluster 3, H2bb	HIST3H2BB	0,73
Memczak2013 ANTISENSE, CDS, coding, INTERNAL, intronic best transcript NM_003049	SLC10A1	0,73
solute carrier family 6 (neutral amino acid transporter), member 18	SLC6A18	0,73
component of oligomeric golgi complex 4	COG4	0,73
carcinoembryonic antigen-related cell adhesion molecule 5	CEACAM5	0,73
transmembrane 4 L six family member 5	TM4SF5	0,73
neuroblastoma breakpoint family, member 6; neuroblastoma breakpoint family, member 5, pseudogene	NBPF6; NBPF5P	0,73
septin 12	sept-12	0,73
cAMP responsive element binding protein 1	CREB1	0,73
calcyphosine-like	CAPSL	0,73
NPC1-like 1	NPC1L1	0,73
Zhang2013 ALT_ACCEPTOR, ALT_DONOR, coding, INTERNAL, intronic best transcript NM_001146018	TJAP1	0,73
MGA, MAX dimerization protein	MGA	0,73
osteoglycin	OGN	0,73
acidic nuclear phosphoprotein 32 family member A	ANP32A	0,73
zinc finger protein 35	ZNF35	0,73
transmembrane protein 258; microRNA 611	TMEM258; MIR611	0,73
leucine rich repeat containing 23	LRRC23	0,73
anoctamin 1, calcium activated chloride channel	ANO1	0,73
Rho GTPase activating protein 24	ARHGAP24	0,73
surfactant protein A2; surfactant protein A1	SFTPA2; SFTPA1	0,73

polymerase (DNA-directed), delta interacting protein 2	POLDIP2	0,73
vascular endothelial growth factor B	VEGFB	0,73
zinc finger with KRAB and SCAN domains 7	ZKSCAN7	0,73
centrosomal protein 162kDa	CEP162	0,73
SRY box 11	SOX11	0,73
family with sequence similarity 208, member B	FAM208B	0,73
ATP synthase, H+ transporting, mitochondrial F1 complex, gamma polypeptide 1	ATP5C1	0,73
nicotinamide N-methyltransferase	NNMT	0,73
olfactory receptor, family 8, subfamily A, member 1	OR8A1	0,73
secreted frizzled-related protein 5	SFRP5	0,73
zinc finger protein 10	ZNF10	0,73
olfactory receptor, family 2, subfamily J, member 1 (gene/pseudogene)	OR2J1	0,73
ribosomal protein S25	RPS25	0,73
axin 1	AXIN1	0,73
caldesmon 1	CALD1	0,73
cleavage and polyadenylation specific factor 2	CPSF2	0,73
calponin 3, acidic	CNN3	0,73
eukaryotic translation initiation factor 4E binding protein 1	EIF4EBP1	0,73
PRAME family member 10	PRAMEF10	0,73
dynein, light chain, LC8-type 2	DYNLL2	0,73
Zhang2013 ALT_ACCEPTOR, ALT_DONOR, coding, INTERNAL, intronic best transcript NM_003908	EIF2S2	0,73
stathmin 1; microRNA 3917	STMN1; MIR3917	0,73
SUB1 homolog, transcriptional regulator	SUB1	0,73
paired related homeobox 2	PRRX2	0,73
Transcript Identified by AceView, Entrez Gene ID(s) 7752	ZNF200	0,73
sterol carrier protein 2	SCP2	0,73
LY6/PLAUR domain containing 8	LYPD8	0,73
guanylate binding protein 1, interferon-inducible	GBP1	0,73
serine hydrolase-like (pseudogene)	SERHL	0,73
cornichon family AMPA receptor auxiliary protein 3	CNIH3	0,73
fermitin family member 2	FERMT2	0,73
SWIM-type zinc finger 7 associated protein 1	SWSAP1	0,73
transmembrane protein 203	TMEM203	0,73
angiogenic factor with G-patch and FHA domains 1	AGGF1	0,73
replication timing regulatory factor 1	RIF1	0,73
McKusick-Kaufman syndrome	MKKS	0,73
syntaxin binding protein 6 (amisyn)	STXBP6	0,73
purinergic receptor P2Y, G-protein coupled, 1	P2RY1	0,73
integrin beta 8	ITGB8	0,73
integrin beta 6	ITGB6	0,73

tachykinin, precursor 1	TAC1	0,73
zinc finger protein 439	ZNF439	0,73
churchill domain containing 1	CHURC1	0,73
protein phosphatase 2, regulatory subunit B, delta	PPP2R5D	0,72
serine/threonine/tyrosine interacting protein	STYX	0,72
taste receptor, type 2, member 46	TAS2R46	0,72
leucine-rich repeat kinase 1	LRRK1	0,72
proline-rich protein HaeIII subfamily 1	PRH1	0,72
surfeit 4	SURF4	0,72
zinc finger protein 577	ZNF577	0,72
MCM3AP antisense RNA 1	MCM3AP-AS1	0,72
vacuolar protein sorting 13 homolog B (yeast)	VPS13B	0,72
serine/threonine kinase 24	STK24	0,72
COP9 signalosome subunit 6	COPS6	0,72
methyltransferase like 25	METTL25	0,72
cilia and flagella associated protein 46	CFAP46	0,72
Memczak2013 ANTISENSE, coding, INTERNAL, intronic best transcript NM_198390	CMIP	0,72
AAR2 splicing factor homolog	AAR2	0,72
TBC1 domain family, member 30	TBC1D30	0,72
Transcript Identified by AceView, Entrez Gene ID(s) 9659	PDE4DIP	0,72
zinc finger and BTB domain containing 39	ZBTB39	0,72
dihydrofolate reductase; dihydrofolate reductase pseudogene 1	DHFR; DHFRP1	0,72
ZFP3 zinc finger protein	ZFP3	0,72
platelet-derived growth factor receptor, alpha polypeptide	PDGFRA	0,72
adducin 2 (beta)	ADD2	0,72
small integral membrane protein 20	SMIM20	0,72
Memczak2013 ANTISENSE, CDS, coding, INTERNAL best transcript NM_004396	DDX5	0,72
RAB40B, member RAS oncogene family	RAB40B	0,72
ATPase, Na+/K+ transporting, alpha 1 polypeptide	ATP1A1	0,72
MED14 opposite strand	MED14OS	0,72
lysine (K)-specific demethylase 5D	KDM5D	0,72
chromosome 1 open reading frame 186	C1orf186	0,72
Memczak2013 ANTISENSE, CDS, coding, INTERNAL best transcript NM_018031	WDR6	0,72
cyclin-dependent kinase 20	CDK20	0,72
UV stimulated scaffold protein A	UVSSA	0,72
flap structure-specific endonuclease 1	FEN1	0,72
Transcript Identified by AceView, Entrez Gene ID(s) 80305	TRABD	0,72
Morf4 family associated protein 1-like 1	MRFAP1L1	0,72

zinc finger, DHHC-type containing 15	ZDHHC15	0,72
uncharacterized LOC388282; novel transcript	LOC388282; CTD-2600O9.1	0,72
transient receptor potential cation channel, subfamily C, member 4	TRPC4	0,72
transmembrane protein 19	TMEM19	0,72
Transcript Identified by AceView, Entrez Gene ID(s) 10777	ARPP21	0,72
Transcript Identified by AceView, Entrez Gene ID(s) 23126	POGZ	0,72
penta-EF-hand domain containing 1	PEF1	0,72
regulator of calcineurin 2	RCAN2	0,72
coiled-coil domain containing 134	CCDC134	0,72
trichorhinophalangeal syndrome I	TRPS1	0,72
HFM1, ATP-dependent DNA helicase homolog	HFM1	0,72
eukaryotic translation initiation factor 4B	EIF4B	0,72
protein phosphatase 2A activator, regulatory subunit 4	PPP2R4	0,72
Yip1 domain family member 6	YIPF6	0,72
Transcript Identified by AceView, Entrez Gene ID(s) 2903	GRIN2A	0,72
nuclear pore complex interacting protein family, member A2	NPIPA2	0,72
Jeck2013 ALT_ACCEPTOR, ALT_DONOR, coding, INTERNAL, intronic, OVERLAPTX best transcript NM_024911	WLS	0,72
cell division cycle 26	CDC26	0,72
zinc finger protein 660	ZNF660	0,72
angiomotin; microRNA 4329	AMOT; MIR4329	0,72
phosphatidylinositol glycan anchor biosynthesis class H	PIGH	0,72
tetratricopeptide repeat domain 21B	TTC21B	0,72
zinc finger protein 326	ZNF326	0,72
wingless-type MMTV integration site family, member 3	WNT3	0,72
GDP dissociation inhibitor 2	GDI2	0,72
anomalous homeobox	ANHX	0,72
insulin-like growth factor 2 mRNA binding protein 2	IGF2BP2	0,72
dynamin 1-like	DNM1L	0,72
ribosomal protein S8; small nucleolar RNA, C/D box 55; small nucleolar RNA, C/D box 46; small nucleolar RNA, C/D box 38A; small nucleolar RNA, C/D box 38B	RPS8; SNORD55; SNORD46; SNORD38A; SNORD38B	0,72
caspase 9	CASP9	0,72
interleukin 18 binding protein	IL18BP	0,72
cell division cycle associated 4	CDCA4	0,72
small nuclear ribonucleoprotein polypeptide G	SNRPG	0,72
prostaglandin F2 receptor inhibitor	PTGFRN	0,72

ER lipid raft associated 1	ERLIN1	0,72
tetratricopeptide repeat domain 7A	TTC7A	0,72
cell death-inducing p53 target 1	CDIP1	0,72
olfactory receptor, family 8, subfamily B, member 8	OR8B8	0,72
zinc finger protein 181	ZNF181	0,72
FK506 binding protein like	FKBPL	0,72
Jeck2013 ALT_DONOR, coding, INTERNAL, intronic best transcript NM_016732	RALY	0,72
SH3 domain containing ring finger 3	SH3RF3	0,72
erythrocyte membrane protein band 4.1 like 5	EPB41L5	0,72
siah E3 ubiquitin protein ligase 2	SIAH2	0,72
Transcript Identified by AceView, Entrez Gene ID(s) 23184; novel transcript, antisense to CEMIP	RP11-351M8.2; MESDC2; RP11-351M8.1	0,72
SWI/SNF related, matrix associated, actin dependent regulator of chromatin, subfamily a, member 2	SMARCA2	0,72
speckle-type POZ protein-like	SPOPL	0,72
NCK-associated protein 5-like	NCKAP5L	0,72
erythrocyte membrane protein band 4.1-like 2	EPB41L2	0,72
zinc finger protein 660	ZNF660	0,72
zinc finger protein 32	ZNF32	0,72
coactivator-associated arginine methyltransferase 1	CARM1	0,72
Transcript Identified by AceView, Entrez Gene ID(s) 166378	SPATA5	0,72
protein associated with topoisomerase II homolog 1 (yeast)	PATL1	0,72
acyl-CoA binding domain containing 3	ACBD3	0,72
solute carrier family 22 (organic cation/carnitine transporter), member 5	SLC22A5	0,72
moesin	MSN	0,72
zinc finger protein 35	ZNF35	0,72
polymerase (RNA) I polypeptide D	POLR1D	0,72
Salzman2013 ANTISENSE, CDS, coding, INTERNAL, intronic, OVCODE, OVERLAPTX, OVEXON, UTR3 best transcript NM_015178; novel transcript, antisense to RHOBTB2	RP11-875O11.1; RHOBTB2	0,72
Homo sapiens TMLHE antisense RNA 1 (TMLHE-AS1), long non-coding RNA.; uncharacterized LOC101927830; Transcript Identified by AceView, Entrez Gene ID(s) 1193; novel transcript	TMLHE-AS1; LOC101927830; CLIC2; RP13-228J13.1	0,72
trans-golgi network protein 2	TGOLN2	0,72
signal recognition particle 68kDa	SRP68	0,72
regulator of chromosome condensation 1; small nucleolar RNA, H/ACA box 73A; small nucleolar RNA host gene 3; small nucleolar RNA, H/ACA box 73B	RCC1; SNORA73A; SNHG3; SNORA73B	0,72

inositol polyphosphate-5-phosphatase D	INPP5D	0,72
poly (ADP-ribose) glycohydrolase	PARG	0,72
histone cluster 2, H2aa4; histone cluster 2, H2aa3	HIST2H2AA4; HIST2H2AA3	0,72
leucine twenty homeobox	LEUTX	0,72
ribosomal protein L19	RPL19	0,72
Transcript Identified by AceView, Entrez Gene ID(s) 57448	BIRC6	0,72
nudix hydrolase 2	NUDT2	0,72
vesicle associated membrane protein 4	VAMP4	0,72
DnaJ (Hsp40) homolog, subfamily B, member 9	DNAJB9	0,72
histone cluster 1, H2aa	HIST1H2AA	0,72
Memczak2013 ANTISENSE, coding, INTERNAL, UTR3 best transcript NM_001195737	FAM213B	0,72
3(2), 5-bisphosphate nucleotidase 1	BPNT1	0,72
chromosome 12 open reading frame 10	C12orf10	0,72
hydroxycarboxylic acid receptor 2	HCAR2	0,72
ankyrin repeat and zinc finger domain containing 1	ANKZF1	0,72
SEC24 homolog C, COPII coat complex component	SEC24C	0,72
calcium channel flower domain containing 1	CACFD1	0,72
hypoxia up-regulated 1	HYOU1	0,72
TNF receptor-associated factor 7, E3 ubiquitin protein ligase	TRAF7	0,72
abhydrolase domain containing 17B	ABHD17B	0,72
prenyl (decaprenyl) diphosphate synthase, subunit 1	PDSS1	0,72
dehydrogenase/reductase (SDR family) member 4	DHRS4	0,72
patched 2	PTCH2	0,72
transmembrane protein 170A	TMEM170A	0,72
transforming growth factor beta receptor II	TGFBR2	0,72
Jeck2013 ANTISENSE, CDS, coding, INTERNAL, intronic, OVCODE, OVEXON best transcript NM_181453	GCC2	0,72
DNA (cytosine-5-)-methyltransferase 1	DNMT1	0,72
aarF domain containing kinase 2	ADCK2	0,72
Src homology 2 domain containing adaptor protein B	SHB	0,72
Zhang2013 ALT_ACCEPTOR, ALT_DONOR, coding, INTERNAL, intronic best transcript NM_001042576	RRBP1	0,72
RAN binding protein 2	RANBP2	0,72
reticulon 4 receptor	RTN4R	0,72
forkhead box D4-like 6	FOXD4L6	0,71
PRR5-ARHGAP8 readthrough	PRR5-ARHGAP8	0,71
calcium channel flower domain containing 1	CACFD1	0,71
family with sequence similarity 151, member B	FAM151B	0,71
surfeit 1	SURF1	0,71
aminopeptidase-like 1	NPEPL1	0,71
leucine rich repeat containing 27	LRRC27	0,71

claudin 20	CLDN20	0,71
mindbomb E3 ubiquitin protein ligase 2	MIB2	0,71
mitochondrial ribosomal protein S17	MRPS17	0,71
Transcript Identified by AceView, Entrez Gene ID(s) 55619	DOCK10	0,71
aminomethyltransferase; nicolin 1	AMT; NICN1	0,71
exocyst complex component 3	EXOC3	0,71
LETM1 domain containing 1	LETMD1	0,71
leucine rich repeat containing 34	LRRC34	0,71
proline-rich protein HaeIII subfamily 2	PRH2	0,71
Bardet-Biedl syndrome 2	BBS2	0,71
protocadherin beta 6	PCDHB6	0,71
leucine rich repeat containing 28	LRRC28	0,71
transcription termination factor, RNA polymerase II	TTF2	0,71
collagen, type IV, alpha 2	COL4A2	0,71
C7orf55-LUC7L2 readthrough; LUC7-like 2 pre-mRNA splicing factor; chromosome 7 open reading frame 55	C7orf55-LUC7L2; LUC7L2; C7orf55	0,71
zinc finger protein, FOG family member 1	ZFPM1	0,71
TYMS opposite strand	TYMSOS	0,71
ubiquinol-cytochrome c reductase binding protein	UQCRB	0,71
fer (fps/fes related) tyrosine kinase	FER	0,71
ZFP69 zinc finger protein	ZFP69	0,71
gamma-aminobutyric acid (GABA) A receptor, rho 3 (gene/pseudogene)	GABRR3	0,71
complexin 4	CPLX4	0,71
olfactory receptor, family 5, subfamily B, member 17	OR5B17	0,71
tripartite motif containing 24	TRIM24	0,71
family with sequence similarity 181, member A	FAM181A	0,71
chromosome 5 open reading frame 46	C5orf46	0,71
histidine triad nucleotide binding protein 2	HINT2	0,71
angiopoietin like 2	ANGPTL2	0,71
fibrillarin	FBL	0,71
nuclear pore complex interacting protein family, member A5	NPIPA5	0,71
glutathione S-transferase omega 1	GSTO1	0,71
ligase III, DNA, ATP-dependent	LIG3	0,71
ribonuclease P/MRP 25kDa subunit-like	RPP25L	0,71
sortilin-related VPS10 domain containing receptor 3	SORCS3	0,71
solute carrier family 50 (sugar efflux transporter), member 1	SLC50A1	0,71
growth factor, augmenter of liver regeneration	GFER	0,71
exportin 4	XPO4	0,71
RNA binding motif protein 43	RBM43	0,71
K(lysine) acetyltransferase 6B	KAT6B	0,71

Transcript Identified by AceView, Entrez Gene ID(s) 26064	RAI14	0,71
ADAMTS like 3	ADAMTSL3	0,71
peroxisomal biogenesis factor 10	PEX10	0,71
eukaryotic translation termination factor 1	ETF1	0,71
brain expressed, associated with NEDD4, 1	BEAN1	0,71
collagen beta(1-O)galactosyltransferase 1	COLGALT1	0,71
cysteine rich, DPF motif domain containing 1	CDPF1	0,71
zinc finger protein 546	ZNF546	0,71
family with sequence similarity 174, member A	FAM174A	0,71
BPI fold containing family B, member 6	BPIFB6	0,71
matrix-remodelling associated 5	MXRA5	0,71
nuclear pore complex interacting protein family, member A1	NPIPA1	0,71
ankyrin repeat domain 20 family, member A3; ankyrin repeat domain 20 family, member A2	ANKRD20A3; ANKRD20A2	0,71
regulator of chromosome condensation 2	RCC2	0,71
family with sequence similarity 53, member C	FAM53C	0,71
transmembrane protein 265; Snf2-related CREBBP activator protein	TMEM265; SRCAP	0,71
Transcript Identified by AceView, Entrez Gene ID(s) 10371	SEMA3A	0,71
serpin peptidase inhibitor, clade I (neuroserpin), member 1	SERPINI1	0,71
acyl-CoA synthetase bubblegum family member 2	ACSBG2	0,71
tetratricopeptide repeat domain 17	TTC17	0,71
heart development protein with EGF-like domains 1	HEG1	0,71
sorting nexin 22	SNX22	0,71
ring finger protein 183	RNF183	0,71
olfactory receptor, family 10, subfamily K, member 2	OR10K2	0,71
cutaneous T-cell lymphoma-associated antigen 1	CTAGE1	0,71
microtubule associated serine/threonine kinase 3	MAST3	0,71
calcium/calmodulin-dependent protein kinase II inhibitor 1	CAMK2N1	0,71
PGAM family member 5, serine/threonine protein phosphatase, mitochondrial	PGAM5	0,71
ZFP36 ring finger protein-like 2	ZFP36L2	0,71
KIAA0319-like	KIAA0319L	0,71
sprouty RTK signaling antagonist 1	SPRY1	0,71
defensin, beta 128	DEFB128	0,71
glycine N-methyltransferase	GNMT	0,71
SMG6 nonsense mediated mRNA decay factor	SMG6	0,71
testis expressed 35	TEX35	0,71
histone cluster 1, H1a	HIST1H1A	0,71
ribosomal protein L29	RPL29	0,71

zinc finger protein 550	ZNF550	0,71
cytochrome c oxidase subunit IV isoform 2 (lung)	COX4I2	0,71
TBC1 domain family, member 15	TBC1D15	0,71
proline rich 4 (lacrimal)	PRR4	0,71
SMYD family member 5	SMYD5	0,71
Jeck2013 ALT_ACCEPTOR, ALT_DONOR, coding, INTERNAL, intronic best transcript NM_024524	ATP13A3	0,71
ArfGAP with GTPase domain, ankyrin repeat and PH domain 5	AGAP5	0,71
KDEL (Lys-Asp-Glu-Leu) endoplasmic reticulum protein retention receptor 3	KDELR3	0,71
histone cluster 1, H3g	HIST1H3G	0,71
SLIT-ROBO Rho GTPase activating protein 2	SRGAP2	0,71
argonaute RISC catalytic component 1	ago-01	0,71
Memczak2013 ANTISENSE, CDS, coding, INTERNAL best transcript NM_004946	DOCK2	0,71
unc-50 homolog (C. elegans)	UNC50	0,71
kinesin light chain 4	KLC4	0,71
Jeck2013 ANTISENSE, coding, INTERNAL, intronic best transcript NM_003380	VIM	0,71
TRAF-interacting protein with forkhead-associated domain	TIFA	0,71
poly(A) binding protein, cytoplasmic 3	PABPC3	0,71
chromobox homolog 7	CBX7	0,71
hydroxymethylbilane synthase	HMBS	0,71
VPS29 retromer complex component	VPS29	0,71
TAO kinase 3	TAOK3	0,71
nuclear transport factor 2-like export factor 1	NXT1	0,71
Memczak2013 ALT_ACCEPTOR, ALT_DONOR, coding, INTERNAL, intronic best transcript NM_006828	ASCC3	0,71
solute carrier family 30 (zinc transporter), member 5	SLC30A5	0,71
guanine monophosphate synthase	GMPS	0,71
dual specificity phosphatase 16	DUSP16	0,71
tankyrase, TRF1-interacting ankyrin-related ADP-ribose polymerase	TNKS	0,71
ALG2, alpha-1,3/1,6-mannosyltransferase	ALG2	0,71
CCR4-NOT transcription complex subunit 8	CNOT8	0,71
heat shock 60kDa protein 1 (chaperonin)	HSPD1	0,71
lactate dehydrogenase A	LDHA	0,71
family with sequence similarity 124 member A	FAM124A	0,71
LEM domain containing 3	LEMD3	0,71
nucleoporin 188kDa	NUP188	0,71
chromosome 16 open reading frame 87	C16orf87	0,71
WD repeat domain 5	WDR5	0,71
zinc finger protein, FOG family member 2	ZFPM2	0,71

anoctamin 3	ANO3	0,71
RANBP2-like and GRIP domain containing 8; RANBP2-like and GRIP domain containing 6	RGPD8; RGPD6	0,71
LIM domain only 2 (rhombotin-like 1)	LMO2	0,71
signal recognition particle receptor, B subunit	SRPRB	0,71
N-acetylglucosamine-1-phosphate transferase, alpha and beta subunits	GNPTAB	0,71
RB-associated KRAB zinc finger; RBAK-RBAKDN readthrough; RBAK downstream neighbor (non-protein coding)	RBAK; RBAK-RBAKDN; RBAKDN	0,71
family with sequence similarity 168, member B	FAM168B	0,70
autism susceptibility candidate 2	AUTS2	0,70
sperm associated antigen 17	SPAG17	0,70
G protein-coupled receptor 141	GPR141	0,70
C-terminal binding protein 2	CTBP2	0,70
thymidylate synthetase	TYMS	0,70
carboxypeptidase E	CPE	0,70
F-box and leucine-rich repeat protein 12	FBXL12	0,70
TBC1 domain family, member 3G	TBC1D3G	0,70
CD93 molecule	CD93	0,70
LYR motif containing 5	LYRM5	0,70
zinc finger, DHHC-type containing 19	ZDHHC19	0,70
erythroid differentiation regulatory factor 1	EDRF1	0,70
microtubule associated protein tau	MAPT	0,70
RANBP2-like and GRIP domain containing 3	RGPD3	0,70
TNFAIP3 interacting protein 3	TNIP3	0,70
translocation associated membrane protein 1	TRAM1	0,70
olfactory receptor, family 2, subfamily M, member 7	OR2M7	0,70
ribosomal protein L35a	RPL35A	0,70
olfactory receptor, family 14, subfamily J, member 1	OR14J1	0,70
cytosolic iron-sulfur assembly component 1	CIAO1	0,70
N-myristoyltransferase 2	NMT2	0,70
troponin I type 2 (skeletal, fast)	TNNI2	0,70
ribosomal protein L23; small nucleolar RNA, H/ACA box 21	RPL23; SNORA21	0,70
regulator of G-protein signaling 18	RGS18	0,70
zinc finger protein 396	ZNF396	0,70
transducin (beta)-like 1, Y-linked	TBL1Y	0,70
Kv channel interacting protein 2	KCNIP2	0,70
zinc finger protein 277	ZNF277	0,70
QKI, KH domain containing, RNA binding	QKI	0,70
family with sequence similarity 134, member C	FAM134C	0,70
chromosome 7 open reading frame 49	C7orf49	0,70
phospholipid scramblase 1	PLSCR1	0,70
zinc finger protein 780A	ZNF780A	0,70

eukaryotic translation initiation factor 3, subunit J	EIF3J	0,70
glutamate receptor, ionotropic, N-methyl D-aspartate 2B	GRIN2B	0,70
RAB15 effector protein	REP15	0,70
retinol binding protein 2, cellular	RBP2	0,70
dedicator of cytokinesis 7	DOCK7	0,70
SRSF protein kinase 1	SRPK1	0,70
thymocyte nuclear protein 1	THYN1	0,70
TBC1 domain family, member 8 (with GRAM domain)	TBC1D8	0,70
Ly1 antibody reactive	LYAR	0,70
ADAM metallopeptidase with thrombospondin type 1 motif 13	ADAMTS13	0,70
cadherin-related family member 1	CDHR1	0,70
Ral GTPase activating protein, alpha subunit 2 (catalytic)	RALGAPA2	0,70
lymphocyte antigen 6 complex, locus H	LY6H	0,70
Fas-activated serine/threonine kinase	FASTK	0,70
zinc finger, RAN-binding domain containing 1	ZRANB1	0,70
integrin, beta 2 (complement component 3 receptor 3 and 4 subunit)	ITGB2	0,70
dedicator of cytokinesis 3	DOCK3	0,70
MAD2 mitotic arrest deficient-like 2 (yeast)	MAD2L2	0,70
chromosome 1 open reading frame 198	C1orf198	0,70
histone cluster 1, H2ak	HIST1H2AK	0,70
cancer/testis antigen 1B	CTAG1B	0,70
heterogeneous nuclear ribonucleoprotein F	HNRNPF	0,70
signal recognition particle 19kDa; zinc finger (CCCH type), RNA binding motif and serine/arginine rich 1	SRP19; ZRSR1	0,70
zinc finger protein 658	ZNF658	0,70
exocyst complex component 4	EXOC4	0,70
zinc finger and BTB domain containing 33	ZBTB33	0,70
Transcript Identified by AceView, Entrez Gene ID(s) 130507	UBR3	0,70
eukaryotic translation initiation factor 2B, subunit 5 epsilon, 82kDa	EIF2B5	0,70
PC4 and SFRS1 interacting protein 1	PSIP1	0,70
zinc activated ligand-gated ion channel	ZACN	0,70
nuclear receptor coactivator 1	NCOA1	0,70
Zhang2013 ALT_ACCEPTOR, ALT_DONOR, coding, INTERNAL, intronic, OVERLAPTX best transcript NM_017623	CNNM3	0,70
chromosome 11 open reading frame 57	C11orf57	0,70
NCK adaptor protein 2	NCK2	0,70
Transcript Identified by AceView, Entrez Gene ID(s) 85444	LRRCC1	0,70

zinc finger protein 443	ZNF443	0,70
uncharacterized LOC654841; Salzman2013 ANTISENSE, CDS, coding, INTERNAL, intronic, OVCODE, OVERLAPTX, OVEXON best transcript NM_000091; novel transcript, antisense to COL4A3	LOC654841; AC097662.2; COL4A3	0,70
Transcript Identified by AceView, Entrez Gene ID(s) 54556	ING3	0,70
mirror-image polydactyly 1	MIPOL1	0,70
tumor necrosis factor receptor superfamily, member 11a, NFKB activator	TNFRSF11A	0,70
leucine rich repeat containing 1	LRRC1	0,70
ER degradation enhancer, mannosidase alpha-like 2	EDEM2	0,70
heterogeneous nuclear ribonucleoprotein U-like 2	HNRNPUL2	0,70
M-phase phosphoprotein 8	MPHOSPH8	0,70
ORAI calcium release-activated calcium modulator 3	ORAI3	0,70
RAB11B, member RAS oncogene family	RAB11B	0,70
protein phosphatase 1, regulatory subunit 12B	PPP1R12B	0,70
Transcript Identified by AceView, Entrez Gene ID(s) 998	CDC42	0,70
mediator complex subunit 12	MED12	0,70
arylsulfatase B	ARSB	0,70
trimethyllysine hydroxylase, epsilon	TMLHE	0,70
Zhang2013 ALT_ACCEPTOR, ALT_DONOR, coding, INTERNAL, intronic best transcript NM_058230	ZNF354B	0,70
mitochondrial ribosomal protein L40	MRPL40	0,70
cornulin	CRNN	0,70
ribosomal protein S25	RPS25	0,70
coiled-coil domain containing 126	CCDC126	0,70
transmembrane p24 trafficking protein 4	TMED4	0,70
cystathionine gamma-lyase	CTH	0,70
PHD finger protein 2	PHF2	0,70
DnaJ (Hsp40) homolog, subfamily C , member 25	DNAJC25	0,70
inositol hexakisphosphate kinase 2	IP6K2	0,70
membrane associated guanylate kinase, WW and PDZ domain containing 1	MAGI1	0,70
cytochrome b561	CYB561	0,70
pre-mRNA processing factor 38B	PRPF38B	0,70
glutamate rich 3	ERICH3	0,70
etoposide induced 2.4	EI24	0,70
Nedd4 family interacting protein 2	NDFIP2	0,70
nascent polypeptide-associated complex alpha subunit 2	NACA2	0,70
TSEN34 tRNA splicing endonuclease subunit	TSEN34	0,70
chromosome 9 open reading frame 3; microRNA 24-1; microRNA 23b; microRNA 27b	C9orf3; MIR24-1; MIR23B; MIR27B	0,70
zinc finger protein 70	ZNF70	0,70

phosphatidylinositol glycan anchor biosynthesis class P	PIGP	0,70
major histocompatibility complex, class I, E	HLA-E	0,70
inositol polyphosphate-5-phosphatase F	INPP5F	0,70
solute carrier family 26 (anion exchanger), member 3	SLC26A3	0,70
ArfGAP with RhoGAP domain, ankyrin repeat and PH domain 2	ARAP2	0,70
olfactory receptor, family 12, subfamily D, member 1 (gene/pseudogene) [Source:HGNC Symbol;Acc:HGNC:8177]	OR12D1	0,70
poly-U binding splicing factor 60KDa	PUF60	0,70
zinc finger protein 780B	ZNF780B	0,70
capicua transcriptional repressor	CIC	0,70
ribosomal protein L7	RPL7	0,70
tubby bipartite transcription factor	TUB	0,70
solute carrier family 25 (mitochondrial carnitine/acylcarnitine carrier), member 29	SLC25A29	0,70
retinol dehydrogenase 13 (all-trans/9-cis)	RDH13	0,70
DNA cross-link repair 1A	DCLRE1A	0,70
Zhang2013 ALT_ACCEPTOR, ALT_DONOR, coding, INTERNAL, intronic best transcript NM_003086	SNAPC4	0,70
cyclin C	CCNC	0,70
family with sequence similarity 217, member B	FAM217B	0,70
glycerol kinase 5 (putative)	GK5	0,70
BRCA1 associated RING domain 1	BARD1	0,70
caseinolytic mitochondrial matrix peptidase chaperone subunit	CLPX	0,70
protein inhibitor of activated STAT 4	PIAS4	0,70
4-aminobutyrate aminotransferase	ABAT	0,70
fem-1 homolog a (C. elegans)	FEM1A	0,70
tryptophanyl tRNA synthetase 2, mitochondrial	WARS2	0,70
ubiquitin specific peptidase 1	USP1	0,70
phospholipid scramblase 2	PLSCR2	0,70
taperin	TPRN	0,70
glutamate receptor, metabotropic 2	GRM2	0,70
Memczak2013 ALT_ACCEPTOR, ALT_DONOR, coding, INTERNAL, intronic best transcript NM_002919	RFX3	0,70
C-type lectin domain family 18, member B	CLEC18B	0,70
fucosyltransferase 3 (galactoside 3(4)-L-fucosyltransferase, Lewis blood group)	FUT3	0,70
dipeptidyl-peptidase 8	DPP8	0,70
signal sequence receptor, alpha	SSR1	0,70
kelch-like family member 23	KLHL23	0,70
glutamate receptor, metabotropic 6	GRM6	0,70
zinc finger protein 639	ZNF639	0,70

Memczak2013 ANTISENSE, CDS, coding, INTERNAL best transcript NM_145295	ZNF627	0,70
solute carrier family 26 (anion exchanger), member 8	SLC26A8	0,70
Transcript Identified by AceView, Entrez Gene ID(s) 23122	CLASP2	0,70
SFT2 domain containing 1	SFT2D1	0,70
CREB regulated transcription coactivator 3	CRTC3	0,70
salt-inducible kinase 2	SIK2	0,70
TSEN54 tRNA splicing endonuclease subunit	TSEN54	0,70
DnaJ (Hsp40) homolog, subfamily C, member 9	DNAJC9	0,69
intermediate filament family orphan 2	IFFO2	0,69
PIGB opposite strand 1; RAB27A, member RAS oncogene family	PIGBOS1; RAB27A	0,69
Transcript Identified by AceView, Entrez Gene ID(s) 10426	TUBGCP3	0,69
HID1 domain containing	HID1	0,69
chromosome 9 open reading frame 116	C9orf116	0,69
ring finger protein 1	RING1	0,69
Transcript Identified by AceView, Entrez Gene ID(s) 10363	HMG20A	0,69
coatomer protein complex subunit gamma 1	COPG1	0,69
high mobility group nucleosomal binding domain 4	HMGN4	0,69
tachykinin 3	TAC3	0,69
PHD finger protein 10	PHF10	0,69
structural maintenance of chromosomes 3	SMC3	0,69
Memczak2013 ANTISENSE, coding, INTERNAL, intronic best transcript NM_014371; TEC	AC006128.2; AKAP8L	0,69
ribosomal protein S20; small nucleolar RNA, C/D box 54	RPS20; SNORD54	0,69
chromosome 1 open reading frame 116	C1orf116	0,69
nucleolar protein 7	NOL7	0,69
solute carrier family 10 (sodium/bile acid cotransporter), member 2	SLC10A2	0,69
coiled-coil domain containing 34	CCDC34	0,69
WAS protein family, member 1	WASF1	0,69
v-ets avian erythroblastosis virus E26 oncogene homolog 2	ETS2	0,69
Memczak2013 ALT_ACCEPTOR, ALT_DONOR, coding, INTERNAL, intronic best transcript NM_001163435	TBCK	0,69
ring finger protein 121	RNF121	0,69
trypsin domain containing 1	TYSND1	0,69
retinoblastoma 1	RB1	0,69
aquaporin 6, kidney specific	AQP6	0,69
leucine rich repeat containing 19	LRRC19	0,69
aryl hydrocarbon receptor interacting protein	AIP	0,69
inhibin alpha	INHA	0,69

serine/threonine kinase 16	STK16	0,69
GTF2I repeat domain containing 2B	GTF2IRD2B	0,69
uncharacterized LOC101928161; Salzman2013 ANTISENSE, coding, INTERNAL, intronic, OVERLAPTX best transcript NM_207363; novel transcript, antisense to NCKAP5; Transcript Identified by AceView	LOC101928161; AC010890.1; NCKAP5; spawfa	0,69
heterogeneous nuclear ribonucleoprotein A1 pseudogene 33; long intergenic non-protein coding RNA 842; annexin A8-like 1	HNRNPA1P33; LINC00842; ANXA8L1	0,69
family with sequence similarity 98, member A	FAM98A	0,69
G protein-coupled receptor 179	GPR179	0,69
olfactory receptor, family 2, subfamily T, member 29	OR2T29	0,69
uncharacterized LOC643355; putative novel transcript; Transcript Identified by AceView	LOC643355; RP11-88H9.2; toyfawbu	0,69
spectrin, beta, non-erythrocytic 4	SPTBN4	0,69
adenylosuccinate synthase like 1	ADSSL1	0,69
serine/arginine-rich splicing factor 1	SRSF1	0,69
ArfGAP with GTPase domain, ankyrin repeat and PH domain 1	AGAP1	0,69
H2A histone family, member Y	H2AFY	0,69
phosphoglycerate mutase 1 (brain)	PGAM1	0,69
disrupted in renal carcinoma 3	DIRC3	0,69
ATPase, class II, type 9B	ATP9B	0,69
Homo sapiens DiGeorge syndrome critical region gene 6 (DGCR6), mRNA.; protein DGCR6; Homo sapiens DiGeorge syndrome critical region gene 6, mRNA (cDNA clone MGC:54086 IMAGE:5229172), complete cds.; Protein DGCR6 [Source:UniProtKB/Swiss-Prot;Acc:Q14129]	DGCR6; LOC102724770	0,69
adenosylmethionine decarboxylase 1	AMD1	0,69
interferon-related developmental regulator 2	IFRD2	0,69
excision repair cross-complementation group 8	ERCC8	0,69
transmembrane protein 101	TMEM101	0,69
mitochondrial ribosomal protein L51	MRPL51	0,69
TSPY-like 1	TSPYL1	0,69
ubiquitin specific peptidase 9, X-linked	USP9X	0,69
transmembrane protein 74B	TMEM74B	0,69
spen family transcriptional repressor	SPEN	0,69
septin 2	sept-02	0,69
KIAA0895-like	KIAA0895L	0,69
zinc finger protein 566	ZNF566	0,69
rearranged L-myc fusion	RLF	0,69
NADH dehydrogenase (ubiquinone) 1 alpha subcomplex, 5	NDUFA5	0,69
ribosomal protein S3; small nucleolar RNA, C/D box 15A	RPS3; SNORD15A	0,69
eukaryotic translation initiation factor 1A, Y-linked	EIF1AY	0,69

Transcript Identified by AceView, Entrez Gene ID(s) 64062	RBM26	0,69
Parkinson disease 7 domain containing 1	PDDC1	0,69
Memczak2013 ANTISENSE, CDS, coding, INTERNAL best transcript NM_001127491	ITGB2	0,69
transmembrane protein 201	TMEM201	0,69
sulfatase 2	SULF2	0,69
solute carrier family 9, subfamily A (NHE6, cation proton antiporter 6), member 6	SLC9A6	0,69
Homo sapiens phosphoglycerate mutase 1 (brain), mRNA (cDNA clone MGC:8462 IMAGE:2821567), complete cds.; Homo sapiens phosphoglycerate mutase 1 (brain), mRNA (cDNA clone MGC:19732 IMAGE:3604026), complete cds.; Homo sapiens phosphoglycerate mutase 1 (brain), mRNA (cDNA clone MGC:15086 IMAGE:4299008), complete cds.; Homo sapiens phosphoglycerate mutase 1 (brain), mRNA (cDNA clone MGC:61519 IMAGE:5787678), complete cds.; Homo sapiens phosphoglycerate mutase 1 (brain), mRNA (cDNA clone MGC:71323 IMAGE:6584466), complete cds.; Homo sapiens phosphoglycerate mutase 1 (brain), mRNA (cDNA clone MGC:87445 IMAGE:5262935), complete cds.; Homo sapiens phosphoglycerate mutase 1 (brain), mRNA (cDNA clone MGC:88744 IMAGE:6297173), complete cds.; Homo sapiens phosphoglycerate mutase 1 (brain), mRNA (cDNA clone MGC:189702 IMAGE:8862699), complete cds.	PGAM1	0,69
acidic nuclear phosphoprotein 32 family member C	ANP32C	0,69
zinc finger protein 546	ZNF546	0,69
transmembrane protein 255A	TMEM255A	0,69
cytochrome c oxidase subunit IV isoform 1	COX4I1	0,69
spermatogenesis associated 1	SPATA1	0,69
Transcript Identified by AceView, Entrez Gene ID(s) 5108	PCM1	0,69
polycomb group ring finger 3	PCGF3	0,69
DDB1 and CUL4 associated factor 10	DCAF10	0,69
natriuretic peptide receptor 3	NPR3	0,69
striatin, calmodulin binding protein 4	STRN4	0,69
diacylglycerol kinase, delta 130kDa	DGKD	0,69
afamin	AFM	0,69
ALG1, chitobiosyldiphosphodolichol beta-mannosyltransferase; NAGPA antisense RNA 1	ALG1; NAGPA-AS1	0,69
ribosomal protein S6 kinase, 52kDa, polypeptide 1	RPS6KC1	0,69
calreticulin	CALR	0,69

leucine rich repeat containing 37B	LRRC37B	0,69
NIN1/RPN12 binding protein 1 homolog	NOB1	0,69
upstream transcription factor 1	USF1	0,69
olfactory receptor, family 2, subfamily AK, member 2	OR2AK2	0,69
uncharacterized LOC100130357; novel transcript, antisense to PHACTR1	LOC100130357; RP1-257A7.4	0,69
glutathione peroxidase 2	GPX2	0,69
ARV1 homolog, fatty acid homeostasis modulator	ARV1	0,69
prolyl 4-hydroxylase, beta polypeptide	P4HB	0,69
solute carrier family 2 (facilitated glucose transporter), member 6 [Source:HGNC Symbol;Acc:HGNC:11011]	SLC2A6	0,69
nicastrin	NCSTN	0,69
collagen, type XV, alpha 1	COL15A1	0,69
diphosphoinositol pentakisphosphate kinase 2	PPIP5K2	0,69
NUF2, NDC80 kinetochore complex component	NUF2	0,69
zinc finger protein 747	ZNF747	0,69
Transcript Identified by AceView, Entrez Gene ID(s) 8502	PKP4	0,69
pancreatic lipase-related protein 2	PNLIPRP2	0,69
aconitase 1, soluble	ACO1	0,69
stromal antigen 1	STAG1	0,69
histidine triad nucleotide binding protein 3	HINT3	0,69
transmembrane protein 70; ribosomal protein S20 pseudogene 21	TMEM70; RPS20P21	0,69
zinc finger and BTB domain containing 7B	ZBTB7B	0,69
pumilio RNA binding family member 2	PUM2	0,69
BCL2-associated athanogene	BAG1	0,69
CCR4-NOT transcription complex subunit 2	CNOT2	0,69
HESX homeobox 1	HESX1	0,69
Transcript Identified by AceView, Entrez Gene ID(s) 23126	POGZ	0,69
La ribonucleoprotein domain family, member 1B	LARP1B	0,69
GATA zinc finger domain containing 1	GATAD1	0,69
YLP motif containing 1	YLPM1	0,69
keratin associated protein 10-7; keratin associated protein 10-8	KRTAP10-7; KRTAP10-8	0,69
transmembrane protein 184A	TMEM184A	0,69
SH3 and SYLF domain containing 1	SH3YL1	0,69
LysM, putative peptidoglycan-binding, domain containing 4	LYSMD4	0,69
replication initiator 1	REPIN1	0,69
exosome component 4; microRNA 6847	EXOSC4; MIR6847	0,69
procollagen-lysine, 2-oxoglutarate 5-dioxygenase 3	PLOD3	0,69

translocase of outer mitochondrial membrane 70 homolog A (S. cerevisiae)	TOMM70A	0,69
ATPase family, AAA domain containing 3A	ATAD3A	0,68
polypeptide N-acetylgalactosaminyltransferase 2	GALNT2	0,68
transmembrane 9 superfamily member 1	TM9SF1	0,68
ribosomal protein S27a	RPS27A	0,68
nuclear factor related to kappaB binding protein	NFRKB	0,68
Salzman2013 ANNOTATED, CDS, coding, INTERNAL, OVCODE, OVEXON best transcript NM_001190810; centaurin, gamma-like family, member 11 pseudogene [Source:HGNC Symbol;Acc:HGNC:23660]	AGAP9; CTGLF11P	0,68
FGFR1 oncogene partner 2	FGFR1OP2	0,68
nicotinamide nucleotide adenylyltransferase 3	NMNAT3	0,68
runt-related transcription factor 1	RUNX1	0,68
ATP binding cassette subfamily C member 5	ABCC5	0,68
ATPase, class VI, type 11B	ATP11B	0,68
speckle-type POZ protein	SPOP	0,68
damage-specific DNA binding protein 1	DDB1	0,68
ankyrin repeat domain 36B	ANKRD36B	0,68
scaffold attachment factor B	SAFB	0,68
elastin microfibril interfacer 1	EMILIN1	0,68
protocadherin beta 2	PCDHB2	0,68
forkhead box O1	FOXO1	0,68
synapse defective 1, Rho GTPase, homolog 2 (C. elegans)	SYDE2	0,68
multimerin 1	MMRN1	0,68
olfactory receptor, family 8, subfamily G, member 2	OR8G2	0,68
G protein-coupled receptor kinase 4	GRK4	0,68
Ets2 repressor factor	ERF	0,68
keratinocyte differentiation factor 1	KDF1	0,68
CTBP1 antisense RNA 2 (head to head)	CTBP1-AS2	0,68
serologically defined colon cancer antigen 3	SDCCAG3	0,68
occludin/ELL domain containing 1	OCEL1	0,68
c-fos induced growth factor (vascular endothelial growth factor D)	FIGF	0,68
Rho guanine nucleotide exchange factor 11	ARHGEF11	0,68
acyl-CoA thioesterase 13	ACOT13	0,68
NmrA-like family domain containing 1	NMRAL1	0,68
nuclear pore complex interacting protein family, member A3	NPIPA3	0,68
natriuretic peptide receptor 2	NPR2	0,68
peroxisome proliferator-activated receptor gamma	PPARG	0,68
family with sequence similarity 198, member B	FAM198B	0,68
D-2-hydroxyglutarate dehydrogenase	D2HGDH	0,68
zinc finger protein 22	ZNF22	0,68

Kruppel-like factor 3 (basic)	KLF3	0,68
beta-1,3-N-acetylgalactosaminyltransferase 2	B3GALNT2	0,68
surfeit 1	SURF1	0,68
embigin	EMB	0,68
chromosome 6 open reading frame 120	C6orf120	0,68
Memczak2013 ANTISENSE, coding, INTERNAL, intronic best transcript NM_005225	E2F1	0,68
elongator acetyltransferase complex subunit 5	ELP5	0,68
F-box protein 25	FBXO25	0,68
oxidation resistance 1	OXR1	0,68
spermatogenesis associated 7	SPATA7	0,68
olfactory receptor, family 2, subfamily T, member 6	OR2T6	0,68
hepatoma-derived growth factor	HDGF	0,68
chromosome 10 open reading frame 111	C10orf111	0,68
SWI/SNF related, matrix associated, actin dependent regulator of chromatin, subfamily b, member 1	SMARCB1	0,68
coenzyme Q3 methyltransferase	COQ3	0,68
NEDD8 activating enzyme E1 subunit 1	NAE1	0,68
SAC3 domain containing 1	SAC3D1	0,68
dpy-19-like 4 (C. elegans)	DPY19L4	0,68
vomeronasal 1 receptor 1	VN1R1	0,68
Memczak2013 ANTISENSE, CDS, coding, INTERNAL best transcript NM_006184	NUCB1	0,68
leucyl-tRNA synthetase 2, mitochondrial	LARS2	0,68
ribophorin I	RPN1	0,68
intraflagellar transport 20	IFT20	0,68
ciliary neurotrophic factor	CNTF	0,68
nonhomologous end-joining factor 1	NHEJ1	0,68
cancer susceptibility candidate 5	CASC5	0,68
malonyl-CoA decarboxylase	MLYCD	0,68
phosphatidylinositol glycan anchor biosynthesis class M	PIGM	0,68
phosphodiesterase 7A	PDE7A	0,68
pre-B-cell leukemia homeobox interacting protein 1	PBXIP1	0,68
acidic nuclear phosphoprotein 32 family member B	ANP32B	0,68
inositol polyphosphate-5-phosphatase J	INPP5J	0,68
adenosine deaminase, RNA-specific	ADAR	0,68
lysine (K)-specific demethylase 3B	KDM3B	0,68
cathepsin K	CTSK	0,68
solute carrier family 2 (facilitated glucose transporter), member 6 [Source:HGNC Symbol;Acc:HGNC:11011]	SLC2A6	0,68
receptor (chemosensory) transporter protein 5 (putative)	RTP5	0,68
torsin family 3, member A	TOR3A	0,68
alkylglycerol monooxygenase	AGMO	0,68
inositol polyphosphate-5-phosphatase A	INPP5A	0,68

Memczak2013 ANTISENSE, coding, INTERNAL, intronic best transcript NM_001161841	SULF2	0,68
phosphomannomutase 1	PMM1	0,68
zinc finger protein 830	ZNF830	0,68
purine-rich element binding protein A	PURA	0,68
cytohesin 1	CYTH1	0,68
musashi RNA binding protein 1	MSI1	0,68
adaptor-related protein complex 5, beta 1 subunit	AP5B1	0,68
myeloid/lymphoid or mixed-lineage leukemia; translocated to, 3	MLLT3	0,68
heterogeneous nuclear ribonucleoprotein R	HNRNPR	0,68
mitochondrial ribosomal protein S27	MRPS27	0,68
UDP-glucuronate decarboxylase 1	UXS1	0,68
sphingosine kinase 1	SPHK1	0,68
hexamethylene bis-acetamide inducible 2	HEXIM2	0,68
sterile alpha motif domain containing 9	SAMD9	0,68
neural retina leucine zipper	NRL	0,68
bone morphogenetic protein receptor type IB	BMPR1B	0,68
Rho guanine nucleotide exchange factor (GEF) 12	ARHGEF12	0,68
sideroflexin 1	SFXN1	0,68
Sin3A associated protein 30kDa	SAP30	0,68
olfactory receptor, family 2, subfamily G, member 2	OR2G2	0,68
defensin, beta 131	DEFB131	0,68
thioesterase superfamily member 5	THEM5	0,68
Transcript Identified by AceView, Entrez Gene ID(s) 220972	MARCH8	0,68
TBC1 domain family, member 10B	TBC1D10B	0,68
RAB28, member RAS oncogene family	RAB28	0,68
eukaryotic translation initiation factor 4E binding protein 2	EIF4EBP2	0,68
histone cluster 1, H3e	HIST1H3E	0,68
family with sequence similarity 76, member A	FAM76A	0,68
spermatogenesis associated 18	SPATA18	0,68
tissue factor pathway inhibitor (lipoprotein-associated coagulation inhibitor)	TFPI	0,68
cellular repressor of E1A-stimulated genes 2	CREG2	0,68
F-box and WD repeat domain containing 4	FBXW4	0,68
ribosomal protein, large, P0	RPLP0	0,68
mediator complex subunit 12 like	MED12L	0,68
calcium/calmodulin-dependent protein kinase I	CAMK1	0,68
solute carrier family 25 (mitochondrial carrier; adenine nucleotide translocator), member 4	SLC25A4	0,68
transmembrane protein 60	TMEM60	0,68
pumilio RNA binding family member 1	PUM1	0,68
lin-7 homolog C (C. elegans)	LIN7C	0,68

adhesion G protein-coupled receptor L2	ADGRL2	0,68
gonadotropin releasing hormone 2	GNRH2	0,68
tigger transposable element derived 4	TIGD4	0,68
sulfotransferase family 4A member 1	SULT4A1	0,68
immunity-related GTPase family, Q	IRGQ	0,68
thioredoxin-related transmembrane protein 1	TMX1	0,68
GrpE-like 2, mitochondrial (E. coli)	GRPEL2	0,68
taste receptor, type 2, member 50	TAS2R50	0,68
voltage-dependent anion channel 1	VDAC1	0,68
mitogen-activated protein kinase kinase kinase 3	MAP3K3	0,67
importin 8	IPO8	0,67
solute carrier family 39 (zinc transporter), member 1	SLC39A1	0,67
acyl-CoA dehydrogenase family, member 9	ACAD9	0,67
heat shock 70kDa protein 5 (glucose-regulated protein, 78kDa)	HSPA5	0,67
cell growth regulator with ring finger domain 1	CGRRF1	0,67
lysine (K)-specific demethylase 5B	KDM5B	0,67
free fatty acid receptor 3	FFAR3	0,67
beta-defensin 131-like	LOC100129216	0,67
phosphomannomutase 2	PMM2	0,67
cytochrome P450, family 21, subfamily A, polypeptide 1 pseudogene; cytochrome P450, family 21, subfamily A, polypeptide 2	CYP21A1P; CYP21A2	0,67
adenylate kinase 3	AK3	0,67
histone cluster 1, H2bc	HIST1H2BC	0,67
RANBP2-like and GRIP domain containing 6; RANBP2-like and GRIP domain containing 5	RGPD6; RGPD5	0,67
acyl-CoA dehydrogenase family, member 10	ACAD10	0,67
Transcript Identified by AceView, Entrez Gene ID(s) 84909; novel transcript	C9orf3; RP11-49O14.3	0,67
sorting nexin 14	SNX14	0,67
coiled-coil domain containing 64B	CCDC64B	0,67
THO complex 2	THOC2	0,67
PAF1 homolog, Paf1/RNA polymerase II complex component	PAF1	0,67
chromosome 7 open reading frame 60	C7orf60	0,67
nudix hydrolase 4 pseudogene 1; nudix hydrolase 4 pseudogene 2; nudix hydrolase 4	NUDT4P1; NUDT4P2; NUDT4	0,67
nudix hydrolase 4; nudix hydrolase 4 pseudogene 1	NUDT4; NUDT4P1	0,67
phosphoglycerate kinase 1	PGK1	0,67
tetratricopeptide repeat domain 9C	TTC9C	0,67
zinc finger protein 780B	ZNF780B	0,67
SEC31 homolog B, COPII coat complex component	SEC31B	0,67

protein tyrosine phosphatase, non-receptor type 18 (brain-derived)	PTPN18	0,67
protein kinase, interferon-inducible double stranded RNA dependent activator	PRKRA	0,67
xyloside xylosyltransferase 1	XXYLT1	0,67
H2B histone family, member M	H2BFM	0,67
SCY1-like, kinase-like 1	SCYL1	0,67
inositol polyphosphate-5-phosphatase D	INPP5D	0,67
acyl-CoA synthetase long-chain family member 6	ACSL6	0,67
olfactory receptor, family 2, subfamily T, member 3	OR2T3	0,67
bromodomain adjacent to zinc finger domain 1B	BAZ1B	0,67
leucine rich repeat containing 39	LRRC39	0,67
macrophage migration inhibitory factor (glycosylation-inhibiting factor)	MIF	0,67
	LRIG1	0,67
SUMO1/sentrin specific peptidase 1	SENP1	0,67
surfeit 1	SURF1	0,67
LDL receptor related protein 1	LRP1	0,67
Transcript Identified by AceView, Entrez Gene ID(s) 7073	TIAL1	0,67
serine racemase	SRR	0,67
inter-alpha-trypsin inhibitor heavy chain family, member 5	ITIH5	0,67
Memczak2013 ALT_ACCEPTOR, ALT_DONOR, coding, INTERNAL, intronic best transcript NM_005534	IFNGR2	0,67
protein kinase C substrate 80K-H	PRKCSH	0,67
golgi-associated PDZ and coiled-coil motif containing	GOPC	0,67
5-hydroxymethylcytosine (hmC) binding, ES cell-specific	HMCES	0,67
F-box protein 34	FBXO34	0,67
activating transcription factor 7 interacting protein 2	ATF7IP2	0,67
UXT antisense RNA 1	UXT-AS1	0,67
FERM, ARH/RhoGEF and pleckstrin domain protein 1; microRNA 3170	FARP1; MIR3170	0,67
transmembrane protein 5	TMEM5	0,67
thymine DNA glycosylase	TDG	0,67
SIN3 transcription regulator family member A	SIN3A	0,67
cleavage and polyadenylation specific factor 6	CPSF6	0,67
high mobility group nucleosome binding domain 1	HMGN1	0,67
golgin A5	GOLGA5	0,67
zinc finger protein 780B	ZNF780B	0,67
protein kinase, cAMP-dependent, catalytic, alpha	PRKACA	0,67
lysine (K)-specific methyltransferase 2A	KMT2A	0,67
coiled-coil domain containing 121	CCDC121	0,67
mitochondrial ribosomal protein L30	MRPL30	0,67

Transcript Identified by AceView, Entrez Gene ID(s) 83941	TM2D1	0,67
kinesin family member 15	KIF15	0,67
peroxiredoxin 3	PRDX3	0,67
DCN1, defective in cullin neddylation 1, domain containing 1	DCUN1D1	0,67
SIX homeobox 1	SIX1	0,67
REX4 homolog, 3'-5' exonuclease [Source:HGNC Symbol;Acc:HGNC:12820]	REXO4	0,67
RNA polymerase II associated protein 3	RPAP3	0,67
zinc finger protein 689	ZNF689	0,67
histone cluster 1, H2be	HIST1H2BE	0,67
polymerase (RNA) III (DNA directed) polypeptide B	POLR3B	0,67
WWC2 antisense RNA 2	WWC2-AS2	0,67
ras homolog family member T2	RHOT2	0,67
OTU deubiquitinase with linear linkage specificity	OTULIN	0,67
gremlin 1, DAN family BMP antagonist [Source:HGNC Symbol;Acc:HGNC:2001]	GREM1	0,67
F-box protein, helicase, 18	FBXO18	0,67
tubulin tyrosine ligase-like family member 5	TTLL5	0,67
chromosome 22 open reading frame 29; guanine nucleotide binding protein (G protein), beta polypeptide 1-like	C22orf29; GNB1L	0,67
hydroxymethylbilane synthase	HMBS	0,67
N(alpha)-acetyltransferase 60, NatF catalytic subunit	NAA60	0,67
transcriptional adaptor 2B	TADA2B	0,67
N-myc downstream regulated 1	NDRG1	0,67
Jeck2013 ANTISENSE, coding, INTERNAL, intronic best transcript NM_000088	COL1A1	0,67
zinc finger protein 253	ZNF253	0,67
synaptic vesicle glycoprotein 2C	SV2C	0,67
hyaluronoglucosaminidase 2	HYAL2	0,67
Zhang2013 ALT_ACCEPTOR, ALT_DONOR, coding, INTERNAL, intronic, OVERLAPTX, OVEXON best transcript NM_003315	DNAJC7	0,67
serine/arginine-rich splicing factor 8	SRSF8	0,67
chemokine (C-X-C motif) ligand 5	CXCL5	0,67
HECT domain containing E3 ubiquitin protein ligase 2	HECTD2	0,67
histone cluster 1, H4i	HIST1H4I	0,67
RPA interacting protein	RPAIN	0,67
olfactory receptor, family 4, subfamily F, member 17	OR4F17	0,67
polycystin (PKD) family receptor for egg jelly	PKDREJ	0,67
WD repeat domain 90	WDR90	0,67
fibrillarin	FBL	0,67

Transcript Identified by AceView, Entrez Gene ID(s) 8438	RAD54L	0,67
ST3 beta-galactoside alpha-2,3-sialyltransferase 2	ST3GAL2	0,67
zinc finger protein 780A	ZNF780A	0,67
von Willebrand factor A domain containing 9	VWA9	0,67
t-complex 10-like	TCP10L	0,67
Transcript Identified by AceView, Entrez Gene ID(s) 10558	SPTLC1	0,67
RAD51 paralog B	RAD51B	0,67
PR domain containing 8	PRDM8	0,67
surfeit 4	SURF4	0,67
nucleobindin 1	NUCB1	0,67
regulator of chromosome condensation 2	RCC2	0,67
kelch-like family member 25	KLHL25	0,67
peptidylprolyl isomerase domain and WD repeat containing 1	PPWD1	0,67
chromosome 10 open reading frame 128	C10orf128	0,67
phosphoprotein membrane anchor with glycosphingolipid microdomains 1	PAG1	0,67
AFG3-like AAA ATPase 2	AFG3L2	0,67
cytochrome P450, family 2, subfamily U, polypeptide 1	CYP2U1	0,67
ELAV like RNA binding protein 1	ELAVL1	0,67
protein phosphatase 1, regulatory subunit 9A	PPP1R9A	0,67
CD180 molecule	CD180	0,67
cytokine receptor-like factor 1	CRLF1	0,66
transmembrane protease, serine 3	TMPRSS3	0,66
hexosaminidase (glycosyl hydrolase family 20, catalytic domain) containing	HEXDC	0,66
Leber congenital amaurosis 5	LCA5	0,66
RWD domain containing 1	RWDD1	0,66
chromosome 8 open reading frame 59	C8orf59	0,66
ubiquitin protein ligase E3 component n-recognin 3 (putative)	UBR3	0,66
catenin (cadherin-associated protein), beta 1	CTNNB1	0,66
dendritic cell-associated nuclear protein	DCANP1	0,66
transmembrane and coiled-coil domains 6	TMCO6	0,66
Memczak2013 ALT_ACCEPTOR, ALT_DONOR, coding, INTERNAL, intronic best transcript NM_006667	PGRMC1	0,66
oxidoreductase NAD-binding domain containing 1	OXNAD1	0,66
HECT domain containing E3 ubiquitin protein ligase 4; microRNA 6861	HECTD4; MIR6861	0,66
protocadherin beta 12	PCDHB12	0,66
Transcript Identified by AceView, Entrez Gene ID(s) 31	ACACA	0,66
beta 3-glucosyltransferase	B3GLCT	0,66
N-myc (and STAT) interactor	NMI	0,66

mannosidase, alpha, class 2B, member 2	MAN2B2	0,66
protein phosphatase, Mg2+/Mn2+ dependent, 1F	PPM1F	0,66
fibrillarin	FBL	0,66
pseudouridylate synthase-like 1	PUSL1	0,66
muscle-related coiled-coil protein	MURC	0,66
Transcript Identified by AceView, Entrez Gene ID(s) 8148	TAF15	0,66
Transcript Identified by AceView, Entrez Gene ID(s) 10019	SH2B3	0,66
TSPY-like 6	TSPYL6	0,66
COMM domain containing 10	COMMD10	0,66
ribokinase; BRE antisense RNA 1	RBKS; BRE-AS1	0,66
guanosine monophosphate reductase 2	GMPR2	0,66
protein phosphatase, Mg2+/Mn2+ dependent, 1D	PPM1D	0,66
ISL LIM homeobox 1	ISL1	0,66
transmembrane protein 30C	TMEM30C	0,66
spermatogenesis associated 5-like 1	SPATA5L1	0,66
F-box protein 11	FBXO11	0,66
opsin 1 (cone pigments), medium-wave-sensitive 2	OPN1MW2	0,66
LIM and senescent cell antigen-like domains 1	LIMS1	0,66
VANGL planar cell polarity protein 1	VANGL1	0,66
hydroxymethylbilane synthase	HMBS	0,66
signal transducer and activator of transcription 3 (acute-phase response factor)	STAT3	0,66
zinc finger protein 548	ZNF548	0,66
epithelial cell adhesion molecule	EPCAM	0,66
BMP and activin membrane-bound inhibitor	BAMBI	0,66
kinesin family member 15	KIF15	0,66
CCZ1 homolog B, vacuolar protein trafficking and biogenesis associated	CCZ1B	0,66
retinoic acid induced 1	RAI1	0,66
cilia and flagella associated protein 100	CFAP100	0,66
ankyrin repeat and SOCS box containing 1	ASB1	0,66
translocase of inner mitochondrial membrane 13 homolog (yeast)	TIMM13	0,66
cochlin	COCH	0,66
3-hydroxyisobutyryl-CoA hydrolase	HIBCH	0,66
histone cluster 1, H4g	HIST1H4G	0,66
	PRAMEF9	0,66
NOP2/Sun domain family, member 4	NSUN4	0,66
zinc finger protein 451	ZNF451	0,66
chloride intracellular channel 4	CLIC4	0,66
inhibitor of growth family member 4	ING4	0,66
chromosome 4 open reading frame 3	C4orf3	0,66
transcription factor A, mitochondrial	TFAM	0,66

diacylglycerol kinase, delta 130kDa	DGKD	0,66
basal body orientation factor 1	BBOF1	0,66
glutaryl-CoA dehydrogenase	GCDH	0,66
olfactory receptor, family 7, subfamily G, member 1	OR7G1	0,66
biogenesis of lysosomal organelles complex-1, subunit 5, muted	BLOC1S5	0,66
claudin 15	CLDN15	0,66
calcium/calmodulin-dependent protein kinase II gamma	CAMK2G	0,66
TRAF-type zinc finger domain containing 1	TRAFD1	0,66
solute carrier family 38, member 2	SLC38A2	0,66
hydroxyacid oxidase (glycolate oxidase) 1	HAO1	0,66
golgin A4	GOLGA4	0,66
colorectal cancer associated 1	COLCA1	0,66
5-3 exoribonuclease 1	XRN1	0,66
TAF5 RNA polymerase II, TATA box binding protein (TBP)-associated factor, 100kDa	TAF5	0,66
F-box protein 39	FBXO39	0,66
Transcript Identified by AceView, Entrez Gene ID(s) 9759	HDAC4	0,66
EF-hand calcium binding domain 2	EFCAB2	0,66
zinc finger protein 821	ZNF821	0,66
Transcript Identified by AceView, Entrez Gene ID(s) 3988	LIPA	0,66
growth differentiation factor 11	GDF11	0,66
ELOVL fatty acid elongase 6	ELOVL6	0,66
A kinase (PRKA) anchor protein 7	AKAP7	0,66
protein arginine methyltransferase 2	PRMT2	0,66
inhibitor of growth family member 1	ING1	0,66
kinesin family member 15	KIF15	0,66
dual specificity phosphatase 22	DUSP22	0,66
Memczak2013 ANTISENSE, coding, INTERNAL, intronic best transcript NM_020821	VPS13C	0,66
cytochrome P450, family 7, subfamily A, polypeptide 1	CYP7A1	0,66
lysine-rich coiled-coil 1	KRCC1	0,66
anaphase promoting complex subunit 4	ANAPC4	0,66
SWI/SNF related, matrix associated, actin dependent regulator of chromatin, subfamily e, member 1	SMARCE1	0,66
inositol polyphosphate-5-phosphatase D	INPP5D	0,66
excision repair cross-complementation group 5	ERCC5	0,66
GCN1 eIF2 alpha kinase activator homolog; microRNA 4498	GCN1; MIR4498	0,66
phosphoinositide kinase, FYVE finger containing	PIKFYVE	0,66
galactosamine (N-acetyl)-6-sulfatase	GALNS	0,66
SREBF chaperone	SCAP	0,66
GC-rich promoter binding protein 1-like 1	GPBP1L1	0,66

zinc finger protein 24	ZNF24	0,66
intraflagellar transport 74	IFT74	0,66
cell division cycle associated 8	CDCA8	0,66
nuclear receptor subfamily 1, group D, member 2	NR1D2	0,66
UBA domain containing 2	UBAC2	0,66
spermatogenesis associated 2-like	SPATA2L	0,66
long intergenic non-protein coding RNA 1465	LINC01465	0,66
Zhang2013 ALT_ACCEPTOR, ALT_DONOR, coding, INTERNAL, intronic best transcript NM_015179	RRP12	0,66
GrpE-like 1, mitochondrial (E. coli)	GRPEL1	0,66
TAR (HIV-1) RNA binding protein 1	TARBP1	0,66
ribosomal protein L5; small nucleolar RNA, C/D box 21; small nucleolar RNA, H/ACA box 66	RPL5; SNORD21; SNORA66	0,66
SRSF protein kinase 2	SRPK2	0,66
neuralized E3 ubiquitin protein ligase 4	NEURL4	0,66
taspase 1	TASP1	0,66
unc-51 like autophagy activating kinase 2	ULK2	0,66
EF-hand calcium binding domain 12	EFCAB12	0,66
HECT domain and ankyrin repeat containing E3 ubiquitin protein ligase 1	HACE1	0,66
leukocyte immunoglobulin-like receptor, subfamily A (with TM domain), member 4	LILRA4	0,66
thiosulfate sulfurtransferase (rhodanese)-like domain containing 1	TSTD1	0,66
MAP7 domain containing 2	MAP7D2	0,66
cytochrome c-1	CYC1	0,66
negative elongation factor complex member C/D	NELFCD	0,66
Jeck2013 ALT_ACCEPTOR, ALT_DONOR, coding, INTERNAL, intronic best transcript NM_020177	FEM1C	0,66
EGF containing fibulin-like extracellular matrix protein 2	EFEMP2	0,66
fucosidase, alpha-L- 1, tissue	FUCA1	0,66
VPS26 retromer complex component B	VPS26B	0,66
N-acetylneuraminic acid phosphatase	NANP	0,66
activating transcription factor 4	ATF4	0,66
Transcript Identified by AceView, Entrez Gene ID(s) 254251	LCORL	0,66
signal transducing adaptor family member 2	STAP2	0,66
K(lysine) acetyltransferase 5	KAT5	0,66
pyruvate carboxylase	PC	0,66
chromosome 16 open reading frame 54	C16orf54	0,66
WD repeat domain, phosphoinositide interacting 1	WIPI1	0,66
stromal antigen 3	STAG3	0,66
EH domain binding protein 1	EHBP1	0,66
spermatogenesis associated, serine-rich 2	SPATS2	0,66
zinc finger, FYVE domain containing 21	ZFYVE21	0,66

MANSC domain containing 1	MANSC1	0,65
PAX interacting (with transcription-activation domain) protein 1	PAXIP1	0,65
ligase I, DNA, ATP-dependent	LIG1	0,65
mitochondrial fission regulator 1	MTFR1	0,65
formiminotransferase cyclodeaminase N-terminal like	FTCDNL1	0,65
nudix hydrolase 12	NUDT12	0,65
DEAH (Asp-Glu-Ala-His) box helicase 15	DHX15	0,65
establishment of sister chromatid cohesion N-acetyltransferase 1	ESCO1	0,65
cathepsin H	CTSH	0,65
Transcript Identified by AceView, Entrez Gene ID(s) 8470	SORBS2	0,65
uracil phosphoribosyltransferase (FUR1) homolog (S. cerevisiae)	UPRT	0,65
apolipoprotein L, 3	APOL3	0,65
cullin 7	CUL7	0,65
NIPA-like domain containing 2	NIPAL2	0,65
COBW domain containing 5	CBWD5	0,65
sorting nexin 13	SNX13	0,65
proline-rich protein HaeIII subfamily 1; taste receptor, type 2, member 14; proline rich 4 (lacrimal)	PRH1; TAS2R14; PRR4	0,65
transcription elongation factor B (SIII), polypeptide 2 (18kDa, elongin B)	TCEB2	0,65
solute carrier family 2 (facilitated glucose transporter), member 6 [Source:HGNC Symbol;Acc:HGNC:11011]	SLC2A6	0,65
platelet factor 4	PF4	0,65
THAP domain containing 5	THAP5	0,65
Transcript Identified by AceView, Entrez Gene ID(s) 6872	TAF1	0,65
fibromodulin	FMOD	0,65
potassium voltage-gated channel, modifier subfamily S, member 3	KCNS3	0,65
Memczak2013 ANTISENSE, CDS, coding, INTERNAL best transcript NM_001195422	GTPBP3	0,65
ADAM metallopeptidase domain 32	ADAM32	0,65
RNA pseudouridylate synthase domain containing 4	RPUSD4	0,65
ribosomal protein L13; small nucleolar RNA, C/D box 68	RPL13; SNORD68	0,65
transmembrane protein 219	TMEM219	0,65
solute carrier family 25, member 48	SLC25A48	0,65
Memczak2013 ANTISENSE, coding, INTERNAL, intronic best transcript NM_006322	TUBGCP3	0,65
oxoeicosanoid (OXE) receptor 1	OXER1	0,65
methionyl-tRNA synthetase; microRNA 6758	MARS; MIR6758	0,65
zinc finger protein 839	ZNF839	0,65

hydroxysteroid (17-beta) dehydrogenase 11	HSD17B11	0,65
calcium channel, voltage-dependent, beta 4 subunit	CACNB4	0,65
fibronectin leucine rich transmembrane protein 3	FLRT3	0,65
TNNI3 interacting kinase	TNNI3K	0,65
transcription factor Dp-1	TFDP1	0,65
enoyl-CoA hydratase domain containing 2	ECHDC2	0,65
GID complex subunit 4 homolog	GID4	0,65
3-hydroxyanthranilate 3,4-dioxygenase	HAAO	0,65
dual specificity phosphatase 19	DUSP19	0,65
ZHX1-C8orf76 readthrough	ZHX1-C8orf76	0,65
diacylglycerol kinase, delta 130kDa	DGKD	0,65
family with sequence similarity 155, member A	FAM155A	0,65
CCR4-NOT transcription complex subunit 1; small nucleolar RNA, H/ACA box 50A	CNOT1; SNORA50A	0,65
GC-rich sequence DNA-binding factor 2	GCFC2	0,65
beta-2-microglobulin	B2M	0,65
TMEM110-MUSTN1 readthrough; transmembrane protein 110	TMEM110-MUSTN1; TMEM110	0,65
scaffold attachment factor B2	SAFB2	0,65
transmembrane protein 177	TMEM177	0,65
ubiquitously transcribed tetratricopeptide repeat containing, Y-linked	UTY	0,65
DEAD (Asp-Glu-Ala-Asp) box polypeptide 46	DDX46	0,65
solute carrier family 27 (fatty acid transporter), member 3	SLC27A3	0,65
kinesin family member 16B	KIF16B	0,65
scribbled planar cell polarity protein; microRNA 937	SCRIB; MIR937	0,65
solute carrier family 22, member 10	SLC22A10	0,65
hydroxymethylbilane synthase	HMBS	0,65
minichromosome maintenance complex component 4	MCM4	0,65
diffuse panbronchiolitis critical region 1	DPCR1	0,65
WAPL cohesin release factor	WAPL	0,65
Transcript Identified by AceView, Entrez Gene ID(s) 7037	TFRC	0,65
UDP-glucose glycoprotein glucosyltransferase 2	UGGT2	0,65
zinc finger and BTB domain containing 14	ZBTB14	0,65
lysine (K)-specific demethylase 4C	KDM4C	0,65
pyruvate dehydrogenase phosphatase regulatory subunit	PDPR	0,65
hemochromatosis	HFE	0,65
V-set and immunoglobulin domain containing 10	VSIG10	0,65
Transcript Identified by AceView, Entrez Gene ID(s) 3839	KPNA3	0,65
protocadherin beta 14	PCDHB14	0,65

ribosomal protein S6 kinase, 90kDa, polypeptide 2	RPS6KA2	0,65
mitogen-activated protein kinase kinase 2	MAP2K2	0,65
zinc finger protein 232	ZNF232	0,65
zinc finger protein 780B	ZNF780B	0,65
zinc finger protein 780B	ZNF780B	0,65
small integral membrane protein 19	SMIM19	0,65
transmembrane protein 128	TMEM128	0,65
ASH1L antisense RNA 1	ASH1L-AS1	0,65
sulfotransferase family 1B member 1	SULT1B1	0,65
FK506 binding protein 3	FKBP3	0,65
zinc finger protein 852	ZNF852	0,65
PTPN13-like, Y-linked; PTPN13-like, Y-linked 2	PRY; PRY2	0,65
small ubiquitin-like modifier 4	SUMO4	0,65
biogenesis of lysosomal organelles complex-1, subunit 4, cappuccino	BLOC1S4	0,65
splicing factor 1	SF1	0,65
replication protein A3	RPA3	0,65
Jeck2013 ALT_ACCEPTOR, ALT_DONOR, coding, INTERNAL, intronic best transcript NM_012474	UCK2	0,65
LDL receptor related protein 6	LRP6	0,65
serine/arginine-rich splicing factor 2; microRNA 636	SRSF2; MIR636	0,65
tripartite motif containing 41	TRIM41	0,65
coagulation factor XIII, B polypeptide	F13B	0,65
STEAP family member 3, metalloreductase	STEAP3	0,65
transmembrane protein 41A	TMEM41A	0,65
golgi glycoprotein 1	GLG1	0,65
Transcript Identified by AceView, Entrez Gene ID(s) 93663; 253582; Jeck2013 ALT_ACCEPTOR, ALT_DONOR, downstream_end, ncRNA, OVEXON, upstream_start best transcript TCONS_l2_00025470; Jeck2013 ALT_ACCEPTOR, ncRNA, OVEXON, upstream_start best transcript TCONS_l2_00024911; Salzman2013 ANTISENSE, CDS, coding, downstream_end, intronic, OVCODE, OVEXON, UTR3 best transcript NM_000426; Salzman2013 ANNOTATED, INTERNAL, ncRNA, OVEXON best transcript TCONS_l2_00025470; putative novel transcript; novel transcript	C6orf191andARHGAP18; RP1-69D17.4; RP1-69D17.3; TCONS_l2_00025470; TCONS_l2_00024911; LAMA2	0,65
forkhead box B1	FOXB1	0,65
chromatin licensing and DNA replication factor 1	CDT1	0,65
nudE neurodevelopment protein 1-like 1	NDEL1	0,65
all-trans retinoic acid-induced differentiation factor	ATRAID	0,65
citrate lyase beta like	CLYBL	0,65
zinc finger protein 33B	ZNF33B	0,65
methylcrotonoyl-CoA carboxylase 2	MCCC2	0,65

solute carrier family 35 (UDP-xylose/UDP-N-acetylglucosamine transporter), member B4	SLC35B4	0,65
phosphatidylinositol glycan anchor biosynthesis class Y; PIGY upstream reading frame	PIGY; PYURF	0,65
PYM homolog 1, exon junction complex associated factor	PYM1	0,65
dual specificity tyrosine-(Y)-phosphorylation regulated kinase 4	DYRK4	0,65
SERPINE1 mRNA binding protein 1	SERBP1	0,65
tumor protein p53 inducible nuclear protein 1	TP53INP1	0,65
elastin microfibril interfacer 2	EMILIN2	0,65
transmembrane protein 169	TMEM169	0,65
neuron navigator 2	NAV2	0,65
post-GPI attachment to proteins 3	PGAP3	0,65
Transcript Identified by AceView, Entrez Gene ID(s) 375248	ANKRD36	0,65
trans-golgi network vesicle protein 23 homolog B (S. cerevisiae)	TVP23B	0,65
nudix hydrolase 18	NUDT18	0,65
Yip1 domain family member 5	YIPF5	0,65
HLA complex P5 (non-protein coding)	HCP5	0,65
sestrin 3	SESN3	0,65
prenyl (decaprenyl) diphosphate synthase, subunit 2	PDSS2	0,65
ribosomal protein L39; small nucleolar RNA, H/ACA box 69	RPL39; SNORA69	0,65
pericentriolar material 1	PCM1	0,65
UDP-GlcNAc:betaGal beta-1,3-N-acetylglucosaminyltransferase 2	B3GNT2	0,65
hemoglobin, zeta	HBZ	0,65
poly(A) binding protein, cytoplasmic 1; microRNA 7705	PABPC1; MIR7705	0,65
TBC1 domain family, member 9 (with GRAM domain)	TBC1D9	0,65
dual specificity phosphatase 16	DUSP16	0,65
NLR family member X1	NLRX1	0,65
G protein-coupled receptor 18	GPR18	0,65
glutathione S-transferase alpha 5	GSTA5	0,65
echinoderm microtubule associated protein like 6	EML6	0,65
SERTA domain containing 3	SERTAD3	0,64
DnaJ (Hsp40) homolog, subfamily B, member 11	DNAJB11	0,64
GTP cyclohydrolase 1	GCH1	0,64
mitogen-activated protein kinase 3	MAPK3	0,64
RCC1 domain containing 1	RCCD1	0,64
acyl-CoA binding domain containing 7	ACBD7	0,64
interleukin 13 receptor, alpha 1	IL13RA1	0,64
ring finger protein 208	RNF208	0,64

Zhang2013 ALT_ACCEPTOR, ALT_DONOR, coding, INTERNAL, intronic best transcript NM_030653	DDX11	0,64
midasin AAA ATPase 1	MDN1	0,64
S100 calcium binding protein A1	S100A1	0,64
RAS-like, family 11, member A	RASL11A	0,64
zinc finger protein 605	ZNF605	0,64
PRAME family member 15; PRAME family member 9	PRAMEF15; PRAMEF9	0,64
tribbles pseudokinase 2	TRIB2	0,64
ribosomal protein L18a	RPL18A	0,64
Memczak2013 ANTISENSE, coding, INTERNAL, intronic best transcript NM_001202514	MXD1	0,64
proteasome 26S subunit, ATPase 3	PSMC3	0,64
SLCO4A1 antisense RNA 1	SLCO4A1-AS1	0,64
Zhang2013 ALT_ACCEPTOR, ALT_DONOR, coding, INTERNAL, intronic best transcript NM_002473	MYH9	0,64
tet methylcytosine dioxygenase 3	TET3	0,64
SWI/SNF-related, matrix-associated actin-dependent regulator of chromatin, subfamily a, containing DEAD/H box 1	SMARCAD1	0,64
cysteine-rich protein 3	CRIP3	0,64
PRC1 antisense RNA 1	PRC1-AS1	0,64
serpin peptidase inhibitor, clade B (ovalbumin), member 9	SERPINB9	0,64
protein tyrosine phosphatase, receptor type, G	PTPRG	0,64
zinc finger protein 254	ZNF254	0,64
protein regulator of cytokinesis 1	PRC1	0,64
Transcript Identified by AceView, Entrez Gene ID(s) 9665	KIAA0430	0,64
integrin alpha 10	ITGA10	0,64
proline-rich protein BstNI subfamily 2	PRB2	0,64
siah E3 ubiquitin protein ligase 1	SIAH1	0,64
Transcript Identified by AceView, Entrez Gene ID(s) 11157	LSM6	0,64
mannosidase, endo-alpha-like	MANEAL	0,64
importin 7; small nucleolar RNA, H/ACA box 23	IPO7; SNORA23	0,64
BLOC-1 related complex subunit 6	BORCS6	0,64
ATP citrate lyase	ACLY	0,64
Abelson helper integration site 1	AHI1	0,64
H3 histone, family 3A; H3 histone, family 3A, pseudogene 4	H3F3A; H3F3AP4	0,64
transducin (beta)-like 2	TBL2	0,64
leucine rich repeat containing 37B	LRRC37B	0,64
Transcript Identified by AceView, Entrez Gene ID(s) 3632	INPP5A	0,64

BCL6 corepressor-like 1	BCORL1	0,64
phosphodiesterase 3B, cGMP-inhibited	PDE3B	0,64
zinc finger, CCHC domain containing 5	ZCCHC5	0,64
zinc finger and SCAN domain containing 25	ZSCAN25	0,64
serum/glucocorticoid regulated kinase family, member 3	SGK3	0,64
Memczak2013 ALT_ACCEPTOR, ALT_DONOR, coding, INTERNAL, intronic best transcript NM_006537	USP3	0,64
solute carrier family 35, member E1	SLC35E1	0,64
PHD finger protein 3	PHF3	0,64
xeroderma pigmentosum, complementation group A	XPA	0,64
SH3 domain binding glutamate-rich protein like 2	SH3BGRL2	0,64
kelch-like family member 22	KLHL22	0,64
spectrin repeat containing, nuclear envelope family member 3	SYNE3	0,64
transmembrane protein 62	TMEM62	0,64
protein-L-isoaspartate (D-aspartate) O-methyltransferase domain containing 2	PCMTD2	0,64
ankyrin repeat domain 36C	ANKRD36C	0,64
TP53 regulated inhibitor of apoptosis 1	TRIAP1	0,64
RAD1 checkpoint DNA exonuclease	RAD1	0,64
estrogen receptor binding site associated, antigen, 9	EBAG9	0,64
suppression of tumorigenicity 14 (colon carcinoma)	ST14	0,64
peroxisomal biogenesis factor 11 beta	PEX11B	0,64
Salzman2013 ANTISENSE, coding, INTERNAL, intronic, OVERLAPTX best transcript NM_152516; novel transcript	AC018462.2; COMMD1	0,64
F-box and leucine-rich repeat protein 17	FBXL17	0,64
cytochrome P450, family 27, subfamily A, polypeptide 1	CYP27A1	0,64
Meis homeobox 2	MEIS2	0,64
hypoxia up-regulated 1	HYOU1	0,64
polybromo 1	PBRM1	0,64
TM4SF19 antisense RNA 1	TM4SF19-AS1	0,64
C1QTNF3-AMACR readthrough (NMD candidate)	C1QTNF3-AMACR	0,64
3-oxoacyl-ACP synthase, mitochondrial	OXSM	0,64
GTPase activating protein (SH3 domain) binding protein 1	G3BP1	0,64
Transcript Identified by AceView, Entrez Gene ID(s) 221937	FOXK1	0,64
superkiller viralicidic activity 2-like 2 (S. cerevisiae)	SKIV2L2	0,64
golgi phosphoprotein 3 (coat-protein)	GOLPH3	0,64
ribosomal protein S21	RPS21	0,64
polypeptide N-acetylgalactosaminyltransferase 16	GALNT16	0,64
dermatan sulfate epimerase	DSE	0,64
nucleolar protein 11	NOL11	0,64

phospholipase D1, phosphatidylcholine-specific	PLD1	0,64
nuclear receptor interacting protein 1	NRIP1	0,64
fibrillarin	FBL	0,64
tyrosylprotein sulfotransferase 1	TPST1	0,64
serine/threonine kinase 11	STK11	0,64
zinc finger protein 366	ZNF366	0,64
PTPN13-like, Y-linked pseudogene 3	PRYP3	0,64
negative elongation factor complex member B	NELFB	0,64
centrin 2	CETN2	0,64
frizzled-related protein	FRZB	0,64
F-box protein 31	FBXO31	0,64
zinc finger protein 599	ZNF599	0,64
N-acetylglucosamine-1-phosphodiester alpha-N-acetylglucosaminidase	NAGPA	0,64
ATPase, class V, type 10D	ATP10D	0,64
cornichon family AMPA receptor auxiliary protein 1	CNIH1	0,64
fibroblast growth factor receptor 2	FGFR2	0,64
chromosome 16 open reading frame 70	C16orf70	0,64
adhesion G protein-coupled receptor F3	ADGRF3	0,64
RAD21 cohesin complex component; microRNA 3610	RAD21; MIR3610	0,64
zinc finger CCCH-type containing 12B	ZC3H12B	0,64
NADH dehydrogenase (ubiquinone) 1 beta subcomplex, 4, 15kDa	NDUFB4	0,64
F-box and leucine-rich repeat protein 4	FBXL4	0,64
synaptogyrin 1	SYNGR1	0,64
FSHD region gene 1 family member B, pseudogene	FRG1BP	0,64
RAB26, member RAS oncogene family	RAB26	0,64
family with sequence similarity 219, member B	FAM219B	0,64
protein O-fucosyltransferase 2	POFUT2	0,64
interferon, omega 1	IFNW1	0,64
desumoylating isopeptidase 2	DESI2	0,64
histone cluster 2, H2aa3; histone cluster 2, H2aa4	HIST2H2AA3; HIST2H2AA4	0,64
patched domain containing 3	PTCHD3	0,64
apolipoprotein B mRNA editing enzyme, catalytic polypeptide-like 3G	APOBEC3G	0,64
zinc and ring finger 2, E3 ubiquitin protein ligase	ZNRF2	0,64
mitochondrial ribosomal protein S31	MRPS31	0,64
endoplasmic reticulum aminopeptidase 2	ERAP2	0,64
interleukin enhancer binding factor 3	ILF3	0,64
nuclear factor, erythroid 2-like 2	NFE2L2	0,64
MAF transcriptional regulator RNA	MAFTRR	0,64
dual specificity phosphatase 16	DUSP16	0,64

Jeck2013 ALT_ACCEPTOR, ALT_DONOR, coding, INTERNAL, intronic best transcript NM_001037131; Transcript Identified by AceView	AGAP1; noygloy; torkeybo	0,64
trafficking protein particle complex 6A	TRAPPC6A	0,64
catenin (cadherin-associated protein), delta 1	CTNND1	0,64
ELM2 and Myb/SANT-like domain containing 1	ELMSAN1	0,63
pre-mRNA processing factor 8	PRPF8	0,63
COP9 signalosome subunit 5	COPS5	0,63
TBC1 domain family, member 5	TBC1D5	0,63
Eukaryotic translation initiation factor 3 subunit F [Source:UniProtKB/Swiss-Prot;Acc:O00303]	EIF3F	0,63
angel homolog 2 (Drosophila)	ANGEL2	0,63
family with sequence similarity 96, member A	FAM96A	0,63
H2A histone family, member V	H2AFV	0,63
lysine (K)-specific demethylase 1A; microRNA 3115	KDM1A; MIR3115	0,63
pleckstrin homology domain containing, family J member 1; microRNA 6789	PLEKHJ1; MIR6789	0,63
contactin associated protein 1	CNTNAP1	0,63
ribosomal protein L7a; small nucleolar RNA, C/D box 36C; small nucleolar RNA, C/D box 36B; small nucleolar RNA, C/D box 24; small nucleolar RNA, C/D box 36A	RPL7A; SNORD36C; SNORD36B; SNORD24; SNORD36A	0,63
chromosome 21 open reading frame 2	C21orf2	0,63
chromosome 1 open reading frame 210	C1orf210	0,63
collagen, type IV, alpha 6	COL4A6	0,63
translocase of inner mitochondrial membrane 10 homolog B (yeast)	TIMM10B	0,63
NDC80 kinetochore complex component	NDC80	0,63
collagen, type III, alpha 1; microRNA 3606	COL3A1; MIR3606	0,63
annexin A6	ANXA6	0,63
mediator complex subunit 29	MED29	0,63
phospholysine phosphohistidine inorganic pyrophosphate phosphatase	LHPP	0,63
F-box protein 22	FBXO22	0,63
ubiquinol-cytochrome c reductase, Rieske iron-sulfur polypeptide 1	UQCRFS1	0,63
excision repair cross-complementation group 6-like 2	ERCC6L2	0,63
K(lysine) acetyltransferase 6B	KAT6B	0,63
3-hydroxybutyrate dehydrogenase, type 2	BDH2	0,63
palmitoyl-protein thioesterase 2; EGF-like-domain, multiple 8; PPT2-EGFL8 readthrough (NMD candidate)	PPT2; EGFL8; PPT2-EGFL8	0,63
mitotic spindle organizing protein 2B	MZT2B	0,63
calsyntenin 1	CLSTN1	0,63

THAP domain containing 4	THAP4	0,63
Z-DNA binding protein 1	ZBP1	0,63
RNA pseudouridylate synthase domain containing 1	RPUSD1	0,63
KAT8 regulatory NSL complex subunit 2; small nucleolar RNA, H/ACA box 2B	KANSL2; SNORA2B	0,63
PHD finger protein 13	PHF13	0,63
U2 snRNP-associated SURP domain containing	U2SURP	0,63
zinc finger protein 174	ZNF174	0,63
kringle containing transmembrane protein 1	KREMEN1	0,63
coiled-coil domain containing 170	CCDC170	0,63
ATP binding cassette subfamily C member 12	ABCC12	0,63
SUMO1/sentrin specific peptidase 6	SENP6	0,63
phosphoribosyl pyrophosphate synthetase-associated protein 2	PRPSAP2	0,63
intercellular adhesion molecule 1	ICAM1	0,63
nuclear cap binding protein subunit 1	NCBP1	0,63
bromodomain and WD repeat domain containing 3	BRWD3	0,63
alpha-methylacyl-CoA racemase	AMACR	0,63
leucine rich repeat transmembrane neuronal 4	LRRTM4	0,63
TM2 domain containing 3	TM2D3	0,63
LSM14A mRNA processing body assembly factor	LSM14A	0,63
post-GPI attachment to proteins 1	PGAP1	0,63
dimethylarginine dimethylaminohydrolase 1	DDAH1	0,63
endoplasmic reticulum metallopeptidase 1	ERMP1	0,63
sorting nexin 7	SNX7	0,63
zinc finger protein 624	ZNF624	0,63
dihydrolipoamide dehydrogenase	DLD	0,63
WD repeat containing planar cell polarity effector	WDPCP	0,63
lactotransferrin	LTF	0,63
Memczak2013 ALT_ACCEPTOR, ALT_DONOR, coding, INTERNAL, intronic best transcript NM_001174072	SERINC5	0,63
ARHGAP19-SLIT1 readthrough (NMD candidate)	ARHGAP19-SLIT1	0,63
synaptonemal complex protein 3	SYCP3	0,63
MON2 homolog, regulator of endosome-to-Golgi trafficking	MON2	0,63
hydroxymethylbilane synthase	HMBS	0,63
lysosomal-associated membrane protein 2	LAMP2	0,63
chromosome 8 open reading frame 82; leucine rich repeat containing 24	C8orf82; LRRC24	0,63
TAF6-like RNA polymerase II, p300/CBP-associated factor (PCAF)-associated factor, 65kDa	TAF6L	0,63
Zhang2013 ALT_ACCEPTOR, ALT_DONOR, coding, INTERNAL, intronic, OVERLAPTX best transcript NM_002140	HNRNPK	0,63
junctional adhesion molecule 3	JAM3	0,63

family with sequence similarity 151, member A	FAM151A	0,63
histone cluster 1, H3a	HIST1H3A	0,63
mitochondrial ribosomal protein L34	MRPL34	0,63
exportin 7	XPO7	0,63
Transcript Identified by AceView, Entrez Gene ID(s) 26009	ZZZ3	0,63
forkhead box A1	FOXA1	0,63
talin 2	TLN2	0,63
adenosylhomocysteinase-like 2	AHCYL2	0,63
CTD small phosphatase like 2	CTDSPL2	0,63
SUZ12 polycomb repressive complex 2 subunit	SUZ12	0,63
MYC binding protein 2, E3 ubiquitin protein ligase	MYCBP2	0,63
coiled-coil domain containing 68	CCDC68	0,63
tumor necrosis factor (ligand) superfamily, member 14	TNFSF14	0,63
LSM family member 14B	LSM14B	0,63
olfactomedin like 1	OLFML1	0,63
BCL2/adenovirus E1B 19kD interacting protein like	BNIPL	0,63
excision repair cross-complementation group 2	ERCC2	0,63
DIRAS family, GTP-binding RAS-like 1	DIRAS1	0,63
protein serine kinase H1	PSKH1	0,63
serine/threonine protein kinase 26	STK26	0,63
mitochondrial ribosomal protein S34	MRPS34	0,63
DOT1-like histone H3K79 methyltransferase	DOT1L	0,63
M-phase specific PLK1 interacting protein	MPLKIP	0,63
mitochondrial ribosomal protein L12	MRPL12	0,63
solute carrier family 35 (UDP-N-acetylglucosamine (UDP-GlcNAc) transporter), member A3	SLC35A3	0,63
ADP-dependent glucokinase	ADPGK	0,63
frataxin	FXN	0,63
oligosaccharyltransferase complex subunit pseudogene 1	OSTCP1	0,63
cytochrome P450, family 21, subfamily A, polypeptide 2	CYP21A2	0,63
fibrillarin	FBL	0,63
GRB2-associated binding protein 1	GAB1	0,63
small EDRK-rich factor 2	SERF2	0,63
C1GALT1 specific chaperone 1	C1GALT1C1	0,63
arsenite methyltransferase	AS3MT	0,63
chromosome 19 open reading frame 12	C19orf12	0,63
transmembrane anterior posterior transformation 1	TAPT1	0,63
zinc finger protein 572	ZNF572	0,63
teratocarcinoma-derived growth factor 1	TDGF1	0,63
biorientation of chromosomes in cell division 1	BOD1	0,63
Ewing tumor-associated antigen 1	ETAA1	0,63
enoyl-CoA delta isomerase 1	ECI1	0,62
ring-box 1, E3 ubiquitin protein ligase	RBX1	0,62

hypoxia up-regulated 1	HYOU1	0,62
follistatin-like 4	FSTL4	0,62
vacuolar protein sorting 37 homolog D (S. cerevisiae)	VPS37D	0,62
LPS-responsive vesicle trafficking, beach and anchor containing	LRBA	0,62
transmembrane protein 94; microRNA 6785	TMEM94; MIR6785	0,62
AT rich interactive domain 3A (BRIGHT-like)	ARID3A	0,62
mitogen-activated protein kinase kinase 7	MAP2K7	0,62
D-aspartate oxidase	DDO	0,62
meiosis-specific nuclear structural 1	MNS1	0,62
ADP-ribosylation factor like GTPase 17B	ARL17B	0,62
ER degradation enhancer, mannosidase alpha-like 3	EDEM3	0,62
KIAA1586	KIAA1586	0,62
ankyrin repeat and SOCS box containing 3	ASB3	0,62
coiled-coil domain containing 69	CCDC69	0,62
interleukin 19	IL19	0,62
mitochondrial ribosomal protein L57	MRPL57	0,62
tyrosine kinase 2	TYK2	0,62
phosphatidylinositol-4-phosphate 3-kinase, catalytic subunit type 2 beta	PIK3C2B	0,62
secretory carrier membrane protein 5	SCAMP5	0,62
frizzled class receptor 9	FZD9	0,62
Rho GTPase activating protein 44	ARHGAP44	0,62
Transcript Identified by AceView, Entrez Gene ID(s) 84267	C9orf64	0,62
inositol-tetrakisphosphate 1-kinase	ITPK1	0,62
TGF-beta activated kinase 1/MAP3K7 binding protein 1	TAB1	0,62
long intergenic non-protein coding RNA 1578	LINC01578	0,62
C2 calcium-dependent domain containing 5	C2CD5	0,62
Transcript Identified by AceView, Entrez Gene ID(s) 51008	ASCC1	0,62
MPN domain containing	MPND	0,62
lysosomal-associated membrane protein family, member 5	LAMP5	0,62
Homo sapiens H3 histone, family 3A, pseudogene 4 (H3F3AP4), non-coding RNA.; Homo sapiens H3 histone, family 3A, mRNA (cDNA clone MGC:47761 IMAGE:6014954), complete cds.; Homo sapiens H3 histone, family 3B (H3.3B), mRNA (cDNA clone MGC:87782 IMAGE:3884607), complete cds.; Homo sapiens H3 histone, family 3A, mRNA (cDNA clone MGC:87783 IMAGE:5162817), complete cds.	H3F3AP4; H3F3A; H3F3B	0,62
enkurin domain containing 1	ENKD1	0,62
SAYSVFN motif domain containing 1	SAYSD1	0,62

bromodomain containing 1	BRD1	0,62
HEAT repeat containing 3	HEATR3	0,62
ribosomal protein L7a	RPL7A	0,62
NIMA-related kinase 9	NEK9	0,62
myosin light chain 6B	MYL6B	0,62
ATP synthase, H+ transporting, mitochondrial Fo complex subunit s (factor B)	ATP5S	0,62
MDM2 binding protein	MTBP	0,62
family with sequence similarity 210, member B	FAM210B	0,62
ribosomal protein S16	RPS16	0,62
zinc finger protein 131	ZNF131	0,62
zinc finger protein 546	ZNF546	0,62
plakophilin 2	PKP2	0,62
collagen, type VIII, alpha 1	COL8A1	0,62
NADPH oxidase 3	NOX3	0,62
tetratricopeptide repeat domain 8	TTC8	0,62
SET binding protein 1	SETBP1	0,62
spectrin repeat containing, nuclear envelope family member 4	SYNE4	0,62
isoamyl acetate-hydrolyzing esterase 1 homolog	IAH1	0,62
ribosomal protein L31	RPL31	0,62
transmembrane protein, adipocyte asscociated 1	TPRA1	0,62
solute carrier family 26 (anion exchanger), member 5	SLC26A5	0,62
spindlin family, member 3	SPIN3	0,62
B-cell scaffold protein with ankyrin repeats 1	BANK1	0,62
PIH1 domain containing 1	PIH1D1	0,62
family with sequence similarity 195, member A	FAM195A	0,62
hes family bHLH transcription factor 6	HES6	0,62
inositol polyphosphate-1-phosphatase	INPP1	0,62
ankyrin repeat domain 10	ANKRD10	0,62
KIAA1958	KIAA1958	0,62
protein arginine methyltransferase 6	PRMT6	0,62
activin A receptor type IIA	ACVR2A	0,62
single-pass membrane protein with aspartate-rich tail 1	SMDT1	0,62
FXYD domain containing ion transport regulator 3; microRNA 6887	FXYD3; MIR6887	0,62
armadillo repeat containing, X-linked 3	ARMCX3	0,62
nitric oxide associated 1	NOA1	0,62
taste receptor, type 2, member 10	TAS2R10	0,62
PMS1 homolog 1, mismatch repair system component	PMS1	0,62
cadherin-related family member 3	CDHR3	0,62
ankyrin repeat and sterile alpha motif domain containing 1B	ANKS1B	0,62
zinc finger protein 700	ZNF700	0,62

leucine-rich repeats and immunoglobulin-like domains 2	LRIG2	0,62
ribonuclease H2, subunit A	RNASEH2A	0,62
uncharacterized LOC100130691; Transcript Identified by AceView, Entrez Gene ID(s) 100130691; 4780, RefSeq ID(s) NR_026966; novel transcript	LOC100130691; AC074286.1; NFE2L2	0,62
forkhead box A2	FOXA2	0,62
Fanconi anemia complementation group M	FANCM	0,62
period circadian clock 2	PER2	0,62
erythrocyte membrane protein band 4.1 like 4B	EPB41L4B	0,62
centrosomal protein 63kDa	CEP63	0,62
SAC1 suppressor of actin mutations 1-like (yeast)	SACM1L	0,62
sarcoglycan zeta	SGCZ	0,62
transducin-like enhancer of split 4	TLE4	0,62
proline rich 11	PRR11	0,62
eukaryotic translation initiation factor 1A, X-linked	EIF1AX	0,62
exosome component 8	EXOSC8	0,62
defensin, beta 133	DEFB133	0,62
phosphoglycerate mutase family member 4	PGAM4	0,62
ALG11, alpha-1,2-mannosyltransferase; UTP14, U3 small nucleolar ribonucleoprotein, homolog C (yeast)	ALG11; UTP14C	0,62
transmembrane and coiled-coil domains 4	TMCO4	0,62
amyloid beta (A4) precursor protein	APP	0,62
nudix hydrolase 21	NUDT21	0,62
X-ray repair complementing defective repair in Chinese hamster cells 1	XRCC1	0,62
calpain 12	CAPN12	0,62
magnesium transporter 1	MAGT1	0,62
ARP8 actin-related protein 8 homolog (yeast)	ACTR8	0,62
secreted frizzled-related protein 4	SFRP4	0,62
A kinase (PRKA) anchor protein 5	AKAP5	0,62
	LRIG1	0,62
argonaute RISC catalytic component 3	ago-03	0,62
BCDIN3 domain containing	BCDIN3D	0,62
olfactory receptor, family 6, subfamily P, member 1	OR6P1	0,62
N-deacetylase/N-sulfotransferase (heparan glucosaminyl) 1	NDST1	0,62
zinc finger protein 547; trafficking protein particle complex 2B; trafficking protein particle complex 2	ZNF547; TRAPPC2B; TRAPPC2	0,62
IKBKB interacting protein	IKBIP	0,62
zw10 kinetochore protein	ZW10	0,61
coiled-coil domain containing 180	CCDC180	0,61
proline-rich nuclear receptor coactivator 2	PNRC2	0,61
propionyl-CoA carboxylase beta subunit	PCCB	0,61

ribosomal protein S25	RPS25	0,61
kinectin 1 (kinesin receptor)	KTN1	0,61
polymerase (RNA) III (DNA directed) polypeptide E (80kD)	POLR3E	0,61
ZFP41 zinc finger protein	ZFP41	0,61
mediator complex subunit 22	MED22	0,61
leucine-rich repeats and IQ motif containing 3	LRRIQ3	0,61
RNA binding motif protein 4B	RBM4B	0,61
peroxiredoxin 2	PRDX2	0,61
carnitine palmitoyltransferase 1B (muscle)	CPT1B	0,61
euchromatic histone-lysine N-methyltransferase 2	EHMT2	0,61
adenosine deaminase, RNA-specific, B1	ADARB1	0,61
acyl-CoA dehydrogenase, C-4 to C-12 straight chain	ACADM	0,61
PAP associated domain containing 7	PAPD7	0,61
interferon, alpha-inducible protein 6	IFI6	0,61
Eukaryotic translation initiation factor 3 subunit F [Source:UniProtKB/Swiss-Prot;Acc:O00303]	EIF3F	0,61
DEAD (Asp-Glu-Ala-Asp) box helicase 3, X-linked	DDX3X	0,61
ATPase family, AAA domain containing 3B	ATAD3B	0,61
solute carrier family 6 (neurotransmitter transporter), member 4	SLC6A4	0,61
electron-transfer-flavoprotein, alpha polypeptide	ETFA	0,61
ankyrin repeat domain 36	ANKRD36	0,61
homeobox A3	HOXA3	0,61
abhydrolase domain containing 8	ABHD8	0,61
anaphase promoting complex subunit 2	ANAPC2	0,61
phosphatidylinositol glycan anchor biosynthesis class L	PIGL	0,61
mitochondrial elongation factor 1	MIEF1	0,61
ECSIT signalling integrator	ECSIT	0,61
SEC22 homolog B, vesicle trafficking protein (gene/pseudogene)	SEC22B	0,61
solute carrier family 27 (fatty acid transporter), member 2	SLC27A2	0,61
zinc finger protein 770	ZNF770	0,61
sosondowah ankyrin repeat domain family member A	SOWAHA	0,61
zinc finger, HIT-type containing 3	ZNHIT3	0,61
sideroflexin 4	SFXN4	0,61
ceramide kinase	CERK	0,61
Finkel-Biskis-Reilly murine sarcoma virus (FBR-MuSV) ubiquitously expressed	FAU	0,61
TNF receptor-associated protein 1	TRAP1	0,61
family with sequence similarity 229, member B	FAM229B	0,61
Jeck2013 ALT_ACCEPTOR, ALT_DONOR, coding, INTERNAL, intronic best transcript NM_004307	APBB2	0,61

minichromosome maintenance 9 homologous recombination repair factor	MCM9	0,61
mitochondrial ribosomal protein L11	MRPL11	0,61
nudix hydrolase 7	NUDT7	0,61
Rap guanine nucleotide exchange factor 4	RAPGEF4	0,61
F-box protein 38	FBXO38	0,61
zinc finger protein 660	ZNF660	0,61
trans-2,3-enoyl-CoA reductase; microRNA 639	TECR; MIR639	0,61
HIV-1 Tat specific factor 1	HTATSF1	0,61
DnaJ (Hsp40) homolog, subfamily C, member 4	DNAJC4	0,61
HIRA interacting protein 3	HIRIP3	0,61
olfactory receptor, family 2, subfamily J, member 3	OR2J3	0,61
acylphosphatase 2, muscle type	ACYP2	0,61
v-myc avian myelocytomatosis viral oncogene homolog	MYC	0,61
small nuclear RNA activating complex polypeptide 2	SNAPC2	0,61
mitochondrial assembly of ribosomal large subunit 1	MALSU1	0,61
methyltransferase like 17	METTL17	0,61
WNK lysine deficient protein kinase 3	WNK3	0,61
ankyrin repeat and KH domain containing 1; eukaryotic translation initiation factor 4E binding protein 3; ANKHD1-EIF4EBP3 readthrough	ANKHD1; EIF4EBP3; ANKHD1-EIF4EBP3	0,61
COP9 signalosome subunit 4	COPS4	0,61
SAMM50 sorting and assembly machinery component	SAMM50	0,61
SAGA complex associated factor 29	SGF29	0,61
zinc finger protein 852	ZNF852	0,61
LON peptidase N-terminal domain and ring finger 1	LONRF1	0,61
transmembrane protein 27	TMEM27	0,61
mortality factor 4 like 1	MORF4L1	0,61
transformer 2 beta homolog (Drosophila)	TRA2B	0,61
DnaJ (Hsp40) homolog, subfamily C, member 14	DNAJC14	0,61
mastermind-like transcriptional coactivator 2	MAML2	0,61
NADH dehydrogenase (ubiquinone) 1 alpha subcomplex, 11, 14.7kDa	NDUFA11	0,61
golgin A2	GOLGA2	0,61
receptor-interacting serine-threonine kinase 4	RIPK4	0,61
succinate dehydrogenase complex assembly factor 4	SDHAF4	0,61
DBF4 zinc finger	DBF4	0,61
TAP binding protein (tapasin)	TAPBP	0,61
transmembrane protein 38A	TMEM38A	0,61
CHURC1-FNTB readthrough	CHURC1-FNTB	0,61
nuclear prelamin A recognition factor-like	NARFL	0,61
fibrillarin	FBL	0,61
dymeclin	DYM	0,61
hypoxia up-regulated 1	HYOU1	0,61

enoyl-CoA hydratase, short chain, 1, mitochondrial	ECHS1	0,61
protein disulfide isomerase family A member 3	PDIA3	0,61
nudix hydrolase 16 like 1	NUDT16L1	0,61
NIMA-related kinase 10	NEK10	0,61
protein phosphatase 1, regulatory subunit 8	PPP1R8	0,61
nuclear receptor coactivator 5	NCOA5	0,61
GPR75-ASB3 readthrough	GPR75-ASB3	0,61
synaptotagmin VII	SYT7	0,61
histone cluster 1, H2bd	HIST1H2BD	0,61
kallikrein B1	KLKB1	0,61
diacylglycerol kinase, delta 130kDa	DGKD	0,61
Transcript Identified by AceView, Entrez Gene ID(s) 6764	ST5	0,61
FANCD2/FANCI-associated nuclease 1	FAN1	0,60
hect domain and RLD 2 pseudogene 2	HERC2P2	0,60
NCBP2 antisense RNA 2 (head to head)	NCBP2-AS2	0,60
peptidase (mitochondrial processing) beta	PMPCB	0,60
Transcript Identified by AceView, Entrez Gene ID(s) 10915	TCERG1	0,60
endonuclease V	ENDOV	0,60
ADP-ribosylation factor guanine nucleotide-exchange factor 1 (brefeldin A-inhibited)	ARFGEF1	0,60
MIS18 binding protein 1	MIS18BP1	0,60
transforming growth factor beta receptor III like	TGFBR3L	0,60
zinc finger protein 594	ZNF594	0,60
CENPB DNA-binding domain containing 1	CENPBD1	0,60
atlastin GTPase 2	ATL2	0,60
integrator complex subunit 9	INTS9	0,60
scavenger receptor class B, member 1	SCARB1	0,60
solute carrier family 16 (monocarboxylate transporter), member 3; microRNA 6787	SLC16A3; MIR6787	0,60
NADH dehydrogenase (ubiquinone) 1 beta subcomplex, 10, 22kDa	NDUFB10	0,60
zinc finger protein 781	ZNF781	0,60
Transcript Identified by AceView, Entrez Gene ID(s) 26249	KLHL3	0,60
KIAA1257	KIAA1257	0,60
transmembrane protein 91	TMEM91	0,60
golgi reassembly stacking protein 1	GORASP1	0,60
Zhang2013 ALT_ACCEPTOR, ALT_DONOR, coding, INTERNAL, intronic best transcript NM_182706	SCRIB	0,60
interferon gamma receptor 2 (interferon gamma transducer 1)	IFNGR2	0,60
amelogenin, Y-linked	AMELY	0,60
quiescin Q6 sulfhydryl oxidase 2	QSOX2	0,60

cyclin-dependent kinase 2	CDK2	0,60
translational activator of mitochondrially encoded cytochrome c oxidase I	TACO1	0,60
chromatin accessibility complex 1	CHRAC1	0,60
serine hydroxymethyltransferase 2 (mitochondrial)	SHMT2	0,60
zinc finger protein 606	ZNF606	0,60
Rap guanine nucleotide exchange factor 5	RAPGEF5	0,60
myosin IG	MYO1G	0,60
vacuolar protein sorting 13 homolog A (S. cerevisiae)	VPS13A	0,60
mesoderm development candidate 2	MESDC2	0,60
high mobility group box 3	HMGB3	0,60
zinc finger, CCHC domain containing 13	ZCCHC13	0,60
HECT domain containing E3 ubiquitin protein ligase 1	HECTD1	0,60
F-box and WD repeat domain containing 5	FBXW5	0,60
ATP binding cassette subfamily B member 6 (Langereis blood group)	ABCB6	0,60
high mobility group 20B	HMG20B	0,60
REX4 homolog, 3'-5' exonuclease [Source:HGNC Symbol;Acc:HGNC:12820]	REXO4	0,60
opioid growth factor receptor	OGFR	0,60
Rho GTPase activating protein 19	ARHGAP19	0,60
tudor domain containing 3	TDRD3	0,60
tetratricopeptide repeat domain 33	TTC33	0,60
transcription elongation factor A (SII)-like 8	TCEAL8	0,60
seven transmembrane helix receptor; Transcript Identified by AceView, Entrez Gene ID(s) 650293, RefSeq ID(s) NM_001040071	LOC650293	0,60
chromosome 22 open reading frame 46	C22orf46	0,60
LYR motif containing 7	LYRM7	0,60
tudor domain containing 7	TDRD7	0,60
bora, aurora kinase A activator	BORA	0,60
NADH dehydrogenase (ubiquinone) 1 beta subcomplex, 8, 19kDa	NDUFB8	0,60
glucosamine-phosphate N-acetyltransferase 1	GNPNAT1	0,60
E74-like factor 3 (ets domain transcription factor, epithelial-specific)	ELF3	0,60
protein O-fucosyltransferase 1; microRNA 1825	POFUT1; MIR1825	0,60
ribosomal protein SA; small nucleolar RNA, H/ACA box 62; small nucleolar RNA, H/ACA box 6	RPSA; SNORA62; SNORA6	0,60
transmembrane protease, serine 11D	TMPRSS11D	0,60
Transcript Identified by AceView, Entrez Gene ID(s) 6904	TBCD	0,60
SEC11 homolog A, signal peptidase complex subunit	SEC11A	0,60

aryl-hydrocarbon receptor repressor; programmed cell death 6	AHRR; PDCD6	0,60
ZXD family zinc finger C	ZXDC	0,60
glutathione peroxidase 4	GPX4	0,60
transducer of ERBB2, 1	TOB1	0,60
purinergic receptor P2Y, G-protein coupled, 8	P2RY8	0,60
Homo sapiens purinergic receptor P2Y, G-protein coupled, 8 (P2RY8), mRNA.; purinergic receptor P2Y, G-protein coupled, 8 [Source:HGNC Symbol;Acc:HGNC:15524]; Homo sapiens purinergic receptor P2Y, G-protein coupled, 8, mRNA (cDNA clone MGC:50878 IMAGE:5787268), complete cds.	P2RY8	0,60
laminin, gamma 1 (formerly LAMB2)	LAMC1	0,60
GINS complex subunit 2 (Psf2 homolog)	GINS2	0,60
Bcl2 modifying factor	BMF	0,60
pyrophosphatase (inorganic) 1	PPA1	0,60
nudix hydrolase 6	NUDT6	0,60
FK506 binding protein 8	FKBP8	0,60
adaptor-related protein complex 4, mu 1 subunit	AP4M1	0,60
zinc finger protein 133	ZNF133	0,60
zinc finger protein 497; alpha-1-B glycoprotein	ZNF497; A1BG	0,60
BIVM-ERCC5 readthrough	BIVM-ERCC5	0,60
olfactory receptor, family 2, subfamily C, member 1	OR2C1	0,60
hepatitis A virus cellular receptor 1	HAVCR1	0,60
Jeck2013 ALT_ACCEPTOR, ALT_DONOR, coding, INTERNAL, intronic best transcript NM_001167740	SMYD3	0,60
REX4 homolog, 3'-5' exonuclease [Source:HGNC Symbol;Acc:HGNC:12820]	REXO4	0,60
BCL2/adenovirus E1B 19kDa interacting protein 3	BNIP3	0,60
advillin	AVIL	0,60
zinc finger protein 763	ZNF763	0,60
aspartic peptidase, retroviral-like 1; PCBP1 antisense RNA 1	ASPRV1; PCBP1-AS1	0,60
Salzman2013 ANTISENSE, CDS, coding, INTERNAL, intronic, OVCODE, OVERLAPTX, OVEXON, UTR5 best transcript NM_005603	ATP8B1	0,60
activin A receptor type IC	ACVR1C	0,60
tubulin tyrosine ligase-like family member 10	TTLL10	0,60
Transcript Identified by AceView, Entrez Gene ID(s) 23126	POGZ	0,60
pleckstrin homology domain containing, family H (with MyTH4 domain) member 1	PLEKHH1	0,60
phosphatidylinositol-5-phosphate 4-kinase, type II, beta	PIP4K2B	0,60
chromosome 15 open reading frame 61	C15orf61	0,60
pleiomorphic adenoma gene-like 2	PLAGL2	0,59

laminin, alpha 5; microRNA 4758	LAMA5; MIR4758	0,59
peroxisomal membrane protein 2	PXMP2	0,59
FAST kinase domains 3	FASTKD3	0,59
neurobeachin	NBEA	0,59
progesterone receptor membrane component 1	PGRMC1	0,59
kinesin family member 4A	KIF4A	0,59
catechol-O-methyltransferase; microRNA 4761	COMT; MIR4761	0,59
YES proto-oncogene 1, Src family tyrosine kinase	YES1	0,59
nudix hydrolase 4	NUDT4	0,59
CXXC finger protein 5	CXXC5	0,59
zinc finger and BTB domain containing 34	ZBTB34	0,59
ArfGAP with SH3 domain, ankyrin repeat and PH domain 3	ASAP3	0,59
nucleoporin 133kDa	NUP133	0,59
transmembrane protein 211	TMEM211	0,59
Transcript Identified by AceView, Entrez Gene ID(s) 4683	NBN	0,59
SKI proto-oncogene	SKI	0,59
STARD7 antisense RNA 1	STARD7-AS1	0,59
MRG/MORF4L binding protein	MRGBP	0,59
BCL2-related ovarian killer	BOK	0,59
ubiquitin specific peptidase 24	USP24	0,59
adrenergic, beta, receptor kinase 2	ADRBK2	0,59
transcription factor B1, mitochondrial	TFB1M	0,59
plakophilin 4	PKP4	0,59
Transcript Identified by AceView, Entrez Gene ID(s) 153129	SLC38A9	0,59
CD7 molecule	CD7	0,59
ring finger protein 175	RNF175	0,59
Memczak2013 ANTISENSE, coding, INTERNAL, intronic best transcript NM_001144382	PLCL2	0,59
succinate-CoA ligase, alpha subunit	SUCLG1	0,59
protein kinase C and casein kinase substrate in neurons 3	PACSIN3	0,59
tectonic family member 3	TCTN3	0,59
zinc finger protein 780A	ZNF780A	0,59
ELL associated factor 2	EAF2	0,59
splicing factor proline/glutamine-rich	SFPQ	0,59
SNF related kinase	SNRK	0,59
Salzman2013 ALT_ACCEPTOR, ALT_DONOR, coding, INTERNAL, intronic best transcript NM_080476	PIGU	0,59
POZ (BTB) and AT hook containing zinc finger 1	PATZ1	0,59
cysteine conjugate-beta lyase, cytoplasmic	CCBL1	0,59
Memczak2013 ANTISENSE, CDS, coding, INTERNAL best transcript NM_012235	SCAP	0,59

component of oligomeric golgi complex 2	COG2	0,59
endoplasmic reticulum protein 29	ERP29	0,59
RAB20, member RAS oncogene family	RAB20	0,59
syntaxin 17	STX17	0,59
fibrillarin	FBL	0,59
fibrillarin	FBL	0,59
tripartite motif containing 13; potassium channel regulator	TRIM13; KCNRG	0,59
nuclear transport factor 2-like export factor 2	NXT2	0,59
leucine rich repeat containing 2	LRRC2	0,59
REX4 homolog, 3'-5' exonuclease [Source:HGNC Symbol;Acc:HGNC:12820]	REXO4	0,59
transcription elongation factor A (SII)-like 1	TCEAL1	0,59
flotillin 1	FLOT1	0,59
Memczak2013 ANTISENSE, CDS, coding, INTERNAL best transcript NM_005207	CRKL	0,59
coatomer protein complex subunit beta 1	COPB1	0,59
phosphatase, orphan 2	PHOSPHO2	0,59
Williams-Beuren syndrome chromosome region 16	WBSCR16	0,59
immunoglobulin superfamily, member 8	IGSF8	0,59
nuclear envelope integral membrane protein 2	NEMP2	0,59
transmembrane and ubiquitin-like domain containing 1	TMUB1	0,59
zinc finger protein 33A	ZNF33A	0,59
diacylglycerol kinase, delta 130kDa	DGKD	0,59
WD repeat domain 6	WDR6	0,59
dCMP deaminase	DCTD	0,59
nucleotide binding protein-like	NUBPL	0,59
chromosome 1 open reading frame 53	C1orf53	0,59
adiponectin receptor 2	ADIPOR2	0,59
Transcript Identified by AceView, Entrez Gene ID(s) 51339	DACT1	0,59
zyg-11 family member B, cell cycle regulator	ZYG11B	0,59
reactive oxygen species modulator 1	ROMO1	0,59
prolactin regulatory element binding	PREB	0,59
single-pass membrane protein with coiled-coil domains 4	SMCO4	0,59
u3 small nucleolar ribonucleoprotein protein MPP10-like	LOC643802	0,59
piezo-type mechanosensitive ion channel component 2	PIEZO2	0,59
multiple EGF-like-domains 8	MEGF8	0,59
PHD finger protein 14	PHF14	0,59
histone deacetylase 8	HDAC8	0,59
gamma-glutamylcyclotransferase	GGCT	0,59
Jeck2013 ALT_ACCEPTOR, ALT_DONOR, coding, INTERNAL, intronic best transcript NM_001145418	TTC28	0,59
zinc finger protein 510	ZNF510	0,59

MHC class I polypeptide-related sequence A	MICA	0,59
acyl-CoA synthetase family member 2	ACSF2	0,59
FK506 binding protein 2	FKBP2	0,58
nuclear casein kinase and cyclin-dependent kinase substrate 1	NUCKS1	0,58
notchless homolog 1 (Drosophila)	NLE1	0,58
HEAT repeat containing 5A	HEATR5A	0,58
thymopoietin	TMPO	0,58
Transcript Identified by AceView, Entrez Gene ID(s) 84312	BRMS1L	0,58
ethylmalonyl-CoA decarboxylase 1	ECHDC1	0,58
acetyl-CoA acetyltransferase 1	ACAT1	0,58
family with sequence similarity 63, member A	FAM63A	0,58
Transcript Identified by AceView, Entrez Gene ID(s) 7813	EVI5	0,58
BORCS7-ASMT readthrough (NMD candidate)	BORCS7-ASMT	0,58
syntaxin binding protein 4	STXBP4	0,58
acetyl-CoA carboxylase alpha	ACACA	0,58
armadillo repeat containing 2	ARMC2	0,58
P450 (cytochrome) oxidoreductase; microRNA 4651	POR; MIR4651	0,58
Transcript Identified by AceView, Entrez Gene ID(s) 7752	ZNF200	0,58
solute carrier family 27 (fatty acid transporter), member 5	SLC27A5	0,58
asialoglycoprotein receptor 2	ASGR2	0,58
zinc finger and SCAN domain containing 2	ZSCAN2	0,58
uridine-cytidine kinase 1-like 1; microRNA 1914	UCKL1; MIR1914	0,58
RAB38, member RAS oncogene family	RAB38	0,58
mitochondrial ribosomal protein L1	MRPL1	0,58
TSPY-like 2	TSPYL2	0,58
proline rich membrane anchor 1	PRIMA1	0,58
ribosomal protein S7	RPS7	0,58
DDHD domain containing 2	DDHD2	0,58
centlein, centrosomal protein	CNTLN	0,58
lactation elevated 1	LACE1	0,58
zinc finger protein 684	ZNF684	0,58
transmembrane protein 150C	TMEM150C	0,58
choline/ethanolamine phosphotransferase 1	CEPT1	0,58
selenocysteine lyase	SCLY	0,58
N-methylpurine DNA glycosylase	MPG	0,58
olfactory receptor, family 4, subfamily F, member 29; olfactory receptor, family 4, subfamily F, member 3; olfactory receptor, family 4, subfamily F, member 16	OR4F29; OR4F3; OR4F16	0,58
olfactory receptor, family 4, subfamily F, member 16; olfactory receptor, family 4, subfamily F, member 29	OR4F16; OR4F29	0,58

suppressor of cytokine signaling 1	SOCS1	0,58
glioblastoma amplified sequence	GBAS	0,58
Transcript Identified by AceView, Entrez Gene ID(s) 8997	KALRN	0,58
lectin, mannose-binding, 1	LMAN1	0,58
RAD54-like (S. cerevisiae)	RAD54L	0,58
Memczak2013 ANTISENSE, coding, INTERNAL, intronic best transcript NM_205548	FAM151B	0,58
2,4-dienoyl-CoA reductase 1, mitochondrial	DECR1	0,58
ring finger protein 126	RNF126	0,58
family with sequence similarity 118, member A	FAM118A	0,58
glypican 3	GPC3	0,58
SH3 and PX domains 2A	SH3PXD2A	0,58
zinc finger protein 580; zinc finger protein 581	ZNF580; ZNF581	0,58
pyruvate dehydrogenase kinase, isozyme 1	PDK1	0,58
secretion associated, Ras related GTPase 1A	SAR1A	0,58
tetraspanin 8	TSPAN8	0,58
carbamoyl-phosphate synthetase 2, aspartate transcarbamylase, and dihydroorotase	CAD	0,58
transforming, acidic coiled-coil containing protein 3	TACC3	0,58
zinc finger protein 852	ZNF852	0,58
kinesin family member 2C	KIF2C	0,58
SLAIN motif family member 1	SLAIN1	0,58
FXYD domain containing ion transport regulator 2	FXYD2	0,58
lysine (K)-specific methyltransferase 5C	KMT5C	0,58
histone deacetylase 5	HDAC5	0,58
myosin, heavy chain 10, non-muscle	MYH10	0,58
ChaC glutathione-specific gamma-glutamylcyclotransferase 1	CHAC1	0,58
ring finger protein 13	RNF13	0,58
UDP glucuronosyltransferase 2 family, polypeptide B15	UGT2B15	0,58
coiled-coil domain containing 142; mitochondrial ribosomal protein L53	CCDC142; MRPL53	0,58
zinc finger protein 852	ZNF852	0,58
solute carrier family 10, member 7	SLC10A7	0,58
discs, large (Drosophila) homolog-associated protein 5	DLGAP5	0,58
male germ cell-associated kinase	MAK	0,58
ephrin-B2	EFNB2	0,58
transmembrane p24 trafficking protein 10	TMED10	0,58
acyl-CoA synthetase family member 3	ACSF3	0,58
glutathione S-transferase mu 1	GSTM1	0,58
glomulin, FKBP associated protein	GLMN	0,58
mannose receptor, C type 2	MRC2	0,58
transmembrane protein 121	TMEM121	0,58

Transcript Identified by AceView, Entrez Gene ID(s) 51663	ZFR	0,58
REV1, DNA directed polymerase	REV1	0,58
phosphoserine aminotransferase 1	PSAT1	0,58
BEN domain containing 3	BEND3	0,58
zinc finger protein 644	ZNF644	0,58
zinc finger protein 101	ZNF101	0,58
chromosome 3 open reading frame 35	C3orf35	0,58
RAN binding protein 1	RANBP1	0,58
serine/arginine-rich splicing factor 9	SRSF9	0,58
translation machinery associated 16 homolog	TMA16	0,58
mannose phosphate isomerase	MPI	0,58
Transcript Identified by AceView, Entrez Gene ID(s) 158219	TTC39B	0,58
ankyrin repeat domain 26	ANKRD26	0,58
zinc finger protein 780A	ZNF780A	0,58
ribosomal protein, large, P1	RPLP1	0,58
armadillo repeat containing 1	ARMC1	0,58
transcription factor-like 5 (basic helix-loop-helix)	TCFL5	0,58
solute carrier family 2 (facilitated glucose transporter), member 6	SLC2A6	0,58
GTP binding protein 3 (mitochondrial)	GTPBP3	0,58
serine/arginine-rich splicing factor 3	SRSF3	0,58
glutathione S-transferase mu 2 (muscle)	GSTM2	0,58
Bet1 golgi vesicular membrane trafficking protein	BET1	0,58
collagen, type XXVI, alpha 1	COL26A1	0,58
enhancer of rudimentary homolog (Drosophila)	ERH	0,58
mitotic spindle organizing protein 2A; microRNA 4784	MZT2A; MIR4784	0,58
ST3 beta-galactoside alpha-2,3-sialyltransferase 1	ST3GAL1	0,58
endoplasmic reticulum oxidoreductase alpha	ERO1A	0,58
SET domain containing 3	SETD3	0,58
mitochondrial ribosomal protein L19	MRPL19	0,58
protein phosphatase 1, regulatory subunit 3C	PPP1R3C	0,58
ST3 beta-galactoside alpha-2,3-sialyltransferase 5	ST3GAL5	0,58
fibrillarin	FBL	0,58
vacuolar protein sorting 13 homolog C (S. cerevisiae)	VPS13C	0,58
histone cluster 1, H2ah	HIST1H2AH	0,58
succinate dehydrogenase complex subunit A, flavoprotein (Fp)	SDHA	0,58
beta-site APP-cleaving enzyme 1	BACE1	0,58
chromobox homolog 2	CBX2	0,58
potassium channel subfamily M regulatory beta subunit 3	KCNMB3	0,58
chromosome 19 open reading frame 52	C19orf52	0,57
long intergenic non-protein coding RNA 174	LINC00174	0,57

NIMA-related kinase 8	NEK8	0,57
abhydrolase domain containing 17A	ABHD17A	0,57
proteasome subunit beta 8	PSMB8	0,57
G protein-coupled receptor kinase 6	GRK6	0,57
integrator complex subunit 6	INTS6	0,57
mannosidase, alpha, class 2A, member 2	MAN2A2	0,57
glutaredoxin 2	GLRX2	0,57
zinc finger protein 780B	ZNF780B	0,57
TOPBP1-interacting checkpoint and replication regulator	TICRR	0,57
osteosarcoma amplified 9, endoplasmic reticulum lectin	OS9	0,57
ephrin-A4	EFNA4	0,57
Jeck2013 ALT_ACCEPTOR, ALT_DONOR, coding, INTERNAL, intronic best transcript NM_000127; Transcript Identified by AceView	EXT1; hunera	0,57
shroom family member 1	SHROOM1	0,57
ADP-ribosylation factor like GTPase 6 interacting protein 6	ARL6IP6	0,57
ornithine decarboxylase antizyme 2	OAZ2	0,57
kelch repeat and BTB (POZ) domain containing 6	KBTBD6	0,57
chromosome 2 open reading frame 82	C2orf82	0,57
polycystic kidney disease 1 (autosomal dominant); microRNA 6511b-2; microRNA 6511b-1	PKD1; MIR6511B2; MIR6511B1	0,57
HAUS augmin like complex subunit 7; three prime repair exonuclease 2	HAUS7; TREX2	0,57
SDS3 homolog, SIN3A corepressor complex component	SUDS3	0,57
small nuclear ribonucleoprotein, U11/U12 48KDa subunit	SNRNP48	0,57
zinc finger, CCHC domain containing 24	ZCCHC24	0,57
UDP-glucose glycoprotein glucosyltransferase 1	UGGT1	0,57
solute carrier family 29 (equilibrative nucleoside transporter), member 2	SLC29A2	0,57
polymerase (DNA directed), epsilon 2, accessory subunit	POLE2	0,57
transmembrane p24 trafficking protein 2	TMED2	0,57
serpin peptidase inhibitor, clade A (alpha-1 antiproteinase, antitrypsin), member 2 (gene/pseudogene)	SERPINA2	0,57
ribose 5-phosphate isomerase A	RPIA	0,57
RNA binding motif protein, X-linked; small nucleolar RNA, C/D box 61	RBMX; SNORD61	0,57
cereblon	CRBN	0,57
KIAA1147	KIAA1147	0,57
histone deacetylase 3	HDAC3	0,57
family with sequence similarity 186, member B	FAM186B	0,57

solute carrier family 25 (mitochondrial carrier; adenine nucleotide translocator), member 5	SLC25A5	0,57
protein kinase, DNA-activated, catalytic polypeptide	PRKDC	0,57
cleavage and polyadenylation specific factor 1; microRNA 939; microRNA 1234; microRNA 6849	CPSF1; MIR939; MIR1234; MIR6849	0,57
ribosomal protein L7a	RPL7A	0,57
FK506 binding protein 10	FKBP10	0,57
inhibitor of growth family member 5	ING5	0,57
prostaglandin D2 receptor 2	PTGDR2	0,57
spermatogenesis associated 24	SPATA24	0,57
solute carrier family 35 (adenosine 3-phospho 5-phosphosulfate transporter), member B2; microRNA 4647	SLC35B2; MIR4647	0,57
DiGeorge syndrome critical region gene 2; DiGeorge syndrome critical region gene 11 (non-protein coding)	DGCR2; DGCR11	0,57
receptor-like tyrosine kinase	RYK	0,57
chromodomain helicase DNA binding protein 7	CHD7	0,57
insulin like growth factor binding protein 3	IGFBP3	0,57
high mobility group 20A	HMG20A	0,57
potassium channel, voltage gated Shaw related subfamily C, member 1	KCNC1	0,57
potassium channel, voltage gated modifier subfamily G, member 3	KCNG3	0,57
ring finger protein 144A	RNF144A	0,57
ubiquitin-conjugating enzyme E2, J2	UBE2J2	0,57
ubiquitin conjugating enzyme E2T	UBE2T	0,57
regulator of chromosome condensation 2	RCC2	0,57
praja ring finger 2, E3 ubiquitin protein ligase	PJA2	0,57
malate dehydrogenase 1	MDH1	0,57
peroxisomal biogenesis factor 11 alpha	PEX11A	0,57
zinc finger and SCAN domain containing 5A	ZSCAN5A	0,57
Fanconi anemia complementation group A	FANCA	0,57
HNF1 homeobox B	HNF1B	0,57
solute carrier family 35 (UDP-galactose transporter), member A2	SLC35A2	0,57
saccharopine dehydrogenase (putative)	SCCPDH	0,57
small nuclear ribonucleoprotein, U5 40kDa subunit	SNRNP40	0,57
DEAD (Asp-Glu-Ala-Asp) box helicase 3, Y-linked	DDX3Y	0,57
coatomer protein complex subunit beta 2 (beta prime)	COPB2	0,57
histone cluster 2, H3d	HIST2H3D	0,57
ribonuclease H2, subunit C	RNASEH2C	0,57
protein disulfide isomerase family A, member 6	PDIA6	0,57
focadhesin	FOCAD	0,57

glutamic-pyruvate transaminase (alanine aminotransferase)	GPT	0,57
adenylate cyclase 5	ADCY5	0,57
thyroid hormone receptor, alpha	THRA	0,57
mediator complex subunit 28	MED28	0,57
baculoviral IAP repeat containing 5	BIRC5	0,57
zinc finger protein 573	ZNF573	0,57
AT rich interactive domain 2 (ARID, RFX-like)	ARID2	0,57
mediator complex subunit 13-like	MED13L	0,57
family with sequence similarity 168, member A	FAM168A	0,57
centromere protein E	CENPE	0,57
NDUFC2-KCTD14 readthrough; NADH dehydrogenase (ubiquinone) 1, subcomplex unknown, 2, 14.5kDa; potassium channel tetramerization domain containing 14	NDUFC2-KCTD14; NDUFC2; KCTD14	0,57
pyruvate dehydrogenase (lipoamide) alpha 1	PDHA1	0,57
Sep (O-phosphoserine) tRNA:Sec (selenocysteine) tRNA synthase	SEPSECS	0,57
sulfatase modifying factor 1	SUMF1	0,57
pyruvate dehydrogenase kinase, isozyme 2	PDK2	0,57
ribosomal protein S29; ribosomal protein L32 pseudogene 29	RPS29; RPL32P29	0,57
polycystic kidney disease 2 (autosomal dominant)	PKD2	0,57
nuclear receptor coactivator 2	NCOA2	0,57
THAP domain containing 11	THAP11	0,57
ArfGAP with GTPase domain, ankyrin repeat and PH domain 3	AGAP3	0,57
mitogen-activated protein kinase kinase kinase 4	MAP3K4	0,57
tetraspanin 12	TSPAN12	0,56
zinc finger protein 76	ZNF76	0,56
WD repeat domain 81	WDR81	0,56
RAB42, member RAS oncogene family	RAB42	0,56
endonuclease G	ENDOG	0,56
cell division cycle associated 3	CDCA3	0,56
long intergenic non-protein coding RNA 494	LINC00494	0,56
olfactory receptor, family 2, subfamily J, member 2	OR2J2	0,56
glucuronic acid epimerase	GLCE	0,56
Transcript Identified by AceView, Entrez Gene ID(s) 7184	HSP90B1	0,56
sema domain, transmembrane domain (TM), and cytoplasmic domain, (semaphorin) 6C	SEMA6C	0,56
VCP-interacting membrane selenoprotein	VIMP	0,56
Src homology 2 domain containing F	SHF	0,56
zinc finger protein 629	ZNF629	0,56
ubiquitin family domain containing 1	UBFD1	0,56

solute carrier family 26 (anion exchanger), member 2	SLC26A2	0,56
regulatory solute carrier protein, family 1, member 1; DNA-damage inducible 1 homolog 2	RSC1A1; DDI2	0,56
ATPase, H+ transporting, lysosomal accessory protein 1	ATP6AP1	0,56
TIA1 cytotoxic granule-associated RNA binding protein	TIA1	0,56
SNF2 histone linker PHD RING helicase, E3 ubiquitin protein ligase	SHPRH	0,56
geminin, DNA replication inhibitor	GMNN	0,56
H1 histone family, member X	H1FX	0,56
aldehyde dehydrogenase 2 family (mitochondrial)	ALDH2	0,56
lamin B receptor	LBR	0,56
sex hormone-binding globulin	SHBG	0,56
TMX2-CTNND1 readthrough (NMD candidate)	TMX2-CTNND1	0,56
CDGSH iron sulfur domain 3	CISD3	0,56
ATP binding cassette subfamily B member 7	ABCB7	0,56
Rho guanine nucleotide exchange factor 26	ARHGEF26	0,56
zinc finger protein 780A	ZNF780A	0,56
kinesin family member 1C	KIF1C	0,56
solute carrier family 39 (zinc transporter), member 3	SLC39A3	0,56
suppressor of variegation 3-9 homolog 2 (Drosophila)	SUV39H2	0,56
acyl-CoA synthetase short-chain family member 3	ACSS3	0,56
rhomboid, veinlet-like 2 (Drosophila)	RHBDL2	0,56
5-methyltetrahydrofolate-homocysteine methyltransferase reductase	MTRR	0,56
chromosome 1 open reading frame 56	C1orf56	0,56
paralemmin	PALM	0,56
myocyte enhancer factor 2C	MEF2C	0,56
Transcript Identified by AceView, Entrez Gene ID(s) 23112	TNRC6B	0,56
hyaluronoglucosaminidase 3	HYAL3	0,56
WW and C2 domain containing 2	WWC2	0,56
proline-rich transmembrane protein 1	PRRT1	0,56
suppressor of cancer cell invasion; golgin A1	SCAI; GOLGA1	0,56
acylphosphatase 2, muscle type	ACYP2	0,56
zinc finger protein 189	ZNF189	0,56
Memczak2013 ANTISENSE, CDS, coding, INTERNAL best transcript NM_153341	RNF19B	0,56
hook microtubule-tethering protein 2	HOOK2	0,56
SR-related CTD-associated factor 11	SCAF11	0,56
6-phosphogluconolactonase	PGLS	0,56
family with sequence similarity 213, member A	FAM213A	0,56
transmembrane protein 117	TMEM117	0,56
LIM domain kinase 2	LIMK2	0,56
arylsulfatase family, member I	ARSI	0,56
forkhead box K2	FOXK2	0,56

chromosome 7 open reading frame 31	C7orf31	0,56
fibrillarin	FBL	0,56
methyltransferase like 21B	METTL21B	0,56
zinc finger protein 780B	ZNF780B	0,56
leukocyte receptor cluster (LRC) member 9	LENG9	0,56
mitogen-activated protein kinase kinase kinase kinase 4	MAP4K4	0,56
pyrophosphatase (inorganic) 2	PPA2	0,56
zinc finger protein 682	ZNF682	0,56
period circadian clock 1; microRNA 6883	PER1; MIR6883	0,56
ubiquitin-like modifier activating enzyme 5	UBA5	0,56
Rab geranylgeranyltransferase, beta subunit; small nucleolar RNA, C/D box 45B; small nucleolar RNA, C/D box 45A; small nucleolar RNA, C/D box 45C	RABGGTB; SNORD45B; SNORD45A; SNORD45C	0,56
Transcript Identified by AceView, Entrez Gene ID(s) 11059	WWP1	0,56
zinc finger protein 772	ZNF772	0,56
Memczak2013 ALT_ACCEPTOR, ALT_DONOR, coding, INTERNAL, intronic best transcript NM_003959	HIP1R	0,56
KIAA2026	KIAA2026	0,56
ribosomal protein S9	RPS9	0,56
cytochrome P450, family 3, subfamily A, polypeptide 7; CYP3A7-CYP3A51P readthrough	CYP3A7; CYP3A7-CYP3A51P	0,56
protein arginine methyltransferase 1	PRMT1	0,56
zinc finger protein 160	ZNF160	0,56
ankyrin repeat domain 13A	ANKRD13A	0,55
histone deacetylase 2	HDAC2	0,55
neural precursor cell expressed, developmentally down-regulated 9	NEDD9	0,55
cardiolipin synthase 1	CRLS1	0,55
ZBED6 C-terminal like	ZBED6CL	0,55
zinc finger protein 282	ZNF282	0,55
leucine zipper transcription factor like 1	LZTFL1	0,55
muscleblind-like splicing regulator 3	MBNL3	0,55
uridine-cytidine kinase 1	UCK1	0,55
growth factor receptor bound protein 7	GRB7	0,55
helicase, lymphoid-specific	HELLS	0,55
mitochondrial ribosomal protein L13	MRPL13	0,55
xylosylprotein beta 1,4-galactosyltransferase, polypeptide 7	B4GALT7	0,55
ribonucleoprotein, PTB-binding 1	RAVER1	0,55
CREB/ATF bZIP transcription factor	CREBZF	0,55
LysM, putative peptidoglycan-binding, domain containing 3	LYSMD3	0,55
dCTP pyrophosphatase 1	DCTPP1	0,55

potassium channel tetramerization domain containing 15	KCTD15	0,55
heterogeneous nuclear ribonucleoprotein A2/B1	HNRNPA2B1	0,55
zinc finger protein 286A	ZNF286A	0,55
neogenin 1	NEO1	0,55
APAF1 interacting protein	APIP	0,55
methylmalonyl-CoA mutase	MUT	0,55
LIM domain only 4	LMO4	0,55
zinc finger, FYVE domain containing 16	ZFYVE16	0,55
dual specificity phosphatase 16	DUSP16	0,55
thioredoxin-like 4A	TXNL4A	0,55
histidine ammonia-lyase	HAL	0,55
NIPA-like domain containing 1	NIPAL1	0,55
hect domain and RLD 2 pseudogene 3	HERC2P3	0,55
NIMA-related kinase 4	NEK4	0,55
major histocompatibility complex, class I, B	HLA-B	0,55
zinc finger protein 233	ZNF233	0,55
G protein-coupled receptor 75	GPR75	0,55
LSM7 homolog, U6 small nuclear RNA and mRNA degradation associated	LSM7	0,55
breast cancer 2, early onset	BRCA2	0,55
chromosome 19 open reading frame 43	C19orf43	0,55
aquaporin 11	AQP11	0,55
secretagogin, EF-hand calcium binding protein	SCGN	0,55
reversion-inducing-cysteine-rich protein with kazal motifs	RECK	0,55
hypermethylated in cancer 2	HIC2	0,55
surfeit 4	SURF4	0,55
fibronectin 1	FN1	0,55
ribosomal protein S3A; small nucleolar RNA, C/D box 73A	RPS3A; SNORD73A	0,55
transferrin receptor 2	TFR2	0,55
UDP glucuronosyltransferase 2 family, polypeptide A2; UDP glucuronosyltransferase 2 family, polypeptide A1, complex locus	UGT2A2; UGT2A1	0,55
metallo-beta-lactamase domain containing 2	MBLAC2	0,55
mitotic spindle organizing protein 1	MZT1	0,55
solute carrier family 39 (zinc transporter), member 10	SLC39A10	0,55
Bet1 golgi vesicular membrane trafficking protein-like	BET1L	0,55
pyruvate dehydrogenase complex, component X	PDHX	0,55
TBC1 domain family, member 12	TBC1D12	0,55
dolichyl-phosphate mannosyltransferase polypeptide 3	DPM3	0,55
uncharacterized protein LOC100652758 [Source:RefSeq peptide;Acc:NP_001265011]; uncharacterized LOC100652758; novel transcript	RP11-54C4.3; LOC100652758	0,55

peptide deformylase (mitochondrial); component of oligomeric golgi complex 8; transmembrane p24 trafficking protein 6	PDF; COG8; TMED6	0,55
nuclear factor I/X (CCAAT-binding transcription factor)	NFIX	0,55
cyclin and CBS domain divalent metal cation transport mediator 2	CNNM2	0,55
Memczak2013 ANTISENSE, CDS, coding, INTERNAL best transcript NM_004719	SCAF11	0,55
crystallin beta-gamma domain containing 3	CRYBG3	0,55
eukaryotic translation initiation factor 2A, 65kDa	EIF2A	0,55
ATP binding cassette subfamily D member 3	ABCD3	0,55
3-hydroxy-3-methylglutaryl-CoA synthase 2 (mitochondrial)	HMGCS2	0,55
fibrillarin	FBL	0,55
ARP3 actin-related protein 3 homolog C (yeast)	ACTR3C	0,55
zinc finger, DHHC-type containing 20	ZDHHC20	0,55
signal peptidase complex subunit 2	SPCS2	0,55
t-complex 10-like 2	TCP10L2	0,55
palmitoyl-protein thioesterase 1	PPT1	0,55
prolyl 3-hydroxylase 3	P3H3	0,55
KIAA1109	KIAA1109	0,55
GLTSCR1-like	GLTSCR1L	0,55
COBW domain containing 2	CBWD2	0,55
caspase activity and apoptosis inhibitor 1	CAAP1	0,55
programmed cell death 7	PDCD7	0,55
osteopetrosis associated transmembrane protein 1	OSTM1	0,55
myomesin 1	MYOM1	0,55
formimidoyltransferase cyclodeaminase	FTCD	0,55
ATP/GTP binding protein-like 3	AGBL3	0,55
small nuclear ribonucleoprotein polypeptide A	SNRPA1	0,55
AT hook containing transcription factor 1	AHCTF1	0,55
ribosome binding protein 1	RRBP1	0,55
ubiquitin specific peptidase 37	USP37	0,55
patched domain containing 4	PTCHD4	0,55
family with sequence similarity 185, member A	FAM185A	0,55
WD repeat containing, antisense to TP73	WRAP73	0,55
histone cluster 2, H2ac	HIST2H2AC	0,54
zinc finger, BED-type containing 8	ZBED8	0,54
lamin B2; microRNA 7108	LMNB2; MIR7108	0,54
neuroepithelial cell transforming 1	NET1	0,54
zinc finger, BED-type containing 3	ZBED3	0,54
heat shock protein 90kDa beta (Grp94), member 1; microRNA 3652	HSP90B1; MIR3652	0,54
solute carrier family 33 (acetyl-CoA transporter), member 1	SLC33A1	0,54

allograft inflammatory factor 1-like	AIF1L	0,54
NUS1 dehydrodolichyl diphosphate synthase subunit	NUS1	0,54
transmembrane protein 245	TMEM245	0,54
small nucleolar RNA, C/D box 102; small nucleolar RNA, H/ACA box 27; ribosomal protein L21	SNORD102; SNORA27; RPL21	0,54
component of oligomeric golgi complex 3	COG3	0,54
histone cluster 1, H2bm	HIST1H2BM	0,54
required for meiotic nuclear division 5 homolog A	RMND5A	0,54
pyridoxal (pyridoxine, vitamin B6) kinase	PDXK	0,54
surfeit 4	SURF4	0,54
hydroxylysine kinase	HYKK	0,54
additional sex combs like transcriptional regulator 1	ASXL1	0,54
HAUS augmin like complex subunit 1	HAUS1	0,54
staphylococcal nuclease and tudor domain containing 1; SND1 intronic transcript 1	SND1; SND1-IT1	0,54
surfeit 4	SURF4	0,54
solute carrier family 2 (facilitated glucose transporter), member 4	SLC2A4	0,54
fibrillarin	FBL	0,54
round spermatid basic protein 1	RSBN1	0,54
janus kinase and microtubule interacting protein 2	JAKMIP2	0,54
tripartite motif containing 4	TRIM4	0,54
major histocompatibility complex, class I, C	HLA-C	0,54
glutathione S-transferase alpha 2	GSTA2	0,54
FERM, ARH/RhoGEF and pleckstrin domain protein 2	FARP2	0,54
TAF11 RNA polymerase II, TATA box binding protein (TBP)-associated factor, 28kDa	TAF11	0,54
fibroblast growth factor receptor 1	FGFR1	0,54
KIAA0141	KIAA0141	0,54
ras homolog family member J	RHOJ	0,54
cysteine rich with EGF-like domains 2	CRELD2	0,54
interleukin 1 receptor associated kinase 1 binding protein 1	IRAK1BP1	0,54
HECT and RLD domain containing E3 ubiquitin protein ligase 2	HERC2	0,54
EPM2A (laforin) interacting protein 1	EPM2AIP1	0,54
cilia and flagella associated protein 20	CFAP20	0,54
homeobox containing 1	HMBOX1	0,54
inversin	INVS	0,54
zinc finger protein 248	ZNF248	0,54
enhancer of zeste 2 polycomb repressive complex 2 subunit	EZH2	0,54
zinc finger protein 429	ZNF429	0,54
spermatogenesis associated 25	SPATA25	0,54
C-terminal binding protein 1	CTBP1	0,54

heterogeneous nuclear ribonucleoprotein L-like	HNRNPLL	0,54
leucine rich repeat containing 61	LRRC61	0,54
v-akt murine thymoma viral oncogene homolog 2	AKT2	0,54
histone cluster 1, H2bi	HIST1H2BI	0,54
acetyl-CoA acyltransferase 2	ACAA2	0,54
ectonucleoside triphosphate diphosphohydrolase 6 (putative)	ENTPD6	0,54
ST6 (alpha-N-acetyl-neuraminyl-2,3-beta-galactosyl-1,3)-N-acetylgalactosaminide alpha-2,6-sialyltransferase 6	ST6GALNAC6	0,54
cadherin, EGF LAG seven-pass G-type receptor 2	CELSR2	0,54
multiple endocrine neoplasia I	MEN1	0,54
translocase of outer mitochondrial membrane 20 homolog (yeast)	TOMM20	0,54
Memczak2013 ANTISENSE, CDS, coding, INTERNAL, UTR3 best transcript NM_001752	CAT	0,54
small nuclear ribonucleoprotein polypeptide F	SNRPF	0,54
pleckstrin homology domain containing, family A (phosphoinositide binding specific) member 8	PLEKHA8	0,54
deoxyuridine triphosphatase	DUT	0,54
DnaJ (Hsp40) homolog, subfamily A, member 3	DNAJA3	0,54
HNF1 homeobox A	HNF1A	0,54
uncharacterized LOC730183; Transcript Identified by AceView; novel transcript, antisense to SRCAP	LOC730183; RP11-146F11.1; hitema	0,54
Rap guanine nucleotide exchange factor 6	RAPGEF6	0,54
neuroligin 4, X-linked	NLGN4X	0,54
GID complex subunit 8	GID8	0,54
serpin peptidase inhibitor, clade A (alpha-1 antiproteinase, antitrypsin), member 9	SERPINA9	0,54
1-acylglycerol-3-phosphate O-acyltransferase 2	AGPAT2	0,54
leucine zipper and CTNNBIP1 domain containing	LZIC	0,54
trafficking protein particle complex 5	TRAPPC5	0,54
coiled-coil-helix-coiled-coil-helix domain containing 1	CHCHD1	0,54
sirtuin 5	SIRT5	0,54
enolase 3 (beta, muscle)	ENO3	0,54
TLC domain containing 1	TLCD1	0,54
Transcript Identified by AceView, Entrez Gene ID(s) 3069	HDLBP	0,54
BCL2/adenovirus E1B 19kDa interacting protein 3-like	BNIP3L	0,54
solute carrier family 25 (pyrimidine nucleotide carrier), member 36	SLC25A36	0,54
farnesyltransferase, CAAX box, beta	FNTB	0,54
DEP domain containing 1	DEPDC1	0,54
mortality factor 4 like 2	MORF4L2	0,54
regulation of nuclear pre-mRNA domain containing 1A	RPRD1A	0,54

mitogen-activated protein kinase kinase kinase 11	MAP3K11	0,54
nuclear factor, interleukin 3 regulated	NFIL3	0,54
protein tyrosine phosphatase, receptor type, N polypeptide 2	PTPRN2	0,54
cancer susceptibility candidate 4	CASC4	0,54
signal transducer and activator of transcription 2	STAT2	0,54
major facilitator superfamily domain containing 8	MFSD8	0,53
zinc finger with KRAB and SCAN domains 1	ZKSCAN1	0,53
utrophin	UTRN	0,53
von Willebrand factor A domain containing 8; microRNA 5006	VWA8; MIR5006	0,53
serum/glucocorticoid regulated kinase 2	SGK2	0,53
motor neuron and pancreas homeobox 1	MNX1	0,53
zinc finger protein 503	ZNF503	0,53
eukaryotic translation initiation factor 2-alpha kinase 3	EIF2AK3	0,53
acyl-CoA binding domain containing 4	ACBD4	0,53
minichromosome maintenance complex component 5	MCM5	0,53
aldehyde dehydrogenase 5 family, member A1	ALDH5A1	0,53
transmembrane protein 223	TMEM223	0,53
major facilitator superfamily domain containing 14A	MFSD14A	0,53
breast cancer metastasis-suppressor 1-like	BRMS1L	0,53
carcinoembryonic antigen-related cell adhesion molecule 1 (biliary glycoprotein)	CEACAM1	0,53
ubiquitin specific peptidase 30	USP30	0,53
HECT, UBA and WWE domain containing 1, E3 ubiquitin protein ligase	HUWE1	0,53
BLOC1S5-TXNDC5 readthrough (NMD candidate)	BLOC1S5-TXNDC5	0,53
RAP1 GTPase activating protein	RAP1GAP	0,53
poly(A) binding protein interacting protein 2 [Source:EntrezGene;Acc:51247]; novel transcript, antisense to PAIP2	PAIP2; CTB-43P18.1	0,53
copper chaperone for superoxide dismutase	CCS	0,53
centrosomal protein 350kDa	CEP350	0,53
COBW domain containing 1	CBWD1	0,53
zinc finger protein 780A	ZNF780A	0,53
long intergenic non-protein coding RNA 493	LINC00493	0,53
POU class 2 associating factor 1	POU2AF1	0,53
KAT8 regulatory NSL complex subunit 1 like	KANSL1L	0,53
beta-1,4-N-acetyl-galactosaminyl transferase 3	B4GALNT3	0,53
translocase of outer mitochondrial membrane 7 homolog (yeast)	TOMM7	0,53
selenophosphate synthetase 2	SEPHS2	0,53
ribosomal protein S14	RPS14	0,53
ankyrin repeat domain 57 pseudogene	LOC389834; AL354822.1	0,53

arylsulfatase family, member K	ARSK	0,53
AT rich interactive domain 4A (RBP1-like)	ARID4A	0,53
ALG5, dolichyl-phosphate beta-glucosyltransferase	ALG5	0,53
sterol regulatory element binding transcription factor 1	SREBF1	0,53
chromosome 17 open reading frame 100	C17orf100	0,53
oncostatin M receptor	OSMR	0,53
zinc finger protein 511	ZNF511	0,53
gamma-secretase activating protein	GSAP	0,53
transmembrane protein 214	TMEM214	0,53
ribosomal protein L10a	RPL10A	0,53
ribosomal protein L21	RPL21	0,53
BRI3 binding protein	BRI3BP	0,53
coiled-coil domain containing 117	CCDC117	0,53
low density lipoprotein receptor class A domain containing 4	LDLRAD4	0,53
RNA binding motif protein 8A	RBM8A	0,53
protein phosphatase, Mg2+/Mn2+ dependent, 1K	PPM1K	0,53
polymerase (RNA) III (DNA directed) polypeptide D, 44kDa	POLR3D	0,53
nuclear factor of activated T-cells 5, tonicity-responsive	NFAT5	0,53
aldehyde dehydrogenase 1 family, member A1	ALDH1A1	0,53
Yip1 domain family member 2	YIPF2	0,53
ATP binding cassette subfamily G member 5	ABCG5	0,53
myeloid differentiation primary response 88	MYD88	0,53
cancer susceptibility candidate 10	CASC10	0,53
inhibitor of growth family member 3	ING3	0,53
arginyltransferase 1	ATE1	0,53
chromodomain helicase DNA binding protein 1-like	CHD1L	0,53
TYRO3 protein tyrosine kinase	TYRO3	0,53
histone cluster 2, H2bf	HIST2H2BF	0,53
ATP synthase, H+ transporting, mitochondrial Fo complex subunit C3 (subunit 9)	ATP5G3	0,53
programmed cell death 4 (neoplastic transformation inhibitor); microRNA 4680	PDCD4; MIR4680	0,53
solute carrier family 51, alpha subunit	SLC51A	0,53
Memczak2013 ANTISENSE, CDS, coding, INTERNAL best transcript NM_014709	USP34	0,53
mitochondrial transcription termination factor 2	MTERF2	0,53
histone cluster 1, H3c	HIST1H3C	0,53
SRY box 8	SOX8	0,53
COBW domain containing 3	CBWD3	0,53
stromal cell derived factor 4	SDF4	0,53
Zhang2013 ALT_ACCEPTOR, ALT_DONOR, coding, INTERNAL, intronic best transcript NM_173595	ANKRD52	0,53
casein kinase 1, gamma 2	CSNK1G2	0,53

chromosome 17 open reading frame 75	C17orf75	0,53
chromobox homolog 3	CBX3	0,53
WAP, follistatin/kazal, immunoglobulin, kunitz and netrin domain containing 1	WFIKKN1	0,53
SCAN domain containing 1	SCAND1	0,53
DEP domain containing MTOR-interacting protein	DEPTOR	0,53
family with sequence similarity 8, member A1	FAM8A1	0,53
glucosidase, alpha; neutral AB	GANAB	0,53
chromosome 6 open reading frame 106	C6orf106	0,53
MRS2 magnesium transporter	MRS2	0,53
Memczak2013 ALT_ACCEPTOR, ALT_DONOR, coding, INTERNAL, intronic best transcript NM_021156	TMX4	0,53
lysine (K)-specific demethylase 6A	KDM6A	0,53
transmembrane protein 57	TMEM57	0,53
golgin, RAB6-interacting	GORAB	0,53
suppressor of cytokine signaling 5	SOCS5	0,53
ATP synthase mitochondrial F1 complex assembly factor 1	ATPAF1	0,53
poliovirus receptor-related 2 (herpesvirus entry mediator B)	PVRL2	0,53
CCAAT/enhancer binding protein (C/EBP), alpha	CEBPA	0,52
Yae1 domain containing 1	YAE1D1	0,52
tetratricopeptide repeat domain 13	TTC13	0,52
intraflagellar transport 172	IFT172	0,52
XRCC6 binding protein 1	XRCC6BP1	0,52
SMAD family member 6	SMAD6	0,52
unc-119 lipid binding chaperone B; microRNA 4700	UNC119B; MIR4700	0,52
Transcript Identified by AceView, Entrez Gene ID(s) 63898	SH2D4A	0,52
sorbitol dehydrogenase	SORD	0,52
sirtuin 3	SIRT3	0,52
DNA fragmentation factor, 40kDa, beta polypeptide (caspase-activated DNase)	DFFB	0,52
insulin-like growth factor 1 receptor	IGF1R	0,52
chromosome 8 open reading frame 49	C8orf49	0,52
NAD synthetase 1	NADSYN1	0,52
TruB pseudouridine (psi) synthase family member 1	TRUB1	0,52
Sec23 homolog A, COPII coat complex component	SEC23A	0,52
mitogen-activated protein kinase kinase kinase 13	MAP3K13	0,52
ephrin-A2	EFNA2	0,52
jade family PHD finger 3	JADE3	0,52
serine palmitoyltransferase, small subunit A	SPTSSA	0,52
transmembrane protein 18	TMEM18	0,52
glyoxylate reductase/hydroxypyruvate reductase	GRHPR	0,52

dedicator of cytokinesis 6	DOCK6	0,52
tetraspanin 3	TSPAN3	0,52
ornithine carbamoyltransferase	OTC	0,52
alpha thalassemia/mental retardation syndrome X-linked	ATRX	0,52
synovial sarcoma, X breakpoint 2 interacting protein	SSX2IP	0,52
ribosomal protein S5	RPS5	0,52
small integral membrane protein 8	SMIM8	0,52
bromodomain containing 3	BRD3	0,52
tripartite motif containing 10	TRIM10	0,52
acyl-CoA synthetase medium-chain family member 5	ACSM5	0,52
heparan sulfate 2-O-sulfotransferase 1	HS2ST1	0,52
NIMA-related kinase 3	NEK3	0,52
ZFP69 zinc finger protein B	ZFP69B	0,52
small nuclear ribonucleoprotein polypeptide N; SNRPN upstream reading frame; small nucleolar RNA, C/D box 107; Prader Willi/Angelman region RNA, SNRPN neighbor	SNRPN; SNURF; SNORD107; PWARSN	0,52
COX18 cytochrome c oxidase assembly factor	COX18	0,52
kinesin family member 13B	KIF13B	0,52
beta-transducin repeat containing E3 ubiquitin protein ligase	BTRC	0,52
THO complex 1	THOC1	0,52
ATPase, Na+/K+ transporting, beta 3 polypeptide	ATP1B3	0,52
dimethylarginine dimethylaminohydrolase 2	DDAH2	0,52
colony stimulating factor 3 receptor	CSF3R	0,52
arginase 1	ARG1	0,52
transmembrane p24 trafficking protein 5	TMED5	0,52
Memczak2013 ANTISENSE, CDS, coding, INTERNAL best transcript NM_001206844	TGOLN2	0,52
A kinase (PRKA) anchor protein 9	AKAP9	0,52
protein tyrosine phosphatase type IVA, member 3	PTP4A3	0,52
MAGE family member H1	MAGEH1	0,52
cyclin Y	CCNY	0,52
adenomatosis polyposis coli down-regulated 1	APCDD1	0,52
calpain 7	CAPN7	0,52
adaptor protein, phosphotyrosine interaction, PH domain and leucine zipper containing 1	APPL1	0,52
HECT and RLD domain containing E3 ubiquitin protein ligase family member 1	HERC1	0,52
CD83 molecule	CD83	0,52
septin 8	sept-08	0,52
solute carrier family 23 (ascorbic acid transporter), member 1	SLC23A1	0,52
metastasis associated 1 family member 3	MTA3	0,52

zinc finger, matrin-type 3	ZMAT3	0,52
insulin receptor	INSR	0,52
MYC induced nuclear antigen	MINA	0,52
chromosome 8 open reading frame 4	C8orf4	0,52
THAP domain containing, apoptosis associated protein 2	THAP2	0,52
SPC25, NDC80 kinetochore complex component	SPC25	0,52
Homo sapiens ribosomal protein L21, mRNA (cDNA clone MGC:2150 IMAGE:3543702), complete cds.; Homo sapiens ribosomal protein L21, mRNA (cDNA clone MGC:4136 IMAGE:2964441), complete cds.; Homo sapiens ribosomal protein L21, mRNA (cDNA clone MGC:71252 IMAGE:6084268), complete cds.; Homo sapiens ribosomal protein L21, mRNA (cDNA clone MGC:88165 IMAGE:4281405), complete cds.; Homo sapiens ribosomal protein L21, mRNA (cDNA clone MGC:88326 IMAGE:4248655), complete cds.; Homo sapiens ribosomal protein L21, mRNA (cDNA clone MGC:88334 IMAGE:3931616), complete cds.; Homo sapiens ribosomal protein L21, mRNA (cDNA clone MGC:88581 IMAGE:4901492), complete cds.; Homo sapiens ribosomal protein L21, mRNA (cDNA clone MGC:104275 IMAGE:6724053), complete cds.	RPL21	0,52
complement component 8, beta polypeptide	C8B	0,52
malectin	MLEC	0,52
coiled-coil domain containing 65	CCDC65	0,52
suppressor of IKBKE 1	SIKE1	0,52
interferon-induced protein 35	IFI35	0,52
family with sequence similarity 126, member A	FAM126A	0,52
alcohol dehydrogenase 4 (class II), pi polypeptide	ADH4	0,52
phosphoinositide-3-kinase, regulatory subunit 3 (gamma)	PIK3R3	0,52
hypoxia up-regulated 1	HYOU1	0,51
histone deacetylase 4	HDAC4	0,51
ribosomal protein S6	RPS6	0,51
selenoprotein T; SELT protein; Selenoprotein T; cDNA FLJ90525 fis, clone NT2RP4001001, highly similar to Selenoprotein T [Source:UniProtKB/TrEMBL;Acc:Q6IAK0]; Transcript Identified by AceView, Entrez Gene ID(s) 51714	SELT; SELT.1	0,51
REV3 like, DNA directed polymerase zeta catalytic subunit	REV3L	0,51
pleiomorphic adenoma gene 1	PLAG1	0,51
TEA domain family member 2	TEAD2	0,51
apolipoprotein L, 6	APOL6	0,51
ATP binding cassette subfamily C member 6	ABCC6	0,51

collagen, type XI, alpha 2	COL11A2	0,51
prostate tumor overexpressed 1; microRNA 4749	PTOV1; MIR4749	0,51
carnitine O-acetyltransferase	CRAT	0,51
Transcript Identified by AceView, Entrez Gene ID(s) 5771	PTPN2	0,51
dolichyl-diphosphooligosaccharide--protein glycosyltransferase subunit (non-catalytic)	DDOST	0,51
nudix hydrolase 3	NUDT3	0,51
chromosome 2 open reading frame 68	C2orf68	0,51
BCL2-associated athanogene 4	BAG4	0,51
kelch-like ECH-associated protein 1	KEAP1	0,51
mitochondrial ribosomal protein L17	MRPL17	0,51
LDL receptor related protein 6	LRP6	0,51
methyl-CpG binding domain protein 3	MBD3	0,51
phosphoenolpyruvate carboxykinase 2 (mitochondrial)	PCK2	0,51
solute carrier family 2 (facilitated glucose transporter), member 13	SLC2A13	0,51
Jeck2013 ALT_ACCEPTOR, ALT_DONOR, coding, INTERNAL, intronic best transcript NM_030796	VOPP1	0,51
family with sequence similarity 213, member B	FAM213B	0,51
ARP3 actin-related protein 3 homolog B (yeast)	ACTR3B	0,51
lysine (K)-specific demethylase 4B	KDM4B	0,51
dyskeratosis congenita 1, dyskerin; small nucleolar RNA, H/ACA box 36A; small nucleolar RNA, H/ACA box 56; microRNA 664b	DKC1; SNORA36A; SNORA56; MIR664B	0,51
suppressor of cytokine signaling 7	SOCS7	0,51
Fanconi anemia complementation group L	FANCL	0,51
retinoblastoma binding protein 9	RBBP9	0,51
Jeck2013 ALT_ACCEPTOR, ALT_DONOR, coding, INTERNAL, intronic best transcript NM_002841	PTPRG	0,51
Wolf-Hirschhorn syndrome candidate 1	WHSC1	0,51
dihydrolipoamide branched chain transacylase E2	DBT	0,51
glutathione S-transferase mu 4	GSTM4	0,51
von Hippel-Lindau binding protein 1	VBP1	0,51
N-acyl phosphatidylethanolamine phospholipase D	NAPEPLD	0,51
MID1 interacting protein 1	MID1IP1	0,51
homeobox D3	HOXD3	0,51
arginyl-tRNA synthetase 2, mitochondrial	RARS2	0,51
CKLF-like MARVEL transmembrane domain containing 4	CMTM4	0,51
zinc finger protein 395	ZNF395	0,51
acyl-CoA synthetase short-chain family member 2	ACSS2	0,51
lysophosphatidylglycerol acyltransferase 1	LPGAT1	0,51
distal-less homeobox 4	DLX4	0,51
TBC1 domain family, member 24	TBC1D24	0,51

DNA methyltransferase 1 associated protein 1	DMAP1	0,51
dystroglycan 1 (dystrophin-associated glycoprotein 1)	DAG1	0,51
jumping translocation breakpoint	JTB	0,51
KIAA1715	KIAA1715	0,51
FSHD region gene 1 pseudogene	LOC283788; AL592183.1	0,51
prolyl 4-hydroxylase, alpha polypeptide II	P4HA2	0,51
family with sequence similarity 53, member B	FAM53B	0,51
ArfGAP with coiled-coil, ankyrin repeat and PH domains 3	ACAP3	0,51
hook microtubule-tethering protein 1	HOOK1	0,51
integrator complex subunit 2	INTS2	0,51
dehydrogenase E1 and transketolase domain containing 1	DHTKD1	0,51
K(lysine) acetyltransferase 8	KAT8	0,51
LEO1 homolog, Paf1/RNA polymerase II complex component	LEO1	0,51
F-box protein 4	FBXO4	0,51
zinc finger, CCHC domain containing 11	ZCCHC11	0,51
histone cluster 1, H2ai	HIST1H2AI	0,51
CD14 molecule	CD14	0,51
MYC binding protein; gap junction protein alpha 9	MYCBP; GJA9	0,51
Transcript Identified by AceView, Entrez Gene ID(s) 6660	SOX5	0,51
claudin 1	CLDN1	0,51
post-GPI attachment to proteins 2	PGAP2	0,51
death-associated protein	DAP	0,51
core-binding factor, runt domain, alpha subunit 2; translocated to, 2	CBFA2T2	0,51
zinc finger protein 69	ZNF69	0,51
cytoskeleton-associated protein 4	CKAP4	0,51
serine/threonine kinase-like domain containing 1 [Source:HGNC Symbol;Acc:HGNC:28669]	STKLD1	0,51
NCK interacting protein with SH3 domain	NCKIPSD	0,51
sprouty RTK signaling antagonist 2	SPRY2	0,51
PET117 homolog; CSRP2 binding protein	PET117; CSRP2BP	0,51
RNA binding motif protein 10	RBM10	0,51
lipocalin 15	LCN15	0,51
aurora kinase B	AURKB	0,51
chromosome 9 open reading frame 64	C9orf64	0,51
cilia and flagella associated protein 97	CFAP97	0,51
ADP-ribosylation factor like GTPase 17B; ADP-ribosylation factor like GTPase 17A	ARL17B; ARL17A	0,50
Sec61 translocon alpha 1 subunit	SEC61A1	0,50
nuclear receptor subfamily 6, group A, member 1	NR6A1	0,50

Transcript Identified by AceView, Entrez Gene ID(s) 8565	YARS	0,50
hypoxia up-regulated 1	HYOU1	0,50
small nuclear ribonucleoprotein polypeptides B and B1	SNRPB	0,50
inositol monophosphatase domain containing 1	IMPAD1	0,50
dehydrogenase/reductase (SDR family) member 13	DHRS13	0,50
fetuin B	FETUB	0,50
FRY microtubule binding protein	FRY	0,50
nipsnap homolog 1 (C. elegans)	NIPSNAP1	0,50
death-associated protein kinase 1	DAPK1	0,50
cytochrome b5 domain containing 2	CYB5D2	0,50
suppression of tumorigenicity 7; ST7 overlapping transcript 4; ST7 overlapping transcript 3; microRNA 6132	ST7; ST7-OT4; ST7-OT3; MIR6132	0,50
ataxin 7-like 3B	ATXN7L3B	0,50
Dexi homolog (mouse)	DEXI	0,50
GTP cyclohydrolase I feedback regulator	GCHFR	0,50
histone cluster 1, H2aj	HIST1H2AJ	0,50
naked cuticle homolog 1 (Drosophila)	NKD1	0,50
aldehyde dehydrogenase 16 family, member A1	ALDH16A1	0,50
proteasome activator subunit 1	PSME1	0,50
helicase with zinc finger	HELZ	0,50
chromosome 2 open reading frame 15	C2orf15	0,50
HAUS augmin like complex subunit 4; microRNA 4707	HAUS4; MIR4707	0,50
calcium/calmodulin-dependent protein kinase kinase 2, beta	CAMKK2	0,50
Alstrom syndrome protein 1	ALMS1	0,50
inter-alpha-trypsin inhibitor heavy chain family, member 4	ITIH4	0,50
mitochondrial ribosomal protein S26	MRPS26	0,50
plexin B1	PLXNB1	0,50
G-2 and S-phase expressed 1	GTSE1	0,50
N-acetylglucosaminidase, alpha	NAGLU	0,50
SUMO-interacting motifs containing 1	SIMC1	0,50
KCTD21 antisense RNA 1	KCTD21-AS1	0,50
BLOC-1 related complex subunit 7	BORCS7	0,50
polyamine-modulated factor 1; PMF1-BGLAP readthrough; bone gamma-carboxyglutamate (gla) protein	PMF1; PMF1-BGLAP; BGLAP	0,50
centrosomal protein 57kDa	CEP57	0,50
immediate early response 3 interacting protein 1	IER3IP1	0,50
cyclin G1	CCNG1	0,50
ADP-ribosylation factor like GTPase 5A	ARL5A	0,50
zinc finger protein 3	ZNF3	0,50
TGFB-induced factor homeobox 2	TGIF2	0,50

signal sequence receptor, delta	SSR4	0,50
A kinase (PRKA) anchor protein 1	AKAP1	0,50
alkylglycerone phosphate synthase	AGPS	0,50
AU RNA binding protein/enoyl-CoA hydratase	AUH	0,50
guanine nucleotide binding protein (G protein), alpha 11 (Gq class)	GNA11	0,50
mitochondrial ribosomal protein L23	MRPL23	0,50
ADAM metallopeptidase domain 10	ADAM10	0,50
mediator complex subunit 4	MED4	0,50
glycine amidinotransferase (L-arginine:glycine amidinotransferase)	GATM	0,50
ash2 (absent, small, or homeotic)-like (Drosophila)	ASH2L	0,50
retinoid X receptor alpha; microRNA 4669	RXRA; MIR4669	0,50
abhydrolase domain containing 14A	ABHD14A	0,50
nuclear receptor coactivator 4	NCOA4	0,50
SET domain containing 5	SETD5	0,50
solute carrier family 39, member 9	SLC39A9	0,50
frizzled class receptor 5	FZD5	0,50
ATP synthase, H+ transporting, mitochondrial F1 complex, alpha subunit 1, cardiac muscle	ATP5A1	0,50
transmembrane protein 106A	TMEM106A	0,50
ribosomal protein S19	RPS19	0,50
phosphodiesterase 9A	PDE9A	0,50
family with sequence similarity 57, member A	FAM57A	0,50
Zhang2013 ALT_ACCEPTOR, ALT_DONOR, coding, INTERNAL, intronic best transcript NM_152227	SNX5	0,50
Homo sapiens mitochondrial ribosomal protein L23 (MRPL23), mRNA.; 39S ribosomal protein L23, mitochondrial-like; Homo sapiens mitochondrial ribosomal protein L23, mRNA (cDNA clone MGC:12795 IMAGE:4298000), complete cds.	MRPL23; LOC102724828; AC004556.1	0,50
solute carrier family 36 (proton/amino acid symporter), member 4	SLC36A4	0,50
transmembrane protein 165	TMEM165	0,50
solute carrier family 7 (amino acid transporter light chain, bo,+ system), member 9	SLC7A9	0,50
myeloid/lymphoid or mixed-lineage leukemia; translocated to, 4	MLLT4	0,50
zinc finger, CCHC domain containing 14	ZCCHC14	0,50
chromodomain helicase DNA binding protein 6	CHD6	0,50
ADP-ribosylation factor GTPase activating protein 3	ARFGAP3	0,50
KIAA0895	KIAA0895	0,50
protocadherin beta 5	PCDHB5	0,49
v-akt murine thymoma viral oncogene homolog 1	AKT1	0,49
polymerase (RNA) II (DNA directed) polypeptide H	POLR2H	0,49

DnaJ (Hsp40) homolog, subfamily C, member 22	DNAJC22	0,49
orosomucoid 2	ORM2	0,49
Raf-1 proto-oncogene, serine/threonine kinase	RAF1	0,49
fibulin 1	FBLN1	0,49
Jeck2013 ALT_DONOR, coding, INTERNAL, intronic best transcript NM_152227	SNX5	0,49
paralemmin 3	PALM3	0,49
family with sequence similarity 195, member B	FAM195B	0,49
solute carrier family 26 (anion exchanger), member 1	SLC26A1	0,49
gasdermin D	GSDMD	0,49
inter-alpha-trypsin inhibitor heavy chain 2	ITIH2	0,49
pecanex-like 3 (Drosophila)	PCNXL3	0,49
cell migration inducing protein, hyaluronan binding	CEMIP	0,49
signal peptidase complex subunit 2	SPCS2	0,49
microRNA 1199	MIR1199	0,49
REX4 homolog, 3'-5' exonuclease [Source:HGNC Symbol;Acc:HGNC:12820]	REXO4	0,49
histone cluster 1, H4j	HIST1H4J	0,49
dihydrolipoamide S-acetyltransferase	DLAT	0,49
enoyl-CoA delta isomerase 2	ECI2	0,49
carbonic anhydrase IX	CA9	0,49
KIAA1919	KIAA1919	0,49
transformation/transcription domain-associated protein	TRRAP	0,49
alcohol dehydrogenase 5 (class III), chi polypeptide	ADH5	0,49
phosphatidylinositol glycan anchor biosynthesis class N	PIGN	0,49
ATPase, aminophospholipid transporter, class I, type 8B, member 2	ATP8B2	0,49
coiled-coil domain containing 138	CCDC138	0,49
ATPase, class V, type 10A	ATP10A	0,49
FUS RNA binding protein	FUS	0,49
dihydropyrimidine dehydrogenase	DPYD	0,49
adenylate cyclase 1 (brain)	ADCY1	0,49
nucleoporin 160kDa	NUP160	0,49
NFKB activating protein	NKAP	0,49
family with sequence similarity 156, member B; family with sequence similarity 156, member A	FAM156B; FAM156A	0,49
phosphatidylinositol glycan anchor biosynthesis class F	PIGF	0,49
SEC24 homolog A, COPII coat complex component	SEC24A	0,49
SEC16 homolog B, endoplasmic reticulum export factor	SEC16B	0,49
ATPase family, AAA domain containing 5	ATAD5	0,49
mesoderm induction early response 1, family member 3	MIER3	0,49
trafficking protein particle complex 4	TRAPPC4	0,49
carbonyl reductase 4	CBR4	0,49
ring finger protein 19A, RBR E3 ubiquitin protein ligase	RNF19A	0,49
dishevelled associated activator of morphogenesis 1	DAAM1	0,49

pituitary tumor-transforming 1	PTTG1	0,49
selenophosphate synthetase 1	SEPHS1	0,49
ring finger protein 187	RNF187	0,49
zinc finger, RAN-binding domain containing 3	ZRANB3	0,49
zinc finger protein 460	ZNF460	0,49
5-methyltetrahydrofolate-homocysteine methyltransferase	MTR	0,49
transmembrane protein 140	TMEM140	0,49
solute carrier family 25, member 40	SLC25A40	0,49
general transcription factor IIi	GTF2I	0,49
homeobox and leucine zipper encoding	HOMEZ	0,49
ubiquitin specific peptidase 13 (isopeptidase T-3)	USP13	0,49
LEM domain containing 2	LEMD2	0,49
Ral GEF with PH domain and SH3 binding motif 1	RALGPS1	0,49
ATM serine/threonine kinase	ATM	0,49
solute carrier family 25, member 30	SLC25A30	0,49
NADH dehydrogenase (ubiquinone) complex I, assembly factor 6	NDUFAF6	0,49
ceramide synthase 4	CERS4	0,49
family with sequence similarity 234, member B	FAM234B	0,49
T-box 2	TBX2	0,49
zinc finger protein 782	ZNF782	0,49
ankyrin repeat domain 52	ANKRD52	0,49
minichromosome maintenance complex component 2	MCM2	0,49
thioredoxin domain containing 16	TXNDC16	0,49
protein tyrosine phosphatase, mitochondrial 1	PTPMT1	0,49
phosphoinositide-3-kinase, regulatory subunit 1 (alpha)	PIK3R1	0,49
chromobox homolog 8	CBX8	0,49
ATP synthase, H+ transporting, mitochondrial Fo complex subunit E	ATP5I	0,49
chromosome 21 open reading frame 140	C21orf140	0,49
chromosome 21 open reading frame 140	C21orf140	0,49
ubiquitin specific peptidase 34	USP34	0,49
dystrophin	DMD	0,49
CD320 molecule	CD320	0,49
iron responsive element binding protein 2	IREB2	0,49
UDP-Gal:betaGal beta 1,3-galactosyltransferase 6	B3GALT6	0,49
serine peptidase inhibitor, Kazal type 1	SPINK1	0,49
chromosome 3 open reading frame 33	C3orf33	0,49
mutS homolog 2	MSH2	0,49

selenoprotein O; selenoprotein O [Source:EntrezGene;Acc:83642]; Salzman2013 ANNOTATED, CDS, coding, INTERNAL, OVCODE, OVERLAPTX, OVEXON best transcript NM_031454; Salzman2013 ANNOTATED, CDS, coding, OVCODE, OVERLAPTX, OVEXON, UTR3 best transcript NM_031454; Transcript Identified by AceView, Entrez Gene ID(s) 83642, RefSeq ID(s) NM_031454	SELO	0,49
interleukin 22 receptor, alpha 1	IL22RA1	0,49
family with sequence similarity 13, member B	FAM13B	0,48
apoptosis-inducing factor, mitochondrion-associated, 3	AIFM3	0,48
SWI/SNF related, matrix associated, actin dependent regulator of chromatin, subfamily d, member 2	SMARCD2	0,48
Bardet-Biedl syndrome 5	BBS5	0,48
nucleoside-triphosphatase, cancer-related	NTPCR	0,48
short coiled-coil protein	SCOC	0,48
phospholipid phosphatase 3	PLPP3	0,48
zinc finger protein 217	ZNF217	0,48
Transcript Identified by AceView, Entrez Gene ID(s) 8660	IRS2	0,48
cytochrome c oxidase assembly factor 5	COA5	0,48
poly(ADP-ribose) polymerase family member 12	PARP12	0,48
pygopus family PHD finger 2	PYGO2	0,48
solute carrier family 7 (neutral amino acid transporter light chain, asc system), member 10	SLC7A10	0,48
alkB homolog 5, RNA demethylase	ALKBH5	0,48
phytanoyl-CoA 2-hydroxylase	PHYH	0,48
homeodomain interacting protein kinase 2	HIPK2	0,48
MAM and LDL receptor class A domain containing 1	MALRD1	0,48
serum/glucocorticoid regulated kinase family, member 3; C8orf44-SGK3 readthrough	SGK3; C8orf44-SGK3	0,48
surfeit 4	SURF4	0,48
UHRF1 binding protein 1	UHRF1BP1	0,48
DDB1 and CUL4 associated factor 11	DCAF11	0,48
adenylate cyclase 6; microRNA 4701	ADCY6; MIR4701	0,48
zinc finger protein 219	ZNF219	0,48
talin 2	TLN2	0,48
inner centromere protein	INCENP	0,48
solute carrier family 35 (UDP-GlcNAc/UDP-glucose transporter), member D2	SLC35D2	0,48
glucoside xylosyltransferase 2	GXYLT2	0,48
musashi RNA binding protein 2	MSI2	0,48
nudix hydrolase 8	NUDT8	0,48
REST corepressor 2	RCOR2	0,48
WW domain containing oxidoreductase	WWOX	0,48

UDP-Gal:betaGlcNAc beta 1,4- galactosyltransferase, polypeptide 4	B4GALT4	0,48
WAS protein family, member 3	WASF3	0,48
nuclear receptor coactivator 7	NCOA7	0,48
IMP3, U3 small nucleolar ribonucleoprotein	IMP3	0,48
catalase	CAT	0,48
mediator complex subunit 30	MED30	0,48
leukemia NUP98 fusion partner 1	LNP1	0,48
CD46 molecule, complement regulatory protein	CD46	0,48
fms-related tyrosine kinase 3 ligand	FLT3LG	0,48
ATP binding cassette subfamily A member 1	ABCA1	0,48
protein kinase, cAMP-dependent, regulatory, type I, alpha	PRKAR1A	0,48
ephrin-A3	EFNA3	0,48
N-deacetylase/N-sulfotransferase (heparan glucosaminyl) 1	NDST1	0,48
membrane bound transcription factor peptidase, site 1	MBTPS1	0,48
chromosome 6 open reading frame 48; small nucleolar RNA, C/D box 52; small nucleolar RNA, C/D box 48	C6orf48; SNORD52; SNORD48	0,48
transcription elongation factor A (SII), 2	TCEA2	0,48
nuclear factor I/A	NFIA	0,48
notum pectinacetylesterase homolog (Drosophila)	NOTUM	0,48
TM2 domain containing 3	TM2D3	0,48
tubulin, delta 1	TUBD1	0,48
mitochondrial fission regulator 1	MTFR1	0,48
glyceronephosphate O-acyltransferase	GNPAT	0,48
solute carrier family 9, subfamily B (NHA2, cation proton antiporter 2), member 2	SLC9B2	0,48
ATP/GTP binding protein-like 5	AGBL5	0,48
decapping mRNA 2	DCP2	0,48
family with sequence similarity 20, member B	FAM20B	0,48
transient receptor potential cation channel, subfamily V, member 1	TRPV1	0,48
enoyl-CoA hydratase 1, peroxisomal	ECH1	0,48
SIVA1, apoptosis-inducing factor	SIVA1	0,48
KDEL (Lys-Asp-Glu-Leu) containing 2	KDELC2	0,48
N-acetyltransferase 8-like (GCN5-related, putative)	NAT8L	0,48
Salzman2013 ALT_ACCEPTOR, CDS, coding, INTERNAL, OVCODE, OVEXON best transcript NM_004892; Salzman2013 ANNOTATED, CDS, coding, OVCODE, OVEXON, UTR3 best transcript NM_004892	SEC22B	0,48
MACRO domain containing 1	MACROD1	0,48
phosphatidylinositol glycan anchor biosynthesis class V	PIGV	0,48
peroxisomal biogenesis factor 13	PEX13	0,48

Memczak2013 ANTISENSE, coding, INTERNAL, intronic best transcript NM_181893	UBE2D3	0,48
phosphoglycolate phosphatase	PGP	0,48
zinc finger protein 780A	ZNF780A	0,47
ubiquitin-conjugating enzyme E2, J1	UBE2J1	0,47
CCAAT/enhancer binding protein (C/EBP), gamma	CEBPG	0,47
upstream transcription factor family member 3	USF3	0,47
DTW domain containing 2	DTWD2	0,47
nuclear factor of activated T-cells, cytoplasmic, calcineurin-dependent 3	NFATC3	0,47
vascular endothelial growth factor A	VEGFA	0,47
Memczak2013 ANTISENSE, CDS, coding, INTERNAL best transcript NM_022047	DEF6	0,47
mitochondrial calcium uptake 1	MICU1	0,47
kinesin family member 11	KIF11	0,47
desmocollin 2	DSC2	0,47
COMM domain containing 2	COMMD2	0,47
RAR-related orphan receptor A	RORA	0,47
mannosyl (alpha-1,3-)-glycoprotein beta-1,2-N-acetylglucosaminyltransferase	MGAT1	0,47
testis expressed 2	TEX2	0,47
chromosome 21 open reading frame 33	C21orf33	0,47
Transcript Identified by AceView, Entrez Gene ID(s) 27086; novel transcript, sense intronic to FOXP1	FOXP1; RP11-298C2.1	0,47
sirtuin 4	SIRT4	0,47
adrenergic, beta, receptor kinase 1	ADRBK1	0,47
zinc finger protein, Y-linked	ZFY	0,47
cell division cycle 23	CDC23	0,47
elongator acetyltransferase complex subunit 6	ELP6	0,47
zinc finger protein 614	ZNF614	0,47
ATPase family, AAA domain containing 2B	ATAD2B	0,47
NPR2-like, GATOR1 complex subunit	NPRL2	0,47
trans-golgi network vesicle protein 23 homolog C (S. cerevisiae); CMT1A duplicated region transcript 4; TVP23C-CDRT4 readthrough	TVP23C; CDRT4; TVP23C-CDRT4	0,47
glutaminyl-peptide cyclotransferase-like	QPCTL	0,47
calumenin	CALU	0,47
attractin	ATRN	0,47
collagen, type II, alpha 1	COL2A1	0,47
echinoderm microtubule associated protein like 4	EML4	0,47
PRELI domain containing 2	PRELID2	0,47
MAGE family member D2	MAGED2	0,47
solute carrier family 22 (organic anion transporter), member 7	SLC22A7	0,47
interleukin 7	IL7	0,47

Transcript Identified by AceView, Entrez Gene ID(s) 378465; 65125	WNK1	0,47
N-acetylated alpha-linked acidic dipeptidase-like 1	NAALADL1	0,47
SET domain, bifurcated 2	SETDB2	0,47
retinoblastoma binding protein 4	RBBP4	0,47
solute carrier family 31 (copper transporter), member 1	SLC31A1	0,47
ARP1 actin-related protein 1 homolog B, centractin beta (yeast)	ACTR1B	0,47
family with sequence similarity 222, member A	FAM222A	0,47
ER membrane protein complex subunit 8	EMC8	0,47
baculoviral IAP repeat containing 6	BIRC6	0,47
G kinase anchoring protein 1	GKAP1	0,47
histone cluster 1, H1b	HIST1H1B	0,47
prospero homeobox 1	PROX1	0,47
F-box and leucine-rich repeat protein 21 (gene/pseudogene)	FBXL21	0,47
phospholipid phosphatase 5	PLPP5	0,47
tripartite motif containing 65	TRIM65	0,47
triosephosphate isomerase 1	TPI1	0,47
heat shock protein 70kDa family, member 13	HSPA13	0,47
patatin-like phospholipase domain containing 2	PNPLA2	0,47
chloride channel, voltage-sensitive 6	CLCN6	0,47
sulfite oxidase	SUOX	0,47
mitochondrial ribosomal protein L50	MRPL50	0,47
proline-rich transmembrane protein 1	PRRT1	0,47
guanine nucleotide binding protein (G protein), alpha z polypeptide	GNAZ	0,47
small integral membrane protein 3	SMIM3	0,47
calcium modulating ligand	CAMLG	0,47
ribosomal protein S27-like	RPS27L	0,47
PWWP domain containing 2A	PWWP2A	0,47
FAD-dependent oxidoreductase domain containing 2	FOXRED2	0,47
lethal giant larvae homolog 2 (Drosophila)	LLGL2	0,47
transient receptor potential cation channel, subfamily C, member 1	TRPC1	0,47
sterol regulatory element binding transcription factor 2	SREBF2	0,47
zinc finger protein 362	ZNF362	0,46
Transcript Identified by AceView, Entrez Gene ID(s) 8613	PPAP2B	0,46
protein-O-mannose kinase	POMK	0,46
Transcript Identified by AceView, Entrez Gene ID(s) 5825	ABCD3	0,46
isovaleryl-CoA dehydrogenase	IVD	0,46
chromosome 15 open reading frame 40	C15orf40	0,46
cystatin C	CST3	0,46

leucine rich repeat and coiled-coil centrosomal protein 1	LRRCC1	0,46
LDL receptor related protein 11	LRP11	0,46
ets variant 4	ETV4	0,46
glutaminyl-tRNA synthetase; microRNA 6890	QARS; MIR6890	0,46
apolipoprotein B	APOB	0,46
acyl-CoA synthetase long-chain family member 3	ACSL3	0,46
potassium channel tetramerization domain containing 6	KCTD6	0,46
V-set and immunoglobulin domain containing 10	VSIG10	0,46
cullin-associated and neddylation-dissociated 1	CAND1	0,46
RING1 and YY1 binding protein	RYBP	0,46
lysozyme	LYZ	0,46
ST20-MTHFS readthrough	ST20-MTHFS	0,46
RALBP1 associated Eps domain containing 2	REPS2	0,46
heterogeneous nuclear ribonucleoprotein A0	HNRNPA0	0,46
NME/NM23 nucleoside diphosphate kinase 4	NME4	0,46
galactose-3-O-sulfotransferase 1	GAL3ST1	0,46
ring finger protein 170; microRNA 4469	RNF170; MIR4469	0,46
protein phosphatase 1, catalytic subunit, gamma isozyme	PPP1CC	0,46
abhydrolase domain containing 10	ABHD10	0,46
LSM6 homolog, U6 small nuclear RNA and mRNA degradation associated	LSM6	0,46
hormonally up-regulated Neu-associated kinase	HUNK	0,46
PAN2 poly(A) specific ribonuclease subunit; canopy FGF signaling regulator 2	PAN2; CNPY2	0,46
Ly6/neurotoxin 1	LYNX1	0,46
hypoxia up-regulated 1	HYOU1	0,46
nuclear autoantigenic sperm protein (histone-binding)	NASP	0,46
ligand dependent nuclear receptor corepressor like	LCORL	0,46
transcription factor AP-4 (activating enhancer binding protein 4)	TFAP4	0,46
protease-associated domain containing 1	PRADC1	0,46
pseudouridylate synthase 7-like	PUS7L	0,46
egl-9 family hypoxia-inducible factor 3	EGLN3	0,46
rhotekin	RTKN	0,46
3-phosphoinositide dependent protein kinase 1	PDPK1	0,46
B9 protein domain 1	B9D1	0,46
lipoic acid synthetase	LIAS	0,46
coiled-coil domain containing 85C	CCDC85C	0,46
spalt-like transcription factor 1	SALL1	0,46
formin like 2	FMNL2	0,46
zinc finger protein 512B	ZNF512B	0,46
chloride channel, voltage-sensitive 2	CLCN2	0,46

visinin like 1	VSNL1	0,46
zinc finger, AN1-type domain 2B	ZFAND2B	0,46
PQ loop repeat containing 1	PQLC1	0,46
bleomycin hydrolase	BLMH	0,46
methylenetetrahydrofolate dehydrogenase (NADP+ dependent) 2, methenyltetrahydrofolate cyclohydrolase	MTHFD2	0,46
histone cluster 1, H2al; histone cluster 1, H2bn	HIST1H2AL; HIST1H2BN	0,46
nuclear receptor subfamily 5, group A, member 1	NR5A1	0,46
surfeit 4	SURF4	0,46
geranylgeranyl diphosphate synthase 1	GGPS1	0,46
folliculin interacting protein 2	FNIP2	0,46
tetraspanin 9	TSPAN9	0,46
cell division cycle 14B	CDC14B	0,46
zinc finger, DHHC-type containing 4	ZDHHC4	0,46
NADH dehydrogenase (ubiquinone) 1 beta subcomplex, 11, 17.3kDa	NDUFB11	0,46
sema domain, immunoglobulin domain (Ig), short basic domain, secreted, (semaphorin) 3A	SEMA3A	0,46
proline rich 4 (lacrimal)	PRR4	0,46
2-phosphoxylose phosphatase 1	PXYLP1	0,46
NLR family, apoptosis inhibitory protein	NAIP	0,46
exostosin glycosyltransferase 2	EXT2	0,46
orosomucoid 1	ORM1	0,46
arrestin, beta 1	ARRB1	0,46
CTD small phosphatase like	CTDSPL	0,46
PDZ binding kinase	PBK	0,46
ankyrin repeat domain 17	ANKRD17	0,46
carbohydrate (N-acetylgalactosamine 4-0) sulfotransferase 14	CHST14	0,46
MYC-associated zinc finger protein (purine-binding transcription factor)	MAZ	0,46
5,10-methenyltetrahydrofolate synthetase (5-formyltetrahydrofolate cyclo-ligase)	MTHFS	0,46
leucine rich repeat and Ig domain containing 4	LINGO4	0,46
surfeit 4	SURF4	0,46
family with sequence similarity 189, member B	FAM189B	0,46
dual specificity phosphatase 16	DUSP16	0,46
histone cluster 1, H4e	HIST1H4E	0,46
POP7 homolog, ribonuclease P/MRP subunit	POP7	0,46
RAN binding protein 3-like	RANBP3L	0,46
histone cluster 1, H1c	HIST1H1C	0,45
phosphoglucomutase 3	PGM3	0,45
bone morphogenetic protein receptor type IA	BMPR1A	0,45
carboxypeptidase D	CPD	0,45

hydroxyacylglutathione hydrolase	HAGH	0,45
mannosidase, alpha, class 1A, member 2	MAN1A2	0,45
importin 5	IPO5	0,45
CCR4-NOT transcription complex subunit 6-like	CNOT6L	0,45
coiled-coil domain containing 163, pseudogene	CCDC163P	0,45
EPH receptor A1	EPHA1	0,45
taste receptor, type 2, member 30; taste receptor, type 2, member 43; proline rich 4 (lacrimal)	TAS2R30; TAS2R43; PRR4	0,45
zinc finger, MYND-type containing 19	ZMYND19	0,45
proteasome subunit beta 9	PSMB9	0,45
polypeptide N-acetylgalactosaminyltransferase 1	GALNT1	0,45
DENN/MADD domain containing 5B	DENND5B	0,45
mitogen-activated protein kinase kinase 5	MAP2K5	0,45
LDL receptor related protein associated protein 1	LRPAP1	0,45
aldehyde dehydrogenase 4 family, member A1	ALDH4A1	0,45
acidic nuclear phosphoprotein 32 family member E	ANP32E	0,45
StAR-related lipid transfer domain containing 13	STARD13	0,45
nephronophthisis 4	NPHP4	0,45
coxsackie virus and adenovirus receptor	CXADR	0,45
hypoxia up-regulated 1	HYOU1	0,45
laminin, beta 2 (laminin S)	LAMB2	0,45
Transcript Identified by AceView, Entrez Gene ID(s) 23126	POGZ	0,45
vav 2 guanine nucleotide exchange factor	VAV2	0,45
mannosyl (alpha-1,6-)-glycoprotein beta-1,2-N-acetylglucosaminyltransferase	MGAT2	0,45
ankyrin 2, neuronal	ANK2	0,45
ATP binding cassette subfamily D member 4	ABCD4	0,45
akirin 1	AKIRIN1	0,45
CTD small phosphatase 2	CTDSP2	0,45
thioredoxin domain containing 11	TXNDC11	0,45
zinc finger protein, X-linked	ZFX	0,45
ATPase, Na+/K+ transporting, beta 1 polypeptide	ATP1B1	0,45
C1q and tumor necrosis factor related protein 6	C1QTNF6	0,45
Transcript Identified by AceView, Entrez Gene ID(s) 10926	DBF4	0,45
protein kinase domain containing, cytoplasmic	PKDCC	0,45
ADP-ribosylation factor like GTPase 1	ARL1	0,45
UDP-Gal:betaGlcNAc beta 1,4- galactosyltransferase, polypeptide 2	B4GALT2	0,45
mitochondrial ribosomal protein L48	MRPL48	0,45
Memczak2013 ALT_ACCEPTOR, ALT_DONOR, coding, INTERNAL, intronic best transcript NM_002015	FOXO1	0,45
integral membrane protein 2B	ITM2B	0,45
RPARP antisense RNA 1	RPARP-AS1	0,45

G-protein signaling modulator 2	GPSM2	0,45
hypoxia up-regulated 1	HYOU1	0,45
zinc finger homeobox 3	ZFHX3	0,44
PDZ domain containing 1	PDZK1	0,44
peptidylprolyl isomerase C (cyclophilin C)	PPIC	0,44
zinc fingers and homeoboxes 2	ZHX2	0,44
DDB1 and CUL4 associated factor 12	DCAF12	0,44
ankyrin repeat domain 16	ANKRD16	0,44
COBW domain containing 6; COBW domain containing 5; COBW domain containing 7	CBWD6; CBWD5; CBWD7	0,44
solute carrier family 22, member 23	SLC22A23	0,44
leucine zipper-EF-hand containing transmembrane protein 1	LETM1	0,44
acetyl-CoA carboxylase beta	ACACB	0,44
RWD domain containing 2A	RWDD2A	0,44
ubiquitin specific peptidase 7 (herpes virus-associated)	USP7	0,44
ribosomal protein L7a	RPL7A	0,44
CMT1A duplicated region transcript 1	CDRT1	0,44
transmembrane protein 198	TMEM198	0,44
WW domain containing transcription regulator 1	WWTR1	0,44
mucin 15, cell surface associated	MUC15	0,44
EH domain containing 3	EHD3	0,44
chemokine (C-C motif) ligand 16	CCL16	0,44
CCR4-NOT transcription complex subunit 11	CNOT11	0,44
tRNA 5-methylaminomethyl-2-thiouridylate methyltransferase	TRMU	0,44
spire-type actin nucleation factor 2	SPIRE2	0,44
kelch domain containing 10	KLHDC10	0,44
zinc finger and BTB domain containing 5	ZBTB5	0,44
dihydropyrimidinase-like 2	DPYSL2	0,44
serpin peptidase inhibitor, clade A (alpha-1 antiproteinase, antitrypsin), member 12	SERPINA12	0,44
apolipoprotein H (beta-2-glycoprotein I)	APOH	0,44
testis expressed 264	TEX264	0,44
B-cell CLL/lymphoma 6	BCL6	0,44
cathepsin C	CTSC	0,44
solute carrier family 2 (facilitated glucose transporter), member 9	SLC2A9	0,44
arginine vasopressin-induced 1	AVPI1	0,44
v-rel avian reticuloendotheliosis viral oncogene homolog	REL	0,44
PDS5 cohesin associated factor B	PDS5B	0,44
centrin 3	CETN3	0,44
tweety family member 1	TTYH1	0,44
histone deacetylase 6	HDAC6	0,44

abhydrolase domain containing 18	ABHD18	0,44
mannosyl-oligosaccharide glucosidase	MOGS	0,44
grancalcin, EF-hand calcium binding protein	GCA	0,44
unkempt family zinc finger	UNK	0,44
thyrotrophic embryonic factor	TEF	0,44
THO complex 6	THOC6	0,44
aminoacylase 3	ACY3	0,44
heat shock transcription factor 2 binding protein	HSF2BP	0,44
zinc finger protein 660	ZNF660	0,44
Memczak2013 ALT_ACCEPTOR, ALT_DONOR, coding, INTERNAL, intronic best transcript NM_001008895	CUL4A	0,44
vacuolar protein sorting 51 homolog (S. cerevisiae)	VPS51	0,44
chromosome 19 open reading frame 66	C19orf66	0,44
kinesin family member 18B	KIF18B	0,44
zinc finger protein 514	ZNF514	0,44
myotubularin 1	MTM1	0,44
UBX domain protein 7	UBXN7	0,44
forkhead box O4	FOXO4	0,44
carboxypeptidase Q	CPQ	0,44
Transcript Identified by AceView, Entrez Gene ID(s) 27332	ZNF638	0,44
Memczak2013 ANTISENSE, CDS, coding, downstream_end, upstream_start, UTR3, UTR5 best transcript NM_004065; CDR1 antisense RNA; CDR1 antisense RNA [Source:HGNC Symbol;Acc:HGNC:48926]	CDR1; CDR1-AS	0,44
serine/arginine-rich splicing factor 4	SRSF4	0,44
arylsulfatase E (chondrodysplasia punctata 1)	ARSE	0,44
carnitine palmitoyltransferase 2	CPT2	0,44
cytoskeleton associated protein 2	CKAP2	0,43
inositol-3-phosphate synthase 1	ISYNA1	0,43
fibronectin type III domain containing 3B	FNDC3B	0,43
SEC63 homolog, protein translocation regulator	SEC63	0,43
acyl-CoA oxidase 1, palmitoyl	ACOX1	0,43
carnosine N-methyltransferase 1	CARNMT1	0,43
transmembrane protease, serine 2	TMPRSS2	0,43
pleckstrin homology domain containing, family A (phosphoinositide binding specific) member 2	PLEKHA2	0,43
YEATS domain containing 4	YEATS4	0,43
caspase 8 associated protein 2	CASP8AP2	0,43
ATPase family, AAA domain containing 2	ATAD2	0,43
dual specificity phosphatase 6	DUSP6	0,43
transmembrane protein 263	TMEM263	0,43
cytochrome P450, family 4, subfamily F, polypeptide 12	CYP4F12	0,43
contactin associated protein-like 3B	CNTNAP3B	0,43
thyroid hormone receptor, beta	THRB	0,43

solute carrier family 43 (amino acid system L transporter), member 1	SLC43A1	0,43
replication factor C subunit 1	RFC1	0,43
nuclear receptor subfamily 2, group F, member 1	NR2F1	0,43
anaphase promoting complex subunit 5	ANAPC5	0,43
solute carrier family 17 (vesicular nucleotide transporter), member 9	SLC17A9	0,43
transforming growth factor, beta receptor 1	TGFBR1	0,43
KIAA1841	KIAA1841	0,43
transmembrane protein 98	TMEM98	0,43
Memczak2013 ALT_ACCEPTOR, ALT_DONOR, coding, INTERNAL, intronic best transcript NM_024766	CAMKMT	0,43
miR-17-92 cluster host gene; microRNA 17; microRNA 18a; microRNA 19a; microRNA 19b-1; microRNA 20a; microRNA 92a-1	MIR17HG; MIR17; MIR18A; MIR19A; MIR19B1; MIR20A; MIR92A1	0,43
family with sequence similarity 58, member A	FAM58A	0,43
zinc finger protein 318	ZNF318	0,43
mitochondrial pyruvate carrier 2	MPC2	0,43
choline dehydrogenase	CHDH	0,43
solute carrier family 37 (glucose-6-phosphate transporter), member 4 [Source:HGNC Symbol;Acc:HGNC:4061]	SLC37A4	0,43
germ cell-less, spermatogenesis associated 1	GMCL1	0,43
ADAM metallopeptidase with thrombospondin type 1 motif 6	ADAMTS6	0,43
phosphate cytidylyltransferase 1, choline, beta	PCYT1B	0,43
acetyl-CoA acyltransferase 1	ACAA1	0,43
round spermatid basic protein 1-like	RSBN1L	0,43
ventral anterior homeobox 2	VAX2	0,43
Opa interacting protein 5	OIP5	0,43
transmembrane protein 129, E3 ubiquitin protein ligase	TMEM129	0,43
asparaginyl-tRNA synthetase 2, mitochondrial (putative)	NARS2	0,43
nucleoporin 205kDa	NUP205	0,43
plasmolipin	PLLP	0,43
solute carrier family 37 (glucose-6-phosphate transporter), member 4	SLC37A4	0,43
cyclin B1	CCNB1	0,43
cellular repressor of E1A-stimulated genes 1	CREG1	0,43
Hermansky-Pudlak syndrome 4	HPS4	0,43
RAB43, member RAS oncogene family	RAB43	0,43

novel transcript; Transcript Identified by AceView, Entrez Gene ID(s) 55737	RP11-93O14.2; VPS35	0,43
DnaJ (Hsp40) homolog, subfamily C, member 12	DNAJC12	0,43
peroxiredoxin 4	PRDX4	0,43
coiled-coil domain containing 183; RAB, member RAS oncogene family-like 6	CCDC183; RABL6	0,43
gamma-glutamyl carboxylase	GGCX	0,43
single stranded DNA binding protein 4	SSBP4	0,43
runt-related transcription factor 1	RUNX1	0,43
proline rich Gla (G-carboxyglutamic acid) 4 (transmembrane)	PRRG4	0,42
IgA-inducing protein	IGIP	0,42
sorting nexin 4	SNX4	0,42
armadillo repeat containing 5	ARMC5	0,42
NLR family, apoptosis inhibitory protein	NAIP	0,42
multivesicular body subunit 12A; BST2 interferon stimulated positive regulator (non-protein coding)	MVB12A; BISPR	0,42
adrenoceptor alpha 2C	ADRA2C	0,42
angel homolog 1 (Drosophila)	ANGEL1	0,42
pyroglutamyl-peptidase I	PGPEP1	0,42
CNKSR family member 3	CNKSR3	0,42
protein kinase, AMP-activated, beta 2 non-catalytic subunit	PRKAB2	0,42
histone cluster 1, H1e	HIST1H1E	0,42
nuclear factor of kappa light polypeptide gene enhancer in B-cells inhibitor, alpha	NFKBIA	0,42
phosphodiesterase 3B, cGMP-inhibited	PDE3B	0,42
chemokine (C-C motif) receptor 6	CCR6	0,42
quinolinate phosphoribosyltransferase	QPRT	0,42
carbohydrate (N-acetylgalactosamine 4-0) sulfotransferase 9	CHST9	0,42
cAMP responsive element binding protein 3-like 2	CREB3L2	0,42
ATPase, Ca++ transporting, plasma membrane 2	ATP2B2	0,42
chromosome 21 open reading frame 33	C21orf33	0,42
adenosine monophosphate deaminase 2	AMPD2	0,42
phytanoyl-CoA 2-hydroxylase interacting protein-like	PHYHIPL	0,42
centriole, cilia and spindle-associated protein	CCSAP	0,42
twinfilin actin binding protein 2	TWF2	0,42
secreted and transmembrane 1	SECTM1	0,42
pantothenate kinase 3	PANK3	0,42
ST3 beta-galactoside alpha-2,3-sialyltransferase 6	ST3GAL6	0,42
interleukin 6 signal transducer	IL6ST	0,42
apolipoprotein A-V	APOA5	0,42
leucine-rich repeat containing G protein-coupled receptor 4	LGR4	0,42

Transcript Identified by AceView, Entrez Gene ID(s) 58155	PTBP2	0,42
kelch domain containing 3	KLHDC3	0,42
uroplakin 3A	UPK3A	0,42
synaptojanin 2	SYNJ2	0,42
mannosidase, endo-alpha	MANEA	0,42
MCF.2 cell line derived transforming sequence like	MCF2L	0,42
mitogen-activated protein kinase kinase kinase 8	MAP3K8	0,42
NIMA-related kinase 6	NEK6	0,42
SFT2 domain containing 3	SFT2D3	0,42
protein C (inactivator of coagulation factors Va and VIIIa)	PROC	0,42
kinesin family member C1	KIFC1	0,42
ribosomal protein L27a; small nucleolar RNA, H/ACA box 3A; small nucleolar RNA, H/ACA box 3B	RPL27A; SNORA3A; SNORA3B	0,42
dehydrogenase/reductase (SDR family) member 11	DHRS11	0,42
SRY box 12	SOX12	0,42
topoisomerase (DNA) II beta; microRNA 4442	TOP2B; MIR4442	0,42
pituitary tumor-transforming 2	PTTG2	0,42
tRNA 5-methylaminomethyl-2-thiouridylate methyltransferase	TRMU	0,42
TRPM8 channel-associated factor 1	TCAF1	0,42
LSM11, U7 small nuclear RNA associated	LSM11	0,42
leucine rich repeat containing 75B	LRRC75B	0,41
tight junction protein 3	TJP3	0,41
histone cluster 1, H4k	HIST1H4K	0,41
WD repeat and FYVE domain containing 2	WDFY2	0,41
TMED7-TICAM2 readthrough; toll-like receptor adaptor molecule 2; transmembrane p24 trafficking protein 7	TMED7-TICAM2; TICAM2; TMED7	0,41
histone cluster 1, H2bg	HIST1H2BG	0,41
mannosyl (alpha-1,6-)-glycoprotein beta-1,6-N-acetyl-glucosaminyltransferase	MGAT5	0,41
isocitrate dehydrogenase 2 (NADP+), mitochondrial	IDH2	0,41
inhibin beta E	INHBE	0,41
calmodulin regulated spectrin-associated protein family, member 3	CAMSAP3	0,41
sex comb on midleg homolog 1 (Drosophila)	SCMH1	0,41
StAR-related lipid transfer domain containing 10	STARD10	0,41
transmembrane protein 216	TMEM216	0,41
Rho guanine nucleotide exchange factor (GEF) 40	ARHGEF40	0,41
PHD finger protein 8	PHF8	0,41
N-acylethanolamine acid amidase	NAAA	0,41
chromosome 1 open reading frame 233	C1orf233	0,41
phosphoserine phosphatase	PSPH	0,41

receptor accessory protein 6	REEP6	0,41
electron-transfer-flavoprotein, beta polypeptide	ETFB	0,41
H2A histone family, member X	H2AFX	0,41
family with sequence similarity 19 (chemokine (C-C motif)-like), member A4	FAM19A4	0,41
growth arrest-specific 2 like 3	GAS2L3	0,41
retinol binding protein 4, plasma	RBP4	0,41
calcium channel flower domain containing 1	CACFD1	0,41
glycine cleavage system protein H (aminomethyl carrier)	GCSH	0,41
minichromosome maintenance complex component 6	MCM6	0,41
tetratricopeptide repeat domain 39C	TTC39C	0,41
solute carrier family 25 (mitochondrial carrier; ornithine transporter) member 15; microRNA 621	SLC25A15; MIR621	0,41
hypoxia up-regulated 1	HYOU1	0,41
FYVE, RhoGEF and PH domain containing 4	FGD4	0,41
solute carrier family 18, subfamily B, member 1	SLC18B1	0,41
eukaryotic translation elongation factor 1 beta 2; small nucleolar RNA, H/ACA box 41	EEF1B2; SNORA41	0,41
fumarate hydratase	FH	0,41
sestrin 1	SESN1	0,41
ring finger protein 122	RNF122	0,41
cell division cycle 25A	CDC25A	0,41
surfeit 4	SURF4	0,41
pyruvate dehydrogenase kinase, isozyme 3	PDK3	0,41
DnaJ (Hsp40) homolog, subfamily C, member 19	DNAJC19	0,41
mitochondrial ribosomal protein L14	MRPL14	0,41
Rab interacting lysosomal protein-like 2	RILPL2	0,41
solute carrier family 35 (UDP-GlcA/UDP-GalNAc transporter), member D1	SLC35D1	0,41
NEDD4 binding protein 2-like 1	N4BP2L1	0,41
mannosidase, alpha, class 1A, member 1	MAN1A1	0,41
ADP-ribosylation factor like GTPase 5A	ARL5A	0,41
transcription factor Dp-2 (E2F dimerization partner 2)	TFDP2	0,41
amylo-alpha-1, 6-glucosidase, 4-alpha-glucanotransferase	AGL	0,41
endoplasmic reticulum lectin 1	ERLEC1	0,41
TBC1 domain family, member 16	TBC1D16	0,41
absent in melanoma 1	AIM1	0,41
zinc finger protein 652	ZNF652	0,41
prolylcarboxypeptidase	PRCP	0,41
histone cluster 1, H2bf	HIST1H2BF	0,41
solute carrier family 6 (neurotransmitter transporter), member 8	SLC6A8	0,41
methylsterol monooxygenase 1	MSMO1	0,41

insulin like growth factor binding protein 1	IGFBP1	0,41
zinc finger protein 740	ZNF740	0,41
FAM3C pseudogene	FAM3C2	0,41
myotubularin related protein 4	MTMR4	0,41
F-box protein 5	FBXO5	0,41
cyclin-dependent kinase 6	CDK6	0,41
tripartite motif containing 16	TRIM16	0,41
matrix metallopeptidase 15 (membrane-inserted)	MMP15	0,41
BRCA1 interacting protein C-terminal helicase 1	BRIP1	0,40
forkhead box N3	FOXN3	0,40
rhotekin 2	RTKN2	0,40
WW domain containing E3 ubiquitin protein ligase 1	WWP1	0,40
hypoxia up-regulated 1	HYOU1	0,40
ORMDL sphingolipid biosynthesis regulator 3	ORMDL3	0,40
ankyrin repeat and SOCS box containing 9	ASB9	0,40
ribonuclease T2	RNASET2	0,40
glycerophosphodiester phosphodiesterase domain containing 1	GDPD1	0,40
primase, DNA, polypeptide 1 (49kDa)	PRIM1	0,40
homogentisate 1,2-dioxygenase	HGD	0,40
chromosome 17 open reading frame 96	C17orf96	0,40
valyl-tRNA synthetase 2, mitochondrial	VARS2	0,40
acyl-CoA synthetase medium-chain family member 3	ACSM3	0,40
CKLF-like MARVEL transmembrane domain containing 8	CMTM8	0,40
apoptosis-inducing factor, mitochondrion-associated, 1	AIFM1	0,40
tetratricopeptide repeat domain 32	TTC32	0,40
phosphodiesterase 3A, cGMP-inhibited	PDE3A	0,40
USP6 N-terminal like	USP6NL	0,40
exostosin-like glycosyltransferase 2	EXTL2	0,40
cyclin B2	CCNB2	0,40
phosphoglycerate dehydrogenase	PHGDH	0,40
nucleolar and spindle associated protein 1	NUSAP1	0,40
metastasis associated 1	MTA1	0,40
procollagen-lysine, 2-oxoglutarate 5-dioxygenase 1	PLOD1	0,40
glutamine--fructose-6-phosphate transaminase 1	GFPT1	0,40
cyclin-dependent kinase 19	CDK19	0,40
nuclear receptor subfamily 5, group A, member 2	NR5A2	0,40
centromere protein V	CENPV	0,40
jade family PHD finger 1	JADE1	0,40
TBC1 domain family, member 8B (with GRAM domain)	TBC1D8B	0,40
spastic paraplegia 20 (Troyer syndrome)	SPG20	0,40
monoacylglycerol O-acyltransferase 2	MOGAT2	0,40
ADP-ribosylation factor like GTPase 3	ARL3	0,40
leucine rich repeat containing 8 family, member D	LRRC8D	0,40
ectonucleoside triphosphate diphosphohydrolase 5	ENTPD5	0,40

phosphofructokinase, liver	PFKL	0,40
cyclin-dependent kinase inhibitor 1B (p27, Kip1)	CDKN1B	0,40
zinc finger, DHHC-type containing 17	ZDHHC17	0,40
chromosome 8 open reading frame 37	C8orf37	0,40
family with sequence similarity 117, member B	FAM117B	0,40
monoacylglycerol O-acyltransferase 1	MOGAT1	0,40
mutS homolog 6	MSH6	0,40
ELMO/CED-12 domain containing 2	ELMOD2	0,39
hypoxia up-regulated 1	HYOU1	0,39
transmembrane protein 181	TMEM181	0,39
KDEL (Lys-Asp-Glu-Leu) endoplasmic reticulum protein retention receptor 2	KDELR2	0,39
spalt-like transcription factor 2	SALL2	0,39
ubiquitin specific peptidase 2	USP2	0,39
solute carrier family 38, member 5	SLC38A5	0,39
5-nucleotidase domain containing 2	NT5DC2	0,39
Jeck2013 ALT_ACCEPTOR, ALT_DONOR, coding, INTERNAL, intronic best transcript NM_000314; novel transcript, sense intronic to PTEN	RP11-380G5.2; PTEN	0,39
galactose mutarotase (aldose 1-epimerase)	GALM	0,39
hypoxia up-regulated 1	HYOU1	0,39
cell growth regulator with EF-hand domain 1	CGREF1	0,39
cyclin A2	CCNA2	0,39
glycine cleavage system protein H (aminomethyl carrier)	GCSH	0,39
transmembrane protein 86B	TMEM86B	0,39
	LRIG1	0,39
nucleobindin 2	NUCB2	0,39
cytochrome P450, family 19, subfamily A, polypeptide 1	CYP19A1	0,39
XIAP associated factor 1	XAF1	0,39
cyclin-dependent kinase inhibitor 3	CDKN3	0,39
stress-associated endoplasmic reticulum protein 1	SERP1	0,39
leucine zipper, putative tumor suppressor 2	LZTS2	0,39
adhesion G protein-coupled receptor G6	ADGRG6	0,39
mitogen-activated protein kinase kinase kinase 1, E3 ubiquitin protein ligase	MAP3K1	0,39
coproporphyrinogen oxidase	CPOX	0,39
ER membrane protein complex subunit 9	EMC9	0,39
adhesion G protein-coupled receptor A3	ADGRA3	0,39
calcium regulated heat stable protein 1	CARHSP1	0,39
hydroxysteroid (17-beta) dehydrogenase 12	HSD17B12	0,39
acyl-CoA synthetase medium-chain family member 2A	ACSM2A	0,39
mesoderm specific transcript	MEST	0,39
proteasome activator subunit 2; microRNA 7703	PSME2; MIR7703	0,39
pantothenate kinase 2	PANK2	0,39

ubiquitin domain containing 2	UBTD2	0,39
zinc finger protein 792	ZNF792	0,39
G protein-coupled receptor 157	GPR157	0,39
hypoxia up-regulated 1	HYOU1	0,39
sortilin 1	SORT1	0,39
phosphatidylcholine transfer protein	PCTP	0,39
chondroitin polymerizing factor	CHPF	0,39
glycerol kinase	GK	0,39
lectin, galactoside-binding, soluble, 2	LGALS2	0,39
Jeck2013 ALT_ACCEPTOR, ALT_DONOR, coding, INTERNAL, intronic best transcript NM_005766	FARP1	0,39
coiled-coil domain containing 138	CCDC138	0,39
hypoxia up-regulated 1	HYOU1	0,39
zinc finger CCCH-type, antiviral 1-like	ZC3HAV1L	0,39
DIS3 like exosome 3-5 exoribonuclease	DIS3L	0,39
mitochondrial ribosomal protein L16	MRPL16	0,38
NudC domain containing 2	NUDCD2	0,38
ribosomal modification protein rimK-like family member A	RIMKLA	0,38
glutathione S-transferase alpha 4	GSTA4	0,38
ADP-ribosylation factor like GTPase 4D	ARL4D	0,38
NHL repeat containing 3	NHLRC3	0,38
homeobox D4	HOXD4	0,38
Janus kinase 3	JAK3	0,38
transient receptor potential cation channel, subfamily V, member 4	TRPV4	0,38
serpin peptidase inhibitor, clade G (C1 inhibitor), member 1	SERPING1	0,38
solute carrier family 44 (choline transporter), member 1	SLC44A1	0,38
Yip1 interacting factor homolog A (S. cerevisiae)	YIF1A	0,38
SEC24 homolog D, COPII coat complex component	SEC24D	0,38
natural killer cell cytotoxicity receptor 3 ligand 1	NCR3LG1	0,38
ankyrin repeat and SOCS box containing 13	ASB13	0,38
splicing factor 3b, subunit 1, 155kDa	SF3B1	0,38
family with sequence similarity 136, member A	FAM136A	0,38
cytochrome P450, family 3, subfamily A, polypeptide 43	CYP3A43	0,38
proline/serine-rich coiled-coil 1	PSRC1	0,38
mannosidase, alpha, class 1B, member 1	MAN1B1	0,38
CAS1 domain containing 1	CASD1	0,38
zinc finger protein 346	ZNF346	0,38
zinc finger protein 358	ZNF358	0,38
proteasome 26S subunit, non-ATPase 10	PSMD10	0,38
hyaluronoglucosaminidase 1	HYAL1	0,38
calcineurin binding protein 1	CABIN1	0,38
tetratricopeptide repeat domain 6	TTC6	0,38

polo-like kinase 1	PLK1	0,38
family with sequence similarity 13, member A	FAM13A	0,38
transmembrane protein 143	TMEM143	0,38
thioredoxin domain containing 5 (endoplasmic reticulum)	TXNDC5	0,38
basic, immunoglobulin-like variable motif containing	BIVM	0,38
hydroxysteroid dehydrogenase like 2	HSDL2	0,37
Sec61 translocon beta subunit	SEC61B	0,37
sideroflexin 2	SFXN2	0,37
family with sequence similarity 35, member A	FAM35A	0,37
NAD(P) dependent steroid dehydrogenase-like	NSDHL	0,37
protein phosphatase 6, regulatory subunit 2	PPP6R2	0,37
chromobox homolog 4	CBX4	0,37
histone cluster 2, H2ab	HIST2H2AB	0,37
KIAA0101; casein kinase 1, gamma 1	KIAA0101; CSNK1G1	0,37
protein phosphatase 4, regulatory subunit 2	PPP4R2	0,37
LDL receptor related protein 4	LRP4	0,37
phospholipase D1, phosphatidylcholine-specific	PLD1	0,37
LDL receptor related protein 6	LRP6	0,37
epilepsy, progressive myoclonus type 2A, Lafora disease (laforin)	EPM2A	0,37
pleckstrin homology domain containing, family G (with RhoGef domain) member 6	PLEKHG6	0,37
maestro heat-like repeat family member 8	MROH8	0,37
integrator complex subunit 10	INTS10	0,37
sema domain, immunoglobulin domain (Ig), transmembrane domain (TM) and short cytoplasmic domain, (semaphorin) 4C	SEMA4C	0,37
FK506 binding protein 7	FKBP7	0,37
FERM domain containing 3	FRMD3	0,37
F-box protein 17	FBXO17	0,37
centromere protein F	CENPF	0,37
histone cluster 1, H2ag	HIST1H2AG	0,37
sepiapterin reductase (7,8-dihydrobiopterin:NADP+ oxidoreductase)	SPR	0,37
calmegin	CLGN	0,37
dicer 1, ribonuclease type III	DICER1	0,37
component of oligomeric golgi complex 6	COG6	0,37
solute carrier family 25, member 43	SLC25A43	0,37
mitochondrial amidoxime reducing component 2	MARC2	0,37
platelet-derived growth factor receptor, beta polypeptide	PDGFRB	0,37
noncompact myelin associated protein	NCMAP	0,37
signal peptide peptidase like 2B	SPPL2B	0,37

cytochrome P450, family 4, subfamily V, polypeptide 2	CYP4V2	0,37
follistatin	FST	0,37
Pim-2 proto-oncogene, serine/threonine kinase	PIM2	0,37
tetraspanin 33	TSPAN33	0,37
UPF1 regulator of nonsense transcripts homolog (yeast)	UPF1	0,37
E2F transcription factor 5, p130-binding	E2F5	0,37
DENN/MADD domain containing 1B	DENND1B	0,37
ankyrin repeat and sterile alpha motif domain containing 4B	ANKS4B	0,37
polypeptide N-acetylgalactosaminyltransferase 13	GALNT13	0,37
carbohydrate (N-acetylgalactosamine 4-sulfate 6-O) sulfotransferase 15	CHST15	0,37
carbonic anhydrase XIII	CA13	0,37
Cbp/p300-interacting transactivator, with Glu/Asp rich carboxy-terminal domain, 2	CITED2	0,37
hCG2028352-like; novel transcript	LOC729970; RP4-639F20.1	0,37
transmembrane protein 243, mitochondrial	TMEM243	0,37
cystathionine-beta-synthase	CBS	0,37
NHS-like 1	NHSL1	0,37
triple QxxK/R motif containing	TRIQK	0,37
gelsolin	GSN	0,37
minichromosome maintenance complex component 3	MCM3	0,37
tetratricopeptide repeat domain 3	TTC3	0,37
Werner helicase interacting protein 1	WRNIP1	0,37
ankyrin repeat domain 46	ANKRD46	0,37
succinate dehydrogenase complex assembly factor 3	SDHAF3	0,36
immunoglobulin superfamily, member 23	IGSF23	0,36
neuronal guanine nucleotide exchange factor	NGEF	0,36
acyl-CoA synthetase short-chain family member 3	ACSS3	0,36
TNF receptor-associated factor 4	TRAF4	0,36
glutamate-ammonia ligase	GLUL	0,36
mannosidase, alpha, class 2A, member 1	MAN2A1	0,36
interleukin 27 receptor, alpha	IL27RA	0,36
high mobility group nucleosomal binding domain 3	HMGN3	0,36
kelch domain containing 2	KLHDC2	0,36
ALG14, UDP-N-acetylglucosaminyltransferase subunit	ALG14	0,36
diacylglycerol O-acyltransferase 1; microRNA 6848	DGAT1; MIR6848	0,36
GTF2I repeat domain containing 2; GTF2I repeat domain containing 2B	GTF2IRD2; GTF2IRD2B	0,36
transmembrane p24 trafficking protein 9	TMED9	0,36
histone cluster 1, H3f	HIST1H3F	0,36
fat storage-inducing transmembrane protein 1	FITM1	0,36
hexokinase 2	HK2	0,36

serpin peptidase inhibitor, clade A (alpha-1 antiproteinase, antitrypsin), member 1	SERPINA1	0,36
zinc finger protein 275	ZNF275	0,36
Rho guanine nucleotide exchange factor 10 like	ARHGEF10L	0,36
serine palmitoyltransferase, long chain base subunit 3	SPTLC3	0,36
solute carrier family 38, member 3	SLC38A3	0,36
mitochondrial fission regulator 1-like	MTFR1L	0,36
DEP domain containing 7	DEPDC7	0,36
cysteine dioxygenase type 1	CDO1	0,36
histone cluster 1, H3b	HIST1H3B	0,36
DnaJ (Hsp40) homolog, subfamily C, member 3	DNAJC3	0,36
yippee like 3	YPEL3	0,36
family with sequence similarity 19 (chemokine (C-C motif)-like), member A5	FAM19A5	0,36
solute carrier family 25 (mitochondrial carrier; phosphate carrier), member 23	SLC25A23	0,36
small nucleolar RNA, H/ACA box 17A; small nucleolar RNA, H/ACA box 17B; small nucleolar RNA host gene 7	SNORA17A; SNORA17B; SNHG7	0,36
methionine adenosyltransferase I, alpha	MAT1A	0,36
tripartite motif containing 71, E3 ubiquitin protein ligase	TRIM71	0,36
Jeck2013 ALT_ACCEPTOR, ALT_DONOR, coding, INTERNAL, intronic, OVERLAPTX, OVEXON best transcript NM_000942	PPIB	0,36
histamine N-methyltransferase	HNMT	0,36
DEAH (Asp-Glu-Ala-His) box polypeptide 33	DHX33	0,36
nuclear factor of activated T-cells, cytoplasmic, calcineurin-dependent 2	NFATC2	0,36
ethanolamine kinase 1	ETNK1	0,36
mercaptopyruvate sulfurtransferase	MPST	0,36
lin-7 homolog A (C. elegans)	LIN7A	0,36
progesterone receptor membrane component 2	PGRMC2	0,36
ATPase, class VI, type 11A	ATP11A	0,36
Transcript Identified by AceView, Entrez Gene ID(s) 8395	PIP5K1B	0,36
cyclin B1 interacting protein 1, E3 ubiquitin protein ligase; small nucleolar RNA, C/D box 126	CCNB1IP1; SNORD126	0,36
COX11 cytochrome c oxidase copper chaperone	COX11	0,36
dolichyldiphosphatase 1	DOLPP1	0,36
family with sequence similarity 156, member A	FAM156A	0,36
ectodermal-neural cortex 1 (with BTB domain)	ENC1	0,36
cystathionine-beta-synthase	CBS	0,35
nuclear factor of kappa light polypeptide gene enhancer in B-cells 1	NFKB1	0,35

Sad1 and UNC84 domain containing 2	SUN2	0,35
ubiquitin-like modifier activating enzyme 7; microRNA 5193	UBA7; MIR5193	0,35
SWI/SNF related, matrix associated, actin dependent regulator of chromatin, subfamily a, member 1	SMARCA1	0,35
serpin peptidase inhibitor, clade F (alpha-2 antiplasmin, pigment epithelium derived factor), member 1	SERPINF1	0,35
protein O-glucosyltransferase 1	POGLUT1	0,35
chromosome 12 open reading frame 57; RNA, U7 small nuclear 1	C12orf57; RNU7-1	0,35
cytochrome P450, family 2, subfamily W, polypeptide 1	CYP2W1	0,35
transmembrane protein 39A	TMEM39A	0,35
high mobility group AT-hook 2	HMGA2	0,35
regulatory factor X, 5 (influences HLA class II expression)	RFX5	0,35
heat-responsive protein 12	HRSP12	0,35
Transcript Identified by AceView, Entrez Gene ID(s) 2146	EZH2	0,35
NGFI-A binding protein 1	NAB1	0,35
peroxisomal trans-2-enoyl-CoA reductase	PECR	0,35
cyclin-dependent kinase inhibitor 2C (p18, inhibits CDK4)	CDKN2C	0,35
prolyl 4-hydroxylase, alpha polypeptide I	P4HA1	0,35
MAGE family member F1	MAGEF1	0,35
kazrin, periplakin interacting protein	KAZN	0,35
TBC1 domain family, member 4	TBC1D4	0,35
Fc fragment of IgG binding protein	FCGBP	0,35
inhibitor of DNA binding 1, dominant negative helix-loop-helix protein	ID1	0,35
solute carrier family 22, member 31	SLC22A31	0,35
methyltransferase like 21A	METTL21A	0,35
LDL receptor related protein 5	LRP5	0,35
PRELI domain containing 3A	PRELID3A	0,35
taste receptor, type 2, member 14	TAS2R14	0,35
hypoxia up-regulated 1	HYOU1	0,35
ATPase, Ca++ transporting, plasma membrane 1	ATP2B1	0,35
contactin associated protein-like 3 pseudogene 2; contactin associated protein-like 3	CNTNAP3P2; CNTNAP3	0,35
golgi integral membrane protein 4	GOLIM4	0,35
IQ motif and Sec7 domain 1	IQSEC1	0,35
dopey family member 1	DOPEY1	0,35
protogenin	PRTG	0,35
3-phosphoadenosine 5-phosphosulfate synthase 1	PAPSS1	0,35
centromere protein A	CENPA	0,35
Rho-related BTB domain containing 1	RHOBTB1	0,35
myotubularin related protein 12	MTMR12	0,35

chemokine (C-X-C motif) ligand 16	CXCL16	0,35
solute carrier family 11 (proton-coupled divalent metal ion transporter), member 2	SLC11A2	0,35
sorting nexin 1	SNX1	0,35
histone cluster 1, H2ae	HIST1H2AE	0,35
solute carrier family 6 (neurotransmitter transporter), member 11	SLC6A11	0,35
zinc finger and SCAN domain containing 5A	ZSCAN5A	0,34
small integral membrane protein 24	SMIM24	0,34
sedoheptulokinase; transient receptor potential cation channel, subfamily V, member 1	SHPK; TRPV1	0,34
UDP-N-acetylglucosamine pyrophosphorylase 1	UAP1	0,34
fatty acid desaturase 1; microRNA 1908	FADS1; MIR1908	0,34
Rho guanine nucleotide exchange factor 25	ARHGEF25	0,34
reticulocalbin 2, EF-hand calcium binding domain	RCN2	0,34
complement component 1, r subcomponent-like	C1RL	0,34
EF-hand calcium binding domain 11	EFCAB11	0,34
tripartite motif containing 50	TRIM50	0,34
Ras association (RalGDS/AF-6) domain family member 4	RASSF4	0,34
chromosome 14 open reading frame 1	C14orf1	0,34
nuclear receptor subfamily 2, group F, member 6	NR2F6	0,34
zinc finger protein 107; microRNA 6839	ZNF107; MIR6839	0,34
threonyl-tRNA synthetase-like 2	TARSL2	0,34
beta-1,4-N-acetyl-galactosaminyl transferase 4	B4GALNT4	0,34
myosin VC	MYO5C	0,34
guanine nucleotide binding protein (G protein), gamma 4	GNG4	0,34
ubiquitin-like with PHD and ring finger domains 1	UHRF1	0,34
pellino E3 ubiquitin protein ligase 1	PELI1	0,34
lin-28 homolog B (C. elegans)	LIN28B	0,34
hydroxysteroid (17-beta) dehydrogenase 4	HSD17B4	0,34
protein phosphatase 4, regulatory subunit 4	PPP4R4	0,34
protein tyrosine phosphatase, non-receptor type 4 (megakaryocyte)	PTPN4	0,34
interferon regulatory factor 2 binding protein 2	IRF2BP2	0,34
golgin B1	GOLGB1	0,34
solute carrier family 9, subfamily A (NHE3, cation proton antiporter 3), member 3 regulator 1; microRNA 3615	SLC9A3R1; MIR3615	0,34
phosphorylase kinase, alpha 2 (liver)	PHKA2	0,34
mbt domain containing 1	MBTD1	0,34
NADH dehydrogenase (ubiquinone) Fe-S protein 7, 20kDa (NADH-coenzyme Q reductase)	NDUFS7	0,34

nudix hydrolase 16	NUDT16	0,34
uracil DNA glycosylase	UNG	0,33
protein S (alpha)	PROS1	0,33
epoxide hydrolase 2, cytoplasmic	EPHX2	0,33
cyclic nucleotide binding domain containing 2	CNBD2	0,33
glucuronidase, beta pseudogene 2	GUSBP2	0,33
selenoprotein N, 1	SEPN1	0,33
dipeptidase 1 (renal)	DPEP1	0,33
septin 6	sept-06	0,33
phosphorylase, glycogen, liver	PYGL	0,33
histone cluster 1, H2am; histone cluster 1, H3j	HIST1H2AM; HIST1H3J	0,33
nuclear receptor subfamily 0, group B, member 2	NR0B2	0,33
histone cluster 1, H3h	HIST1H3H	0,33
melanoma inhibitory activity 2	MIA2	0,33
zinc finger and BTB domain containing 12	ZBTB12	0,33
insulin-like growth factor 2; INS-IGF2 readthrough	IGF2; INS-IGF2	0,33
pregnancy-zone protein	PZP	0,33
zinc and ring finger 1, E3 ubiquitin protein ligase	ZNRF1	0,33
solute carrier family 19 (folate transporter), member 1	SLC19A1	0,33
alpha-2-glycoprotein 1, zinc-binding	AZGP1	0,33
EPH receptor B4	EPHB4	0,33
CD74 molecule, major histocompatibility complex, class II invariant chain	CD74	0,33
family with sequence similarity 3, member C	FAM3C	0,33
chromosome 11 open reading frame 71	C11orf71	0,33
solute carrier family 9, subfamily A (NHE3, cation proton antiporter 3), member 3 regulator 2	SLC9A3R2	0,33
inositol(myo)-1(or 4)-monophosphatase 2	IMPA2	0,33
prominin 1	PROM1	0,32
alkB homolog 7	ALKBH7	0,32
ST6 beta-galactosamide alpha-2,6-sialyltranferase 1	ST6GAL1	0,32
metaxin 2	MTX2	0,32
leucine-rich alpha-2-glycoprotein 1	LRG1	0,32
pre-B-cell leukemia homeobox 2	PBX2	0,32
histone cluster 1, H4a	HIST1H4A	0,32
ubiquitin specific peptidase 27, X-linked	USP27X	0,32
arylformamidase	AFMID	0,32
platelet-derived growth factor receptor-like	PDGFRL	0,32
chromosome 1 open reading frame 115	C1orf115	0,32
cyclin-dependent kinase 1	CDK1	0,32
protocadherin beta 13	PCDHB13	0,32
family with sequence similarity 208, member A	FAM208A	0,32
neighbor of BRCA1 gene 1	NBR1	0,32
zinc finger, CCHC domain containing 3	ZCCHC3	0,32

brain expressed X-linked 1	BEX1	0,32
choline kinase alpha	CHKA	0,32
phosphodiesterase 4D, cAMP-specific	PDE4D	0,32
tetraspanin 6	TSPAN6	0,32
family with sequence similarity 171, member A1	FAM171A1	0,32
period circadian clock 3	PER3	0,32
mesencephalic astrocyte-derived neurotrophic factor	MANF	0,32
transcriptional adaptor 1	TADA1	0,32
Jeck2013 ALT_ACCEPTOR, ALT_DONOR, coding, INTERNAL, intronic best transcript NM_145177	DHRSX	0,32
zinc finger protein 331	ZNF331	0,32
WD repeat domain 59	WDR59	0,32
aminolevulinate dehydratase	ALAD	0,32
prenylcysteine oxidase 1	PCYOX1	0,32
SH2 domain containing 4A	SH2D4A	0,32
transmembrane protein 53	TMEM53	0,32
secreted protein, acidic, cysteine-rich (osteonectin)	SPARC	0,32
	LRIG1	0,32
hexose-6-phosphate dehydrogenase (glucose 1-dehydrogenase)	H6PD	0,32
vacuolar protein sorting 54 homolog (S. cerevisiae)	VPS54	0,32
cell death-inducing DFFA-like effector b	CIDEB	0,32
ethanolaminephosphotransferase 1	EPT1	0,32
ectonucleotide pyrophosphatase/phosphodiesterase 1	ENPP1	0,32
acyl-CoA oxidase 2, branched chain	ACOX2	0,32
von Hippel-Lindau tumor suppressor, E3 ubiquitin protein ligase	VHL	0,31
Memczak2013 ANTISENSE, CDS, coding, INTERNAL best transcript NM_033133	CNP	0,31
pyruvate dehyrogenase phosphatase catalytic subunit 2	PDP2	0,31
histone cluster 1, H4c	HIST1H4C	0,31
sorbin and SH3 domain containing 3	SORBS3	0,31
C1q and tumor necrosis factor related protein 6	C1QTNF6	0,31
Memczak2013 ANTISENSE, CDS, coding, INTERNAL best transcript NM_003105	SORL1	0,31
endoplasmic reticulum aminopeptidase 1	ERAP1	0,31
agmatinase	AGMAT	0,31
SLC2A4 regulator	SLC2A4RG	0,31
BTB (POZ) domain containing 2	BTBD2	0,31
lectin, mannose-binding 2-like	LMAN2L	0,31
carboxypeptidase N, polypeptide 2	CPN2	0,31
transmembrane protein 120A	TMEM120A	0,31
biotinidase	BTD	0,31
1-acylglycerol-3-phosphate O-acyltransferase 3	AGPAT3	0,31
chromosome 1 open reading frame 131	C1orf131	0,31

calmodulin-like 4	CALML4	0,31
ornithine aminotransferase	OAT	0,31
SPARC related modular calcium binding 1	SMOC1	0,31
lipin 2	LPIN2	0,31
LDL receptor related protein 6	LRP6	0,31
sex determining region Y	SRY	0,31
polycomb group ring finger 5	PCGF5	0,31
protein tyrosine phosphatase, receptor type, F	PTPRF	0,31
histone cluster 1, H2bo	HIST1H2BO	0,31
phosphoribosyl pyrophosphate synthetase-associated protein 1	PRPSAP1	0,31
solute carrier family 44, member 3	SLC44A3	0,31
farnesyl-diphosphate farnesyltransferase 1	FDFT1	0,30
family with sequence similarity 83, member D	FAM83D	0,30
sperm associated antigen 5	SPAG5	0,30
progestin and adipoQ receptor family member VII	PAQR7	0,30
COX20 cytochrome c oxidase assembly factor	COX20	0,30
filamin A interacting protein 1-like	FILIP1L	0,30
leucine-rich repeats and immunoglobulin-like domains 1	LRIG1	0,30
retinol dehydrogenase 11 (all-trans/9-cis/11-cis)	RDH11	0,30
ilvB (bacterial acetolactate synthase)-like	ILVBL	0,30
interleukin 17 receptor B	IL17RB	0,30
regulatory factor X, 7	RFX7	0,30
solute carrier family 47 (multidrug and toxin extrusion), member 1	SLC47A1	0,30
aldehyde dehydrogenase 1 family, member B1	ALDH1B1	0,30
multiple inositol-polyphosphate phosphatase 1	MINPP1	0,30
macrophage stimulating 1-like	MST1L	0,30
thioredoxin domain containing 15	TXNDC15	0,30
histone cluster 1, H4b	HIST1H4B	0,30
Rh family, B glycoprotein (gene/pseudogene)	RHBG	0,30
calmin (calponin-like, transmembrane)	CLMN	0,30
collagen, type IV, alpha 5	COL4A5	0,30
LDL receptor related protein 6	LRP6	0,30
protein tyrosine phosphatase, receptor type, K	PTPRK	0,30
DNA damage inducible transcript 4	DDIT4	0,30
glycosylphosphatidylinositol specific phospholipase D1	GPLD1	0,30
activin A receptor type IIB	ACVR2B	0,30
glutaredoxin 5	GLRX5	0,30
cleavage and polyadenylation specific factor 4-like	CPSF4L	0,30
chromosome 10 open reading frame 82	C10orf82	0,30
cyclin-dependent kinase 2 associated protein 1	CDK2AP1	0,30
zinc finger protein 124	ZNF124	0,30

glucosamine (UDP-N-acetyl)-2-epimerase/N-acetylmannosamine kinase	GNE	0,30
Rho GTPase activating protein 18	ARHGAP18	0,29
ATP binding cassette subfamily B member 4	ABCB4	0,29
thioesterase superfamily member 6	THEM6	0,29
syntrophin, beta 1 (dystrophin-associated protein A1, 59kDa, basic component 1)	SNTB1	0,29
SEC13 homolog, nuclear pore and COPII coat complex component	SEC13	0,29
heparan sulfate (glucosamine) 3-O-sulfotransferase 3A1	HS3ST3A1	0,29
cingulin-like 1	CGNL1	0,29
solute carrier family 39 (zinc transporter), member 7	SLC39A7	0,29
tripartite motif containing 47	TRIM47	0,29
hepatocyte nuclear factor 4, gamma	HNF4G	0,29
angiotensinogen (serpin peptidase inhibitor, clade A, member 8)	AGT	0,29
transducin (beta)-like 1X-linked	TBL1X	0,29
alpha-2-HS-glycoprotein	AHSG	0,29
sorbin and SH3 domain containing 1	SORBS1	0,29
acyl-CoA synthetase long-chain family member 4	ACSL4	0,29
biphenyl hydrolase-like (serine hydrolase)	BPHL	0,29
solute carrier family 22 (organic cation transporter), member 3	SLC22A3	0,29
tripartite motif containing 15	TRIM15	0,29
ATP binding cassette subfamily B member 10	ABCB10	0,29
leukemia inhibitory factor receptor alpha	LIFR	0,29
butyrophilin, subfamily 3, member A3	BTN3A3	0,29
acyl-CoA synthetase short-chain family member 1	ACSS1	0,29
isochorismatase domain containing 2	ISOC2	0,29
NSL1, MIS12 kinetochore complex component	NSL1	0,29
solute carrier family 5 (sodium/myo-inositol cotransporter), member 3; mitochondrial ribosomal protein S6	SLC5A3; MRPS6	0,29
fucosyltransferase 11 (alpha (1,3) fucosyltransferase)	FUT11	0,29
family with sequence similarity 117, member A	FAM117A	0,29
Kazal-type serine peptidase inhibitor domain 1	KAZALD1	0,29
histone cluster 1, H2bl	HIST1H2BL	0,29
metastasis suppressor 1-like	MTSS1L	0,29
M-phase phosphoprotein 10 (U3 small nucleolar ribonucleoprotein)	MPHOSPH10	0,29
chromosome 14 open reading frame 28	C14orf28	0,28
transmembrane p24 trafficking protein 1	TMED1	0,28
patatin-like phospholipase domain containing 6	PNPLA6	0,28
placental growth factor	PGF	0,28

Cdk5 and Abl enzyme substrate 2	CABLES2	0,28
DEAQ box RNA-dependent ATPase 1	DQX1	0,28
complement component 4 binding protein, beta	C4BPB	0,28
solute carrier family 25 (mitochondrial carrier; citrate transporter), member 1	SLC25A1	0,28
creatine kinase, brain	CKB	0,28
pyruvate dehydrogenase (lipoamide) beta	PDHB	0,28
N-acylsphingosine amidohydrolase (non-lysosomal ceramidase) 2B	ASAH2B	0,28
transmembrane and tetratricopeptide repeat containing 4	TMTC4	0,28
zinc fingers and homeoboxes 1	ZHX1	0,28
PH domain and leucine rich repeat protein phosphatase 1	PHLPP1	0,28
PR domain containing 11	PRDM11	0,28
growth differentiation factor 7	GDF7	0,28
Ral GTPase activating protein, alpha subunit 1 (catalytic)	RALGAPA1	0,28
microRNA 4728; erb-b2 receptor tyrosine kinase 2	MIR4728; ERBB2	0,28
leucine-rich repeats and immunoglobulin-like domains 3	LRIG3	0,28
ketohexokinase	KHK	0,28
inter-alpha-trypsin inhibitor heavy chain 1	ITIH1	0,28
histone cluster 1, H3d; histone cluster 1, H2ad	HIST1H3D; HIST1H2AD	0,28
ras homolog family member U	RHOU	0,28
solute carrier family 7 (cationic amino acid transporter, y+ system), member 2	SLC7A2	0,28
tensin 3	TNS3	0,28
pyrroline-5-carboxylate reductase 1	PYCR1	0,28
ATP synthase, H+ transporting, mitochondrial F1 complex, delta subunit	ATP5D	0,28
procollagen C-endopeptidase enhancer	PCOLCE	0,28
phosphoinositide-3-kinase adaptor protein 1	PIK3AP1	0,28
apolipoprotein L1	APOL1	0,28
ZFP14 zinc finger protein	ZFP14	0,28
choline phosphotransferase 1	CHPT1	0,28
ras homolog family member U	RHOU	0,28
LDL receptor related protein 6	LRP6	0,28
24-dehydrocholesterol reductase	DHCR24	0,28
proline dehydrogenase (oxidase) 2	PRODH2	0,27
phospholipase A2, group IIA (platelets, synovial fluid)	PLA2G2A	0,27
family with sequence similarity 162, member A	FAM162A	0,27
high mobility group box 2	HMGB2	0,27
espin	ESPN	0,27

prune exopolyphosphatase	PRUNE	0,27
RAB33B, member RAS oncogene family	RAB33B	0,27
mannan-binding lectin serine peptidase 2	MASP2	0,27
cyclin F	CCNF	0,27
E2F transcription factor 8	E2F8	0,27
IQ motif containing GTPase activating protein 2	IQGAP2	0,27
protein disulfide isomerase family A, member 5; microRNA 7110	PDIA5; MIR7110	0,27
ribonucleoprotein, PTB-binding 2	RAVER2	0,27
Sp5 transcription factor	SP5	0,27
sulfotransferase family 1E member 1	SULT1E1	0,27
ATPase, H+ transporting V0 subunit e2	ATP6V0E2	0,27
X-box binding protein 1	XBP1	0,27
tubulin tyrosine ligase-like family member 11	TTLL11	0,27
fatty acid synthase	FASN	0,27
Transcript Identified by AceView, Entrez Gene ID(s) 6660	SOX5	0,27
protein disulfide isomerase family A, member 4	PDIA4	0,27
secretion regulating guanine nucleotide exchange factor	SERGEF	0,27
selenium binding protein 1	SELENBP1	0,27
cell adhesion molecule 1	CADM1	0,27
methylmalonic aciduria (cobalamin deficiency) cblC type, with homocystinuria	MMACHC	0,27
serine hydroxymethyltransferase 1 (soluble); microRNA 6778	SHMT1; MIR6778	0,27
emopamil binding protein (sterol isomerase)	EBP	0,27
Fc fragment of IgG binding protein	FCGBP	0,27
uncharacterized LOC389831; Salzman2013 ANNOTATED, CDS, coding, OVCODE, OVEXON, UTR3 best transcript NM_001242480; Salzman2013 ANNOTATED, CDS, coding, OVCODE, OVEXON, UTR3, UTR5 best transcript NM_001242480; Salzman2013 ANNOTATED, ncRNA, OVEXON best transcript TCONS_00030034; Salzman2013 ANNOTATED, ncRNA, OVCODE, OVEXON best transcript TCONS_00030034; Salzman2013 ALT_ACCEPTOR, ncRNA, OVCODE, OVEXON, upstream_start best transcript TCONS_00030034; Salzman2013 ALT_DONOR, downstream_end, ncRNA, OVEXON best transcript TCONS_00030035; Salzman2013 ALT_DONOR, coding, intronic, OVEXON, UTR5 best transcript NM_001242480; uncharacterized protein LOC389831 [Source:RefSeq peptide;Acc:NP_001229409]	LOC389831; TCONS_00030034 ; TCONS_00030035 ; AC011043.1	0,26
tetratricopeptide repeat domain 38	TTC38	0,26
histone cluster 1, H2bb	HIST1H2BB	0,26

eukaryotic elongation factor, selenocysteine-tRNA-specific	EEFSEC	0,26
spire-type actin nucleation factor 2	SPIRE2	0,26
insulin-like growth factor 2	IGF2	0,26
diazepam binding inhibitor (GABA receptor modulator, acyl-CoA binding protein)	DBI	0,26
par-6 family cell polarity regulator gamma	PARD6G	0,26
interferon regulatory factor 2 binding protein-like	IRF2BPL	0,26
transcription elongation factor A (SII), 3	TCEA3	0,26
topoisomerase (DNA) II alpha	TOP2A	0,26
sarcoglycan epsilon	SGCE	0,26
E2F transcription factor 1	E2F1	0,26
placenta specific 8	PLAC8	0,26
nephronectin	NPNT	0,26
transmembrane protein 97	TMEM97	0,26
cytochrome P450, family 8, subfamily B, polypeptide 1	CYP8B1	0,26
phospholipid scramblase 4	PLSCR4	0,26
transmembrane protein 64	TMEM64	0,26
Fraser extracellular matrix complex subunit 1	FRAS1	0,26
FK506 binding protein 11; ADP-ribosylation factor 3	FKBP11; ARF3	0,26
amine oxidase, copper containing 3	AOC3	0,26
aldehyde dehydrogenase 6 family, member A1	ALDH6A1	0,26
carbohydrate (chondroitin 4) sulfotransferase 12	CHST12	0,26
oligosaccharyltransferase complex subunit (non-catalytic)	OSTC	0,26
regulator of microtubule dynamics 2	RMDN2	0,26
MER proto-oncogene, tyrosine kinase	MERTK	0,26
H2A histone family, member Y2	H2AFY2	0,26
chromosome 5 open reading frame 15	C5orf15	0,26
collagen, type IX, alpha 3	COL9A3	0,25
connective tissue growth factor	CTGF	0,25
lanosterol synthase (2,3-oxidosqualene-lanosterol cyclase)	LSS	0,25
protein tyrosine kinase 7 (inactive)	PTK7	0,25
lamin B1	LMNB1	0,25
adenylate kinase 4	AK4	0,25
butyrophilin, subfamily 3, member A1	BTN3A1	0,25
zinc finger, DHHC-type containing 3	ZDHHC3	0,25
KIAA1161	KIAA1161	0,25
interferon-induced protein with tetratricopeptide repeats 1	IFIT1	0,25
KDEL (Lys-Asp-Glu-Leu) containing 1	KDELC1	0,25
glycolipid transfer protein domain containing 2	GLTPD2	0,25
interferon induced, with helicase C domain 1	IFIH1	0,25
isochorismatase domain containing 1	ISOC1	0,25

dual specificity phosphatase 16	DUSP16	0,25
family with sequence similarity 114, member A1	FAM114A1	0,25
alpha-1-microglobulin/bikunin precursor	AMBP	0,25
procollagen-lysine, 2-oxoglutarate 5-dioxygenase 2	PLOD2	0,25
Rho guanine nucleotide exchange factor 39	ARHGEF39	0,25
family with sequence similarity 101, member B	FAM101B	0,25
SMAD family member 9	SMAD9	0,25
cadherin 16, KSP-cadherin	CDH16	0,24
complement component 2	C2	0,24
zinc finger protein 704	ZNF704	0,24
kelch repeat and BTB (POZ) domain containing 7	KBTBD7	0,24
phosphatidylinositol glycan anchor biosynthesis class Z	PIGZ	0,24
orthodenticle homeobox 1	OTX1	0,24
phosphoenolpyruvate carboxykinase 1 (soluble)	PCK1	0,24
coagulation factor II (thrombin)	F2	0,24
zinc finger, MYM-type 3	ZMYM3	0,24
MAX dimerization protein 4; microRNA 4800	MXD4; MIR4800	0,24
transthyretin	TTR	0,24
mal, T-cell differentiation protein 2 (gene/pseudogene)	MAL2	0,24
pseudouridylate synthase 10	PUS10	0,24
macrophage stimulating 1	MST1	0,24
kininogen 1	KNG1	0,24
UDP glucuronosyltransferase 2 family, polypeptide A3	UGT2A3	0,24
thiosulfate sulfurtransferase (rhodanese)	TST	0,24
DAZ interacting zinc finger protein 1	DZIP1	0,24
glycerate kinase	GLYCTK	0,24
inhibin beta C	INHBC	0,24
prolyl 3-hydroxylase family member 4 (non-enzymatic)	P3H4	0,24
alveolar soft part sarcoma chromosome region, candidate 1	ASPSCR1	0,24
MLX interacting protein-like	MLXIPL	0,24
complement component 4B (Chido blood group)	C4B	0,24
complement component 4A (Rodgers blood group)	C4A	0,24
tumor necrosis factor receptor superfamily, member 19	TNFRSF19	0,24
histone cluster 2, H3a; histone cluster 2, H3c	HIST2H3A; HIST2H3C	0,24
apolipoprotein F	APOF	0,24
DNA (cytosine-5-)-methyltransferase 3 alpha	DNMT3A	0,24
family with sequence similarity 169, member A	FAM169A	0,24
StAR-related lipid transfer domain containing 4	STARD4	0,24
cyclin and CBS domain divalent metal cation transport mediator 3	CNNM3	0,24
sine oculis binding protein homolog	SOBP	0,24
isopentenyl-diphosphate delta isomerase 1	IDI1	0,23
isocitrate dehydrogenase 1 (NADP+)	IDH1	0,23

complement component 3	C3	0,23
serpin peptidase inhibitor, clade A (alpha-1 antiproteinase, antitrypsin), member 10	SERPINA10	0,23
proprotein convertase subtilisin/kexin type 6	PCSK6	0,23
alanyl (membrane) aminopeptidase	ANPEP	0,23
coiled-coil domain containing 92	CCDC92	0,23
regulatory factor X-associated protein	RFXAP	0,23
ubiquitin specific peptidase 41	USP41	0,23
stearoyl-CoA desaturase (delta-9-desaturase)	SCD	0,23
solute carrier family 2 (facilitated glucose transporter), member 2	SLC2A2	0,23
zinc finger, CCHC domain containing 2	ZCCHC2	0,23
coagulation factor X	F10	0,23
tripartite motif containing 74	TRIM74	0,23
transmembrane 7 superfamily member 2	TM7SF2	0,23
procollagen C-endopeptidase enhancer 2	PCOLCE2	0,23
paternally expressed 10	PEG10	0,23
Rab9 effector protein with kelch motifs	RABEPK	0,23
SEC11 homolog C, signal peptidase complex subunit	SEC11C	0,23
solute carrier family 39, member 11	SLC39A11	0,23
inositol 1,4,5-trisphosphate receptor, type 2	ITPR2	0,23
sperm associated antigen 4	SPAG4	0,23
BPI fold containing family B, member 1	BPIFB1	0,23
ADP-ribosylation factor like GTPase 15	ARL15	0,23
pipecolic acid oxidase	PIPOX	0,23
leucine rich repeat containing 3	LRRC3	0,23
prolactin receptor	PRLR	0,23
pyridoxal-dependent decarboxylase domain containing 2, pseudogene	PDXDC2P	0,23
von Willebrand factor A domain containing 1	VWA1	0,23
crystallin lambda 1	CRYL1	0,23
pyridoxal-dependent decarboxylase domain containing 1	PDXDC1	0,22
potassium channel, inwardly rectifying subfamily J, member 10	KCNJ10	0,22
ethanolamine-phosphate phospho-lyase	ETNPPL	0,22
transmembrane protein 141	TMEM141	0,22
Jeck2013 ANTISENSE, CDS, coding, INTERNAL, OVCODE, OVEXON, UTR3 best transcript NM_003542	HIST1H4C	0,22
histone cluster 2, H3a	HIST2H3A	0,22
glutathione peroxidase 7	GPX7	0,22
DEP domain containing 1B	DEPDC1B	0,22
annexin A13	ANXA13	0,22
poliovirus receptor-related 3	PVRL3	0,22
histone cluster 1, H2ab	HIST1H2AB	0,22
enoyl-CoA, hydratase/3-hydroxyacyl CoA dehydrogenase	EHHADH	0,22

insulin induced gene 2	INSIG2	0,22
glycosyltransferase 8 domain containing 1	GLT8D1	0,22
LDL receptor related protein 6	LRP6	0,22
fucosyltransferase 6 (alpha (1,3) fucosyltransferase)	FUT6	0,22
lipase A, lysosomal acid, cholesterol esterase	LIPA	0,22
transmembrane p24 trafficking protein 3	TMED3	0,22
Fc fragment of IgG, receptor, transporter, alpha	FCGRT	0,22
TOX high mobility group box family member 3	TOX3	0,22
KN motif and ankyrin repeat domains 1	KANK1	0,22
potassium channel tetramerization domain containing 3	KCTD3	0,22
solute carrier family 46 (folate transporter), member 1	SLC46A1	0,22
interleukin 1 receptor antagonist	IL1RN	0,22
catenin, beta interacting protein 1	CTNNBIP1	0,22
farnesyl diphosphate synthase	FDPS	0,22
glycogenin 2	GYG2	0,22
UDP glucuronosyltransferase 2 family, polypeptide B7	UGT2B7	0,22
cell adhesion associated, oncogene regulated	CDON	0,22
Memczak2013 ALT_ACCEPTOR, ALT_DONOR, coding, INTERNAL, intronic best transcript NM_001098207	HNRNPF	0,21
APC membrane recruitment protein 1	AMER1	0,21
feline leukemia virus subgroup C cellular receptor 1	FLVCR1	0,21
CTD small phosphatase 1	CTDSP1	0,21
glycosyltransferase-like 1B	GYLTL1B	0,21
solute carrier family 19 (thiamine transporter), member 2	SLC19A2	0,21
transmembrane protein 170B	TMEM170B	0,21
yippee like 2	YPEL2	0,21
non-SMC condensin I complex subunit D2	NCAPD2	0,21
sterol-C5-desaturase	SC5D	0,21
KIAA2013	KIAA2013	0,21
histone cluster 1, H4d	HIST1H4D	0,21
phospholipase A2, group IB (pancreas)	PLA2G1B	0,21
lipase maturation factor 1	LMF1	0,21
S-phase kinase-associated protein 2, E3 ubiquitin protein ligase	SKP2	0,21
phosphatidylinositol-specific phospholipase C, X domain containing 1	PLCXD1	0,21
cordon-bleu WH2 repeat protein like 1	COBLL1	0,21
LDL receptor related protein 3	LRP3	0,21
NEDD4 binding protein 2	N4BP2	0,21
microsomal glutathione S-transferase 2	MGST2	0,21
aarF domain containing kinase 3	ADCK3	0,21
phosphate cytidylyltransferase 2, ethanolamine	PCYT2	0,21
Memczak2013 ANTISENSE, CDS, coding, INTERNAL best transcript NM_005319	HIST1H1C	0,21

DNA-damage regulated autophagy modulator 1	DRAM1	0,21
glycerophosphodiester phosphodiesterase domain containing 5	GDPD5	0,21
GDP-mannose 4,6-dehydratase	GMDS	0,21
cytochrome b5 type A (microsomal)	CYB5A	0,21
retinoic acid receptor responder (tazarotene induced) 2	RARRES2	0,21
carboxylesterase 3; carboxylesterase 2	CES3; CES2	0,21
N-acetylneuraminic acid synthase	NANS	0,21
von Willebrand factor C and EGF domains	VWCE	0,21
interleukin 1 receptor, type I	IL1R1	0,21
ArfGAP with FG repeats 2	AGFG2	0,21
melanoma antigen family D4B; melanoma antigen family D4; small nucleolar RNA, H/ACA box 11D	MAGED4B; MAGED4; SNORA11D	0,20
TIMP metallopeptidase inhibitor 3	TIMP3	0,20
phosphoinositide-3-kinase, regulatory subunit 2 (beta); interferon, gamma-inducible protein 30	PIK3R2; IFI30	0,20
zinc finger, SWIM-type containing 5	ZSWIM5	0,20
Wolfram syndrome 1 (wolframin)	WFS1	0,20
plasminogen-like B2	PLGLB2	0,20
mannosyl (alpha-1,3-)-glycoprotein beta-1,4-N-acetylglucosaminyltransferase, isozyme A	MGAT4A	0,20
nuclear factor, erythroid 2	NFE2	0,20
hepatocellular carcinoma, down-regulated 1	HEPN1	0,20
solute carrier family 38, member 4	SLC38A4	0,20
guanine nucleotide binding protein (G protein), gamma 7	GNG7	0,20
receptor tyrosine kinase-like orphan receptor 1	ROR1	0,20
hepatocyte nuclear factor 4, alpha	HNF4A	0,20
APOBEC1 complementation factor	A1CF	0,20
ATPase, Cu++ transporting, beta polypeptide	ATP7B	0,20
melanoma antigen family D4; melanoma antigen family D4B; small nucleolar RNA, H/ACA box 11D; small nucleolar RNA, H/ACA box 11E	MAGED4; MAGED4B; SNORA11D; SNORA11E	0,20
unc-93 homolog A (C. elegans)	UNC93A	0,20
multivesicular body subunit 12B	MVB12B	0,20
butyrophilin, subfamily 3, member A2	BTN3A2	0,20
ADAM metallopeptidase domain 22	ADAM22	0,20
coiled-coil domain containing 92	CCDC92	0,20
tumor necrosis factor, alpha-induced protein 8-like 1	TNFAIP8L1	0,20
chemokine (C-C motif) ligand 20	CCL20	0,20
oxysterol binding protein 2	OSBP2	0,19
crystallin alpha A	CRYAA	0,19
acyl-CoA thioesterase 2	ACOT2	0,19

7-dehydrocholesterol reductase	DHCR7	0,19
RAB4A, member RAS oncogene family; S-phase response (cyclin related)	RAB4A; SPHAR	0,19
apolipoprotein B mRNA editing enzyme, catalytic polypeptide-like 3F	APOBEC3F	0,19
acyl-CoA thioesterase 1	ACOT1	0,19
peroxisome proliferator-activated receptor alpha	PPARA	0,19
forkhead box A3	FOXA3	0,19
ATP binding cassette subfamily G member 8	ABCG8	0,19
nucleoporin 210kDa	NUP210	0,19
tetratricopeptide repeat domain 21B	TTC21B	0,19
glycerol-3-phosphate acyltransferase, mitochondrial	GPAM	0,19
G protein-coupled receptor 160	GPR160	0,19
dopa decarboxylase	DDC	0,19
tocopherol (alpha) transfer protein	TTPA	0,19
sialidase 4	NEU4	0,19
glypican 6	GPC6	0,19
ADP-ribosyltransferase 4 (Dombrock blood group)	ART4	0,19
hepatic leukemia factor	HLF	0,19
interleukin 1 receptor accessory protein	IL1RAP	0,19
neuronal regeneration related protein	NREP	0,19
serpin peptidase inhibitor, clade D (heparin cofactor), member 1	SERPIND1	0,19
G protein-coupled receptor 119	GPR119	0,19
triokinase/FMN cyclase	TKFC	0,19
monocyte to macrophage differentiation-associated	MMD	0,19
ubiquitin specific peptidase 18	USP18	0,19
FXYD domain containing ion transport regulator 1	FXYD1	0,19
serpin peptidase inhibitor, clade C (antithrombin), member 1	SERPINC1	0,19
RAB11 family interacting protein 3 (class II)	RAB11FIP3	0,19
asparaginase like 1	ASRGL1	0,19
fatty acid hydroxylase domain containing 2	FAXDC2	0,19
fatty acid desaturase 2	FADS2	0,18
pyruvate kinase, liver and RBC	PKLR	0,18
ubiquitin D	UBD	0,18
matrilin 3	MATN3	0,18
KN motif and ankyrin repeat domains 4	KANK4	0,18
galactokinase 1	GALK1	0,18
phosphatidylethanolamine binding protein 1	PEBP1	0,18
regucalcin	RGN	0,18
scavenger receptor cysteine rich family, 4 domains	SSC4D	0,18
methylmalonyl CoA epimerase	MCEE	0,18
collagen, type XXVII, alpha 1	COL27A1	0,18
serum/glucocorticoid regulated kinase 1	SGK1	0,18

fibroblast growth factor receptor 4	FGFR4	0,18
LDL receptor related protein 6	LRP6	0,18
transmembrane protein 135	TMEM135	0,18
UDP glucuronosyltransferase 2 family, polypeptide B28	UGT2B28	0,18
myosin X	MYO10	0,18
coagulation factor V (proaccelerin, labile factor)	F5	0,18
carboxypeptidase N, polypeptide 1	CPN1	0,18
transmembrane protein 37	TMEM37	0,18
growth hormone regulated TBC protein 1	GRTP1	0,18
phenylalanine hydroxylase	PAH	0,18
sema domain, transmembrane domain (TM), and cytoplasmic domain, (semaphorin) 6B	SEMA6B	0,18
GATA binding protein 4	GATA4	0,18
electron-transferring-flavoprotein dehydrogenase	ETFDH	0,18
delta-like 1 homolog (Drosophila)	DLK1	0,18
cytochrome P450, family 51, subfamily A, polypeptide 1	CYP51A1	0,17
phosphatidylinositol-specific phospholipase C, X domain containing 1	PLCXD1	0,17
RAB11 family interacting protein 4 (class II); microRNA 4724	RAB11FIP4; MIR4724	0,17
protein phosphatase, Mg2+/Mn2+ dependent, 1H	PPM1H	0,17
melanocortin 2 receptor accessory protein 2	MRAP2	0,17
coagulation factor X	F10	0,17
histone cluster 2, H4a; histone cluster 2, H4b	HIST2H4A; HIST2H4B	0,17
glutamate dehydrogenase 2	GLUD2	0,17
complement component 6	C6	0,17
peroxisome proliferator-activated receptor gamma, coactivator 1 alpha	PPARGC1A	0,17
solute carrier family 6 (neurotransmitter transporter), member 12	SLC6A12	0,17
kinesin family member 20A	KIF20A	0,17
angiopoietin like 8	ANGPTL8	0,17
SRY box 9	SOX9	0,17
smoothened, frizzled class receptor	SMO	0,17
ankyrin repeat domain 54; microRNA 658	ANKRD54; MIR658	0,17
histone cluster 2, H4b; histone cluster 2, H4a	HIST2H4B; HIST2H4A	0,17
cAMP responsive element binding protein 3-like 3	CREB3L3	0,17
aminoadipate-semialdehyde synthase	AASS	0,17
lysophosphatidic acid receptor 6	LPAR6	0,17
glutamate dehydrogenase 1	GLUD1	0,17
glycoprotein hormone alpha 2	GPHA2	0,16
vasorin	VASN	0,16

NDRG family member 2; microRNA 6717	NDRG2; MIR6717	0,16
coenzyme Q10A	COQ10A	0,16
heparan sulfate (glucosamine) 3-O-sulfotransferase 3B1	HS3ST3B1	0,16
heparan sulfate 6-O-sulfotransferase 1	HS6ST1	0,16
complement factor B	CFB	0,16
chromosome 2 open reading frame 72	C2orf72	0,16
5-hydroxytryptamine (serotonin) receptor 3B, ionotropic	HTR3B	0,16
complement component 7	C7	0,16
MAGE family member D1	MAGED1	0,16
3-hydroxy-3-methylglutaryl-CoA reductase	HMGCR	0,16
solute carrier family 17, member 2	SLC17A2	0,16
complement component 4 binding protein, alpha	C4BPA	0,16
pleckstrin homology domain containing, family B (evectins) member 1	PLEKHB1	0,16
solute carrier family 16 (aromatic amino acid transporter), member 10	SLC16A10	0,16
glutathione S-transferase alpha 1	GSTA1	0,16
inhibitor of kappa light polypeptide gene enhancer in B-cells, kinase complex-associated protein	IKBKAP	0,16
coagulation factor X	F10	0,16
tet methylcytosine dioxygenase 1	TET1	0,15
transforming, acidic coiled-coil containing protein 1	TACC1	0,15
neuroligin 4, Y-linked	NLGN4Y	0,15
sema domain, immunoglobulin domain (Ig), transmembrane domain (TM) and short cytoplasmic domain, (semaphorin) 4G	SEMA4G	0,15
gap junction protein beta 1	GJB1	0,15
sodium channel, voltage gated, type IX alpha subunit	SCN9A	0,15
solute carrier family 39 (zinc transporter), member 5	SLC39A5	0,15
CDC42 effector protein (Rho GTPase binding) 4	CDC42EP4	0,15
mevalonate kinase	MVK	0,15
solute carrier family 39 (zinc transporter), member 14	SLC39A14	0,15
SRY box 5	SOX5	0,15
carbohydrate (chondroitin 4) sulfotransferase 13	CHST13	0,15
colorectal cancer associated 2	COLCA2	0,15
2,3-cyclic nucleotide 3 phosphodiesterase	CNP	0,15
angiotensin II receptor, type 1	AGTR1	0,15
angiopoietin like 1	ANGPTL1	0,15
mitochondrial amidoxime reducing component 1	MARC1	0,15
tumor necrosis factor (ligand) superfamily, member 10	TNFSF10	0,15
syndecan 2	SDC2	0,15
GIPC PDZ domain containing family, member 2	GIPC2	0,14
Ca++-dependent secretion activator 2	CADPS2	0,14
choline phosphotransferase 1	CHPT1	0,14

ephrin-A1	EFNA1	0,14
dual specificity phosphatase 9	DUSP9	0,14
chromosome 11 open reading frame 54	C11orf54	0,14
phosphoglucomutase 1	PGM1	0,14
fibrinogen alpha chain	FGA	0,14
apolipoprotein M	APOM	0,14
ribosomal protein S6 kinase, 90kDa, polypeptide 3	RPS6KA3	0,14
Memczak2013 ANTISENSE, coding, INTERNAL, UTR3 best transcript NM_002567	PEBP1	0,14
3-hydroxy-3-methylglutaryl-CoA synthase 1 (soluble)	HMGCS1	0,14
hydroxyacyl-CoA dehydrogenase	HADH	0,14
guanidinoacetate N-methyltransferase	GAMT	0,14
sarcosine dehydrogenase	SARDH	0,14
DAB2 interacting protein	DAB2IP	0,14
reelin	RELN	0,14
acyl-CoA dehydrogenase, short/branched chain	ACADSB	0,14
sorbin and SH3 domain containing 2	SORBS2	0,14
scavenger receptor class A, member 3	SCARA3	0,14
solute carrier family 19 (thiamine transporter), member 3	SLC19A3	0,14
aldolase C, fructose-bisphosphate	ALDOC	0,13
amidohydrolase domain containing 1	AMDHD1	0,13
methylmalonic aciduria (cobalamin deficiency) cblB type	MMAB	0,13
FK506 binding protein 14	FKBP14	0,13
membrane protein, palmitoylated 7	MPP7	0,13
apolipoprotein A-I	APOA1	0,13
vav 3 guanine nucleotide exchange factor	VAV3	0,13
marker of proliferation Ki-67	MKI67	0,13
vitronectin	VTN	0,13
obscurin-like 1	OBSL1	0,13
solute carrier family 25, member 42	SLC25A42	0,13
mitogen-activated protein kinase kinase 6	MAP2K6	0,13
frizzled class receptor 4	FZD4	0,13
ectonucleoside triphosphate diphosphohydrolase 7	ENTPD7	0,13
mevalonate (diphospho) decarboxylase	MVD	0,13
tripartite motif containing 73	TRIM73	0,13
prolyl 3-hydroxylase 1	P3H1	0,13
hepcidin antimicrobial peptide	HAMP	0,13
transmembrane protein 45A	TMEM45A	0,13
tubulin, beta 1 class VI	TUBB1	0,13
villin 1	VIL1	0,12
aldehyde dehydrogenase 18 family, member A1	ALDH18A1	0,12
family with sequence similarity 20, member C	FAM20C	0,12
sterol O-acyltransferase 2	SOAT2	0,12

heparan sulfate (glucosamine) 3-O-sulfotransferase 5	HS3ST5	0,12
erythropoietin	EPO	0,12
anthrax toxin receptor 1	ANTXR1	0,12
angiogenin, ribonuclease, RNase A family, 5; ribonuclease, RNase A family, 4	ANG; RNASE4	0,12
phosphatidylinositol-4-phosphate 5-kinase, type I, beta	PIP5K1B	0,12
membrane bound O-acyltransferase domain containing 4	MBOAT4	0,12
ELOVL fatty acid elongase 2	ELOVL2	0,12
alpha-fetoprotein	AFP	0,12
solute carrier family 2 (facilitated glucose transporter), member 10	SLC2A10	0,12
methyltransferase like 7A	METTL7A	0,12
argininosuccinate synthase 1	ASS1	0,12
phospholipid phosphatase related 1	PLPPR1	0,12
sortilin-related receptor, L(DLR class) A repeats containing	SORL1	0,11
pantothenate kinase 1	PANK1	0,11
carboxypeptidase B2 (plasma)	CPB2	0,11
erb-b2 receptor tyrosine kinase 3	ERBB3	0,11
immunoglobulin superfamily, DCC subclass, member 3	IGDCC3	0,11
lecithin-cholesterol acyltransferase	LCAT	0,11
hydroxysteroid (11-beta) dehydrogenase 2	HSD11B2	0,11
LY75-CD302 readthrough; CD302 molecule; lymphocyte antigen 75	LY75-CD302; CD302; LY75	0,11
family with sequence similarity 20, member A	FAM20A	0,11
N-acetylated alpha-linked acidic dipeptidase 2	NAALAD2	0,11
carbonic anhydrase VA, mitochondrial	CA5A	0,11
cyclin and CBS domain divalent metal cation transport mediator 1	CNNM1	0,11
NAD kinase 2, mitochondrial	NADK2	0,11
solute carrier family 6 (neurotransmitter transporter, glycine), member 9	SLC6A9	0,11
squalene epoxidase	SQLE	0,11
glutamic pyruvate transaminase (alanine aminotransferase) 2	GPT2	0,11
complement component 5	C5	0,11
solute carrier family 10, member 5	SLC10A5	0,11
coagulation factor VII (serum prothrombin conversion accelerator)	F7	0,11
tetratricopeptide repeat domain 39B	TTC39B	0,11
docking protein 6	DOK6	0,10
alpha-2-macroglobulin	A2M	0,10
3-hydroxybutyrate dehydrogenase, type 1	BDH1	0,10
DIX domain containing 1	DIXDC1	0,10

hepatic and glial cell adhesion molecule	HEPACAM	0,10
testis-specific kinase 2	TESK2	0,10
deiodinase, iodothyronine, type I	DIO1	0,10
small leucine-rich protein 1	SMLR1	0,10
hydroxysteroid (17-beta) dehydrogenase 7	HSD17B7	0,10
hemopexin	HPX	0,10
complement factor I	CFI	0,10
acetyl-CoA acetyltransferase 2	ACAT2	0,10
UDP glucuronosyltransferase 2 family, polypeptide B11	UGT2B11	0,10
sodium channel, voltage gated, type I alpha subunit	SCN1A	0,10
asialoglycoprotein receptor 1	ASGR1	0,10
G protein-coupled estrogen receptor 1	GPER1	0,10
solute carrier family 25 (pyrimidine nucleotide carrier), member 33	SLC25A33	0,10
protein Z, vitamin K-dependent plasma glycoprotein	PROZ	0,10
6-phosphofructo-2-kinase/fructose-2,6-biphosphatase 4; microRNA 6823	PFKFB4; MIR6823	0,10
beta-1,4-glucuronyltransferase 1	B4GAT1	0,10
lipase, hepatic	LIPC	0,09
fibroblast growth factor receptor 3	FGFR3	0,09
nidogen 1	NID1	0,09
glycoprotein 2 (zymogen granule membrane)	GP2	0,09
family with sequence similarity 3, member B	FAM3B	0,09
albumin	ALB	0,09
phospholipase A2, group XIIB	PLA2G12B	0,09
liver expressed antimicrobial peptide 2	LEAP2	0,09
transforming growth factor beta receptor III	TGFBR3	0,09
SRY box 6; microRNA 6073	SOX6; MIR6073	0,08
iroquois homeobox 3	IRX3	0,08
UDP glucuronosyltransferase 2 family, polypeptide B4	UGT2B4	0,08
acyl-CoA synthetase medium-chain family member 2B	ACSM2B	0,08
RAB17, member RAS oncogene family	RAB17	0,08
hematopoietically expressed homeobox	HHEX	0,08
N-acylsphingosine amidohydrolase (non-lysosomal ceramidase) 2	ASAH2	0,08
fibrinogen gamma chain	FGG	0,08
MPV17 mitochondrial membrane protein-like	MPV17L	0,08
mannan-binding lectin serine peptidase 1 (C4/C2 activating component of Ra-reactive factor)	MASP1	0,08
transferrin	TF	0,08
alcohol dehydrogenase 6 (class V)	ADH6	0,08
mitochondrial pyruvate carrier 1	MPC1	0,08
ectonucleotide pyrophosphatase/phosphodiesterase 2	ENPP2	0,08
chromosome 10 open reading frame 10	C10orf10	0,08
paternally expressed 3; zinc finger, imprinted 2	PEG3; ZIM2	0,07

UDP glucuronosyltransferase 2 family, polypeptide B10	UGT2B10	0,07
tescalcin	TESC	0,07
crystallin alpha A	CRYAA	0,07
inter-alpha-trypsin inhibitor heavy chain 3	ITIH3	0,07
growth arrest-specific 2	GAS2	0,07
plasminogen-like B1; plasminogen-like B2	PLGLB1; PLGLB2	0,07
progestin and adipoQ receptor family member VIII	PAQR8	0,07
serpin peptidase inhibitor, clade A (alpha-1 antiproteinase, antitrypsin), member 3	SERPINA3	0,06
flavin containing monooxygenase 5	FMO5	0,06
basal cell adhesion molecule (Lutheran blood group)	BCAM	0,06
hepsin	HPN	0,06
aldo-keto reductase family 1, member D1	AKR1D1	0,06
solute carrier family 5 (sodium/sugar cotransporter), member 9	SLC5A9	0,06
complement component 8, alpha polypeptide	C8A	0,06
solute carrier family 13 (sodium-dependent citrate transporter), member 5	SLC13A5	0,05
kelch-like family member 14	KLHL14	0,05
ectonucleotide pyrophosphatase/phosphodiesterase 3	ENPP3	0,05
glucagon-like peptide 2 receptor	GLP2R	0,05
growth regulation by estrogen in breast cancer 1	GREB1	0,05
fibrinogen beta chain	FGB	0,05
proprotein convertase subtilisin/kexin type 9	PCSK9	0,05
long intergenic non-protein coding RNA 668	LINC00668	0,05
fibrinogen like 1	FGL1	0,05
endoplasmic reticulum protein 27	ERP27	0,05
progestin and adipoQ receptor family member IX	PAQR9	0,05
serpin peptidase inhibitor, clade A (alpha-1 antiproteinase, antitrypsin), member 11	SERPINA11	0,05
patatin-like phospholipase domain containing 3	PNPLA3	0,05
FRAS1 related extracellular matrix 1	FREM1	0,04
one cut homeobox 1	ONECUT1	0,04
solute carrier family 22 (organic anion transporter), member 9	SLC22A9	0,04
selenoprotein P, plasma, 1	SEPP1	0,04
serpin peptidase inhibitor, clade A (alpha-1 antiproteinase, antitrypsin), member 6	SERPINA6	0,04
alanine-glyoxylate aminotransferase	AGXT	0,04
mannose-binding lectin (protein C) 2, soluble	MBL2	0,04
serpin peptidase inhibitor, clade A (alpha-1 antiproteinase, antitrypsin), member 4	SERPINA4	0,04
Mix paired-like homeobox	MIXL1	0,04
diacylglycerol kinase, kappa	DGKK	0,04

solute carrier organic anion transporter family, member 4C1	SLCO4C1	0,04
chemokine (C-X3-C motif) ligand 1	CX3CL1	0,04
haptoglobin	HP	0,04
solute carrier family 38, member 11	SLC38A11	0,04
haptoglobin-related protein	HPR	0,03
plasminogen	PLG	0,03
solute carrier family 30, member 10	SLC30A10	0,03
acyl-CoA synthetase medium-chain family member 3	ACSM3	0,03
apolipoprotein C-III	APOC3	0,03
BPI fold containing family B, member 2	BPIFB2	0,03
serpin peptidase inhibitor, clade F (alpha-2 antiplasmin, pigment epithelium derived factor), member 2	SERPINF2	0,03
immunoglobulin superfamily, member 1	IGSF1	0,02
solute carrier family 29 (equilibrative nucleoside transporter), member 4	SLC29A4	0,02
coagulation factor XI	F11	0,02
microsomal triglyceride transfer protein	MTTP	0,02
glucose-6-phosphatase, catalytic subunit	G6PC	0,02
nuclear receptor subfamily 1, group H, member 4	NR1H4	0,02
ceruloplasmin (ferroxidase)	CP	0,02
lysyl oxidase	LOX	0,02
serpin peptidase inhibitor, clade A (alpha-1 antiproteinase, antitrypsin), member 5	SERPINA5	0,02
progastricsin (pepsinogen C)	PGC	0,02
fatty acid binding protein 1, liver	FABP1	0,02
serpin peptidase inhibitor, clade A (alpha-1 antiproteinase, antitrypsin), member 7	SERPINA7	0,01
sulfotransferase family 2A member 1	SULT2A1	0,01
odontogenic, ameloblast asssociated	ODAM	0,01
homeobox D1; microRNA 7704	HOXD1; MIR7704	0,01
klotho beta	KLB	0,01
solute carrier family 6 (amino acid transporter), member 14	SLC6A14	0,00
