# Supplementary figures and images for: A Multi-Omics Approach to Evaluate the Toxicity Mechanisms Associated with Silver Nanoparticles Exposure
Source: Nanomaterials (Basel). 2022 May 22;12(10):1762. doi: 10.3390/nano12101762 (PMC9146515; doi:10.3390/nano12101762)

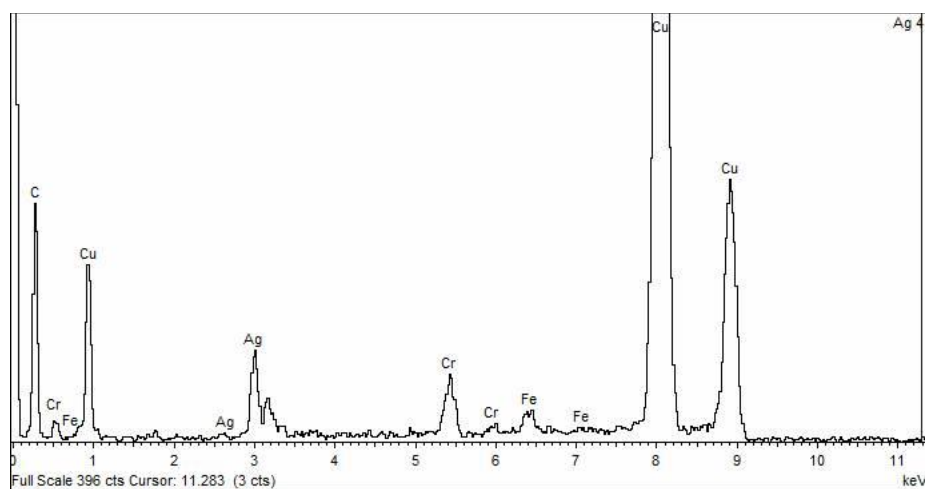

Figure S1. EDX spectra obtained for the 10 nm AgNPs.

Supplement: Supplementary file 1 [file nanomaterials-12-01762-s001.zip › Figure S1.pdf]

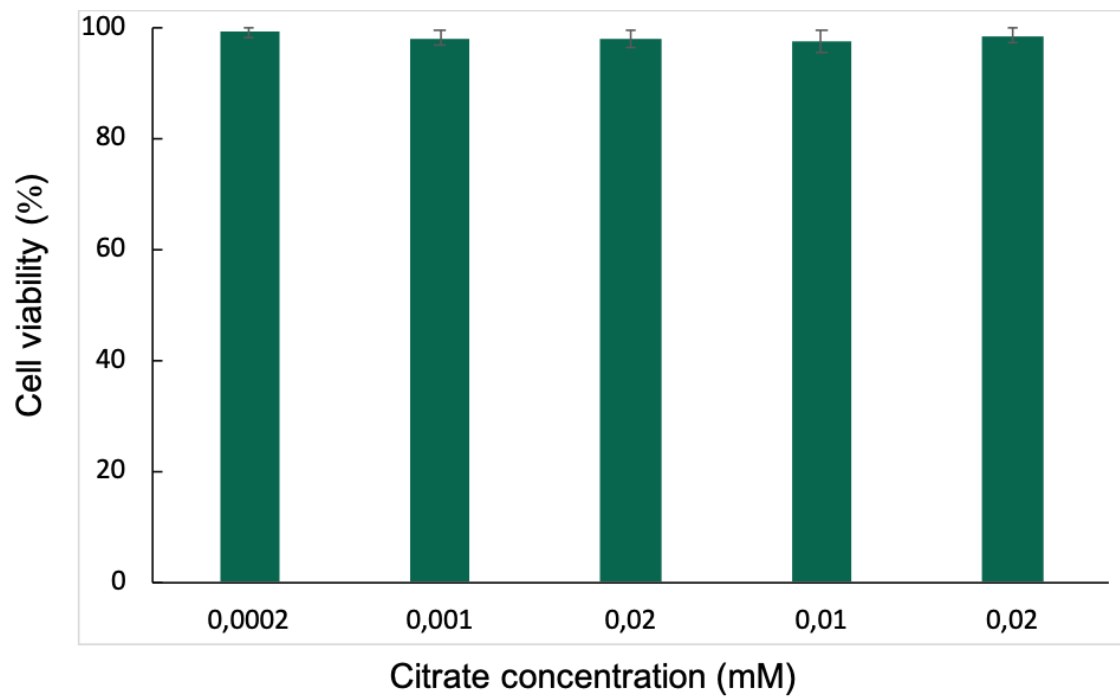

Figure S2. MTT-based cell viability assay of HepG2 cells exposed to citrate

Supplement: Supplementary file 1 [file nanomaterials-12-01762-s001.zip › Figure S2.pdf]
